# Supplementary figures and images for: The Slingshot phosphatase 2 is required for acrosome biogenesis during spermatogenesis in mice (part 1 of 4)
Source: eLife. 2023 Mar 21;12:e83129. doi: 10.7554/eLife.83129 (PMC10065795; doi:10.7554/eLife.83129)

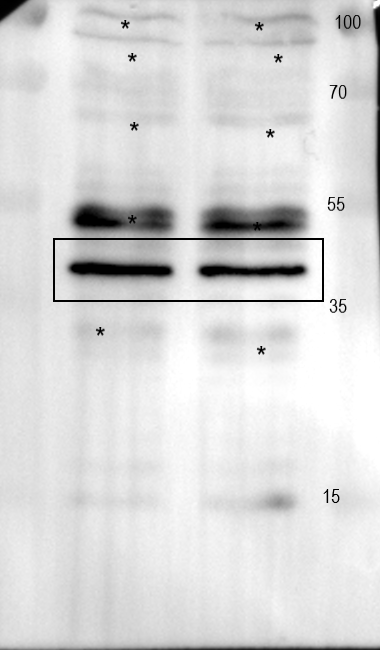

Supplement: Figure 1—source data 1. [file elife-83129-fig1-data1.zip › Figure 1/Labelled blots of Figure1B-GAPDH.Tif]

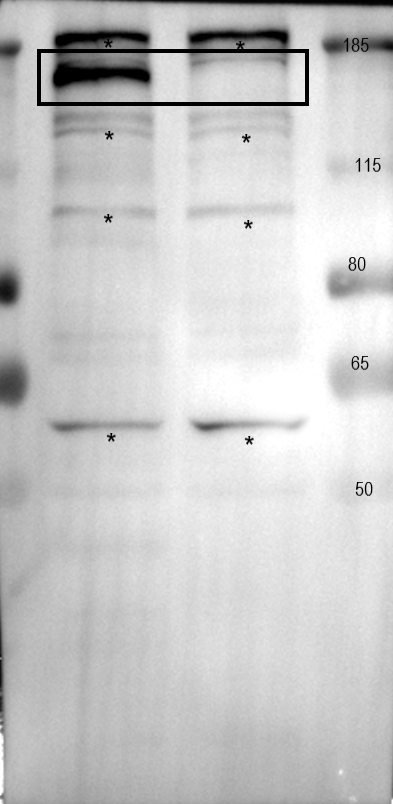

Supplement: Figure 1—source data 1. [file elife-83129-fig1-data1.zip › Figure 1/Labelled blots of Figure1B-SSH2.Tif]

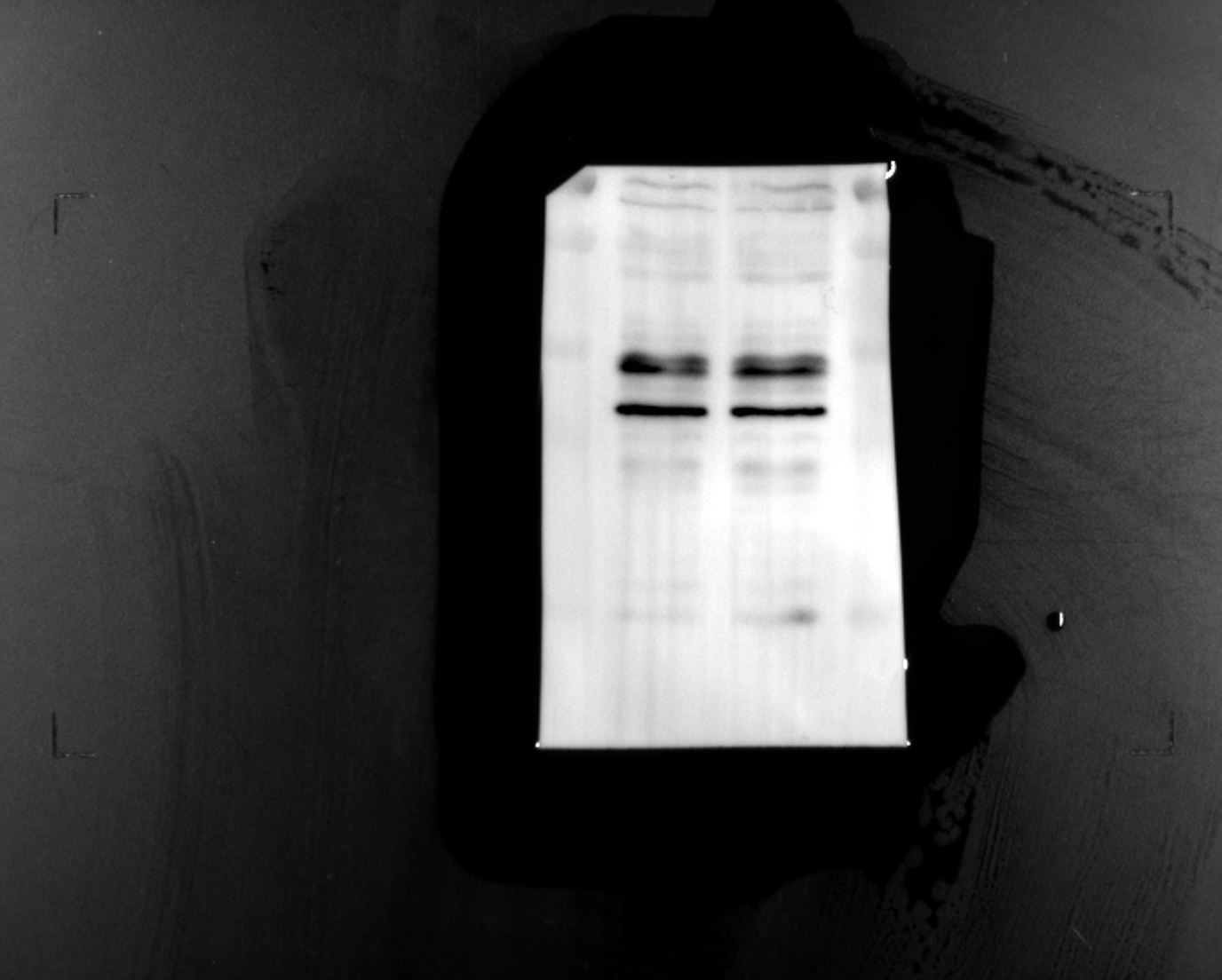

Supplement: Figure 1—source data 1. [file elife-83129-fig1-data1.zip › Figure 1/Raw blots of Figure1B-GAPDH.Tif]

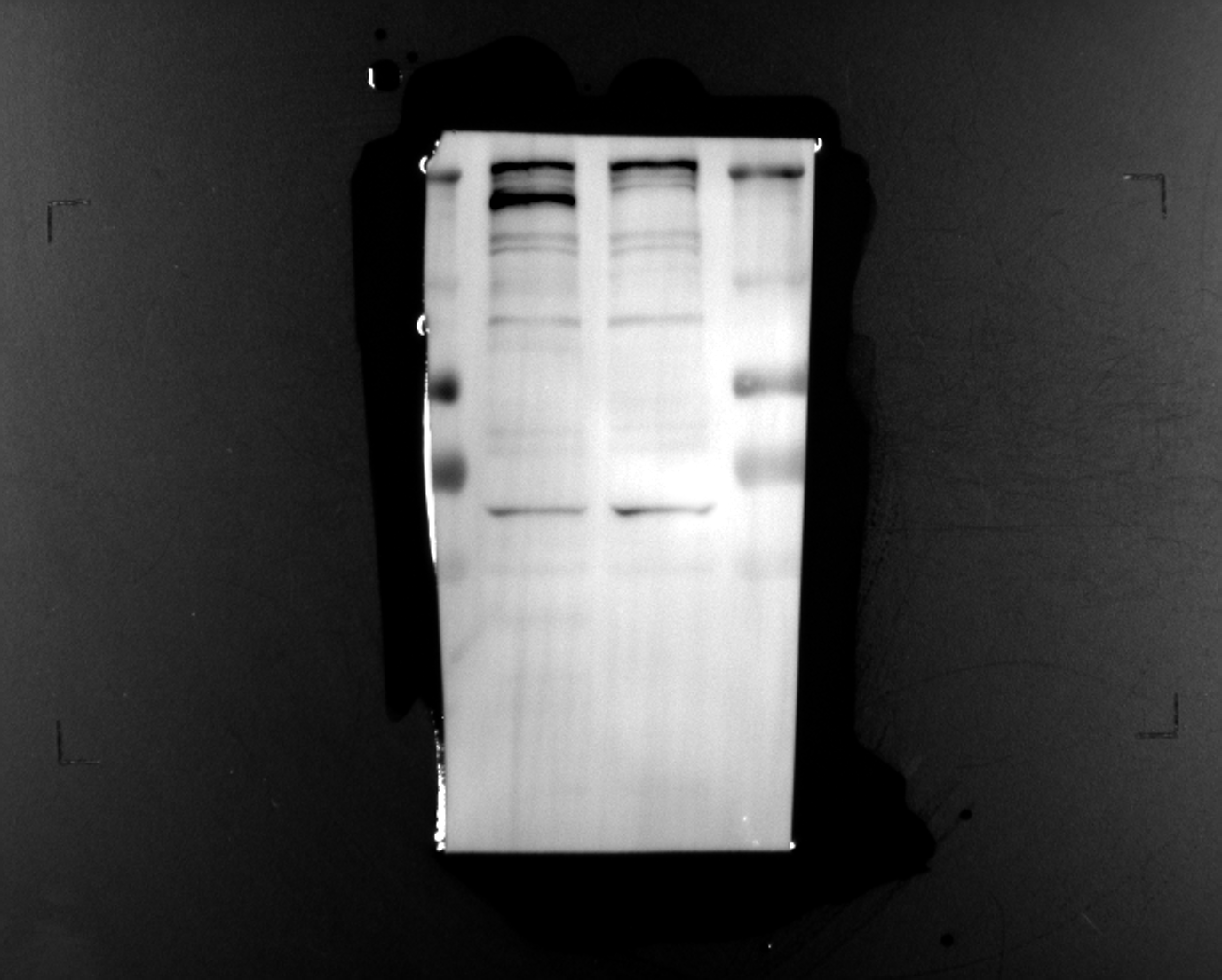

Supplement: Figure 1—source data 1. [file elife-83129-fig1-data1.zip › Figure 1/Raw blots of Figure1B-SSH2.Tif]

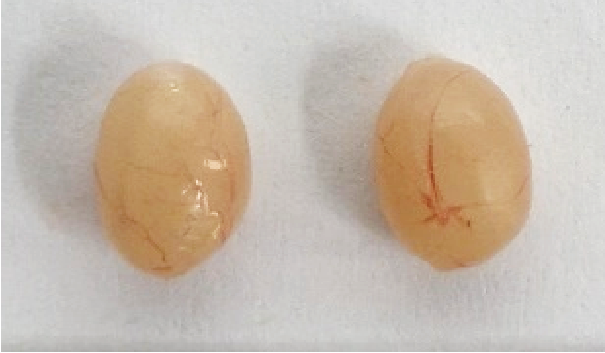

Supplement: Figure 1—source data 1. [file elife-83129-fig1-data1.zip › Figure 1/Source data of Figure1D.png]

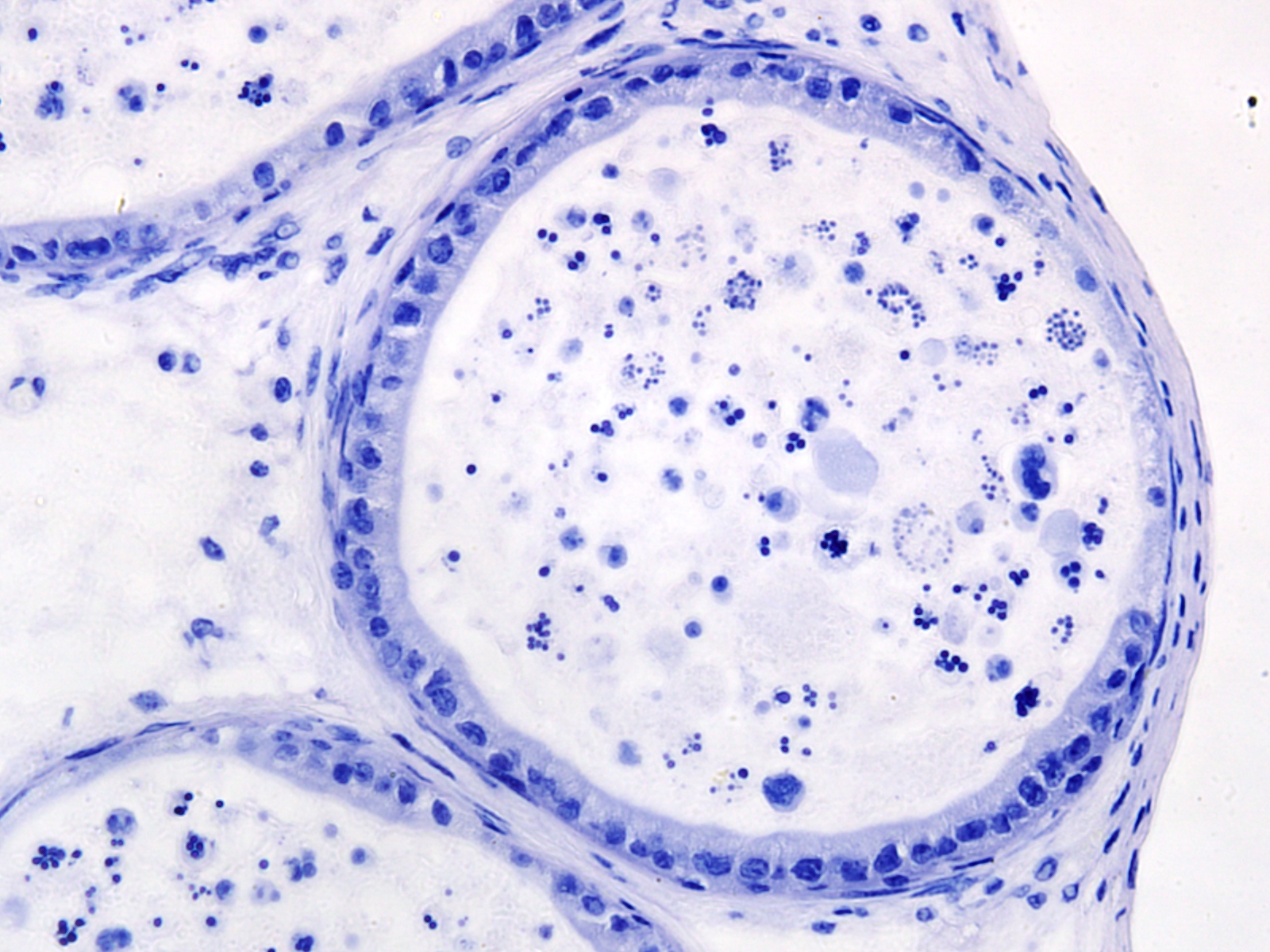

Supplement: Figure 1—source data 1. [file elife-83129-fig1-data1.zip › Figure 1/Source data of Figure1H/KO-epi.jpg]

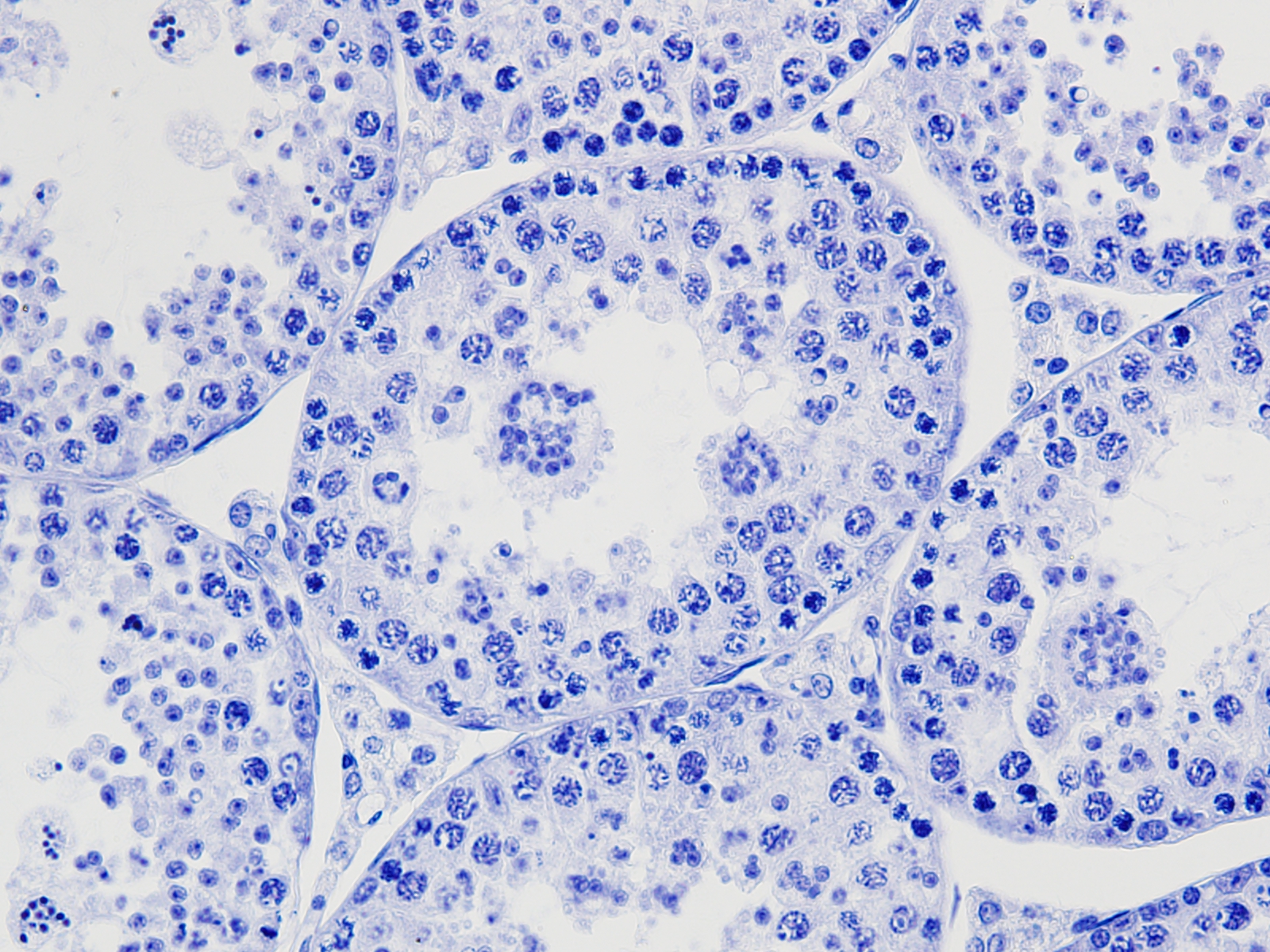

Supplement: Figure 1—source data 1. [file elife-83129-fig1-data1.zip › Figure 1/Source data of Figure1H/KO-testis.jpg]

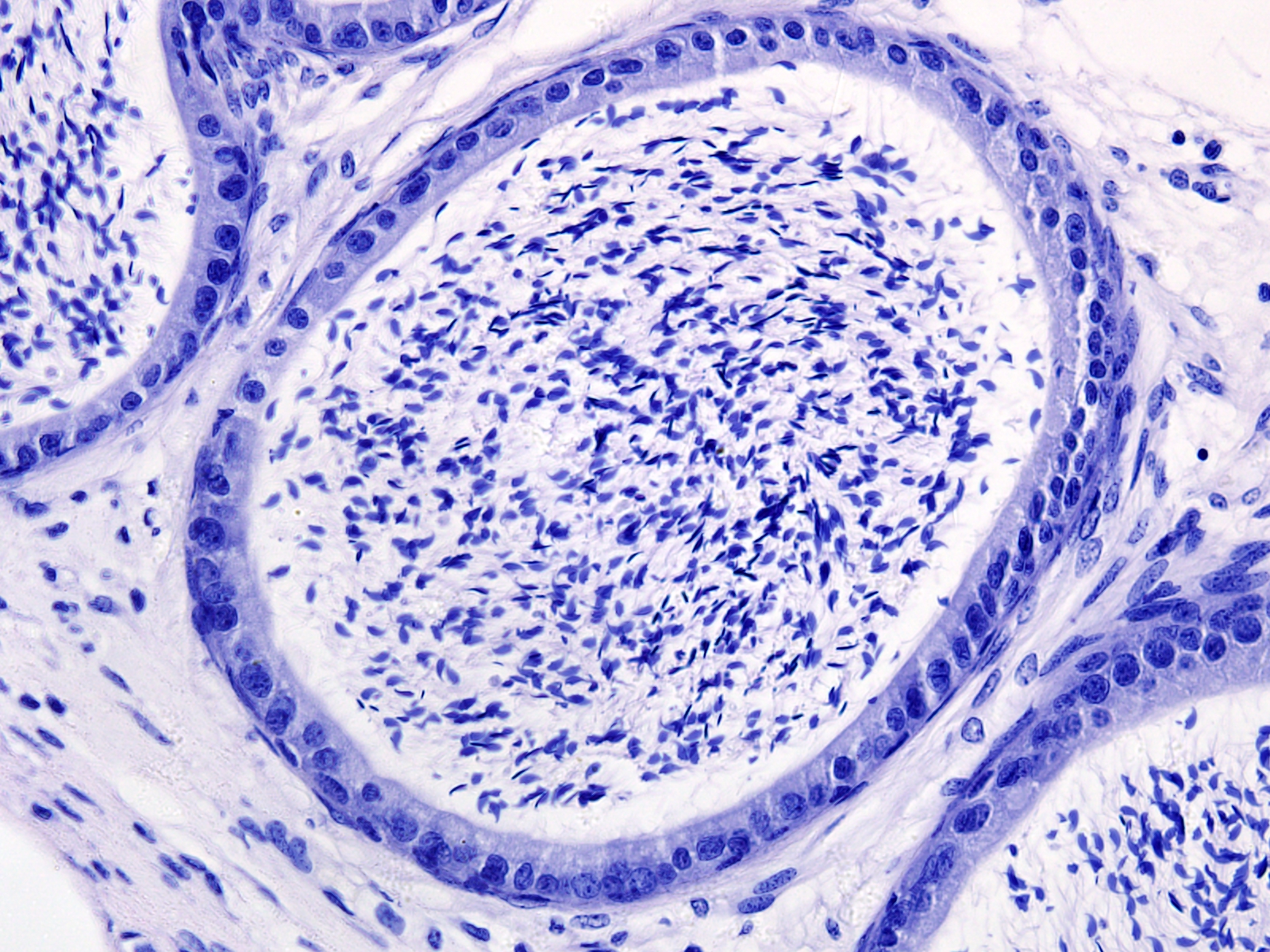

Supplement: Figure 1—source data 1. [file elife-83129-fig1-data1.zip › Figure 1/Source data of Figure1H/WT-epi.jpg]

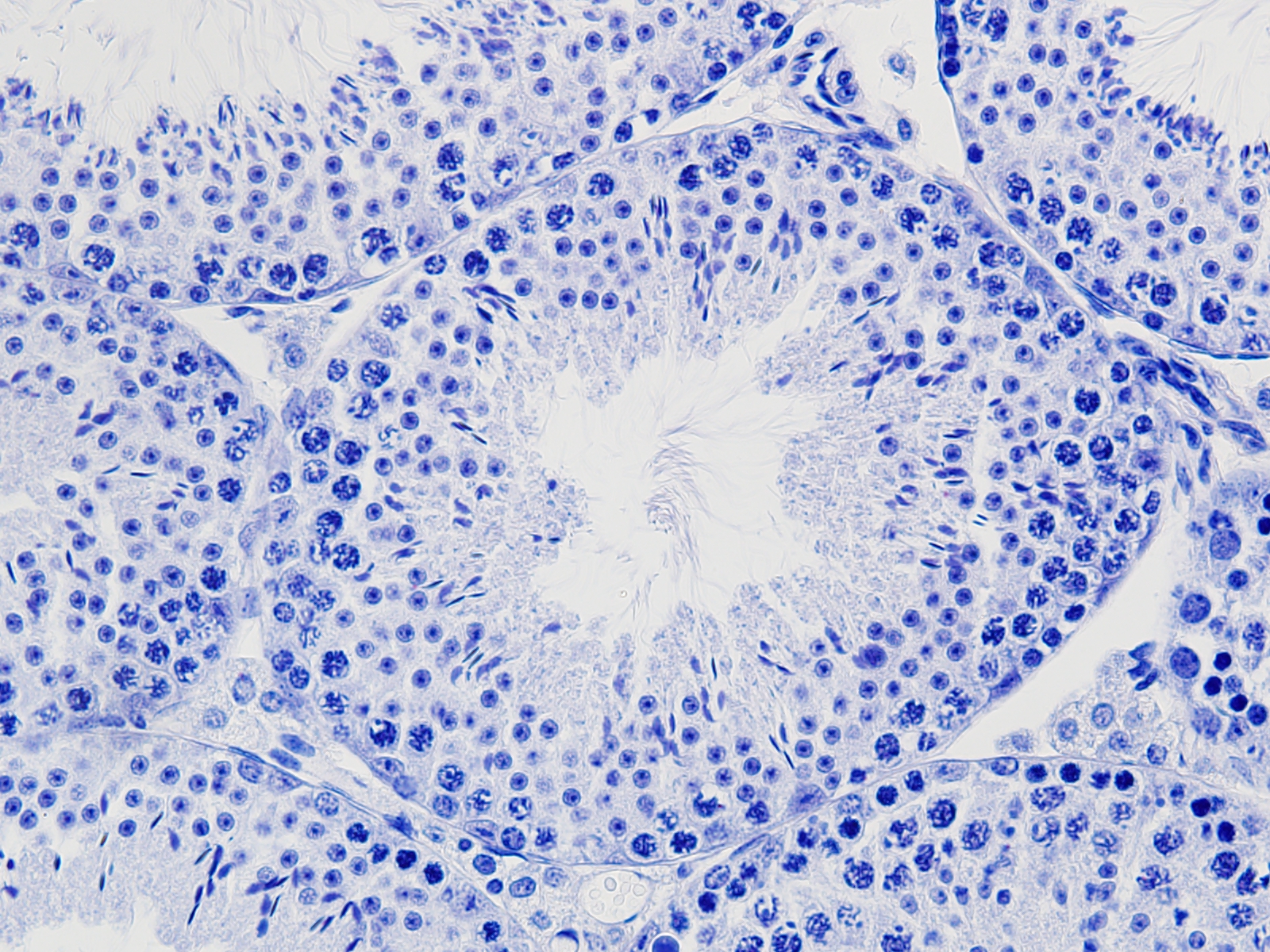

Supplement: Figure 1—source data 1. [file elife-83129-fig1-data1.zip › Figure 1/Source data of Figure1H/WT-testis.jpg]

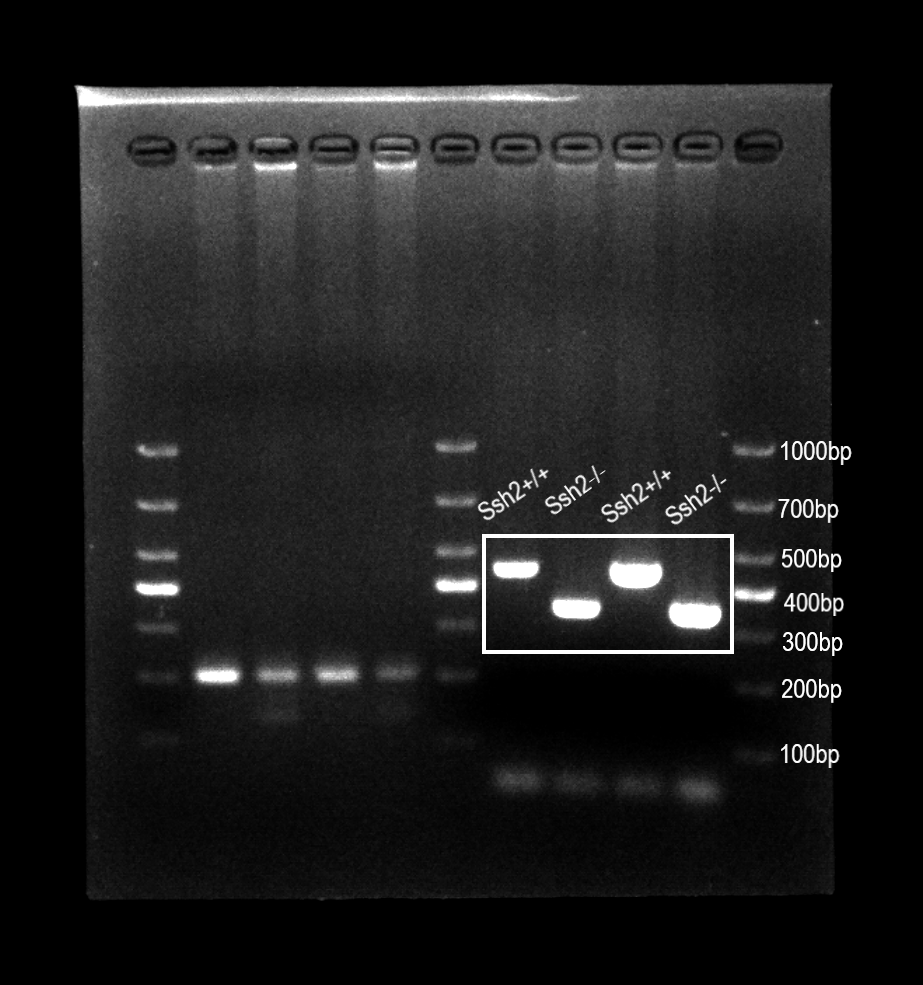

Supplement: Figure 1—figure supplement 1—source data 1. [file elife-83129-fig1-figsupp1-data1.zip › Figure supplement S1-source data 8/Labelled gels of Figure supplement S1.tiff]

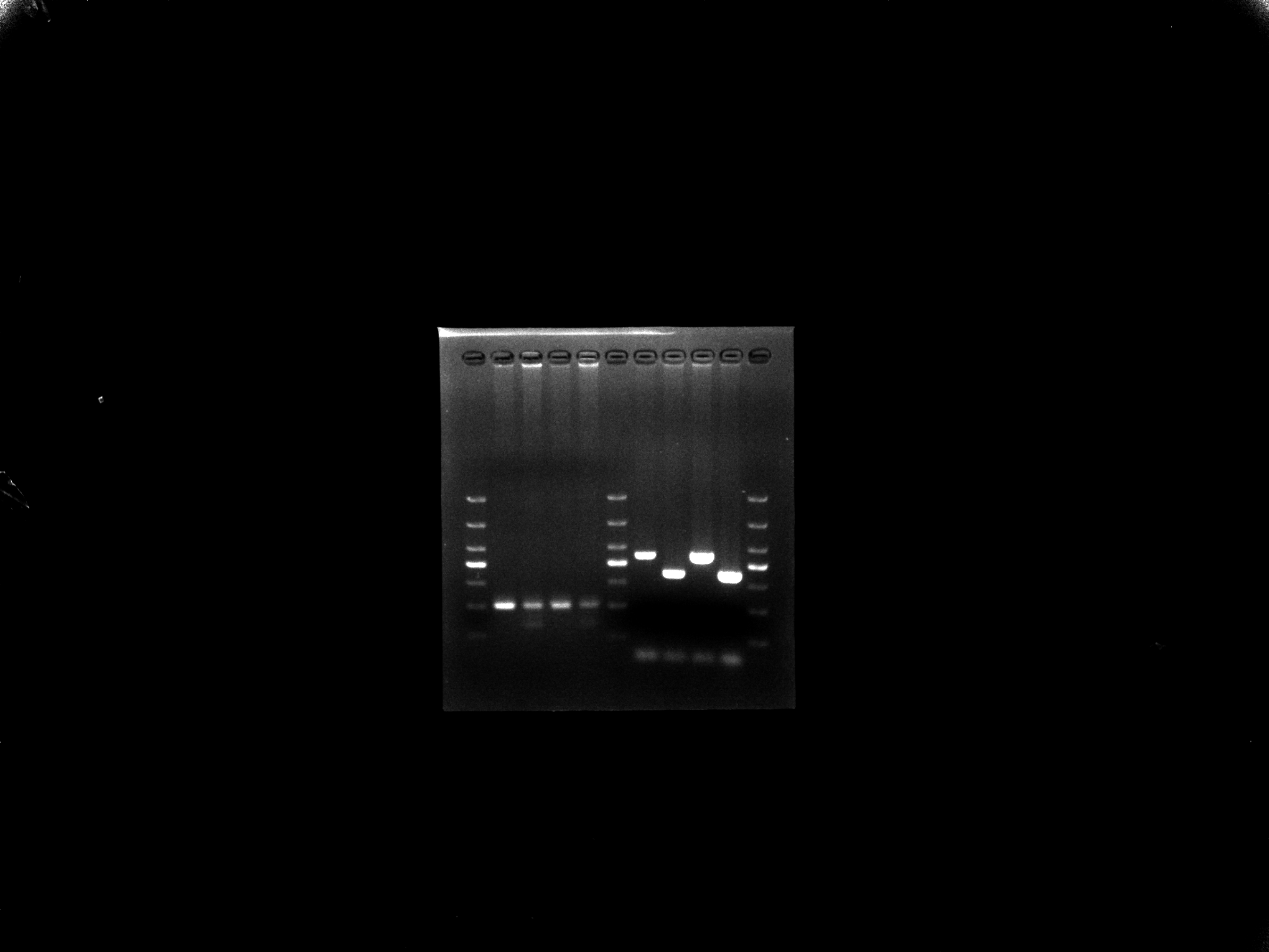

Supplement: Figure 1—figure supplement 1—source data 1. [file elife-83129-fig1-figsupp1-data1.zip › Figure supplement S1-source data 8/Raw gels of Figure supplement S1.tif]

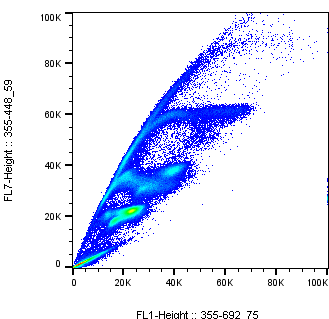

Supplement: Figure 1—figure supplement 2—source data 1. [file elife-83129-fig1-figsupp2-data1.zip › Figure supplement S2-source data 9/FACS-KO.tif]

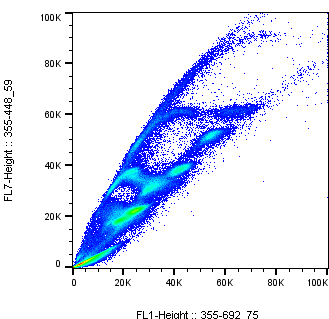

Supplement: Figure 1—figure supplement 2—source data 1. [file elife-83129-fig1-figsupp2-data1.zip › Figure supplement S2-source data 9/FACS-WT.tif]

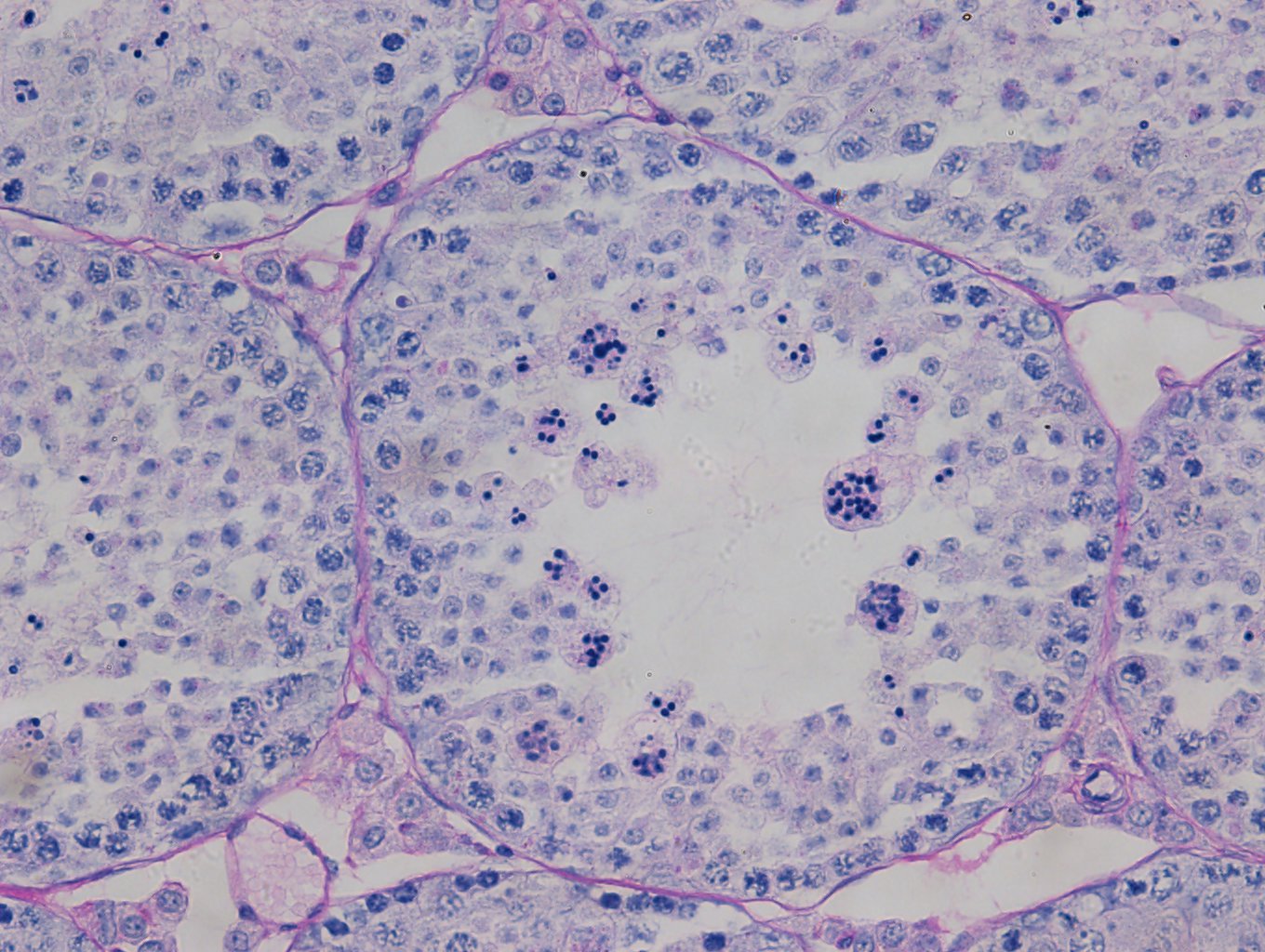

Supplement: Figure 2—source data 1. [file elife-83129-fig2-data1.zip › Figure2/Source data of Figure2A/Source data of KO-PAS staining/═╝╧±_39719.jpg]

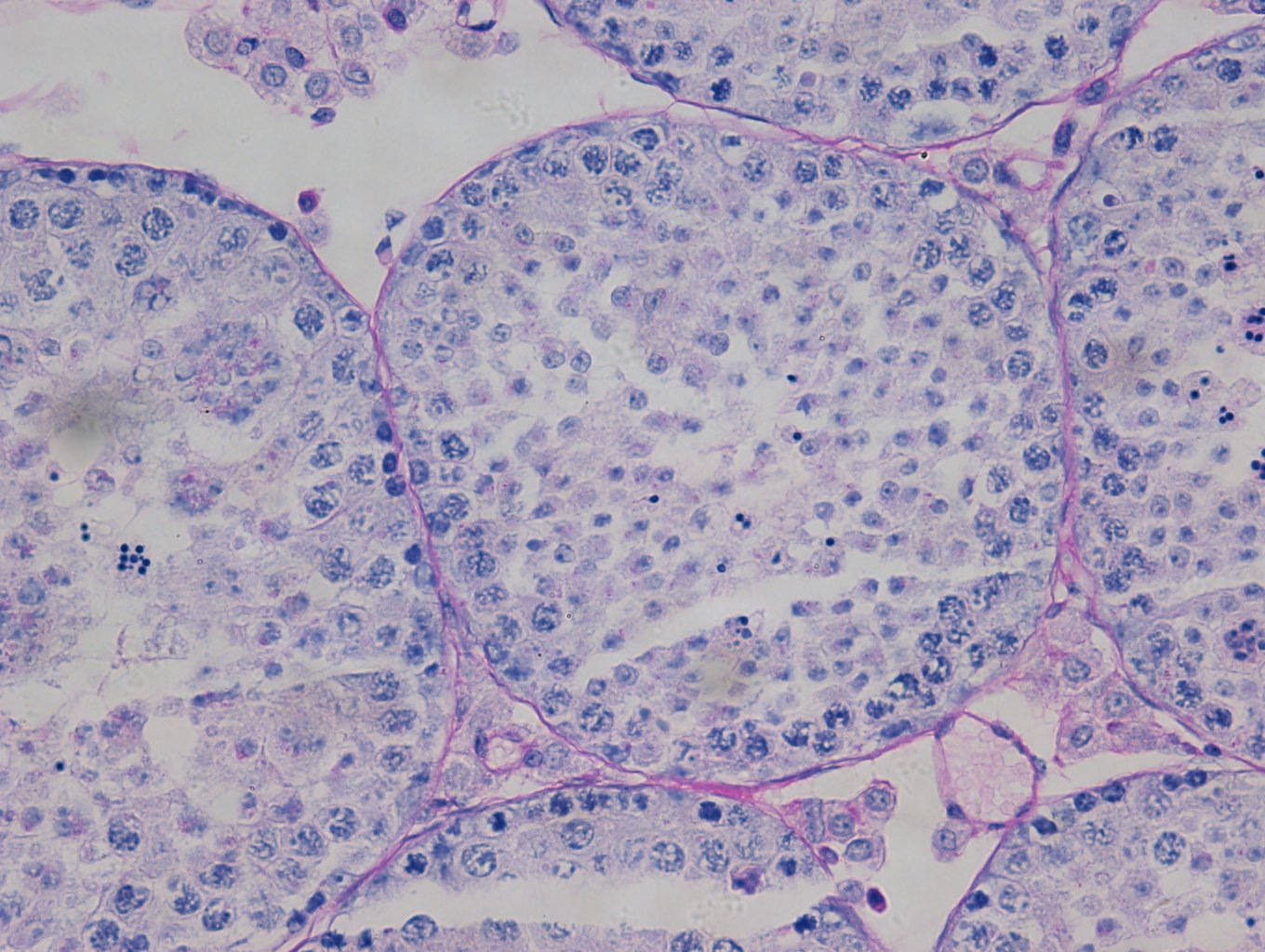

Supplement: Figure 2—source data 1. [file elife-83129-fig2-data1.zip › Figure2/Source data of Figure2A/Source data of KO-PAS staining/═╝╧±_39720.jpg]

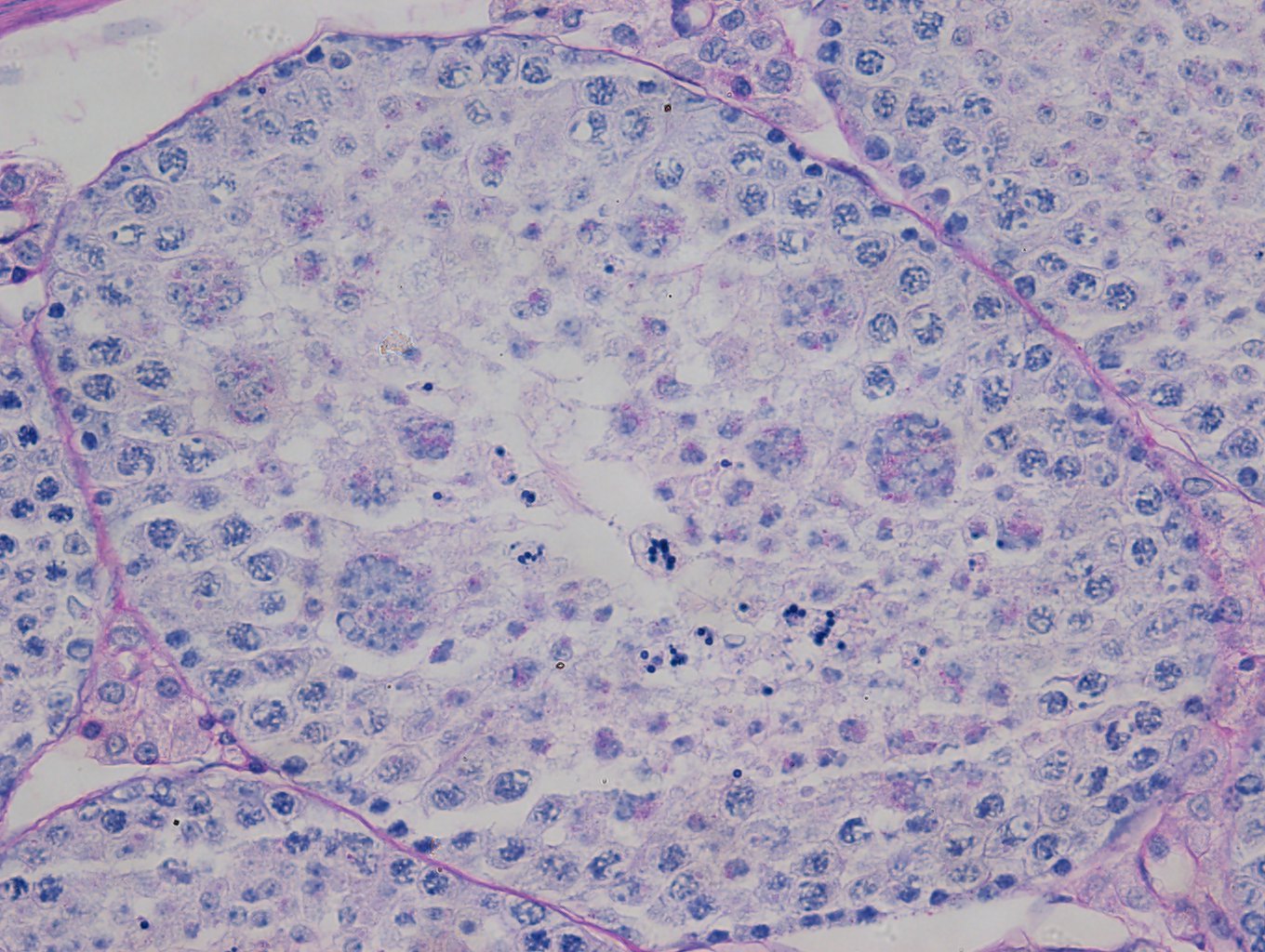

Supplement: Figure 2—source data 1. [file elife-83129-fig2-data1.zip › Figure2/Source data of Figure2A/Source data of KO-PAS staining/═╝╧±_39721.jpg]

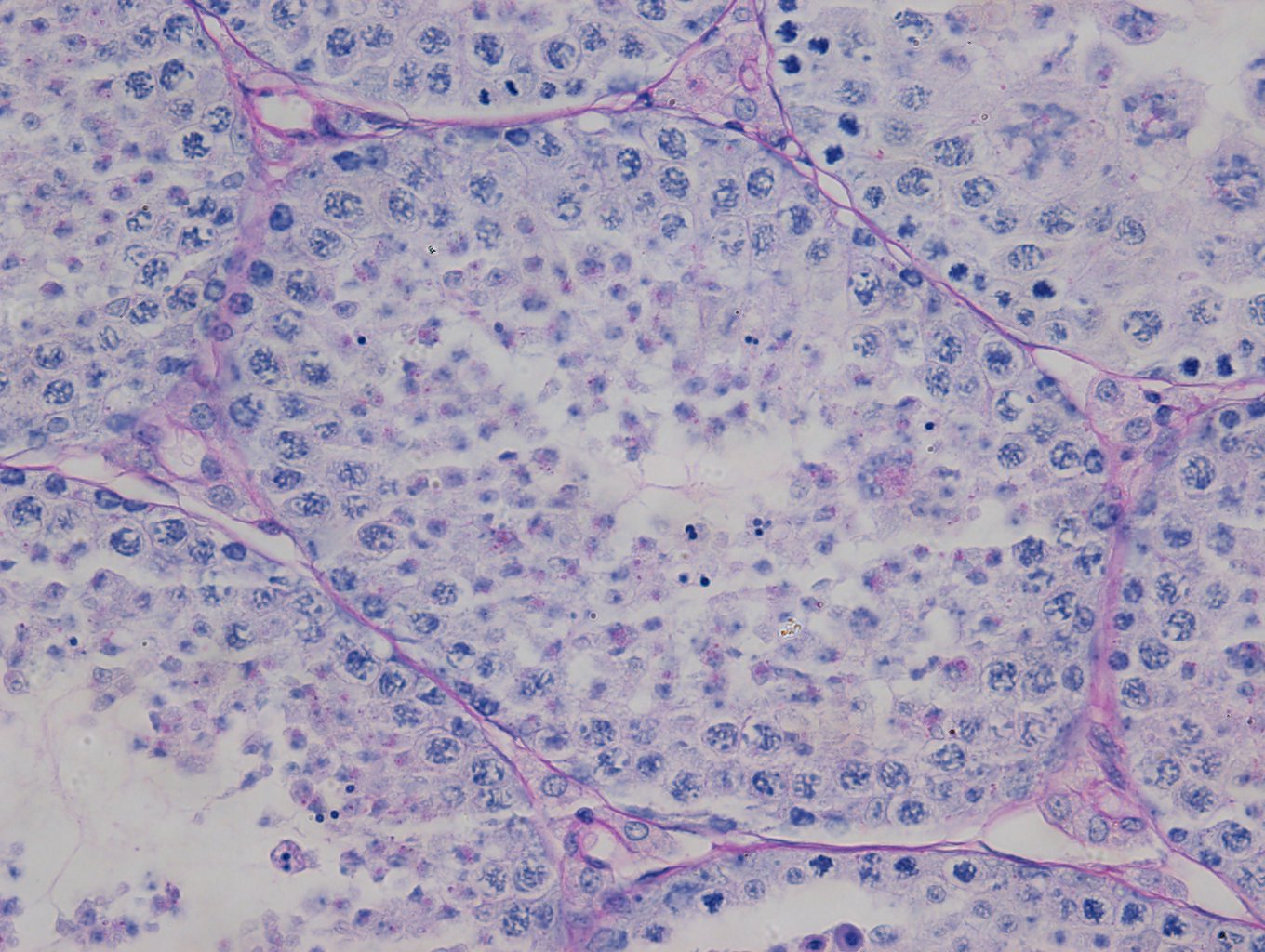

Supplement: Figure 2—source data 1. [file elife-83129-fig2-data1.zip › Figure2/Source data of Figure2A/Source data of KO-PAS staining/═╝╧±_39722.jpg]

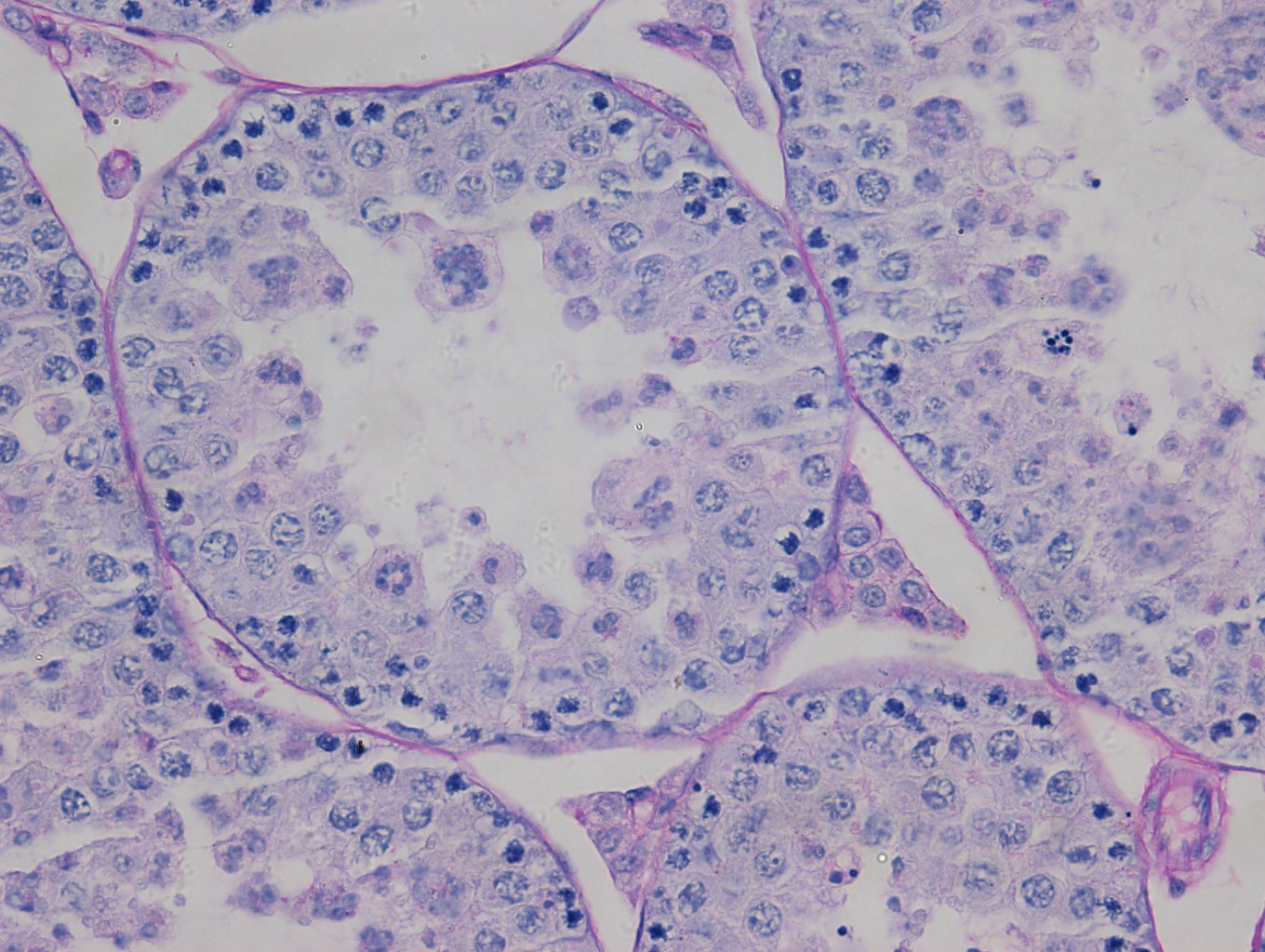

Supplement: Figure 2—source data 1. [file elife-83129-fig2-data1.zip › Figure2/Source data of Figure2A/Source data of KO-PAS staining/═╝╧±_39723.jpg]

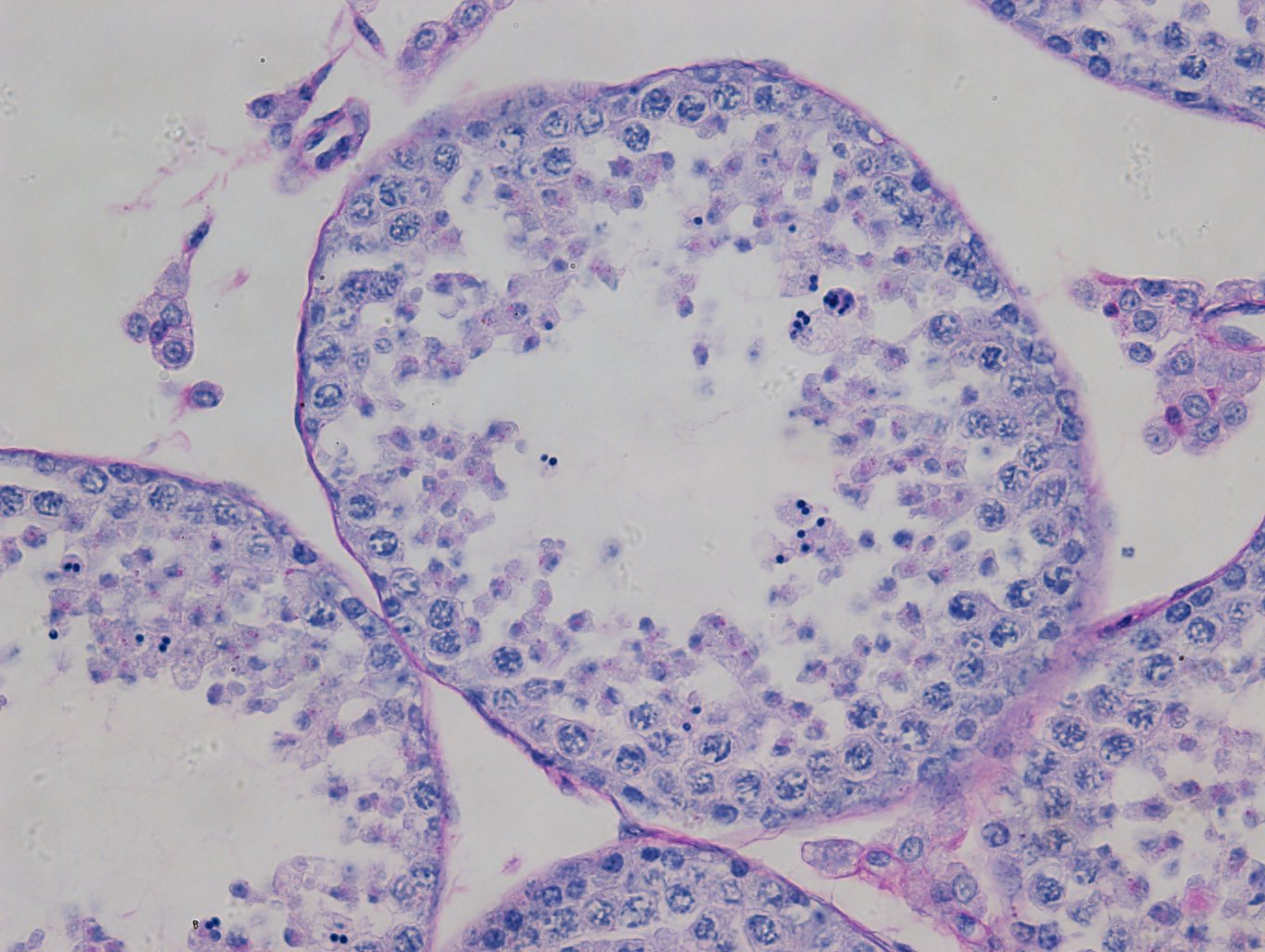

Supplement: Figure 2—source data 1. [file elife-83129-fig2-data1.zip › Figure2/Source data of Figure2A/Source data of KO-PAS staining/═╝╧±_39724.jpg]

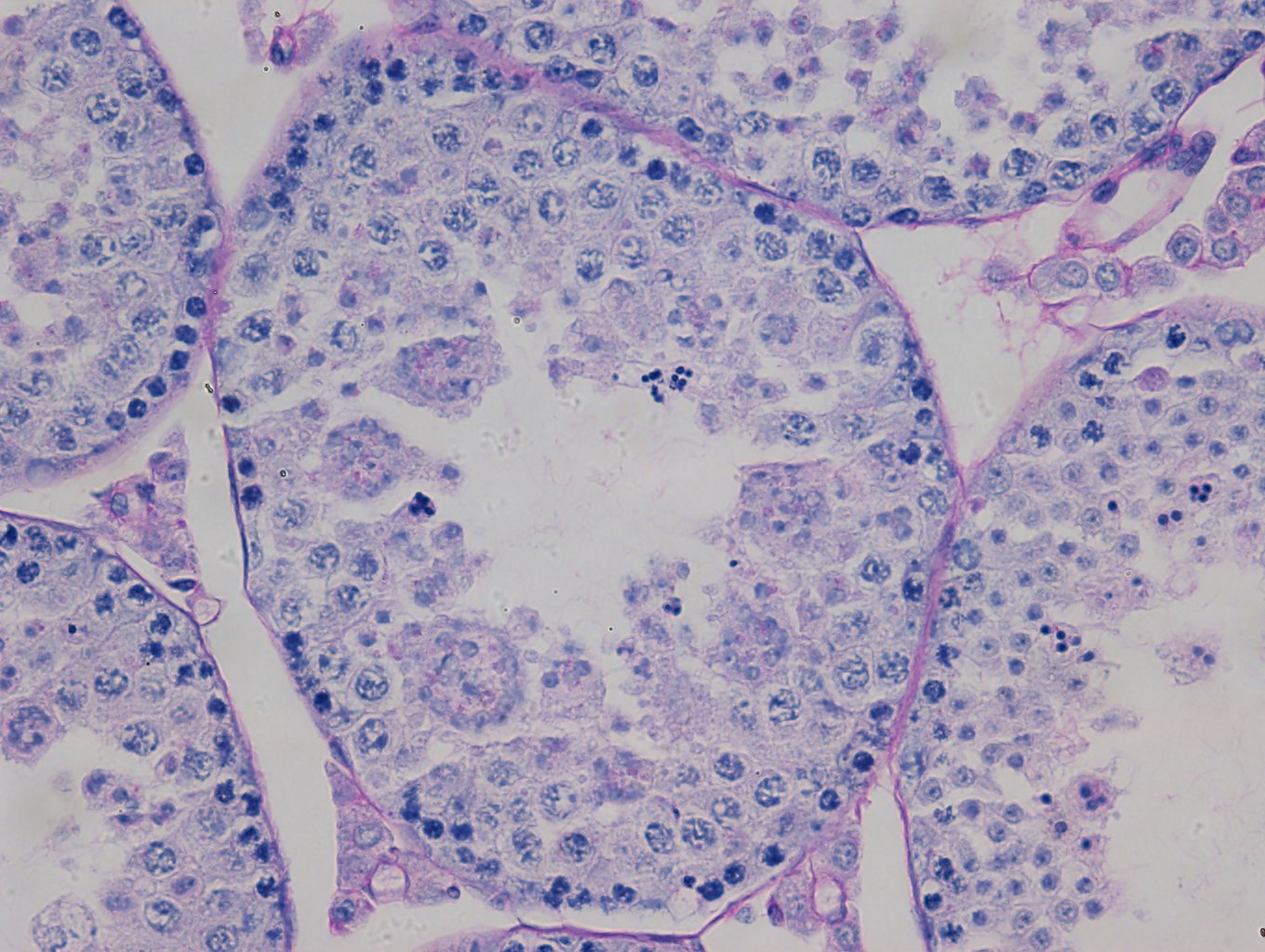

Supplement: Figure 2—source data 1. [file elife-83129-fig2-data1.zip › Figure2/Source data of Figure2A/Source data of KO-PAS staining/═╝╧±_39725.jpg]

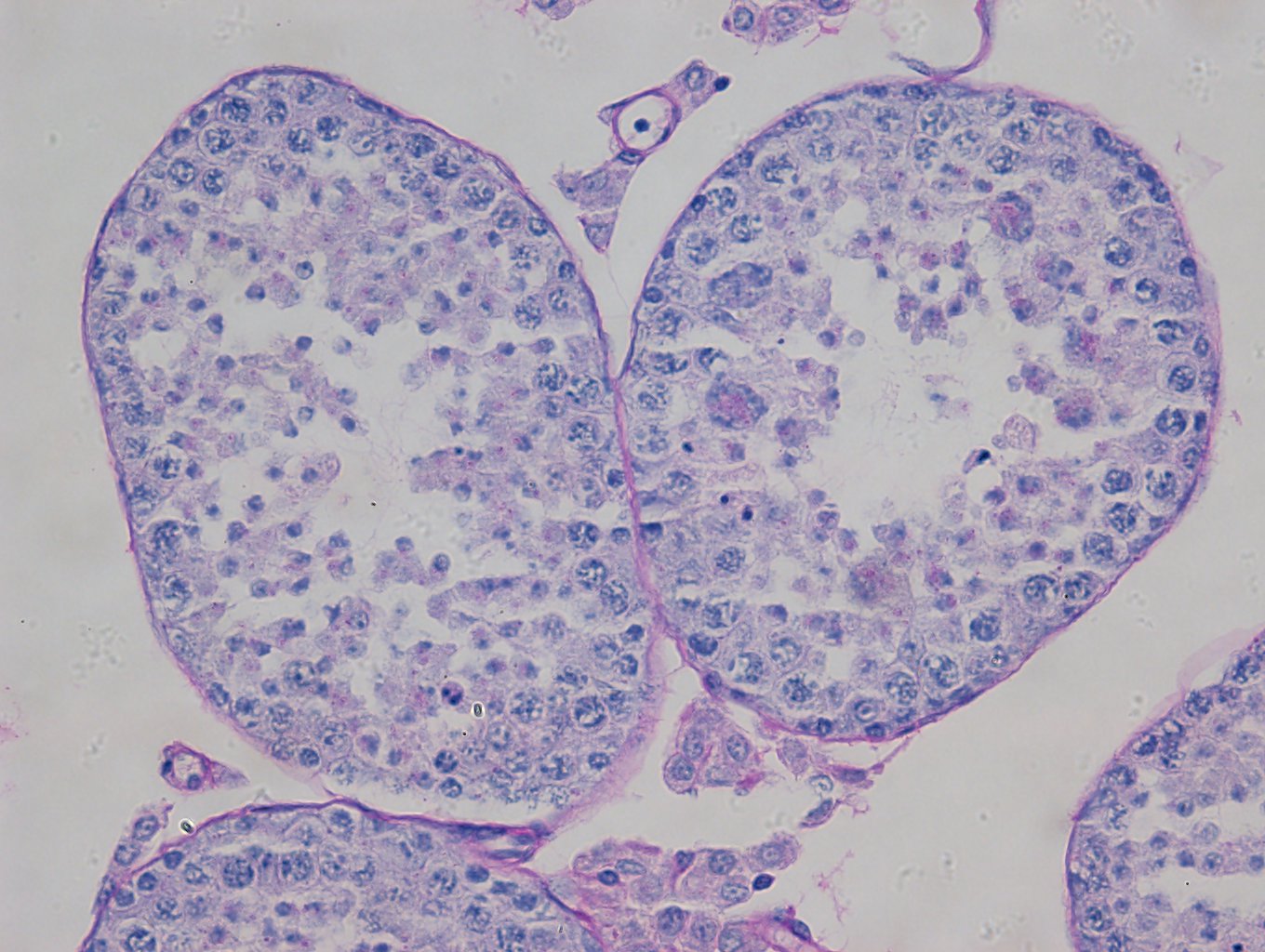

Supplement: Figure 2—source data 1. [file elife-83129-fig2-data1.zip › Figure2/Source data of Figure2A/Source data of KO-PAS staining/═╝╧±_39726.jpg]

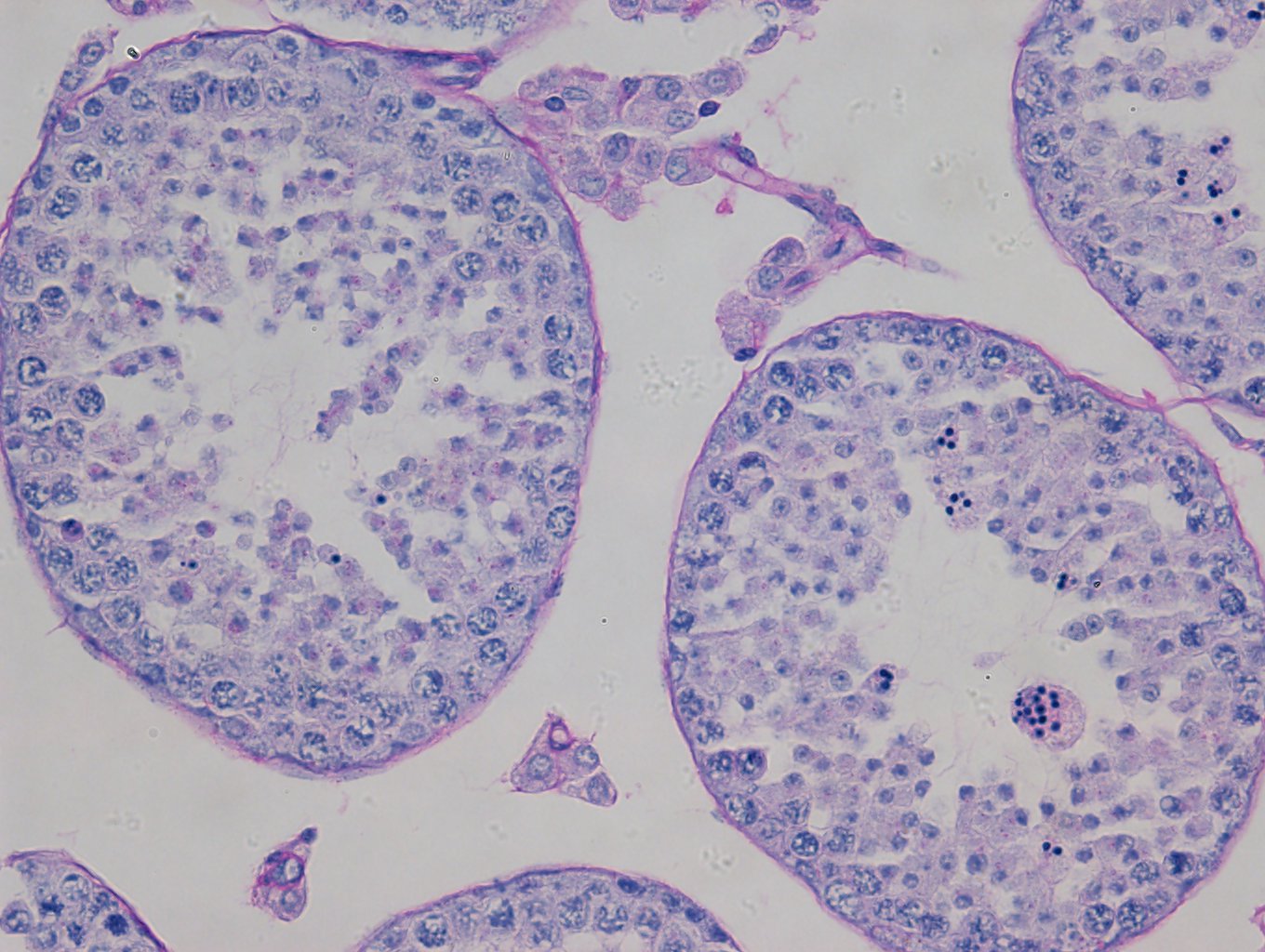

Supplement: Figure 2—source data 1. [file elife-83129-fig2-data1.zip › Figure2/Source data of Figure2A/Source data of KO-PAS staining/═╝╧±_39727.jpg]

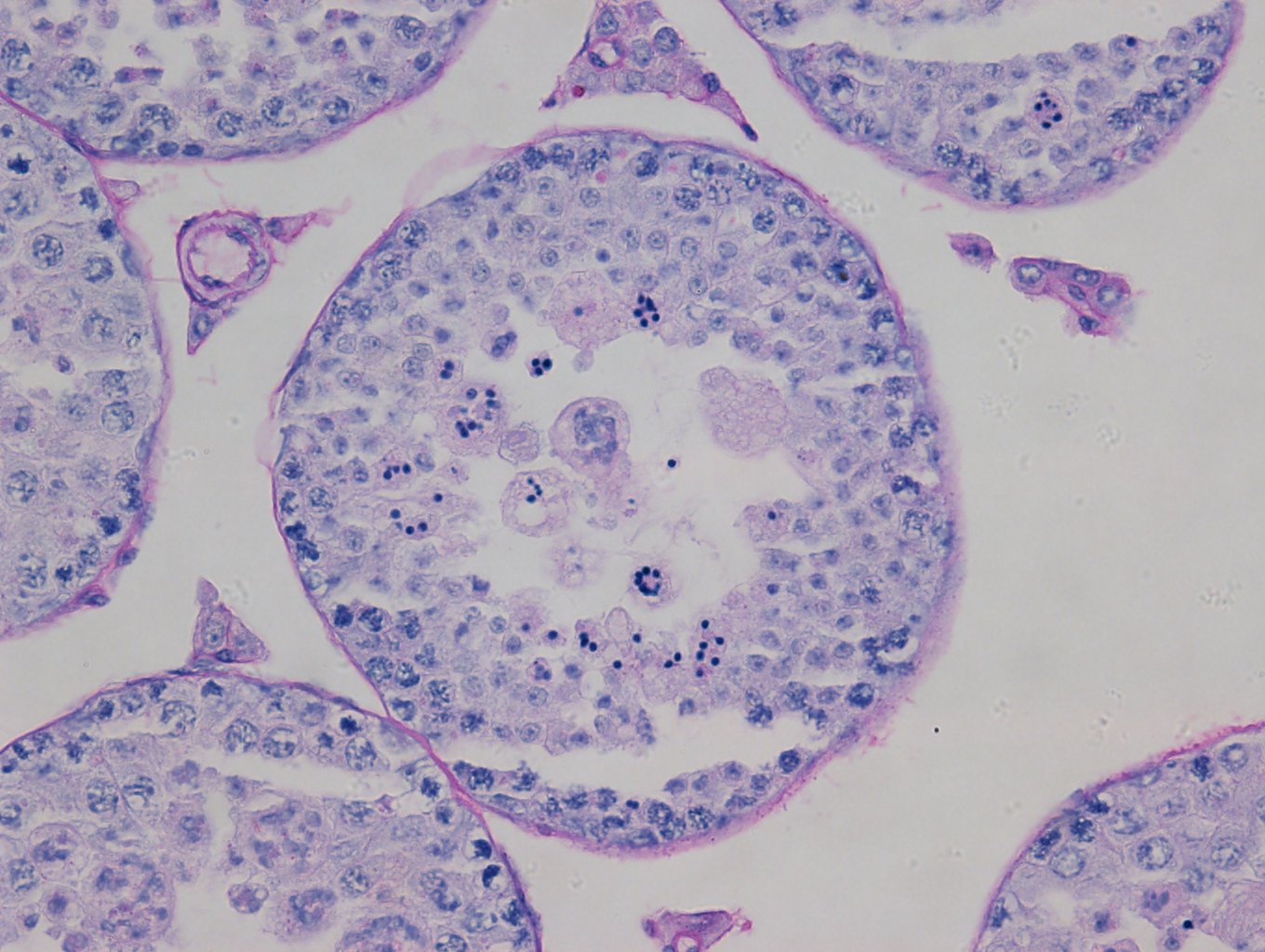

Supplement: Figure 2—source data 1. [file elife-83129-fig2-data1.zip › Figure2/Source data of Figure2A/Source data of KO-PAS staining/═╝╧±_39728.jpg]

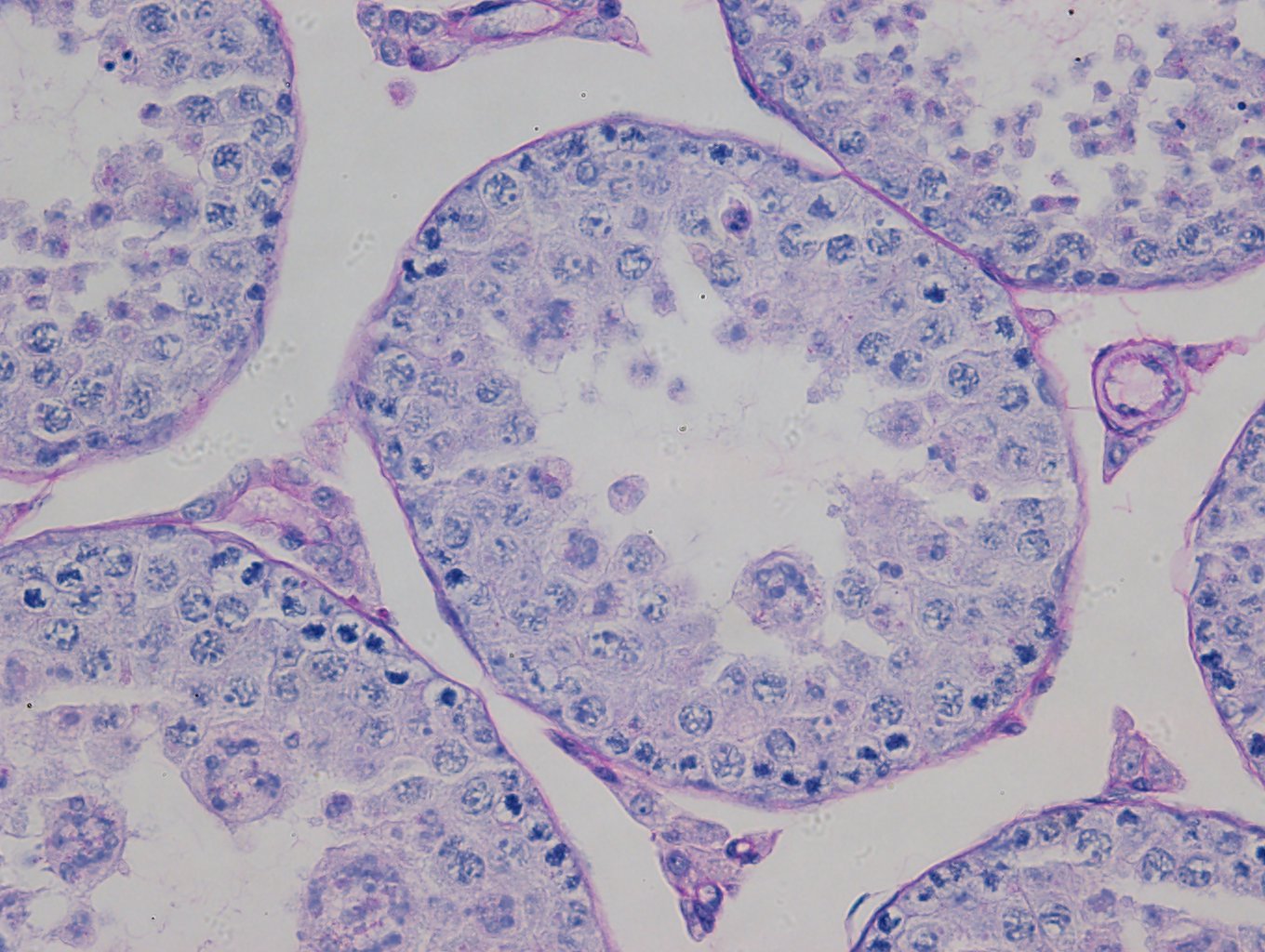

Supplement: Figure 2—source data 1. [file elife-83129-fig2-data1.zip › Figure2/Source data of Figure2A/Source data of KO-PAS staining/═╝╧±_39729.jpg]

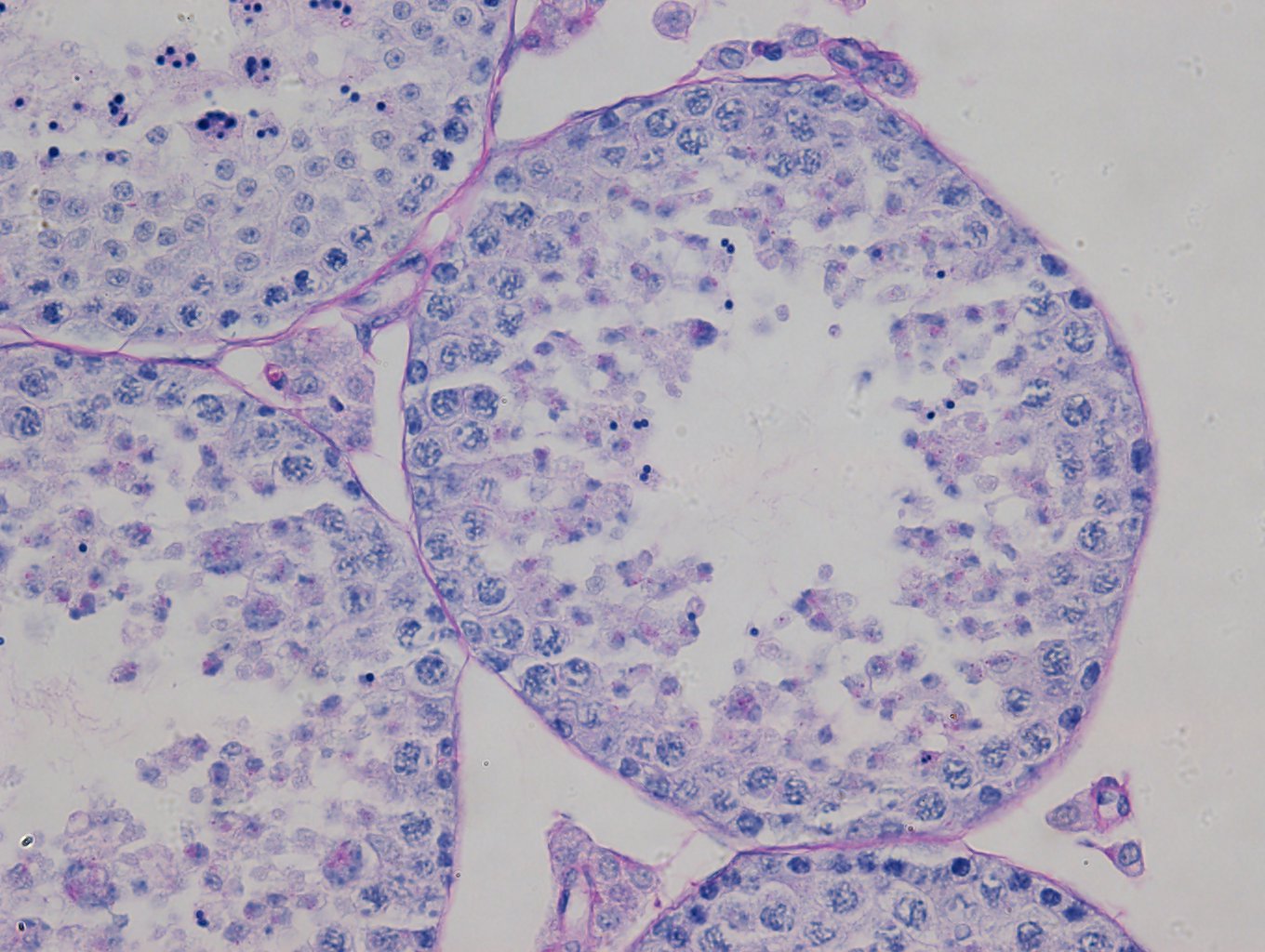

Supplement: Figure 2—source data 1. [file elife-83129-fig2-data1.zip › Figure2/Source data of Figure2A/Source data of KO-PAS staining/═╝╧±_39730.jpg]

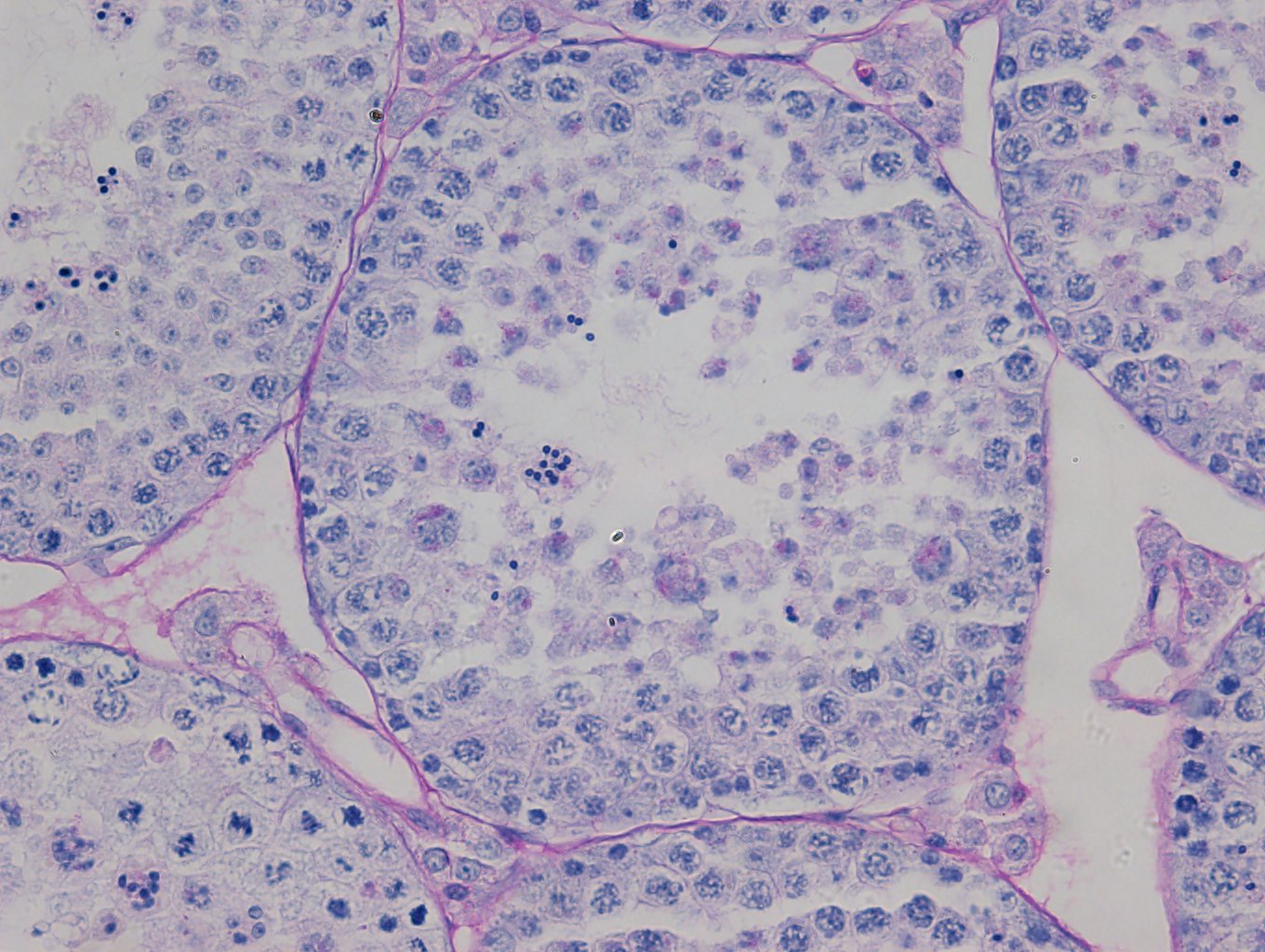

Supplement: Figure 2—source data 1. [file elife-83129-fig2-data1.zip › Figure2/Source data of Figure2A/Source data of KO-PAS staining/═╝╧±_39731.jpg]

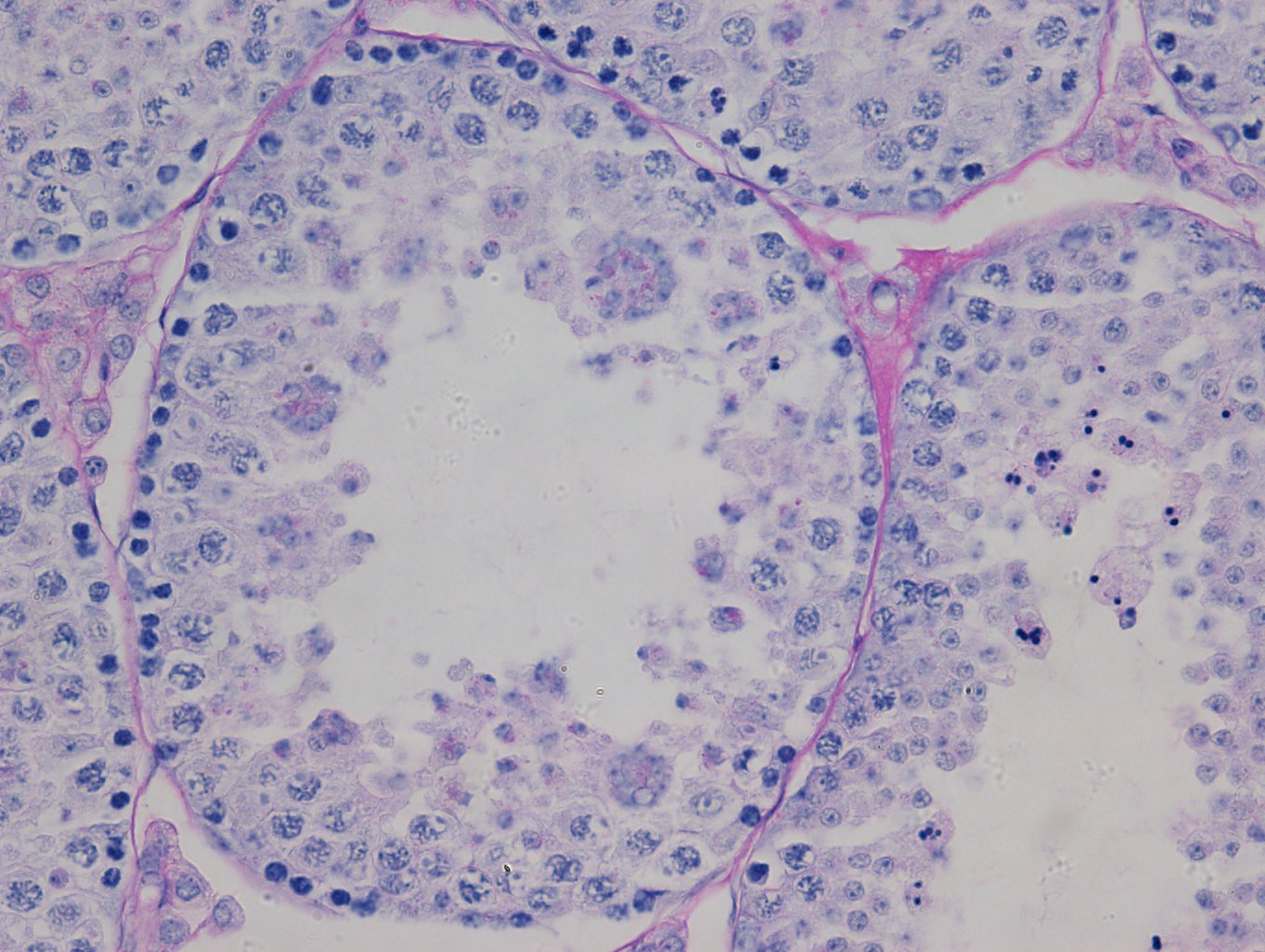

Supplement: Figure 2—source data 1. [file elife-83129-fig2-data1.zip › Figure2/Source data of Figure2A/Source data of KO-PAS staining/═╝╧±_39732.jpg]

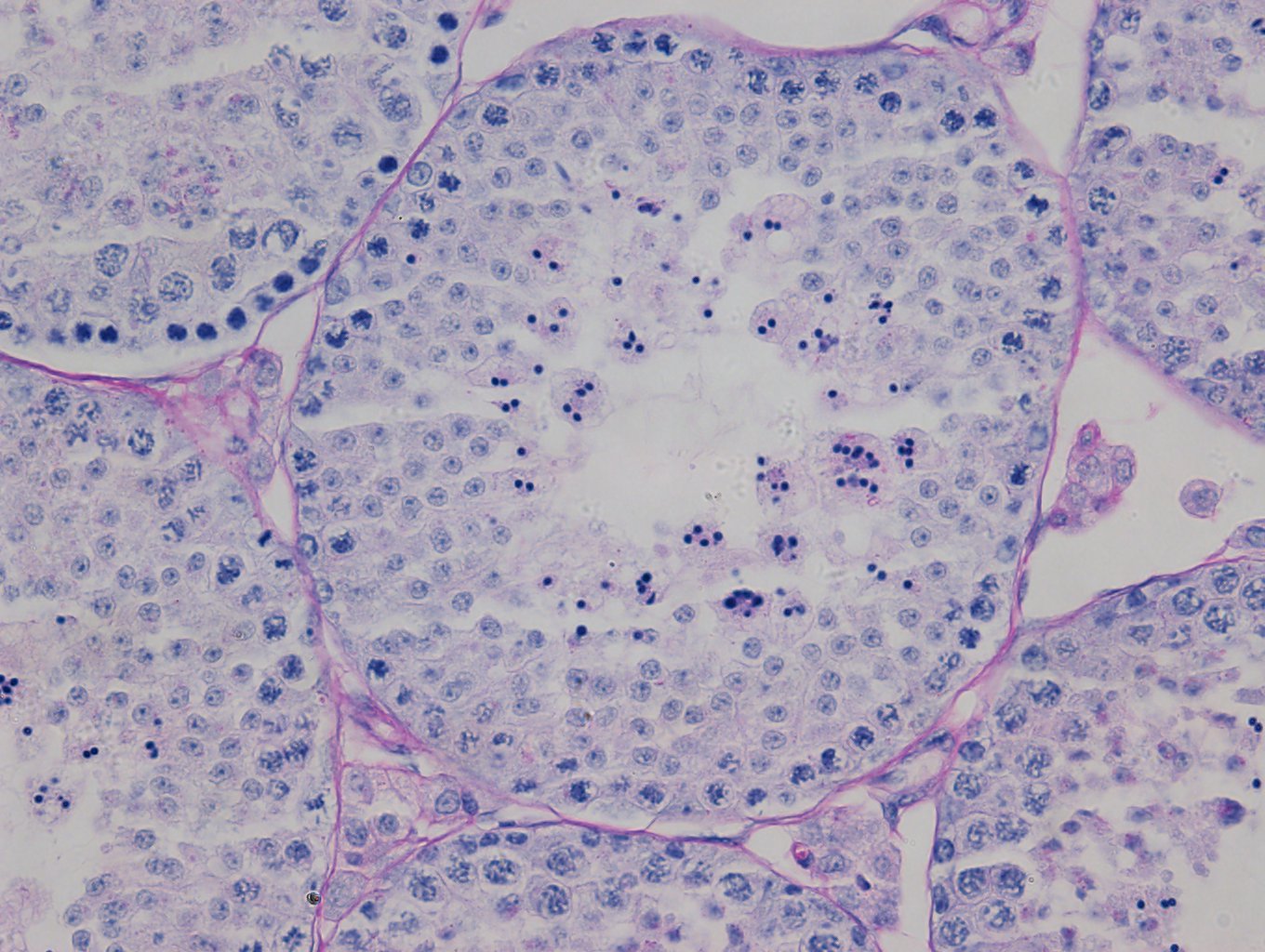

Supplement: Figure 2—source data 1. [file elife-83129-fig2-data1.zip › Figure2/Source data of Figure2A/Source data of KO-PAS staining/═╝╧±_39733.jpg]

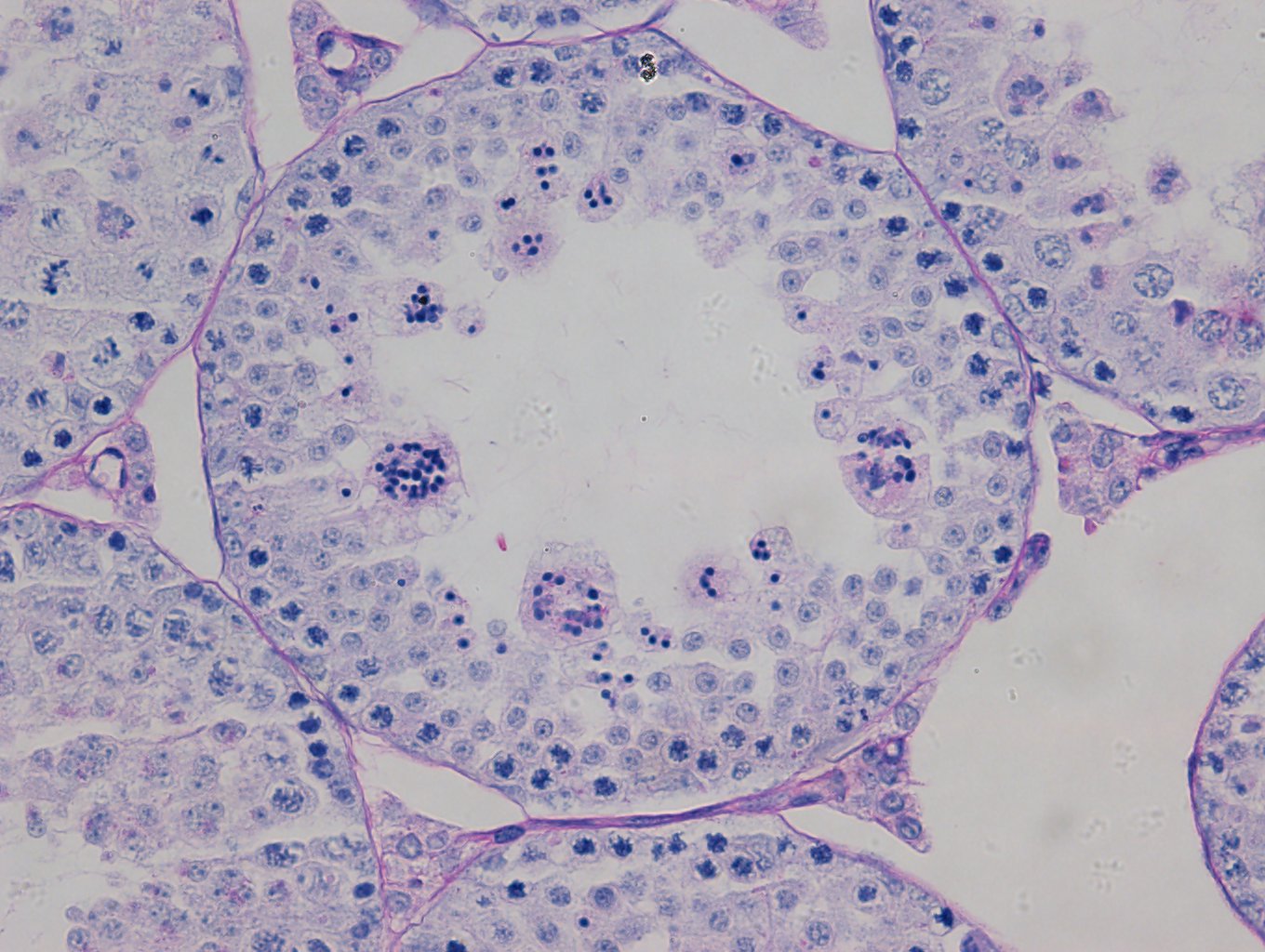

Supplement: Figure 2—source data 1. [file elife-83129-fig2-data1.zip › Figure2/Source data of Figure2A/Source data of KO-PAS staining/═╝╧±_39734.jpg]

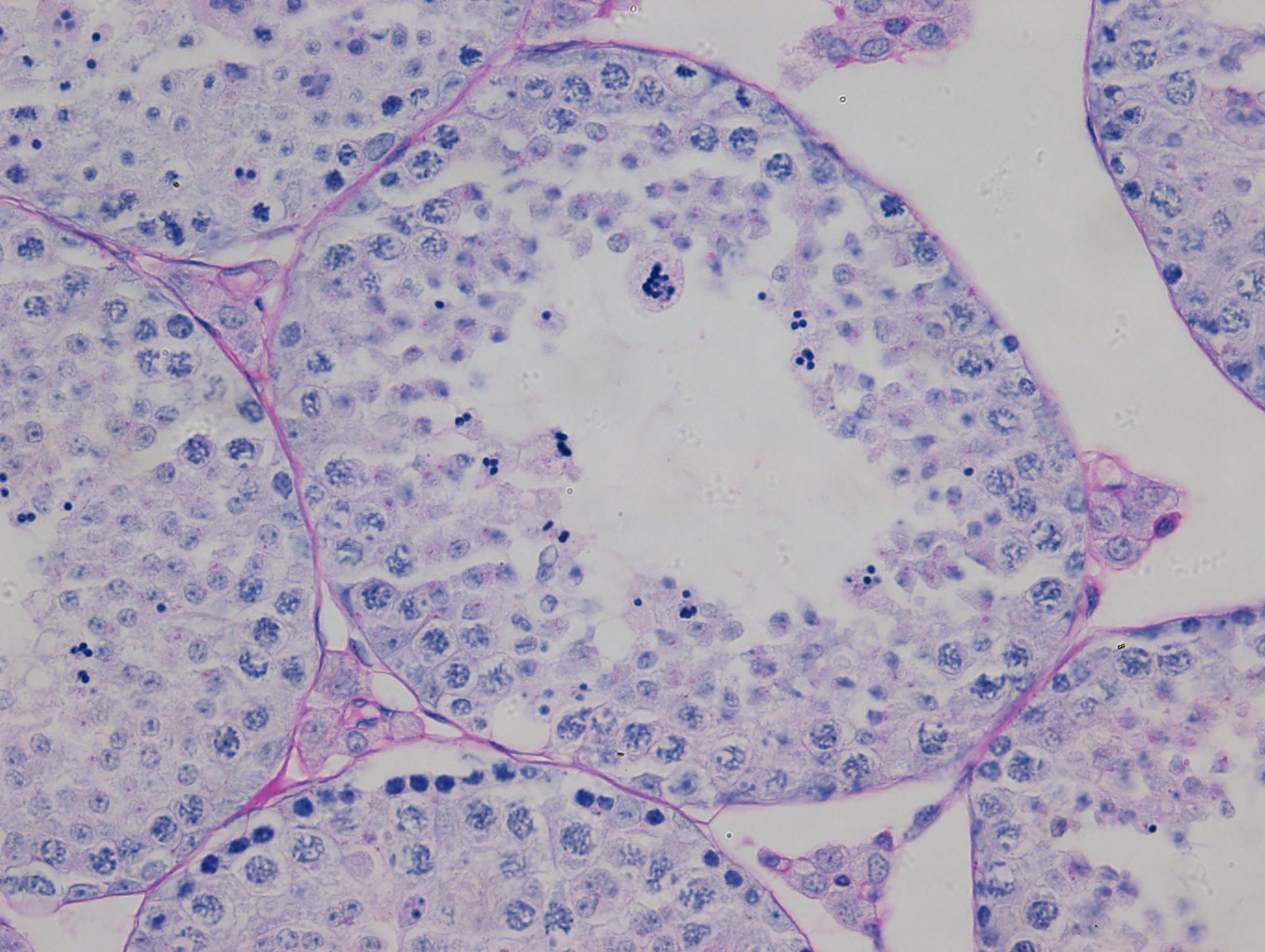

Supplement: Figure 2—source data 1. [file elife-83129-fig2-data1.zip › Figure2/Source data of Figure2A/Source data of KO-PAS staining/═╝╧±_39735.jpg]

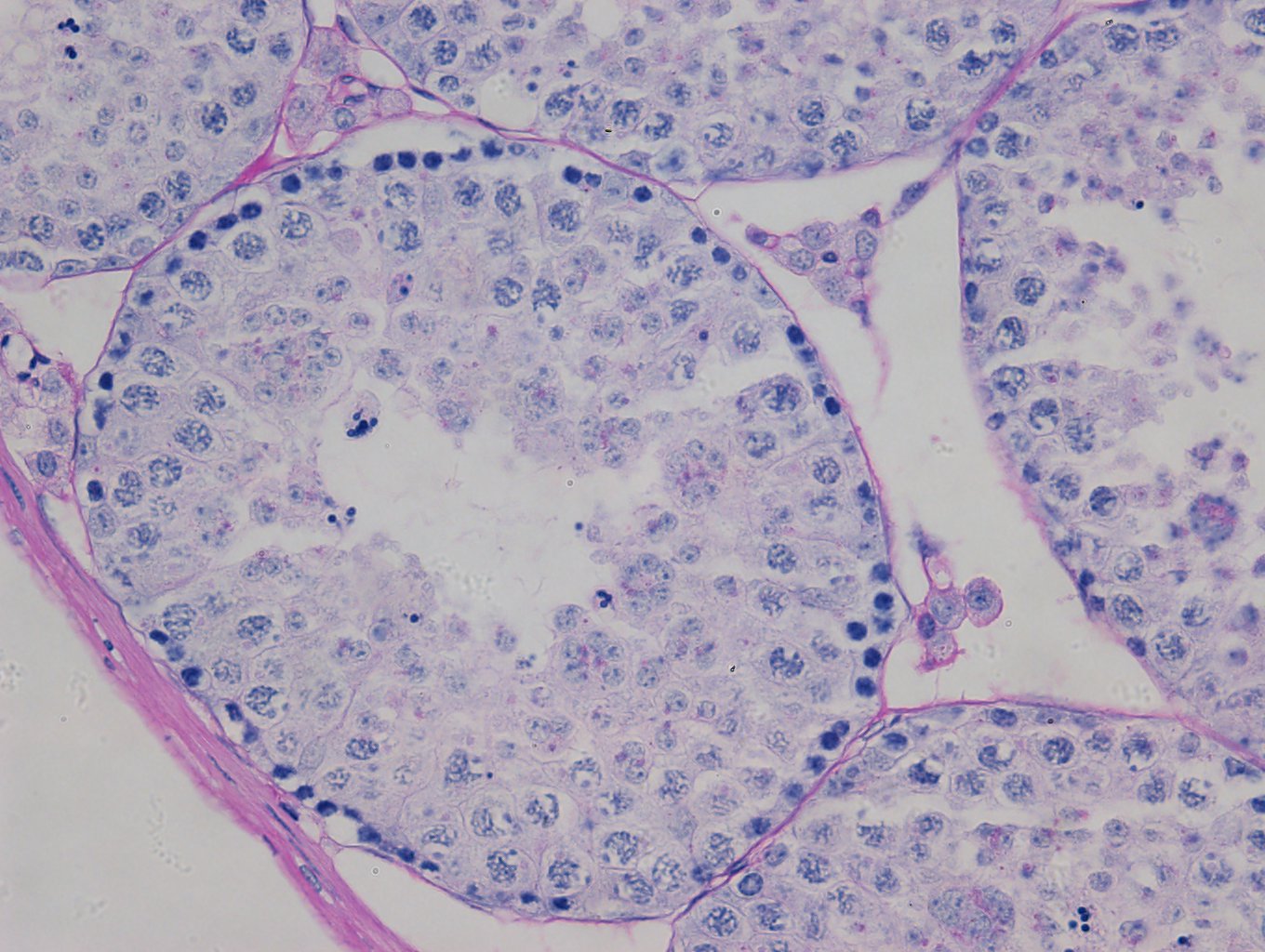

Supplement: Figure 2—source data 1. [file elife-83129-fig2-data1.zip › Figure2/Source data of Figure2A/Source data of KO-PAS staining/═╝╧±_39736.jpg]

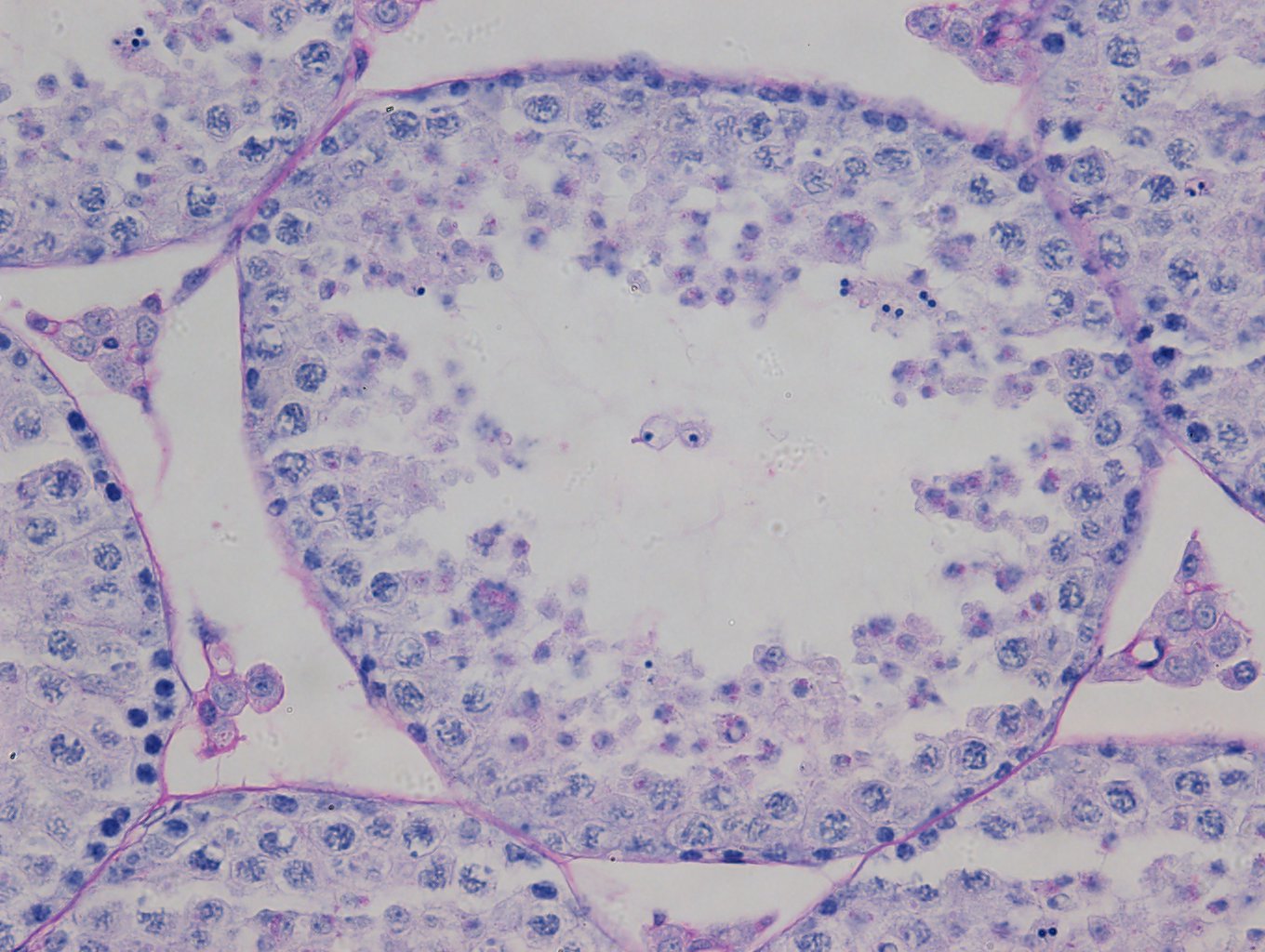

Supplement: Figure 2—source data 1. [file elife-83129-fig2-data1.zip › Figure2/Source data of Figure2A/Source data of KO-PAS staining/═╝╧±_39737.jpg]

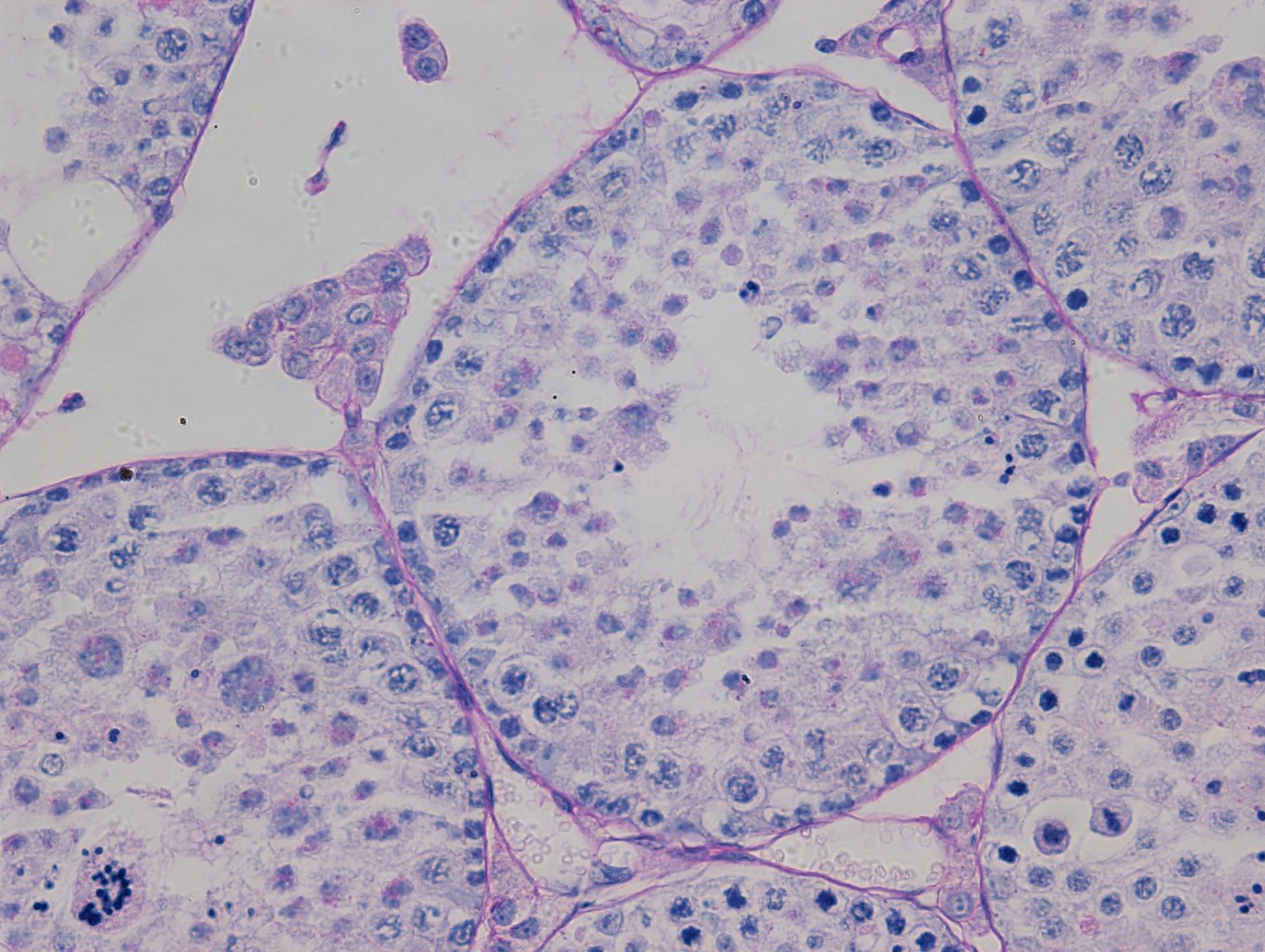

Supplement: Figure 2—source data 1. [file elife-83129-fig2-data1.zip › Figure2/Source data of Figure2A/Source data of KO-PAS staining/═╝╧±_39741.jpg]

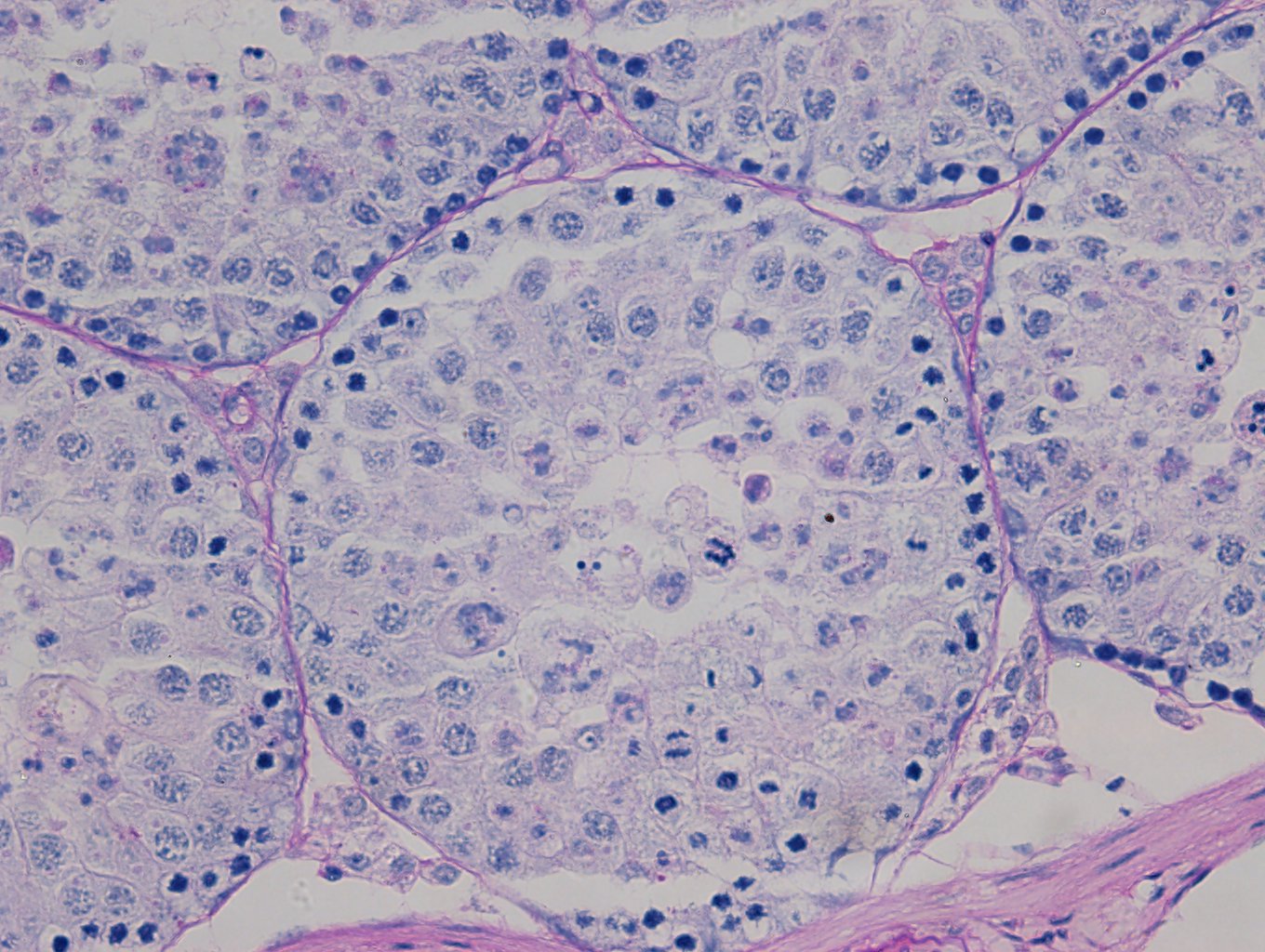

Supplement: Figure 2—source data 1. [file elife-83129-fig2-data1.zip › Figure2/Source data of Figure2A/Source data of KO-PAS staining/═╝╧±_39742.jpg]

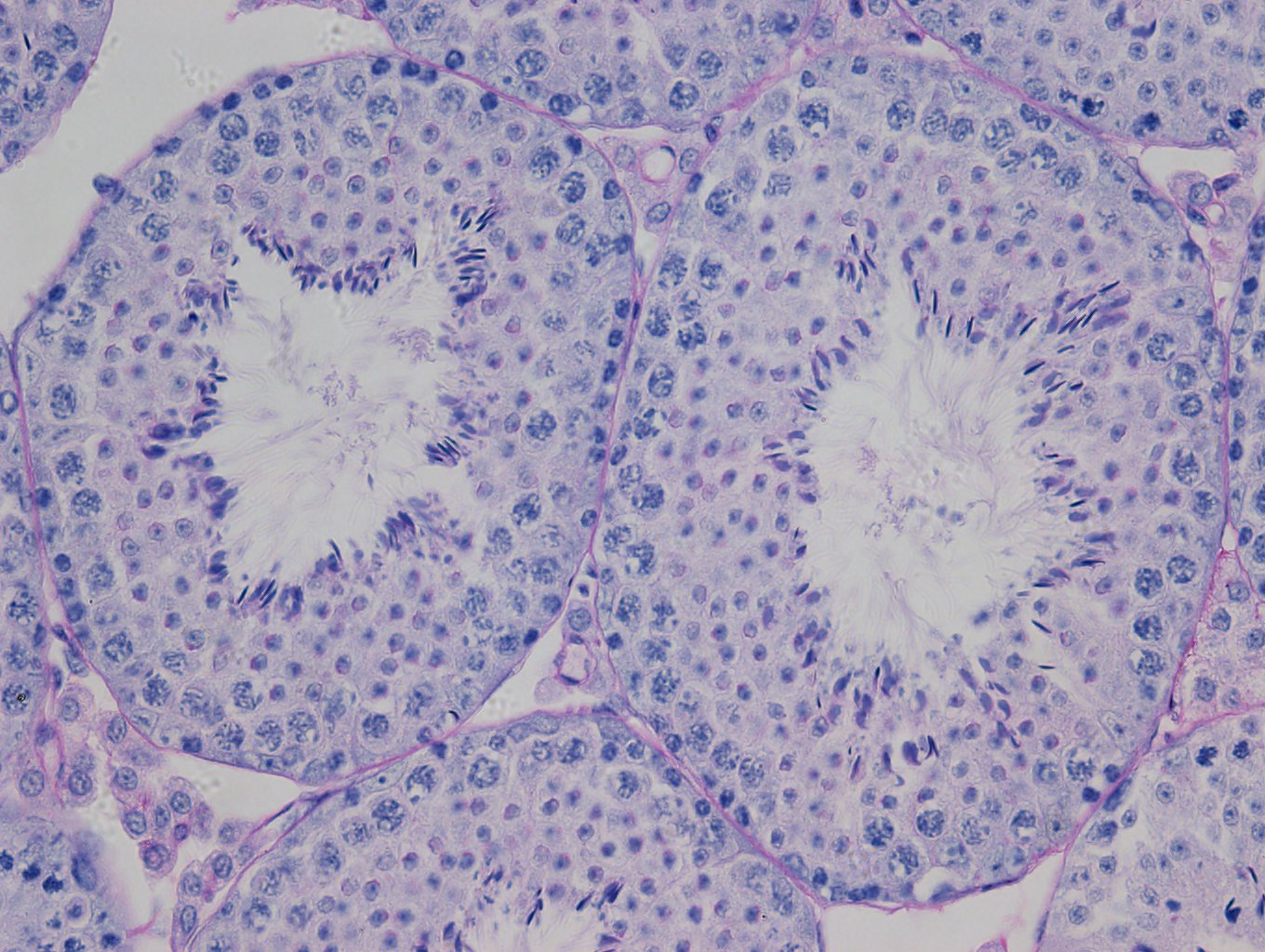

Supplement: Figure 2—source data 1. [file elife-83129-fig2-data1.zip › Figure2/Source data of Figure2A/Source data of WT-PAS staining/═╝╧±_39699.jpg]

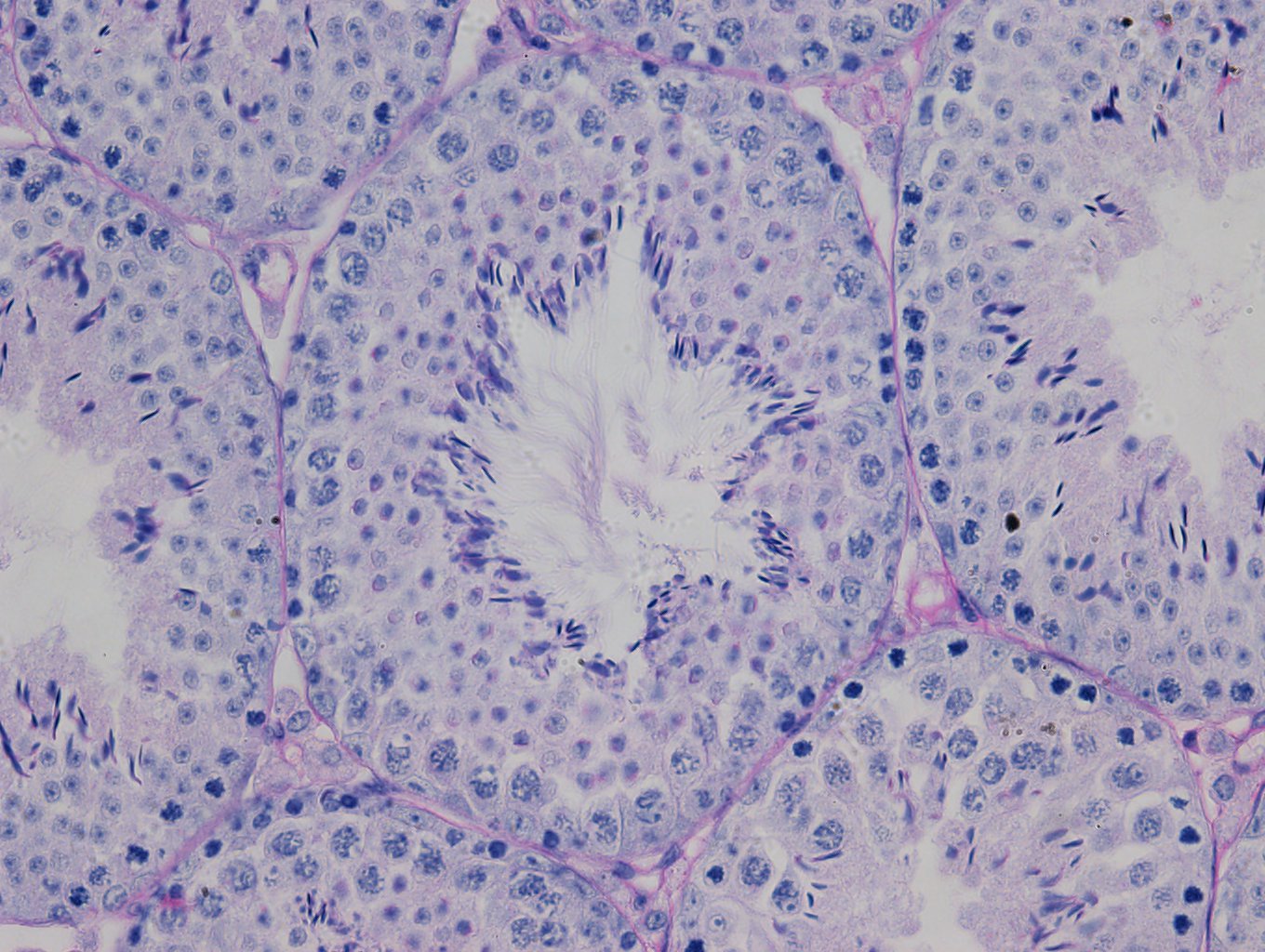

Supplement: Figure 2—source data 1. [file elife-83129-fig2-data1.zip › Figure2/Source data of Figure2A/Source data of WT-PAS staining/═╝╧±_39700.jpg]

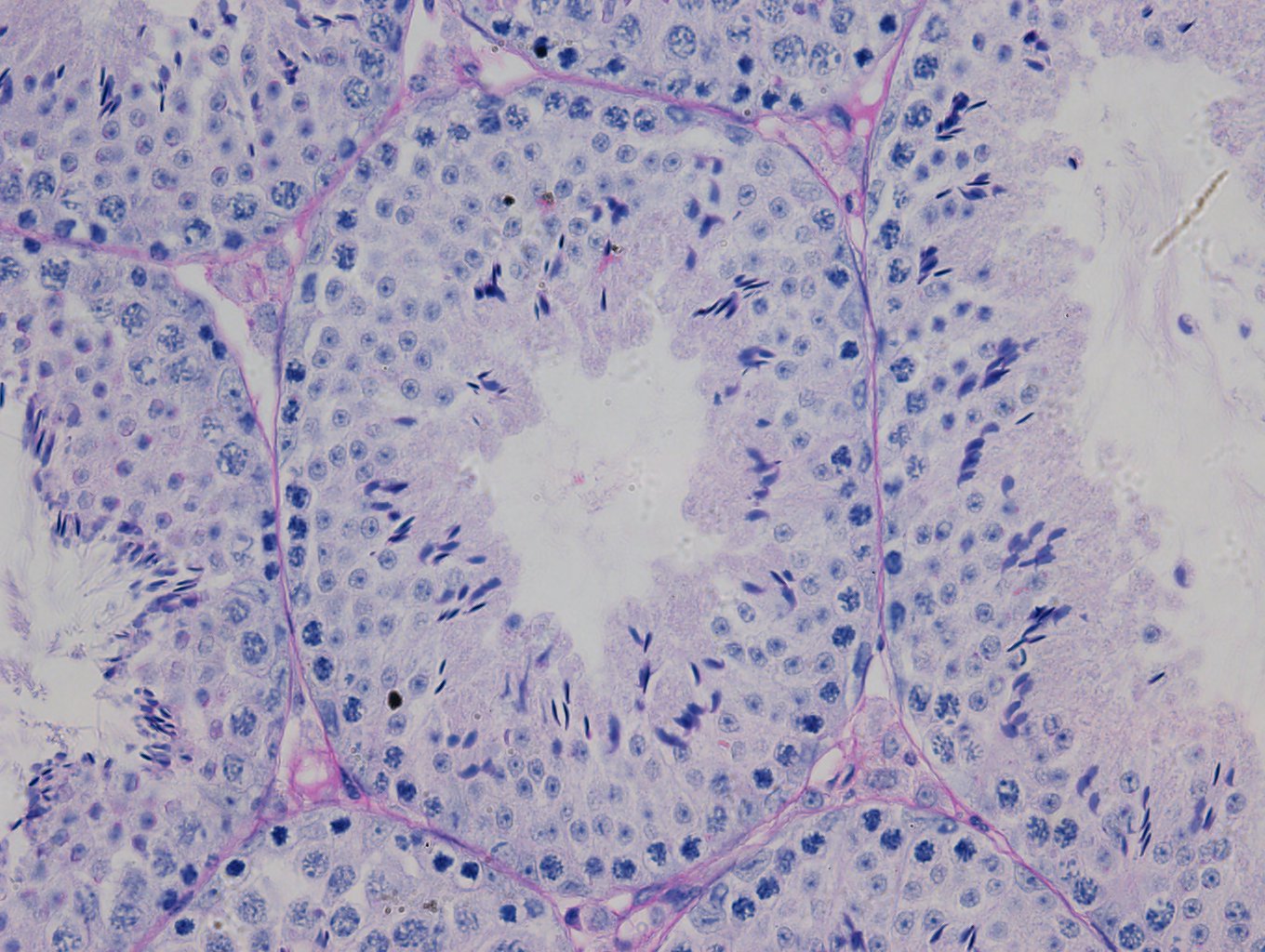

Supplement: Figure 2—source data 1. [file elife-83129-fig2-data1.zip › Figure2/Source data of Figure2A/Source data of WT-PAS staining/═╝╧±_39701.jpg]

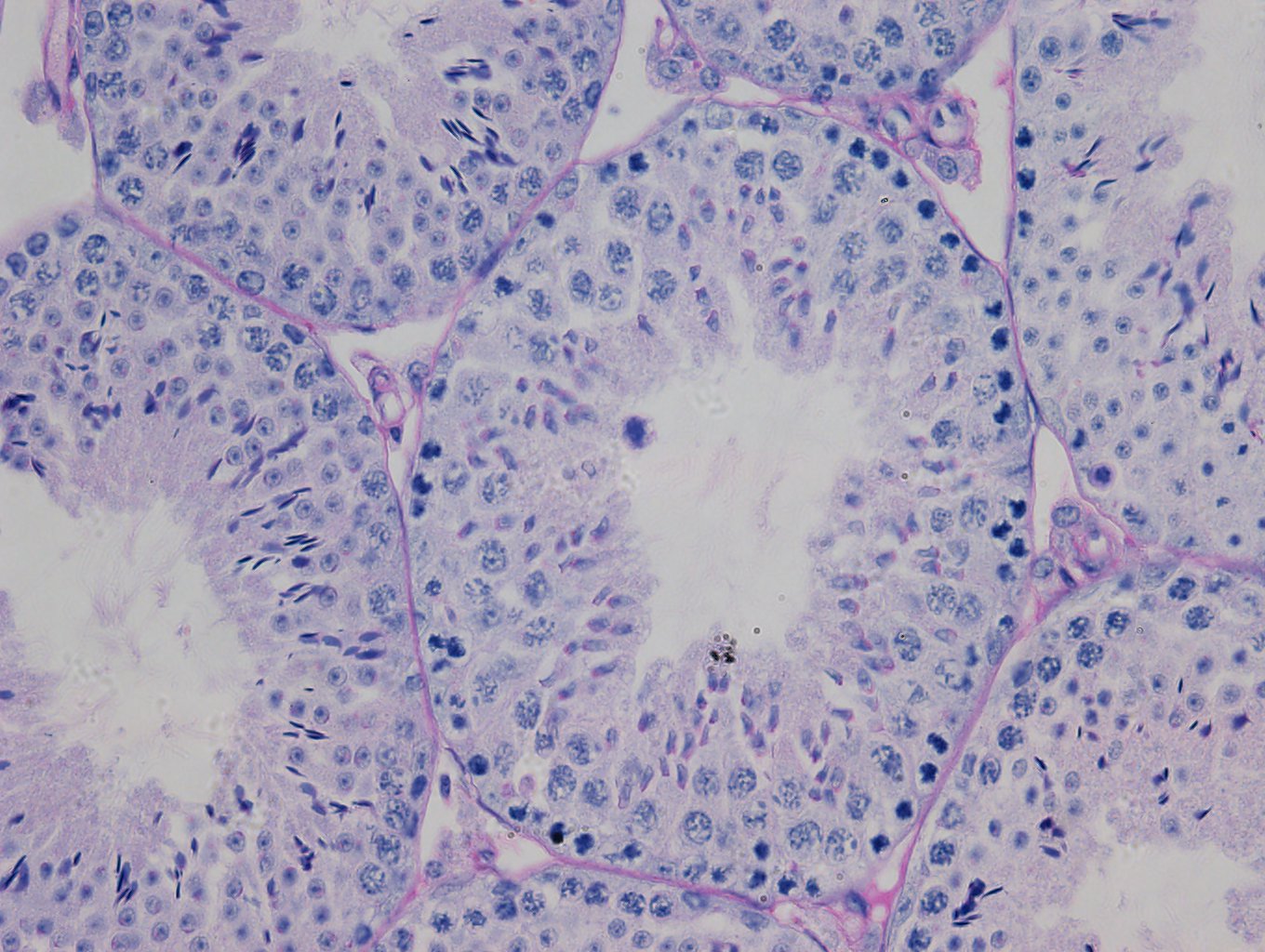

Supplement: Figure 2—source data 1. [file elife-83129-fig2-data1.zip › Figure2/Source data of Figure2A/Source data of WT-PAS staining/═╝╧±_39702.jpg]

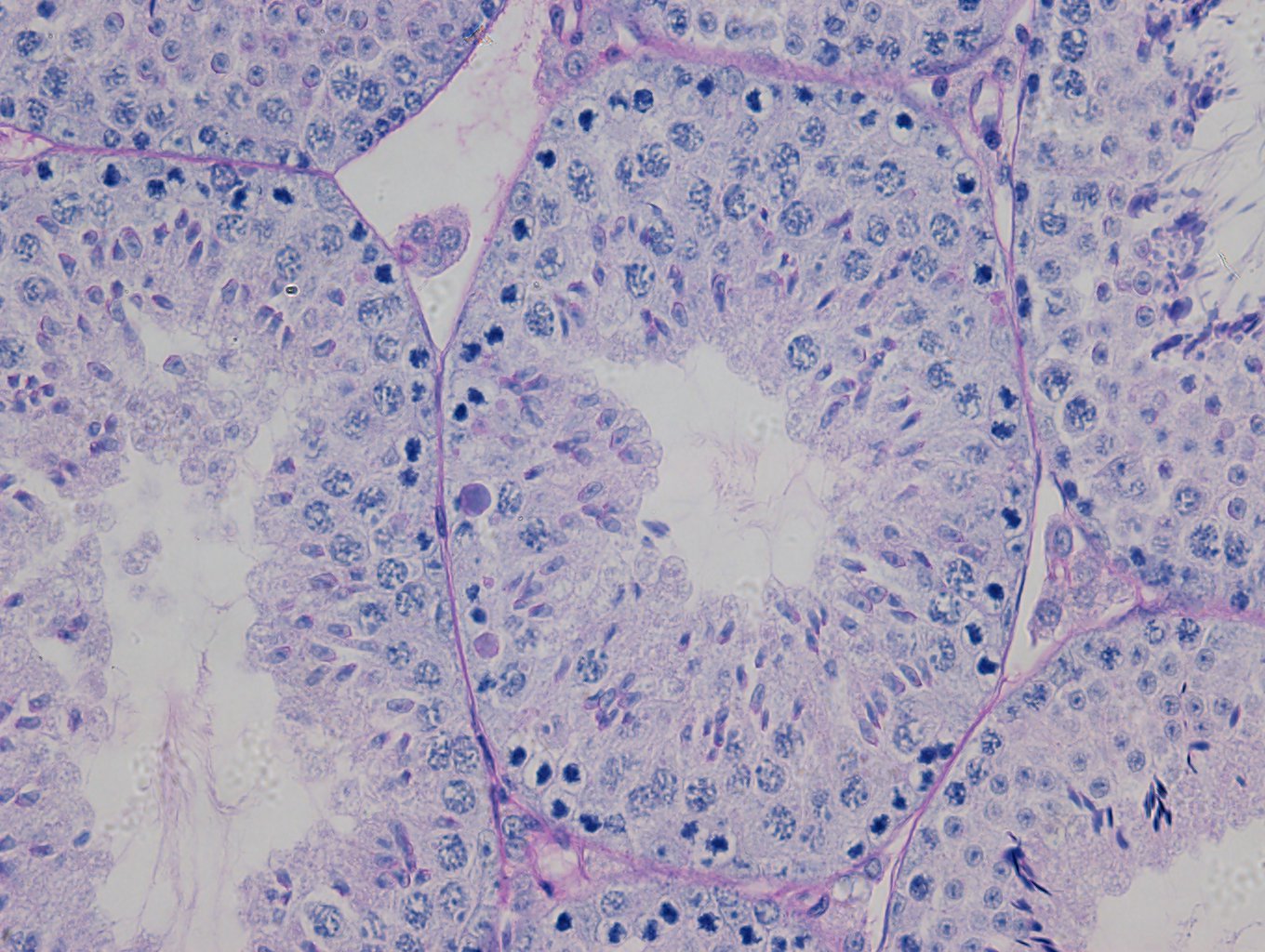

Supplement: Figure 2—source data 1. [file elife-83129-fig2-data1.zip › Figure2/Source data of Figure2A/Source data of WT-PAS staining/═╝╧±_39703.jpg]

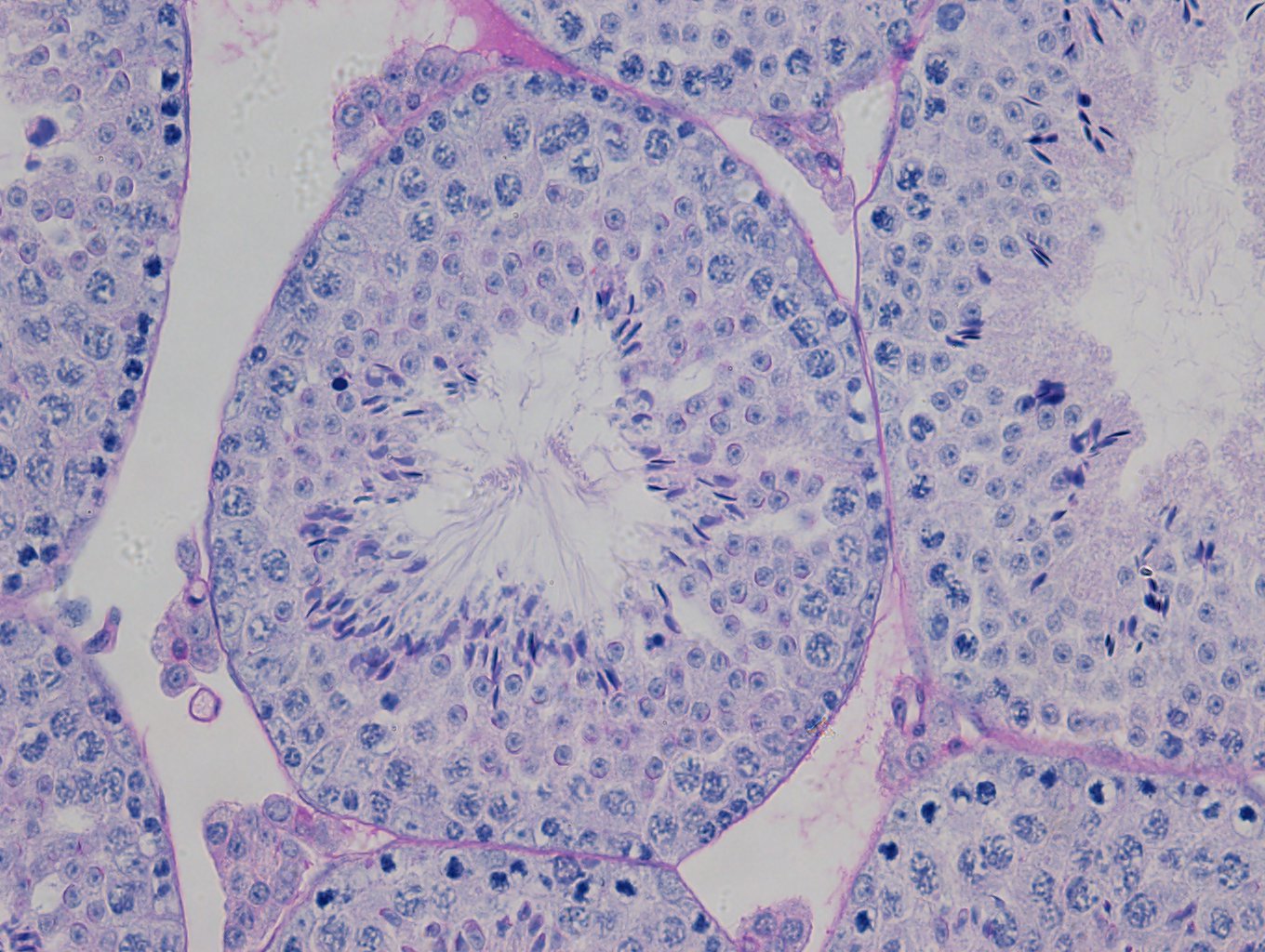

Supplement: Figure 2—source data 1. [file elife-83129-fig2-data1.zip › Figure2/Source data of Figure2A/Source data of WT-PAS staining/═╝╧±_39704.jpg]

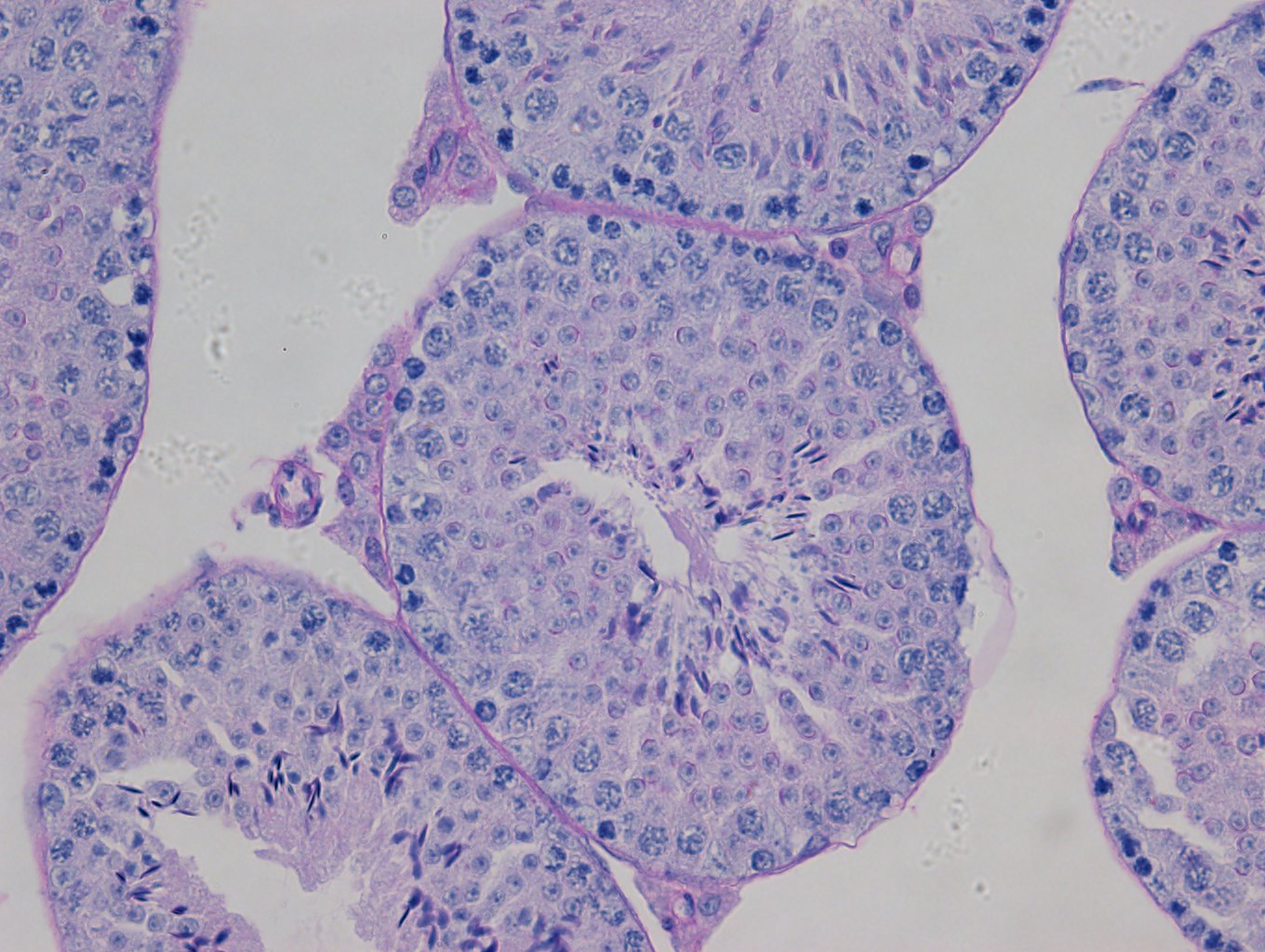

Supplement: Figure 2—source data 1. [file elife-83129-fig2-data1.zip › Figure2/Source data of Figure2A/Source data of WT-PAS staining/═╝╧±_39705.jpg]

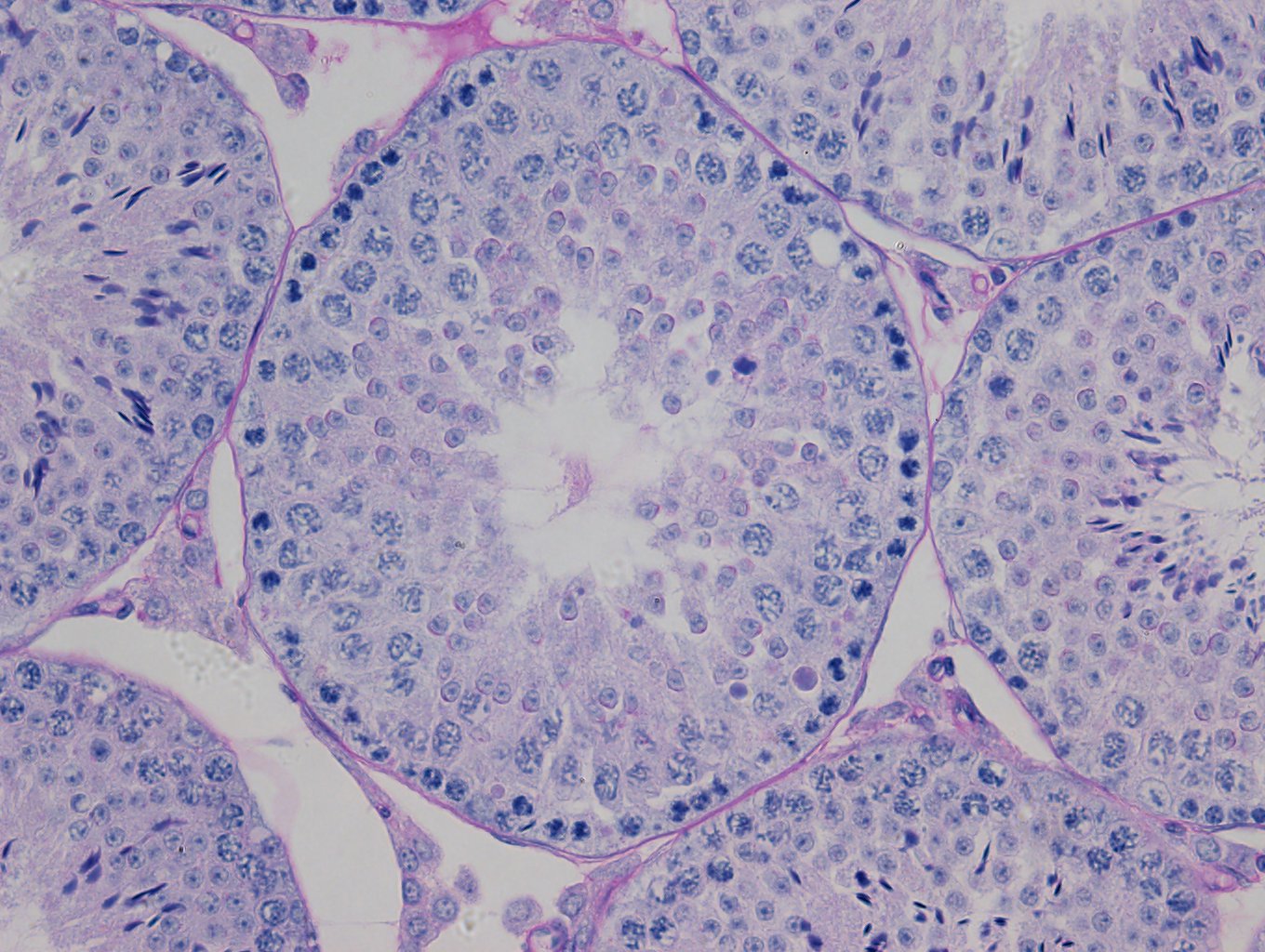

Supplement: Figure 2—source data 1. [file elife-83129-fig2-data1.zip › Figure2/Source data of Figure2A/Source data of WT-PAS staining/═╝╧±_39706.jpg]

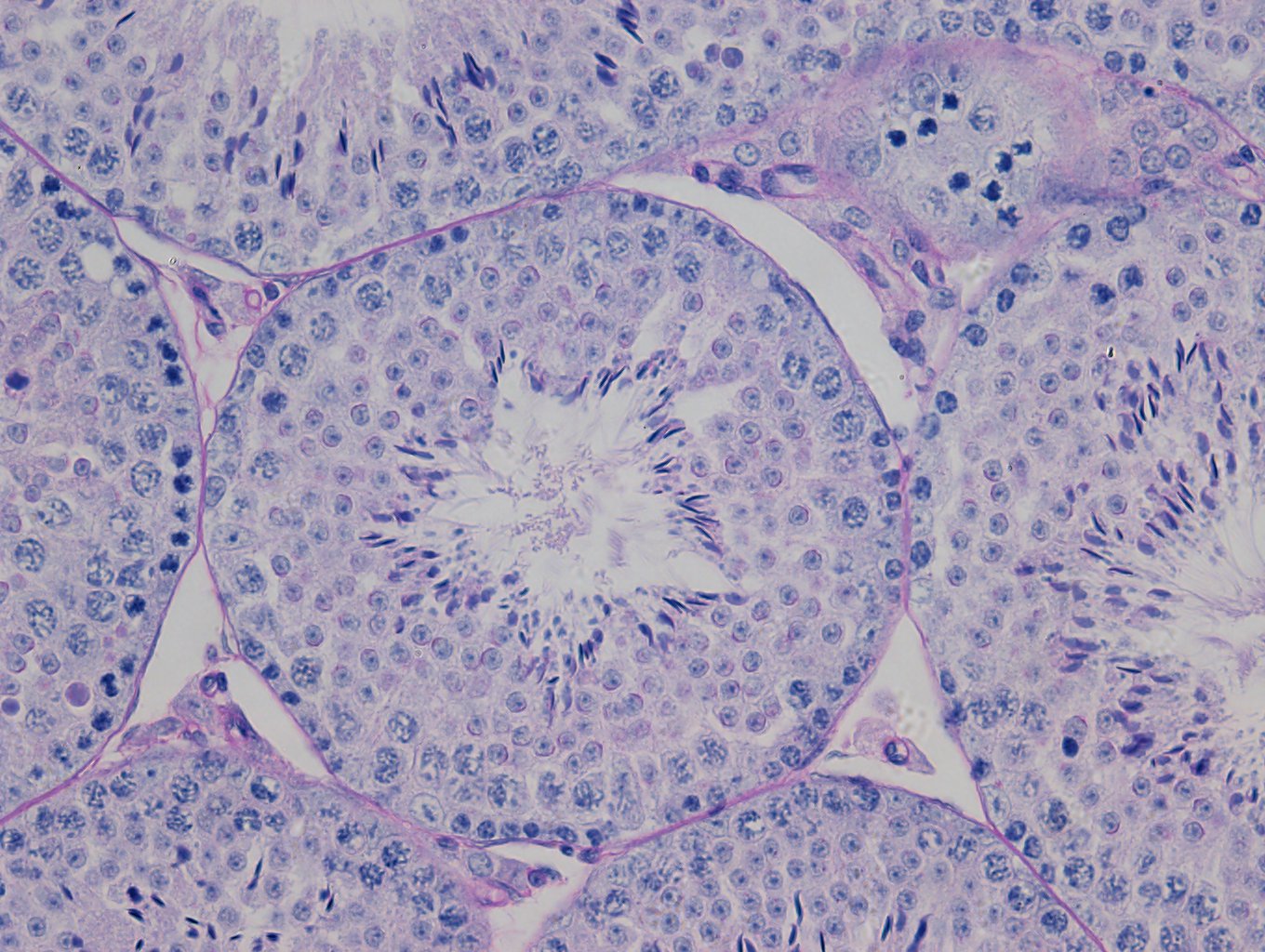

Supplement: Figure 2—source data 1. [file elife-83129-fig2-data1.zip › Figure2/Source data of Figure2A/Source data of WT-PAS staining/═╝╧±_39707.jpg]

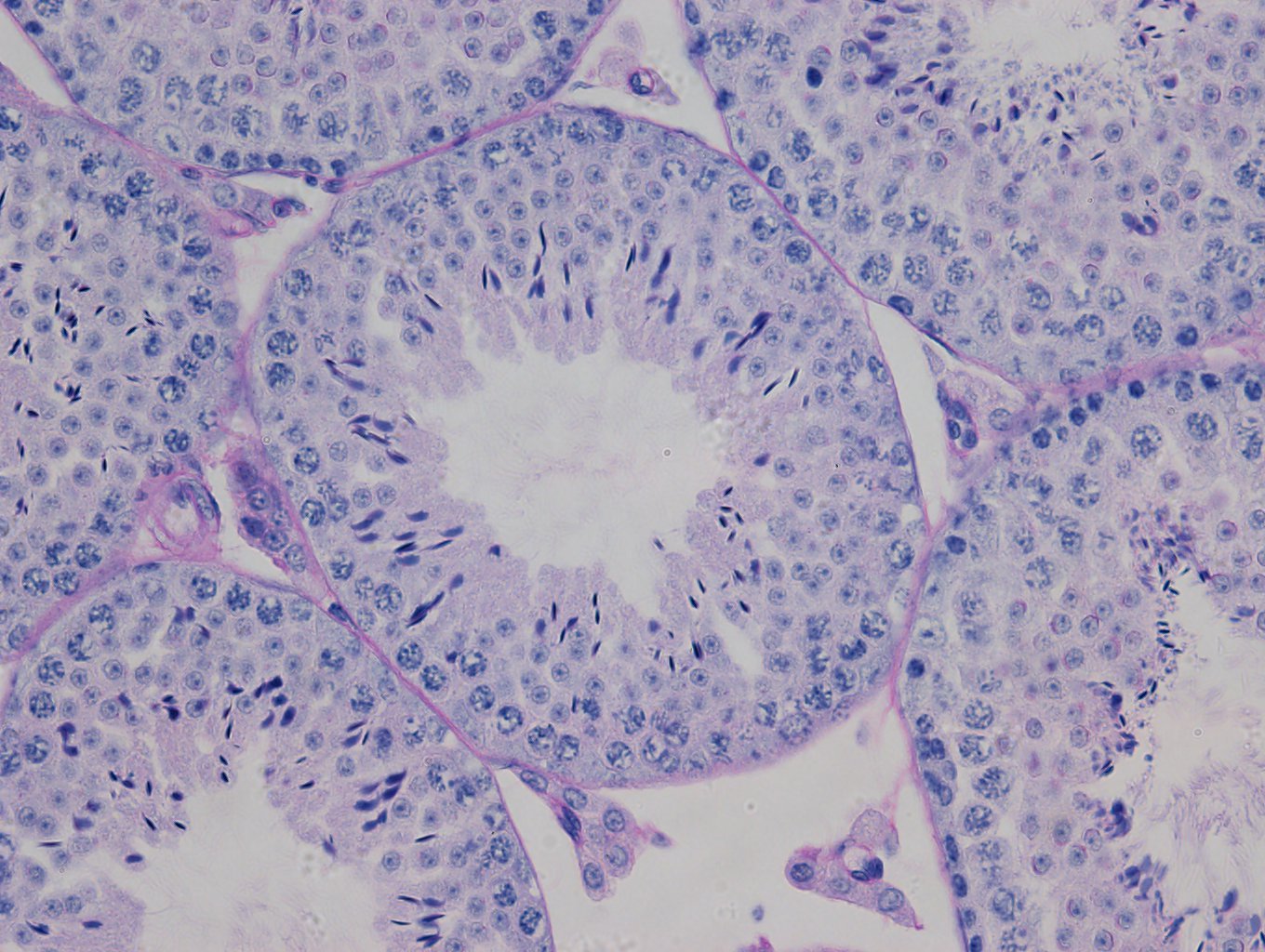

Supplement: Figure 2—source data 1. [file elife-83129-fig2-data1.zip › Figure2/Source data of Figure2A/Source data of WT-PAS staining/═╝╧±_39708.jpg]

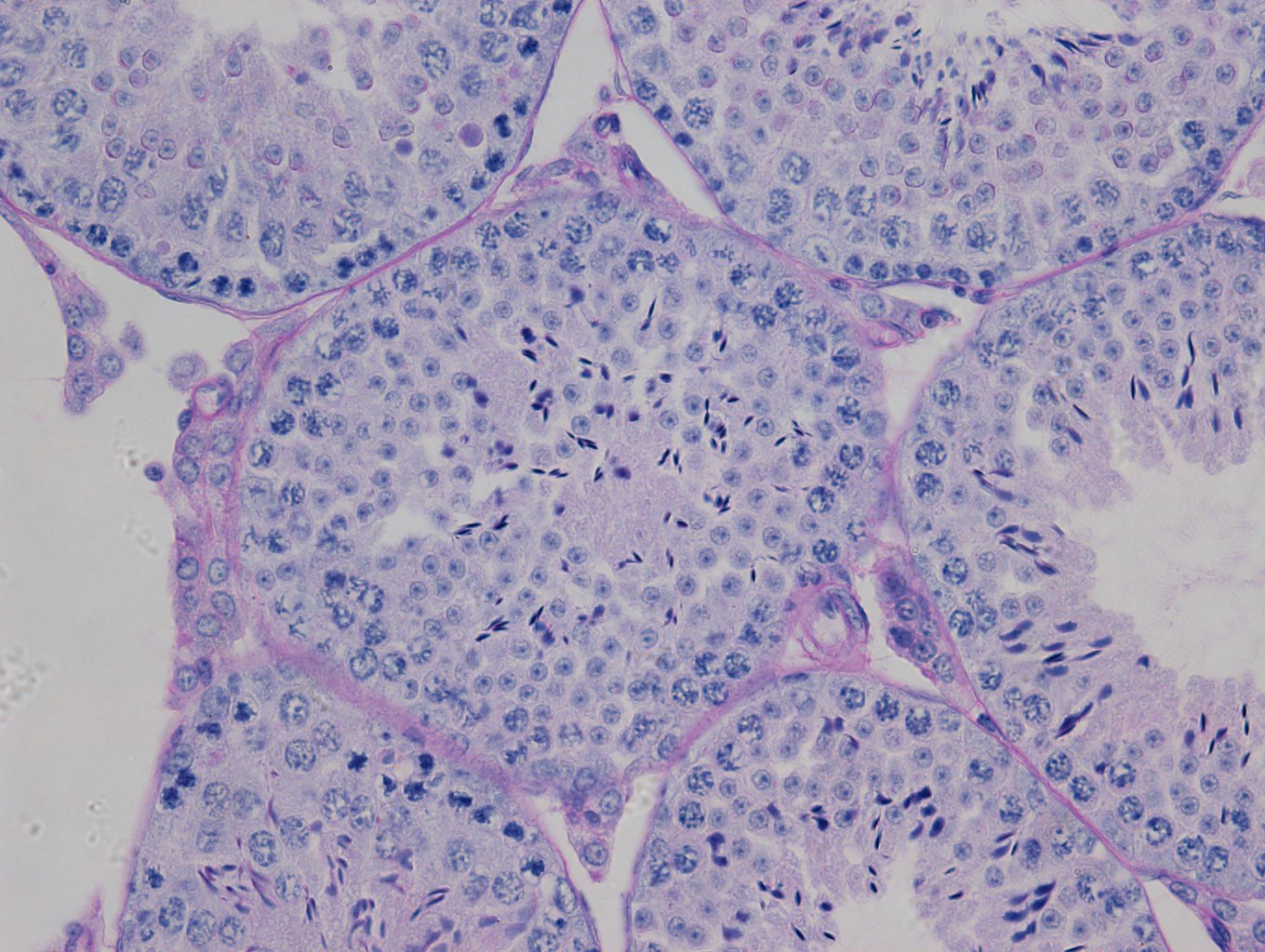

Supplement: Figure 2—source data 1. [file elife-83129-fig2-data1.zip › Figure2/Source data of Figure2A/Source data of WT-PAS staining/═╝╧±_39709.jpg]

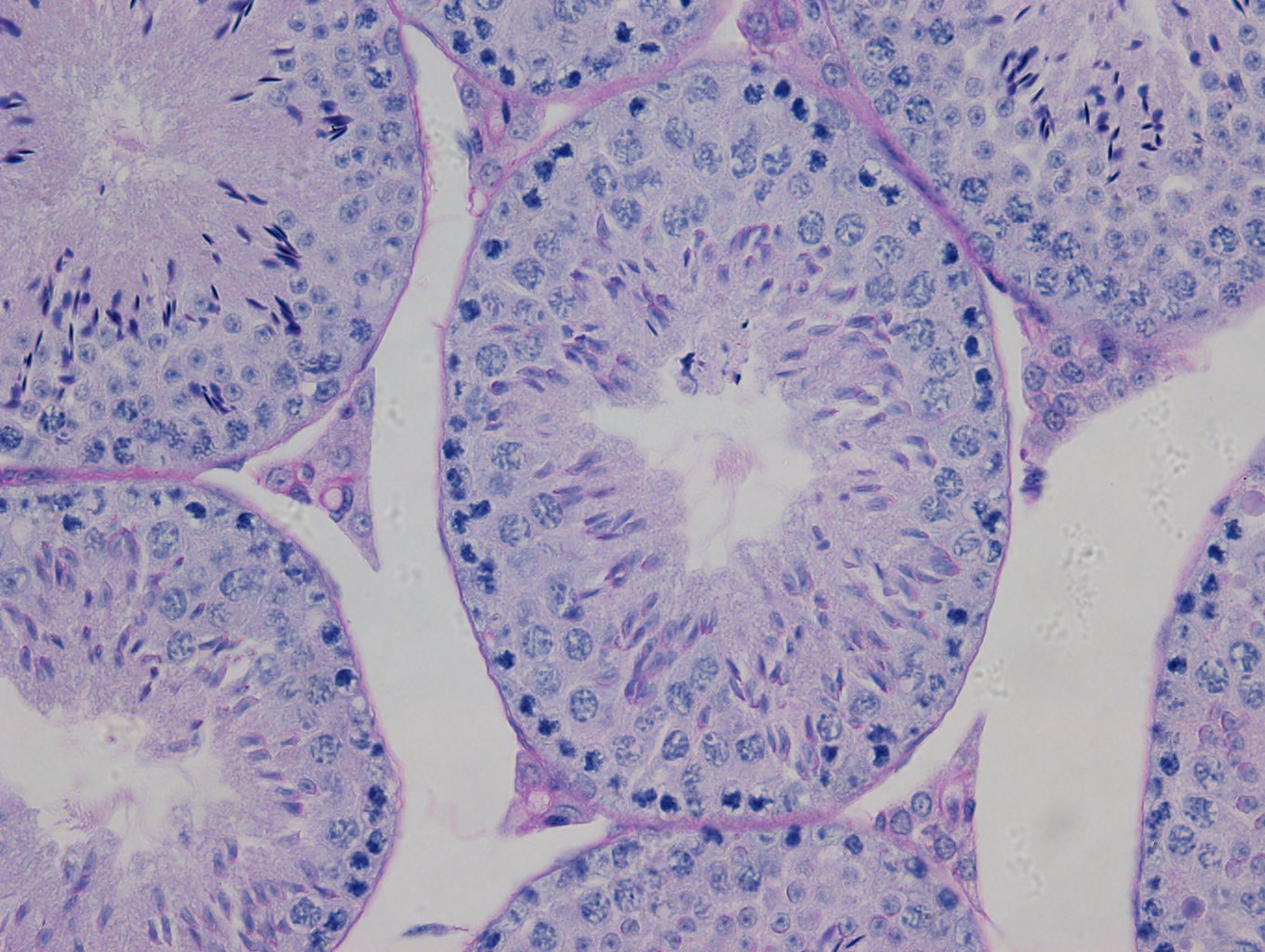

Supplement: Figure 2—source data 1. [file elife-83129-fig2-data1.zip › Figure2/Source data of Figure2A/Source data of WT-PAS staining/═╝╧±_39710.jpg]

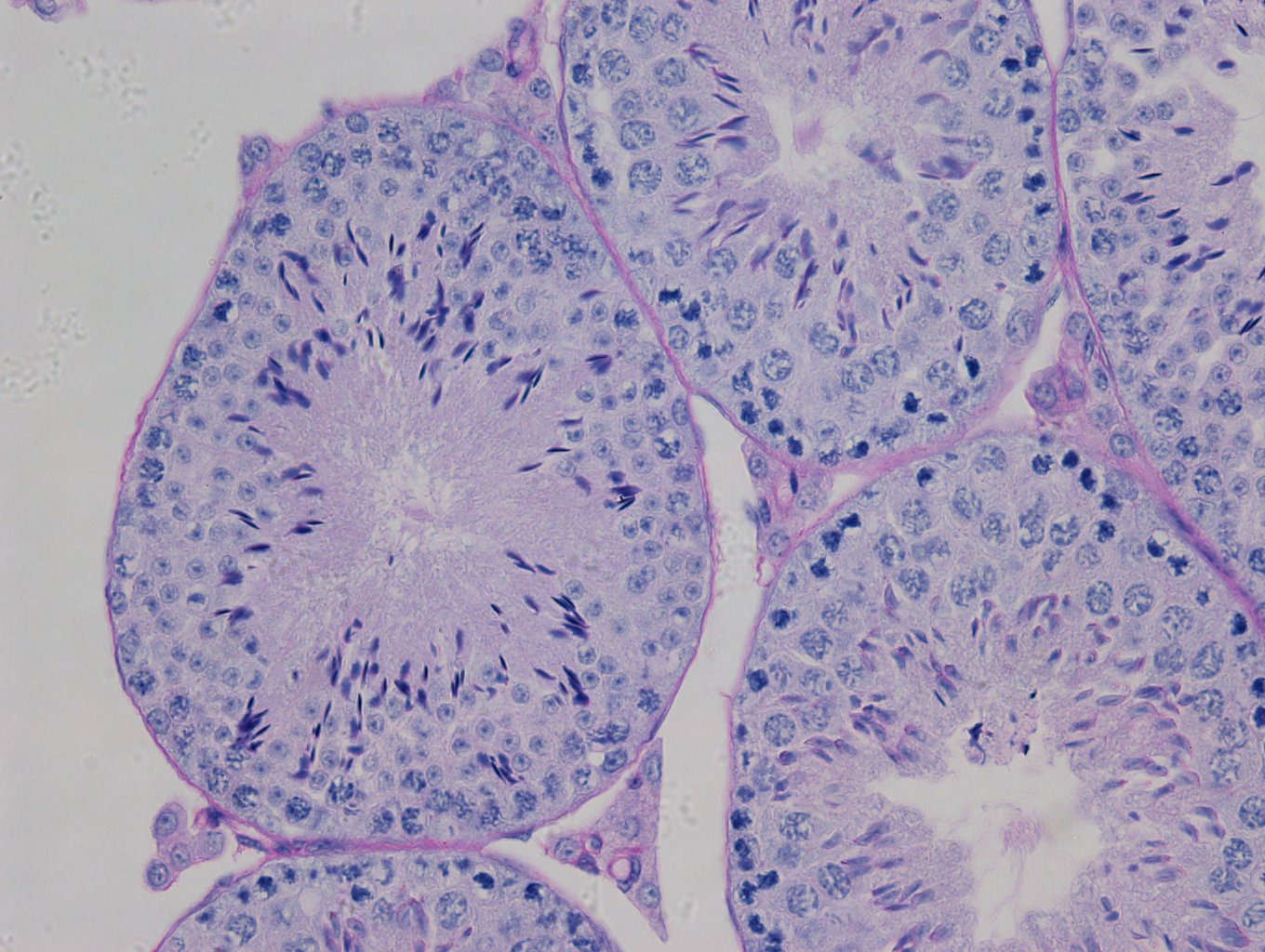

Supplement: Figure 2—source data 1. [file elife-83129-fig2-data1.zip › Figure2/Source data of Figure2A/Source data of WT-PAS staining/═╝╧±_39711.jpg]

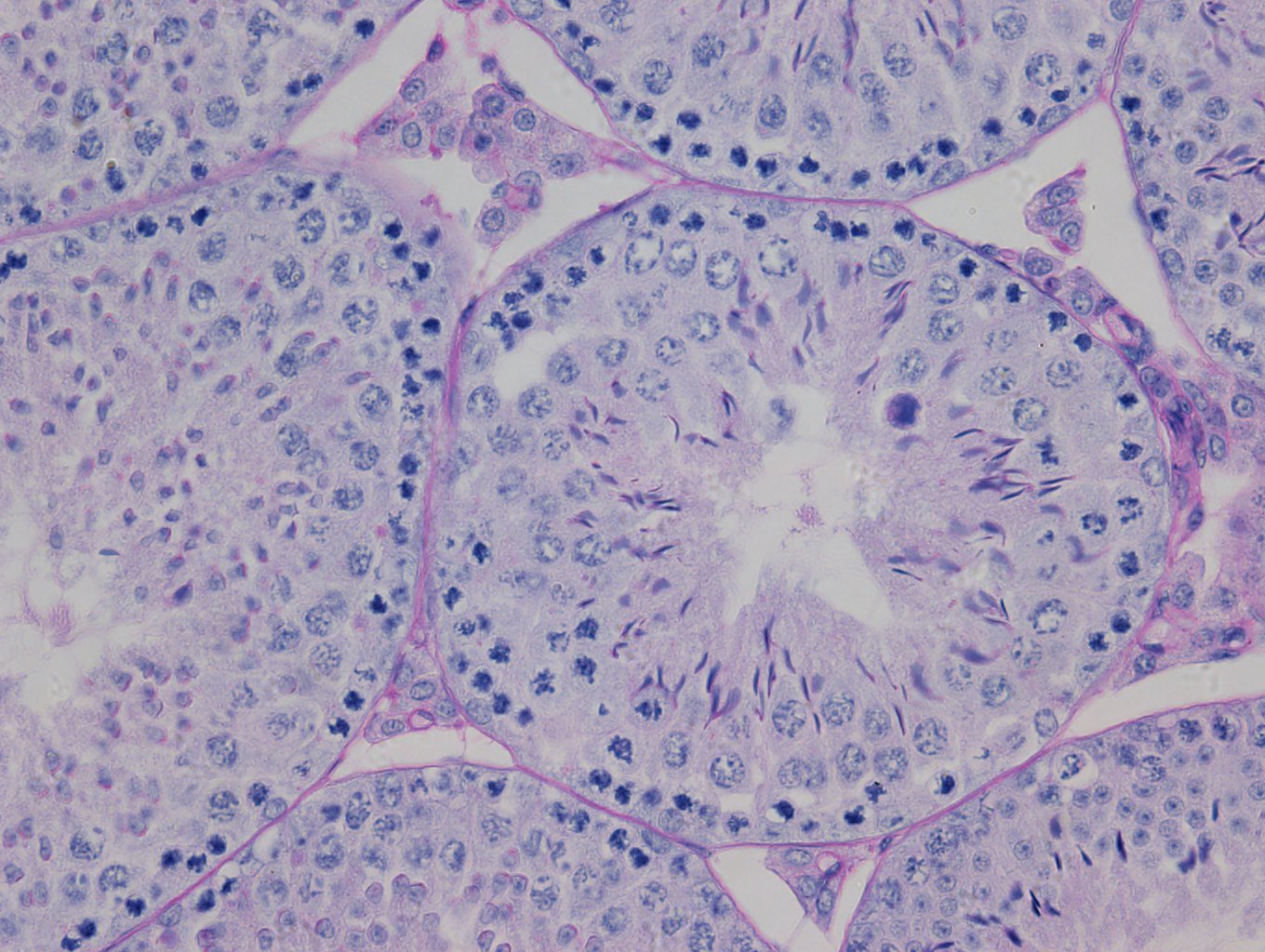

Supplement: Figure 2—source data 1. [file elife-83129-fig2-data1.zip › Figure2/Source data of Figure2A/Source data of WT-PAS staining/═╝╧±_39712.jpg]

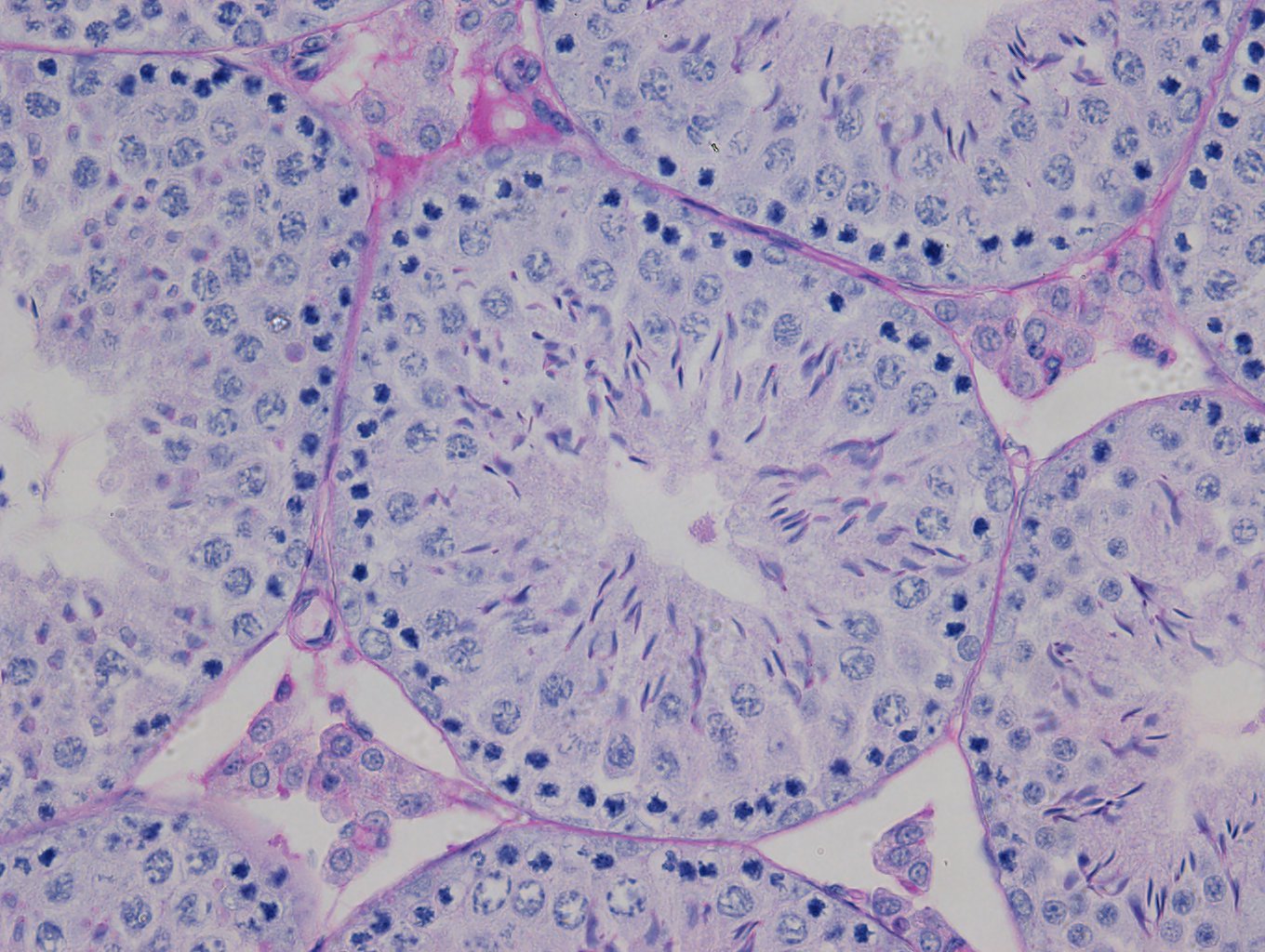

Supplement: Figure 2—source data 1. [file elife-83129-fig2-data1.zip › Figure2/Source data of Figure2A/Source data of WT-PAS staining/═╝╧±_39713.jpg]

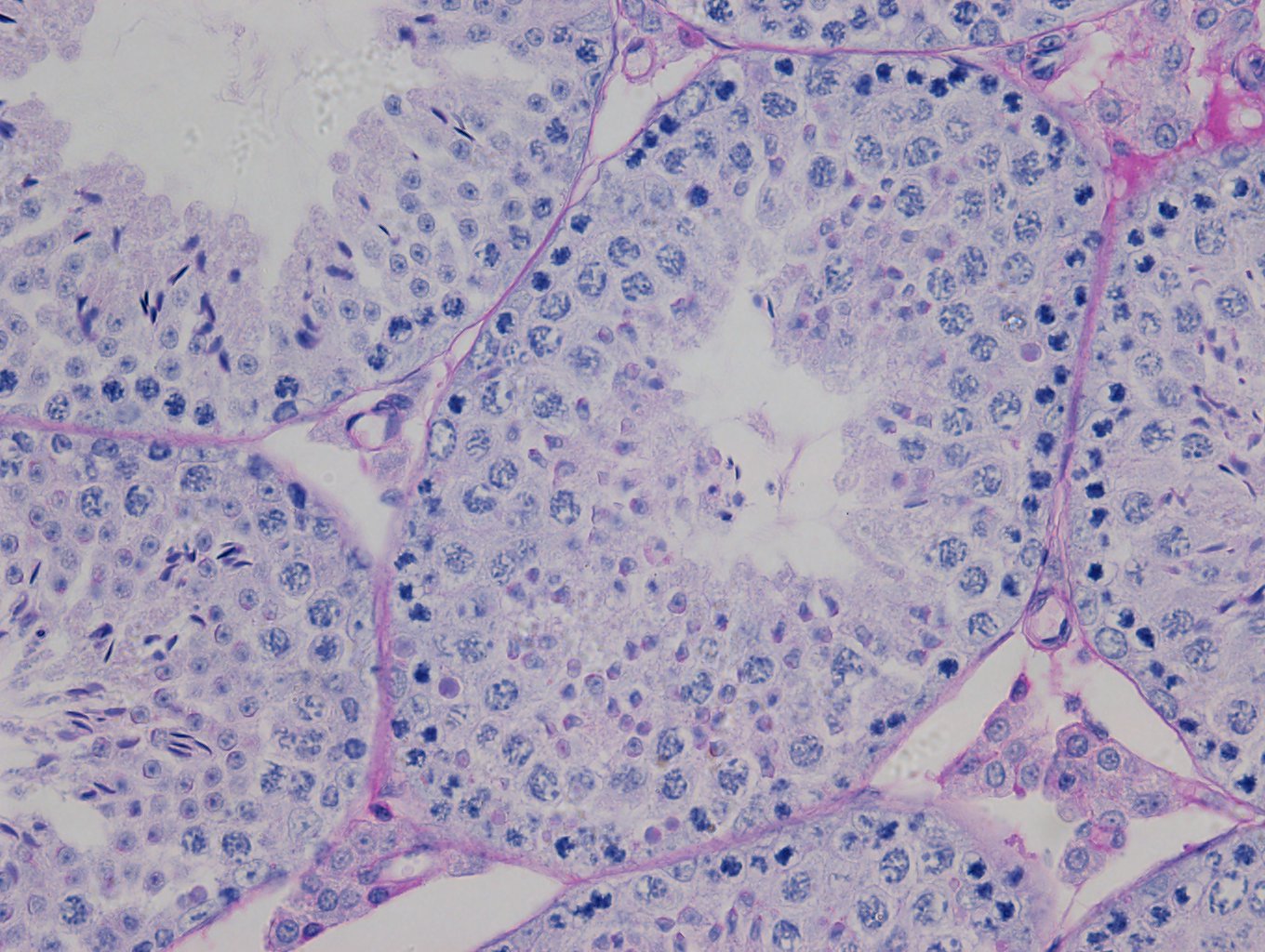

Supplement: Figure 2—source data 1. [file elife-83129-fig2-data1.zip › Figure2/Source data of Figure2A/Source data of WT-PAS staining/═╝╧±_39714.jpg]

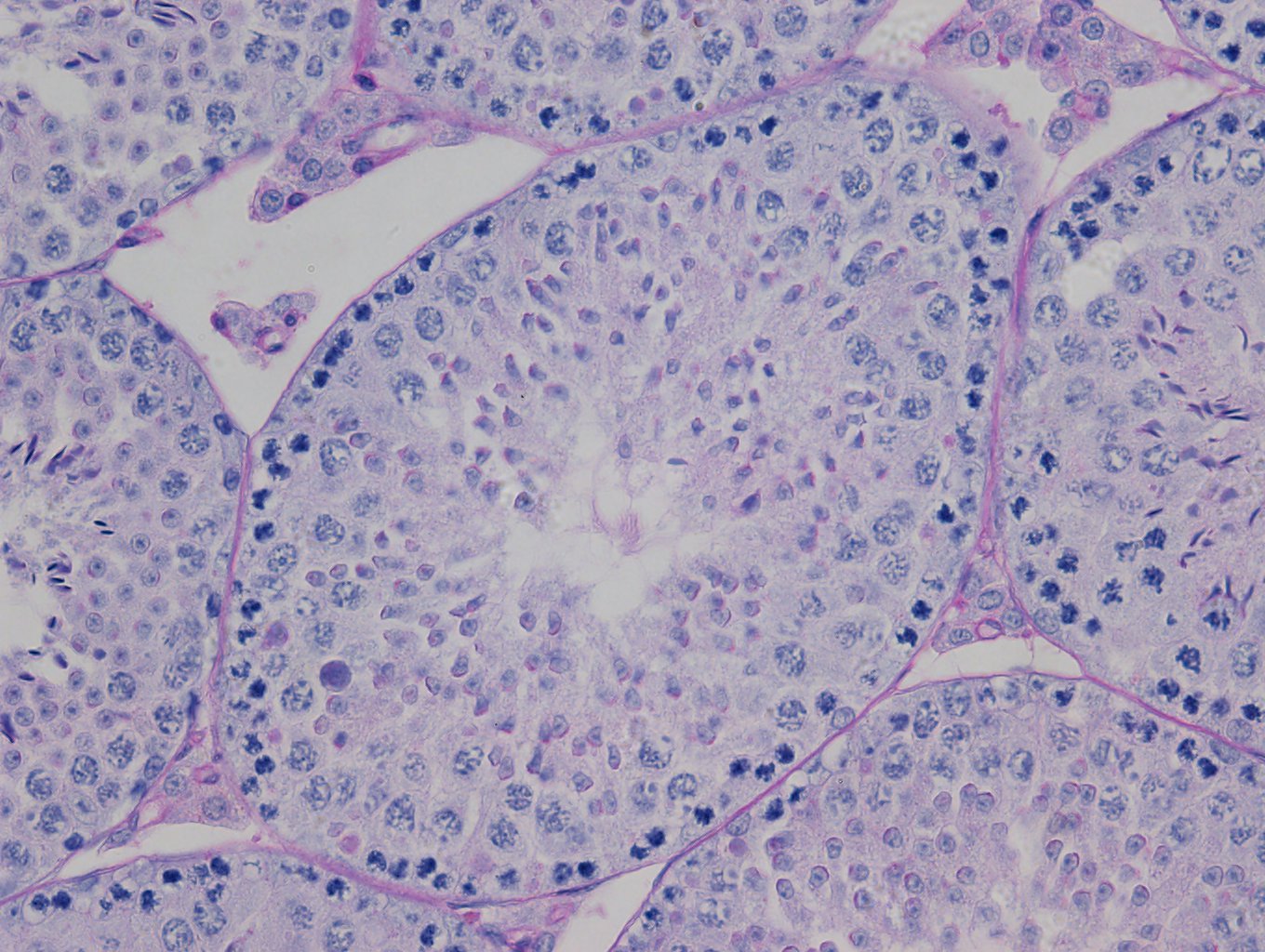

Supplement: Figure 2—source data 1. [file elife-83129-fig2-data1.zip › Figure2/Source data of Figure2A/Source data of WT-PAS staining/═╝╧±_39715.jpg]

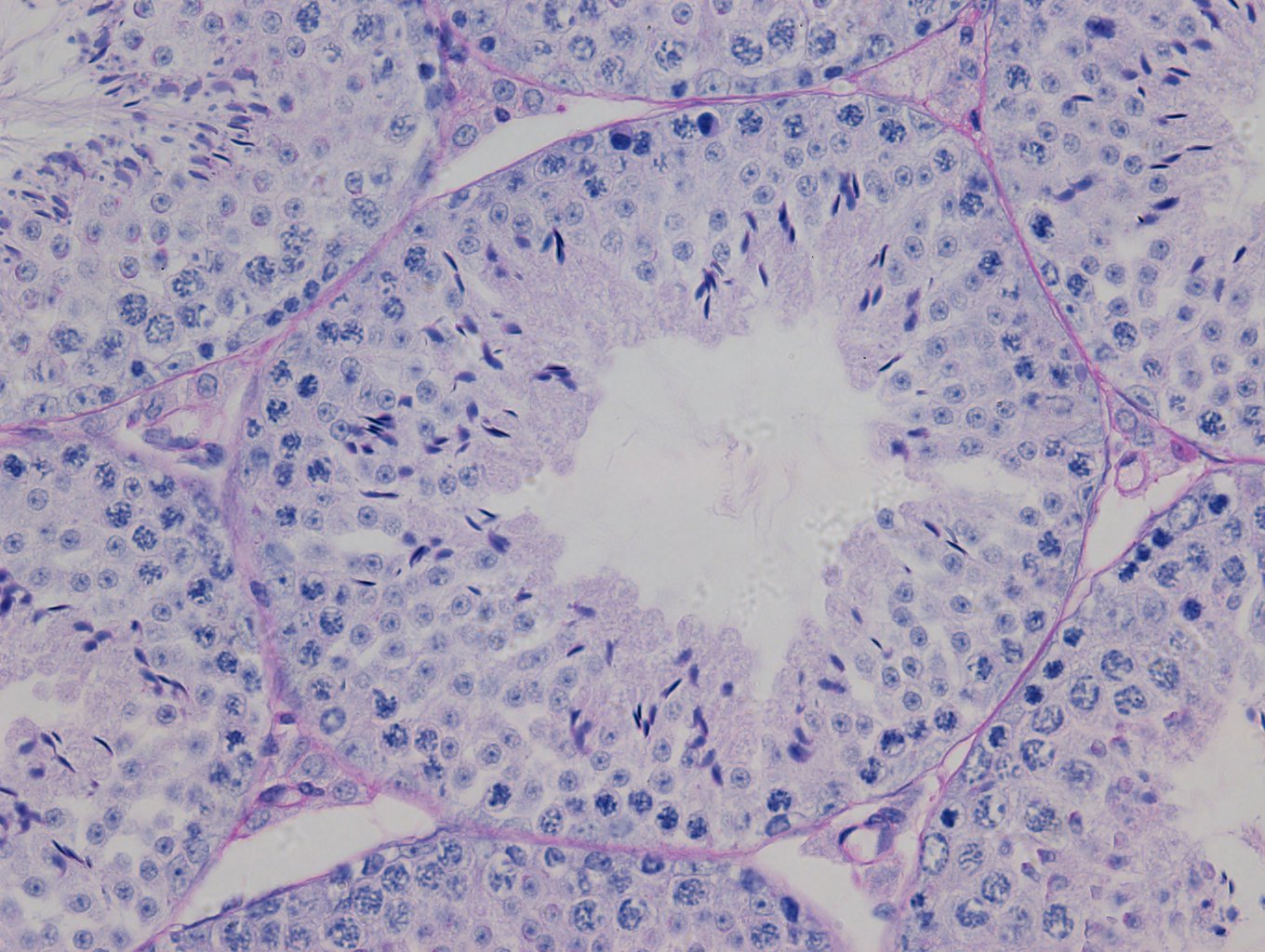

Supplement: Figure 2—source data 1. [file elife-83129-fig2-data1.zip › Figure2/Source data of Figure2A/Source data of WT-PAS staining/═╝╧±_39716.jpg]

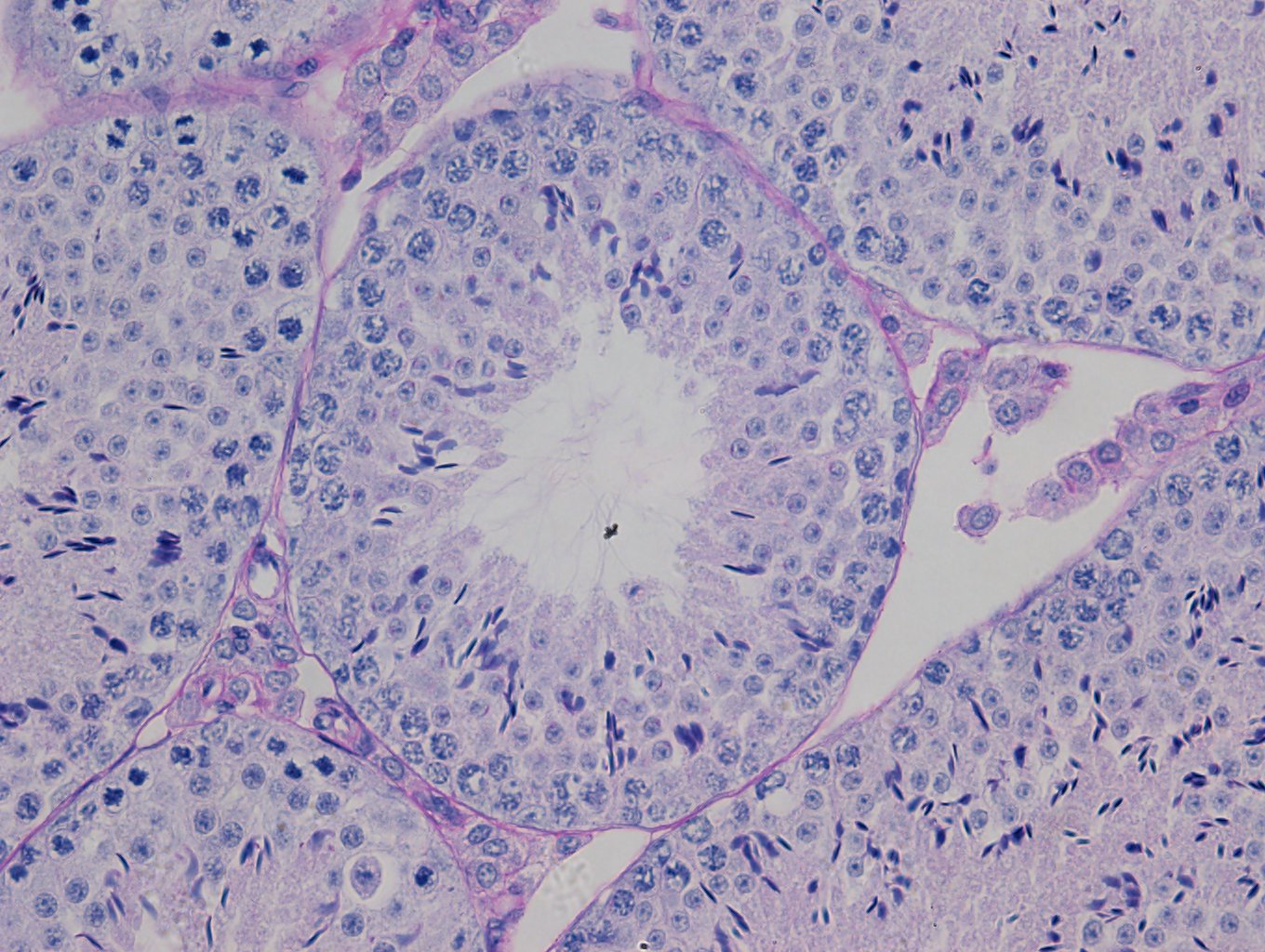

Supplement: Figure 2—source data 1. [file elife-83129-fig2-data1.zip › Figure2/Source data of Figure2A/Source data of WT-PAS staining/═╝╧±_39717.jpg]

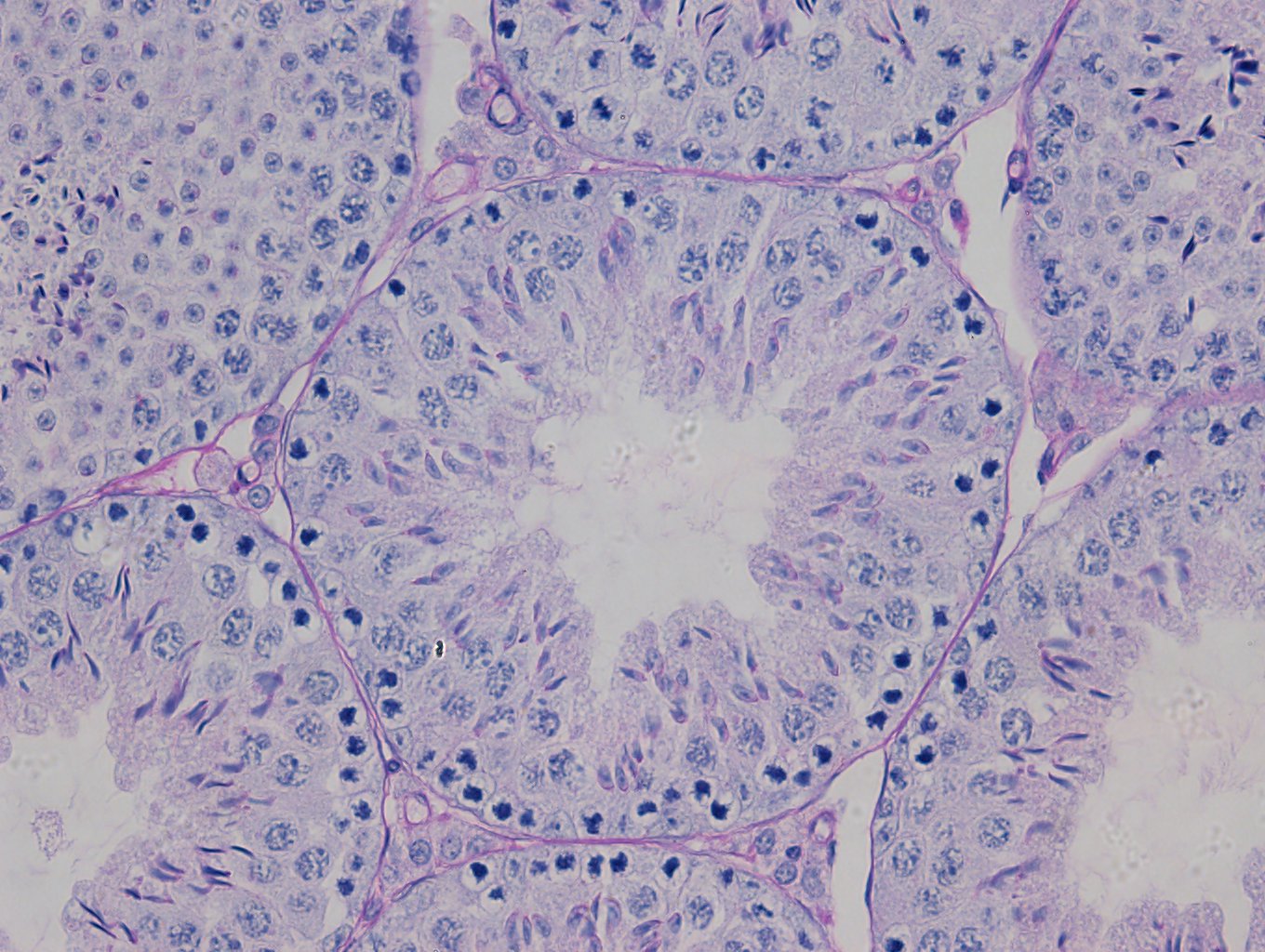

Supplement: Figure 2—source data 1. [file elife-83129-fig2-data1.zip › Figure2/Source data of Figure2A/Source data of WT-PAS staining/═╝╧±_39718.jpg]

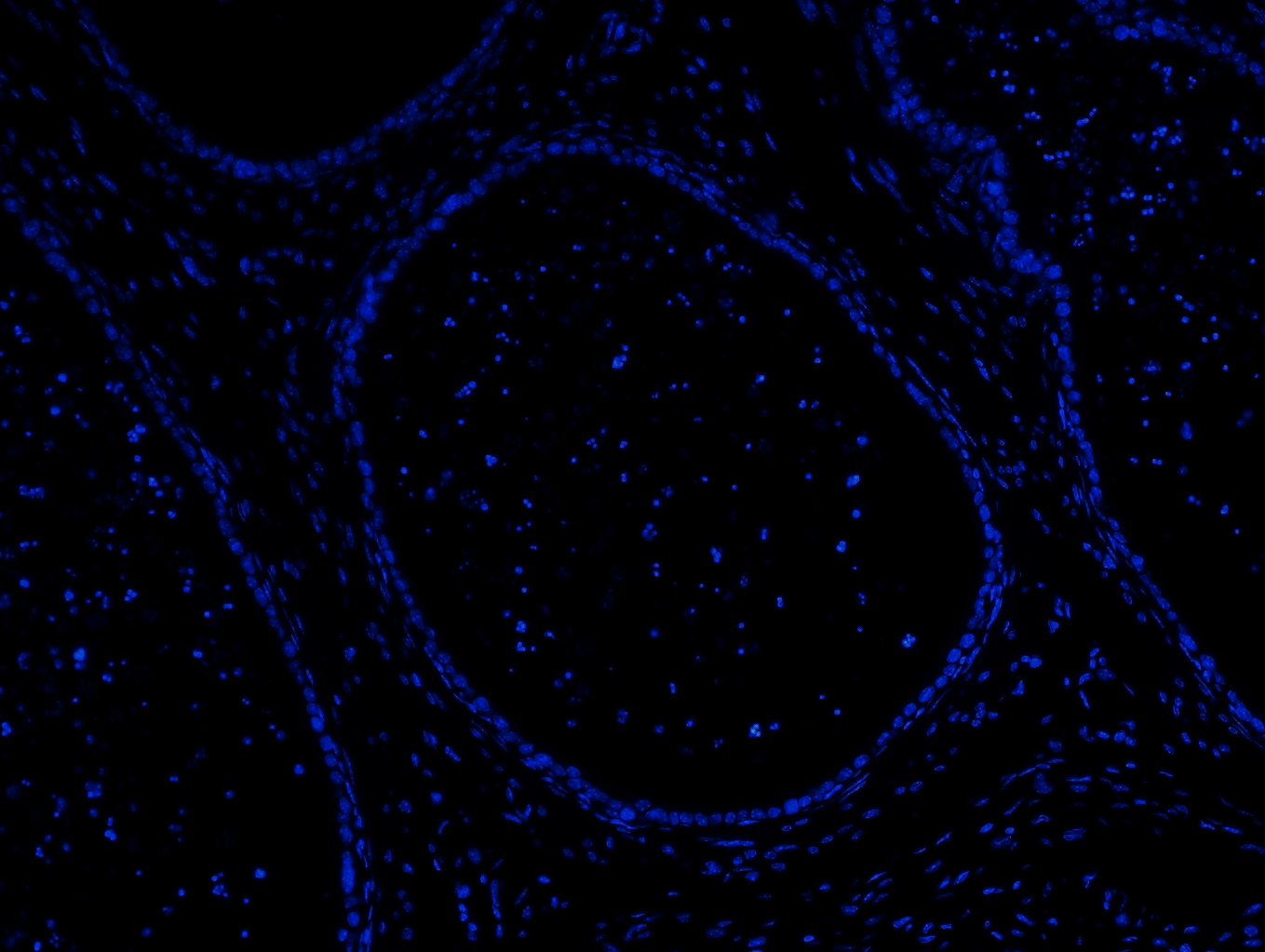

Supplement: Figure 2—source data 1. [file elife-83129-fig2-data1.zip › Figure2/Source data of Figure2B/KO-epi/KO-epi-DAPI-20X-1.tif]

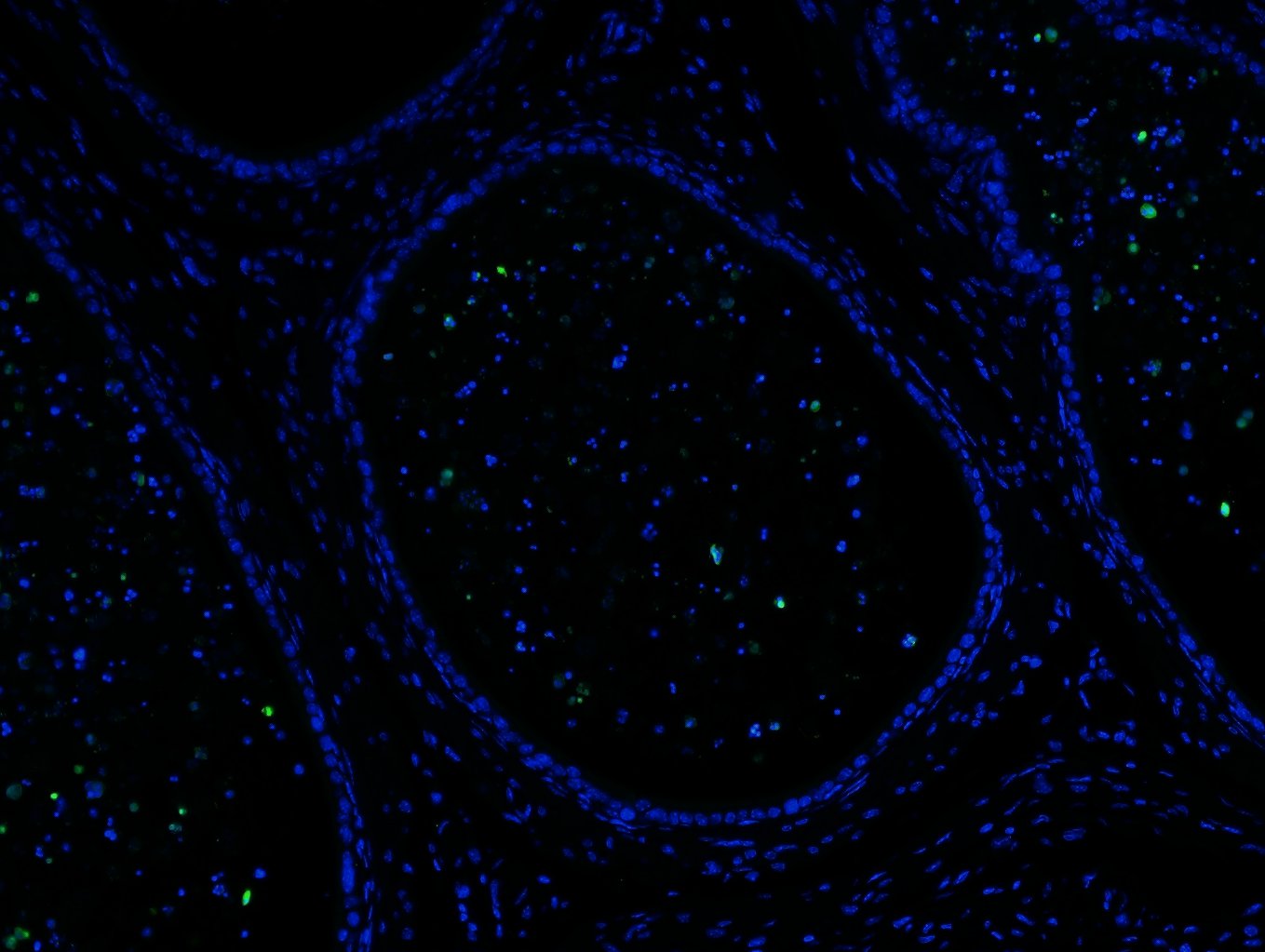

Supplement: Figure 2—source data 1. [file elife-83129-fig2-data1.zip › Figure2/Source data of Figure2B/KO-epi/KO-epi-TUNEL-20X-1.jpg]

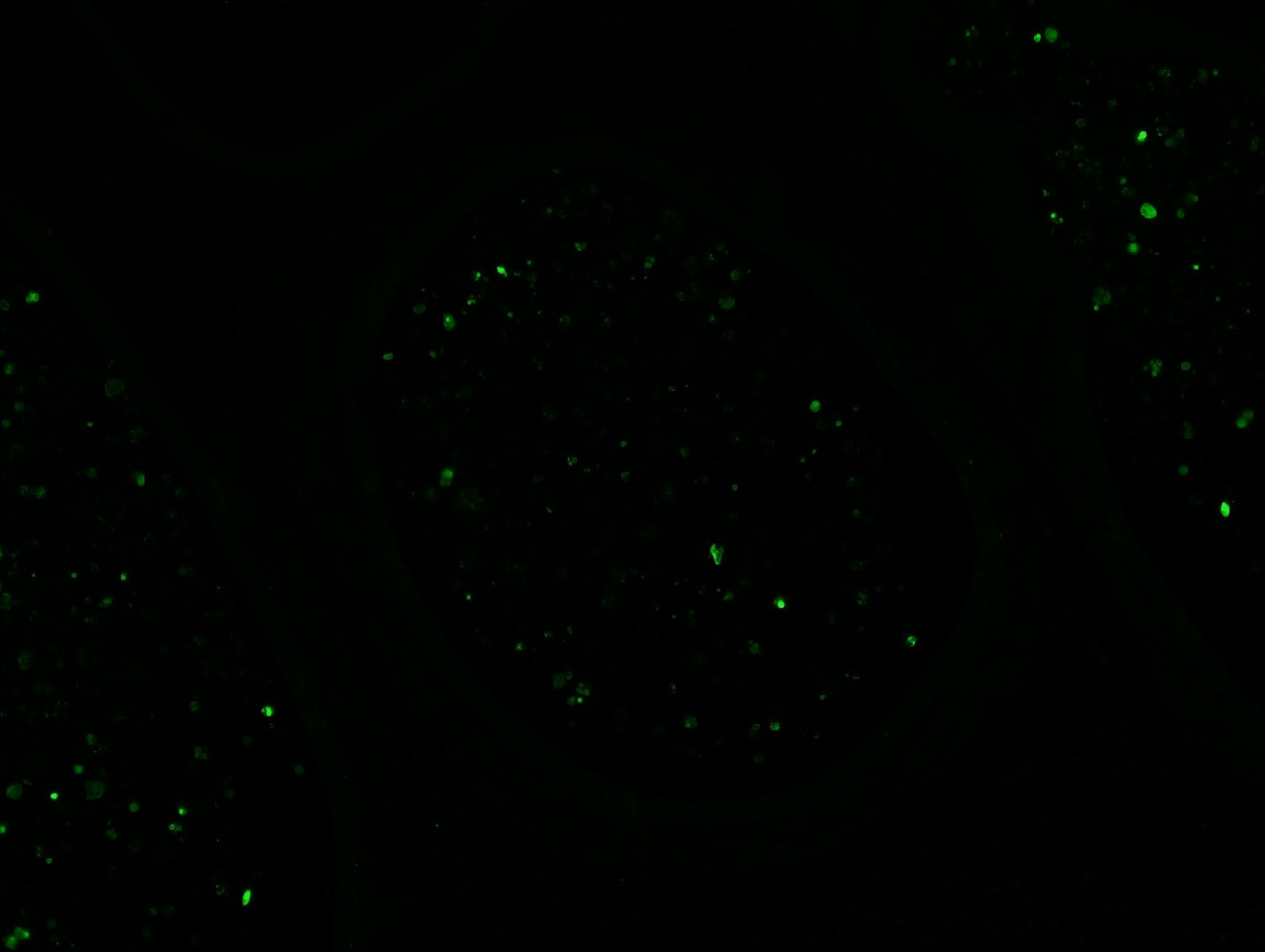

Supplement: Figure 2—source data 1. [file elife-83129-fig2-data1.zip › Figure2/Source data of Figure2B/KO-epi/KO-epi-TUNEL-20X-1.tif]

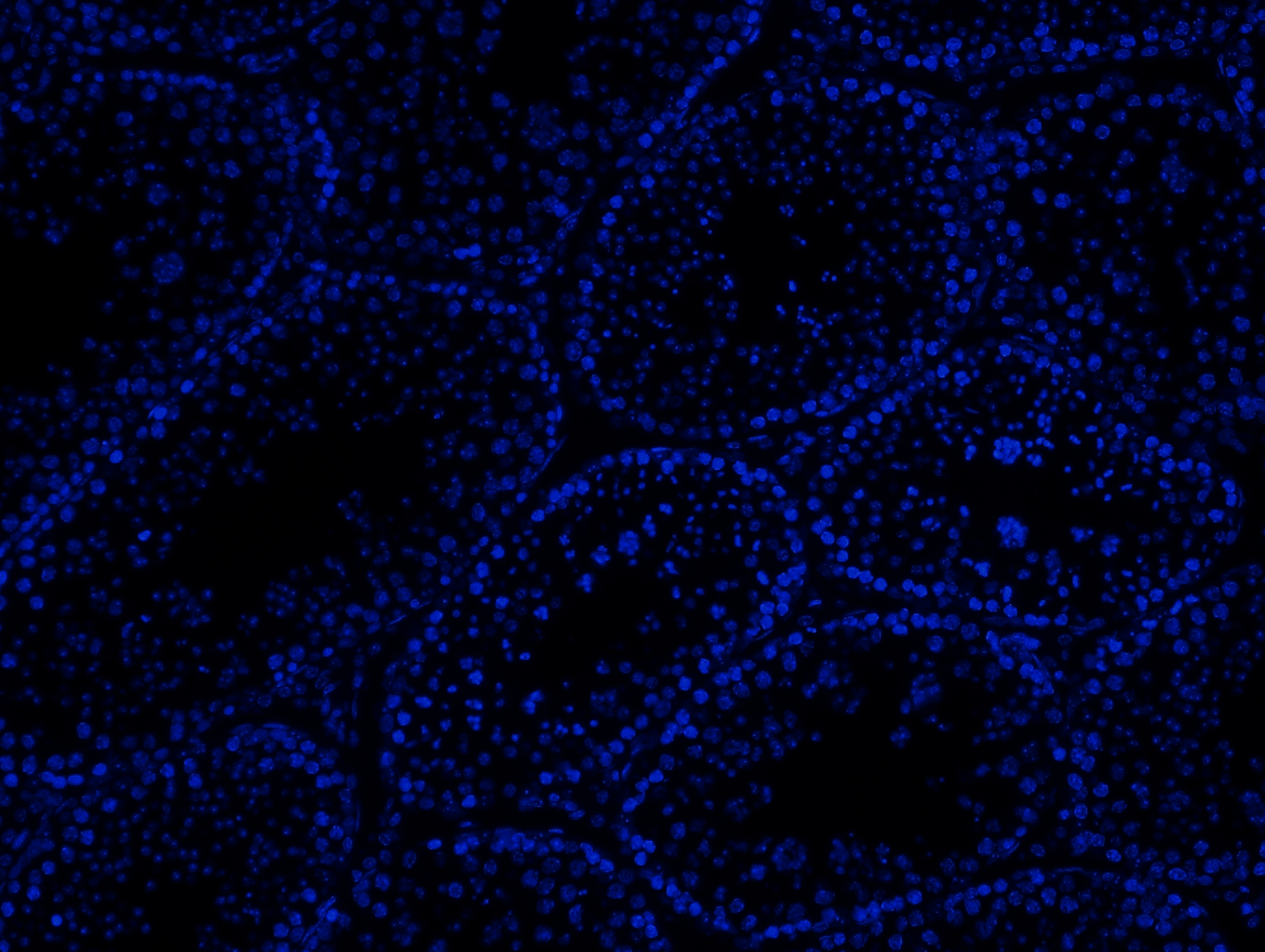

Supplement: Figure 2—source data 1. [file elife-83129-fig2-data1.zip › Figure2/Source data of Figure2B/KO-testis/KO-Testis-DAPI-20X-1.tif]

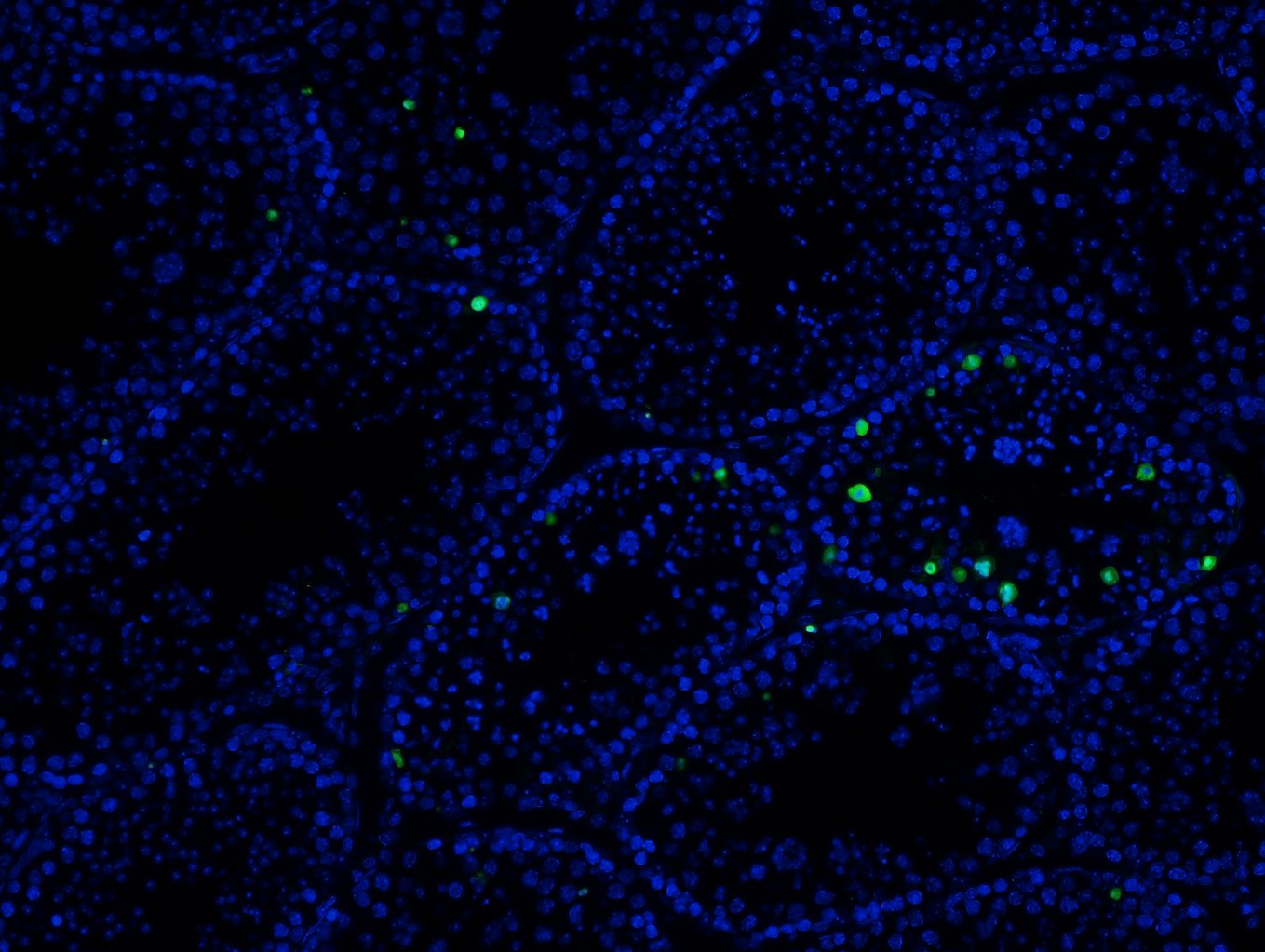

Supplement: Figure 2—source data 1. [file elife-83129-fig2-data1.zip › Figure2/Source data of Figure2B/KO-testis/KO-Testis-TUNEL-20X-1.jpg]

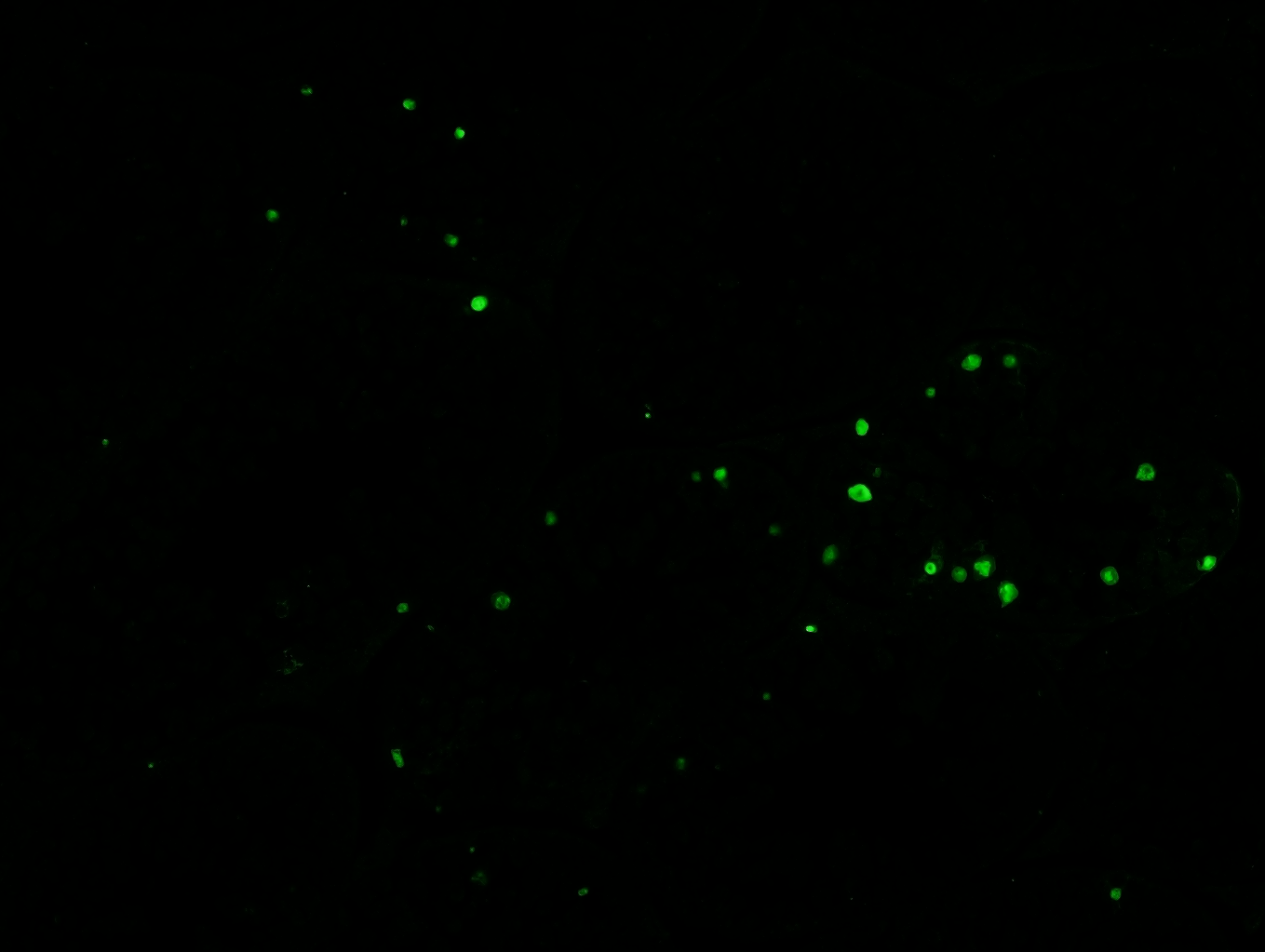

Supplement: Figure 2—source data 1. [file elife-83129-fig2-data1.zip › Figure2/Source data of Figure2B/KO-testis/KO-Testis-TUNEL-20X-1.tif]

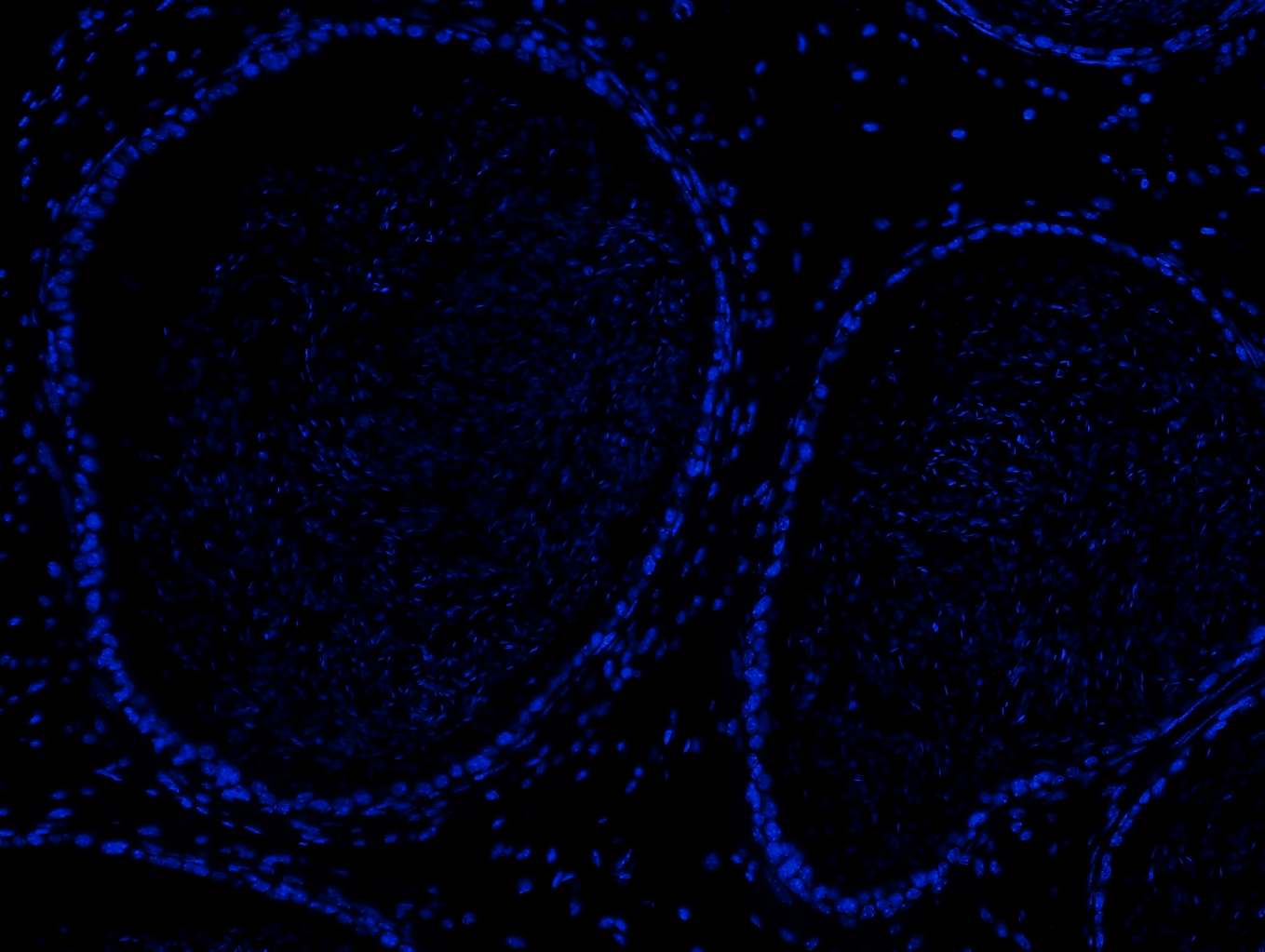

Supplement: Figure 2—source data 1. [file elife-83129-fig2-data1.zip › Figure2/Source data of Figure2B/WT-epi/WT-epi-DAPI-20X-1.tif]

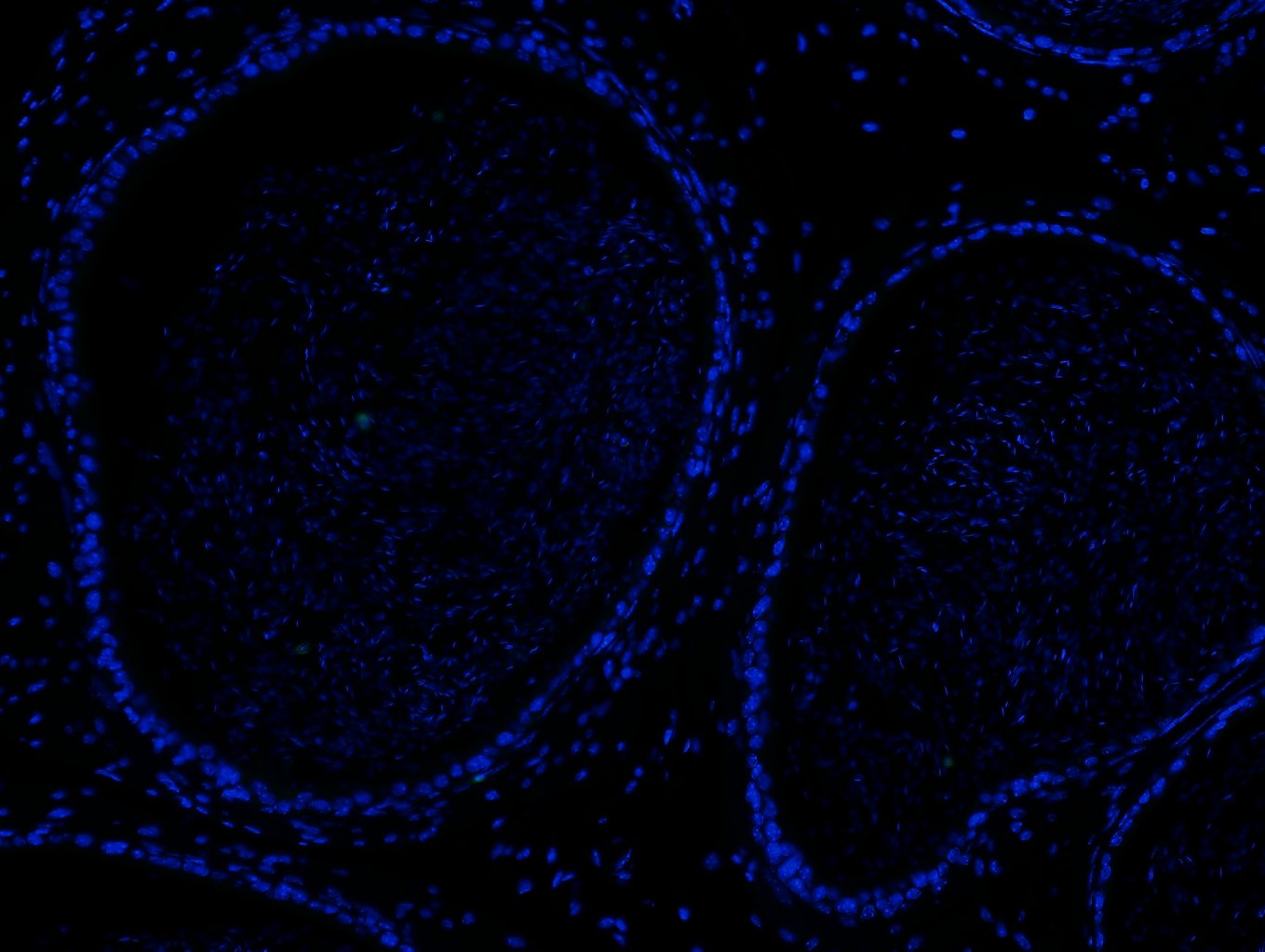

Supplement: Figure 2—source data 1. [file elife-83129-fig2-data1.zip › Figure2/Source data of Figure2B/WT-epi/WT-epi-TUNEL-20X-1.jpg]

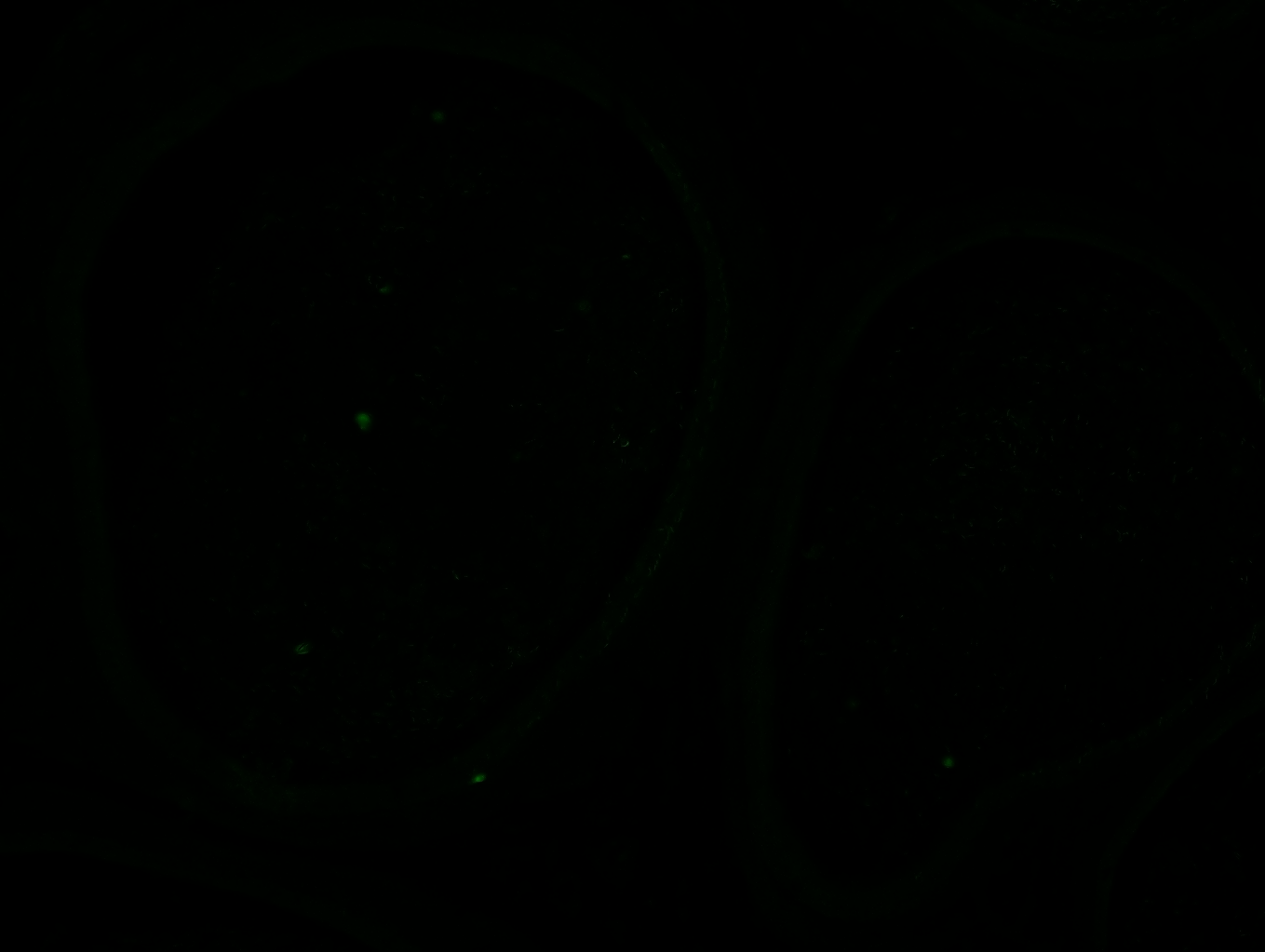

Supplement: Figure 2—source data 1. [file elife-83129-fig2-data1.zip › Figure2/Source data of Figure2B/WT-epi/WT-epi-TUNEL-20X-1.tif]

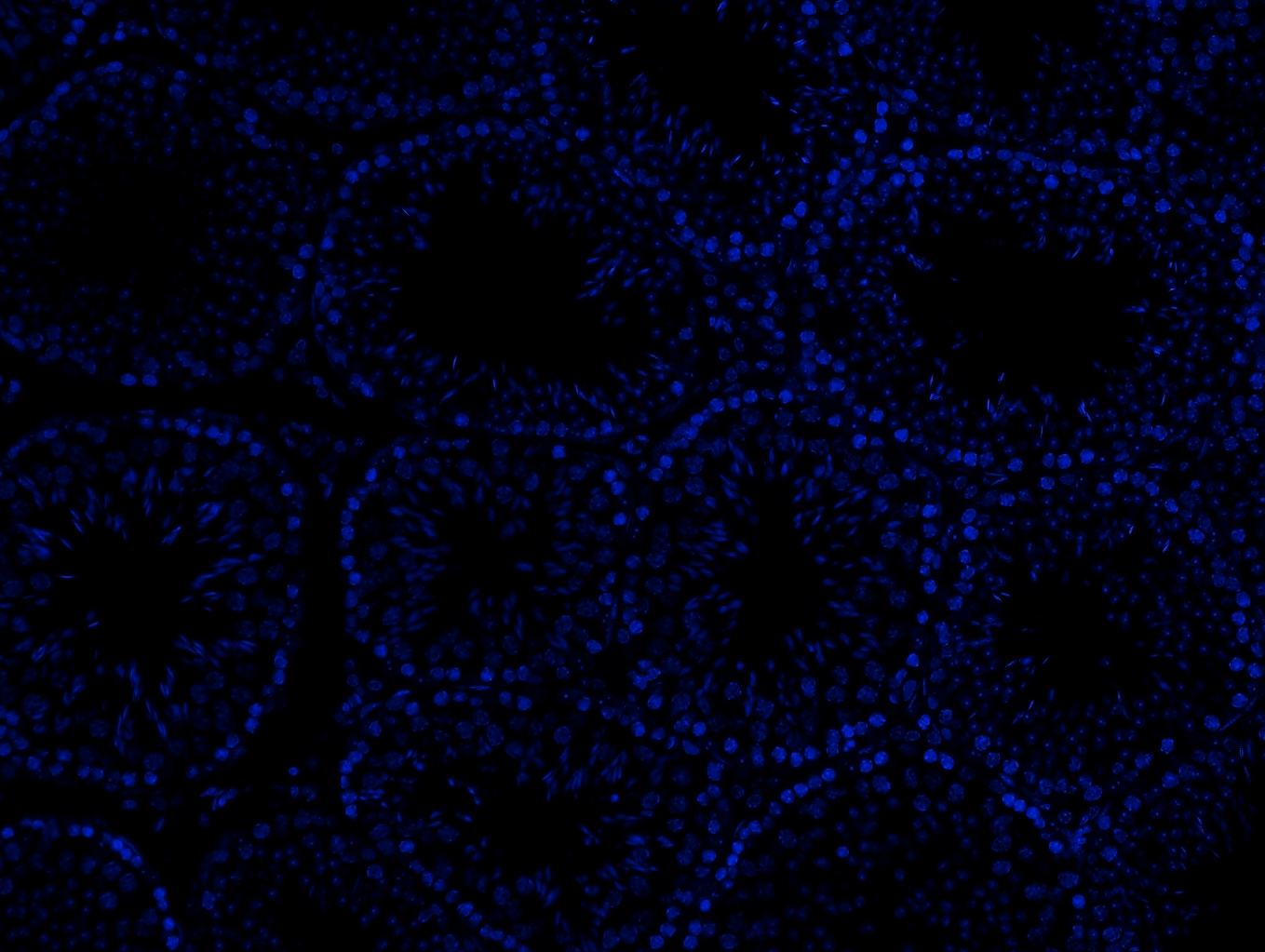

Supplement: Figure 2—source data 1. [file elife-83129-fig2-data1.zip › Figure2/Source data of Figure2B/WT-testis/WT-Testis-DAPI-20X-2.tif]

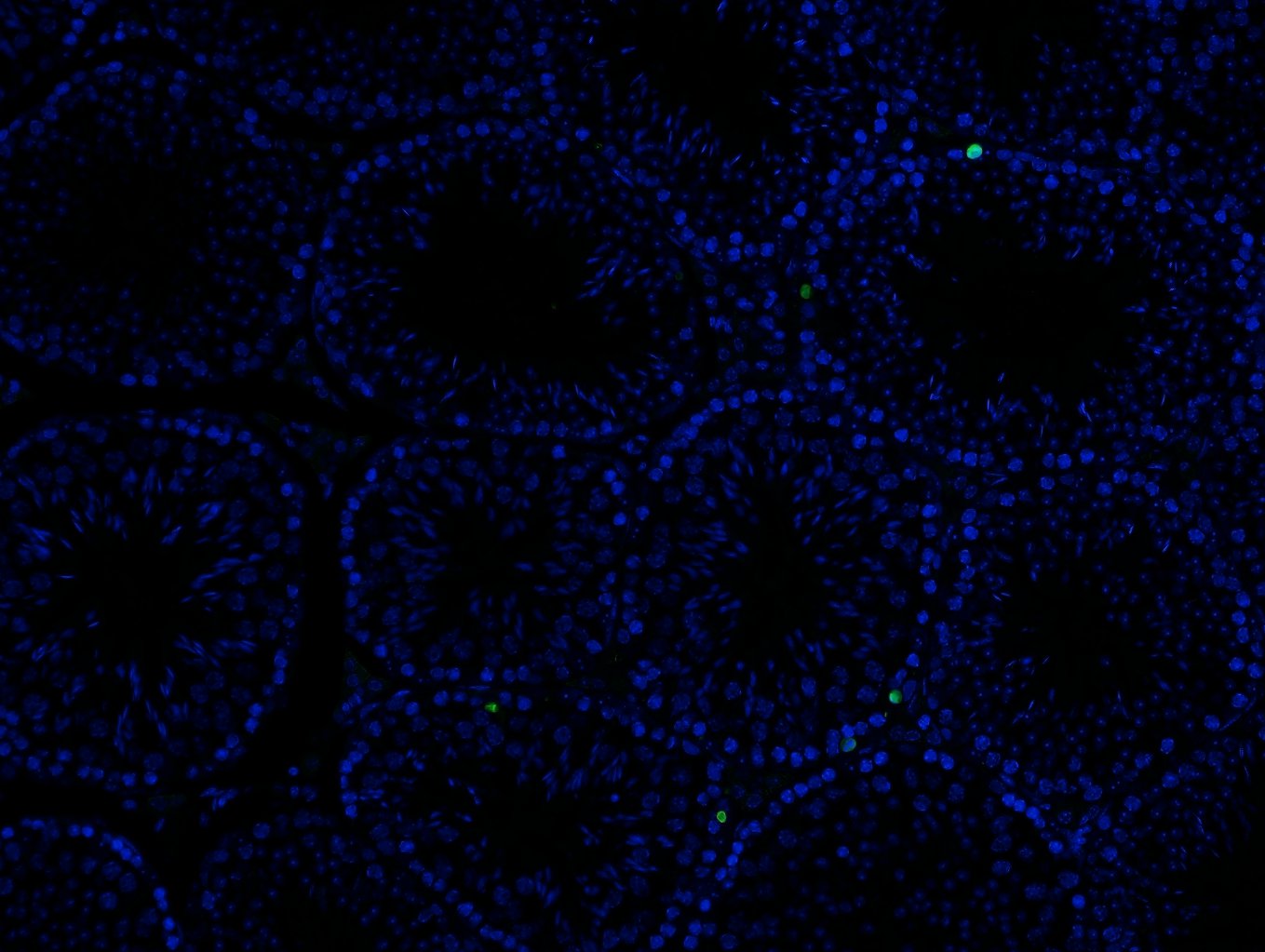

Supplement: Figure 2—source data 1. [file elife-83129-fig2-data1.zip › Figure2/Source data of Figure2B/WT-testis/WT-Testis-TUNEL-20X-2.jpg]

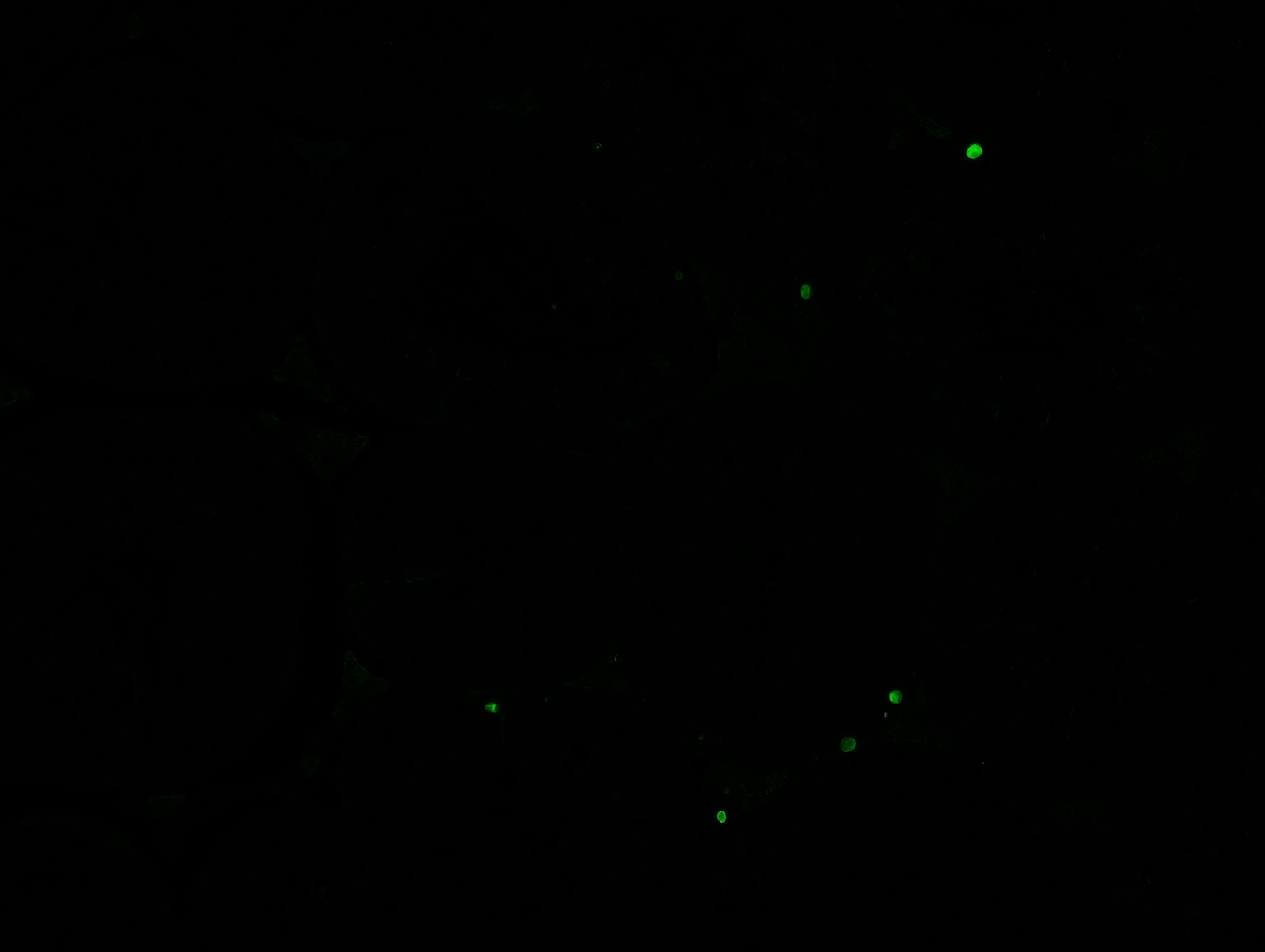

Supplement: Figure 2—source data 1. [file elife-83129-fig2-data1.zip › Figure2/Source data of Figure2B/WT-testis/WT-Testis-TUNEL-20X-2.tif]

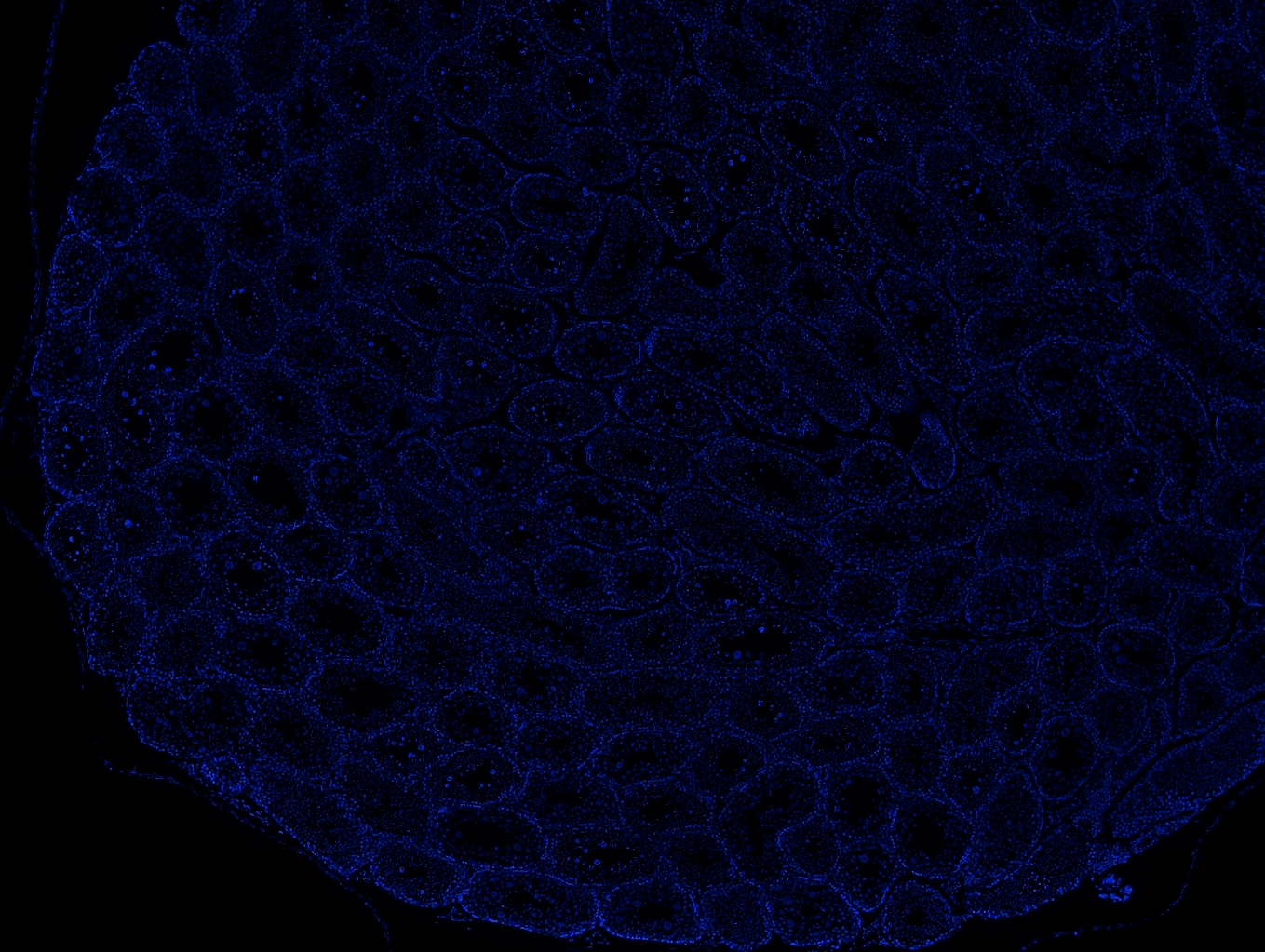

Supplement: Figure 2—source data 1. [file elife-83129-fig2-data1.zip › Figure2/Source data of Figure2C-2D/Count-KO-Testis-DAPI-4X.tif]

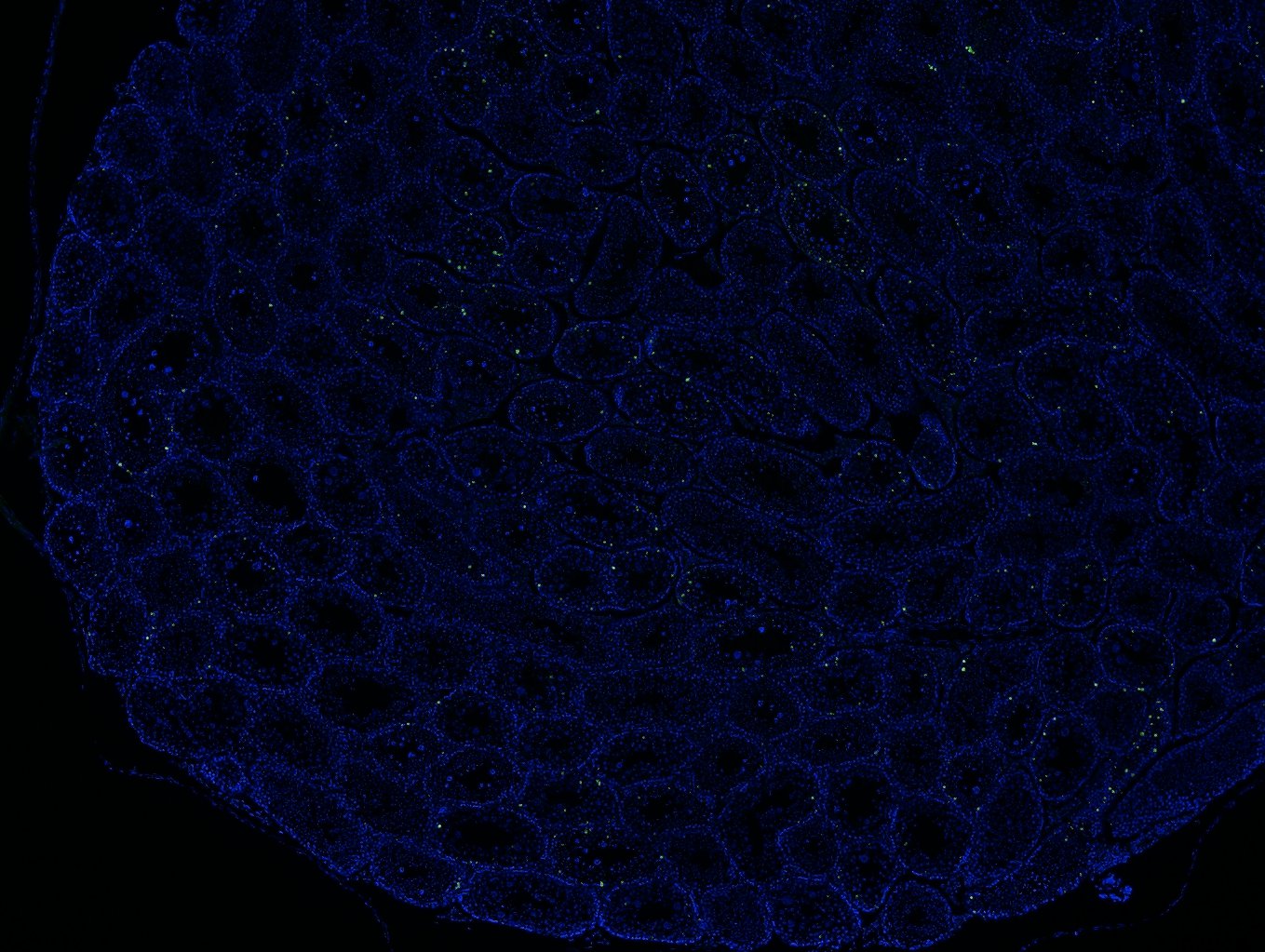

Supplement: Figure 2—source data 1. [file elife-83129-fig2-data1.zip › Figure2/Source data of Figure2C-2D/Count-KO-Testis-Merge-4X.jpg]

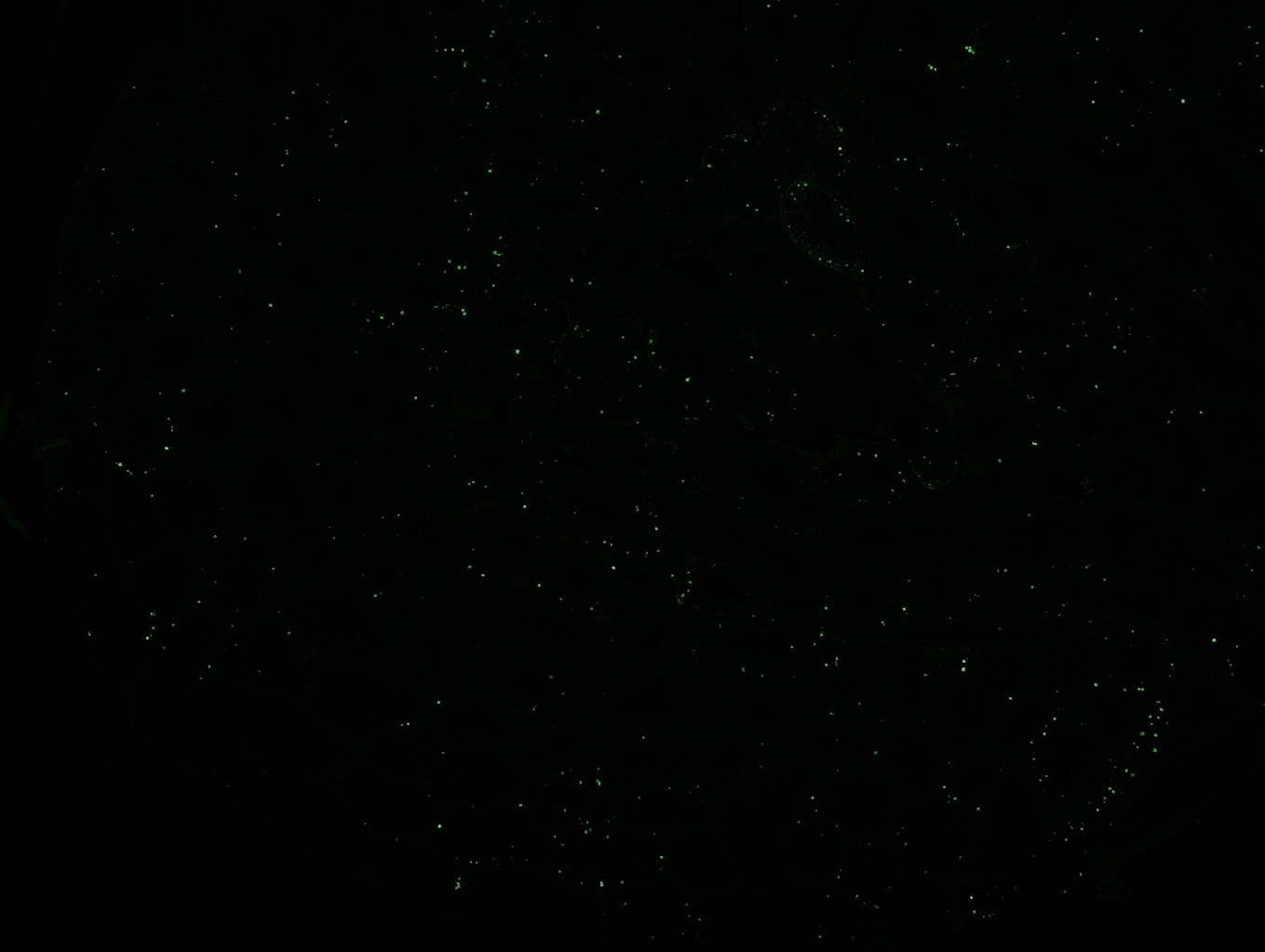

Supplement: Figure 2—source data 1. [file elife-83129-fig2-data1.zip › Figure2/Source data of Figure2C-2D/Count-KO-Testis-TUNEL-4X.tif]

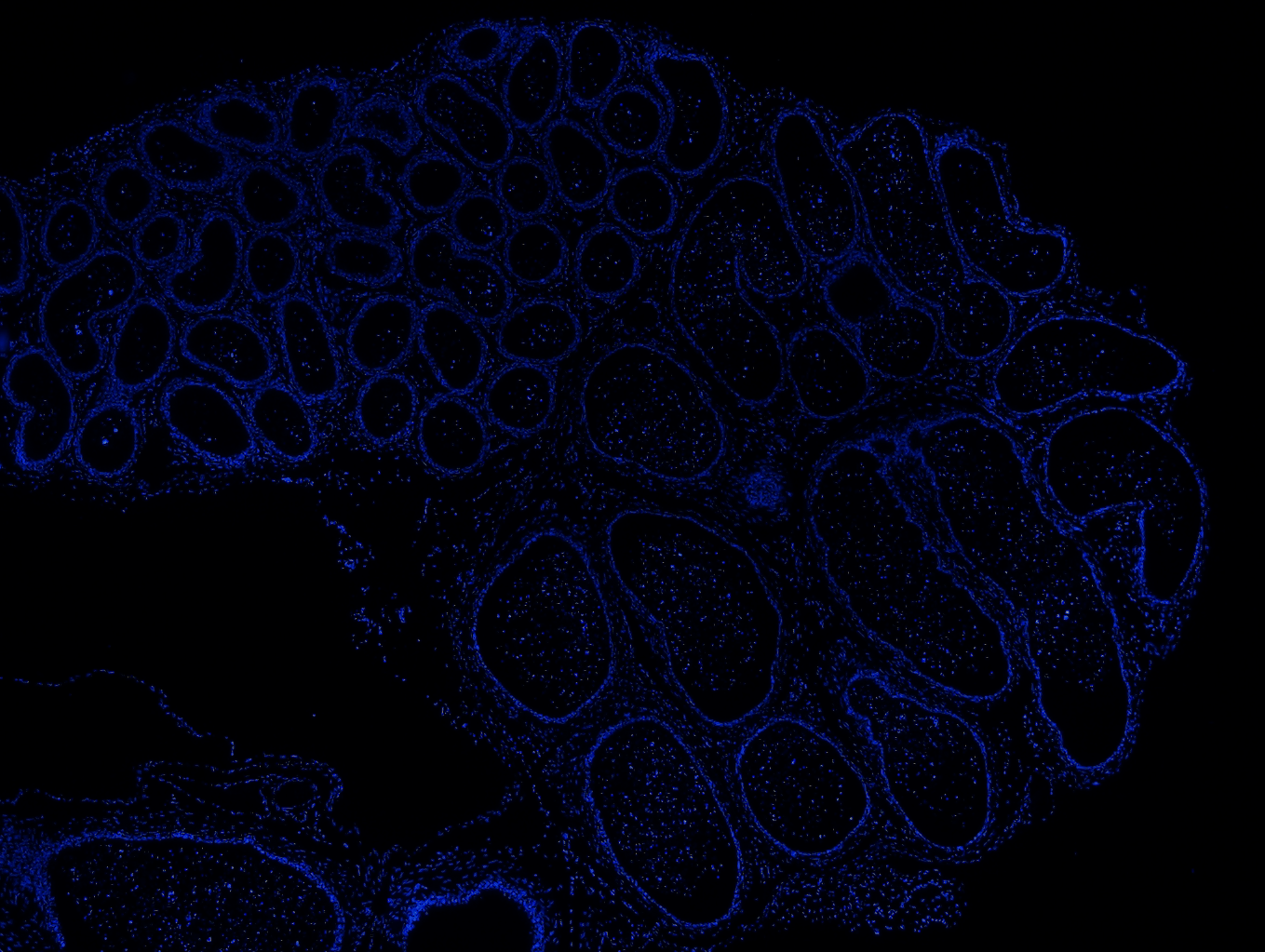

Supplement: Figure 2—source data 1. [file elife-83129-fig2-data1.zip › Figure2/Source data of Figure2C-2D/Count-KO-epi-DAPI-4X.tif]

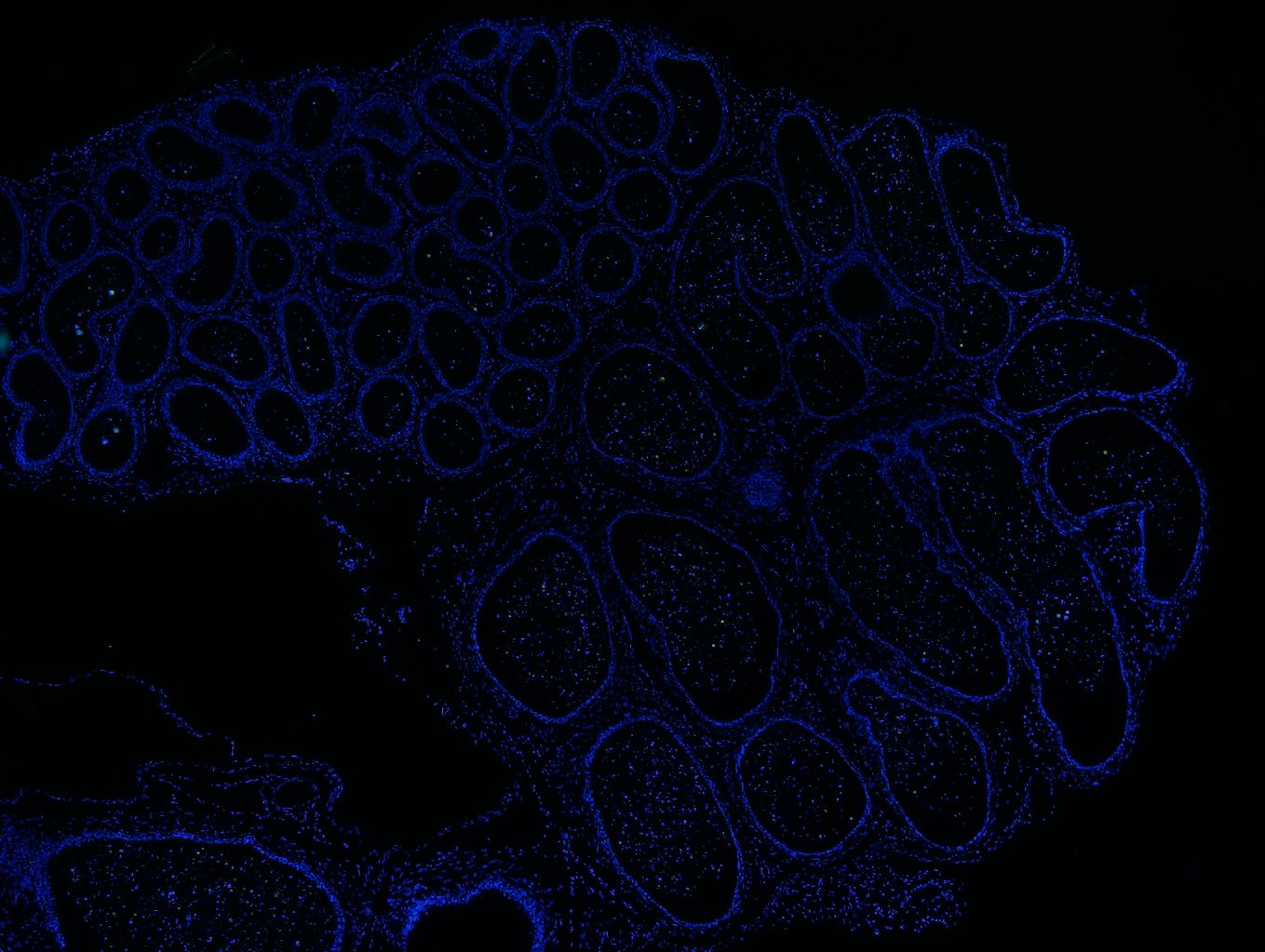

Supplement: Figure 2—source data 1. [file elife-83129-fig2-data1.zip › Figure2/Source data of Figure2C-2D/Count-KO-epi-Merge-4X.jpg]

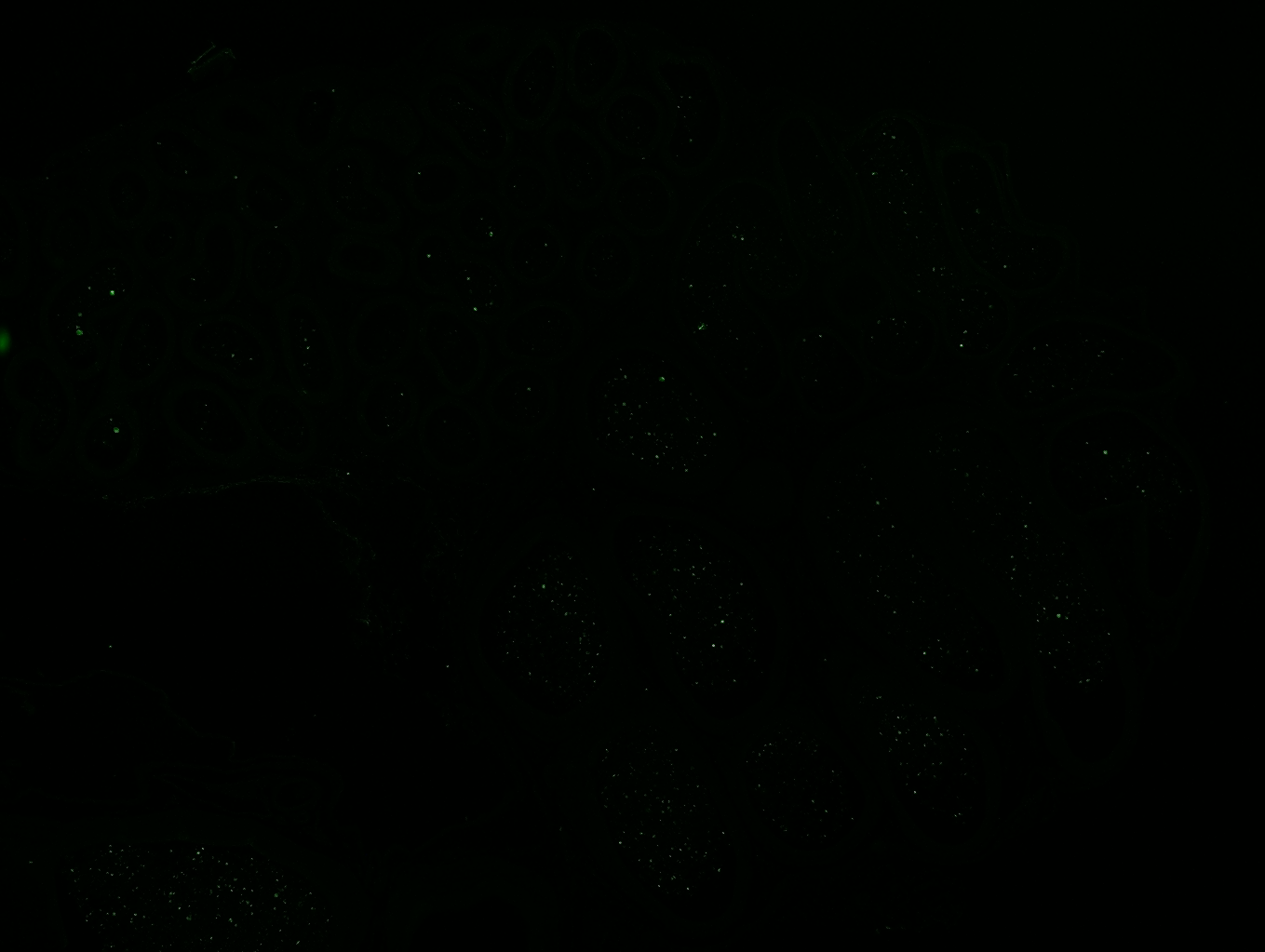

Supplement: Figure 2—source data 1. [file elife-83129-fig2-data1.zip › Figure2/Source data of Figure2C-2D/Count-KO-epi-TUNEL-4X.tif]

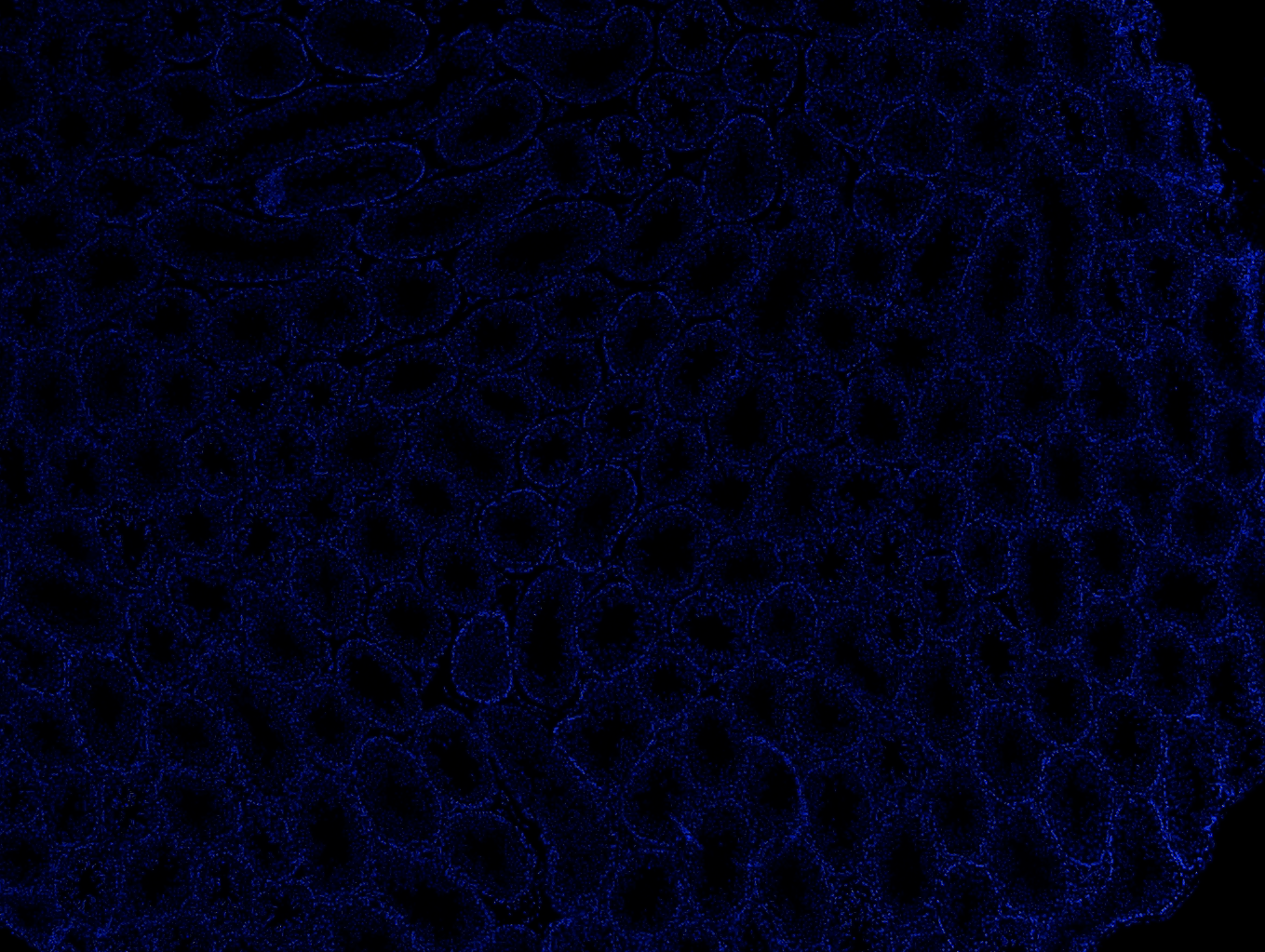

Supplement: Figure 2—source data 1. [file elife-83129-fig2-data1.zip › Figure2/Source data of Figure2C-2D/Count-WT-Testis-DAPI-4X.tif]

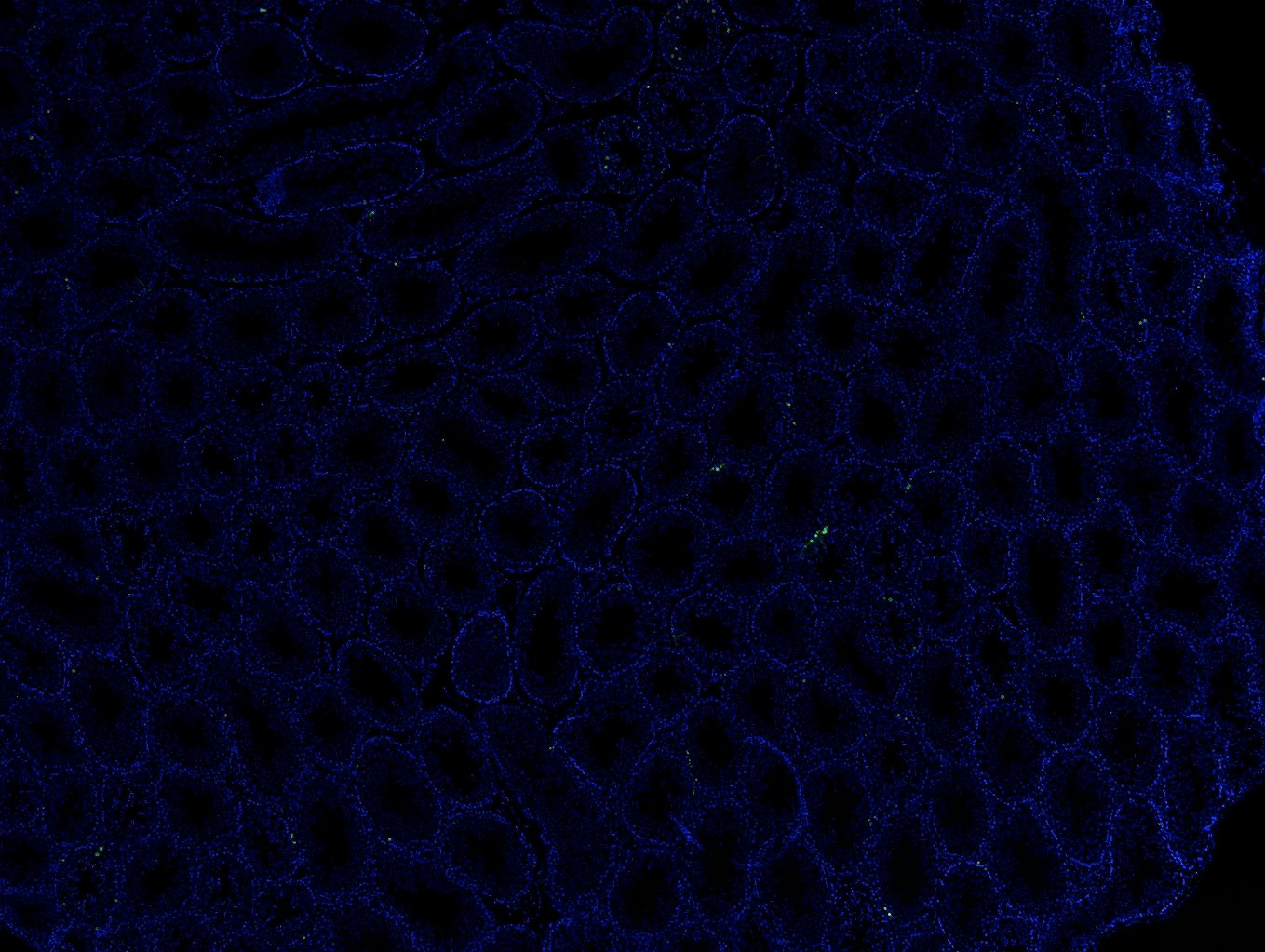

Supplement: Figure 2—source data 1. [file elife-83129-fig2-data1.zip › Figure2/Source data of Figure2C-2D/Count-WT-Testis-Merge-4X.jpg]

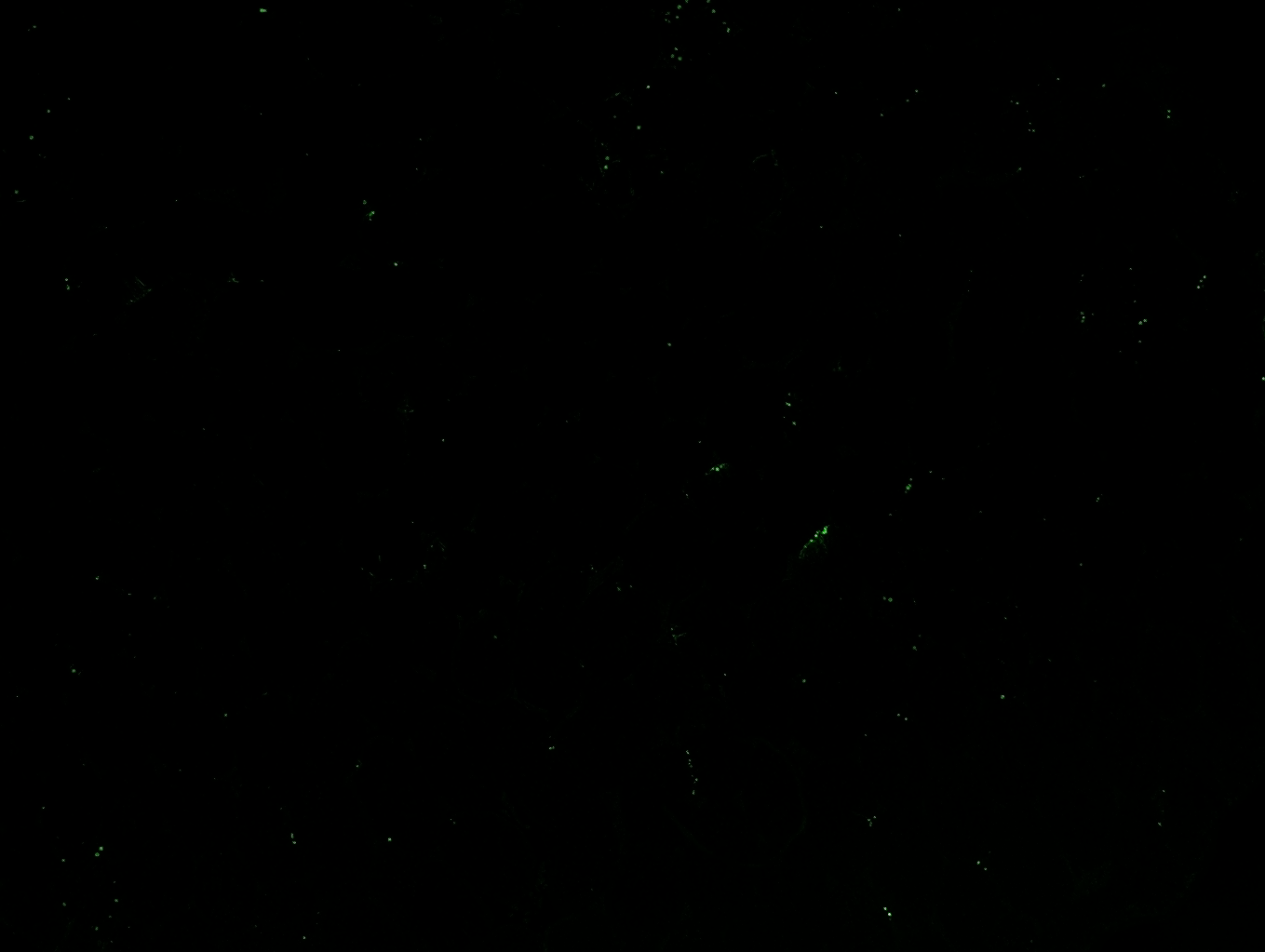

Supplement: Figure 2—source data 1. [file elife-83129-fig2-data1.zip › Figure2/Source data of Figure2C-2D/Count-WT-Testis-TUNEL-4X.tif]

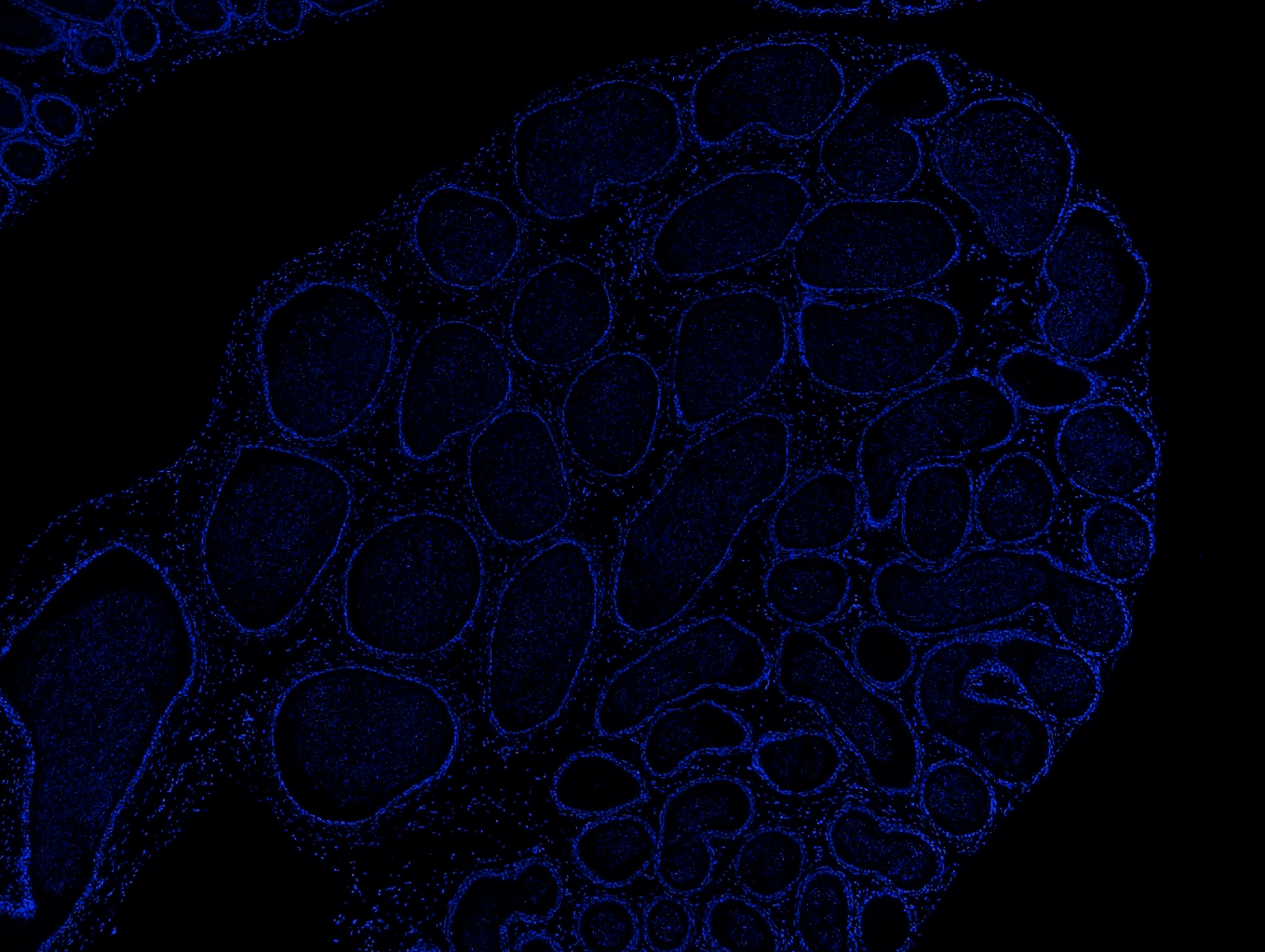

Supplement: Figure 2—source data 1. [file elife-83129-fig2-data1.zip › Figure2/Source data of Figure2C-2D/Count-WT-epi-DAPI-4X.tif]

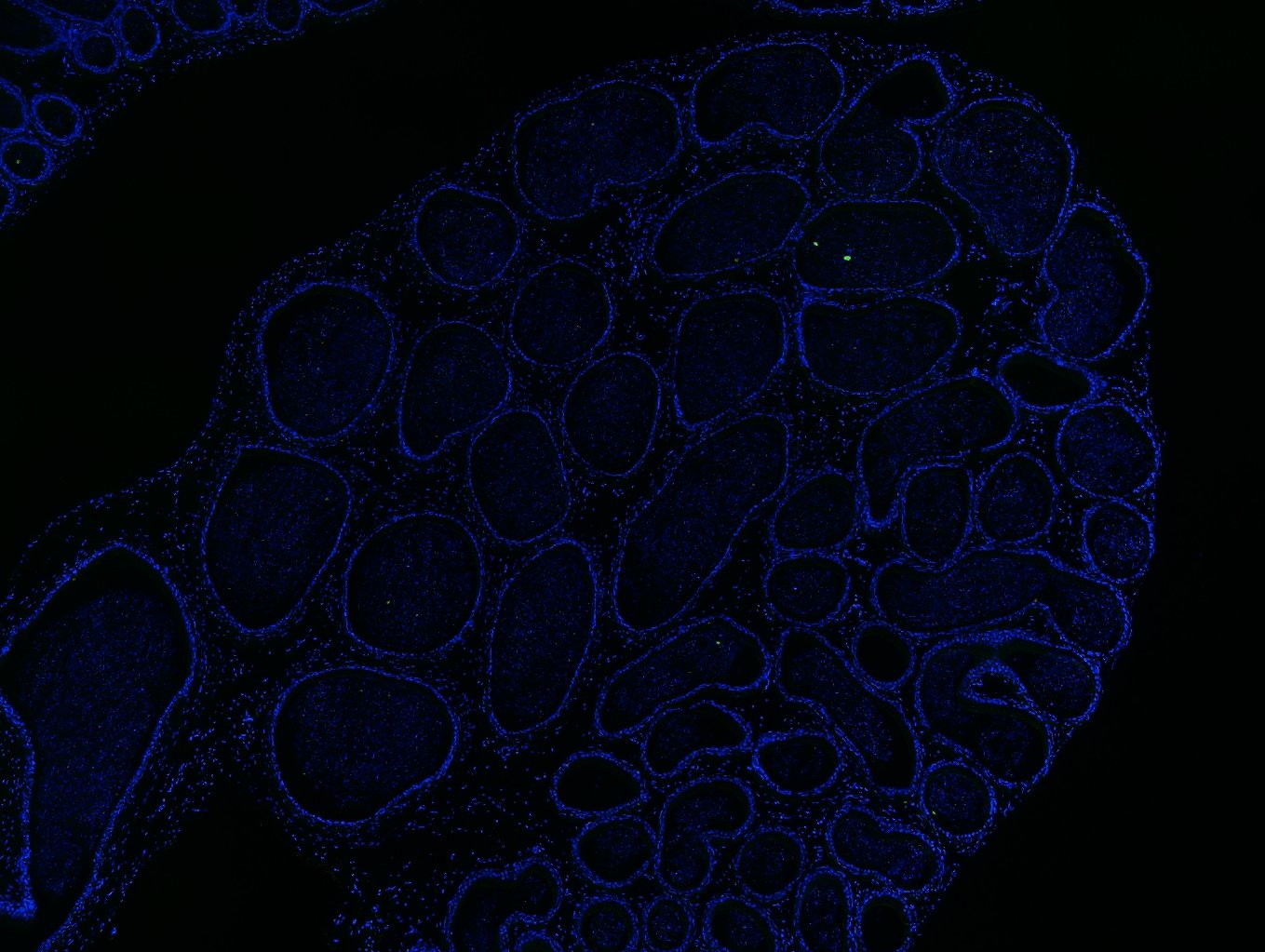

Supplement: Figure 2—source data 1. [file elife-83129-fig2-data1.zip › Figure2/Source data of Figure2C-2D/Count-WT-epi-Merge-4X.jpg]

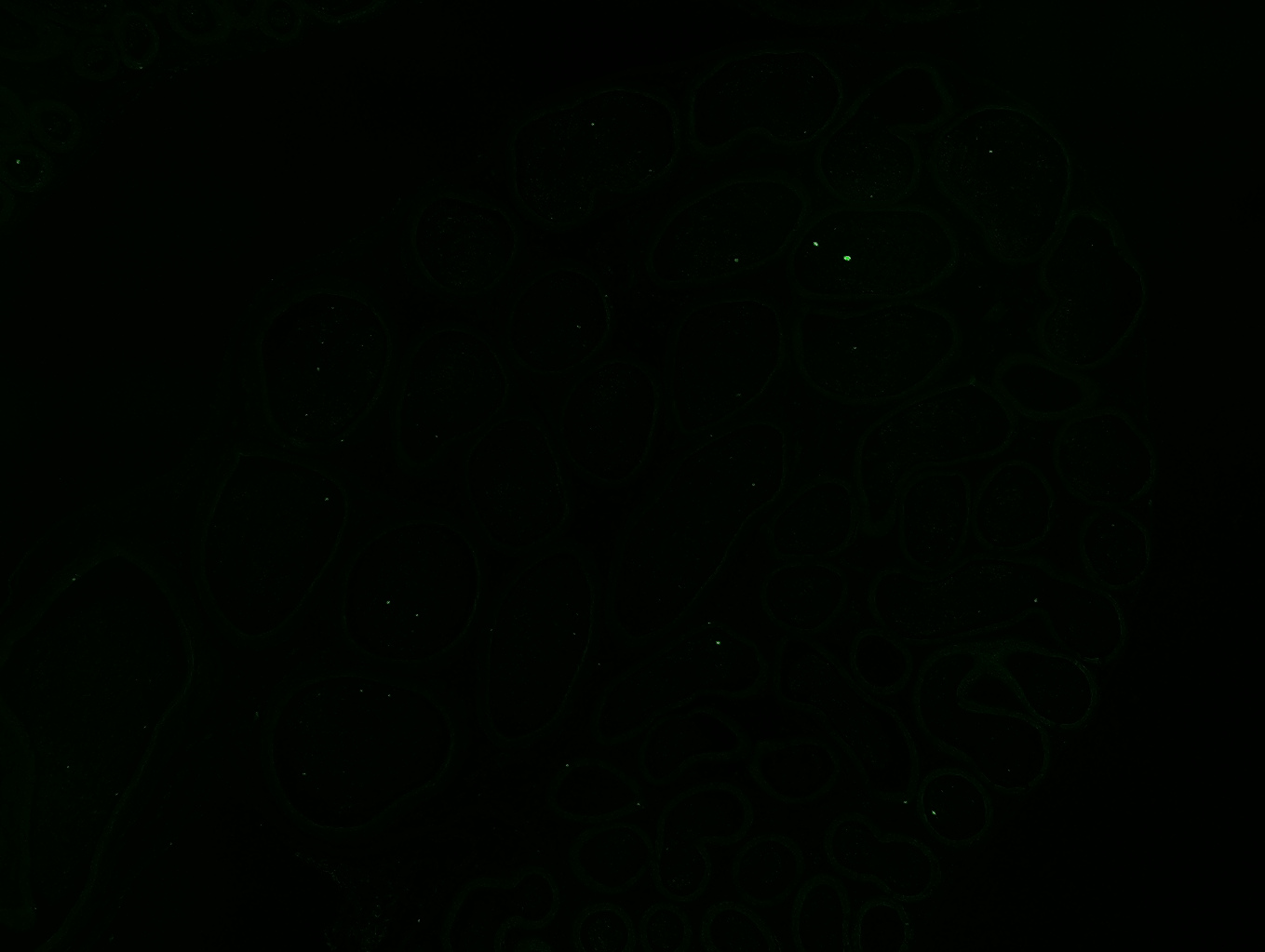

Supplement: Figure 2—source data 1. [file elife-83129-fig2-data1.zip › Figure2/Source data of Figure2C-2D/Count-WT-epi-TUNEL-4X.tif]

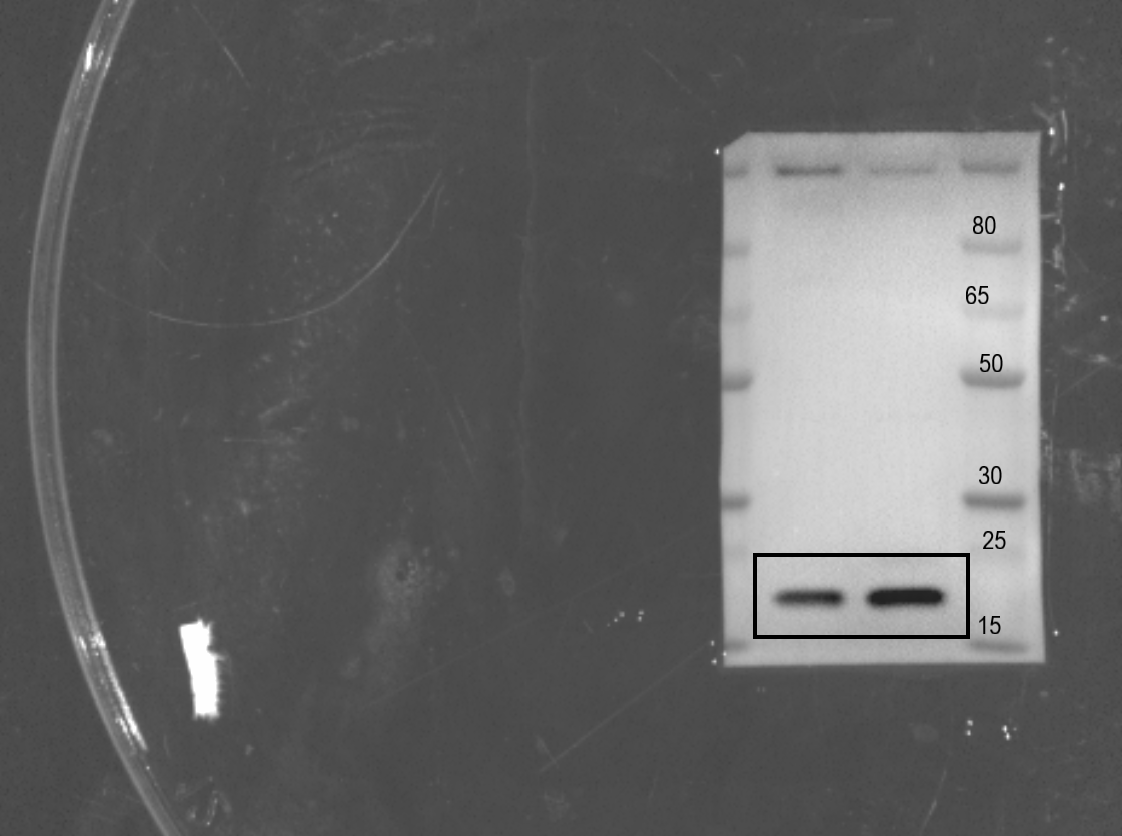

Supplement: Figure 2—source data 1. [file elife-83129-fig2-data1.zip › Figure2/Source data of Figure2E/Labbelled blots of Figure2E-BAX.tiff]

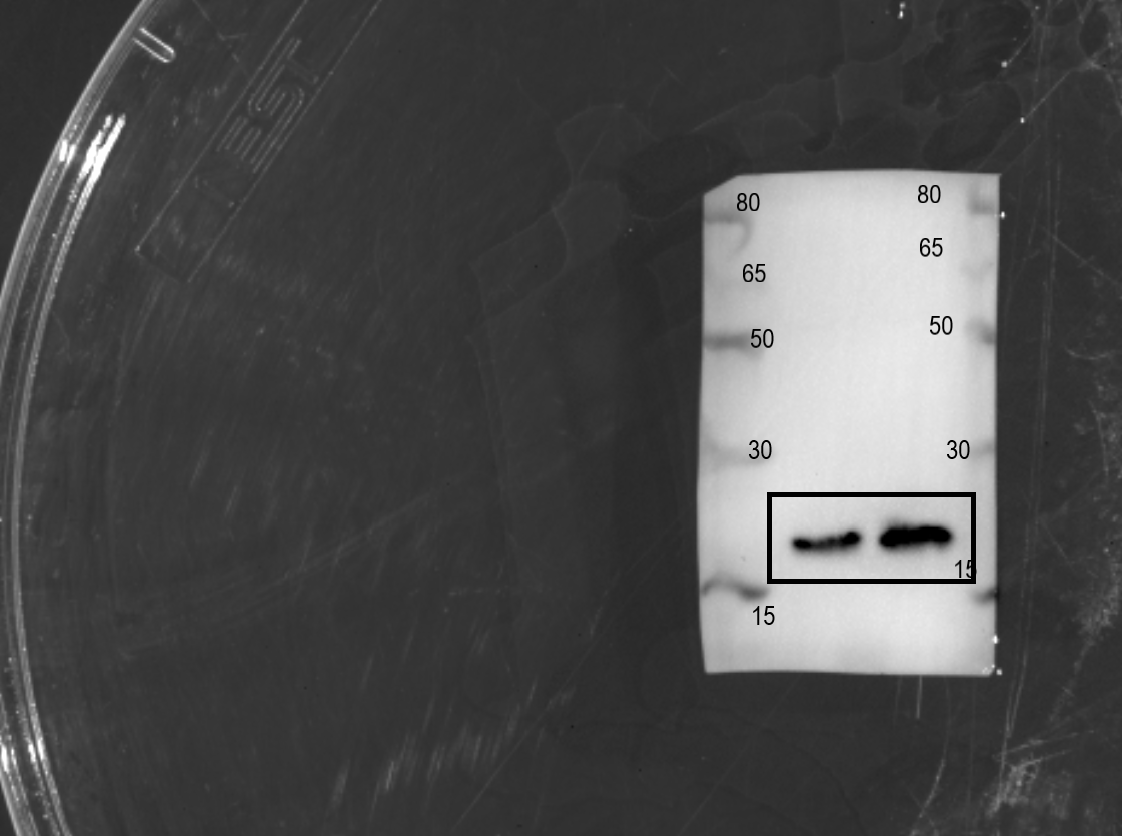

Supplement: Figure 2—source data 1. [file elife-83129-fig2-data1.zip › Figure2/Source data of Figure2E/Labelled blots of Figure2E-BAD.tiff]

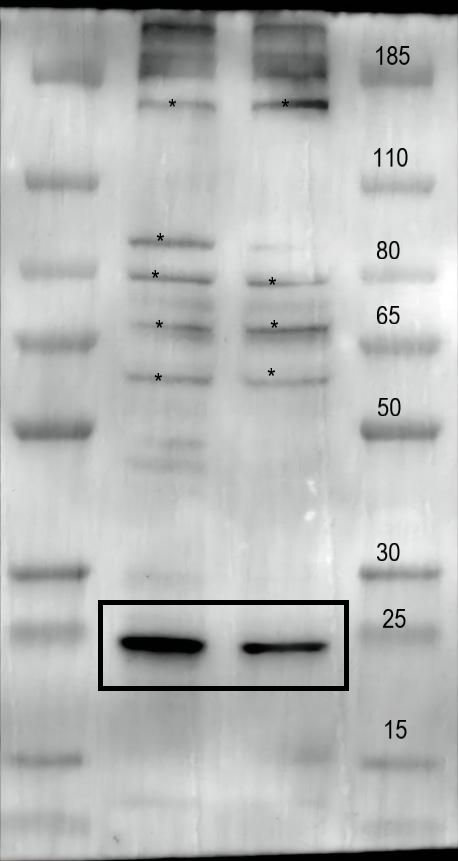

Supplement: Figure 2—source data 1. [file elife-83129-fig2-data1.zip › Figure2/Source data of Figure2E/Labelled blots of Figure2E-BCL2.tiff]

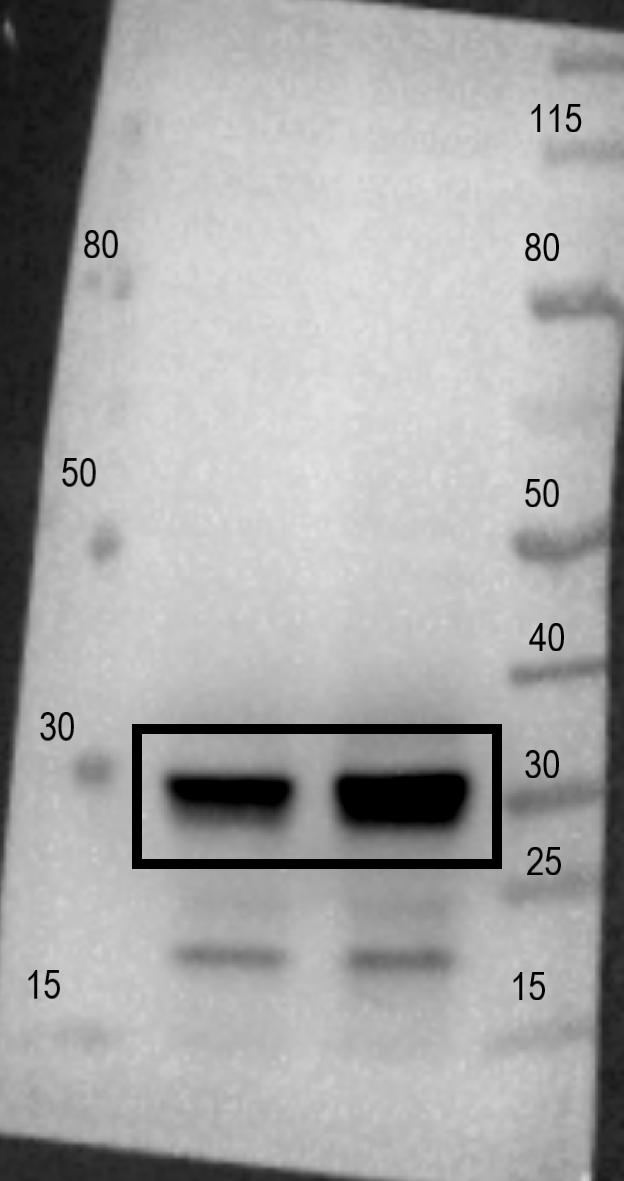

Supplement: Figure 2—source data 1. [file elife-83129-fig2-data1.zip › Figure2/Source data of Figure2E/Labelled blots of Figure2E-Caspase-3.tif]

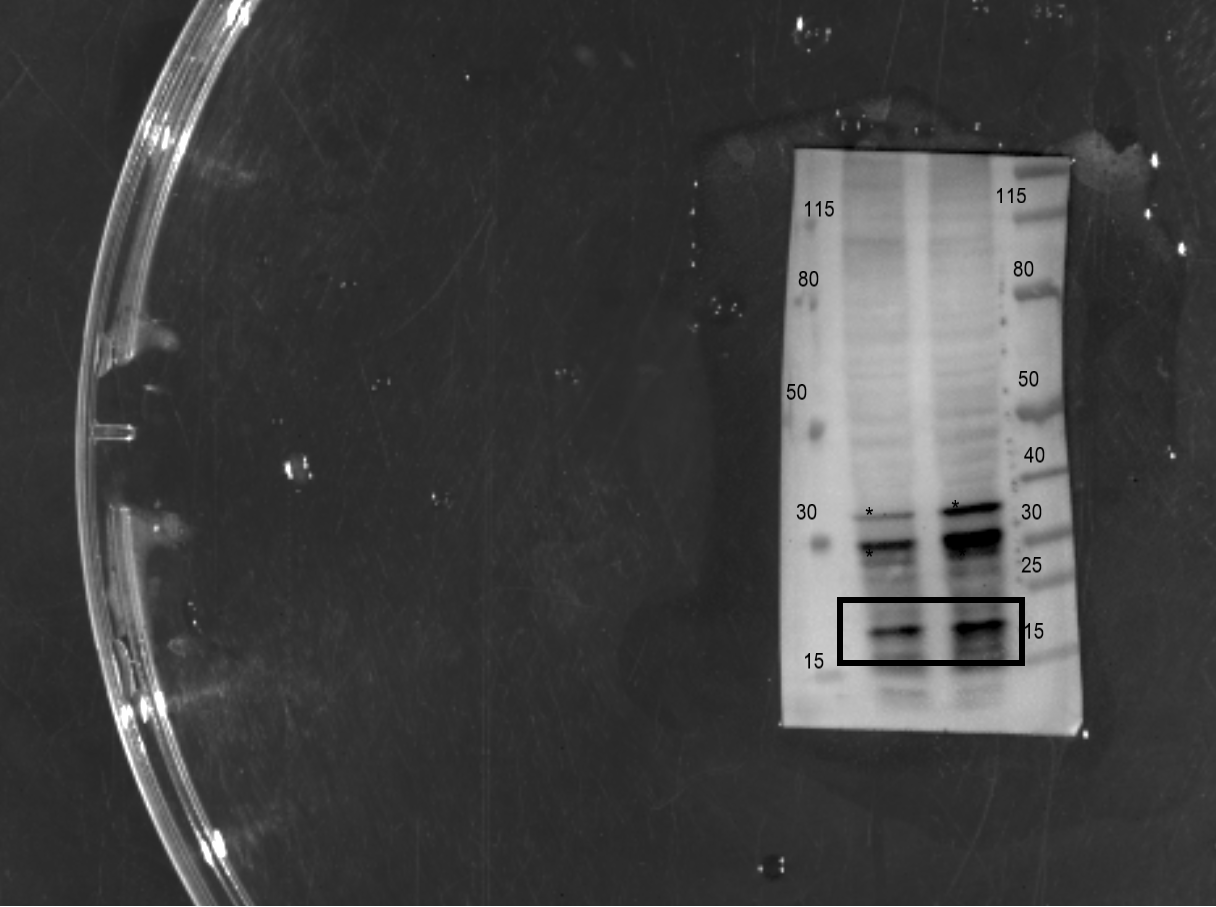

Supplement: Figure 2—source data 1. [file elife-83129-fig2-data1.zip › Figure2/Source data of Figure2E/Labelled blots of Figure2E-Cleaved-Caspase-3.tif]

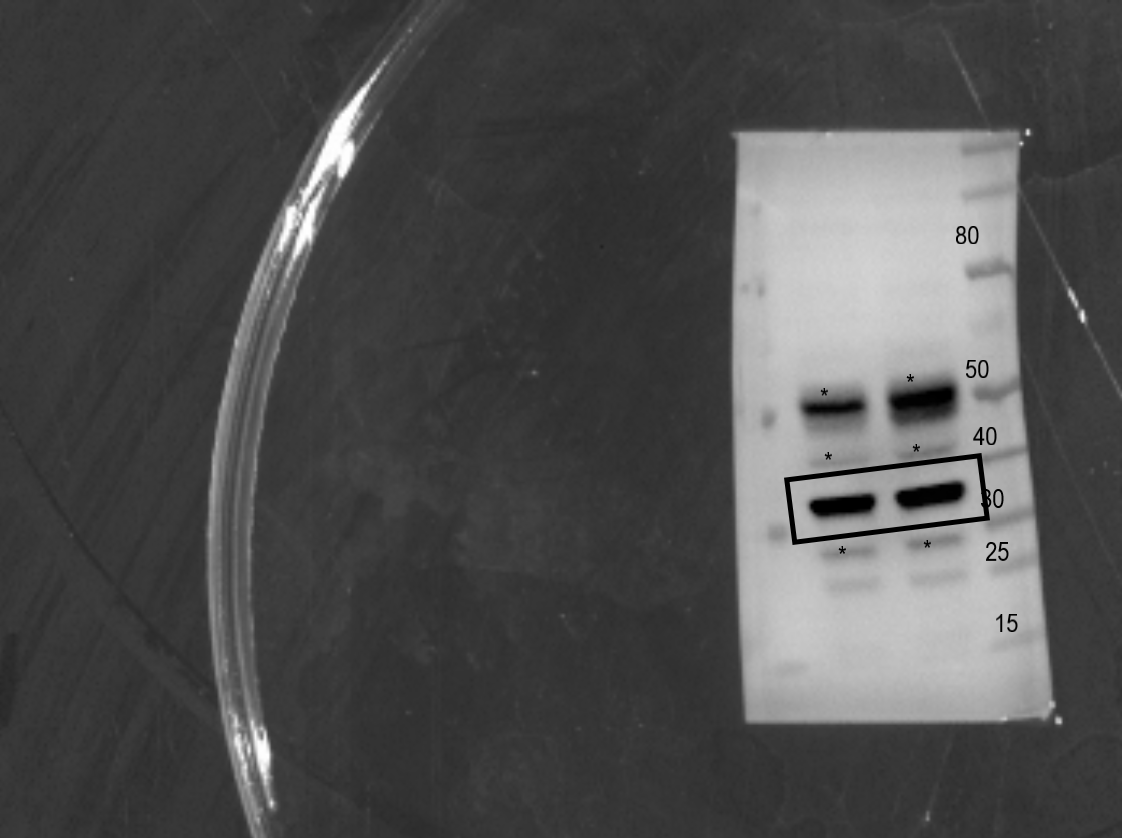

Supplement: Figure 2—source data 1. [file elife-83129-fig2-data1.zip › Figure2/Source data of Figure2E/Labelled blots of Figure2E-GAPDH.tif]

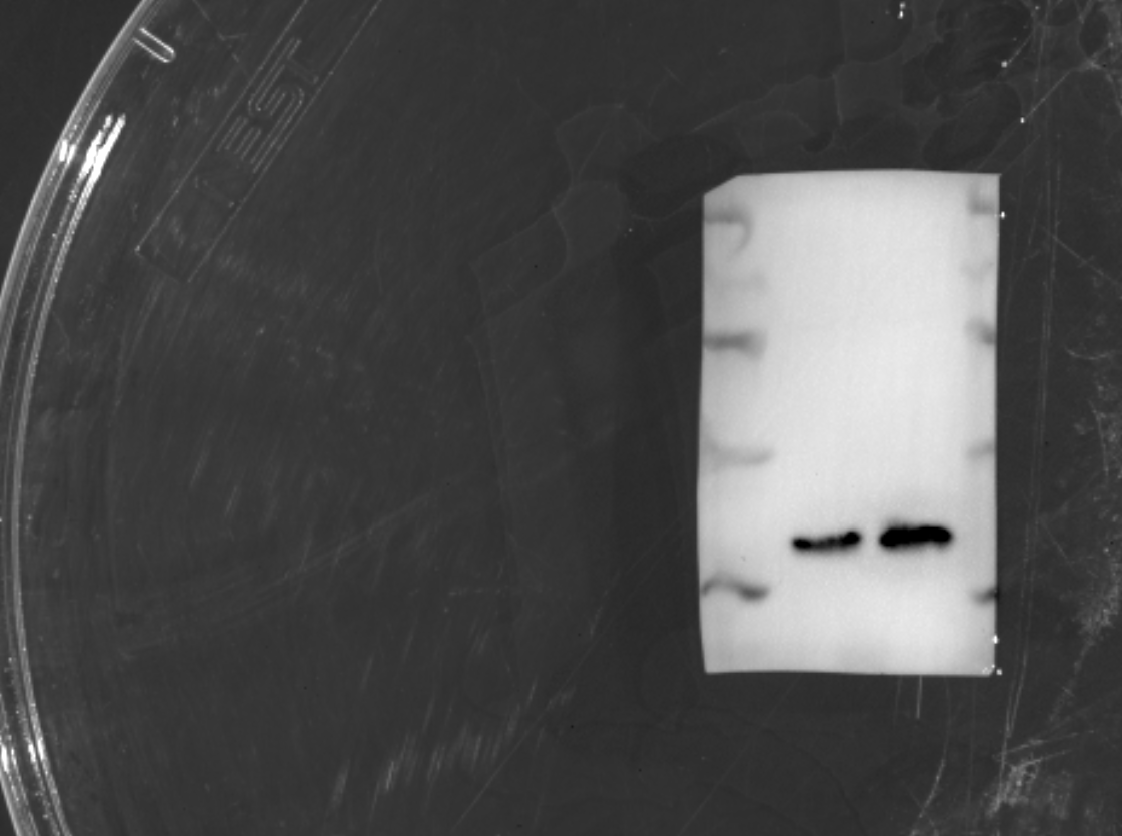

Supplement: Figure 2—source data 1. [file elife-83129-fig2-data1.zip › Figure2/Source data of Figure2E/Raw blots of Figure2E-BAD.tif]

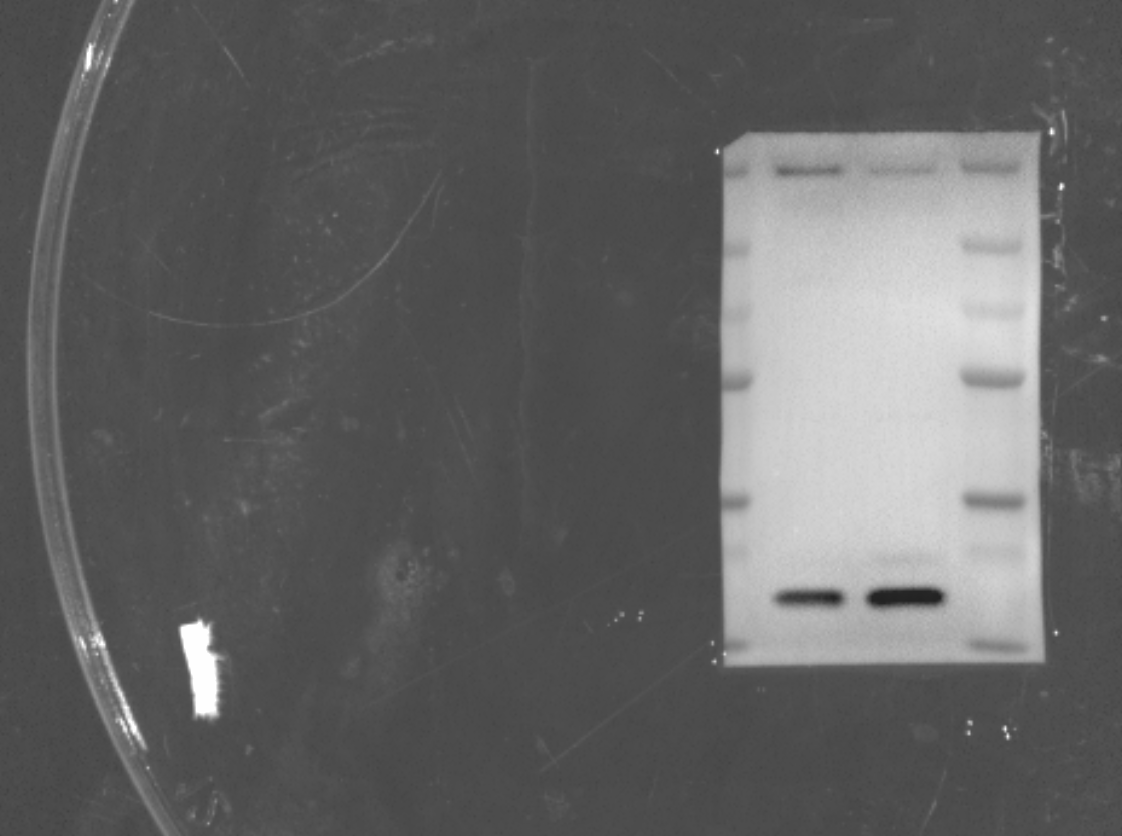

Supplement: Figure 2—source data 1. [file elife-83129-fig2-data1.zip › Figure2/Source data of Figure2E/Raw blots of Figure2E-BAX.tif]

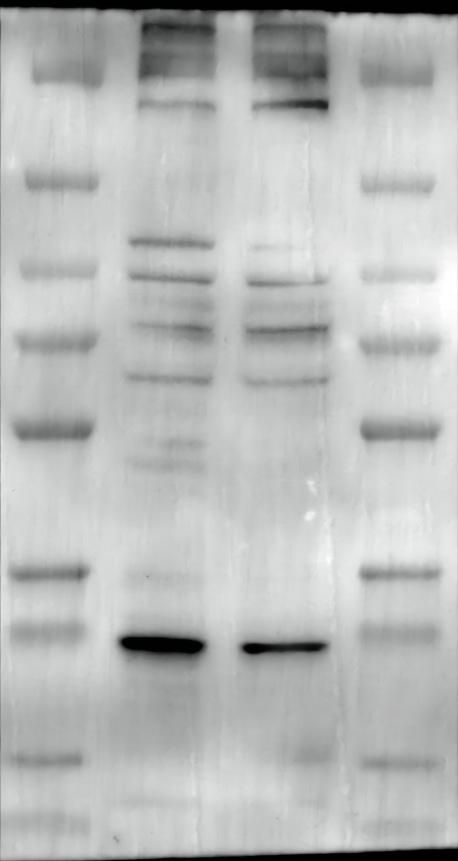

Supplement: Figure 2—source data 1. [file elife-83129-fig2-data1.zip › Figure2/Source data of Figure2E/Raw blots of Figure2E-BCL2.jpg]

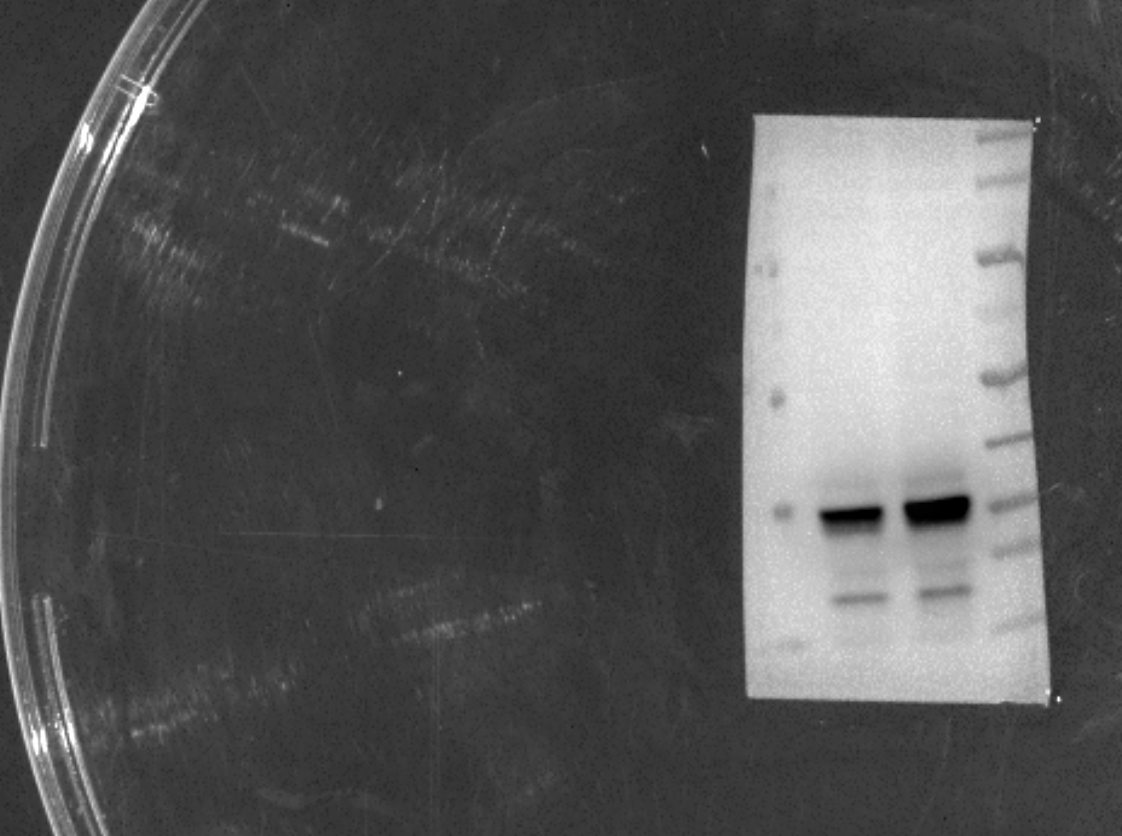

Supplement: Figure 2—source data 1. [file elife-83129-fig2-data1.zip › Figure2/Source data of Figure2E/Raw blots of Figure2E-Caspase-3.tif]

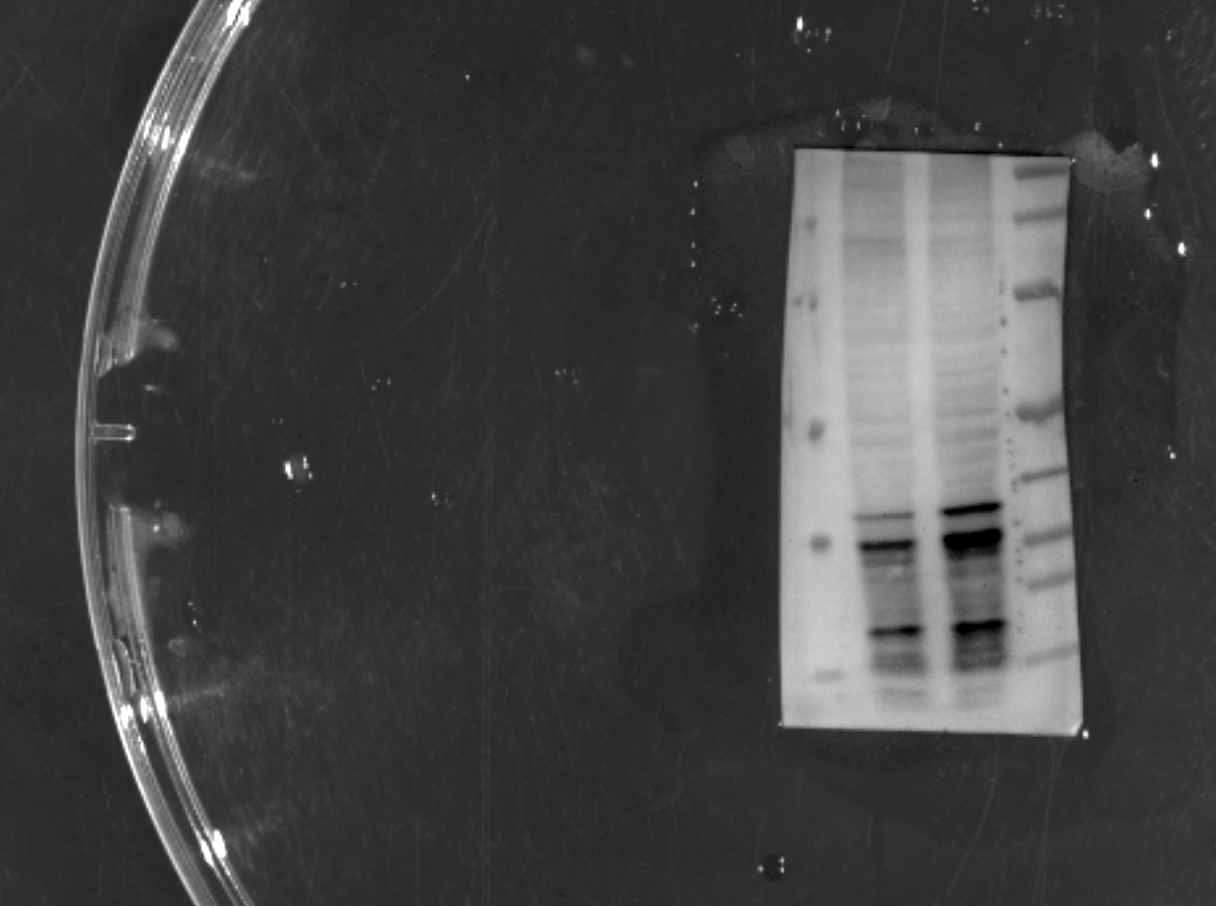

Supplement: Figure 2—source data 1. [file elife-83129-fig2-data1.zip › Figure2/Source data of Figure2E/Raw blots of Figure2E-Cleaved-Caspase-3.tif]

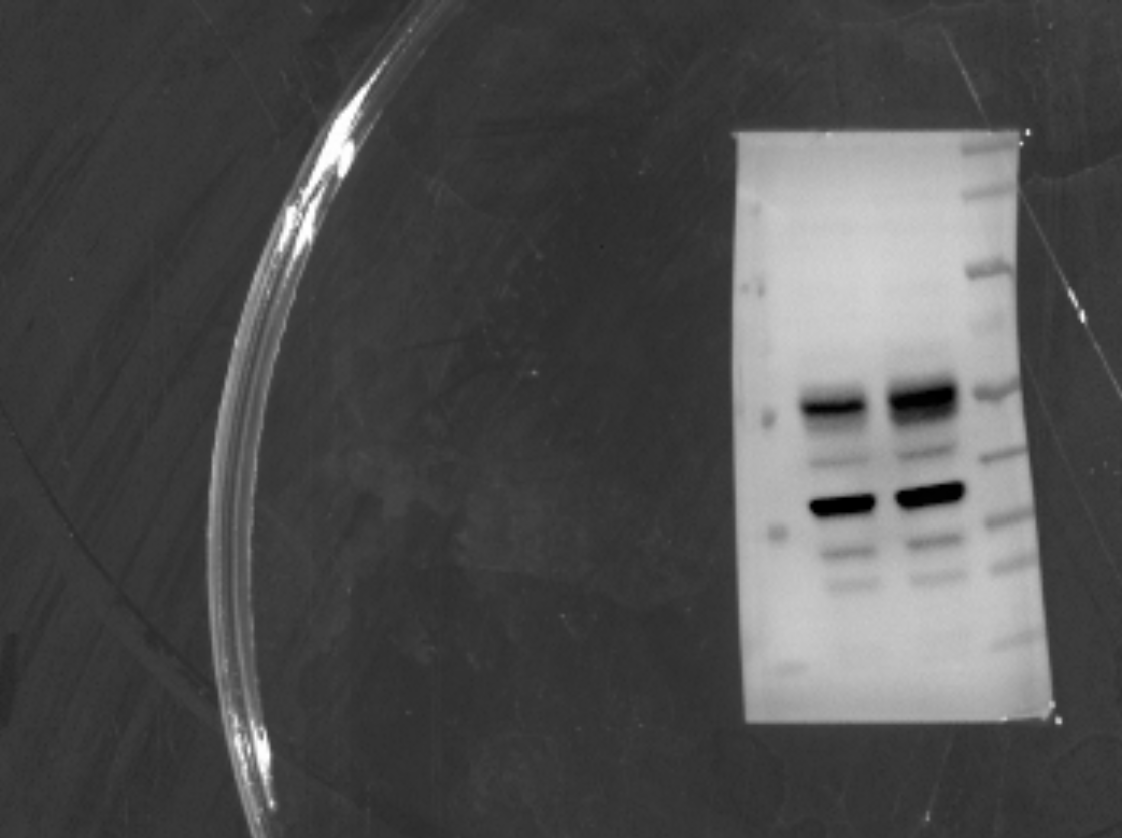

Supplement: Figure 2—source data 1. [file elife-83129-fig2-data1.zip › Figure2/Source data of Figure2E/Raw blots of Figure2E-GAPDH.tif]

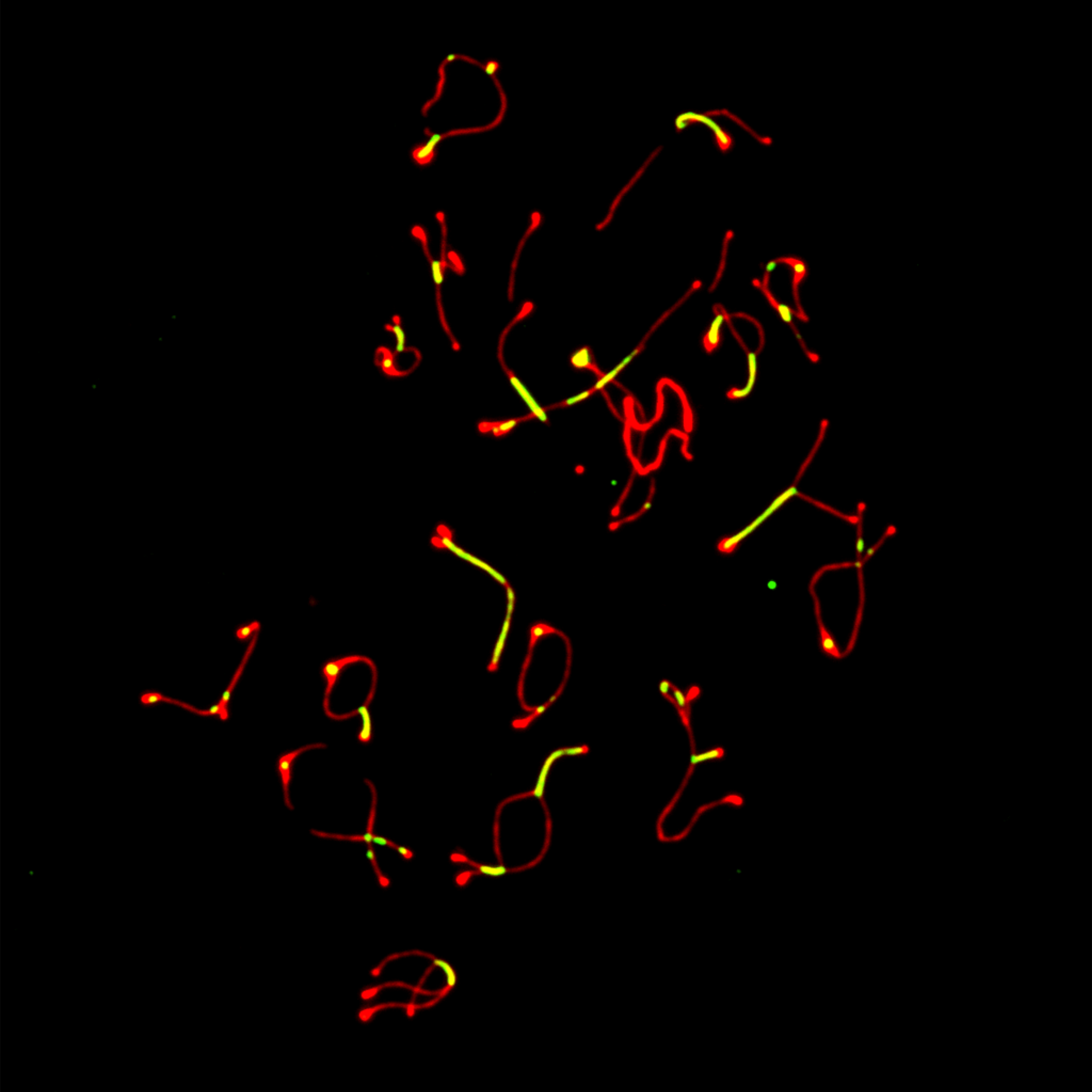

Supplement: Figure 2—figure supplement 1—source data 1. [file elife-83129-fig2-figsupp1-data1.zip › Figure supplement S3-source data 10/Diplotene/KO MERGE.tif]

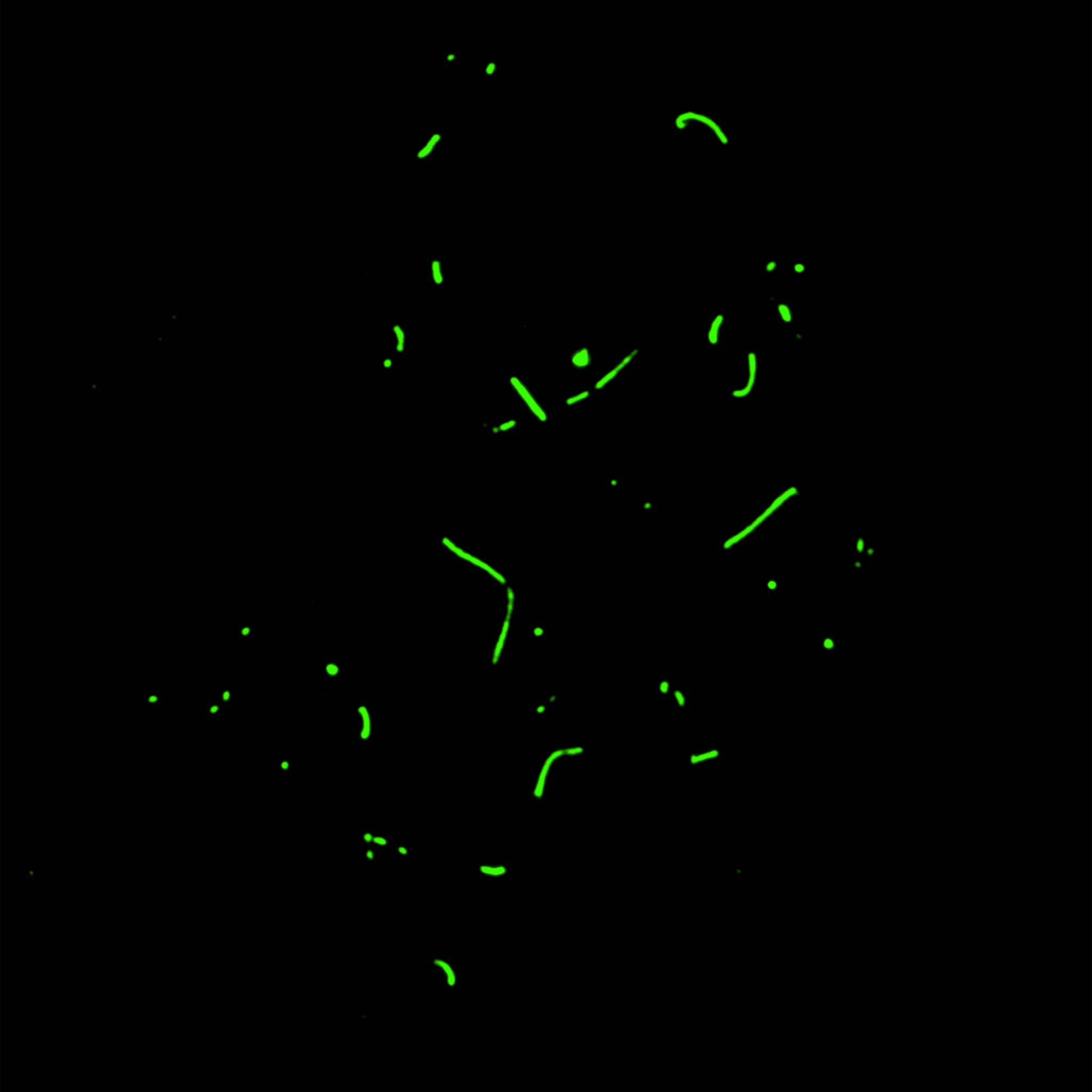

Supplement: Figure 2—figure supplement 1—source data 1. [file elife-83129-fig2-figsupp1-data1.zip › Figure supplement S3-source data 10/Diplotene/KO SYCP1.tif]

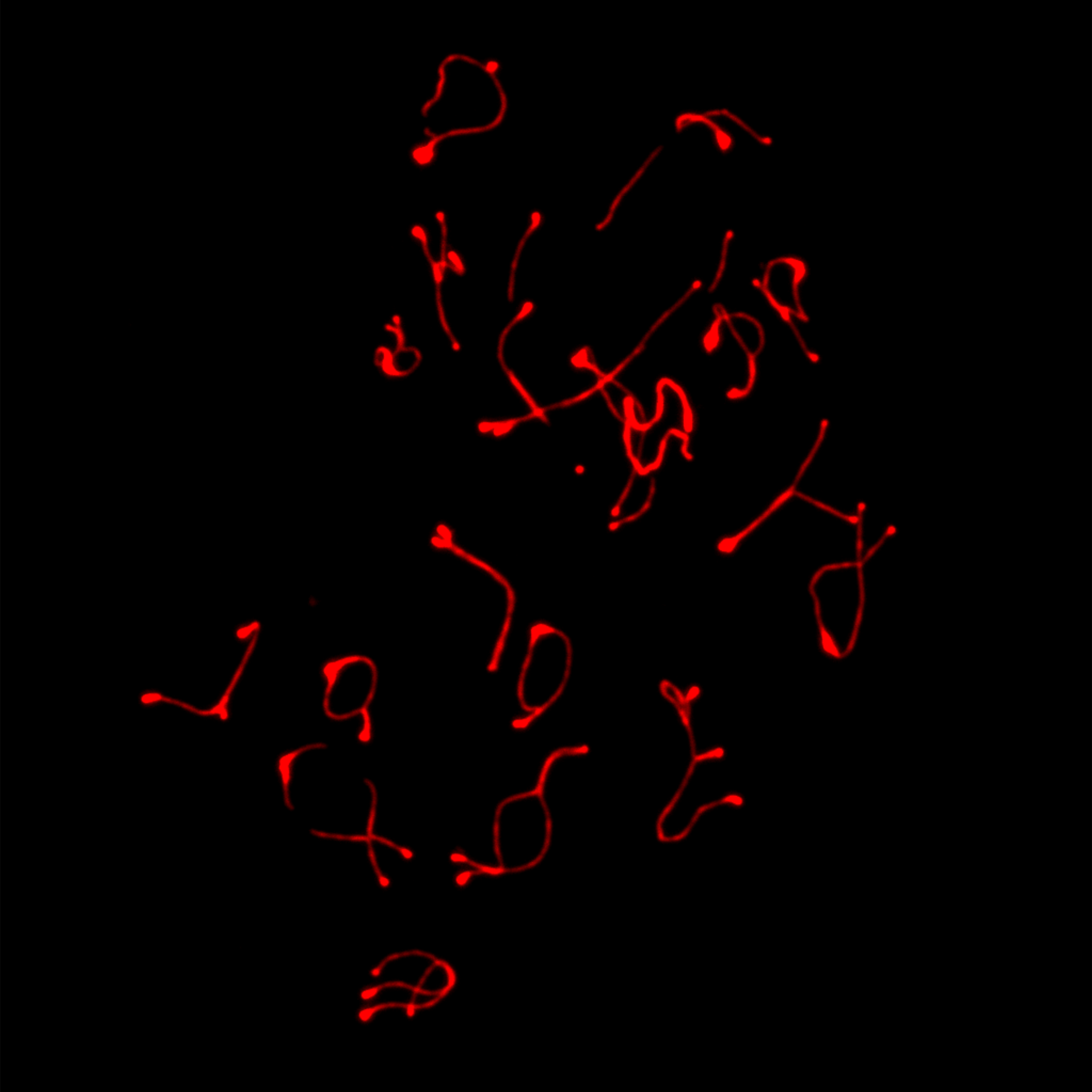

Supplement: Figure 2—figure supplement 1—source data 1. [file elife-83129-fig2-figsupp1-data1.zip › Figure supplement S3-source data 10/Diplotene/KO SYCP3.tif]

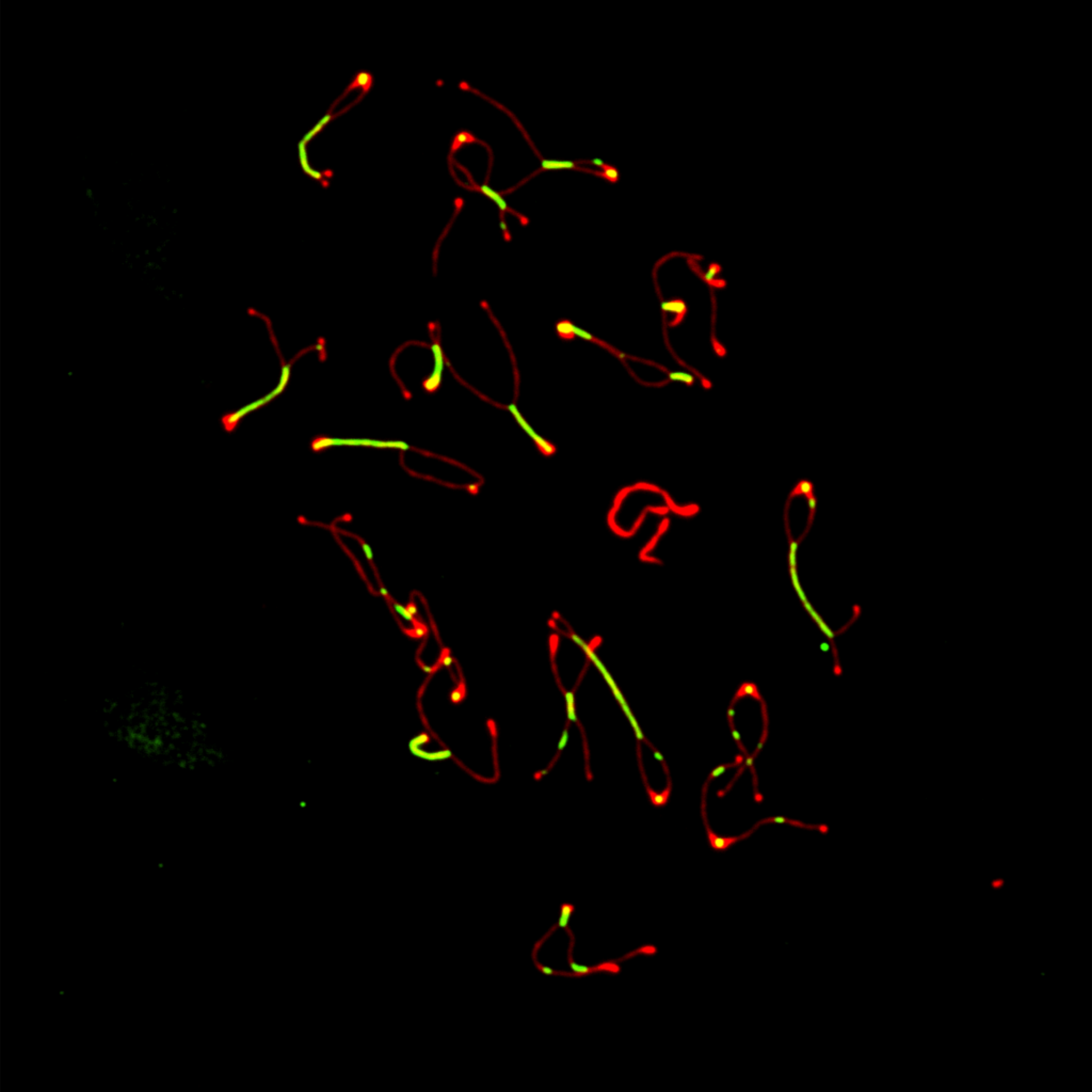

Supplement: Figure 2—figure supplement 1—source data 1. [file elife-83129-fig2-figsupp1-data1.zip › Figure supplement S3-source data 10/Diplotene/WT MERGE.tif]

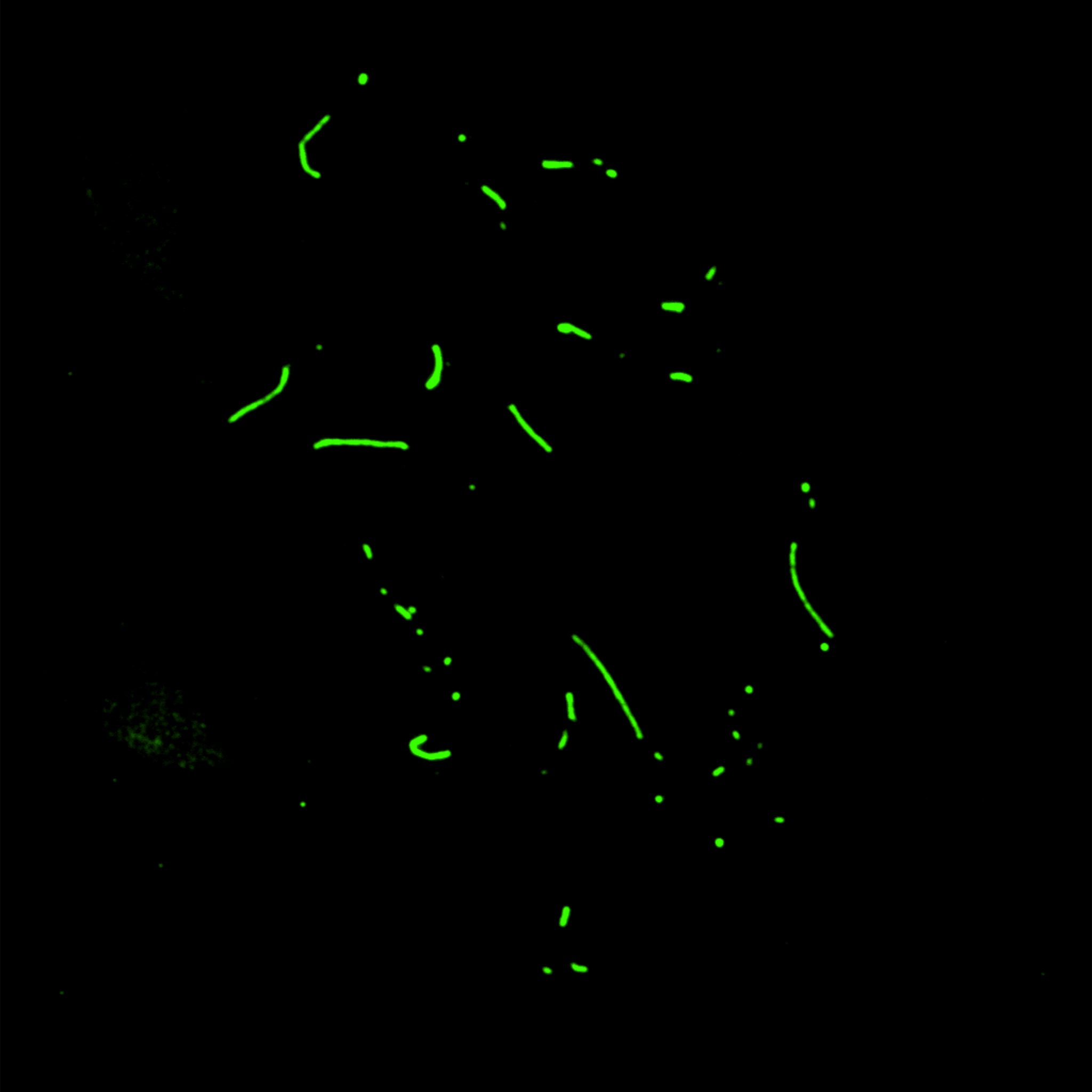

Supplement: Figure 2—figure supplement 1—source data 1. [file elife-83129-fig2-figsupp1-data1.zip › Figure supplement S3-source data 10/Diplotene/WT SYCP1.tif]

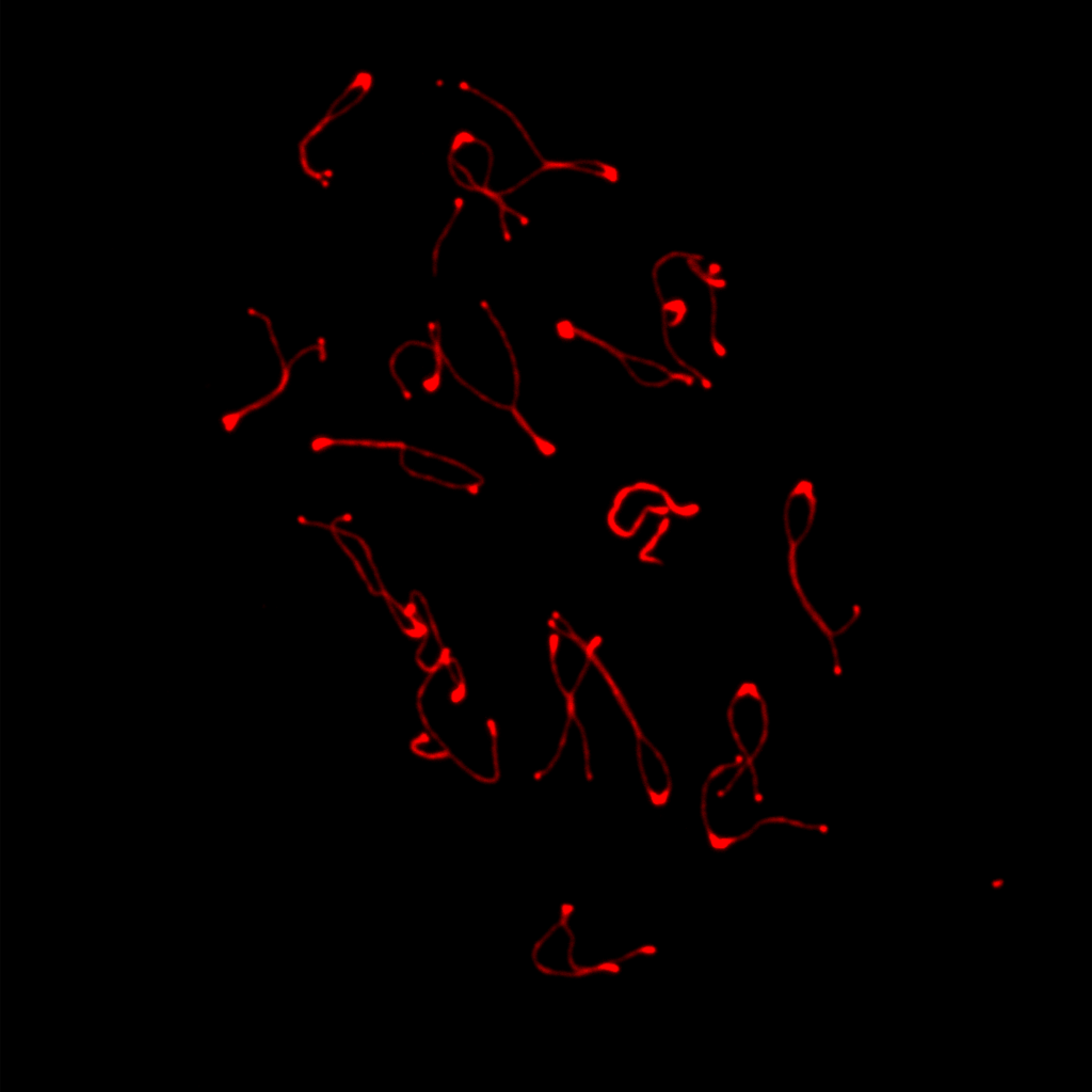

Supplement: Figure 2—figure supplement 1—source data 1. [file elife-83129-fig2-figsupp1-data1.zip › Figure supplement S3-source data 10/Diplotene/WT SYCP3.tif]

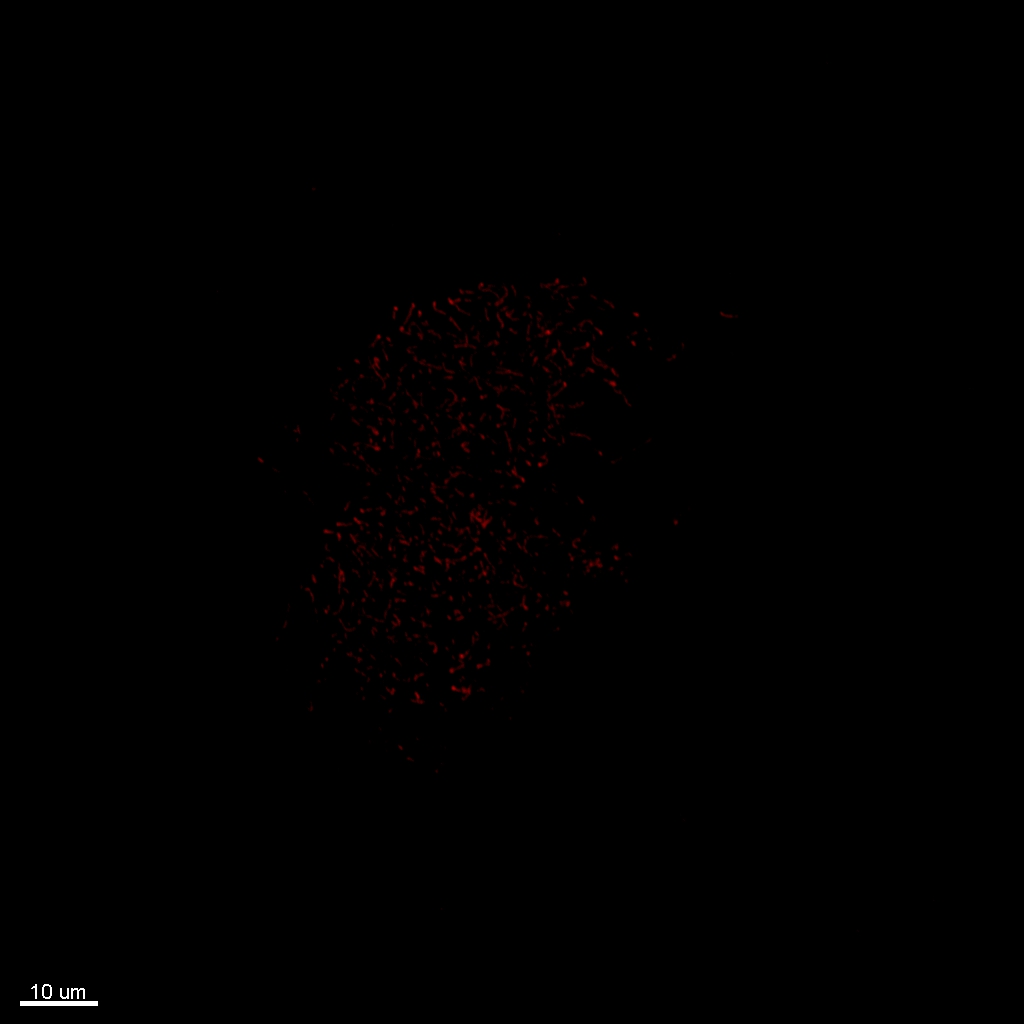

Supplement: Figure 2—figure supplement 1—source data 1. [file elife-83129-fig2-figsupp1-data1.zip › Figure supplement S3-source data 10/Leptotene/KO MERGE.jpg]

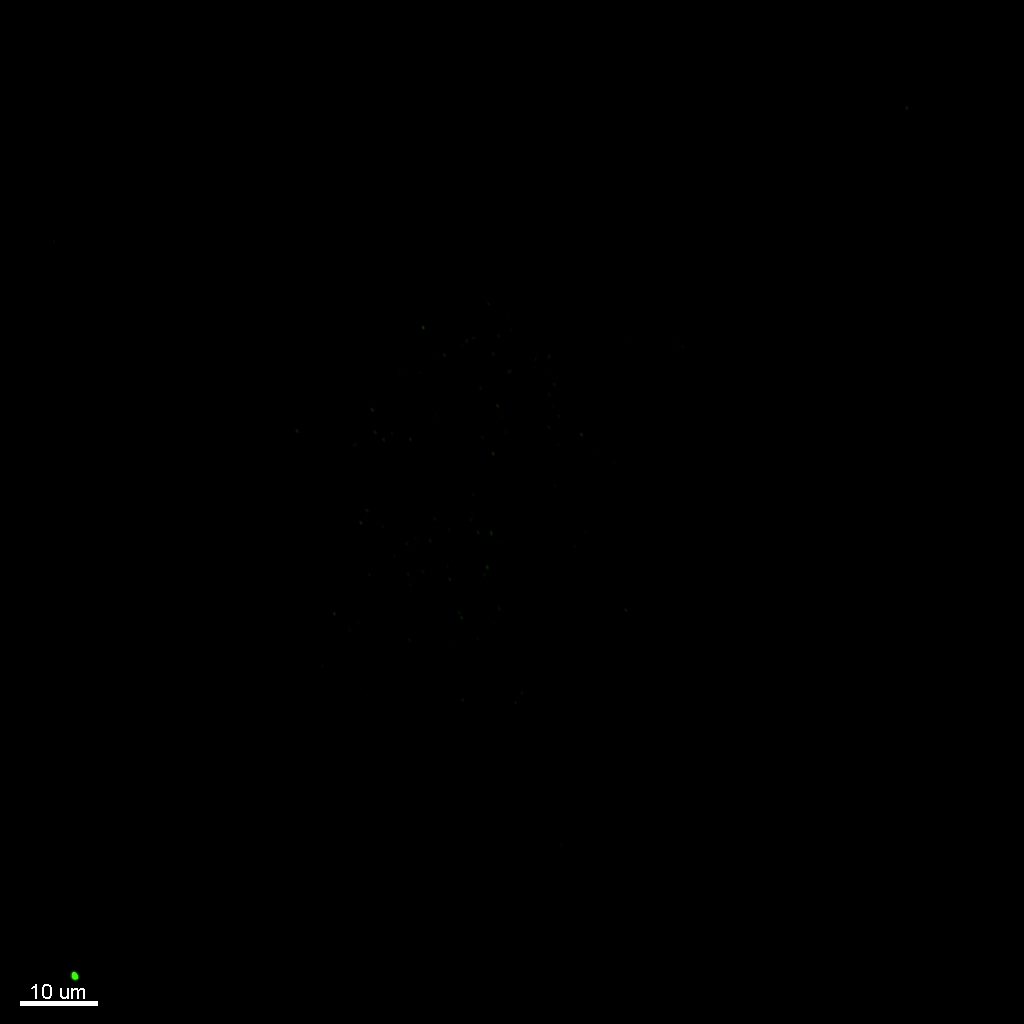

Supplement: Figure 2—figure supplement 1—source data 1. [file elife-83129-fig2-figsupp1-data1.zip › Figure supplement S3-source data 10/Leptotene/KO SYCP1.jpg]

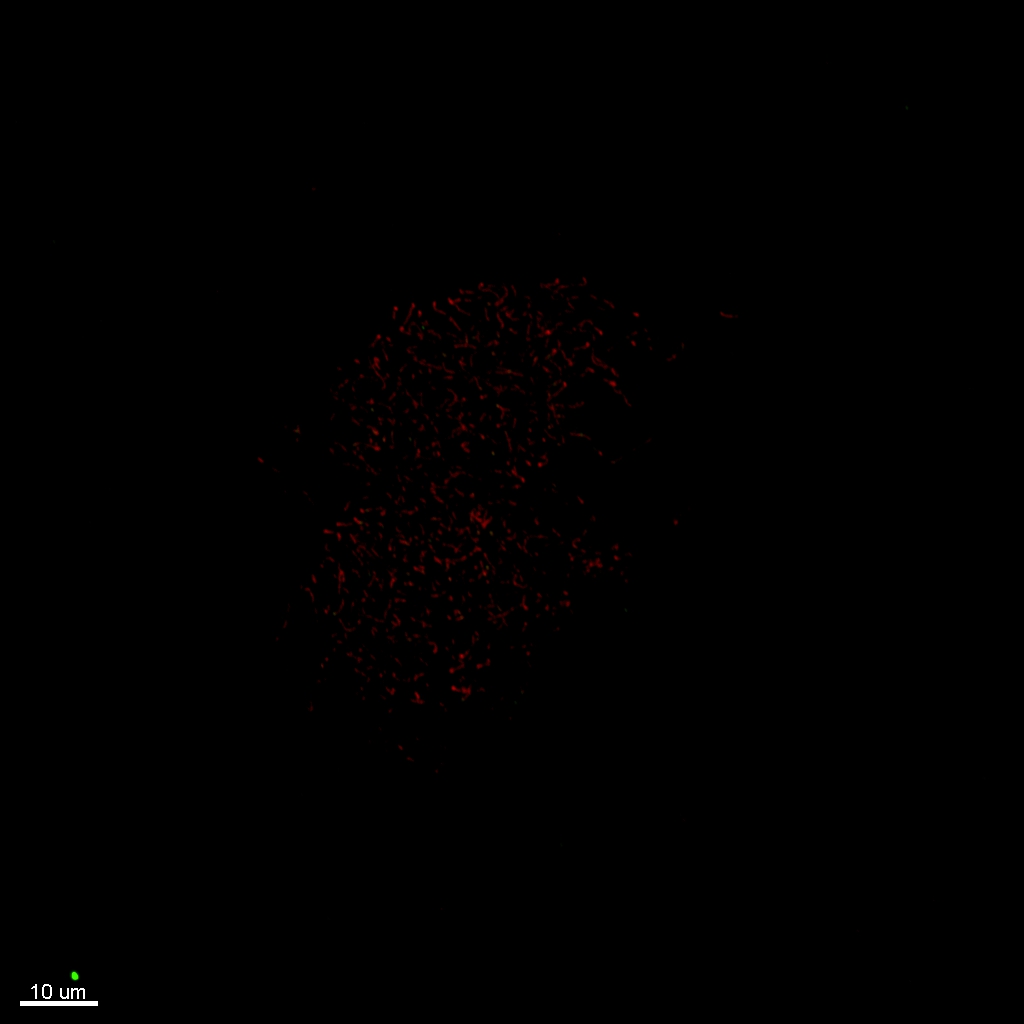

Supplement: Figure 2—figure supplement 1—source data 1. [file elife-83129-fig2-figsupp1-data1.zip › Figure supplement S3-source data 10/Leptotene/KO SYCP3.jpg]

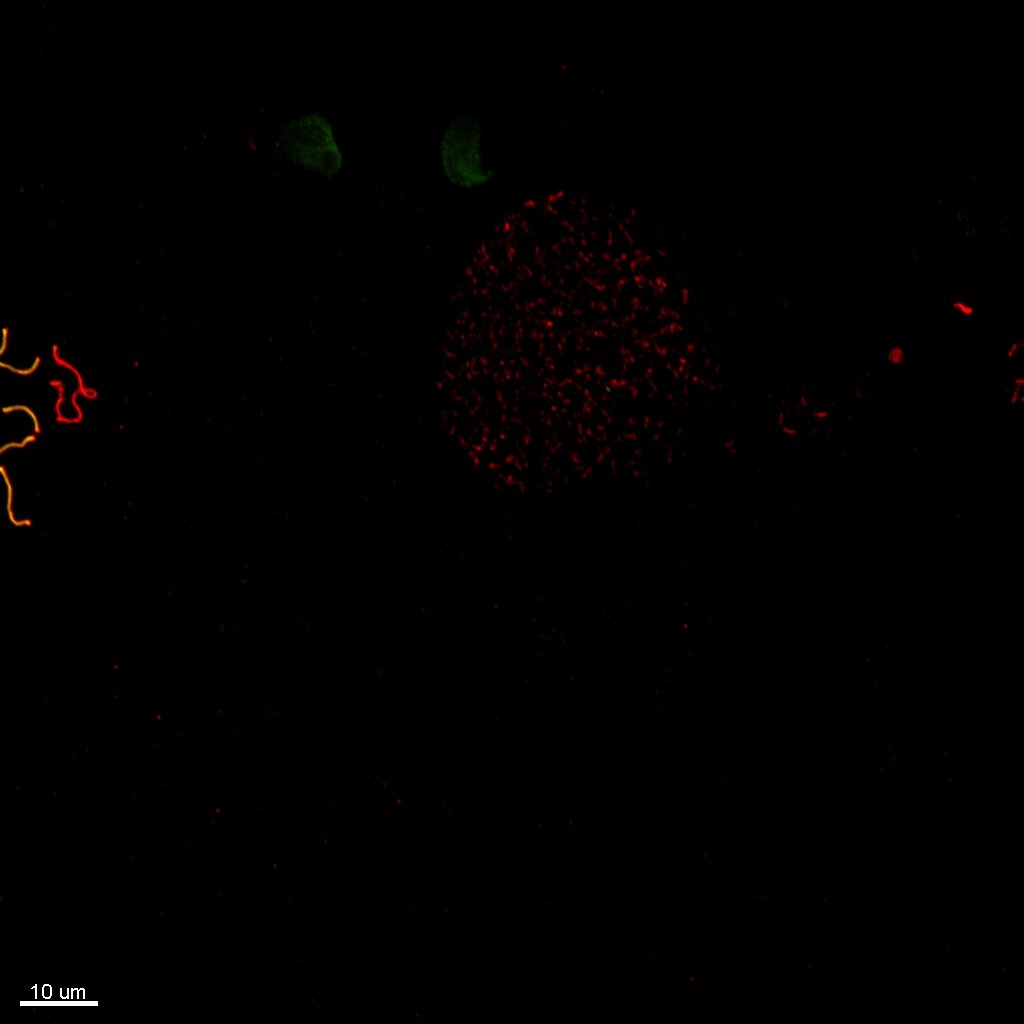

Supplement: Figure 2—figure supplement 1—source data 1. [file elife-83129-fig2-figsupp1-data1.zip › Figure supplement S3-source data 10/Leptotene/WT MERGE.jpg]
