# Supplementary figures and images for: The Slingshot phosphatase 2 is required for acrosome biogenesis during spermatogenesis in mice (part 2 of 4)
Source: eLife. 2023 Mar 21;12:e83129. doi: 10.7554/eLife.83129 (PMC10065795; doi:10.7554/eLife.83129)

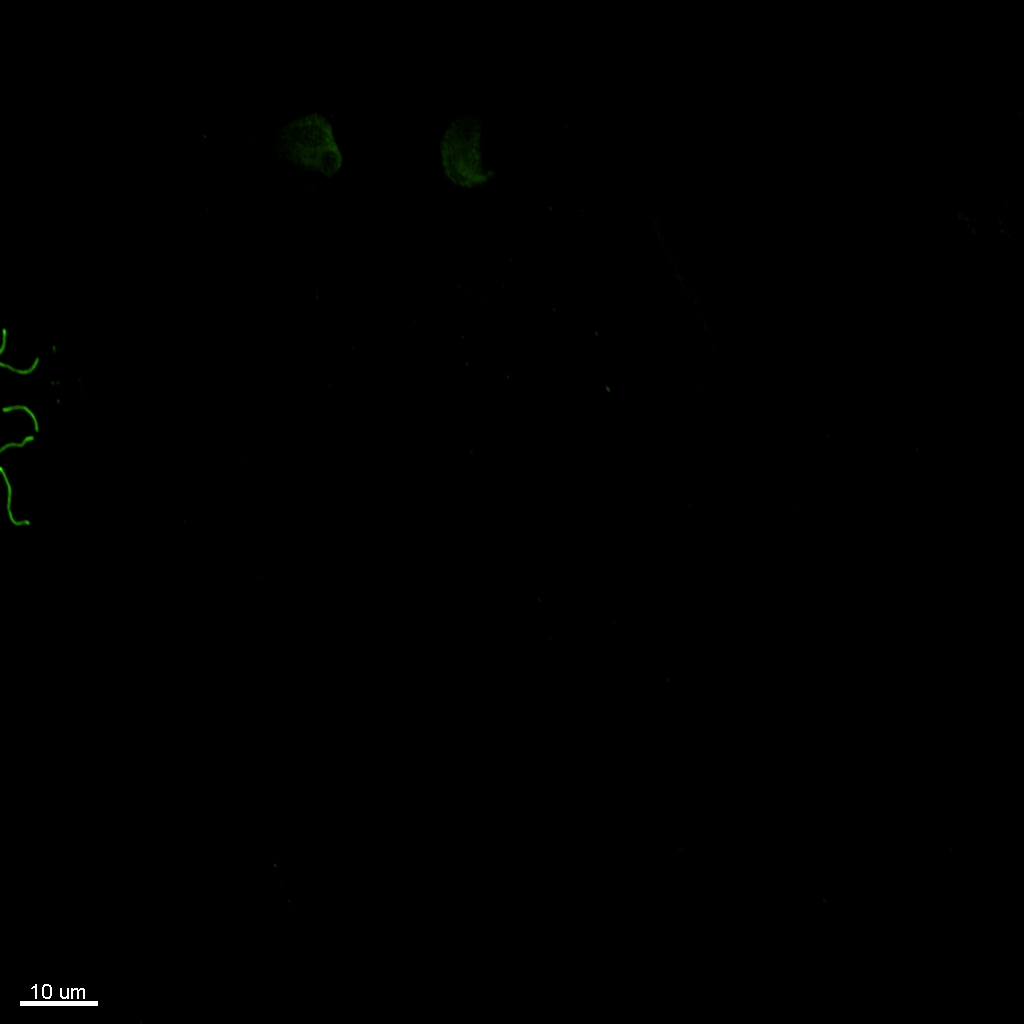

Supplement: Figure 2—figure supplement 1—source data 1. [file elife-83129-fig2-figsupp1-data1.zip › Figure supplement S3-source data 10/Leptotene/WT SYCP1.jpg]

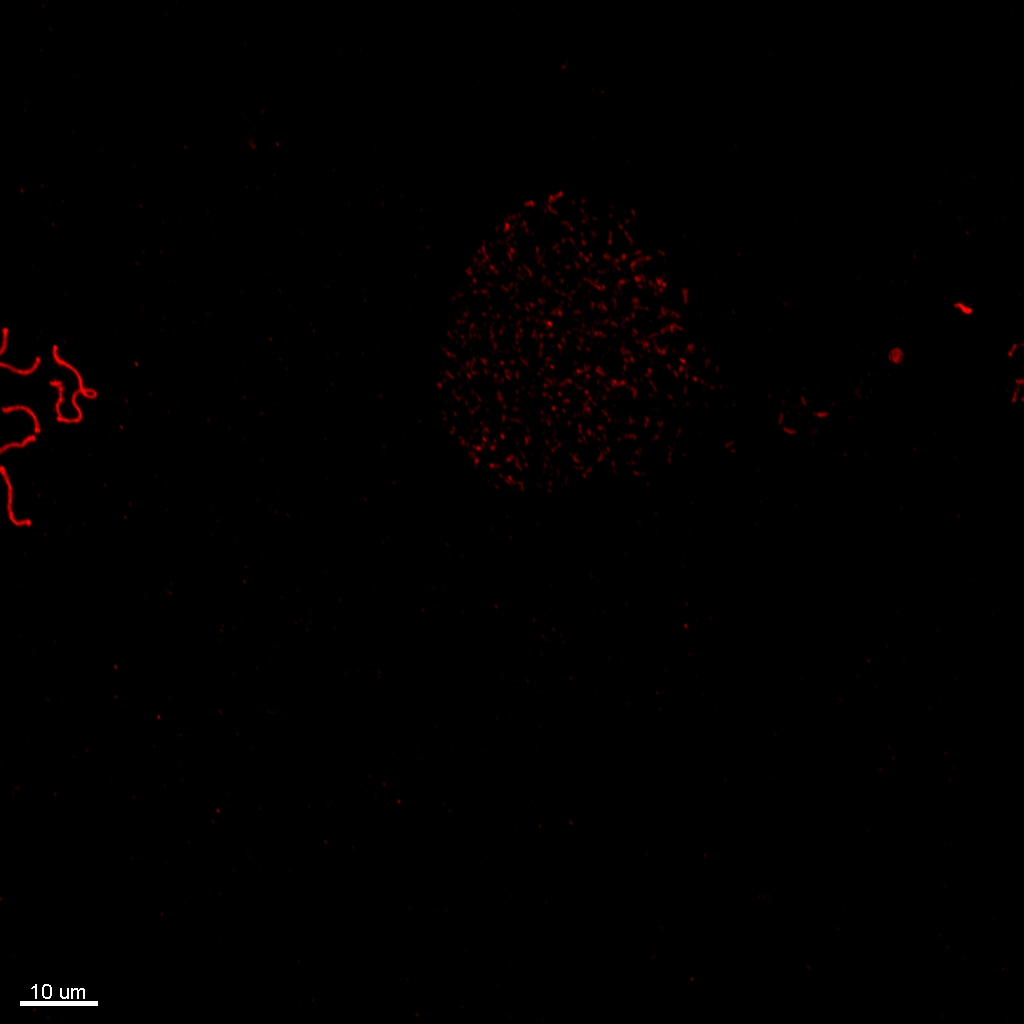

Supplement: Figure 2—figure supplement 1—source data 1. [file elife-83129-fig2-figsupp1-data1.zip › Figure supplement S3-source data 10/Leptotene/WT SYCP3.jpg]

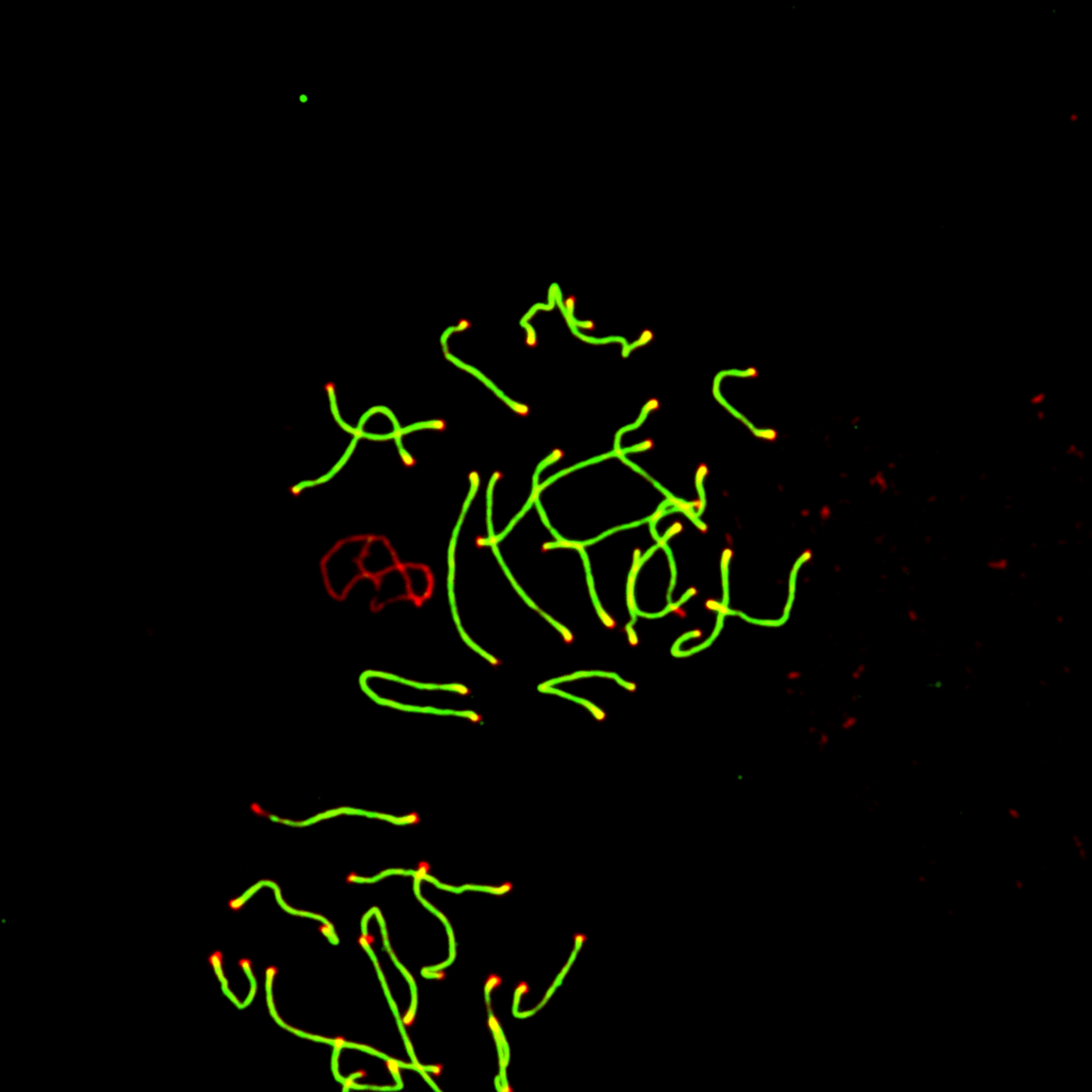

Supplement: Figure 2—figure supplement 1—source data 1. [file elife-83129-fig2-figsupp1-data1.zip › Figure supplement S3-source data 10/Pachytene/KO MERGE.tif]

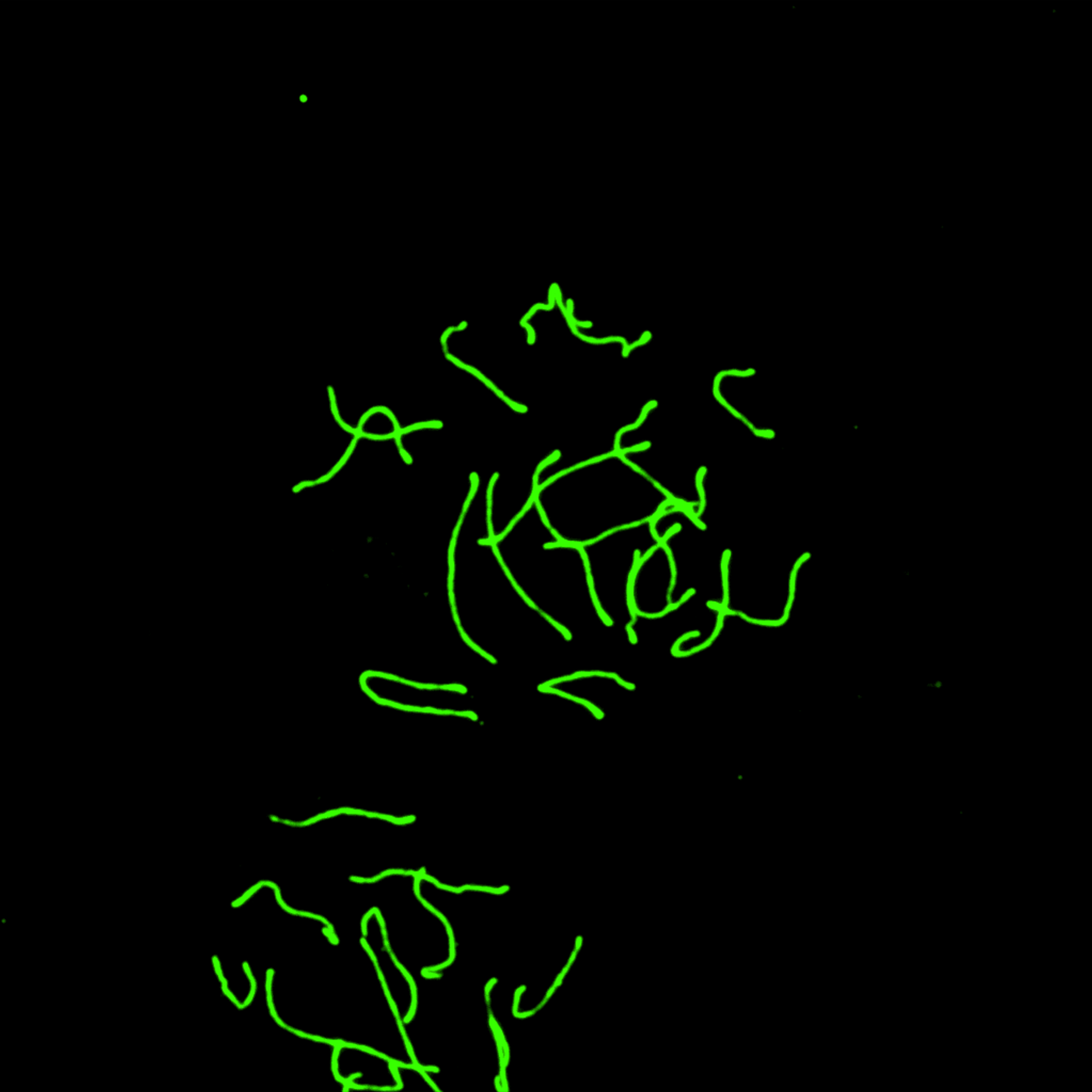

Supplement: Figure 2—figure supplement 1—source data 1. [file elife-83129-fig2-figsupp1-data1.zip › Figure supplement S3-source data 10/Pachytene/KO SYCP1.tif]

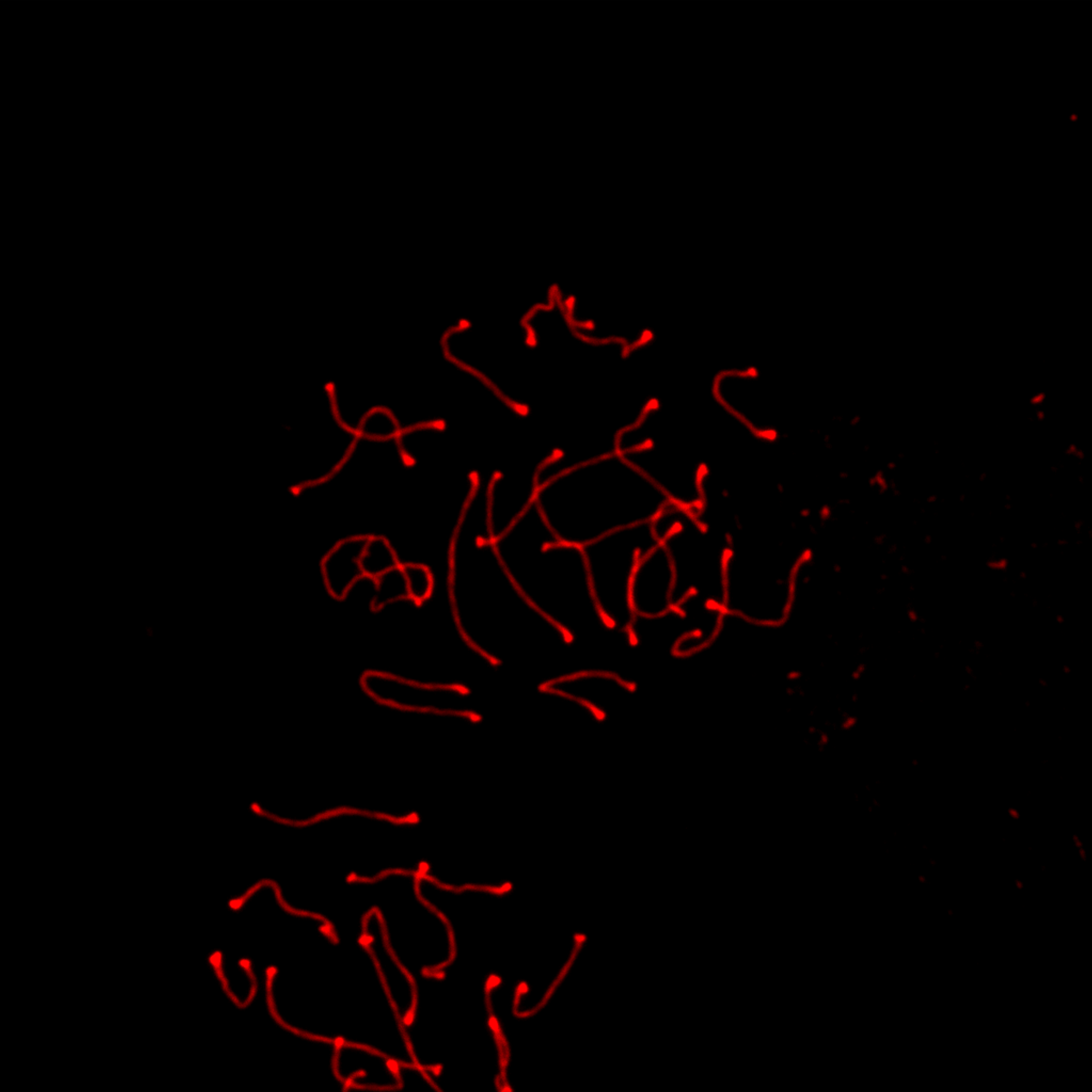

Supplement: Figure 2—figure supplement 1—source data 1. [file elife-83129-fig2-figsupp1-data1.zip › Figure supplement S3-source data 10/Pachytene/KO SYCP3.tif]

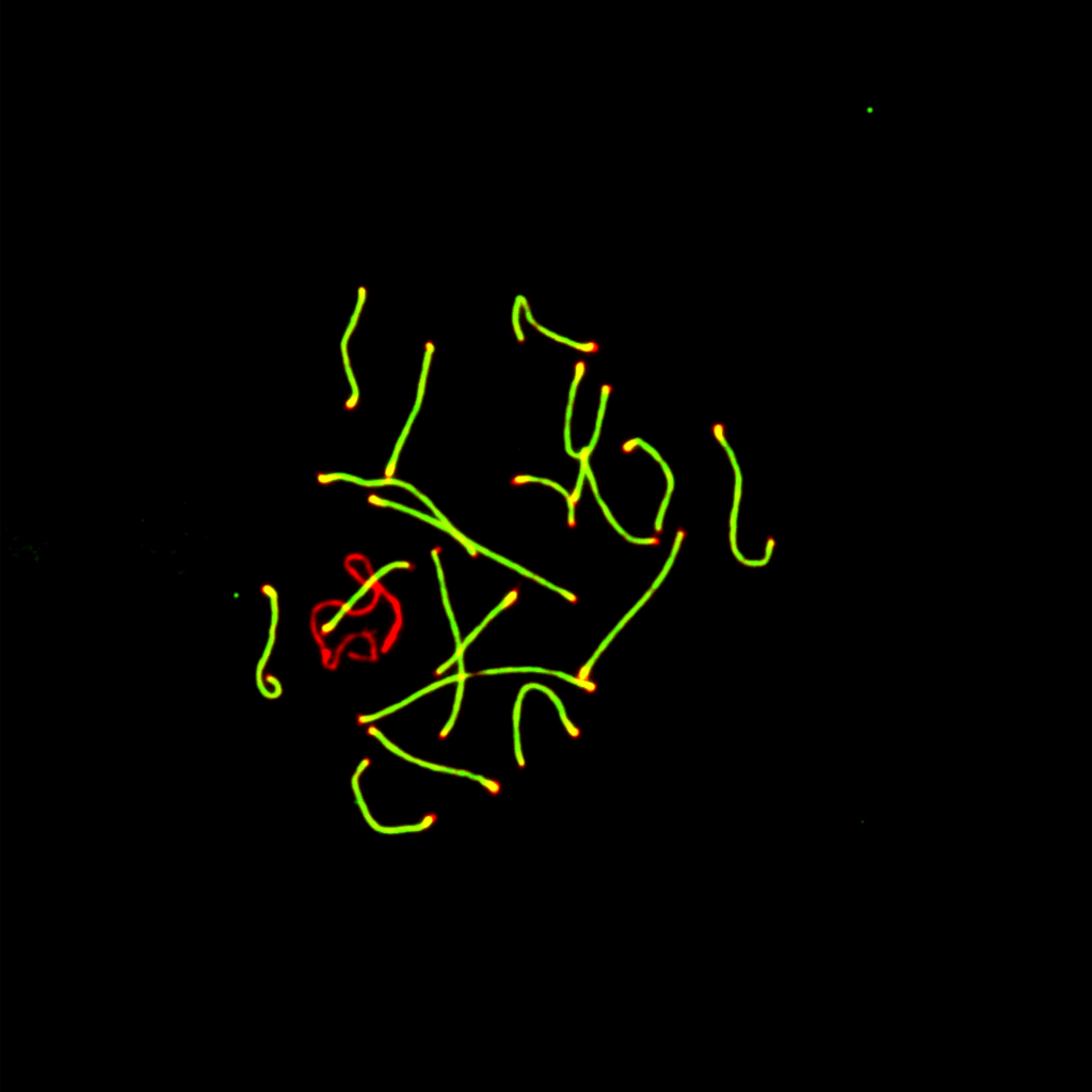

Supplement: Figure 2—figure supplement 1—source data 1. [file elife-83129-fig2-figsupp1-data1.zip › Figure supplement S3-source data 10/Pachytene/WT MERGE.tif]

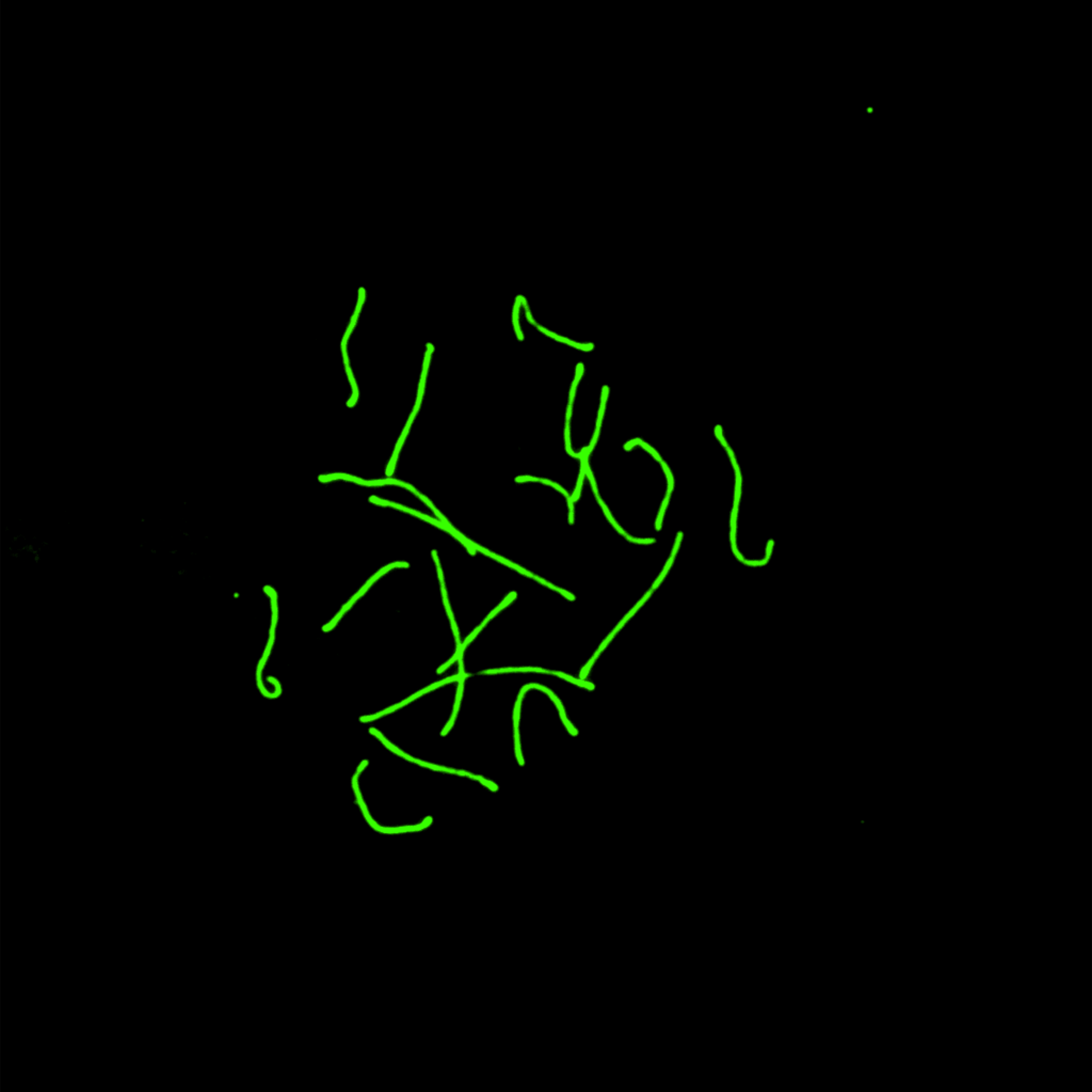

Supplement: Figure 2—figure supplement 1—source data 1. [file elife-83129-fig2-figsupp1-data1.zip › Figure supplement S3-source data 10/Pachytene/WT SYCP1.tif]

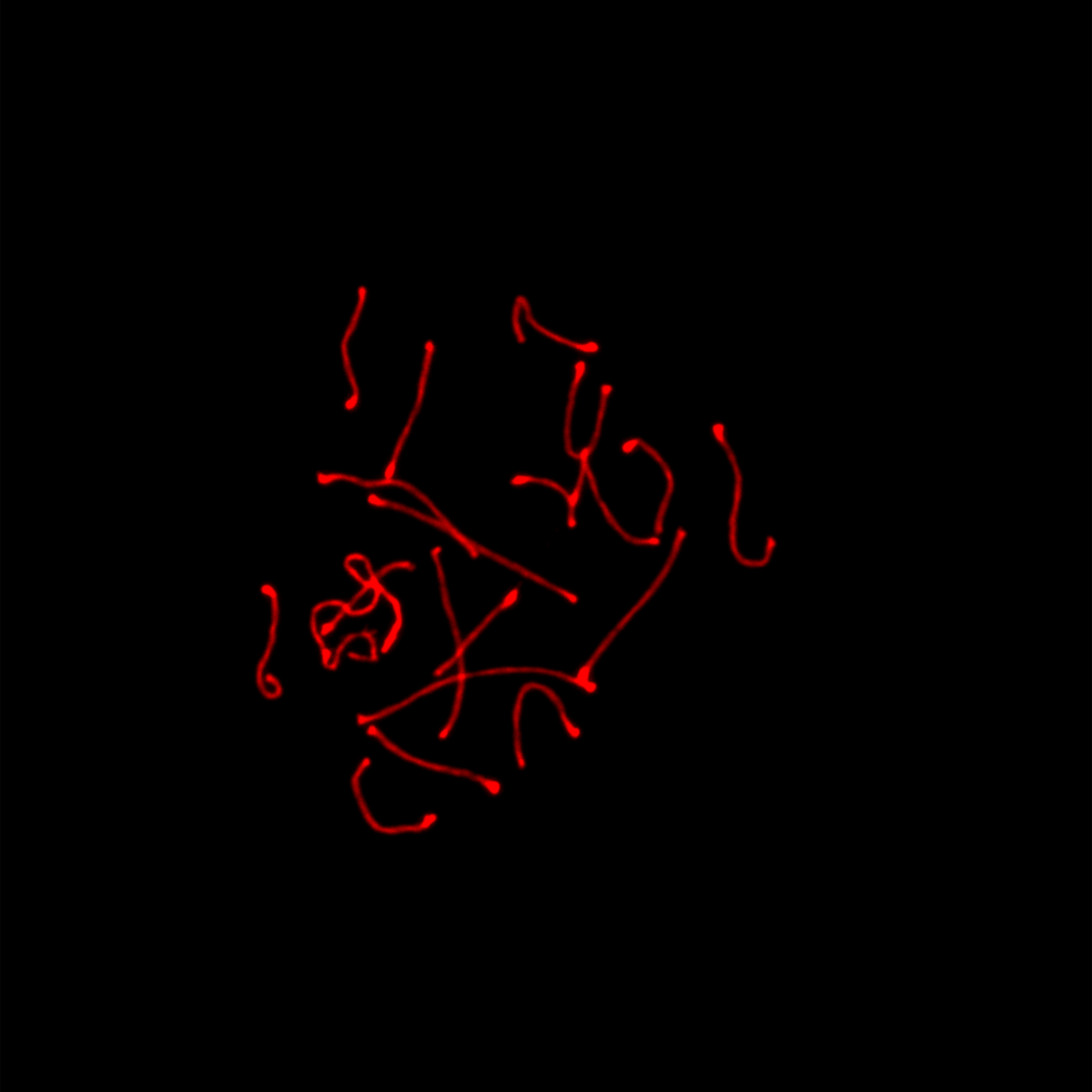

Supplement: Figure 2—figure supplement 1—source data 1. [file elife-83129-fig2-figsupp1-data1.zip › Figure supplement S3-source data 10/Pachytene/WT SYCP3.tif]

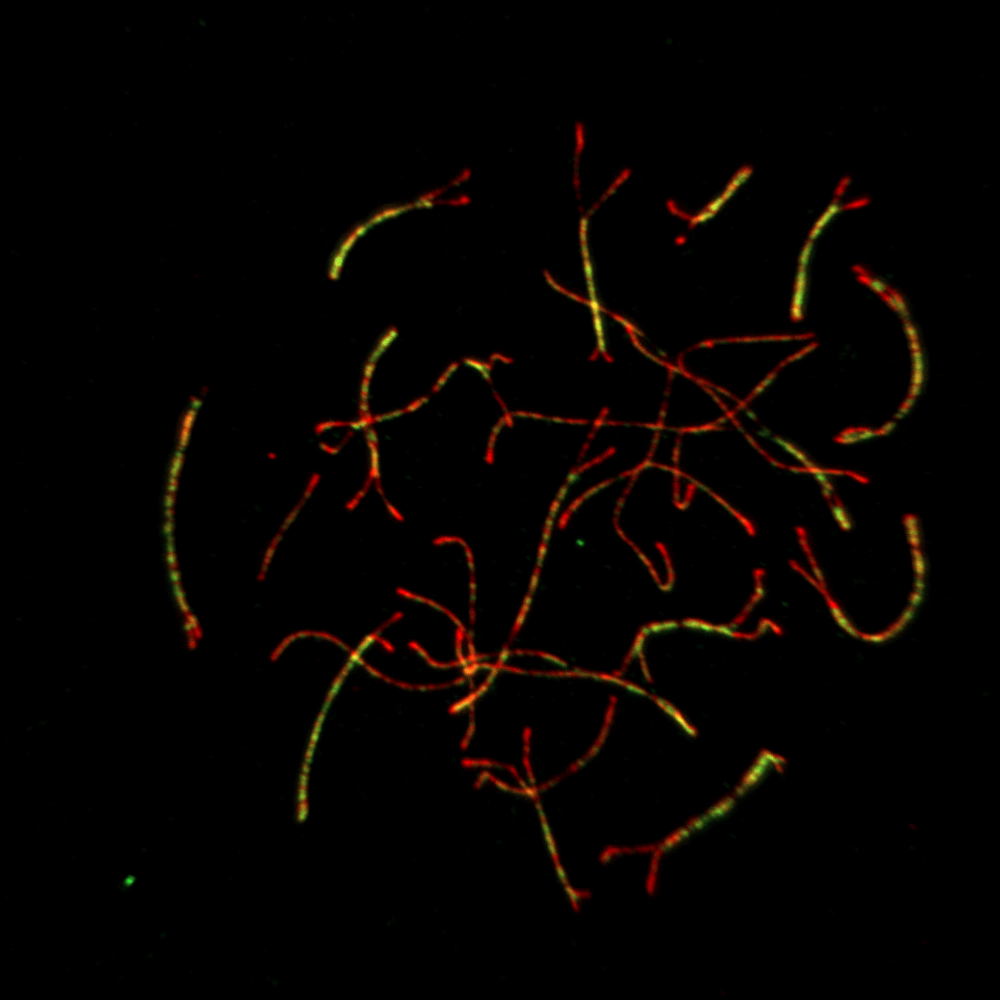

Supplement: Figure 2—figure supplement 1—source data 1. [file elife-83129-fig2-figsupp1-data1.zip › Figure supplement S3-source data 10/Zygotene/KO MERGE.tif]

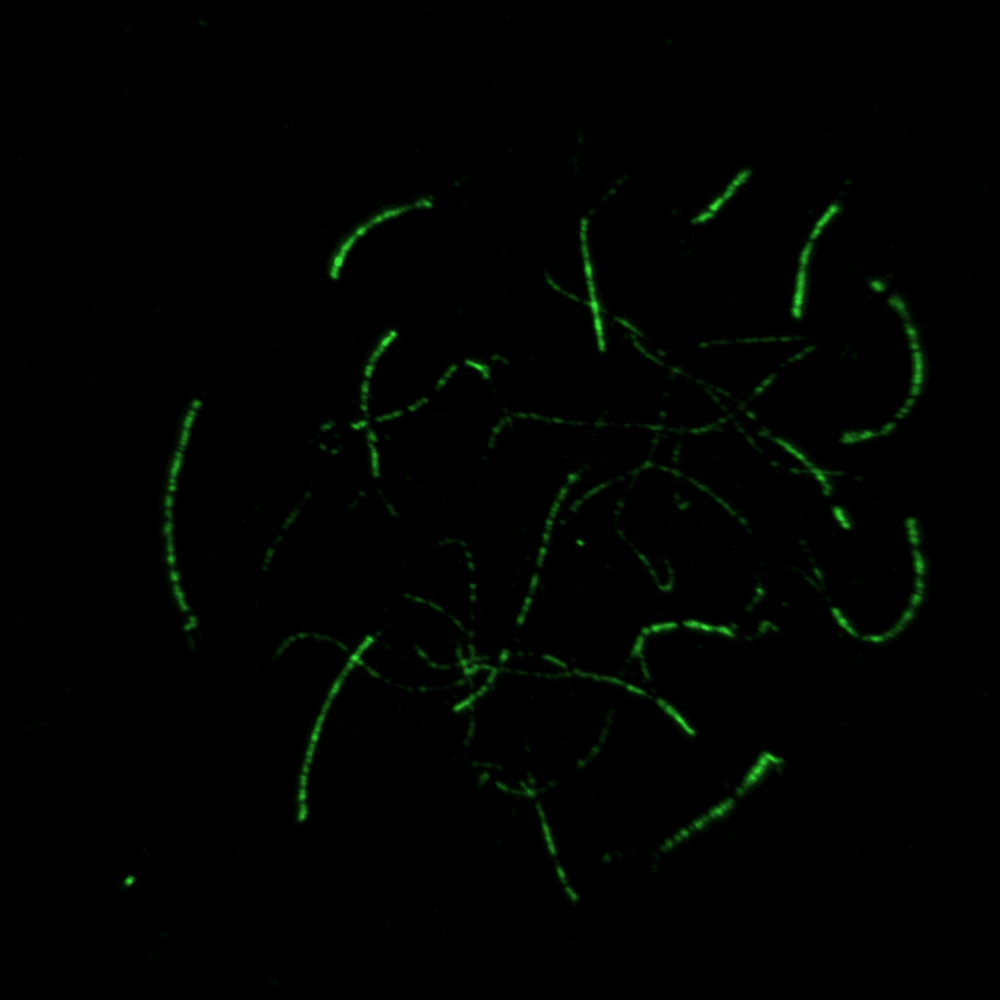

Supplement: Figure 2—figure supplement 1—source data 1. [file elife-83129-fig2-figsupp1-data1.zip › Figure supplement S3-source data 10/Zygotene/KO SYCP1.tif]

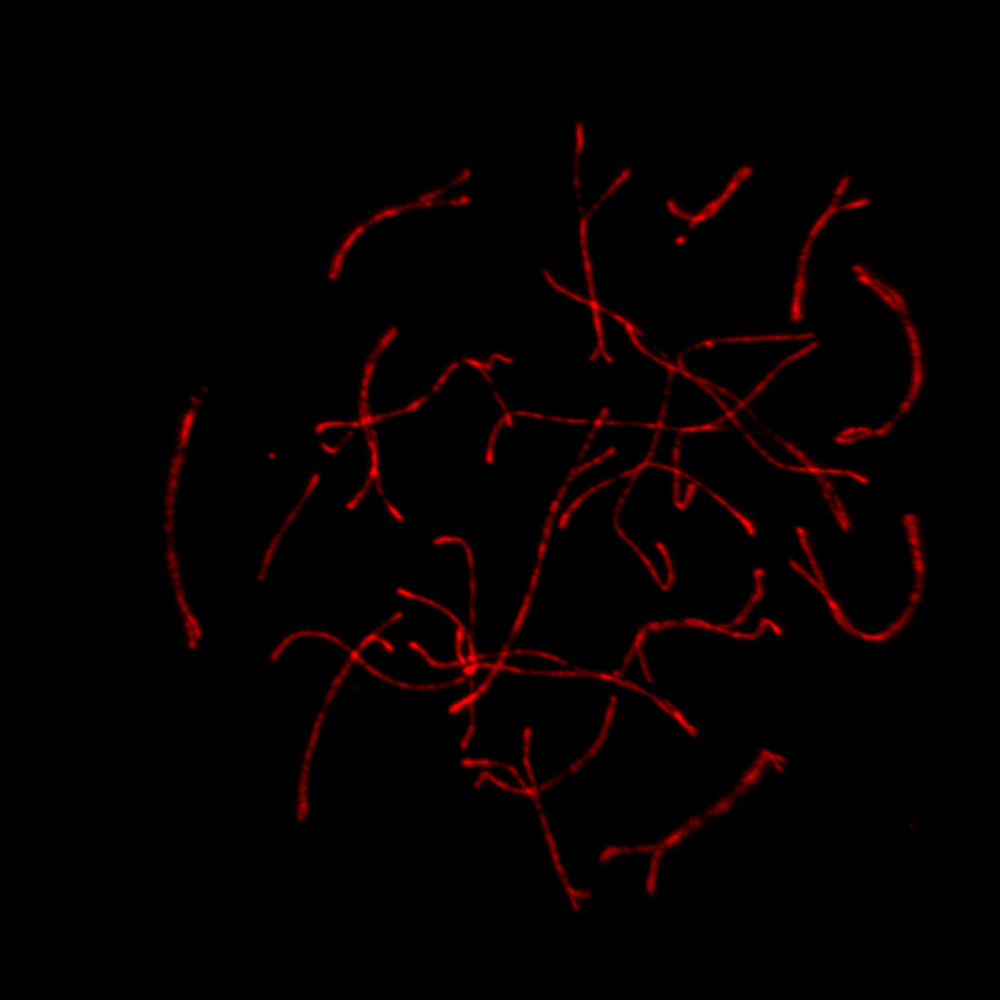

Supplement: Figure 2—figure supplement 1—source data 1. [file elife-83129-fig2-figsupp1-data1.zip › Figure supplement S3-source data 10/Zygotene/KO SYCP3.tif]

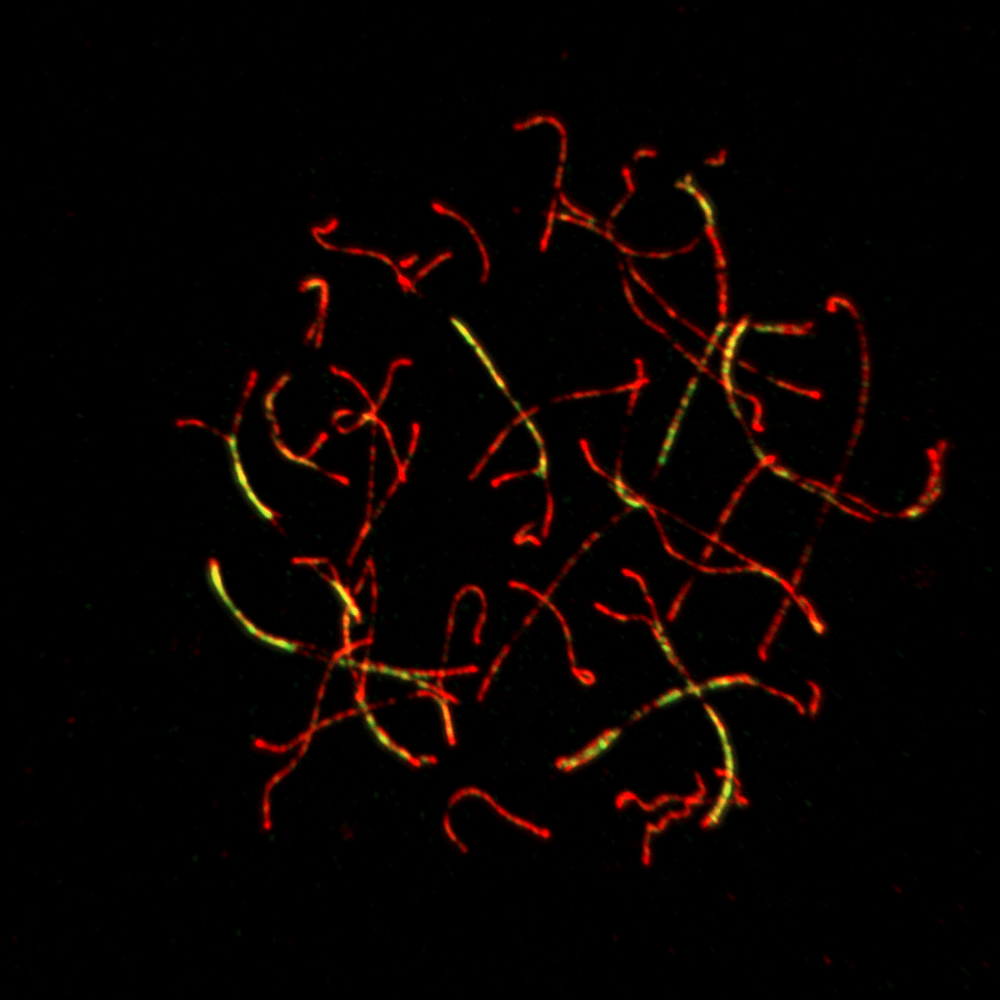

Supplement: Figure 2—figure supplement 1—source data 1. [file elife-83129-fig2-figsupp1-data1.zip › Figure supplement S3-source data 10/Zygotene/WT MERGE.tif]

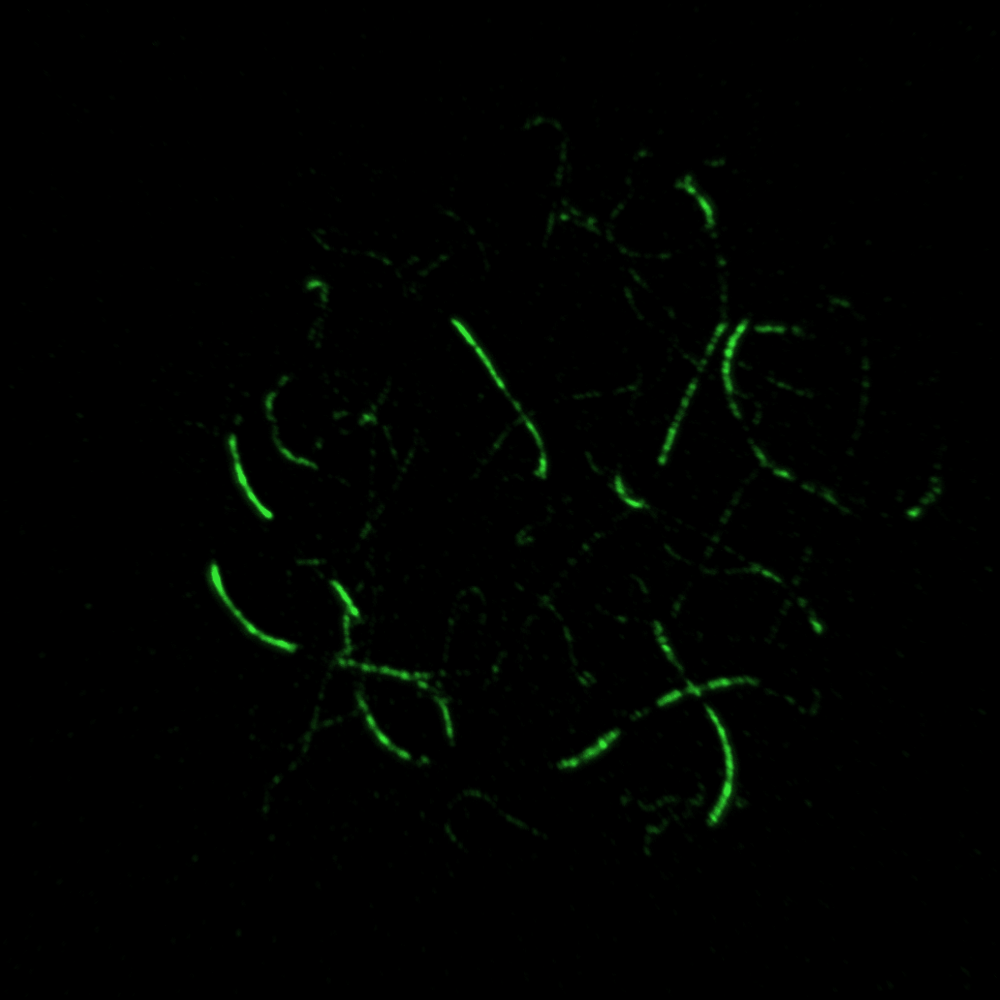

Supplement: Figure 2—figure supplement 1—source data 1. [file elife-83129-fig2-figsupp1-data1.zip › Figure supplement S3-source data 10/Zygotene/WT SYCP1.tif]

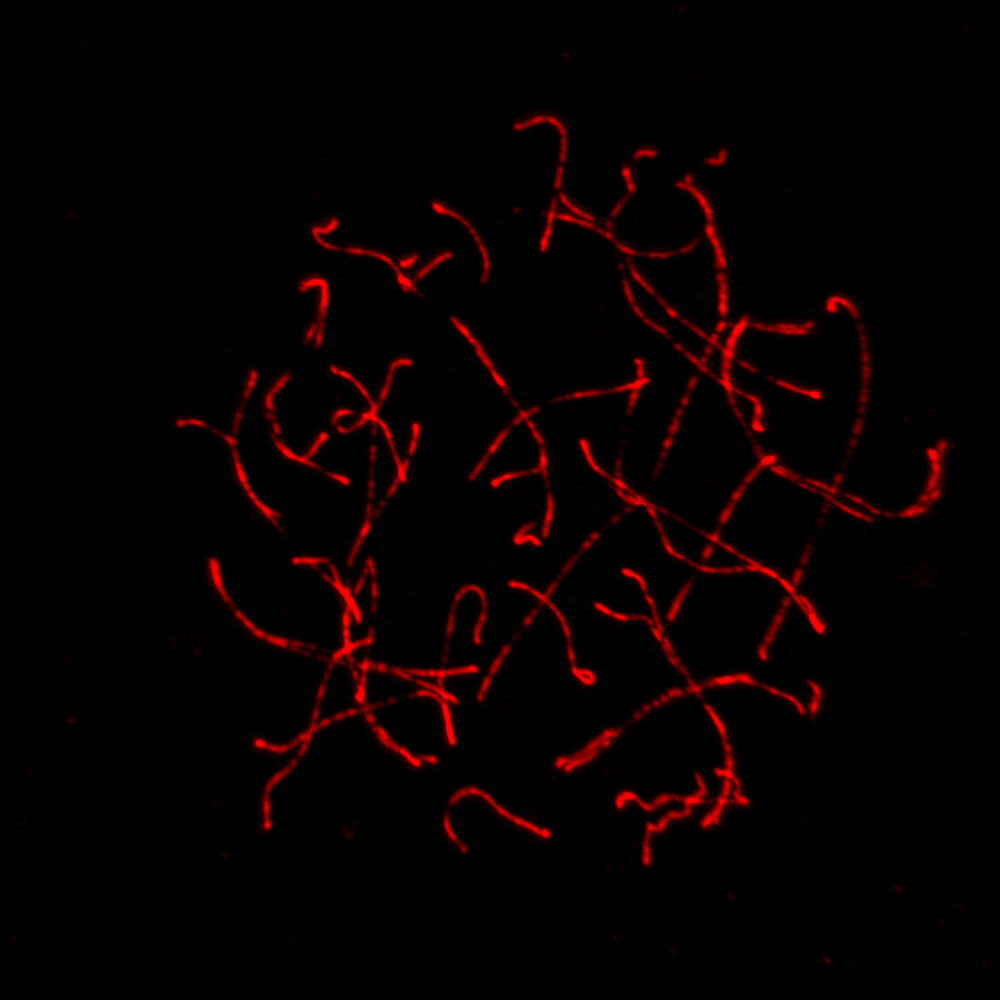

Supplement: Figure 2—figure supplement 1—source data 1. [file elife-83129-fig2-figsupp1-data1.zip › Figure supplement S3-source data 10/Zygotene/WT SYCP3.tif]

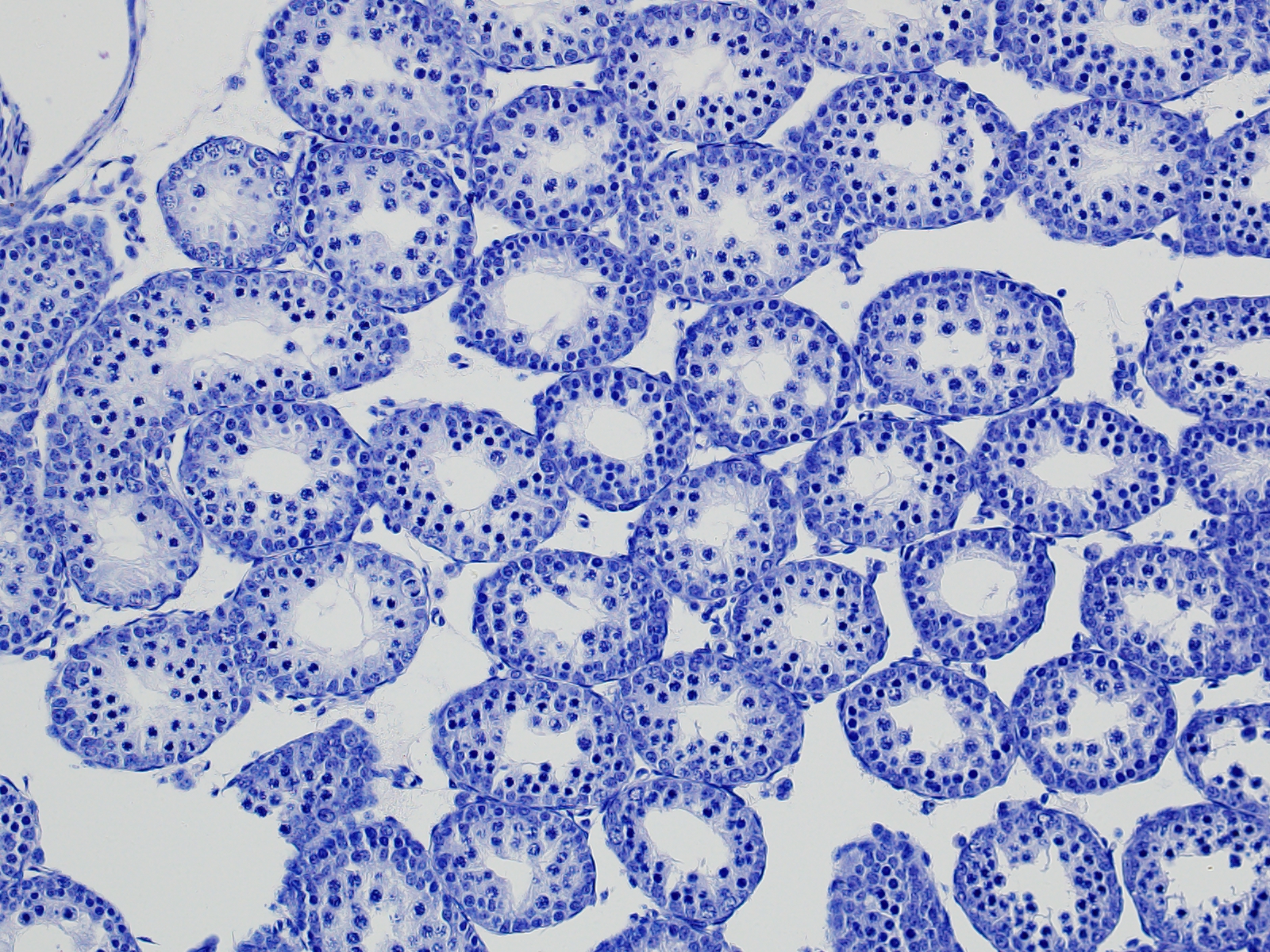

Supplement: Figure 2—figure supplement 2—source data 1. [file elife-83129-fig2-figsupp2-data1.zip › Figure supplement S4-source data 11/H Staining/hs-pd14-WT-20x-1.jpg]

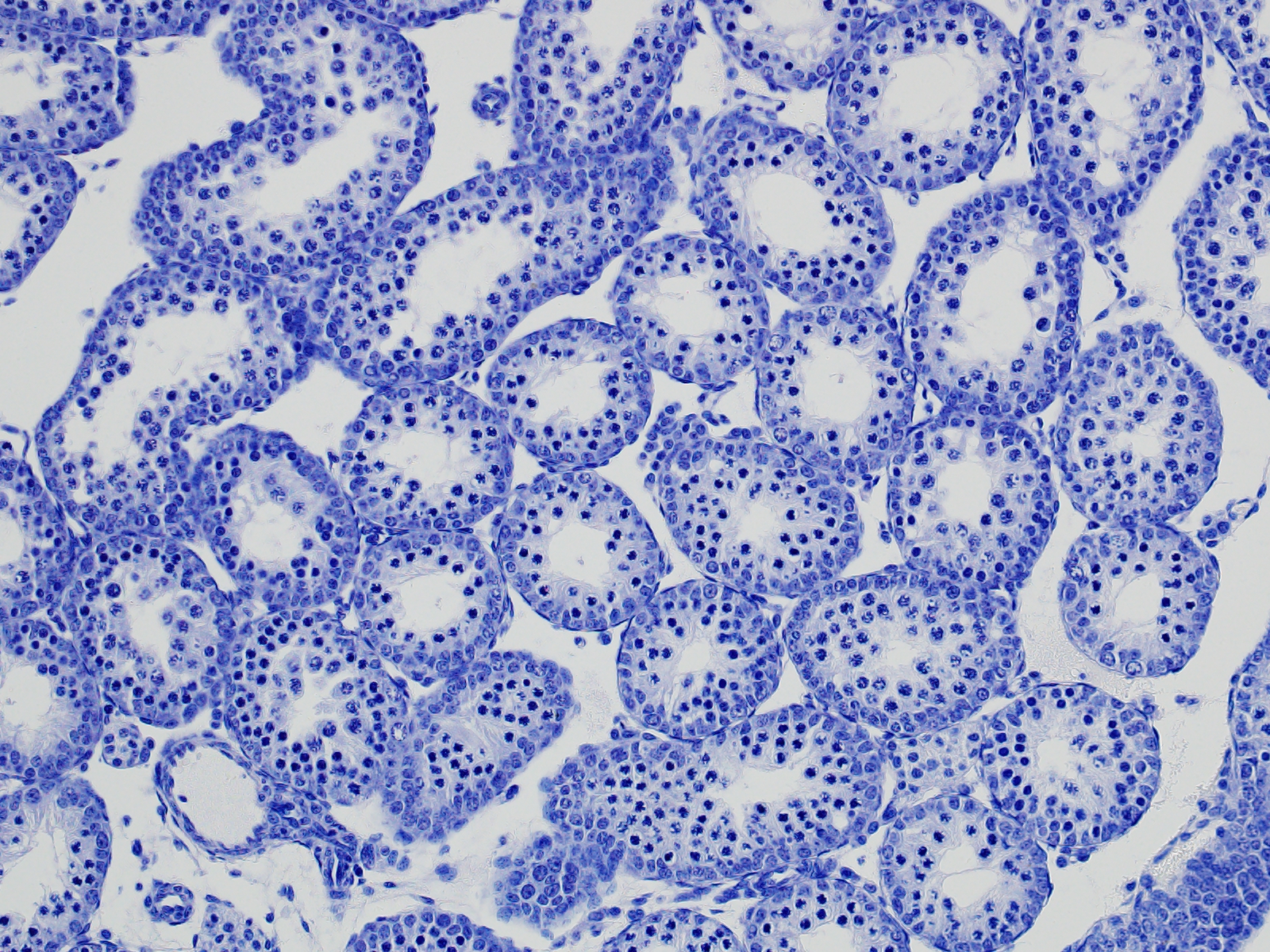

Supplement: Figure 2—figure supplement 2—source data 1. [file elife-83129-fig2-figsupp2-data1.zip › Figure supplement S4-source data 11/H Staining/hs-pd14-WT-20x.jpg]

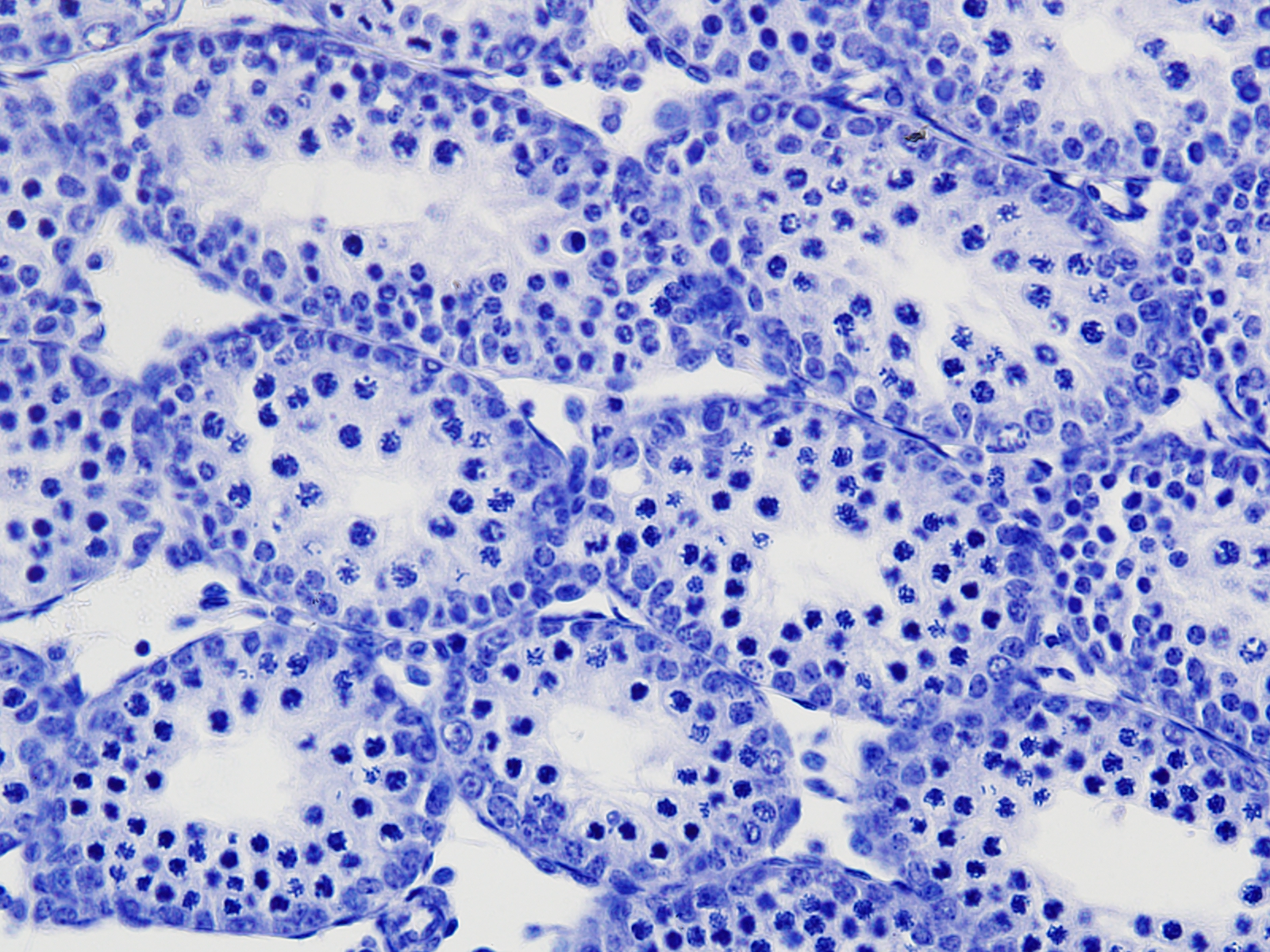

Supplement: Figure 2—figure supplement 2—source data 1. [file elife-83129-fig2-figsupp2-data1.zip › Figure supplement S4-source data 11/H Staining/hs-pd14-WT-40x (2).jpg]

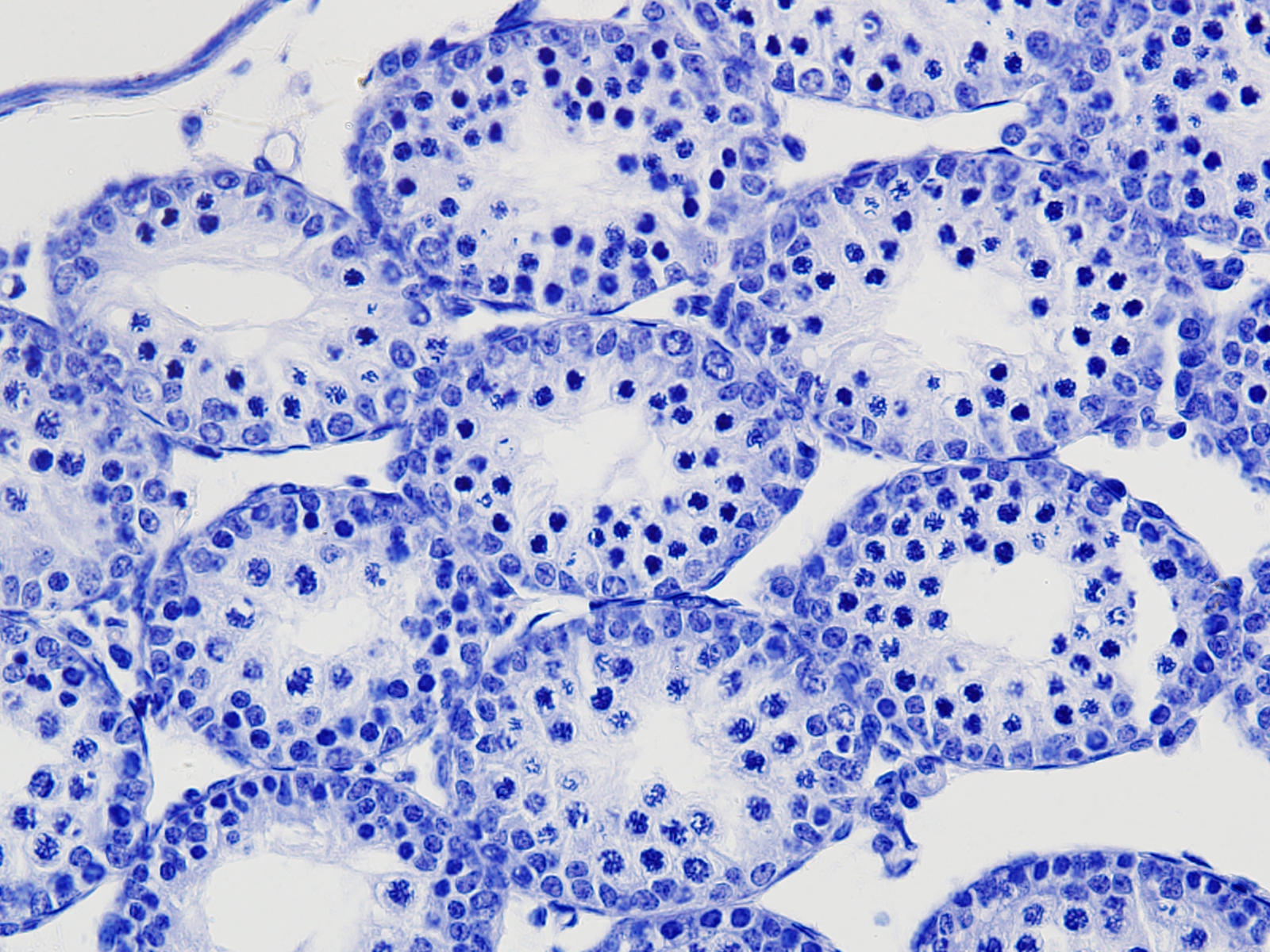

Supplement: Figure 2—figure supplement 2—source data 1. [file elife-83129-fig2-figsupp2-data1.zip › Figure supplement S4-source data 11/H Staining/hs-pd14-WT-40x.jpg]

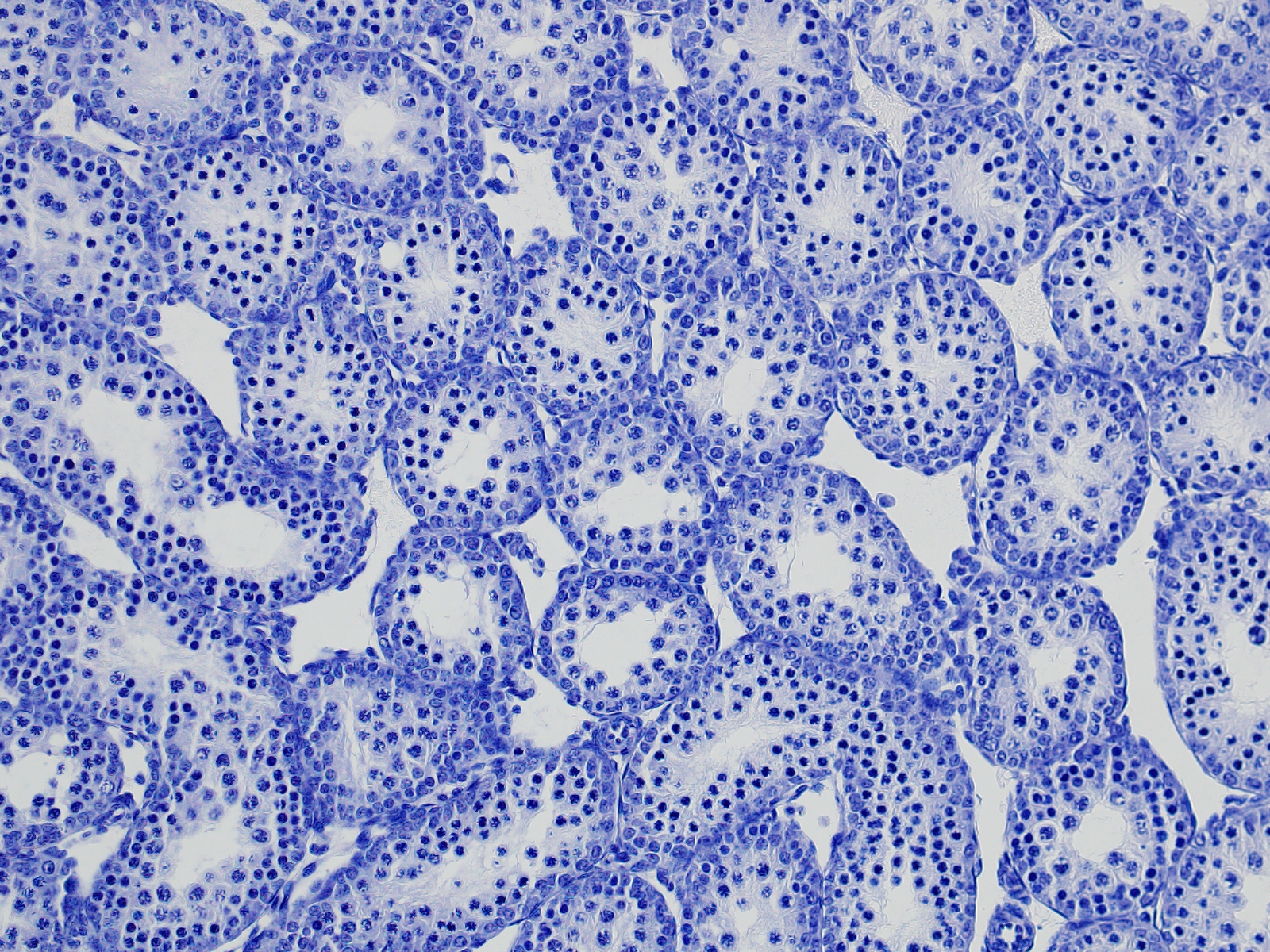

Supplement: Figure 2—figure supplement 2—source data 1. [file elife-83129-fig2-figsupp2-data1.zip › Figure supplement S4-source data 11/H Staining/hs-pd14-ko-20x-1.jpg]

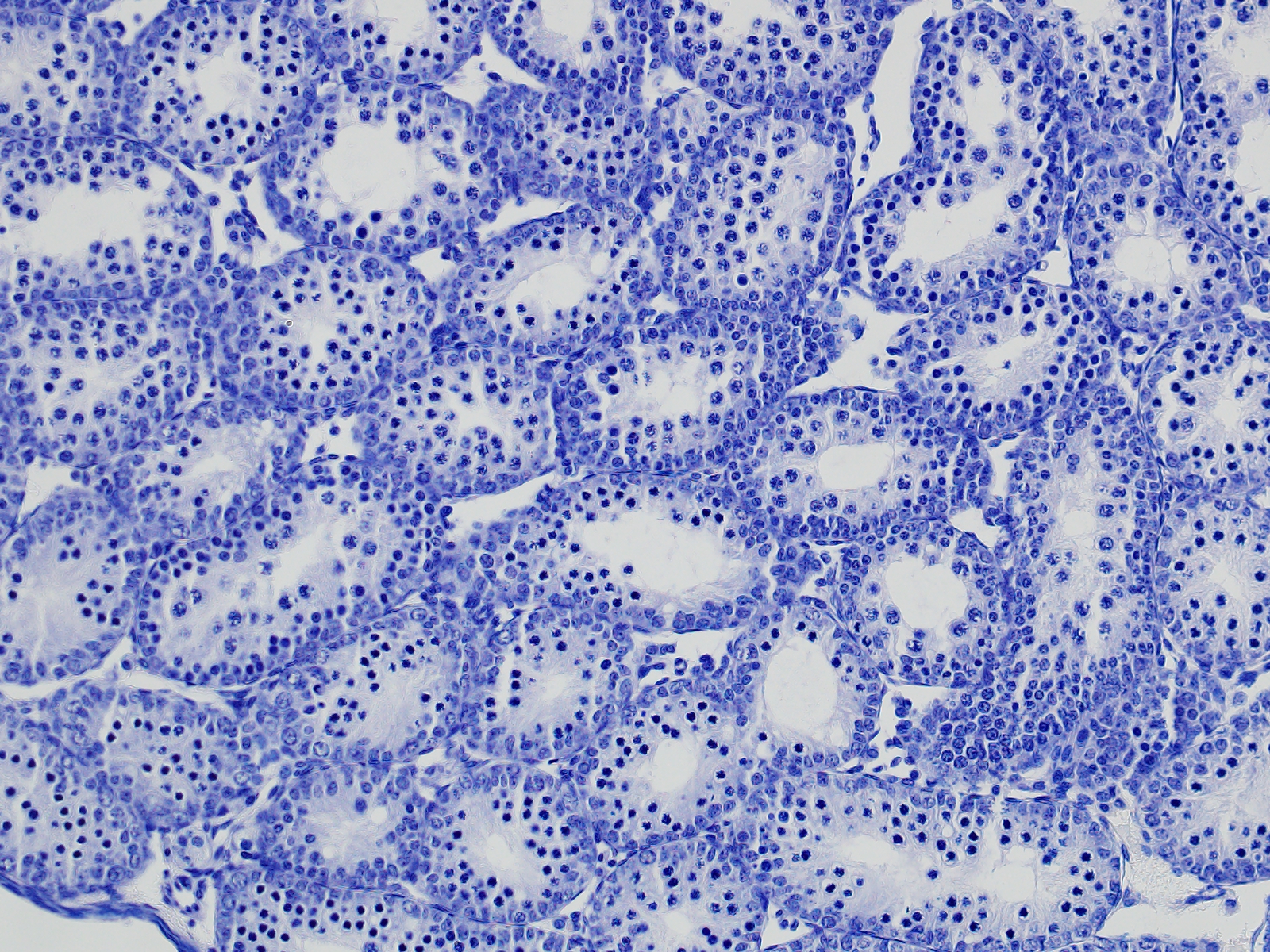

Supplement: Figure 2—figure supplement 2—source data 1. [file elife-83129-fig2-figsupp2-data1.zip › Figure supplement S4-source data 11/H Staining/hs-pd14-ko-20x.jpg]

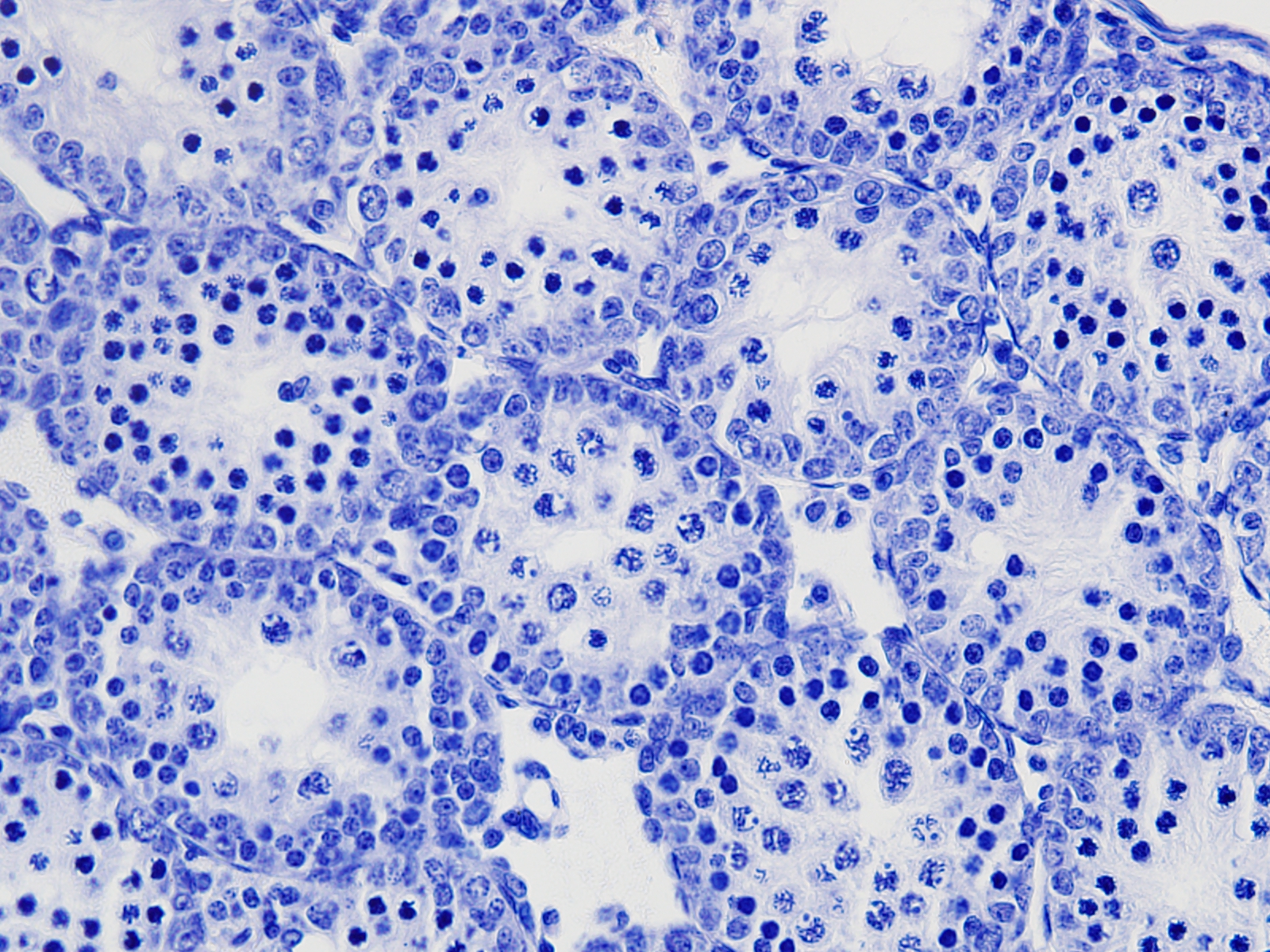

Supplement: Figure 2—figure supplement 2—source data 1. [file elife-83129-fig2-figsupp2-data1.zip › Figure supplement S4-source data 11/H Staining/hs-pd14-ko-40x-1.jpg]

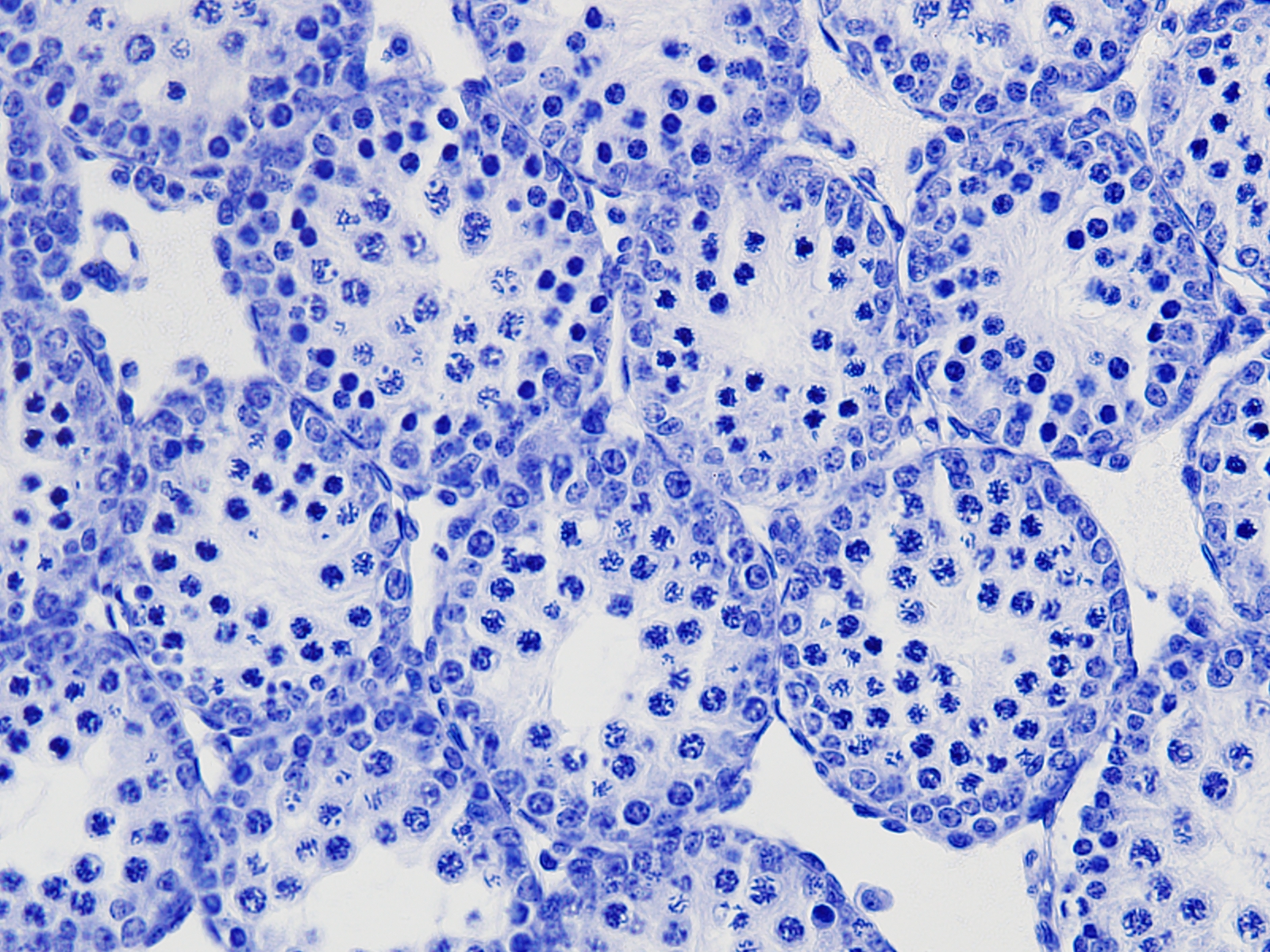

Supplement: Figure 2—figure supplement 2—source data 1. [file elife-83129-fig2-figsupp2-data1.zip › Figure supplement S4-source data 11/H Staining/hs-pd14-ko-40x.jpg]

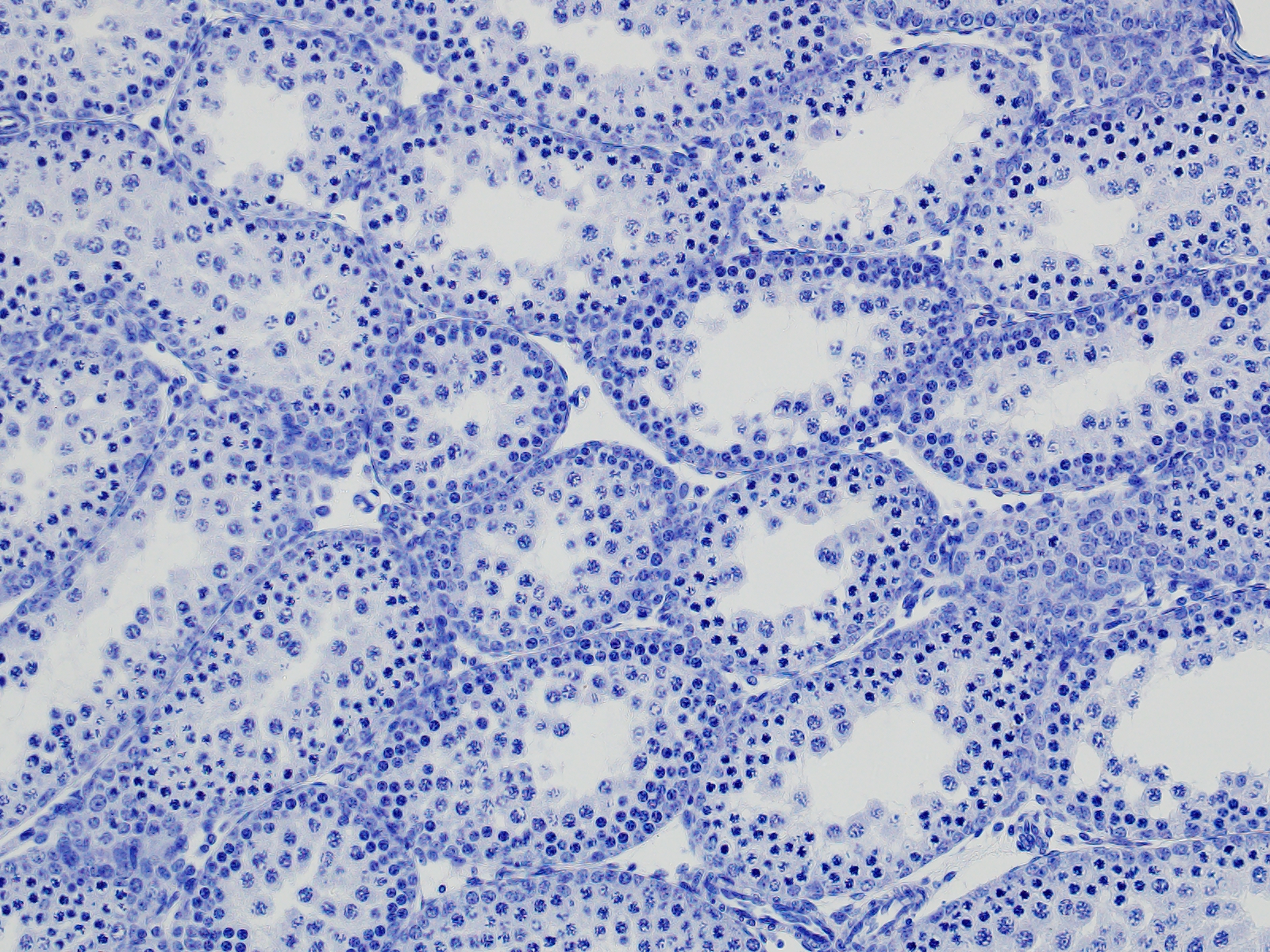

Supplement: Figure 2—figure supplement 2—source data 1. [file elife-83129-fig2-figsupp2-data1.zip › Figure supplement S4-source data 11/H Staining/hs-pd21-ko-20x.jpg]

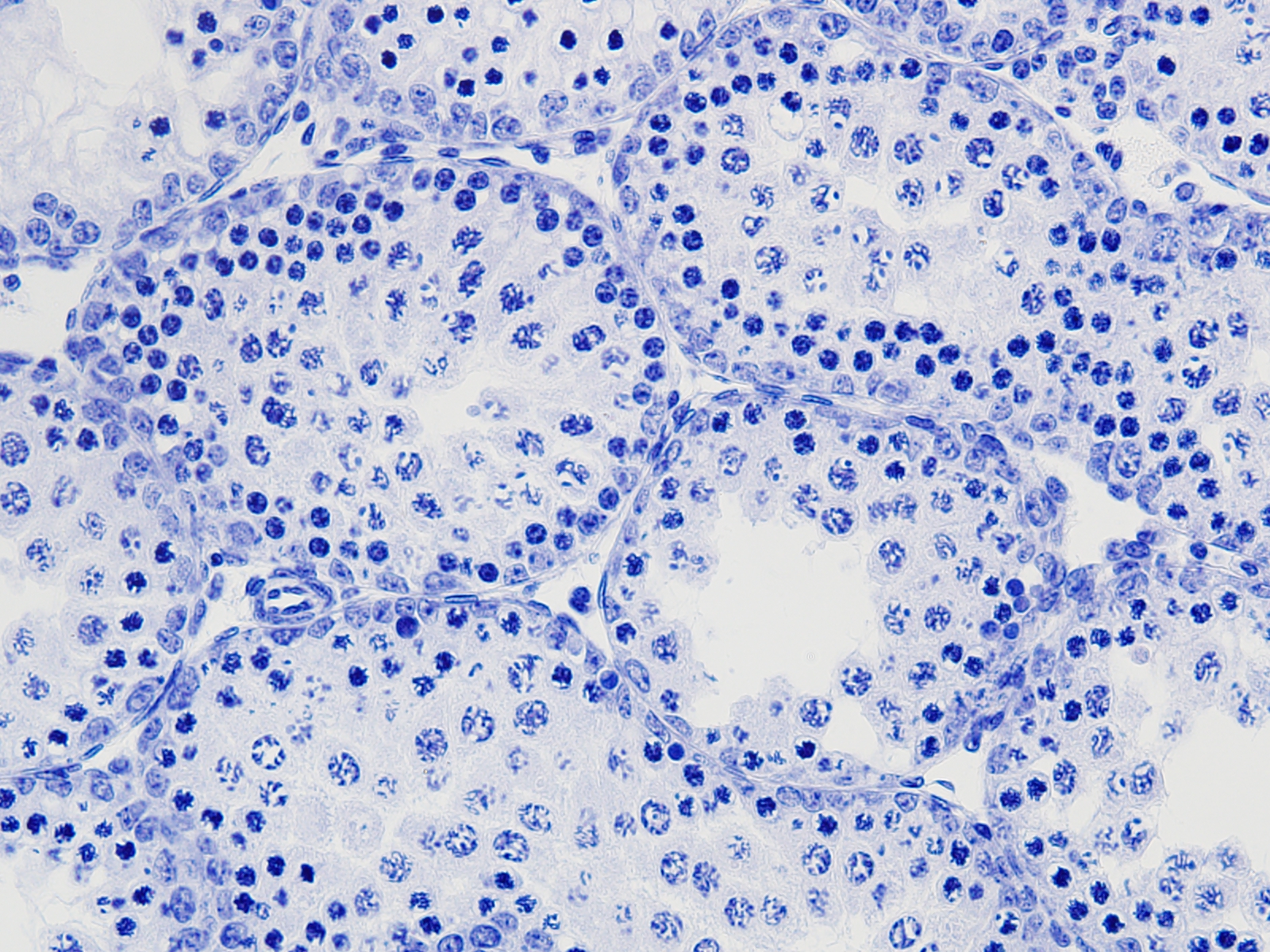

Supplement: Figure 2—figure supplement 2—source data 1. [file elife-83129-fig2-figsupp2-data1.zip › Figure supplement S4-source data 11/H Staining/hs-pd21-ko-40x-1.jpg]

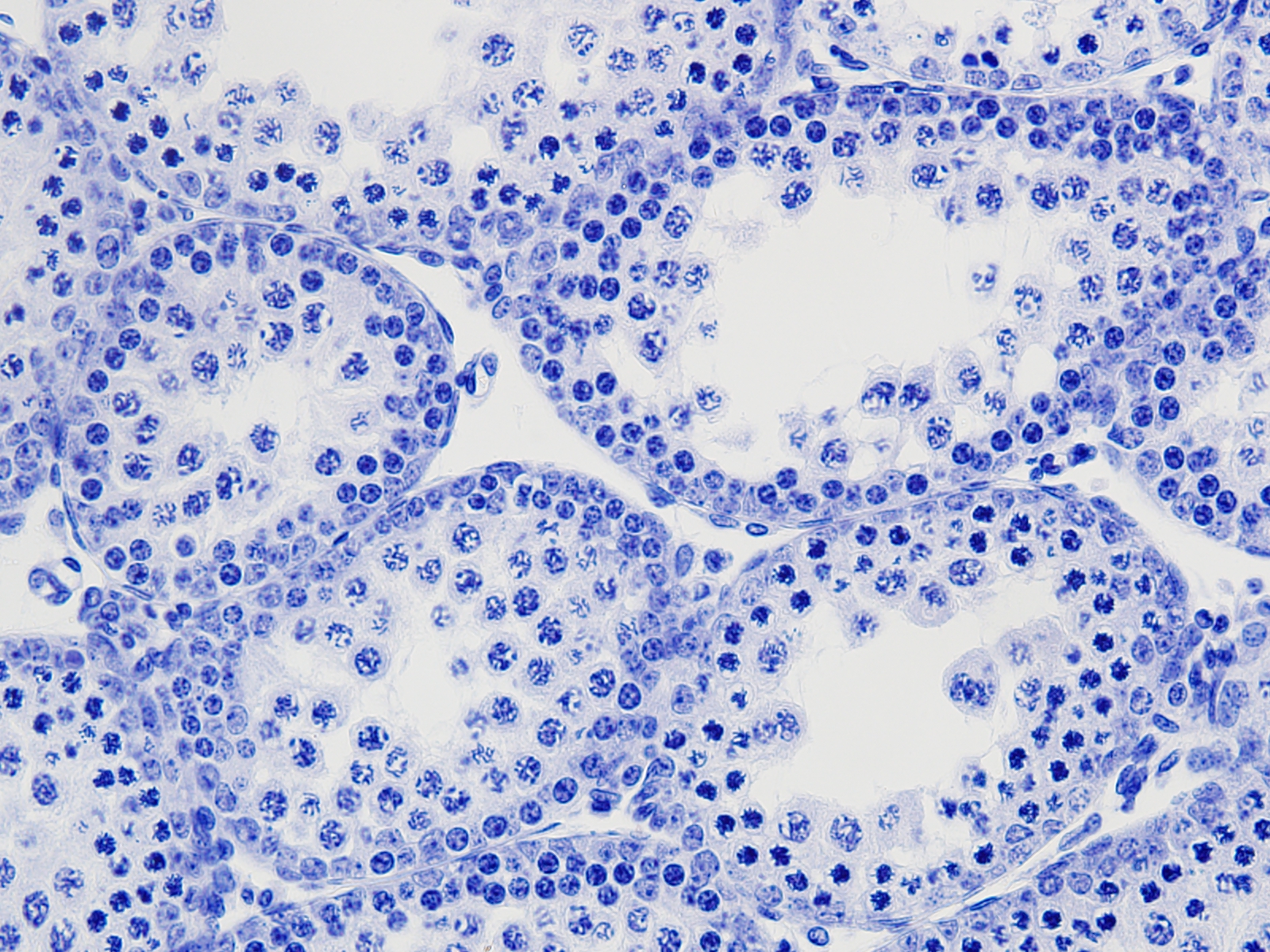

Supplement: Figure 2—figure supplement 2—source data 1. [file elife-83129-fig2-figsupp2-data1.zip › Figure supplement S4-source data 11/H Staining/hs-pd21-ko-40x.jpg]

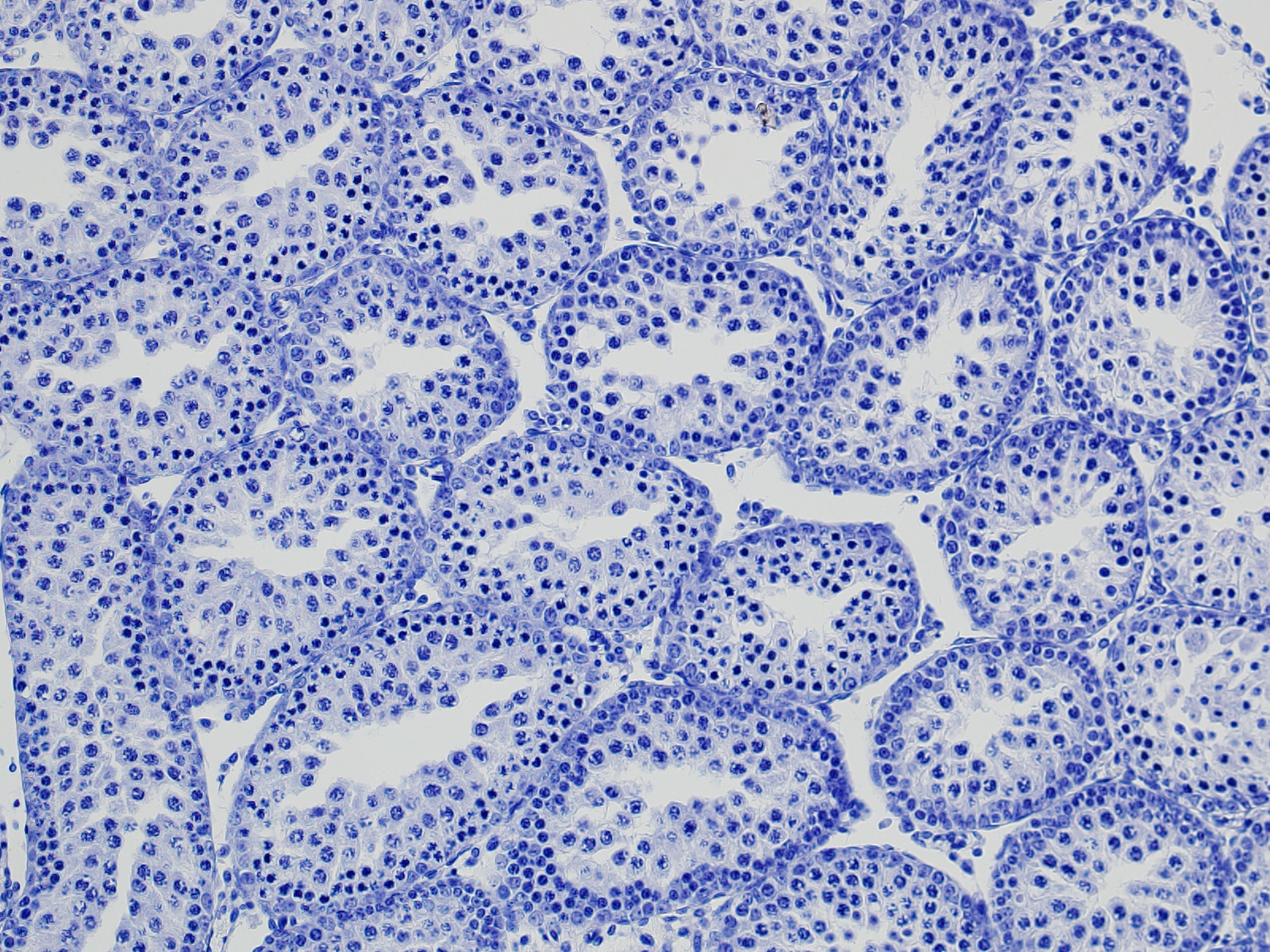

Supplement: Figure 2—figure supplement 2—source data 1. [file elife-83129-fig2-figsupp2-data1.zip › Figure supplement S4-source data 11/H Staining/hs-pd21-wt-20x.jpg]

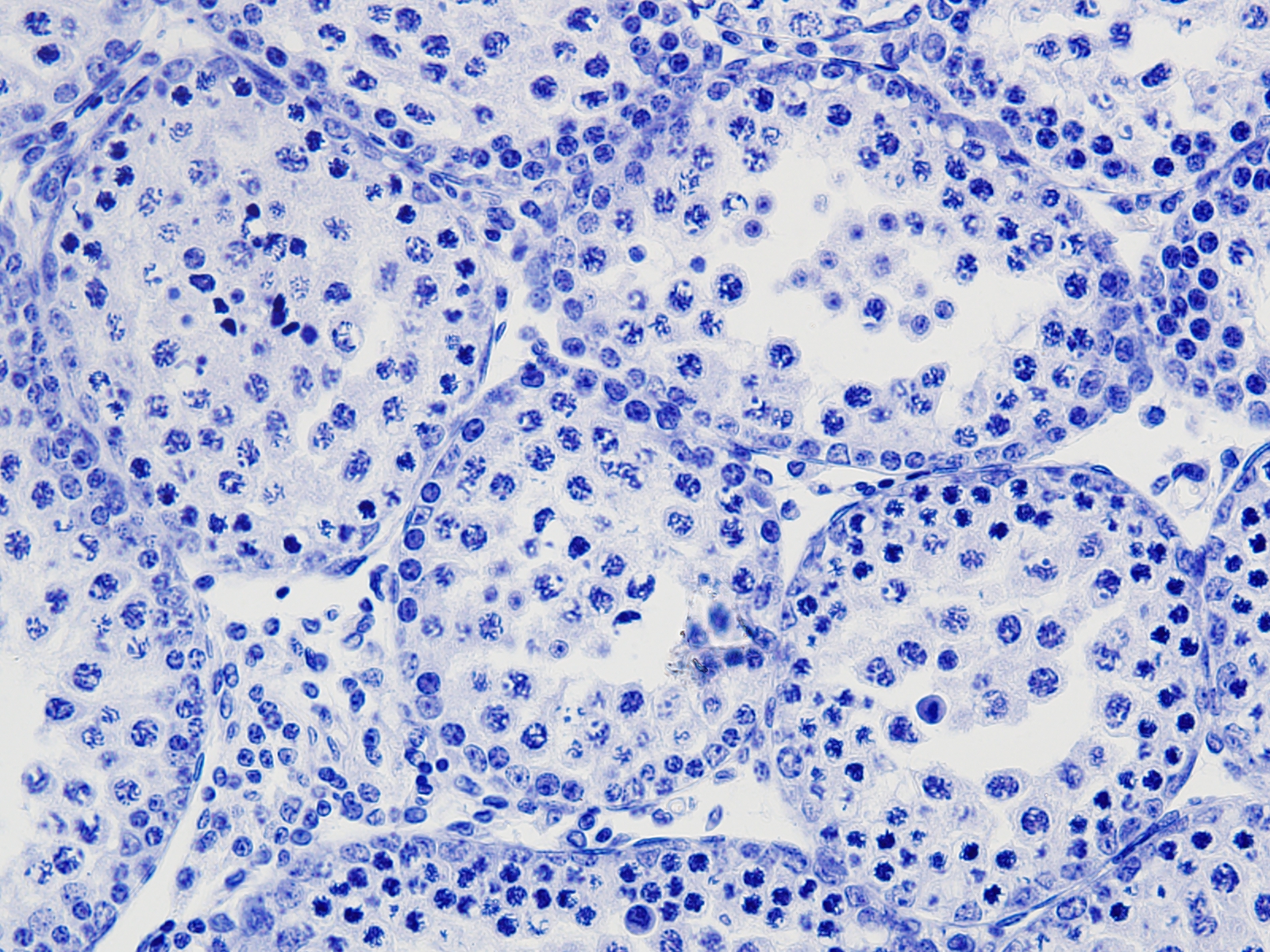

Supplement: Figure 2—figure supplement 2—source data 1. [file elife-83129-fig2-figsupp2-data1.zip › Figure supplement S4-source data 11/H Staining/hs-pd21-wt-40x-1.jpg]

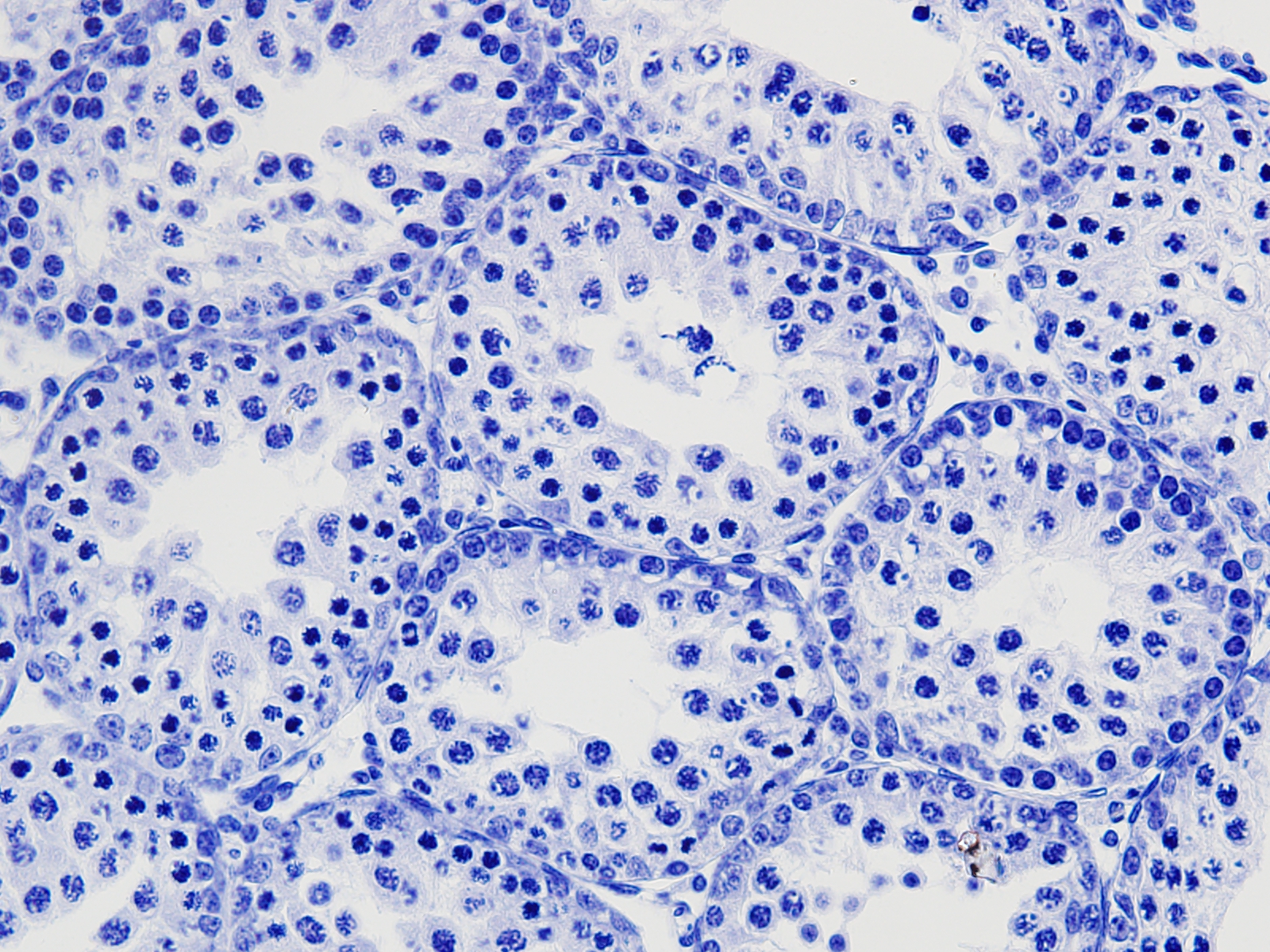

Supplement: Figure 2—figure supplement 2—source data 1. [file elife-83129-fig2-figsupp2-data1.zip › Figure supplement S4-source data 11/H Staining/hs-pd21-wt-40x.jpg]

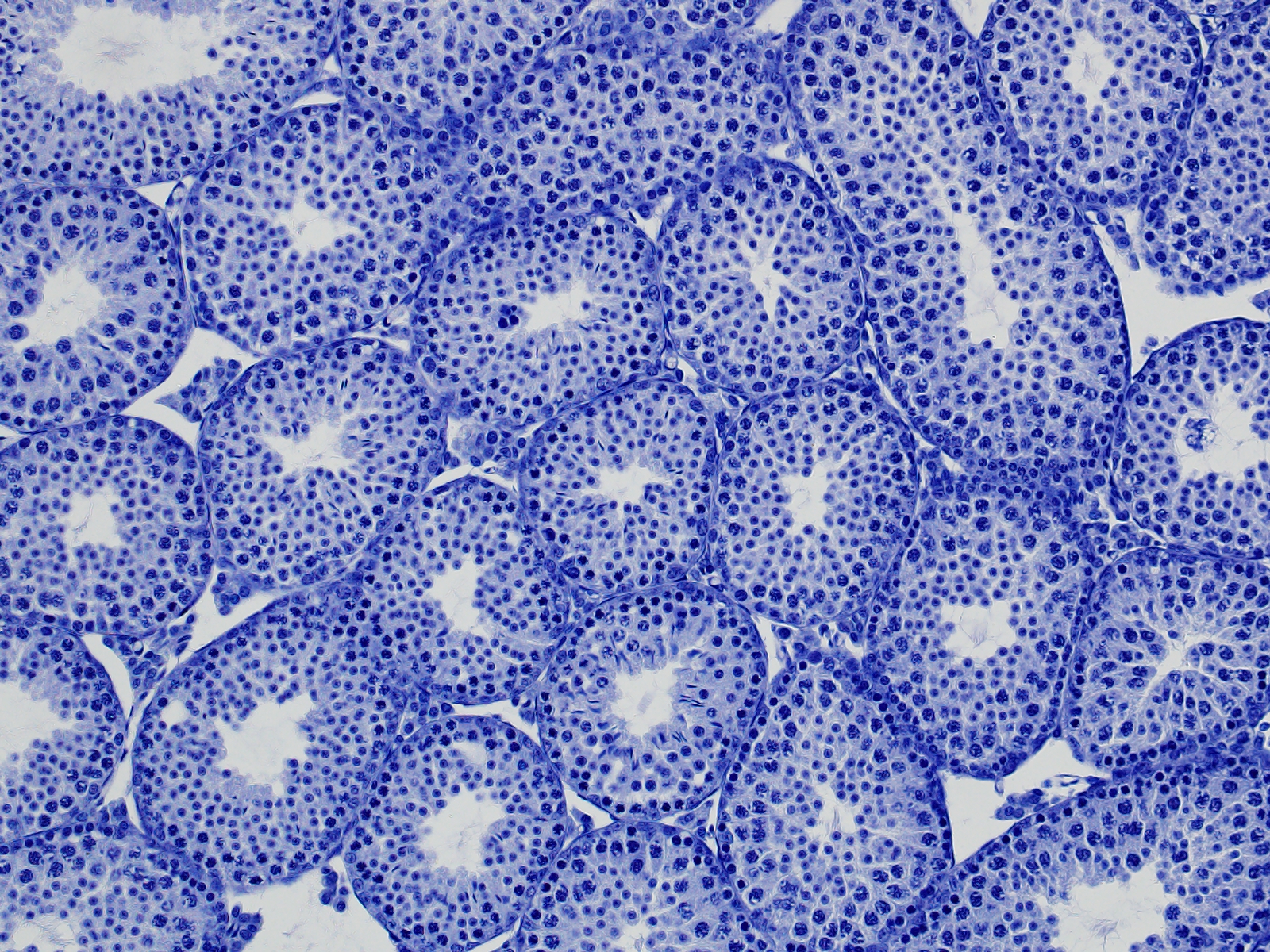

Supplement: Figure 2—figure supplement 2—source data 1. [file elife-83129-fig2-figsupp2-data1.zip › Figure supplement S4-source data 11/H Staining/hs-pd28-WT-20x.jpg]

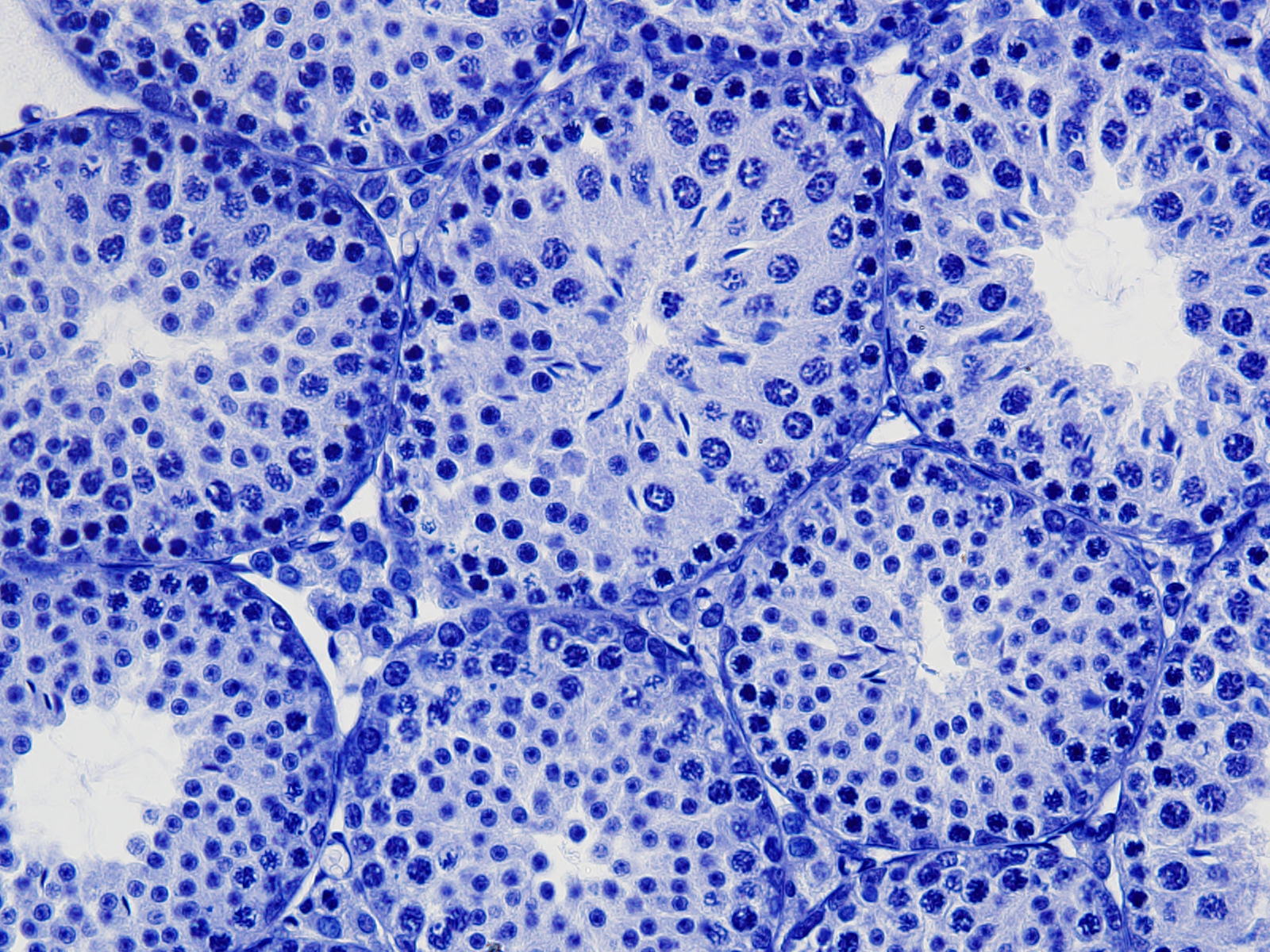

Supplement: Figure 2—figure supplement 2—source data 1. [file elife-83129-fig2-figsupp2-data1.zip › Figure supplement S4-source data 11/H Staining/hs-pd28-WT-40x-1.jpg]

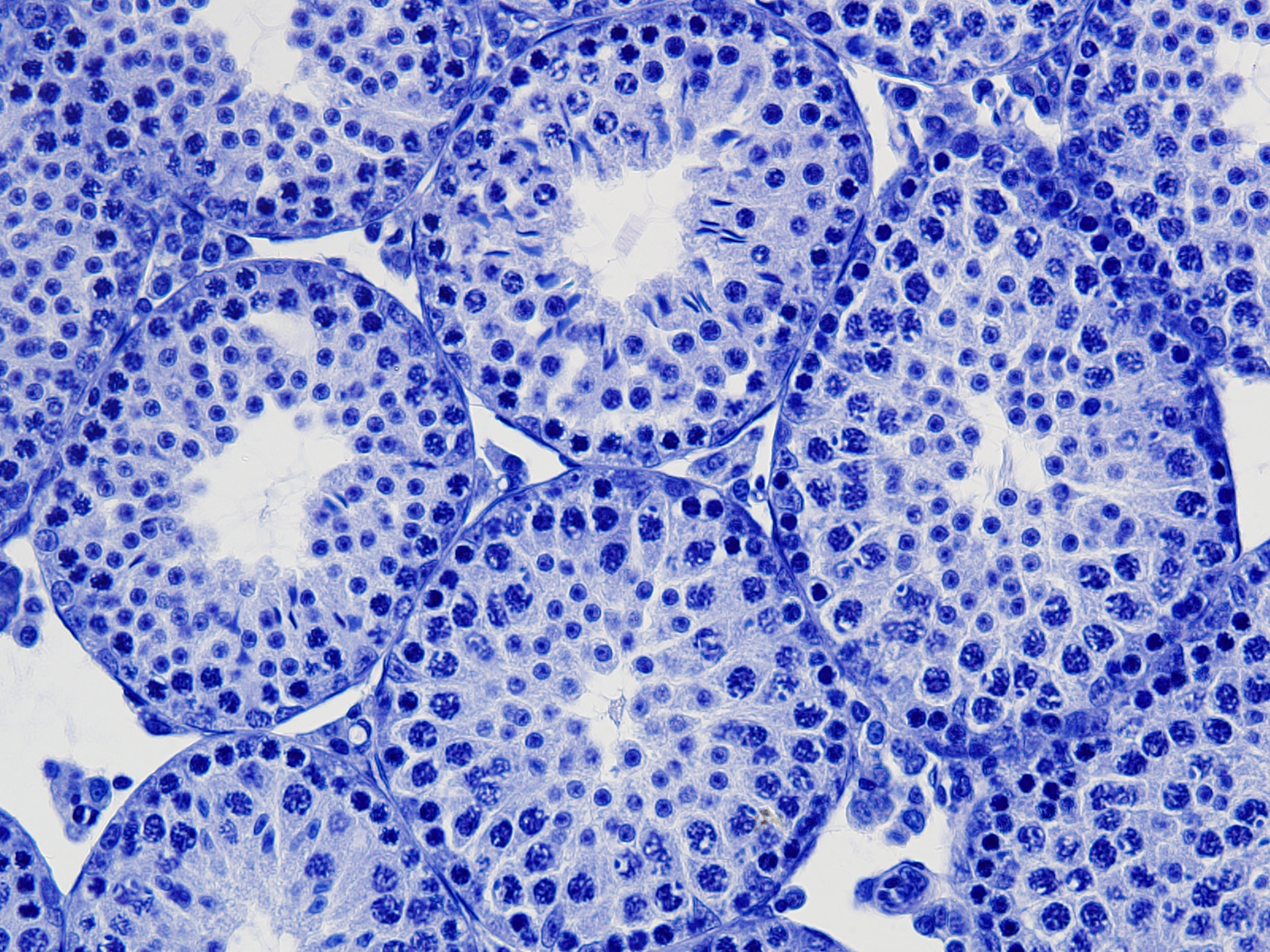

Supplement: Figure 2—figure supplement 2—source data 1. [file elife-83129-fig2-figsupp2-data1.zip › Figure supplement S4-source data 11/H Staining/hs-pd28-WT-40x.jpg]

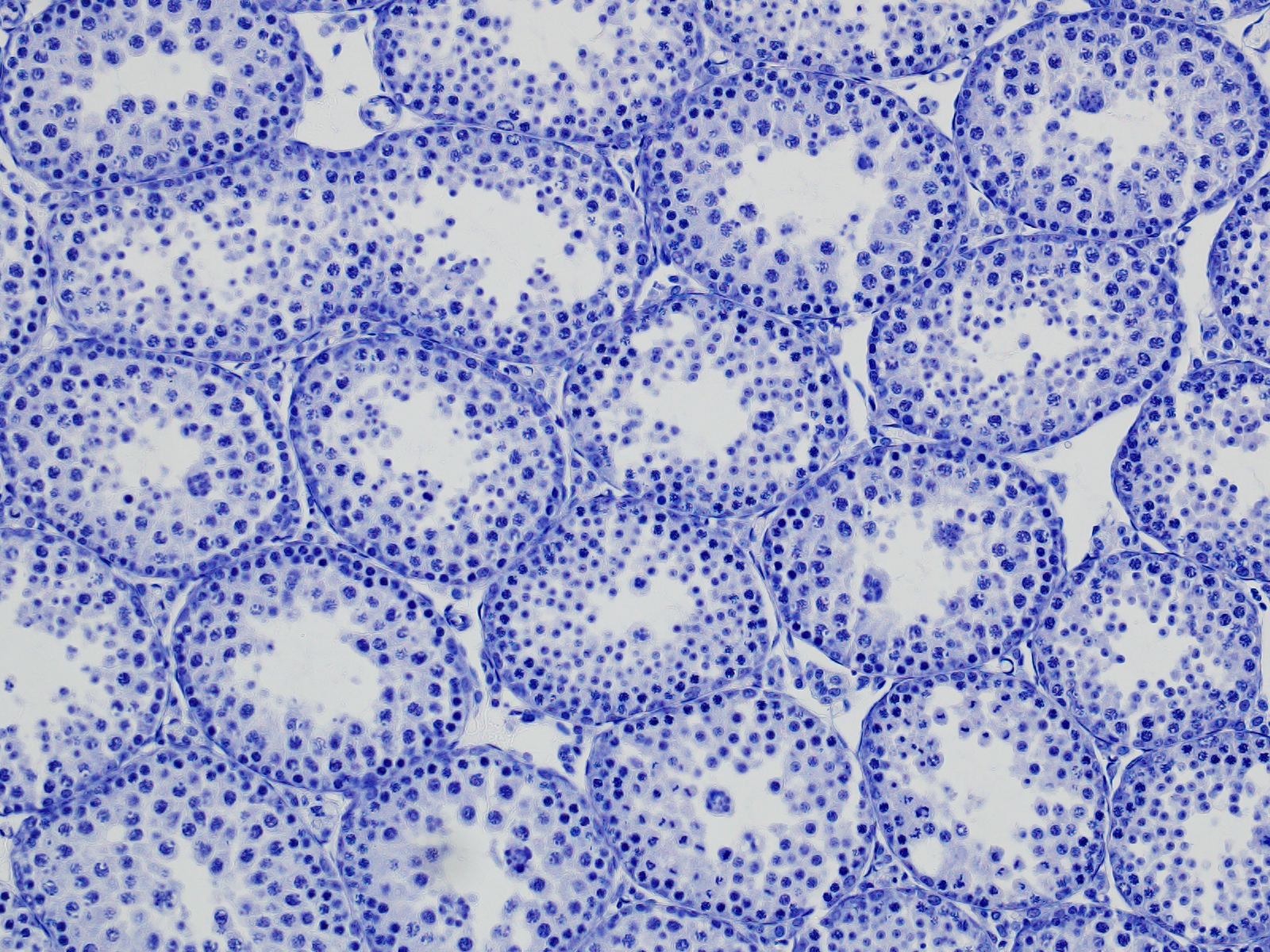

Supplement: Figure 2—figure supplement 2—source data 1. [file elife-83129-fig2-figsupp2-data1.zip › Figure supplement S4-source data 11/H Staining/hs-pd28-ko-20x.jpg]

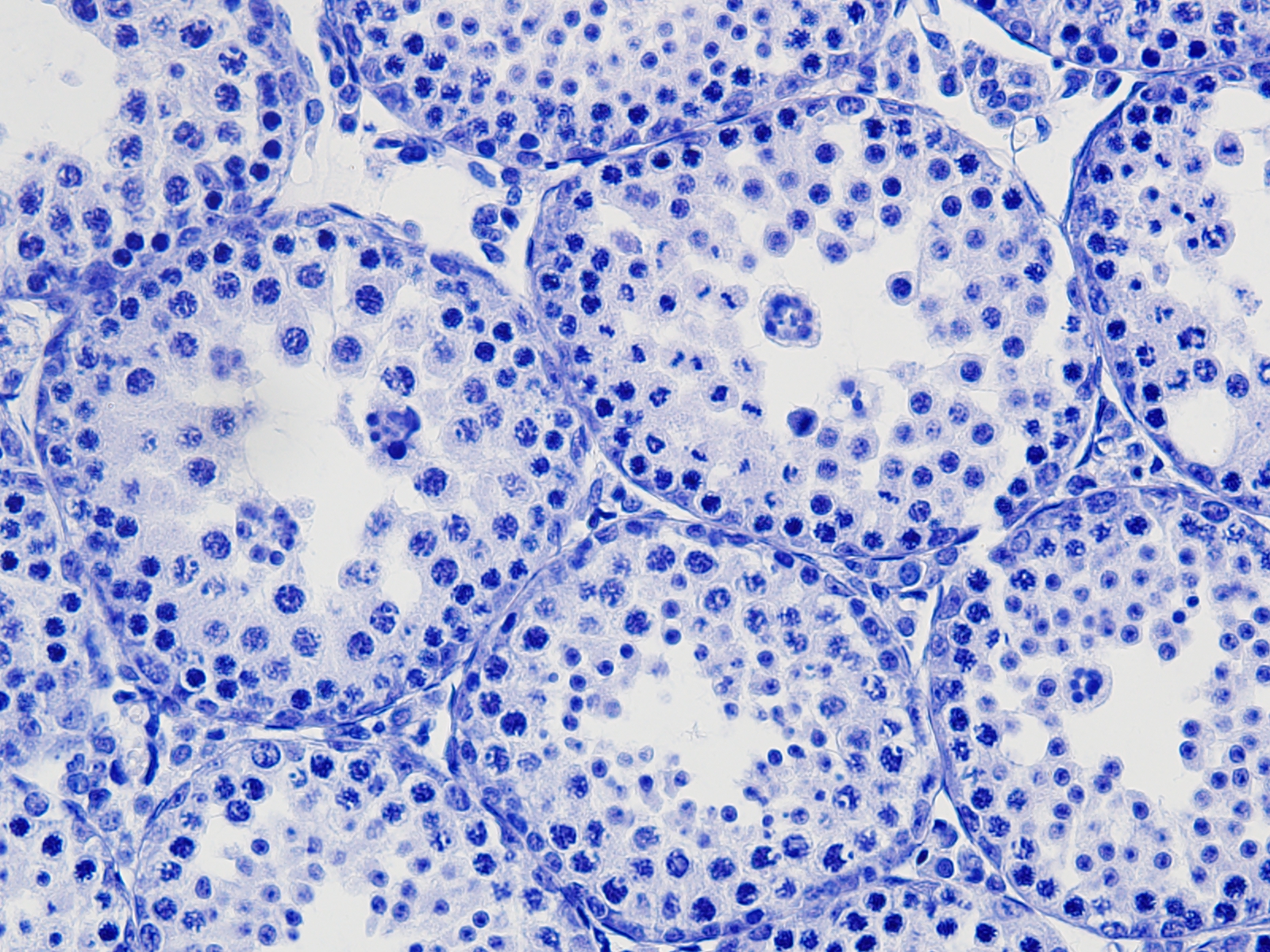

Supplement: Figure 2—figure supplement 2—source data 1. [file elife-83129-fig2-figsupp2-data1.zip › Figure supplement S4-source data 11/H Staining/hs-pd28-ko-40x-1.jpg]

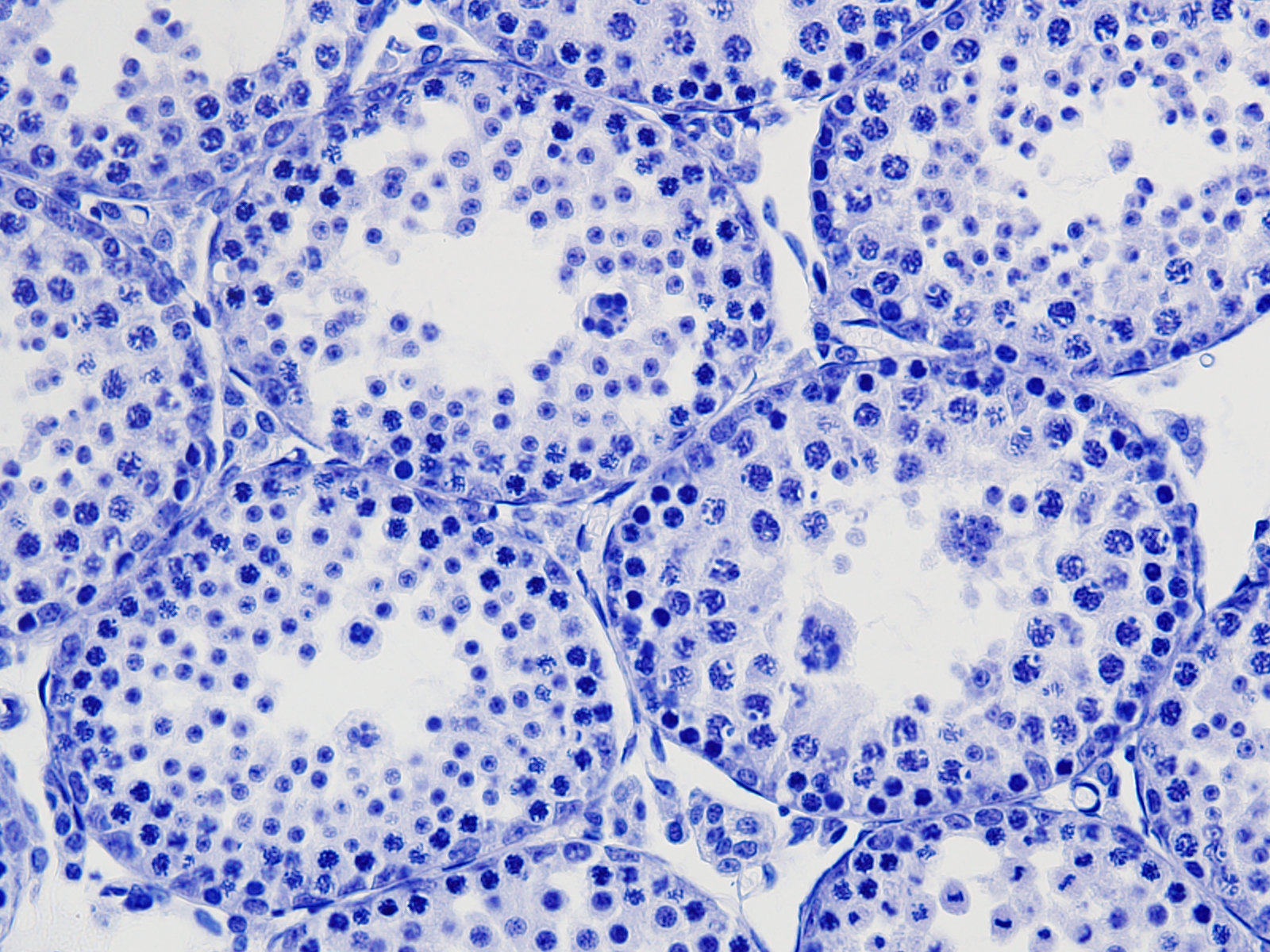

Supplement: Figure 2—figure supplement 2—source data 1. [file elife-83129-fig2-figsupp2-data1.zip › Figure supplement S4-source data 11/H Staining/hs-pd28-ko-40x.jpg]

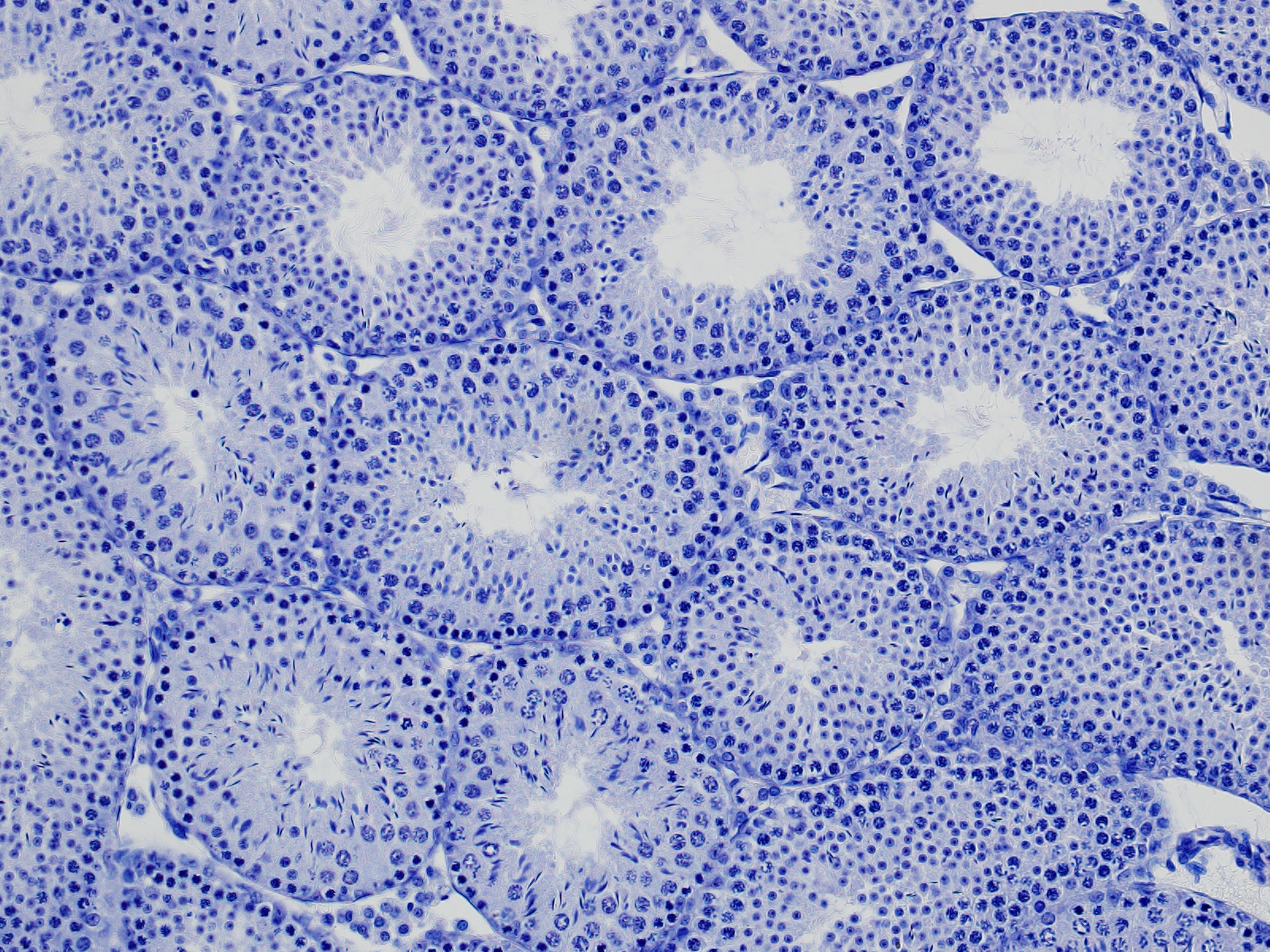

Supplement: Figure 2—figure supplement 2—source data 1. [file elife-83129-fig2-figsupp2-data1.zip › Figure supplement S4-source data 11/H Staining/hs-pd35-WT-20x.jpg]

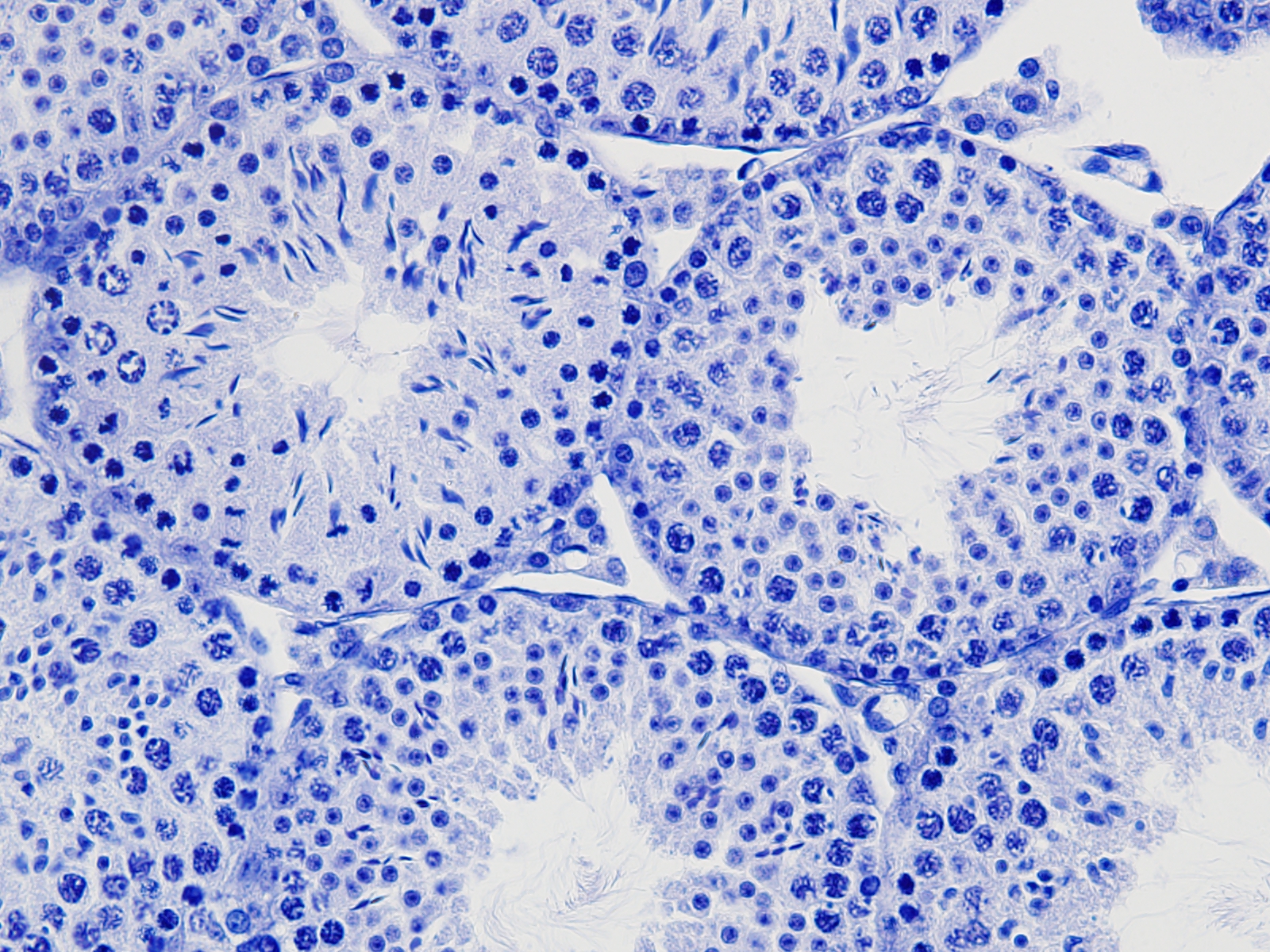

Supplement: Figure 2—figure supplement 2—source data 1. [file elife-83129-fig2-figsupp2-data1.zip › Figure supplement S4-source data 11/H Staining/hs-pd35-WT-40x-1.jpg]

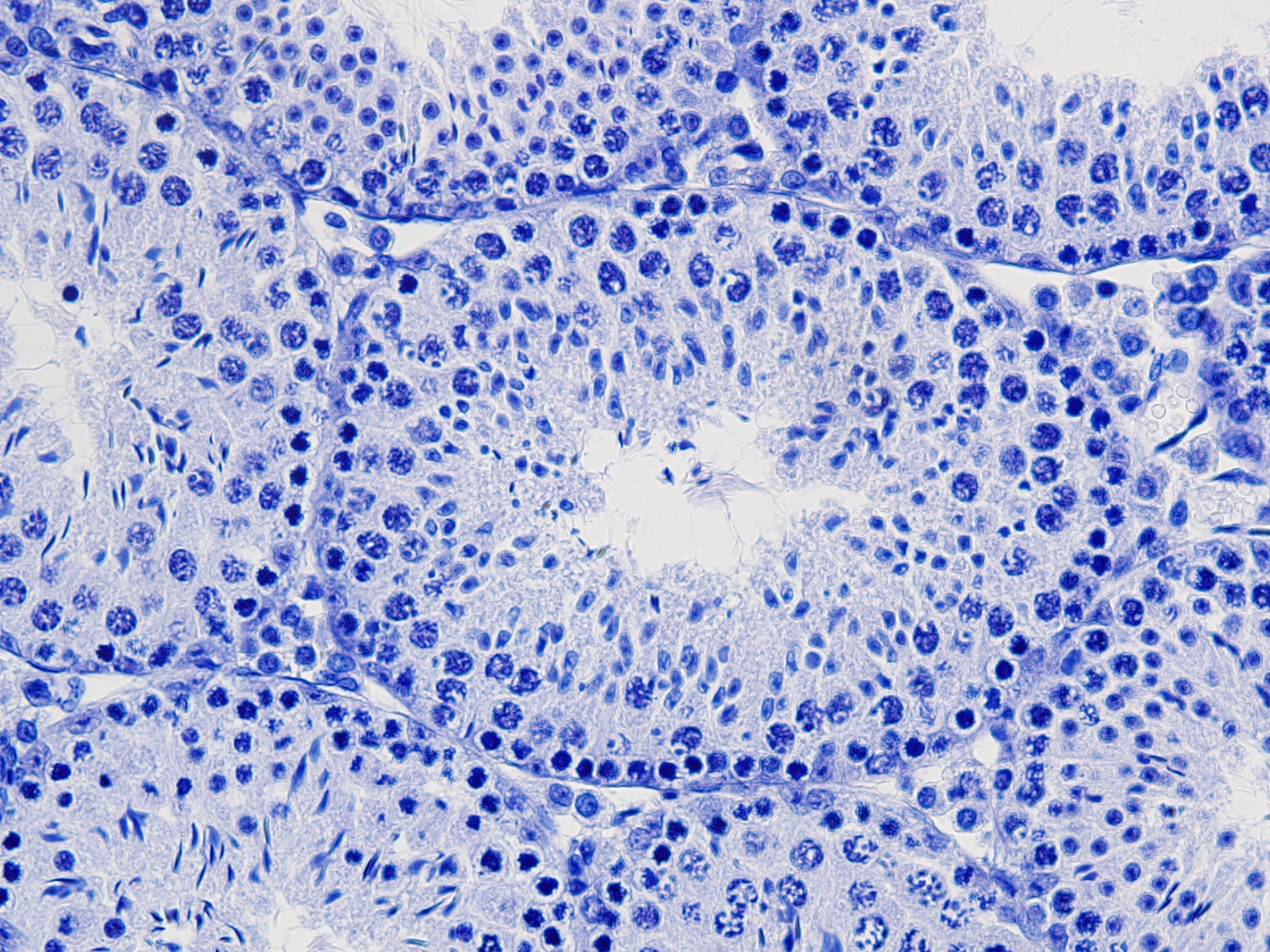

Supplement: Figure 2—figure supplement 2—source data 1. [file elife-83129-fig2-figsupp2-data1.zip › Figure supplement S4-source data 11/H Staining/hs-pd35-WT-40x.jpg]

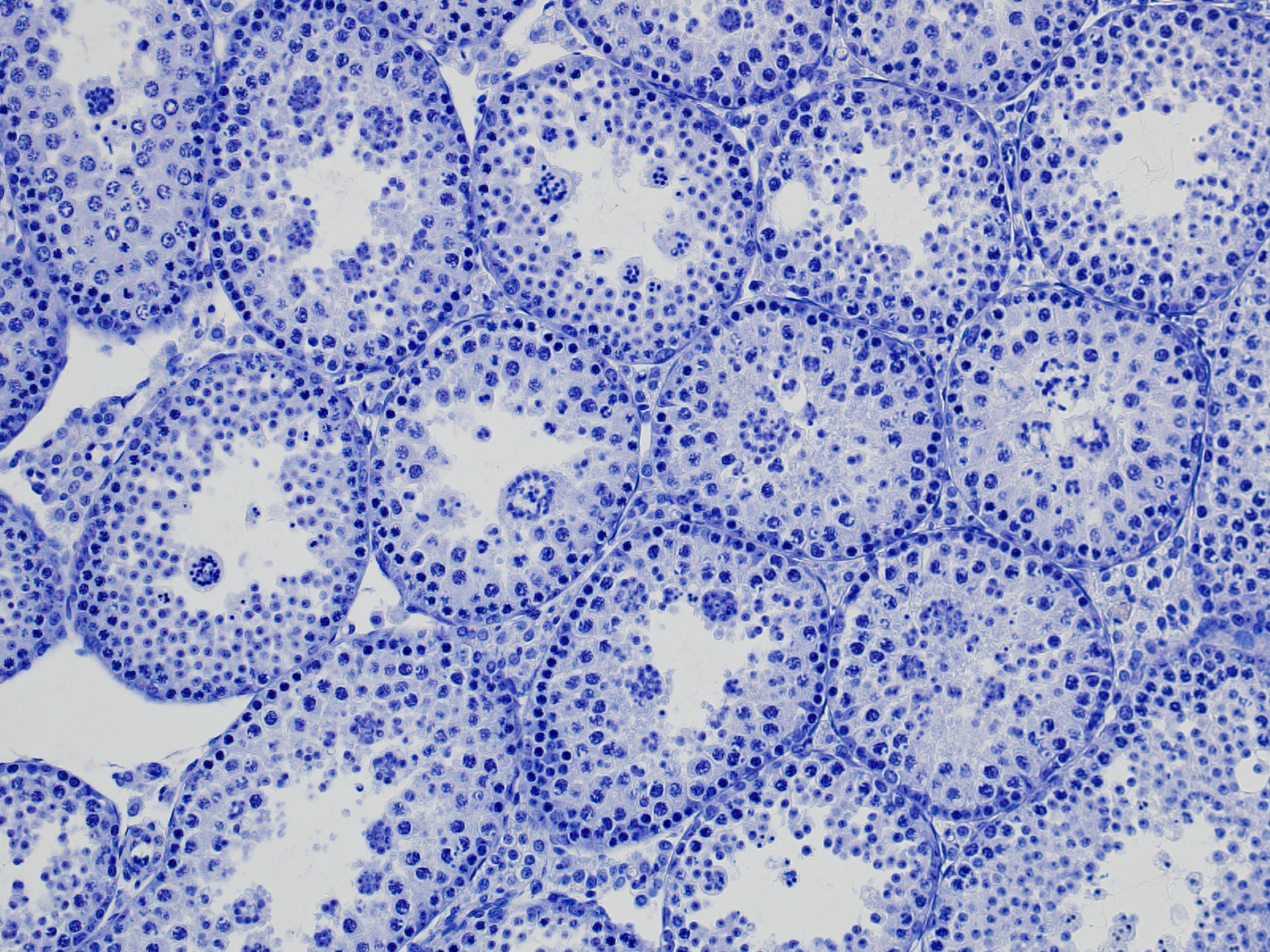

Supplement: Figure 2—figure supplement 2—source data 1. [file elife-83129-fig2-figsupp2-data1.zip › Figure supplement S4-source data 11/H Staining/hs-pd35-ko-20x.jpg]

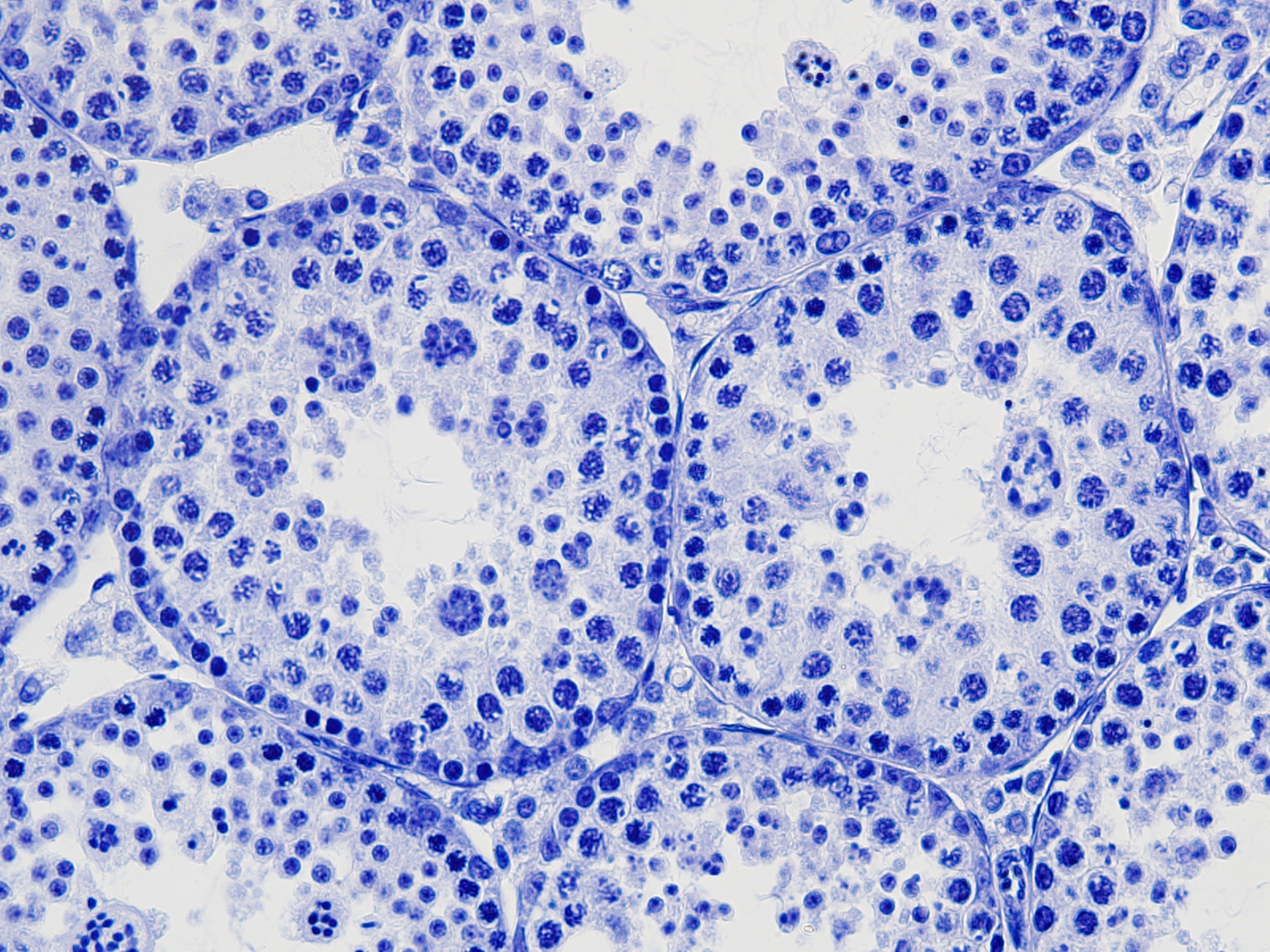

Supplement: Figure 2—figure supplement 2—source data 1. [file elife-83129-fig2-figsupp2-data1.zip › Figure supplement S4-source data 11/H Staining/hs-pd35-ko-40x-1.jpg]

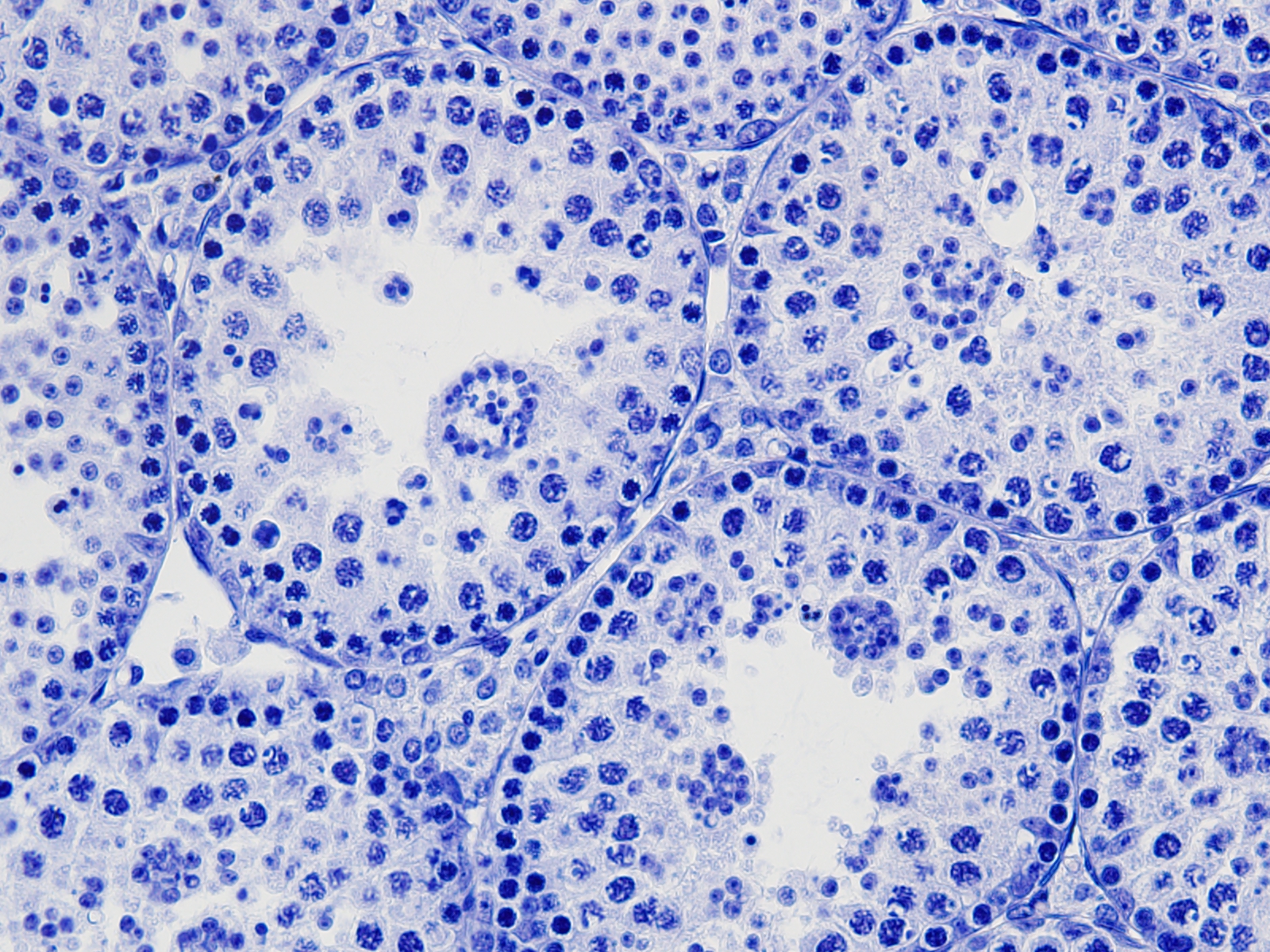

Supplement: Figure 2—figure supplement 2—source data 1. [file elife-83129-fig2-figsupp2-data1.zip › Figure supplement S4-source data 11/H Staining/hs-pd35-ko-40x.jpg]

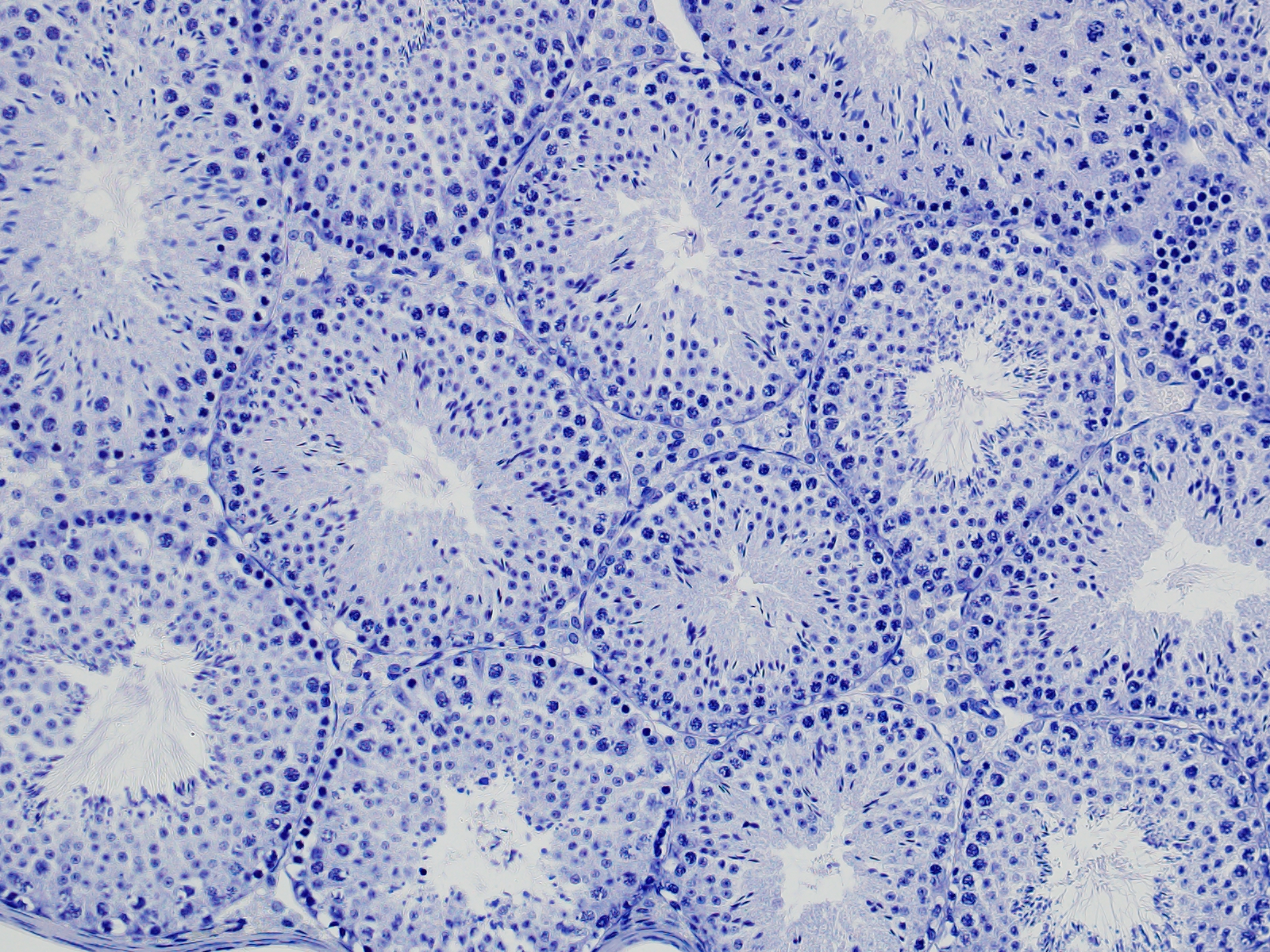

Supplement: Figure 2—figure supplement 2—source data 1. [file elife-83129-fig2-figsupp2-data1.zip › Figure supplement S4-source data 11/H Staining/hs-pd60-WT-20x.jpg]

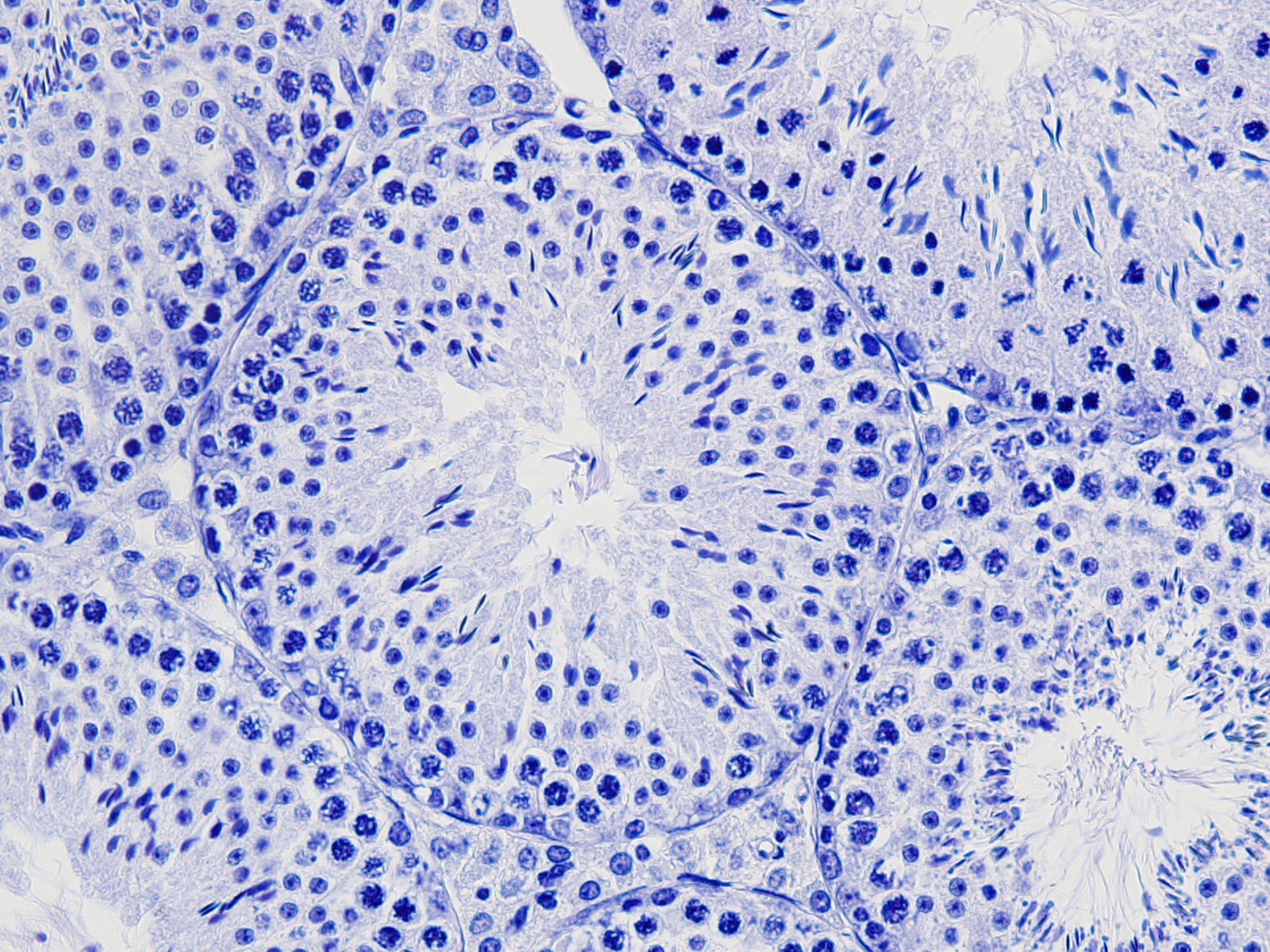

Supplement: Figure 2—figure supplement 2—source data 1. [file elife-83129-fig2-figsupp2-data1.zip › Figure supplement S4-source data 11/H Staining/hs-pd60-WT-40x-1.jpg]

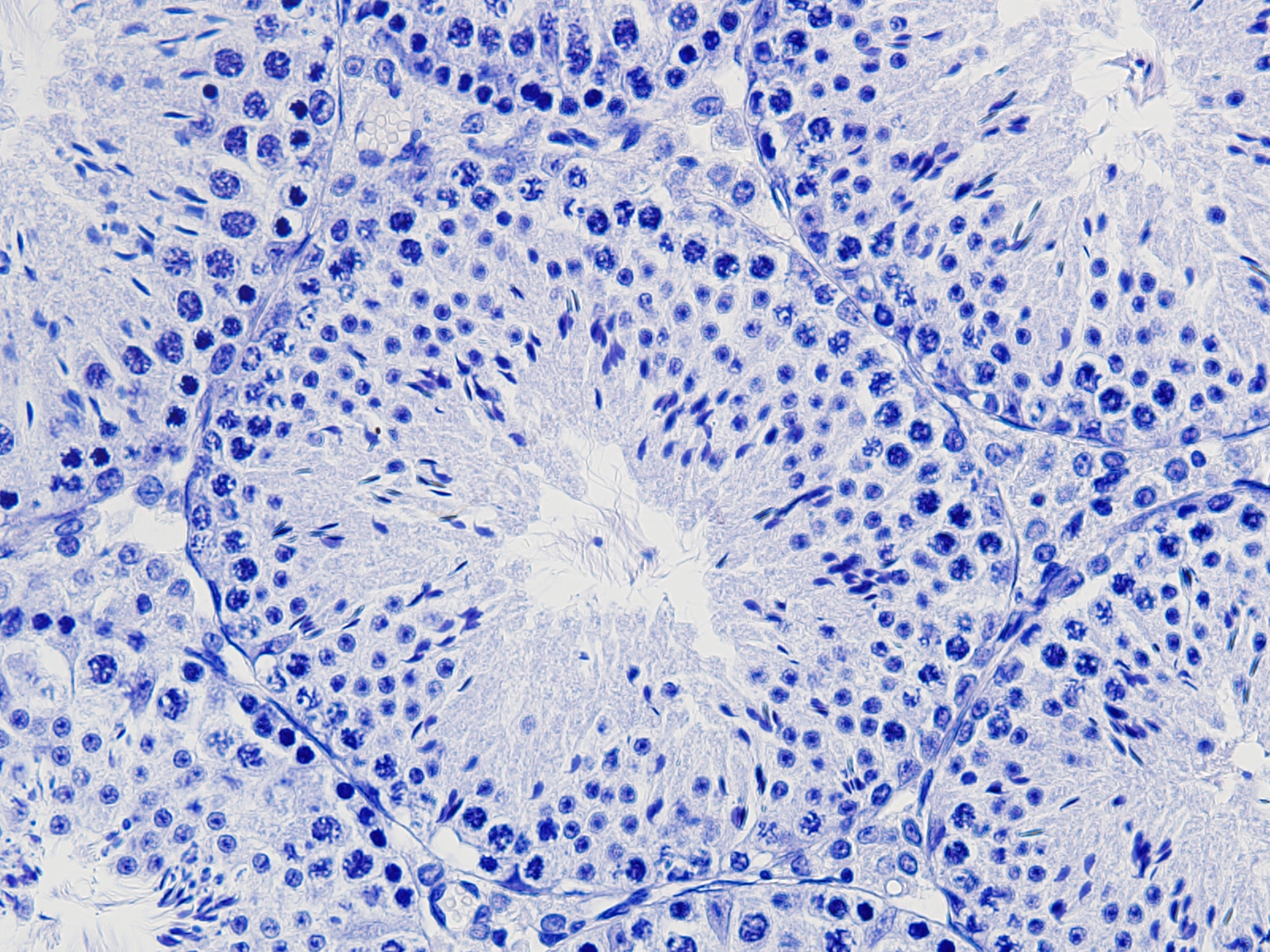

Supplement: Figure 2—figure supplement 2—source data 1. [file elife-83129-fig2-figsupp2-data1.zip › Figure supplement S4-source data 11/H Staining/hs-pd60-WT-40x.jpg]

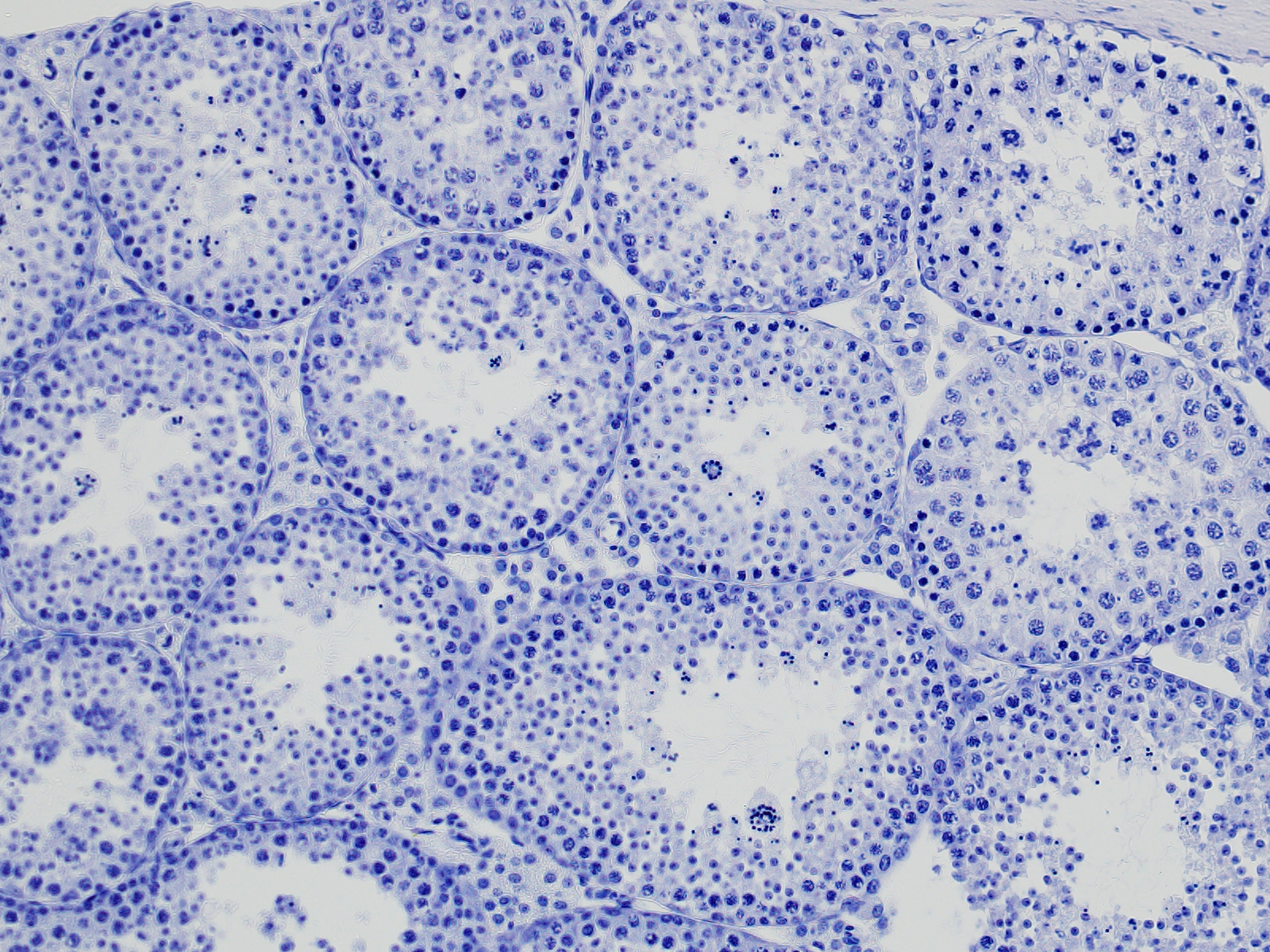

Supplement: Figure 2—figure supplement 2—source data 1. [file elife-83129-fig2-figsupp2-data1.zip › Figure supplement S4-source data 11/H Staining/hs-pd60-ko-20x.jpg]

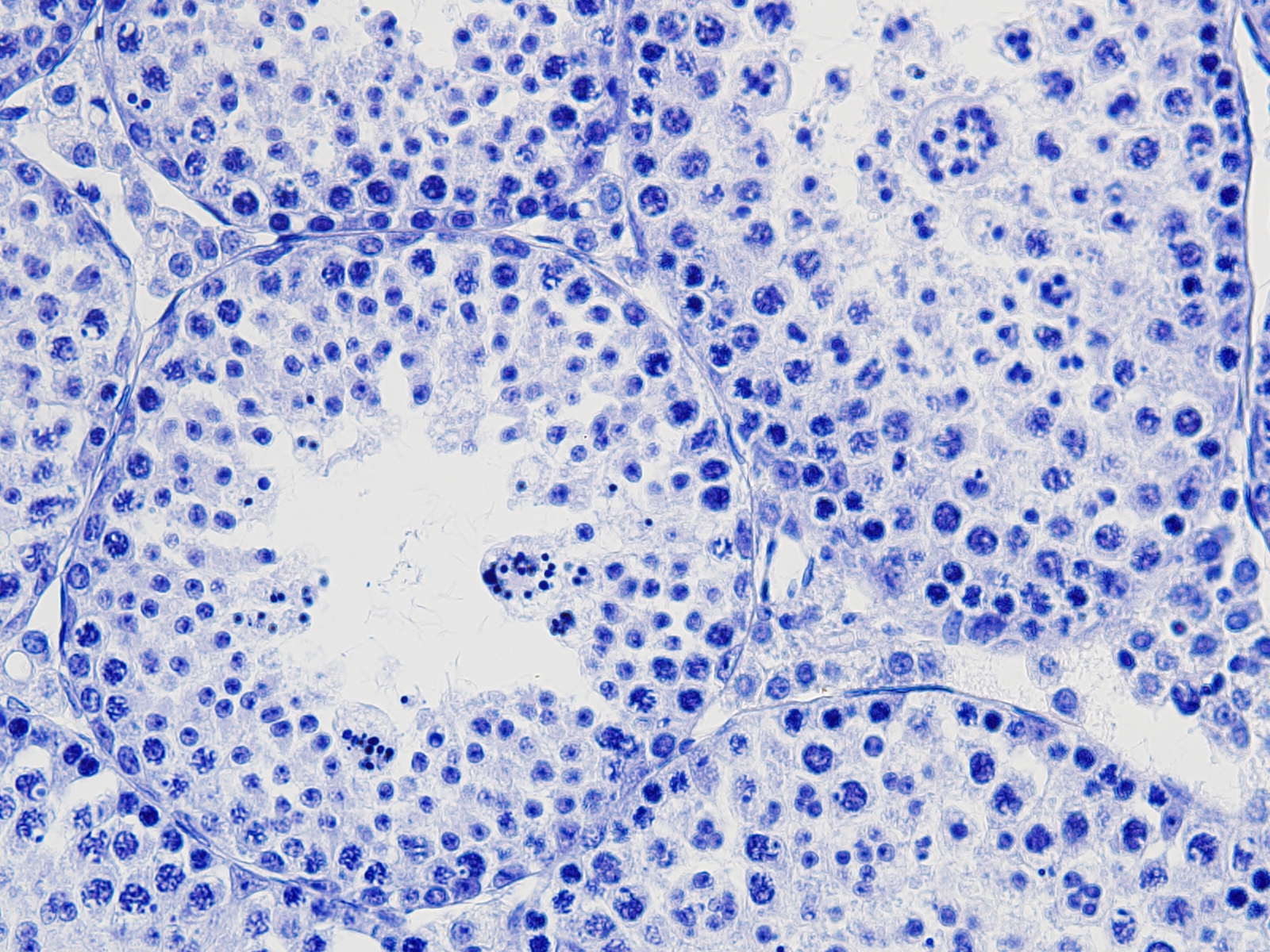

Supplement: Figure 2—figure supplement 2—source data 1. [file elife-83129-fig2-figsupp2-data1.zip › Figure supplement S4-source data 11/H Staining/hs-pd60-ko-40x-1.jpg]

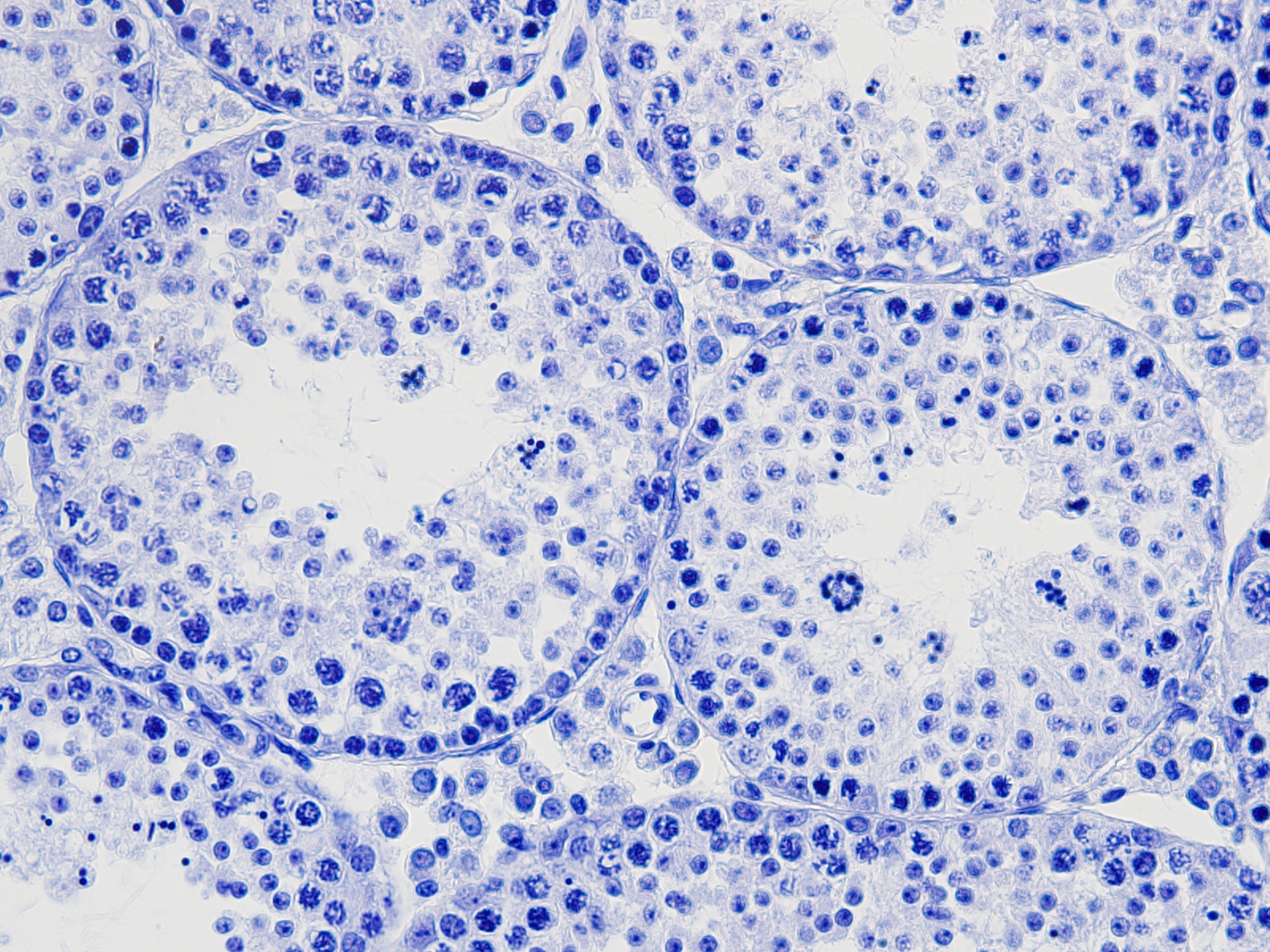

Supplement: Figure 2—figure supplement 2—source data 1. [file elife-83129-fig2-figsupp2-data1.zip › Figure supplement S4-source data 11/H Staining/hs-pd60-ko-40x.jpg]

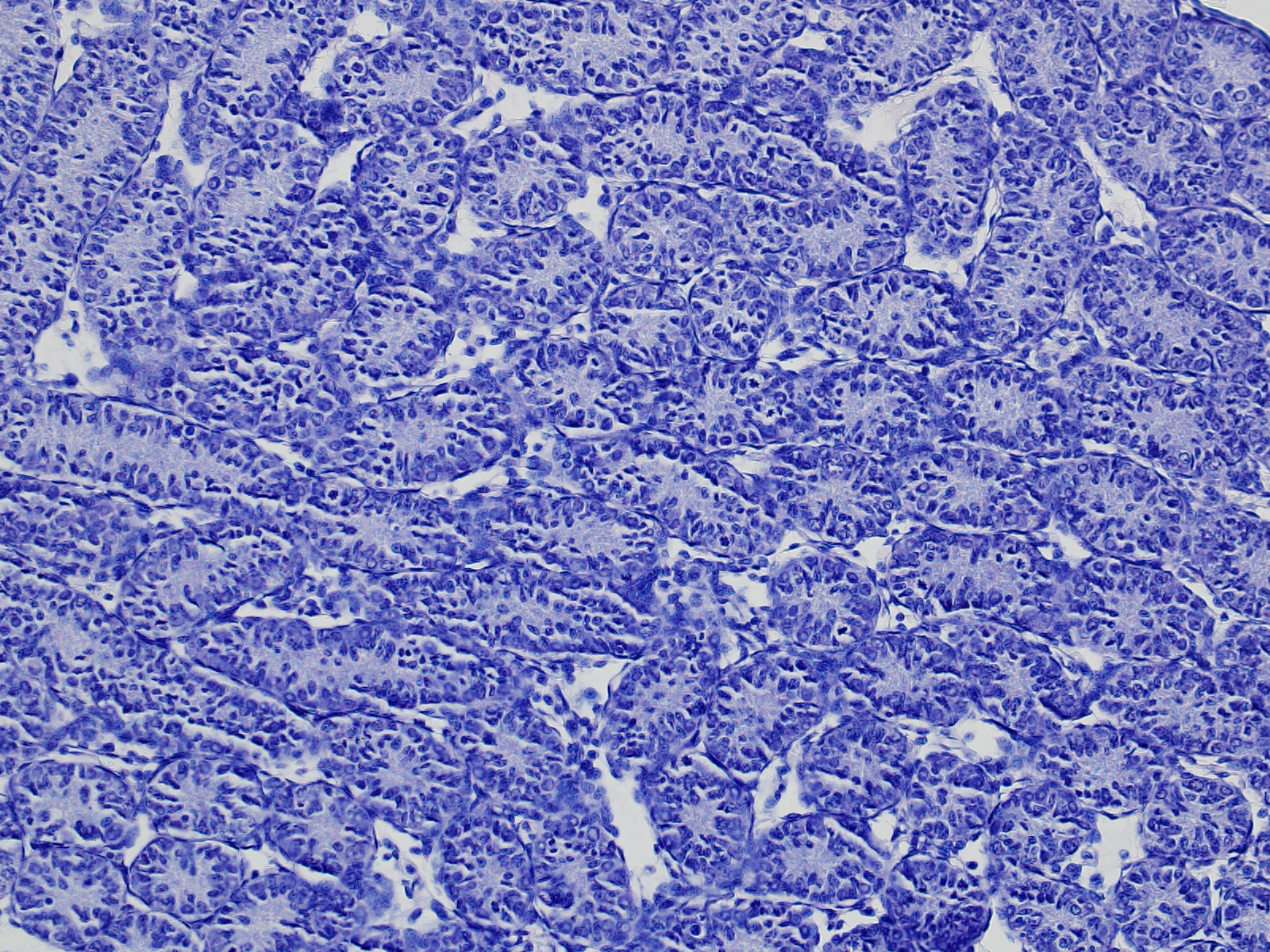

Supplement: Figure 2—figure supplement 2—source data 1. [file elife-83129-fig2-figsupp2-data1.zip › Figure supplement S4-source data 11/H Staining/hs-pd7-WT-20x.jpg]

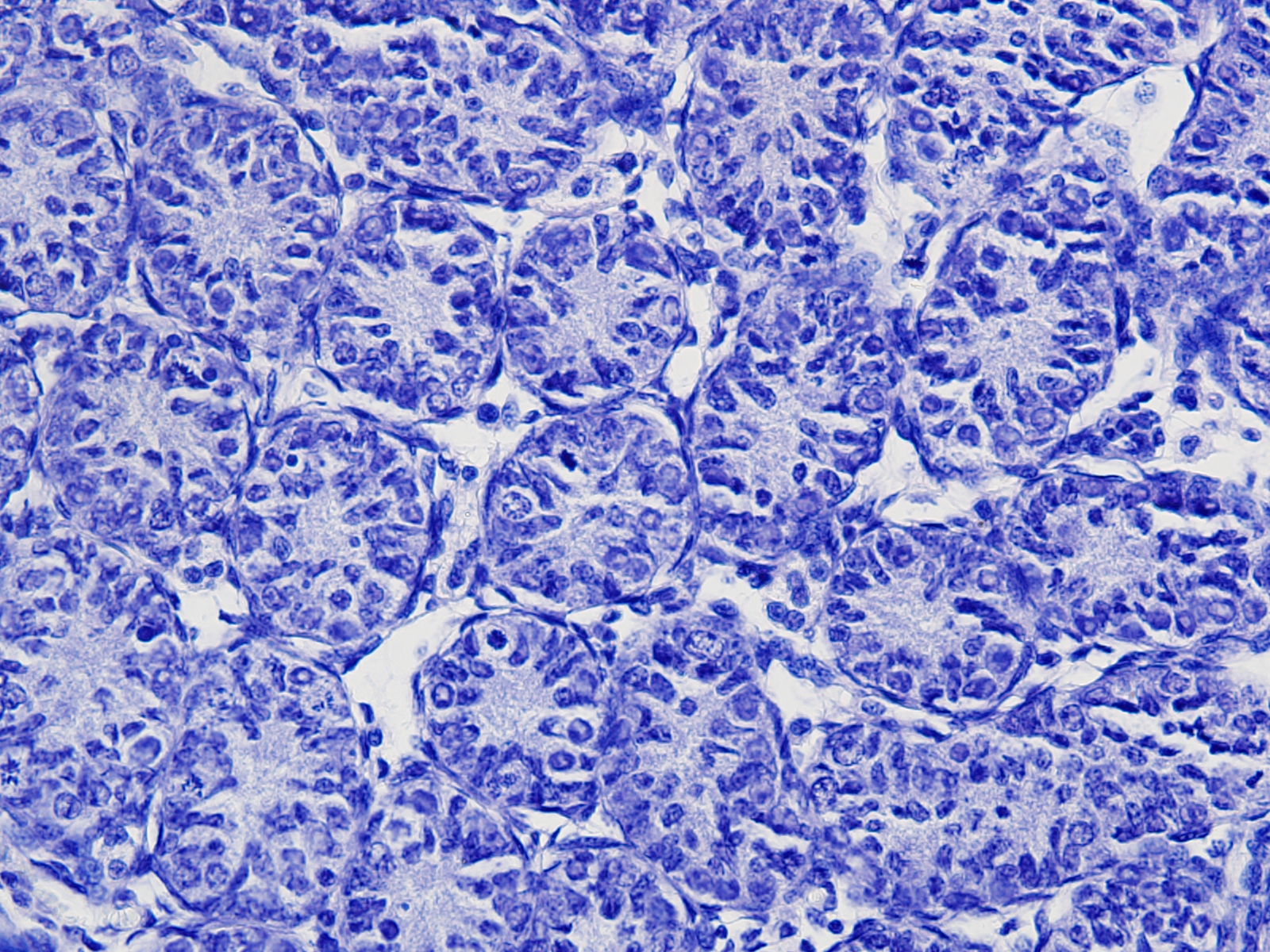

Supplement: Figure 2—figure supplement 2—source data 1. [file elife-83129-fig2-figsupp2-data1.zip › Figure supplement S4-source data 11/H Staining/hs-pd7-WT-40x 2.jpg]

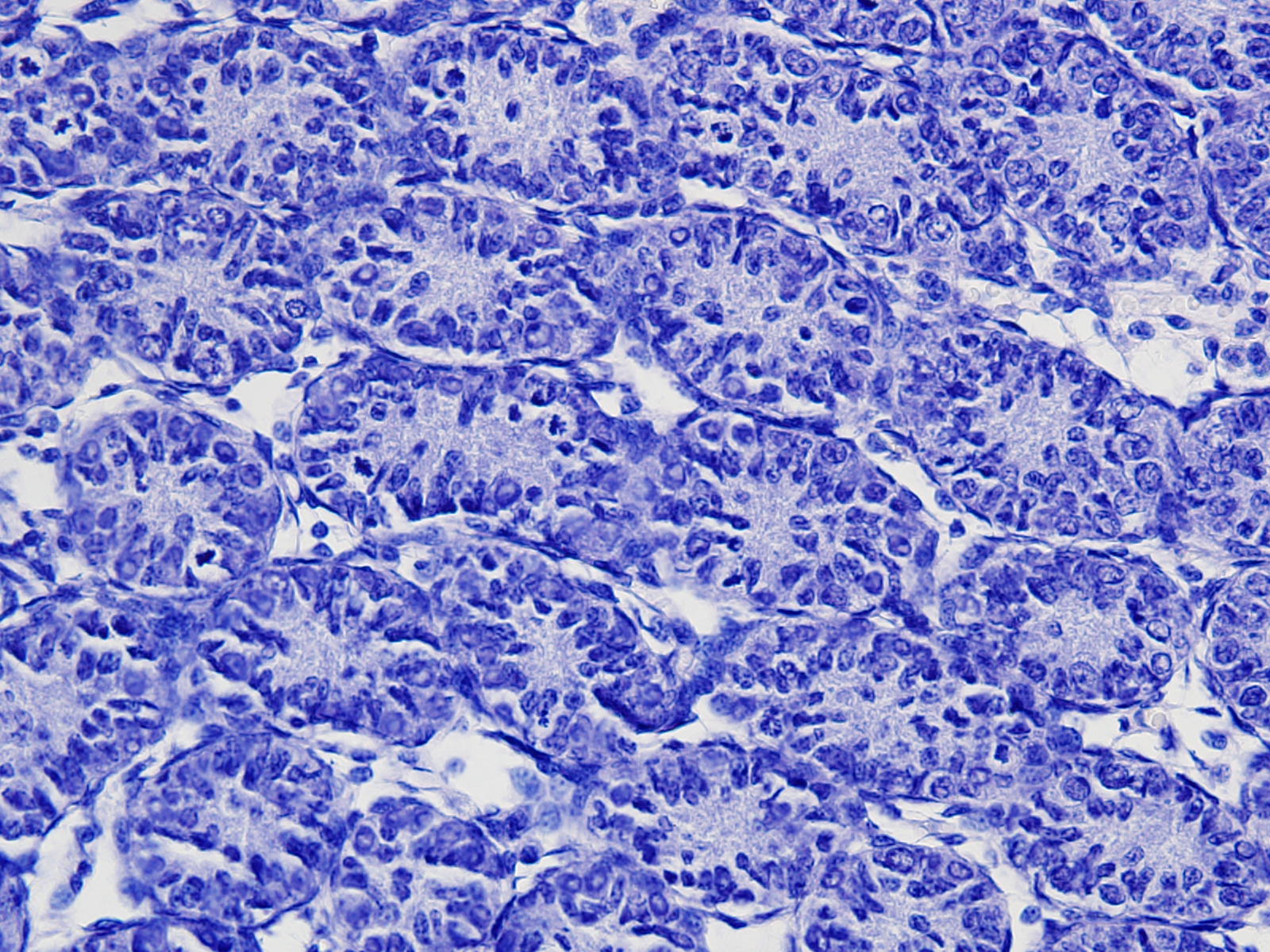

Supplement: Figure 2—figure supplement 2—source data 1. [file elife-83129-fig2-figsupp2-data1.zip › Figure supplement S4-source data 11/H Staining/hs-pd7-WT-40x-1.jpg]

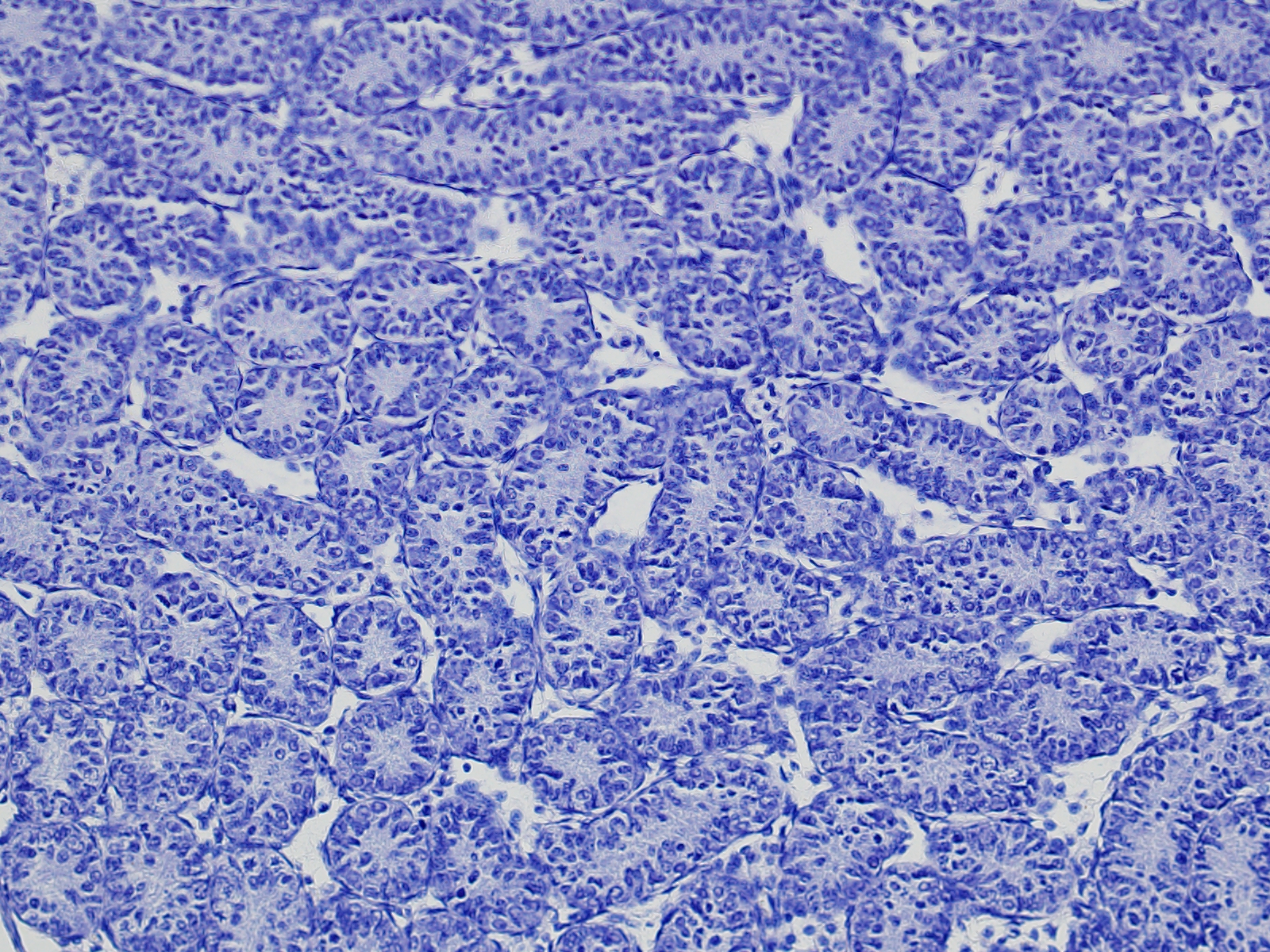

Supplement: Figure 2—figure supplement 2—source data 1. [file elife-83129-fig2-figsupp2-data1.zip › Figure supplement S4-source data 11/H Staining/hs-pd7-ko-20x.jpg]

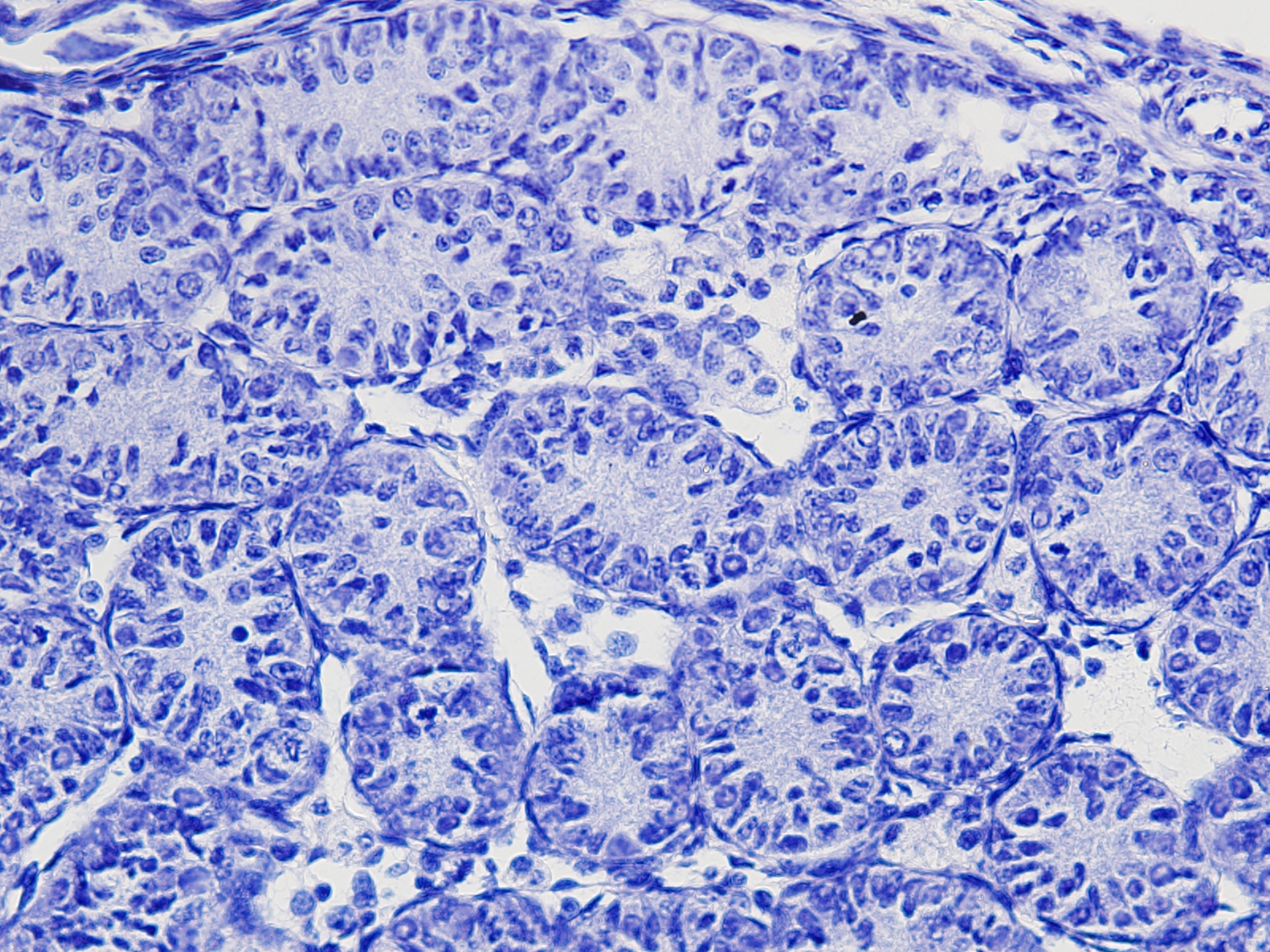

Supplement: Figure 2—figure supplement 2—source data 1. [file elife-83129-fig2-figsupp2-data1.zip › Figure supplement S4-source data 11/H Staining/hs-pd7-ko-40x-1.jpg]

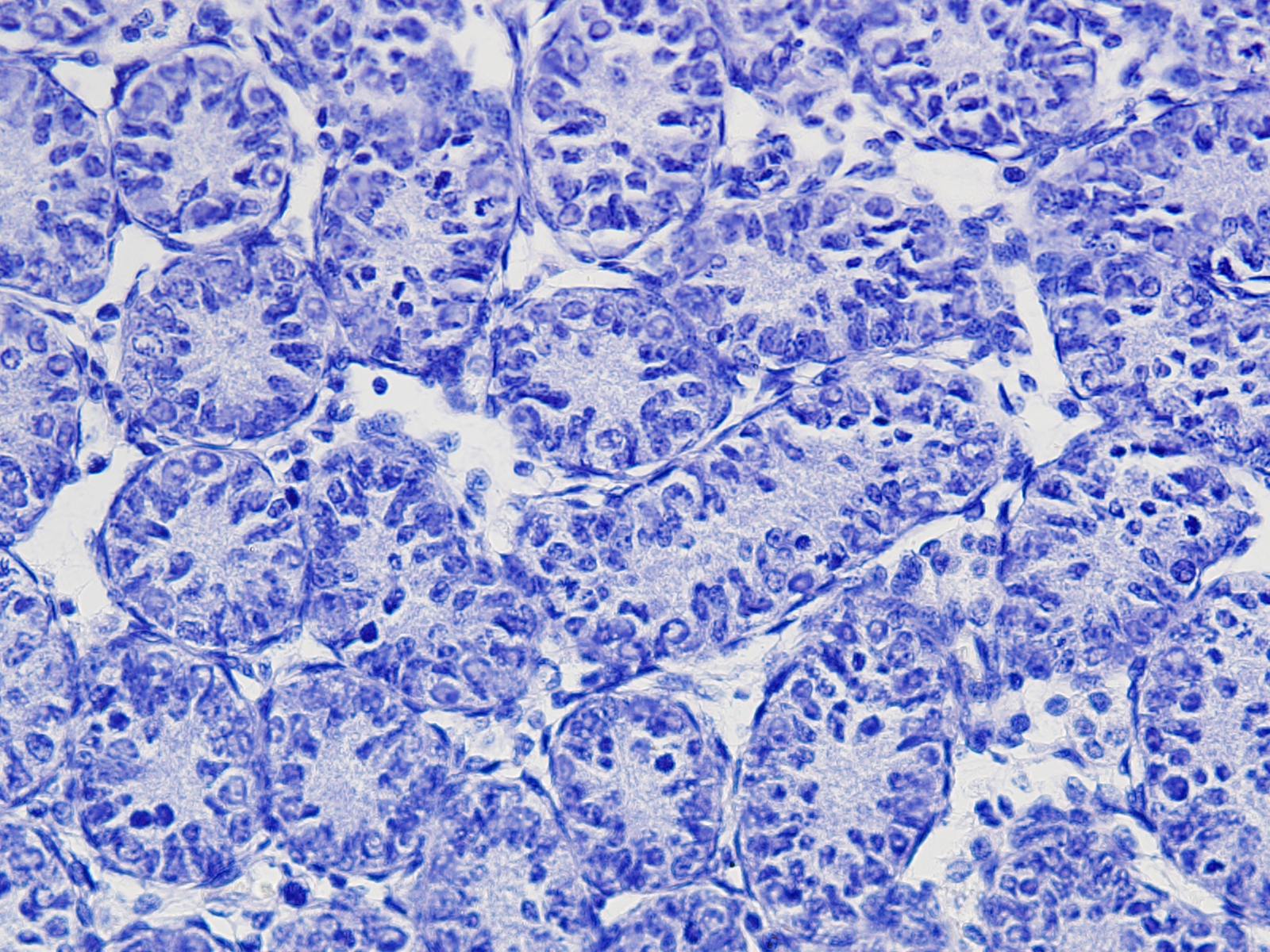

Supplement: Figure 2—figure supplement 2—source data 1. [file elife-83129-fig2-figsupp2-data1.zip › Figure supplement S4-source data 11/H Staining/hs-pd7-ko-40x.jpg]

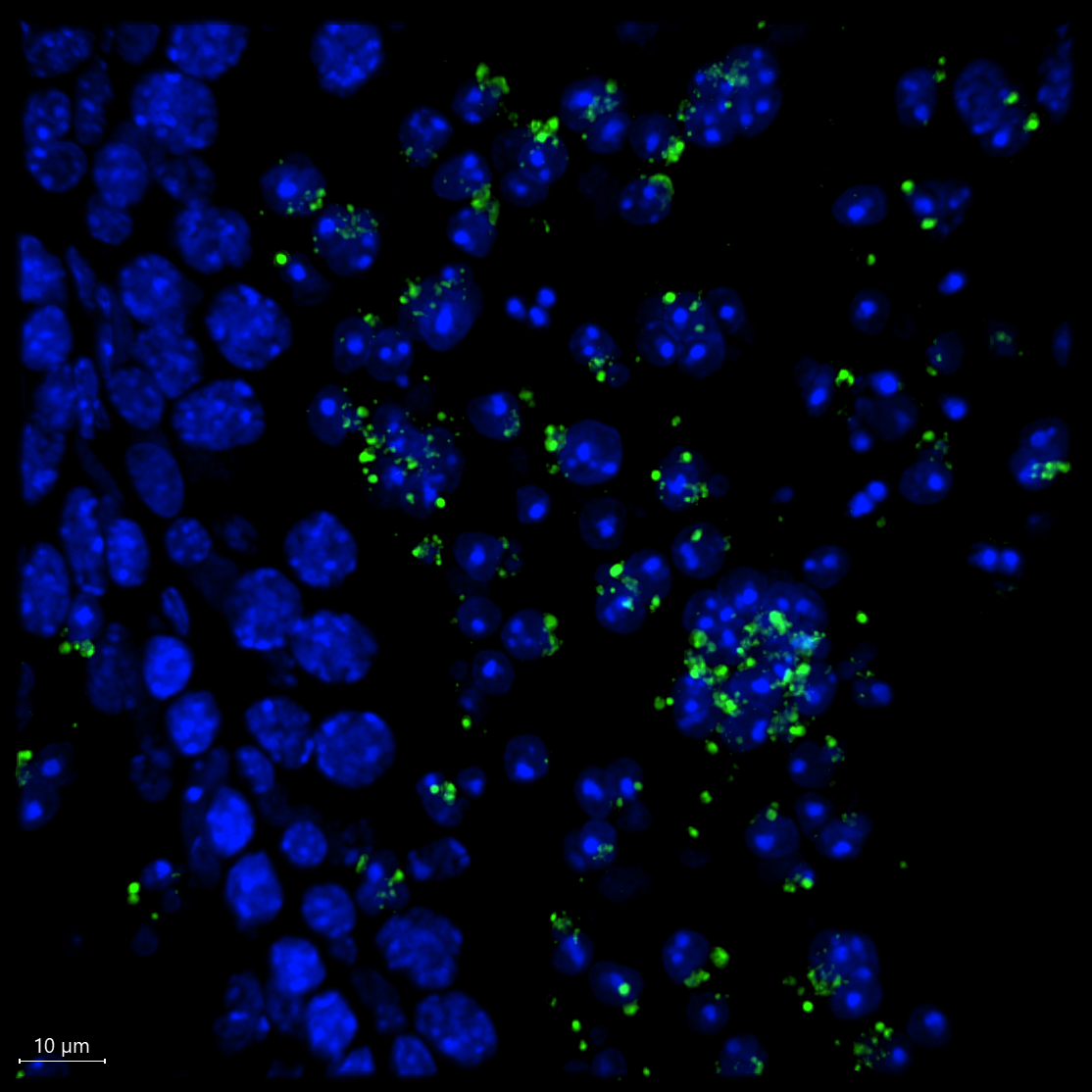

Supplement: Figure 3—source data 1. [file elife-83129-fig3-data1.zip › Figure3/Source data of Figure3A/pd60-KO_1.tif]

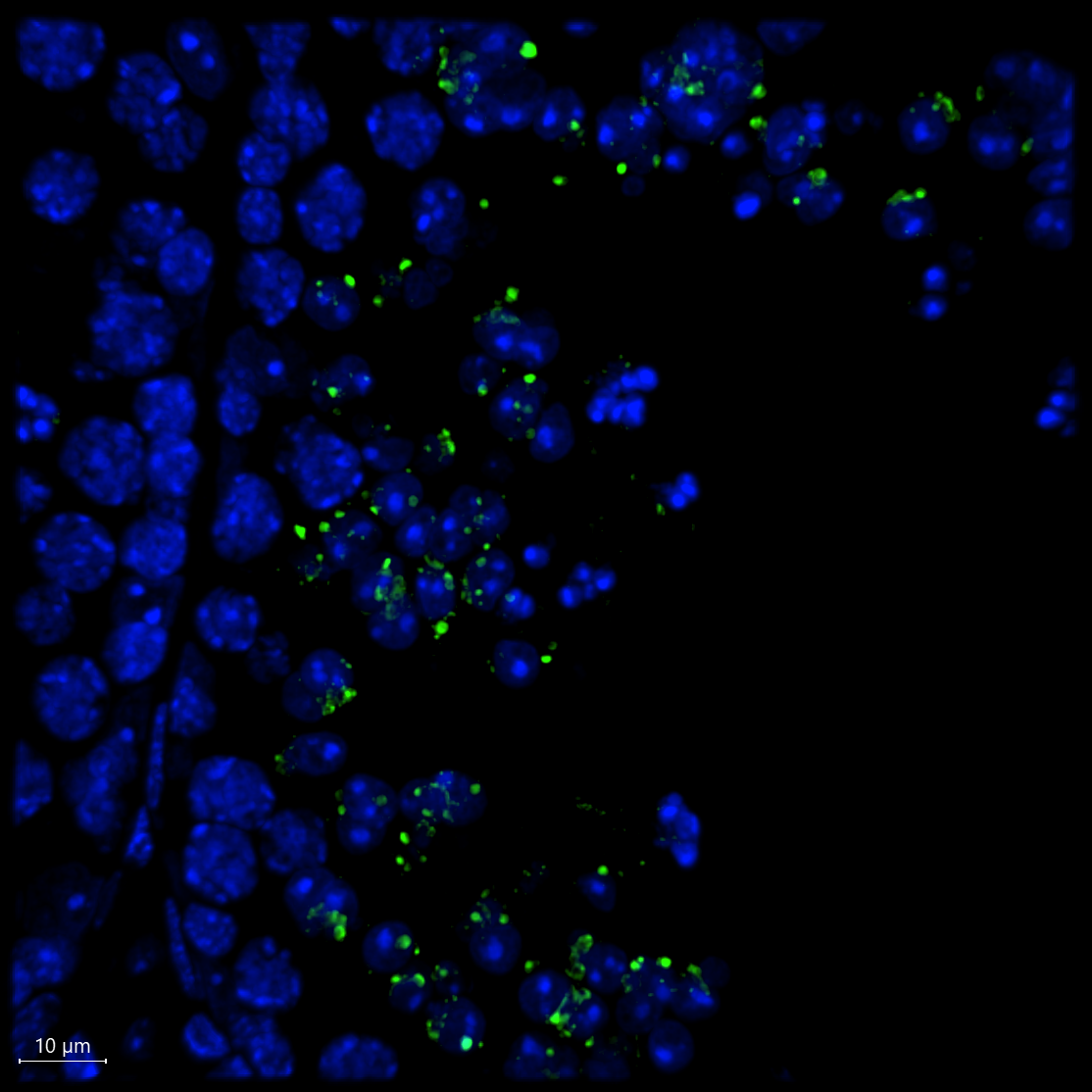

Supplement: Figure 3—source data 1. [file elife-83129-fig3-data1.zip › Figure3/Source data of Figure3A/pd60-KO_2.tif]

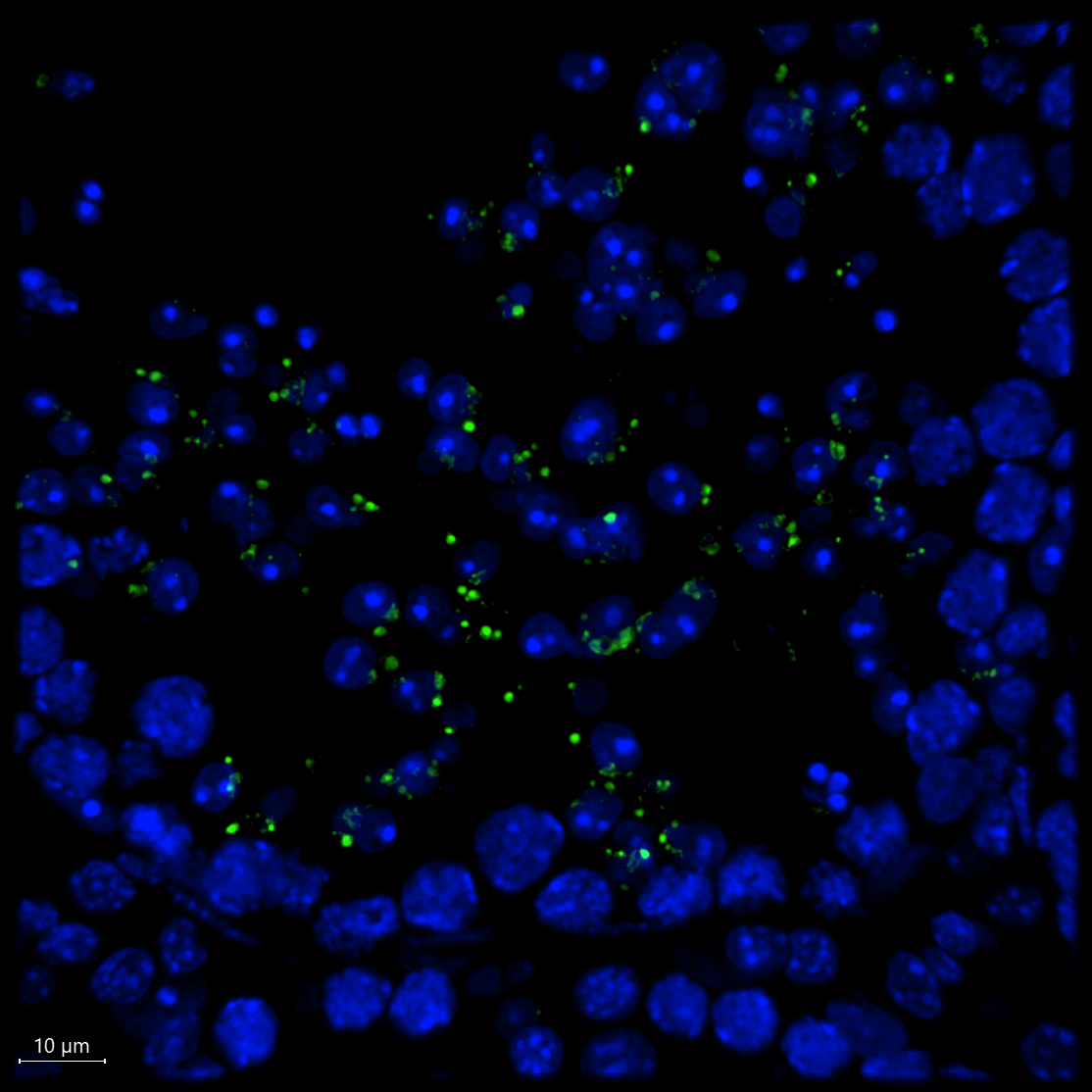

Supplement: Figure 3—source data 1. [file elife-83129-fig3-data1.zip › Figure3/Source data of Figure3A/pd60-KO_3.tif]

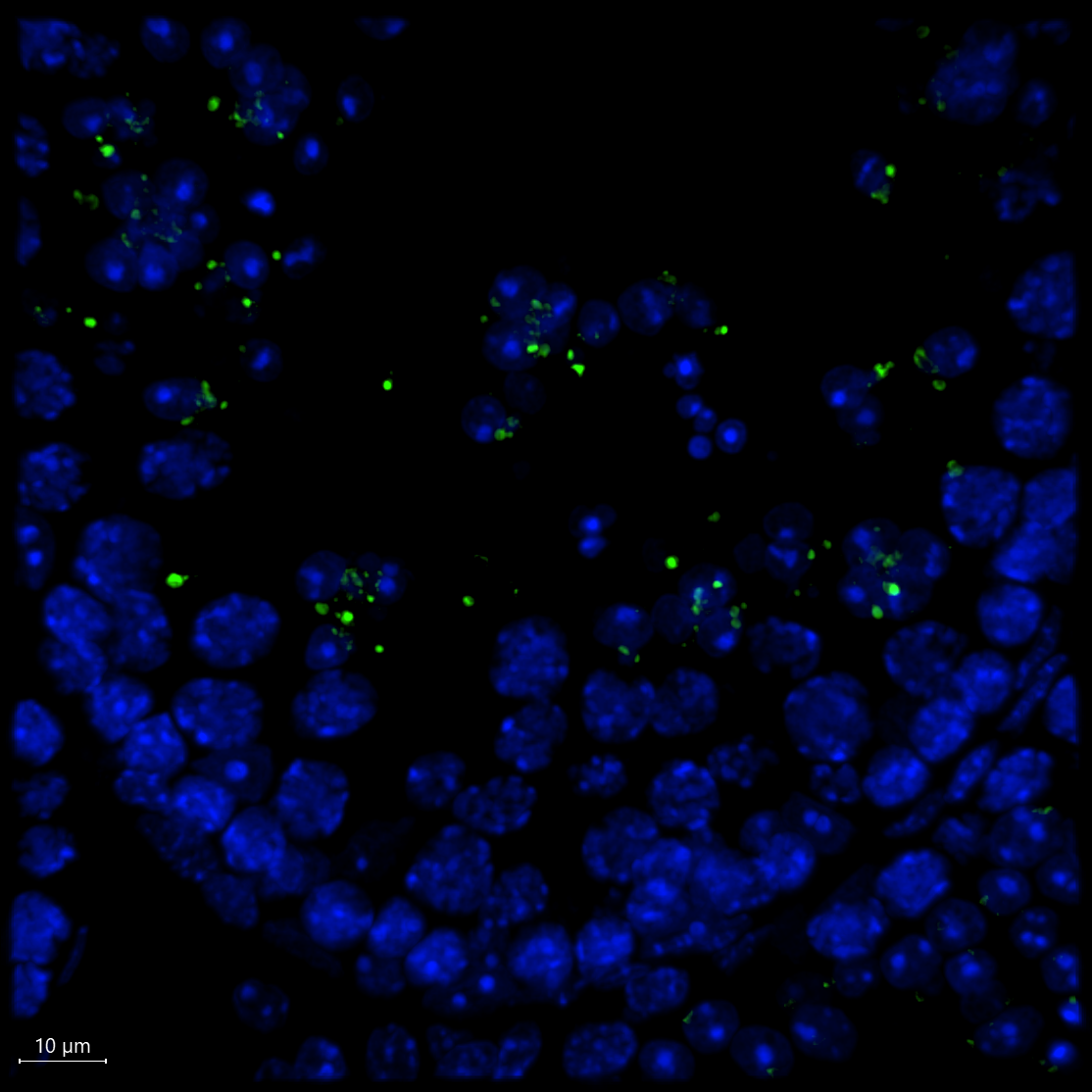

Supplement: Figure 3—source data 1. [file elife-83129-fig3-data1.zip › Figure3/Source data of Figure3A/pd60-KO_4.tif]

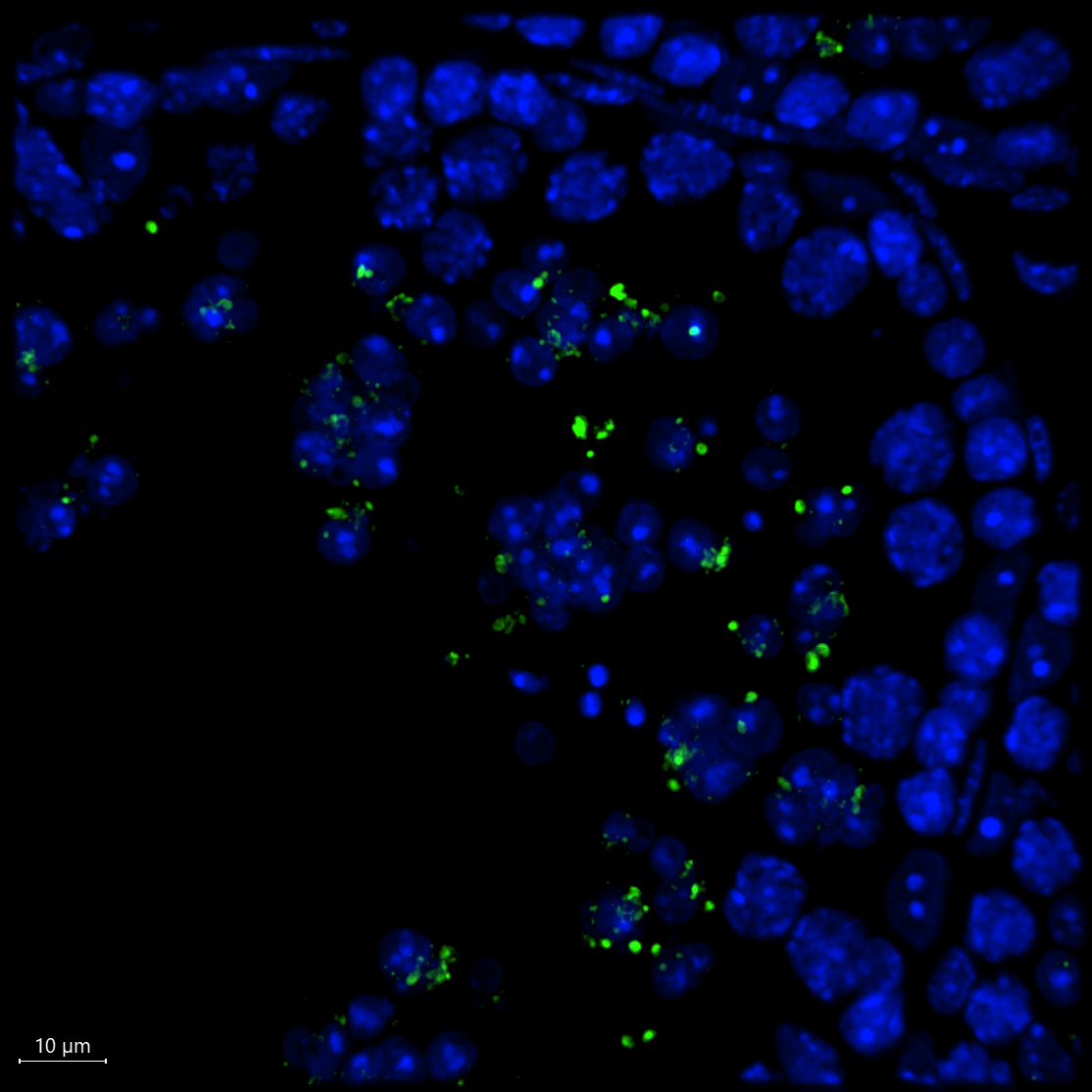

Supplement: Figure 3—source data 1. [file elife-83129-fig3-data1.zip › Figure3/Source data of Figure3A/pd60-KO_5.tif]

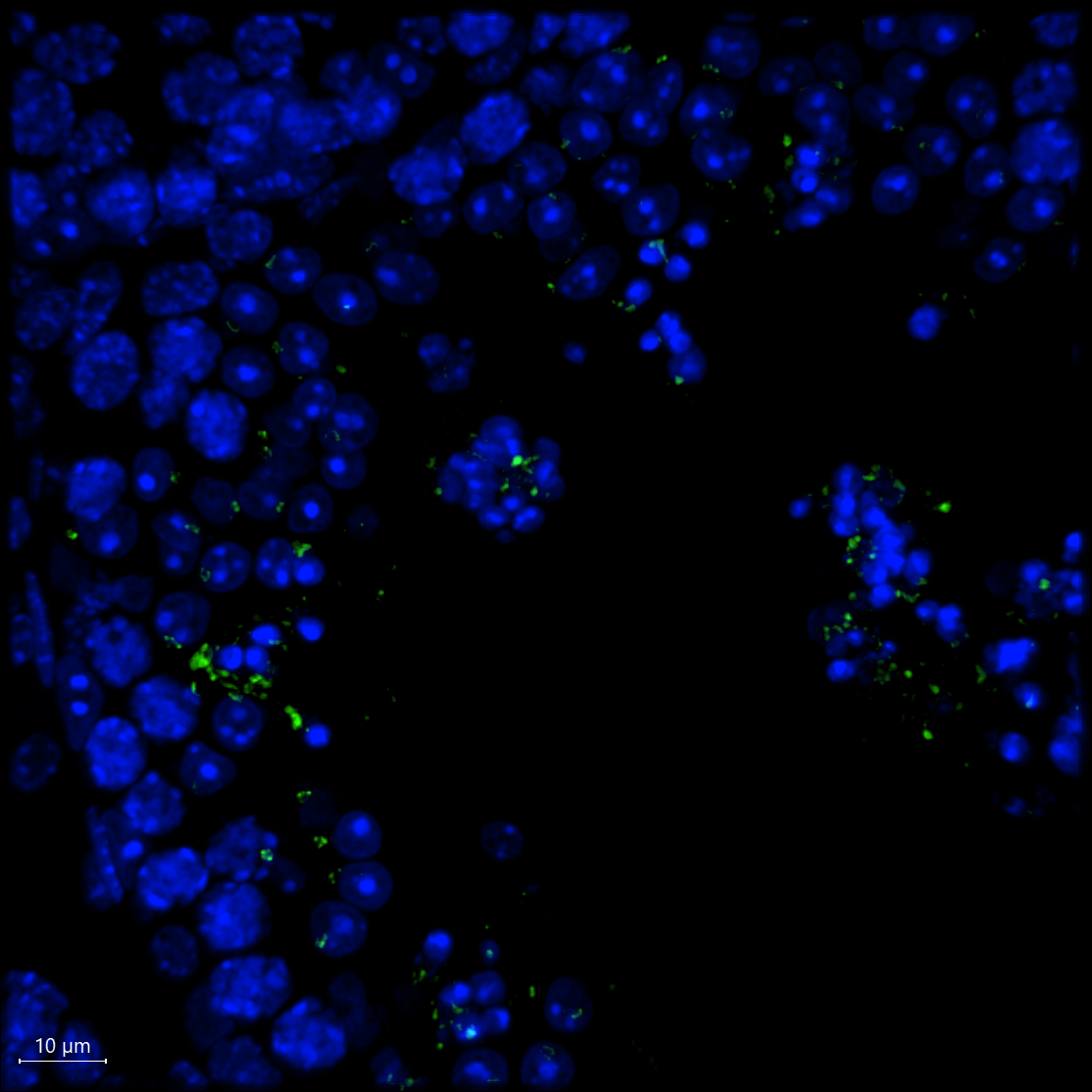

Supplement: Figure 3—source data 1. [file elife-83129-fig3-data1.zip › Figure3/Source data of Figure3A/pd60-KO_6.tif]

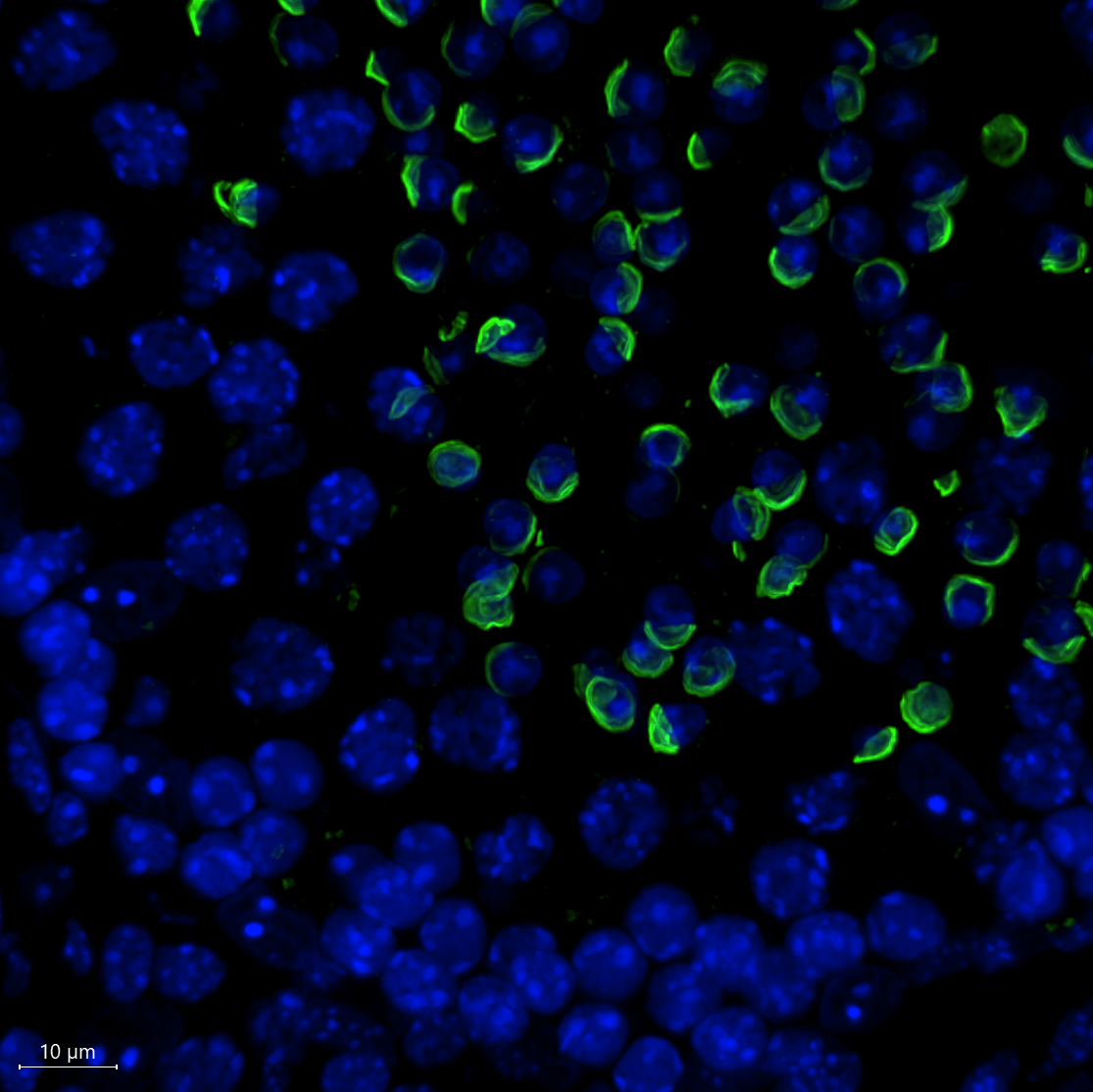

Supplement: Figure 3—source data 1. [file elife-83129-fig3-data1.zip › Figure3/Source data of Figure3A/pd60-WT_1.tif]

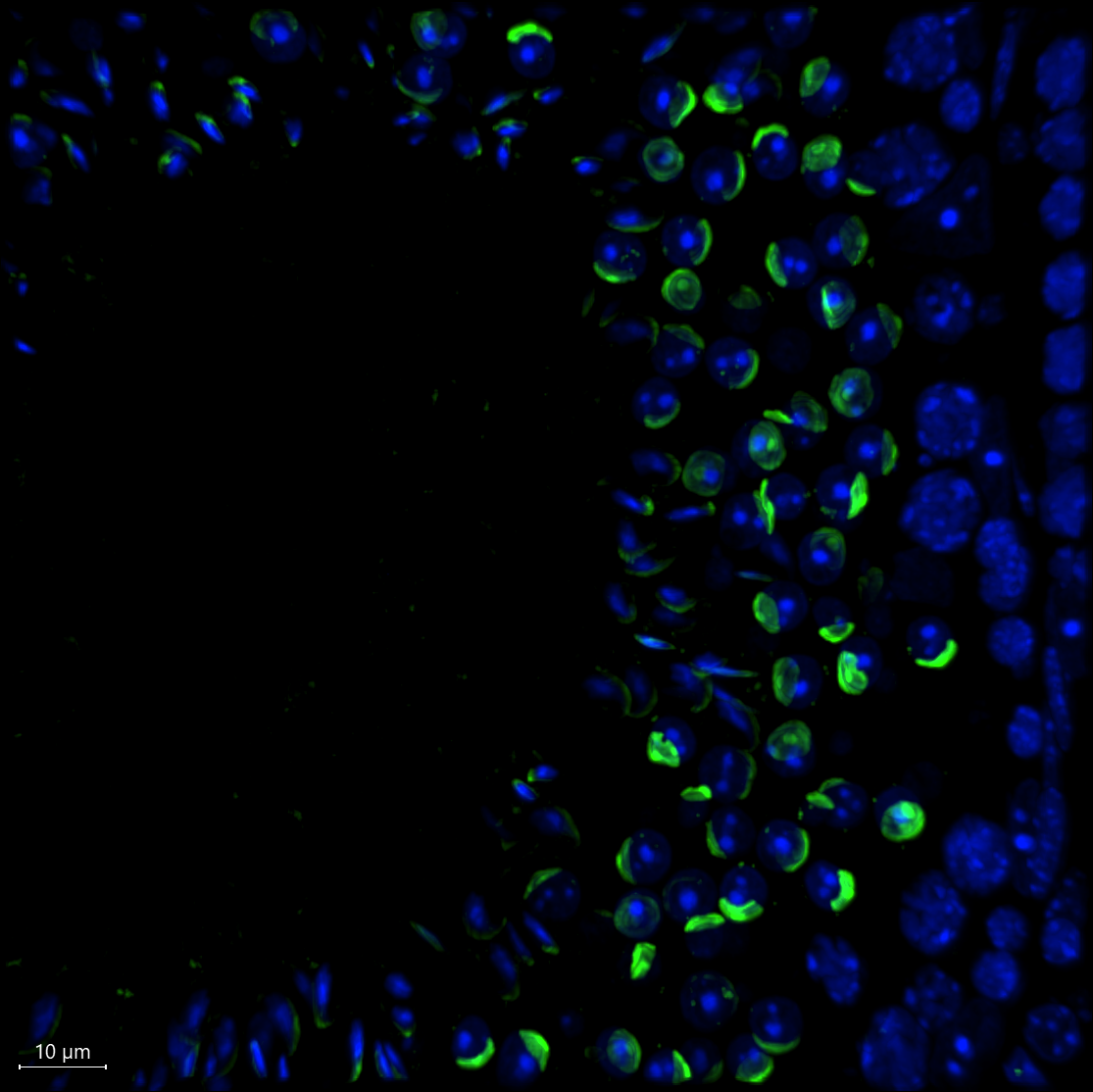

Supplement: Figure 3—source data 1. [file elife-83129-fig3-data1.zip › Figure3/Source data of Figure3A/pd60-WT_2.tif]

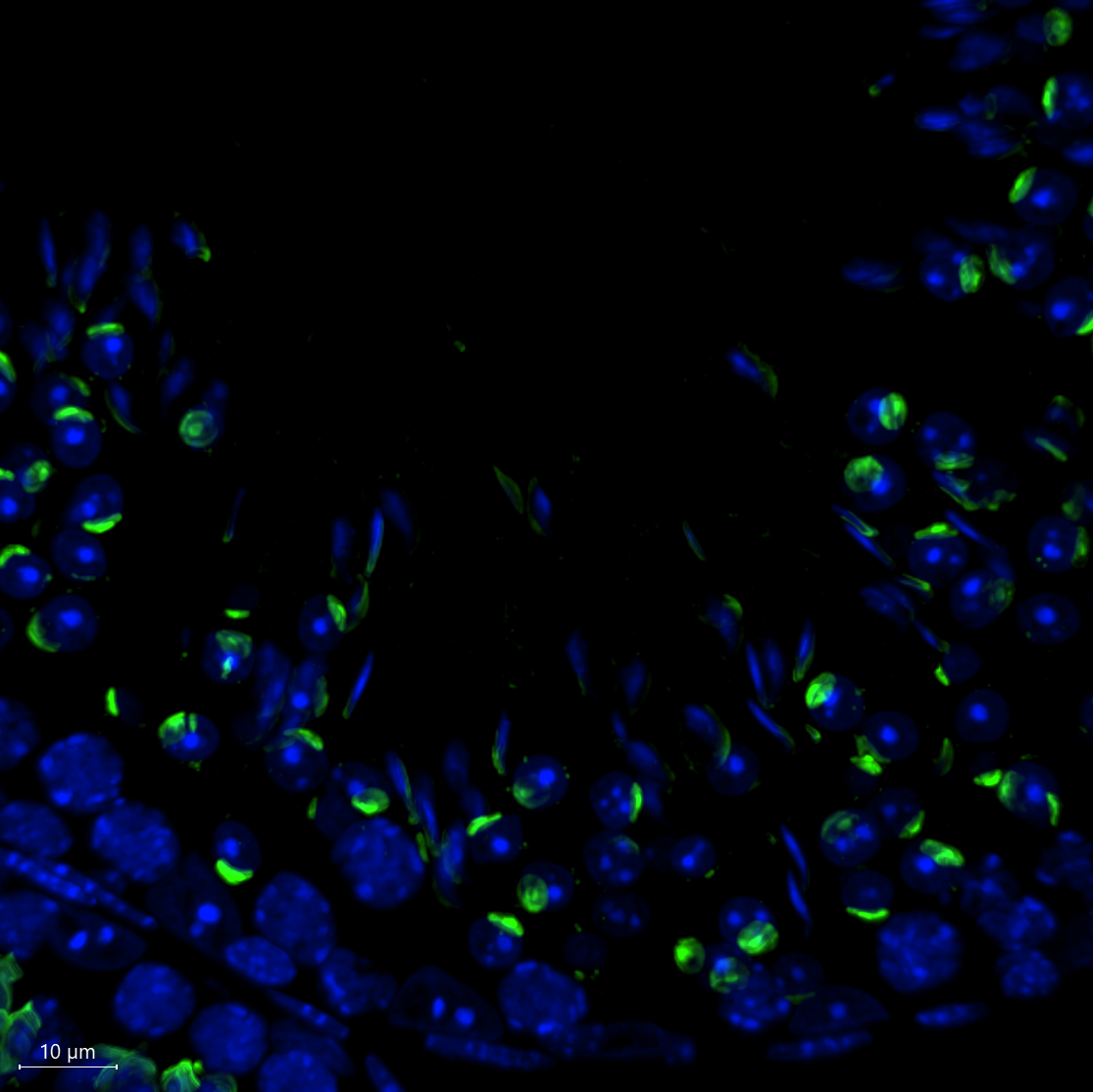

Supplement: Figure 3—source data 1. [file elife-83129-fig3-data1.zip › Figure3/Source data of Figure3A/pd60-WT_3.tif]

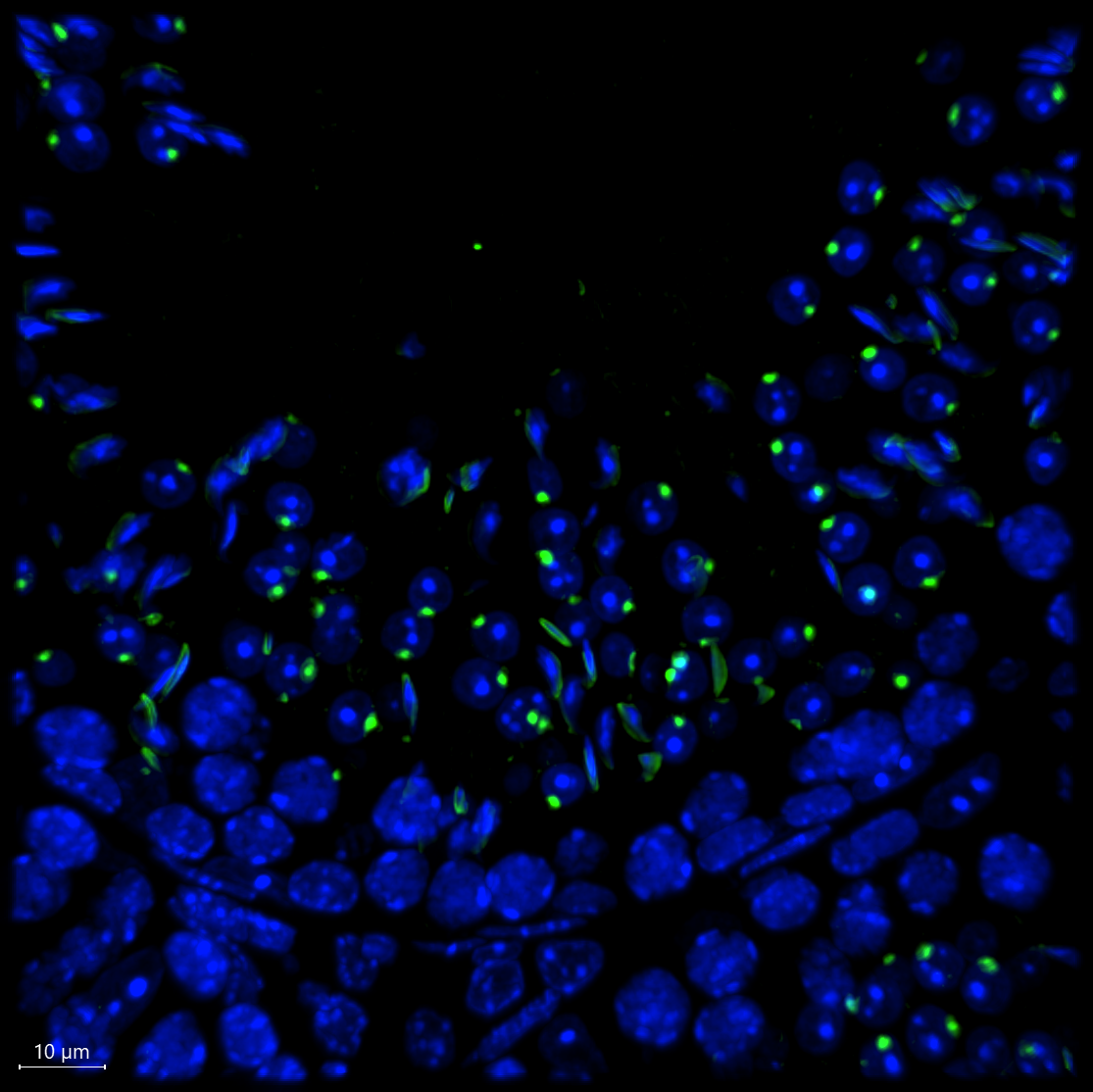

Supplement: Figure 3—source data 1. [file elife-83129-fig3-data1.zip › Figure3/Source data of Figure3A/pd60-WT_4.tif]

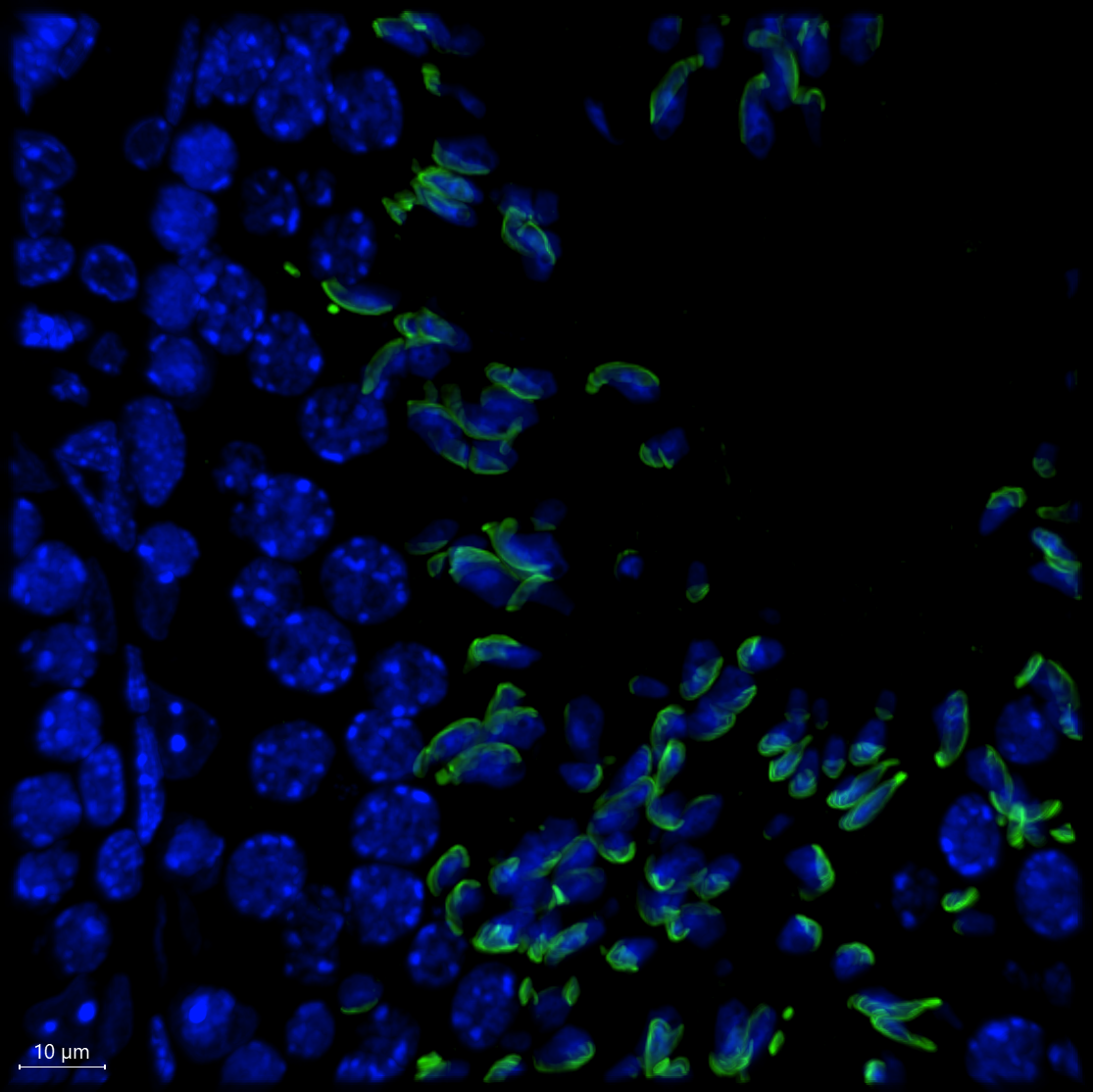

Supplement: Figure 3—source data 1. [file elife-83129-fig3-data1.zip › Figure3/Source data of Figure3A/pd60-WT_5.tif]

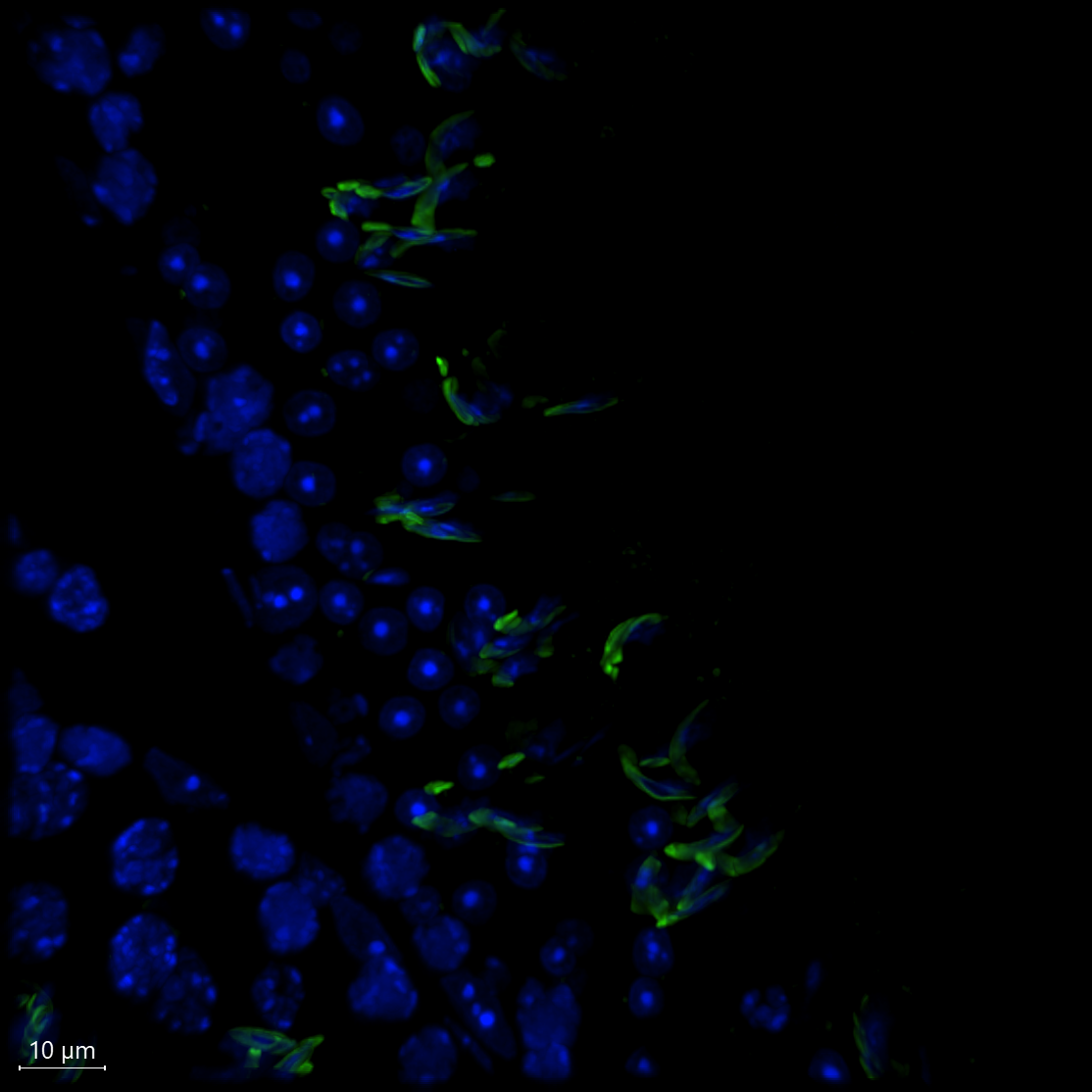

Supplement: Figure 3—source data 1. [file elife-83129-fig3-data1.zip › Figure3/Source data of Figure3A/pd60-WT_6.tif]

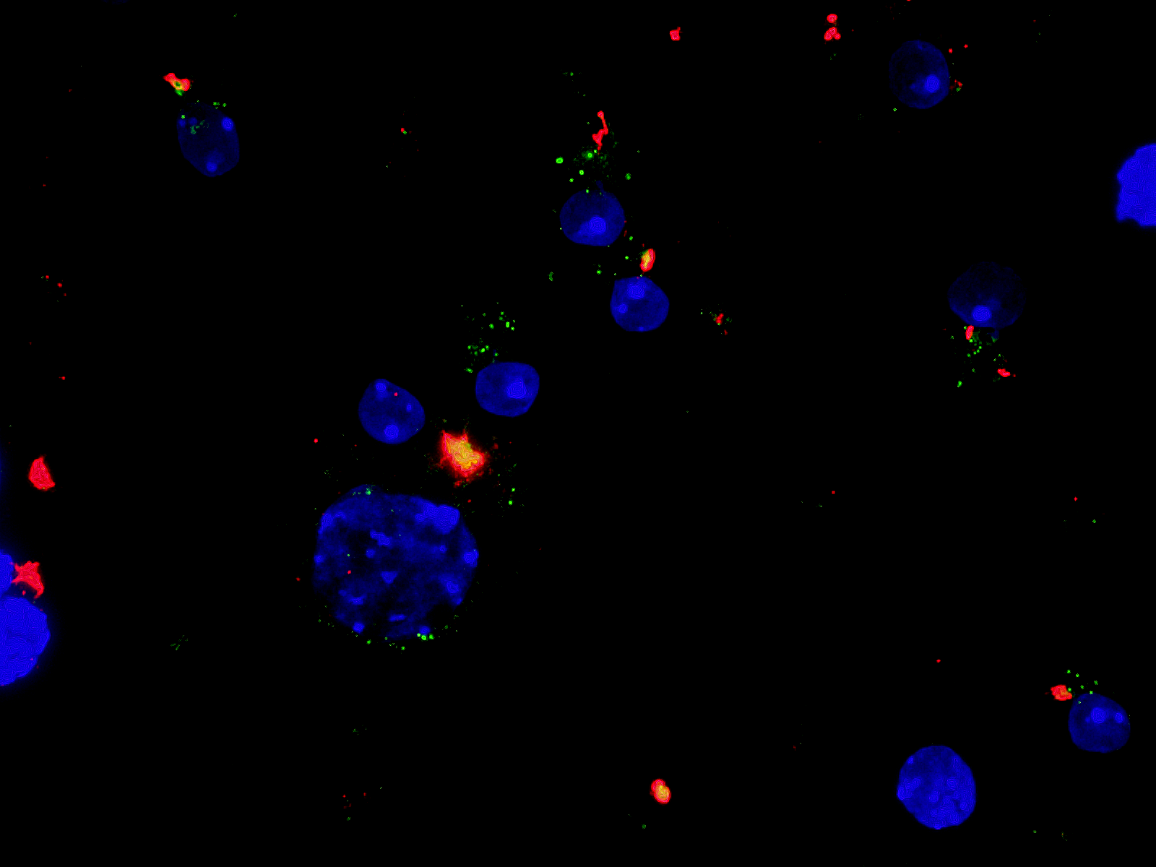

Supplement: Figure 3—source data 1. [file elife-83129-fig3-data1.zip › Figure3/Source data of Figure3C/KO/1/merge.tif]

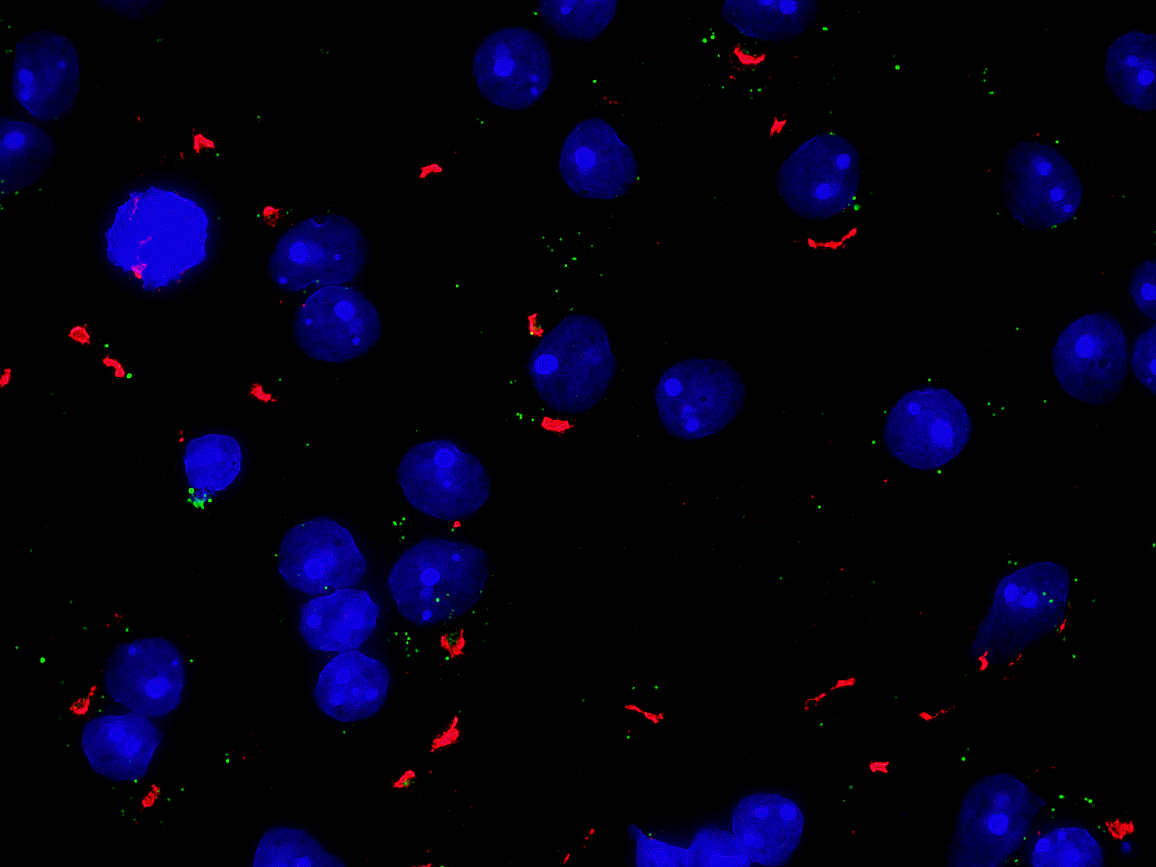

Supplement: Figure 3—source data 1. [file elife-83129-fig3-data1.zip › Figure3/Source data of Figure3C/KO/2/merge.tif]

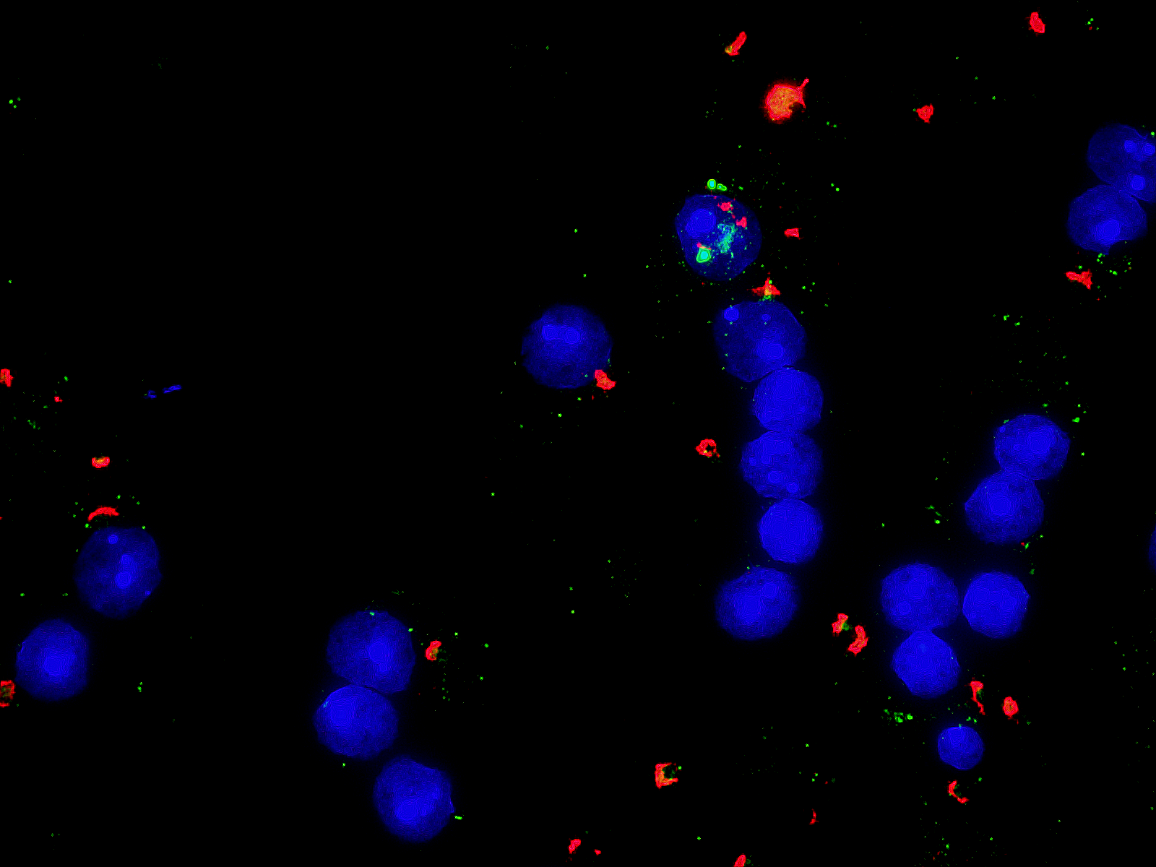

Supplement: Figure 3—source data 1. [file elife-83129-fig3-data1.zip › Figure3/Source data of Figure3C/KO/3/merge.tif]

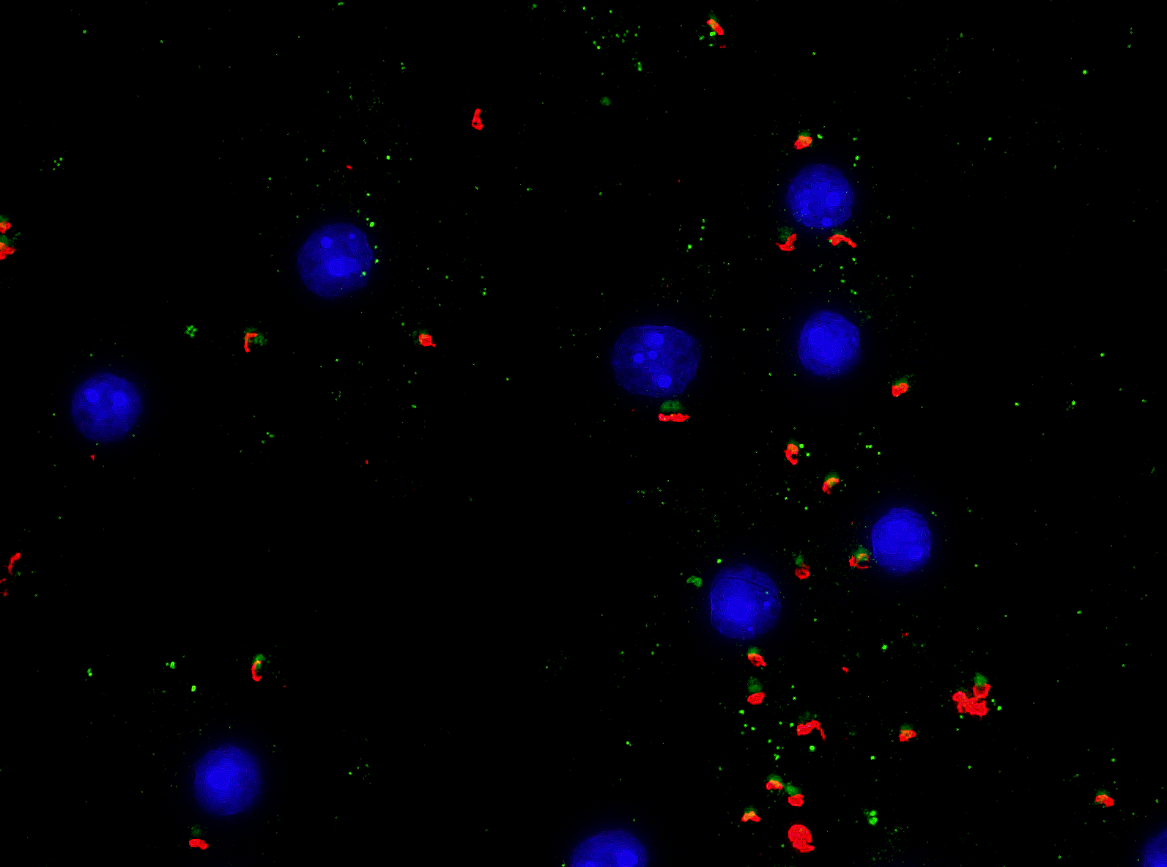

Supplement: Figure 3—source data 1. [file elife-83129-fig3-data1.zip › Figure3/Source data of Figure3C/KO/4/merge.tif]

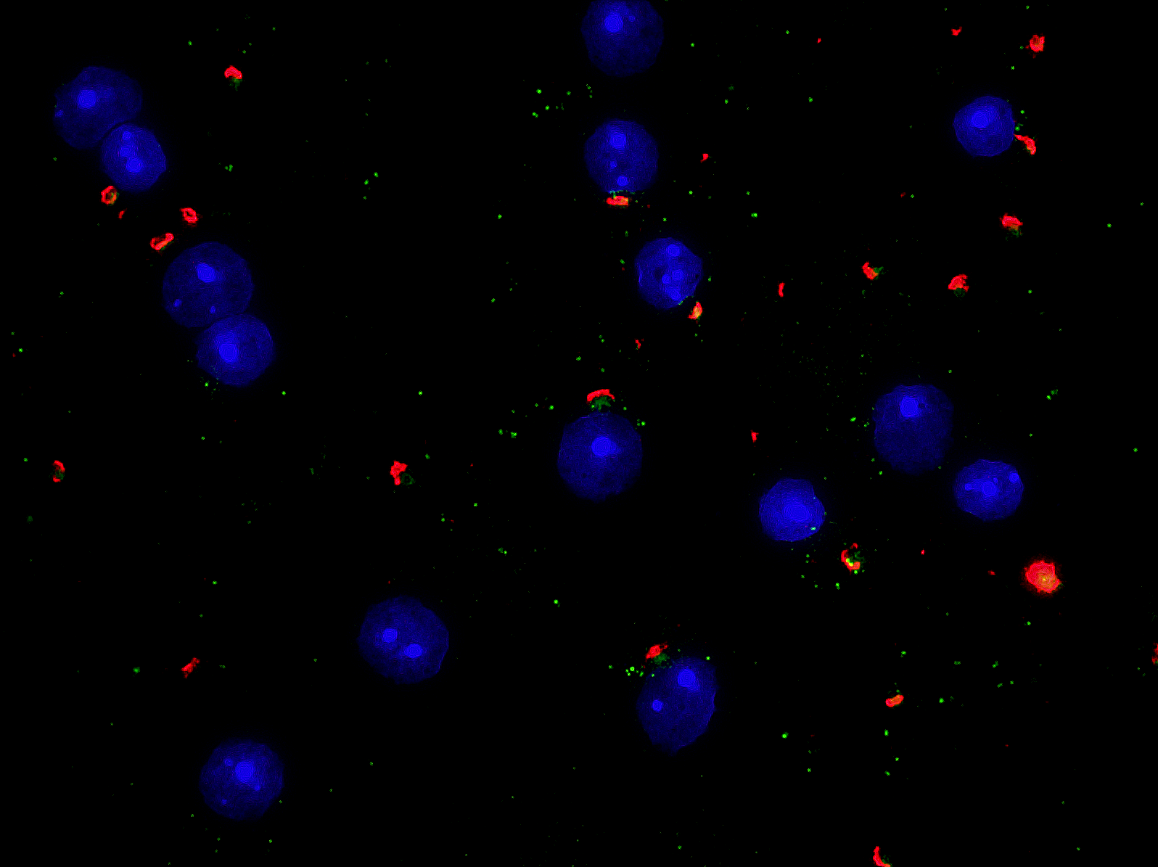

Supplement: Figure 3—source data 1. [file elife-83129-fig3-data1.zip › Figure3/Source data of Figure3C/KO/5/merge.tif]

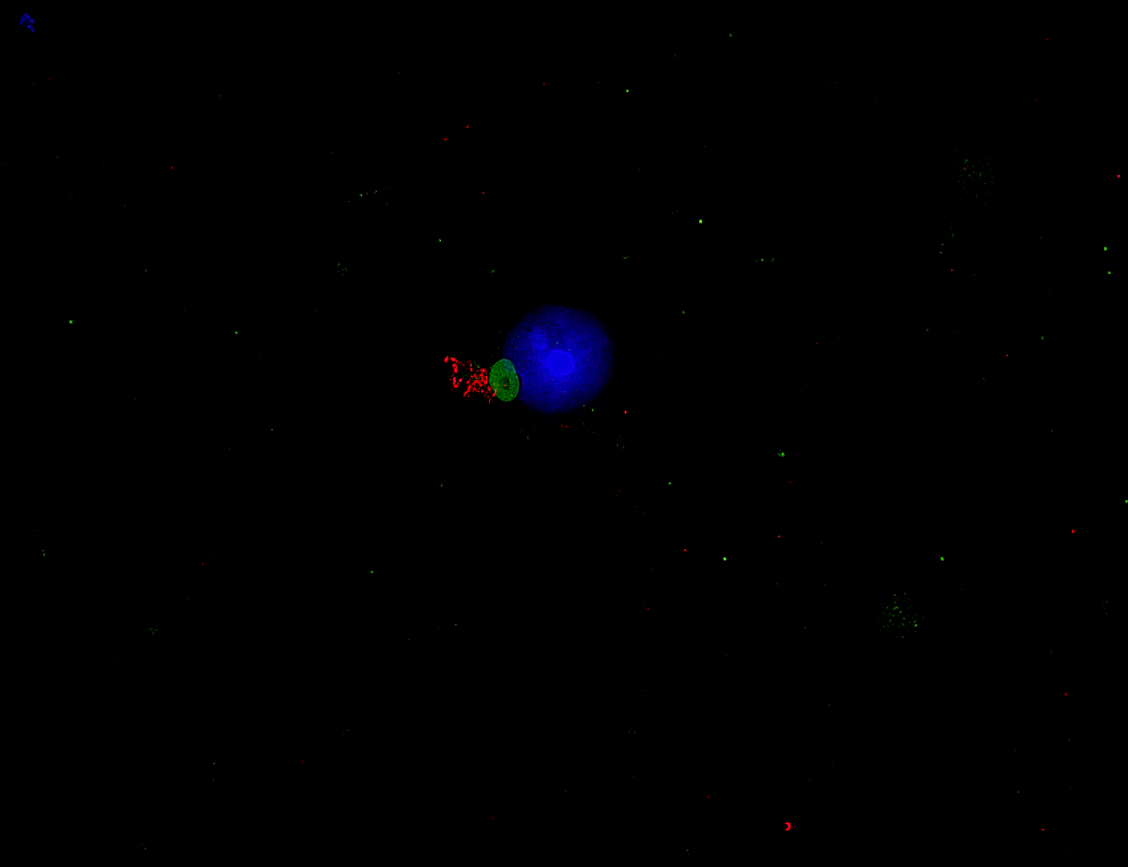

Supplement: Figure 3—source data 1. [file elife-83129-fig3-data1.zip › Figure3/Source data of Figure3C/WT/1/merge.tif]

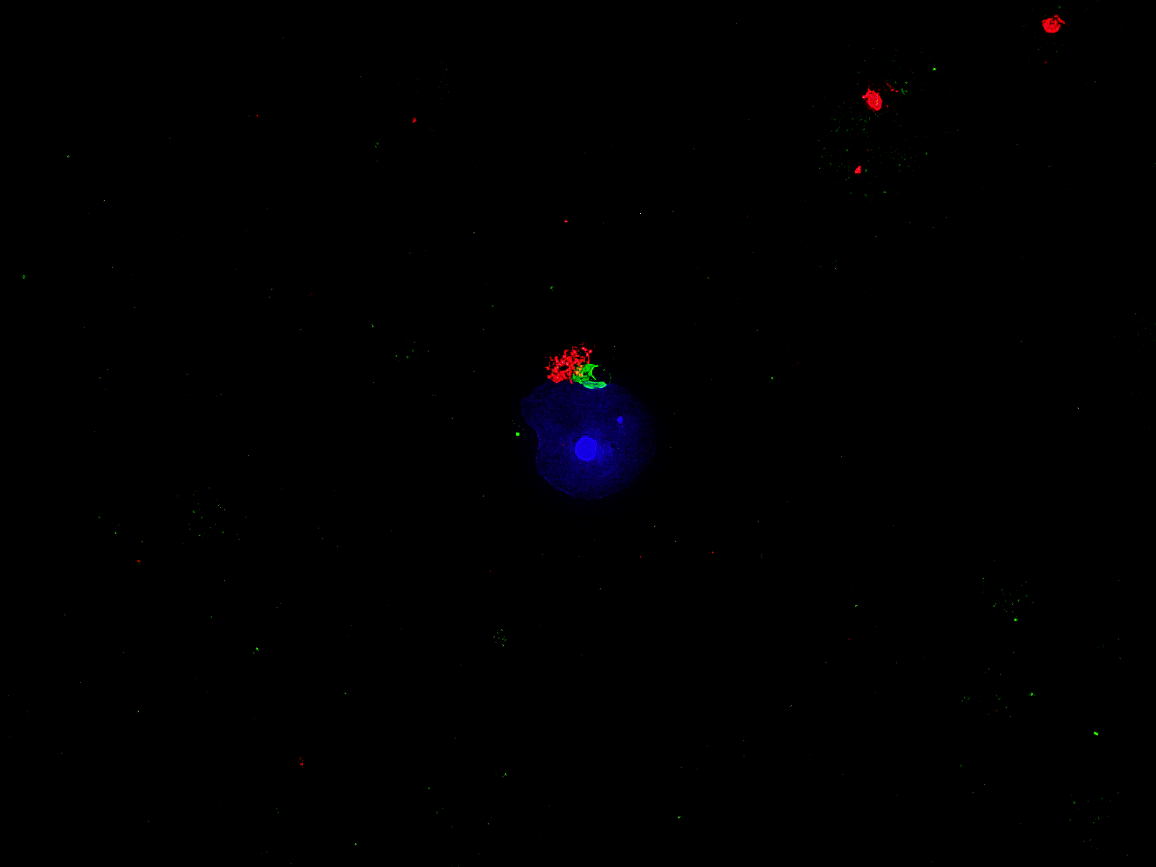

Supplement: Figure 3—source data 1. [file elife-83129-fig3-data1.zip › Figure3/Source data of Figure3C/WT/2/merge.tif]

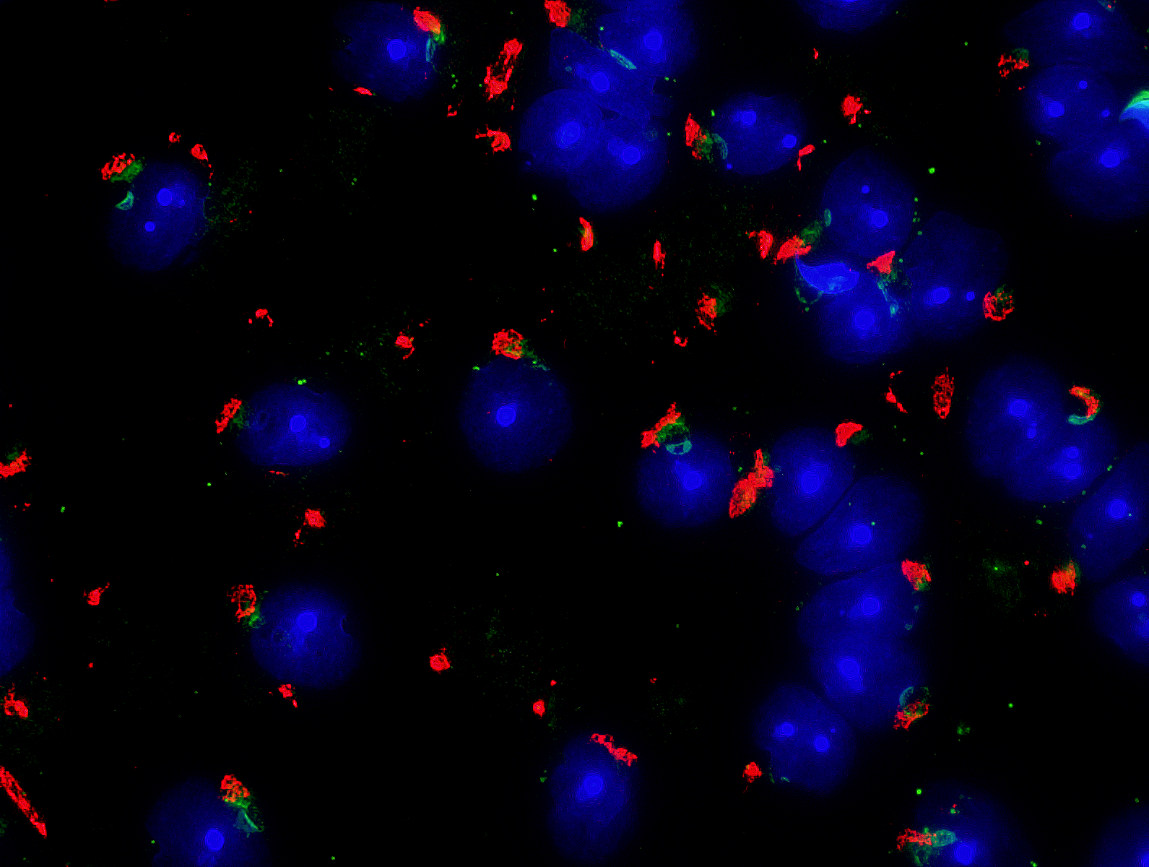

Supplement: Figure 3—source data 1. [file elife-83129-fig3-data1.zip › Figure3/Source data of Figure3C/WT/3/merge.tif]

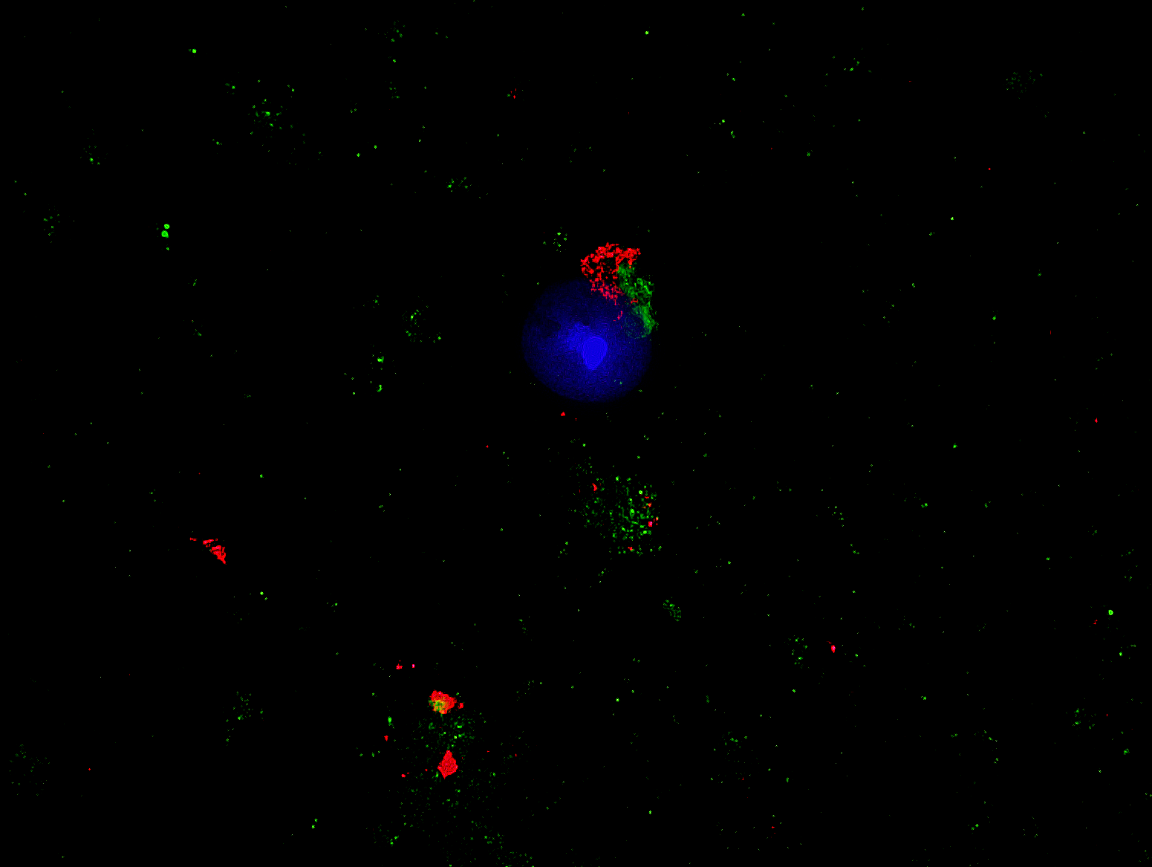

Supplement: Figure 3—source data 1. [file elife-83129-fig3-data1.zip › Figure3/Source data of Figure3C/WT/4/merge.tif]

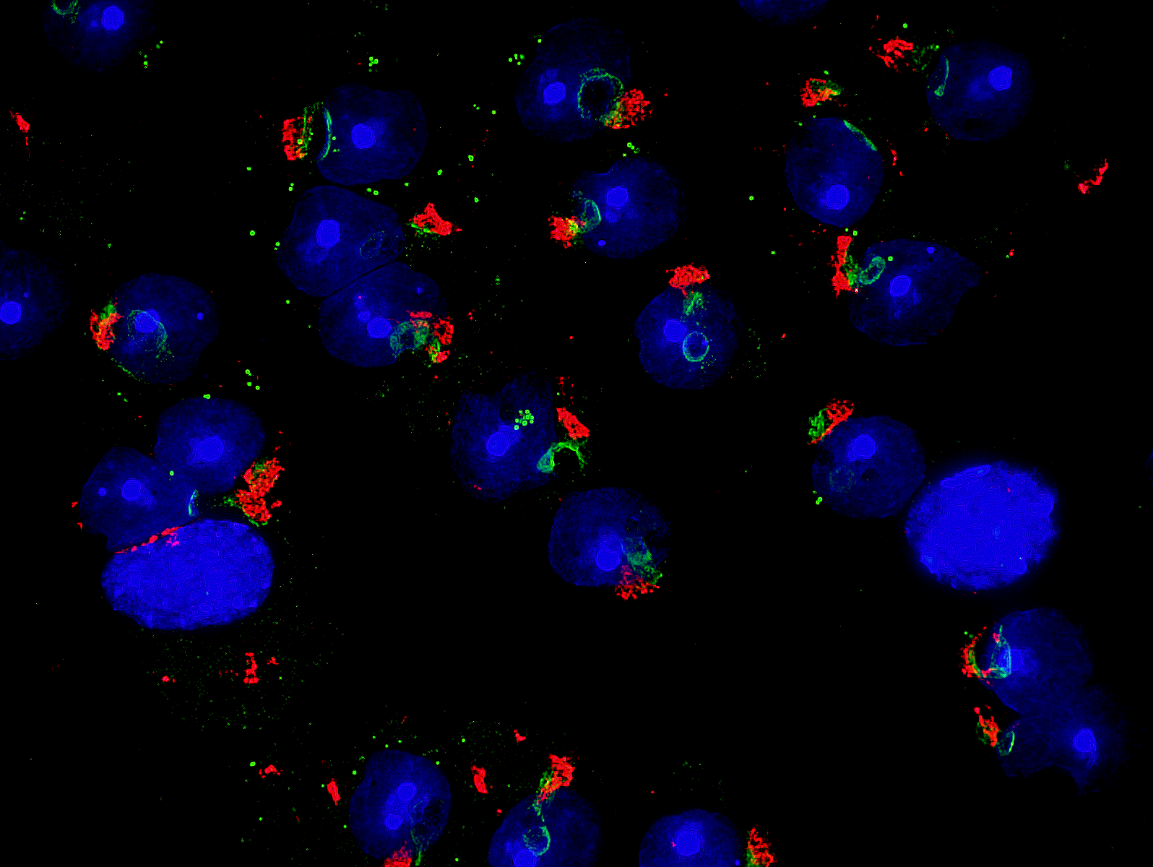

Supplement: Figure 3—source data 1. [file elife-83129-fig3-data1.zip › Figure3/Source data of Figure3C/WT/5/MERGE.tif]

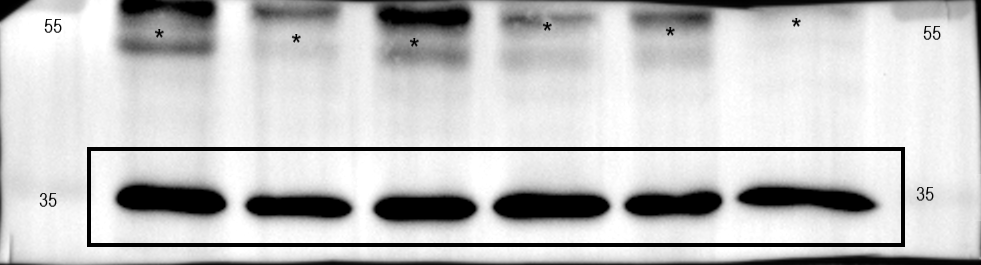

Supplement: Figure 4—source data 1. [file elife-83129-fig4-data1.zip › Figure4/Source data of Figure4A/Labelled blots of Figure4A-GAPDH.tif]

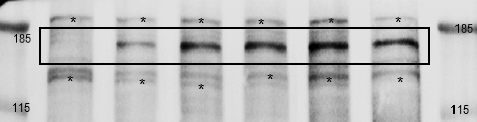

Supplement: Figure 4—source data 1. [file elife-83129-fig4-data1.zip › Figure4/Source data of Figure4A/Labelled blots of Figure4A-SSH2.Tif]

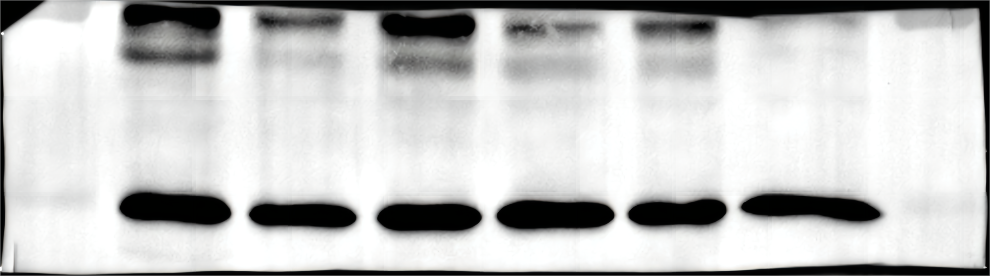

Supplement: Figure 4—source data 1. [file elife-83129-fig4-data1.zip › Figure4/Source data of Figure4A/Raw blots of Figure4A-GAPDH.png]

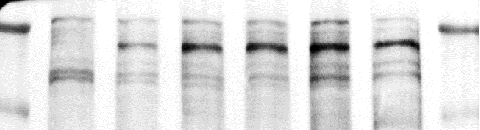

Supplement: Figure 4—source data 1. [file elife-83129-fig4-data1.zip › Figure4/Source data of Figure4A/Raw blots of Figure4A-SSH2.tif]

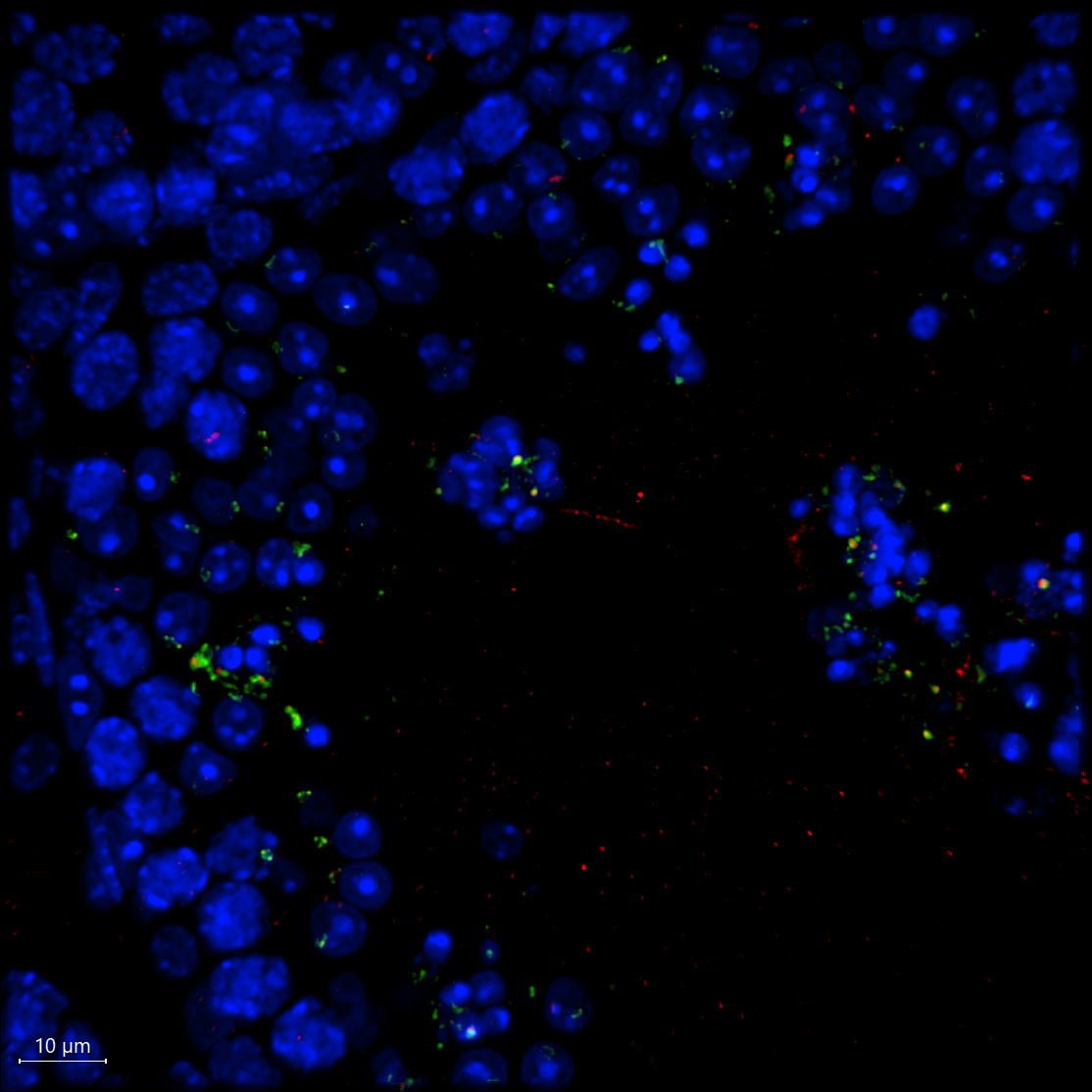

Supplement: Figure 4—source data 1. [file elife-83129-fig4-data1.zip › Figure4/Source data of Figure4B-4C/1 pd60-KO.tif]

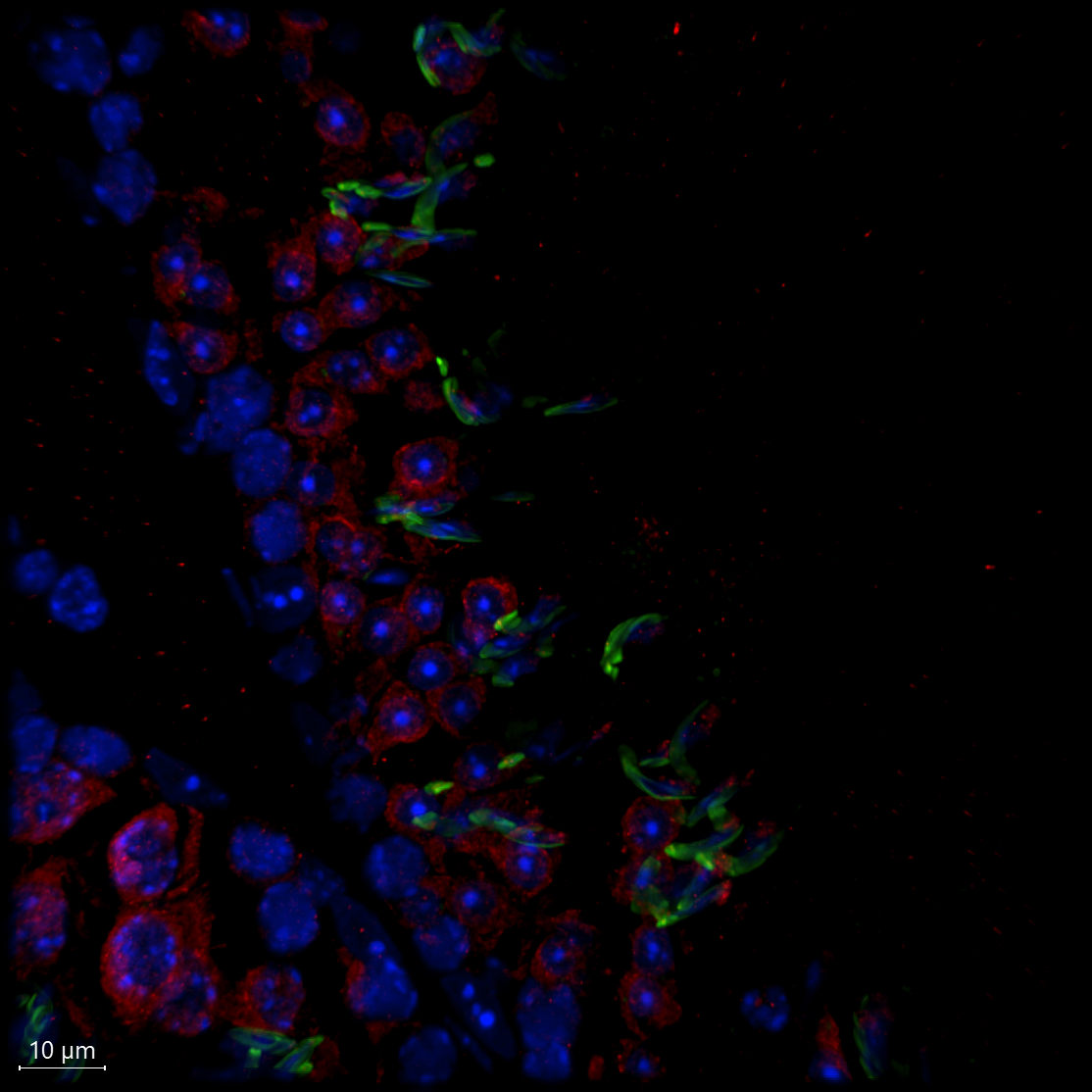

Supplement: Figure 4—source data 1. [file elife-83129-fig4-data1.zip › Figure4/Source data of Figure4B-4C/1 pd60-WT.tif]

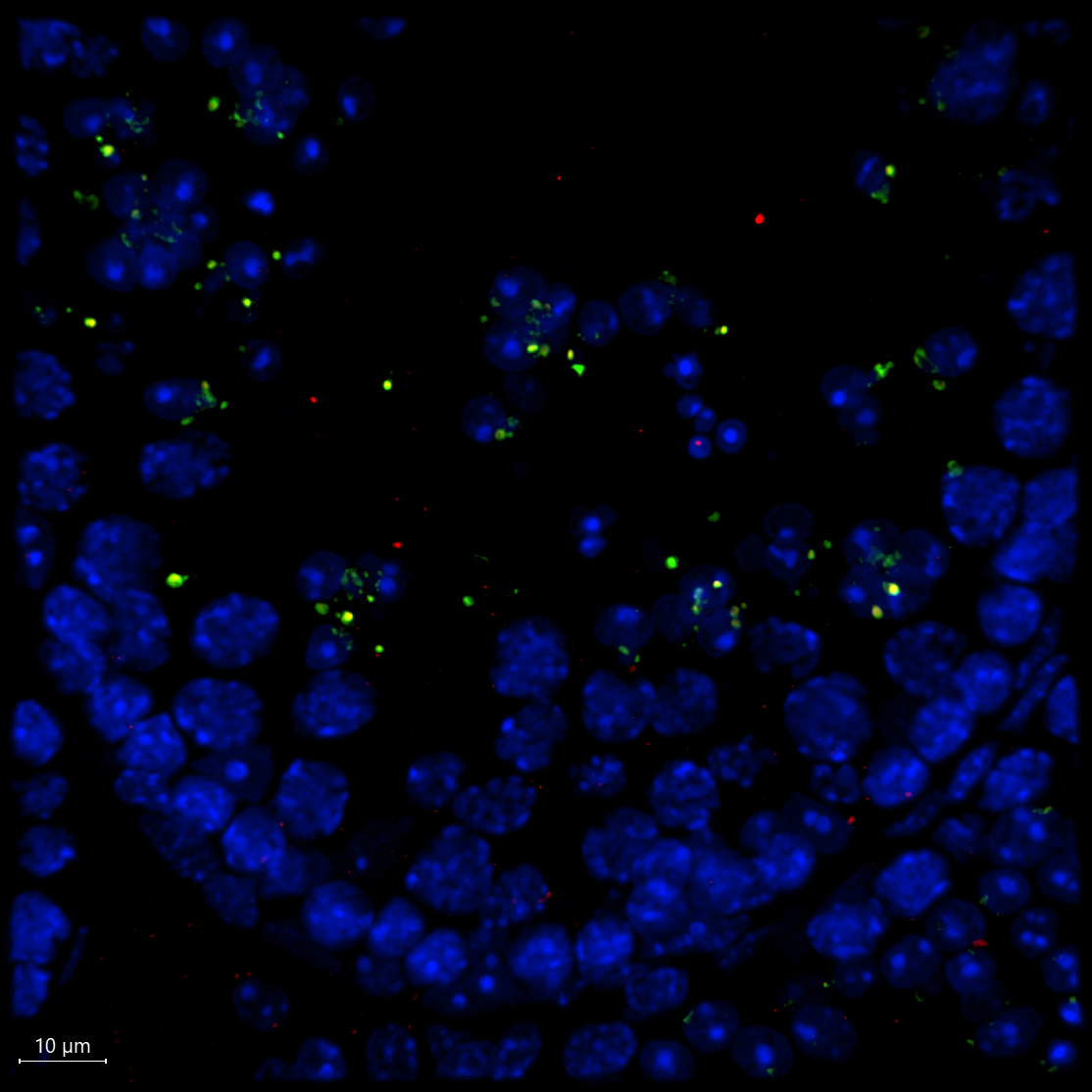

Supplement: Figure 4—source data 1. [file elife-83129-fig4-data1.zip › Figure4/Source data of Figure4B-4C/11-12 pd60-KO.tif]

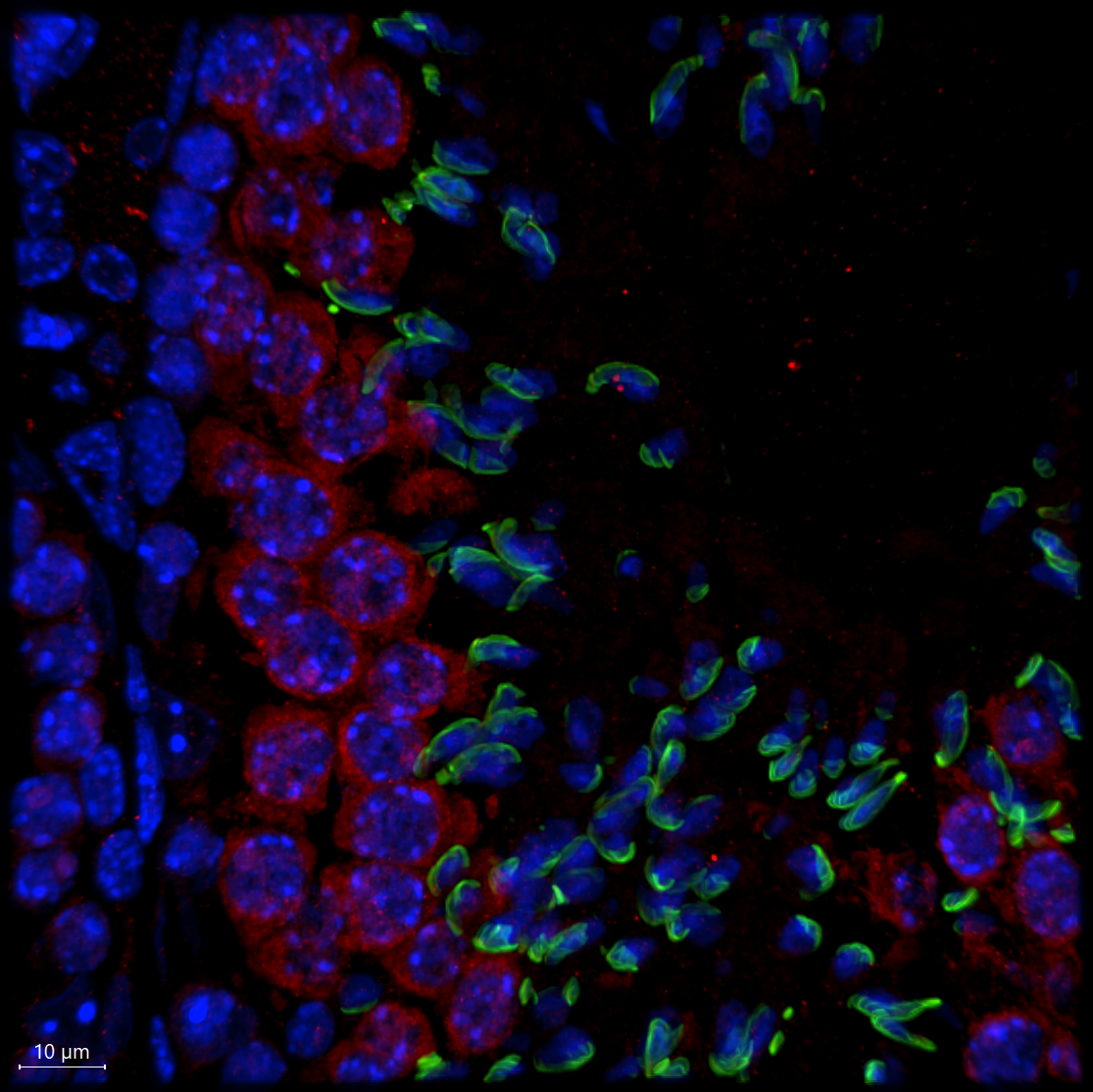

Supplement: Figure 4—source data 1. [file elife-83129-fig4-data1.zip › Figure4/Source data of Figure4B-4C/11-12 pd60-WT.tif]

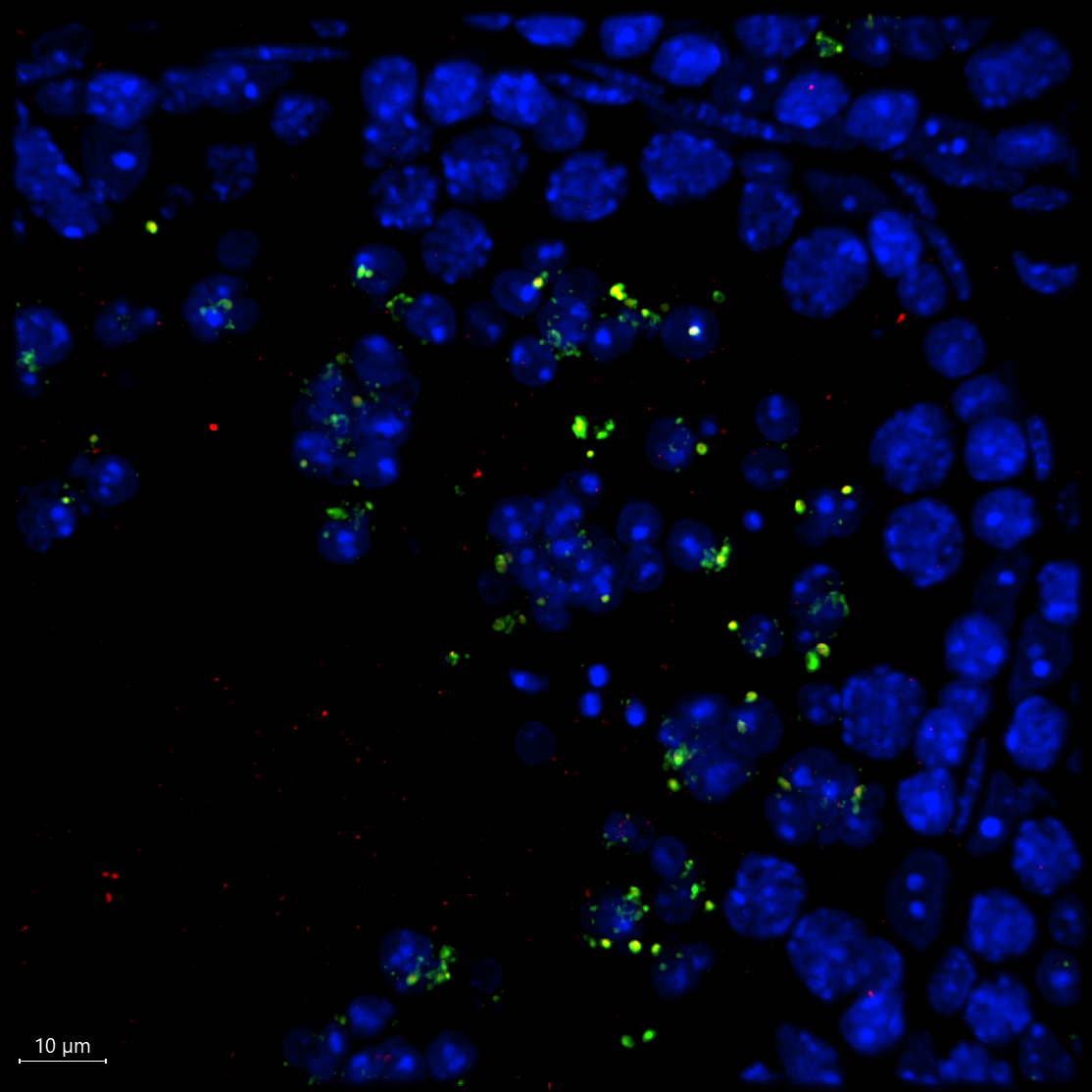

Supplement: Figure 4—source data 1. [file elife-83129-fig4-data1.zip › Figure4/Source data of Figure4B-4C/2-3 pd60-KO.tif]

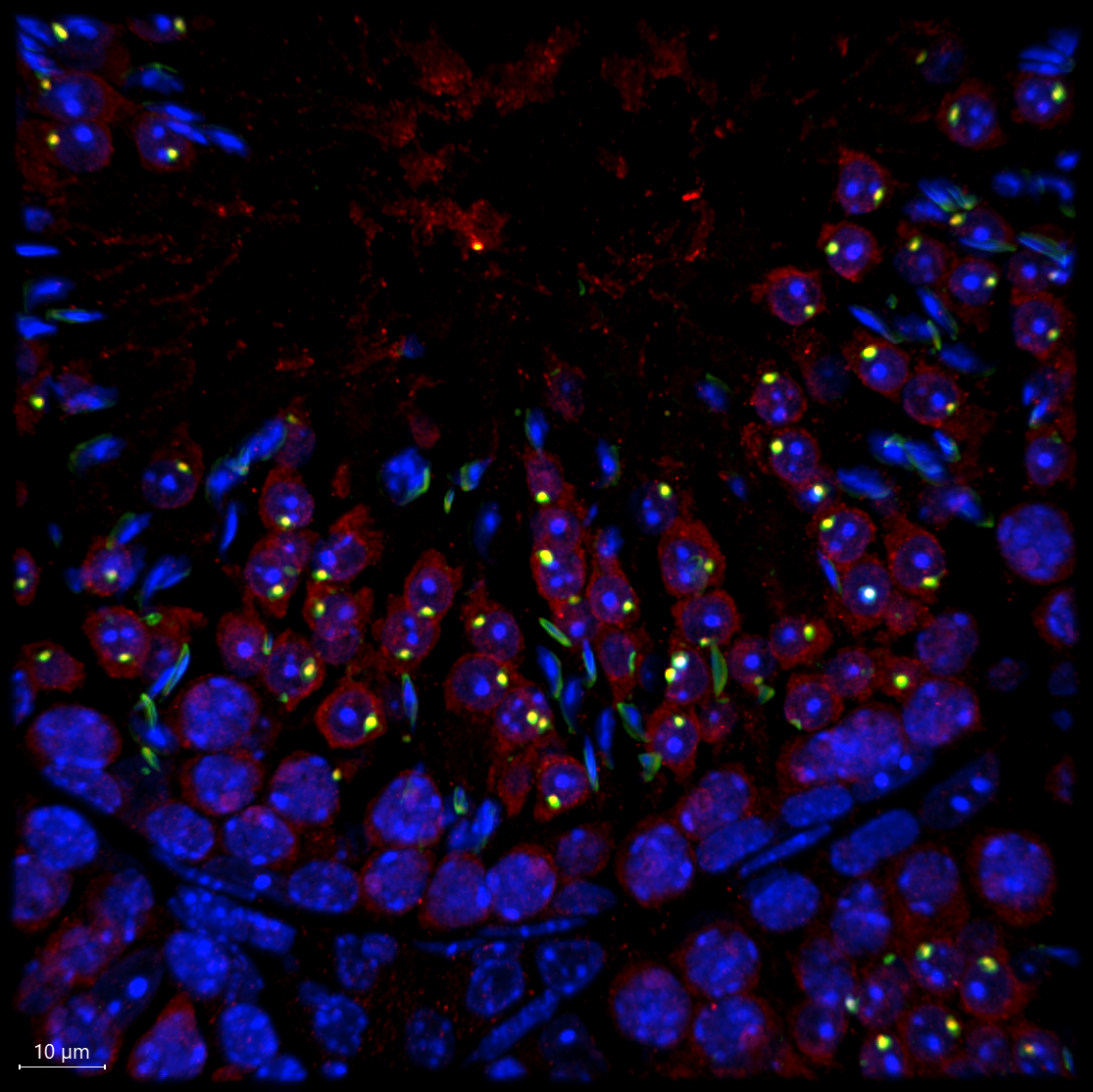

Supplement: Figure 4—source data 1. [file elife-83129-fig4-data1.zip › Figure4/Source data of Figure4B-4C/2-3 pd60-WT.tif]

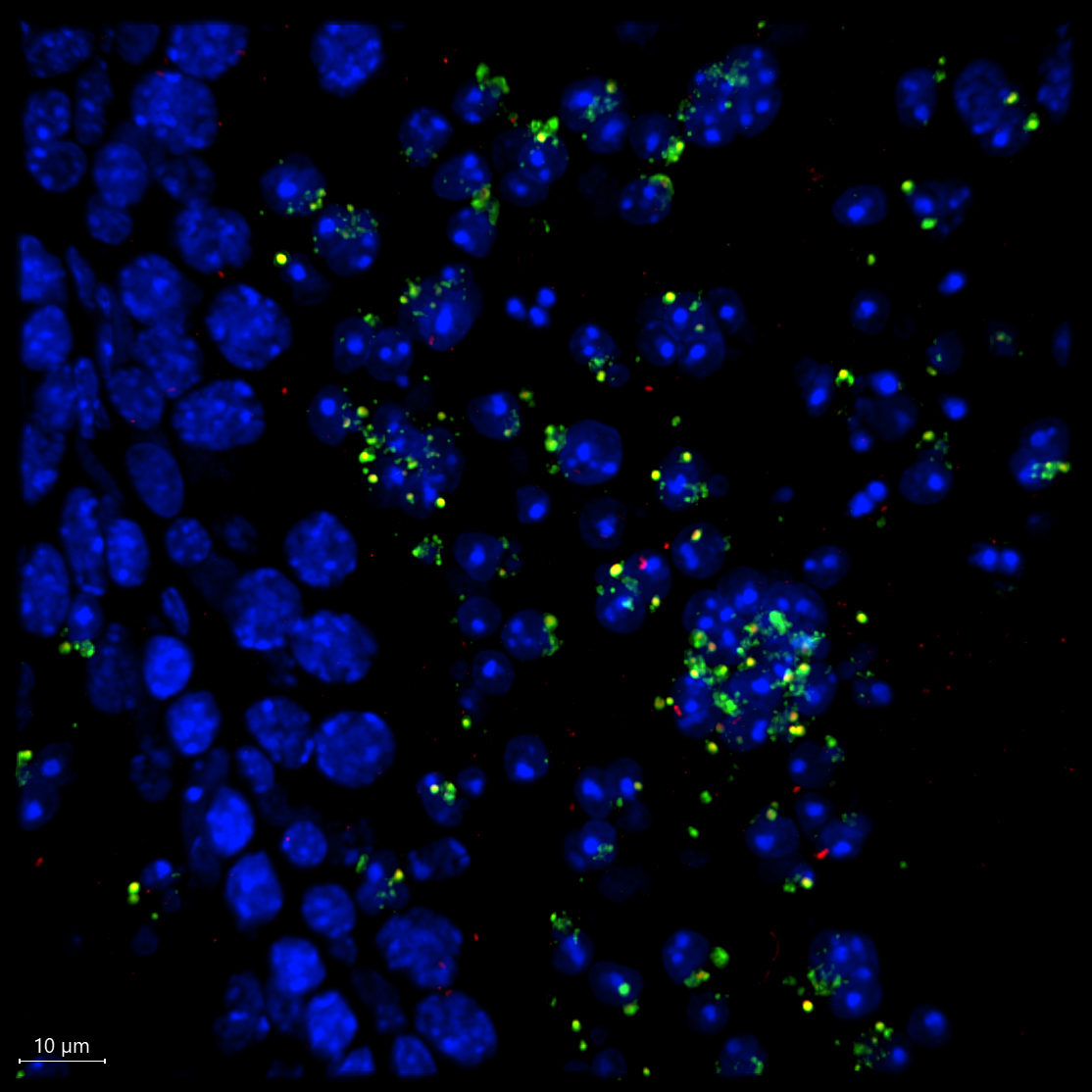

Supplement: Figure 4—source data 1. [file elife-83129-fig4-data1.zip › Figure4/Source data of Figure4B-4C/4-6 pd60-KO.tif]

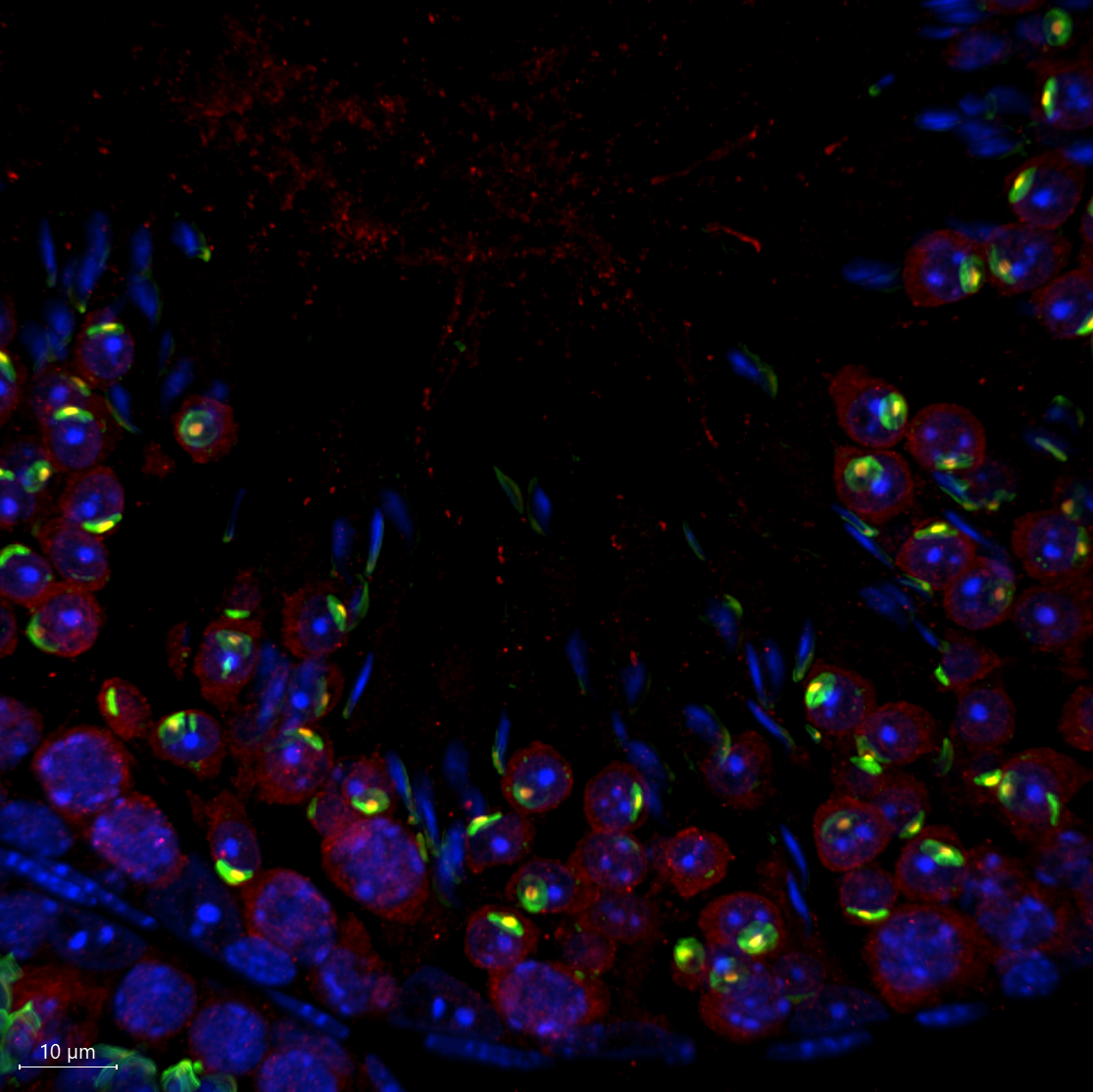

Supplement: Figure 4—source data 1. [file elife-83129-fig4-data1.zip › Figure4/Source data of Figure4B-4C/4-6 pd60-WT.tif]

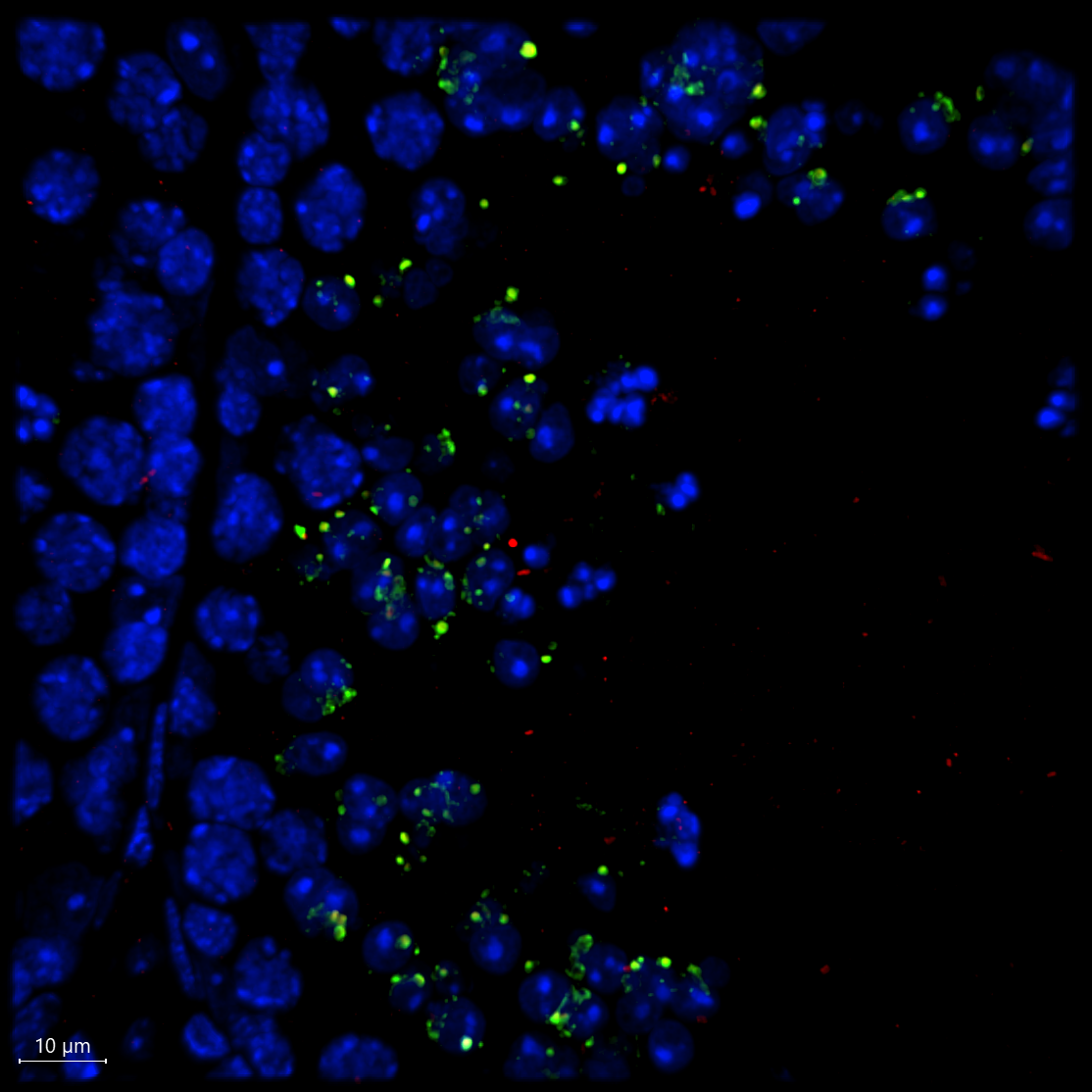

Supplement: Figure 4—source data 1. [file elife-83129-fig4-data1.zip › Figure4/Source data of Figure4B-4C/7-8 pd60-KO.tif]

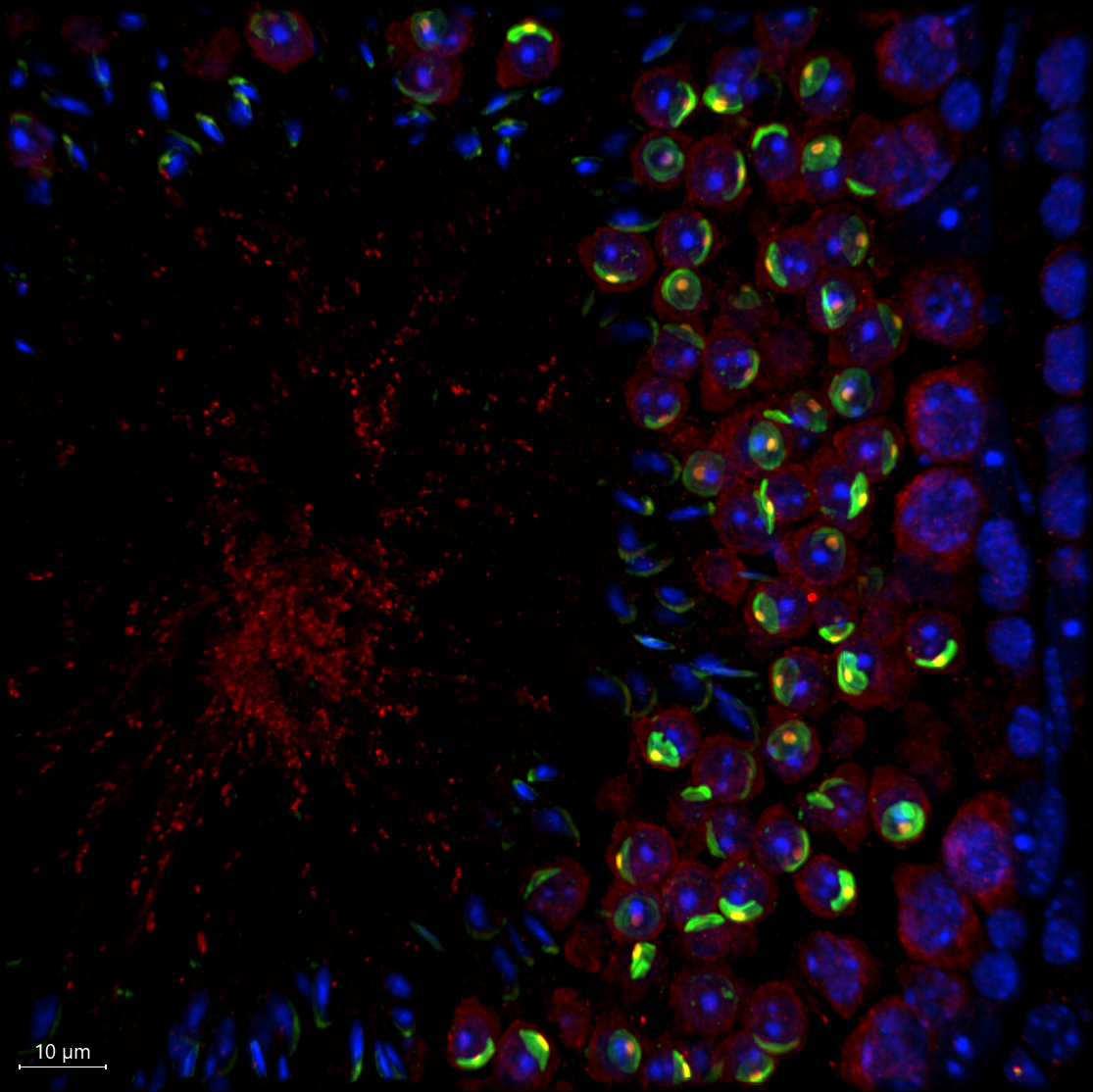

Supplement: Figure 4—source data 1. [file elife-83129-fig4-data1.zip › Figure4/Source data of Figure4B-4C/7-8 pd60-WT.tif]

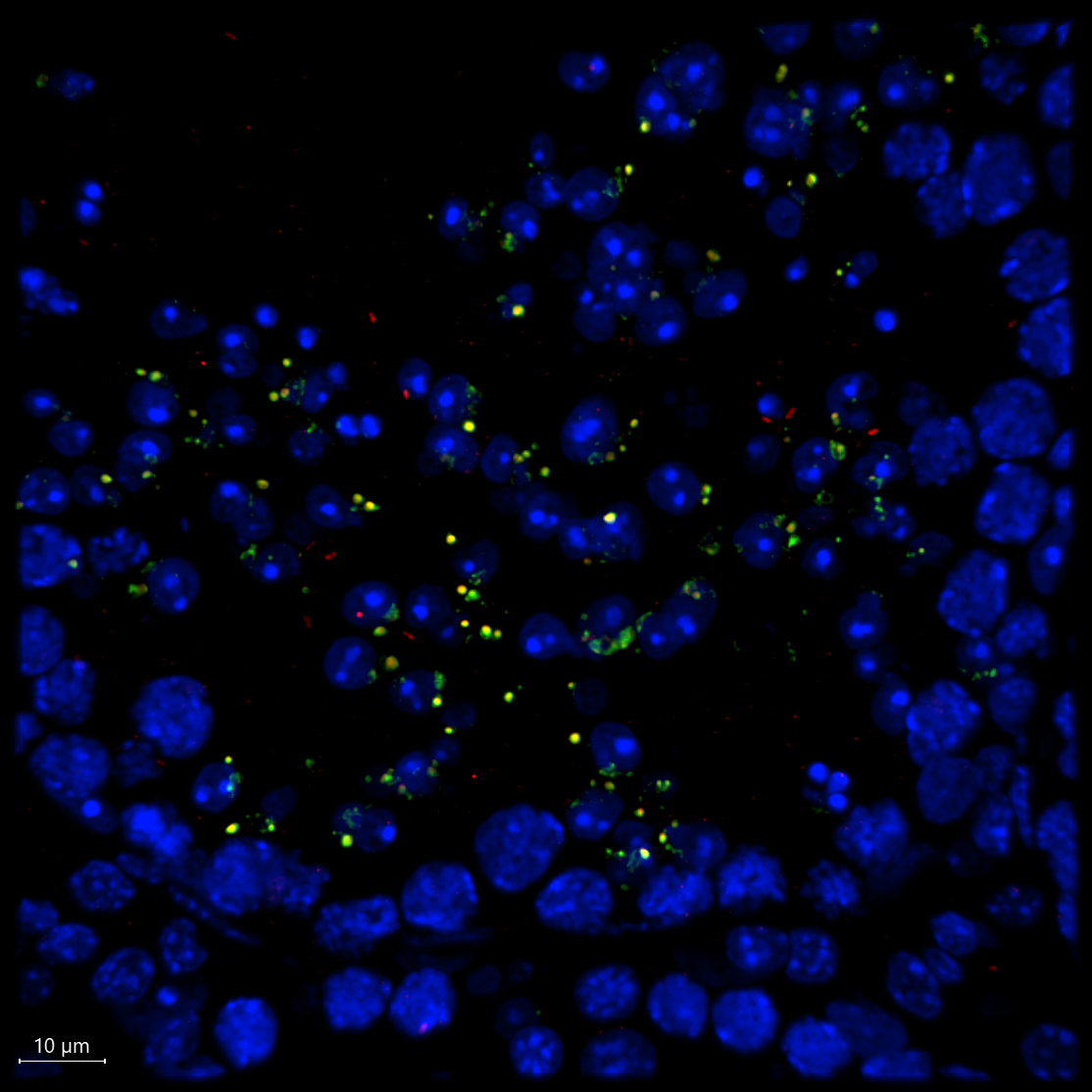

Supplement: Figure 4—source data 1. [file elife-83129-fig4-data1.zip › Figure4/Source data of Figure4B-4C/9-10 pd60-KO.tif]

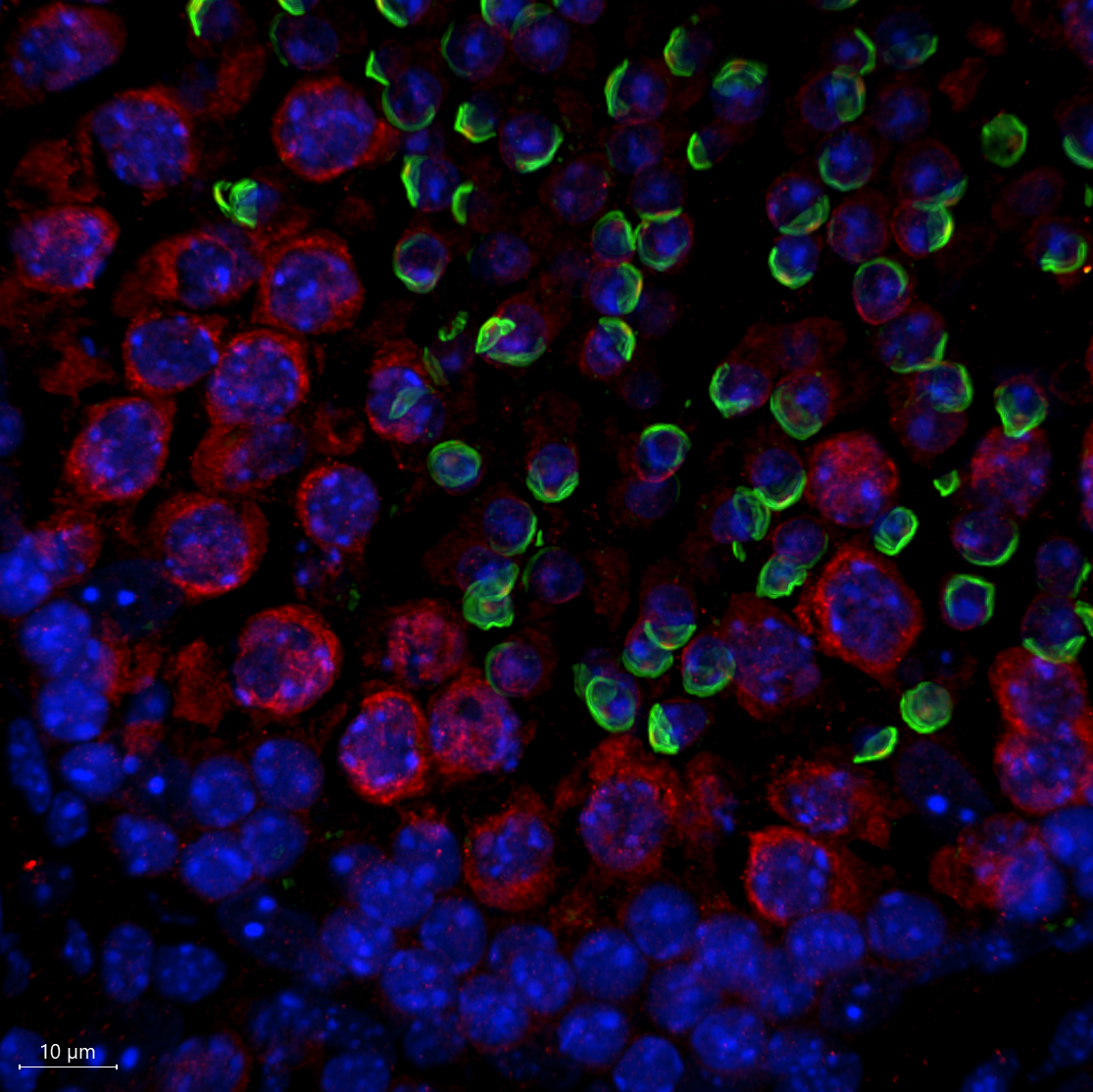

Supplement: Figure 4—source data 1. [file elife-83129-fig4-data1.zip › Figure4/Source data of Figure4B-4C/9-10 pd60-WT.tif]

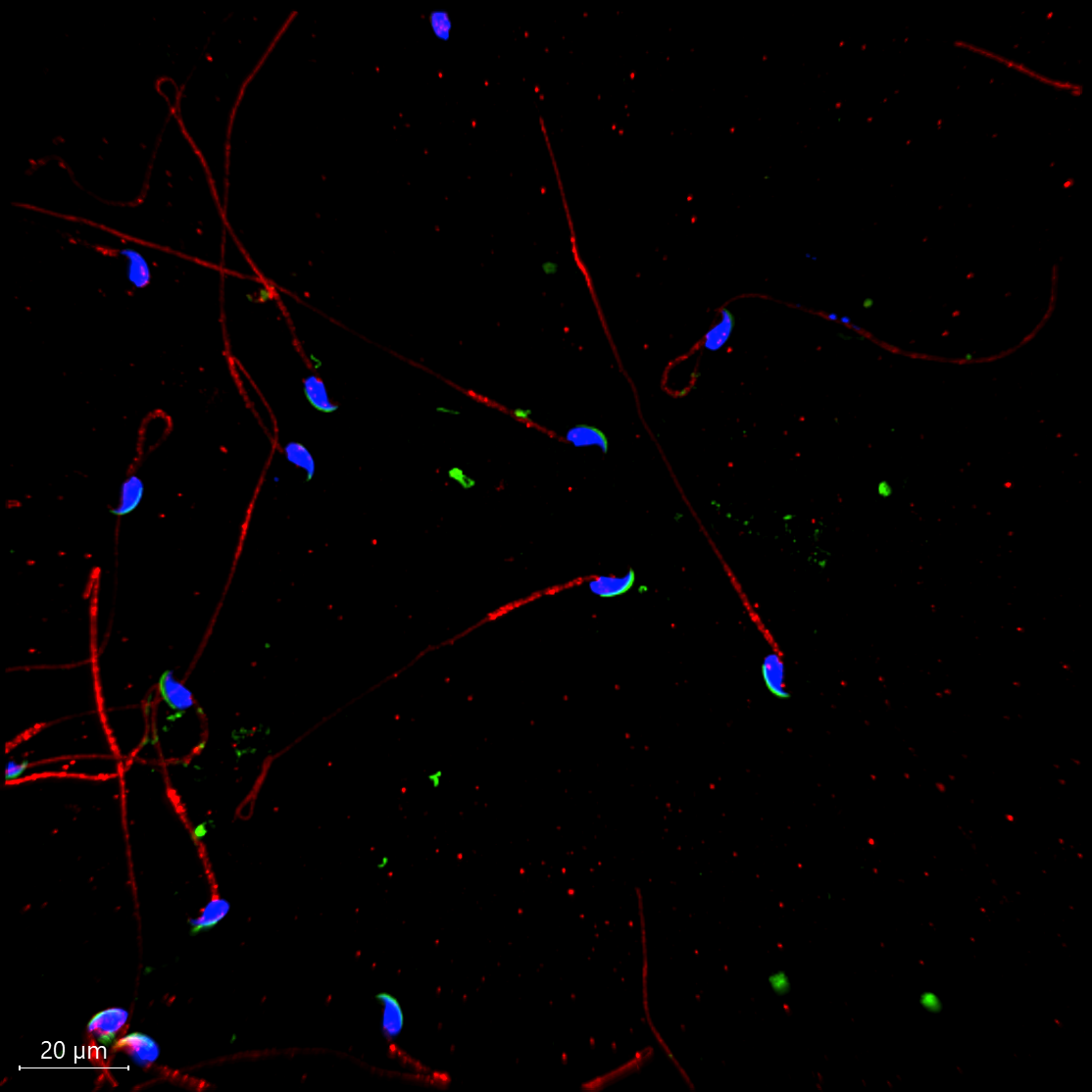

Supplement: Figure 4—source data 1. [file elife-83129-fig4-data1.zip › Figure4/Source data of Figure4B-4C/sperm-1.tif]

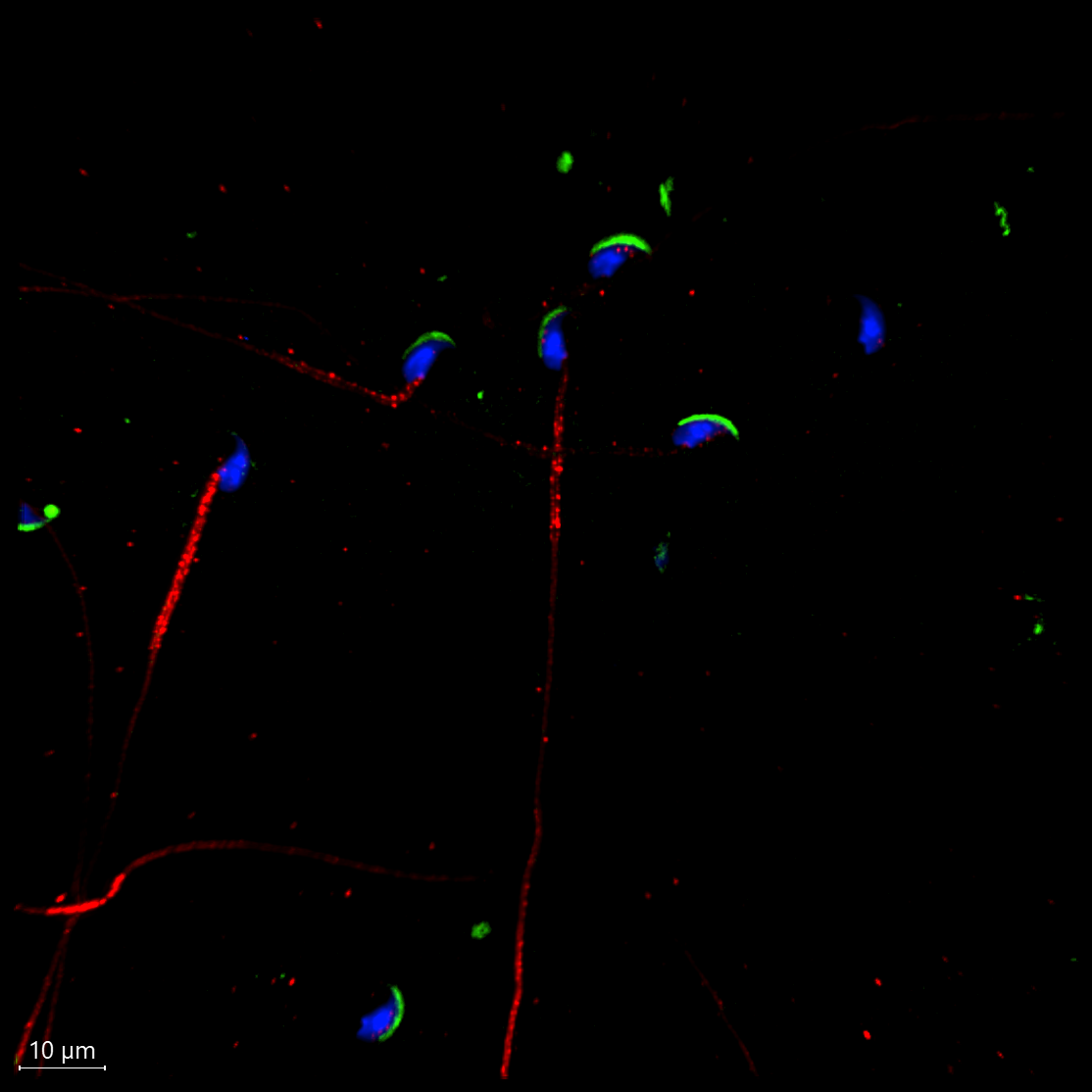

Supplement: Figure 4—source data 1. [file elife-83129-fig4-data1.zip › Figure4/Source data of Figure4B-4C/sperm-2.tif]

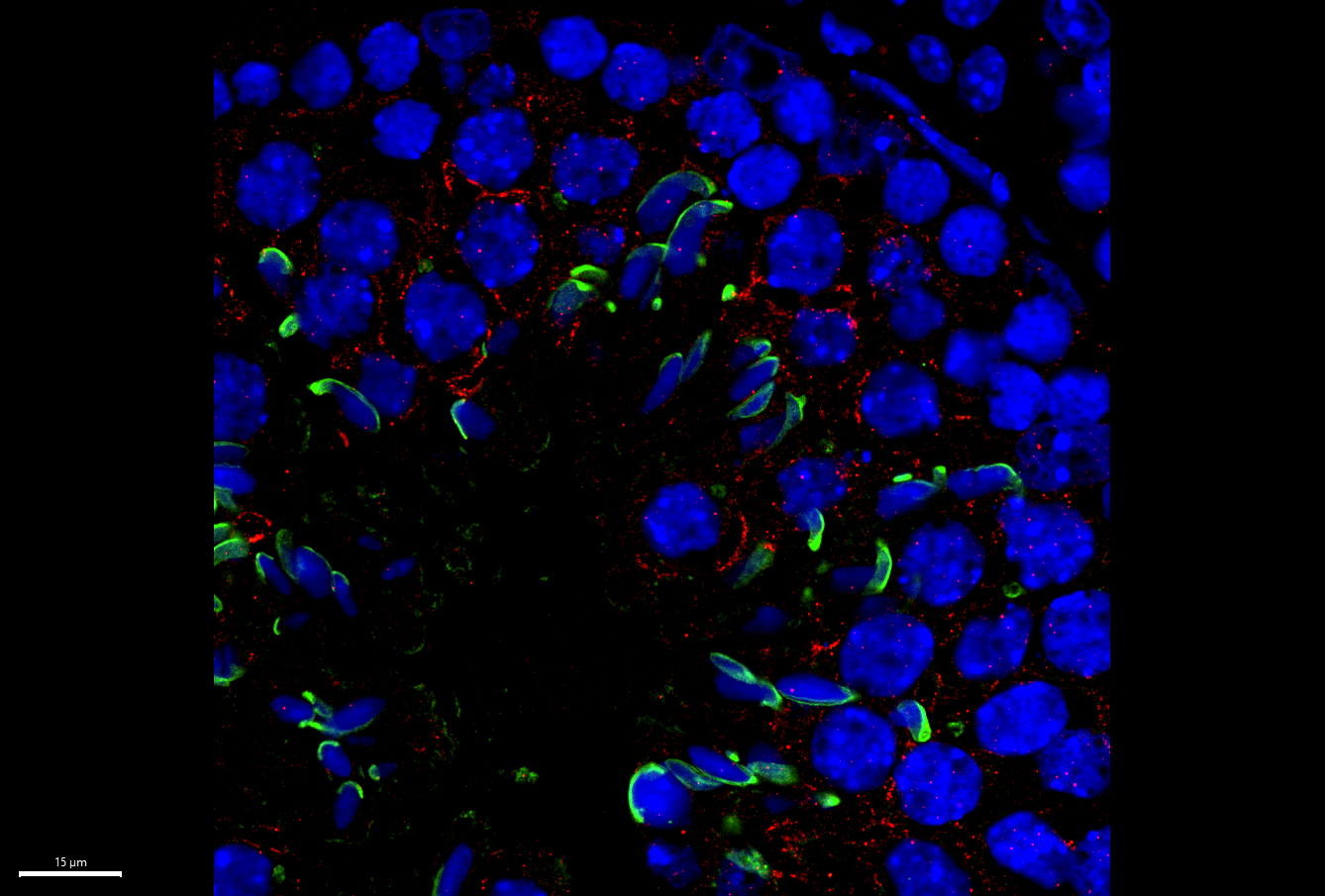

Supplement: Figure 4—source data 1. [file elife-83129-fig4-data1.zip › Figure4/Source data of Figure4D-4E/HA-100x-1.tif]

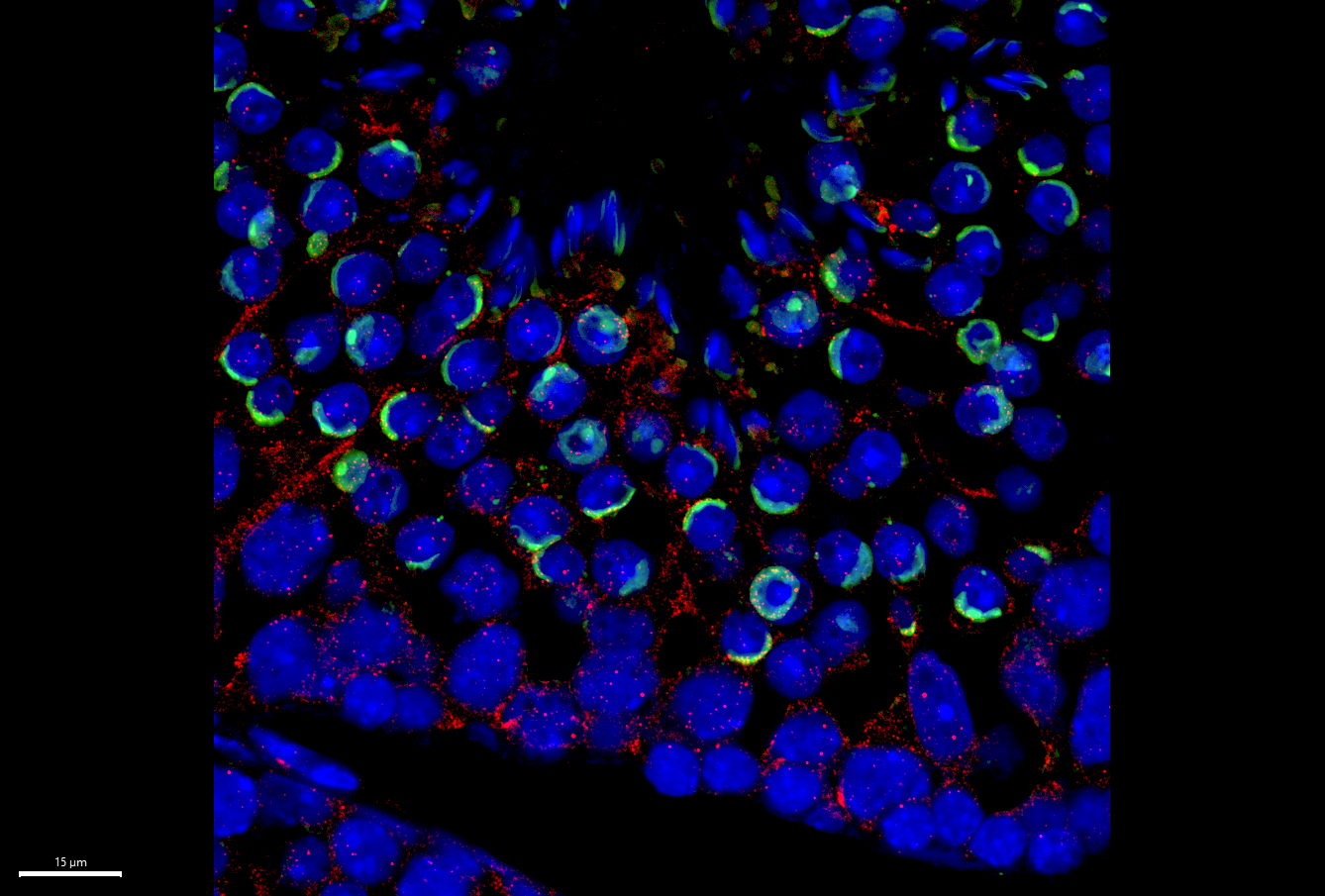

Supplement: Figure 4—source data 1. [file elife-83129-fig4-data1.zip › Figure4/Source data of Figure4D-4E/HA-100x-2.tif]

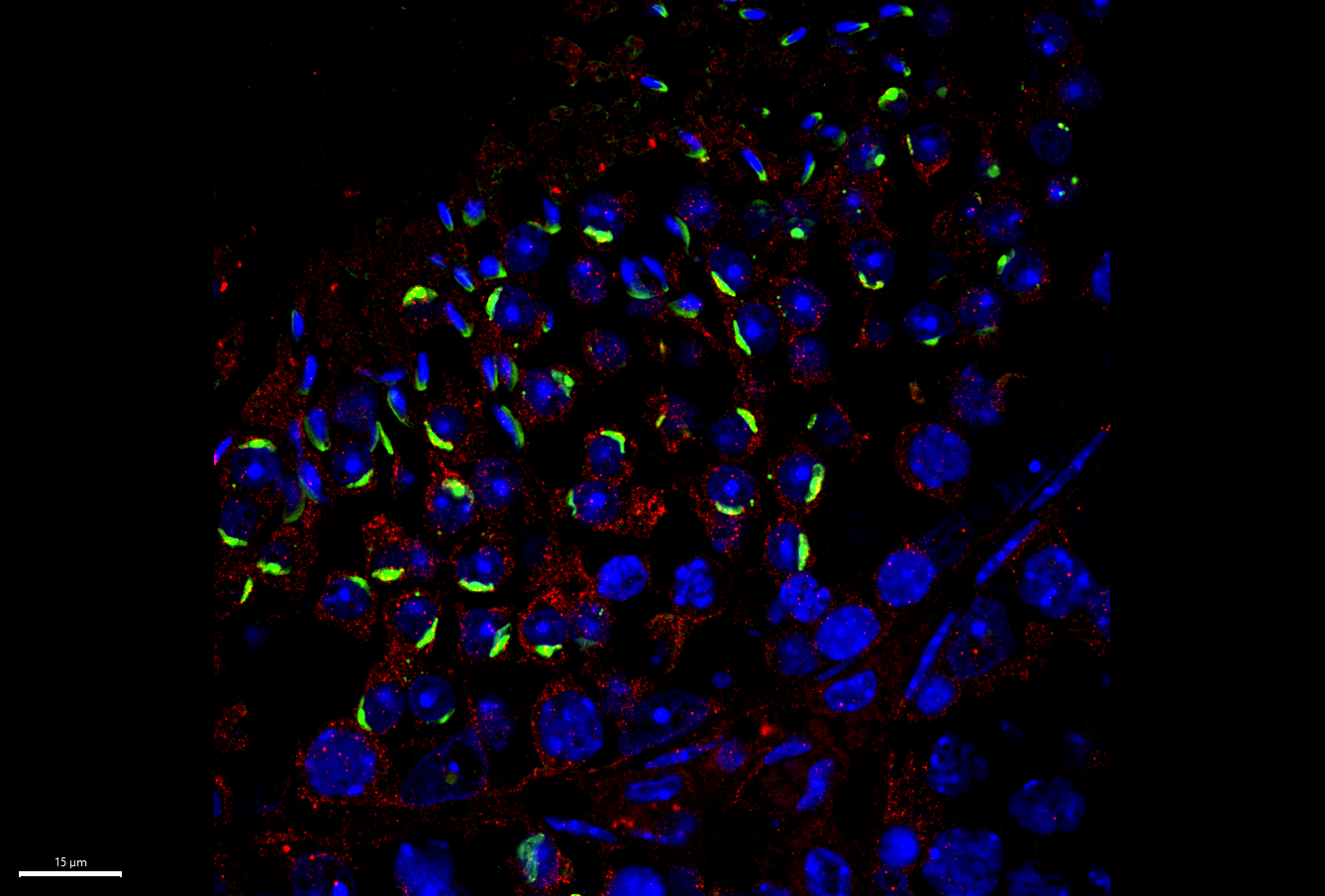

Supplement: Figure 4—source data 1. [file elife-83129-fig4-data1.zip › Figure4/Source data of Figure4D-4E/HA-100x-3.tif]

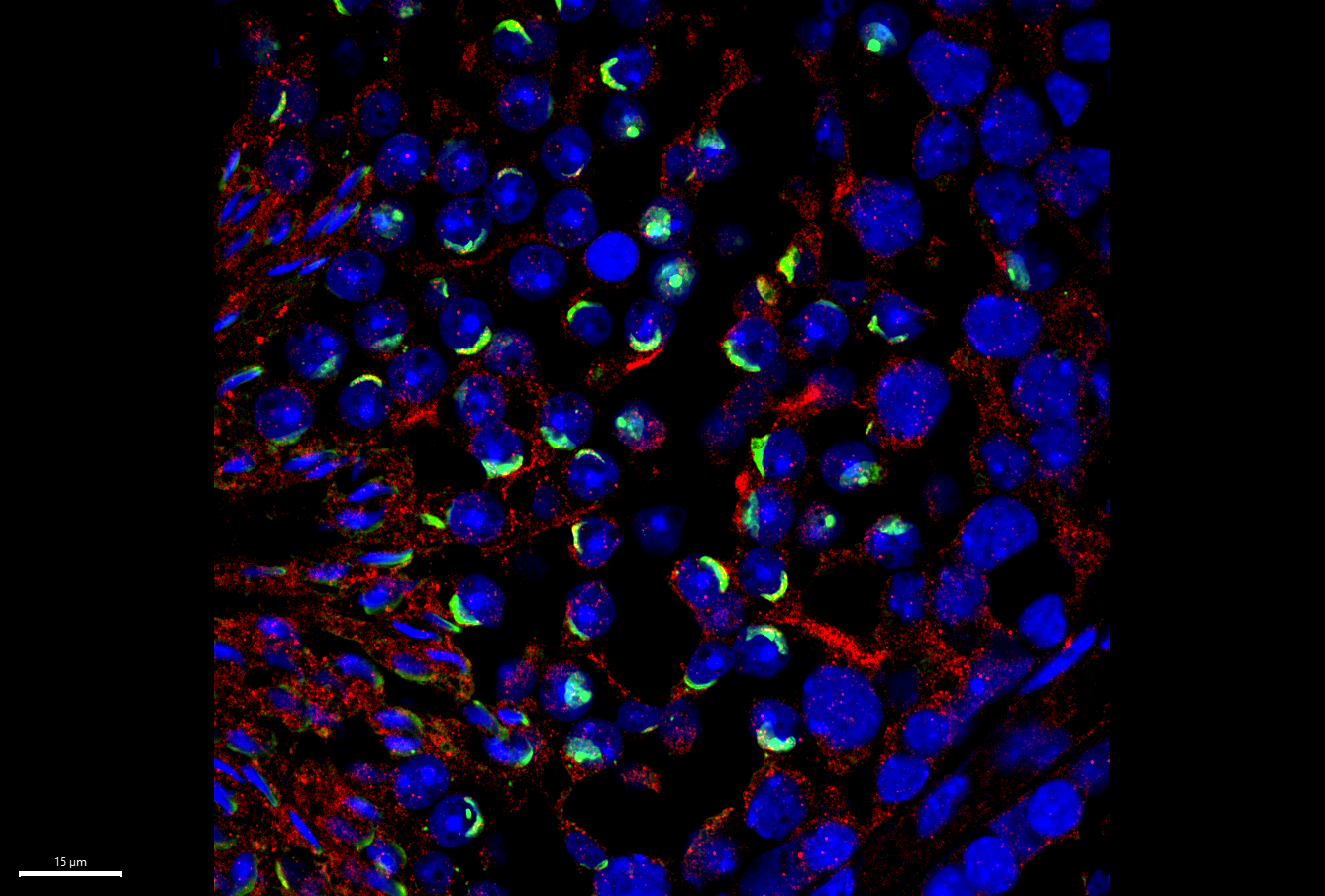

Supplement: Figure 4—source data 1. [file elife-83129-fig4-data1.zip › Figure4/Source data of Figure4D-4E/HA-100x-4.tif]

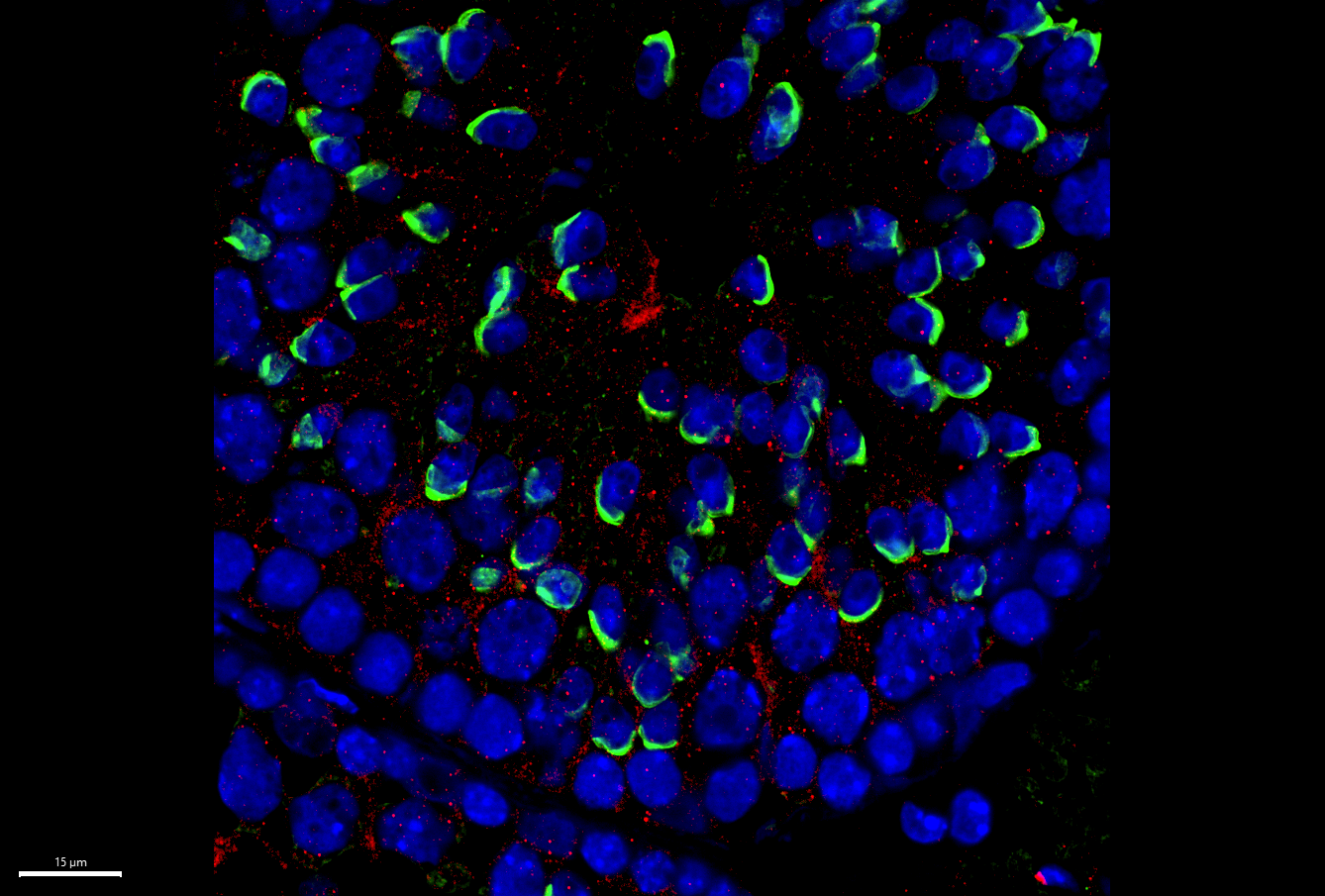

Supplement: Figure 4—source data 1. [file elife-83129-fig4-data1.zip › Figure4/Source data of Figure4D-4E/HA-100x-5.tif]

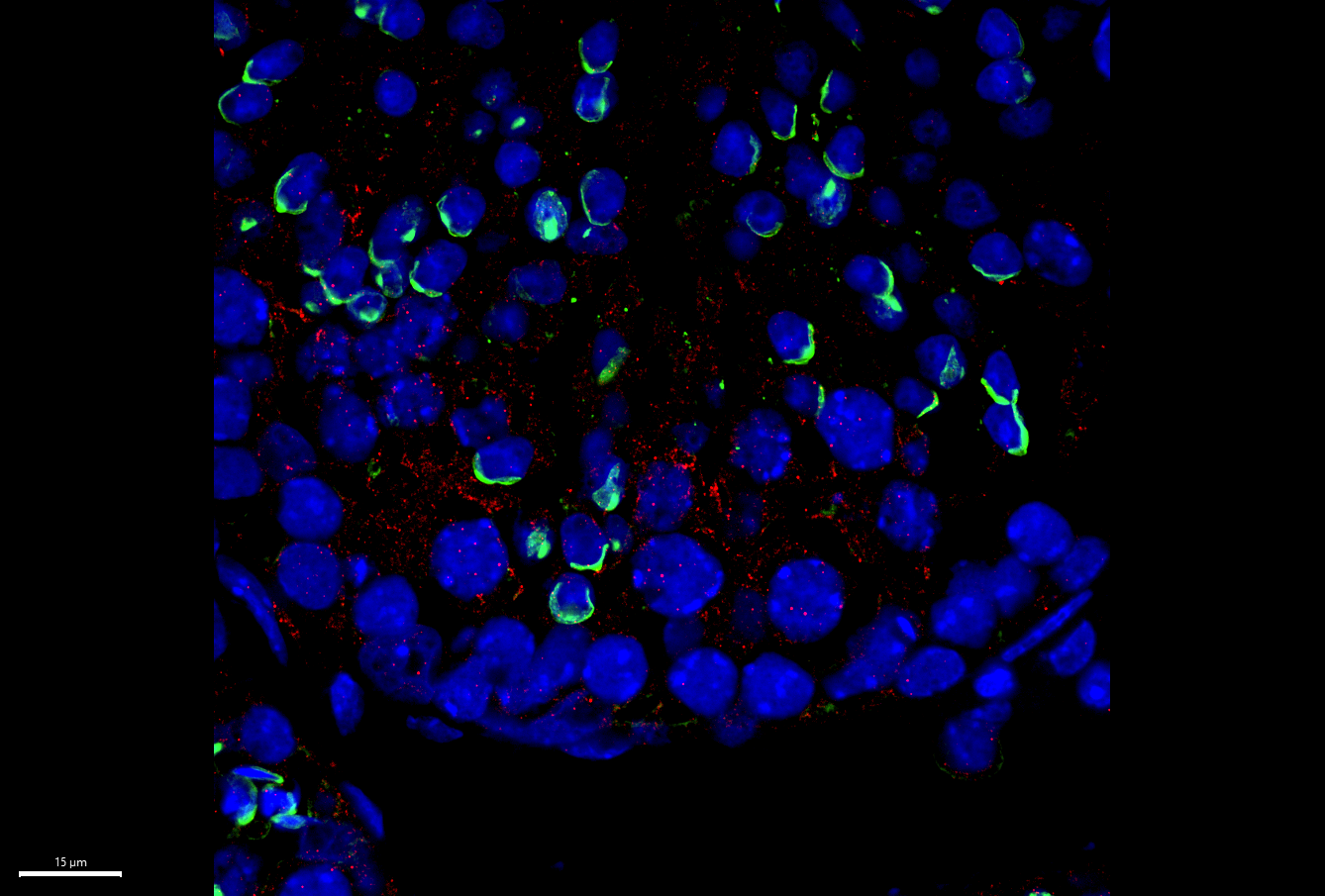

Supplement: Figure 4—source data 1. [file elife-83129-fig4-data1.zip › Figure4/Source data of Figure4D-4E/HA-100x-6.tif]

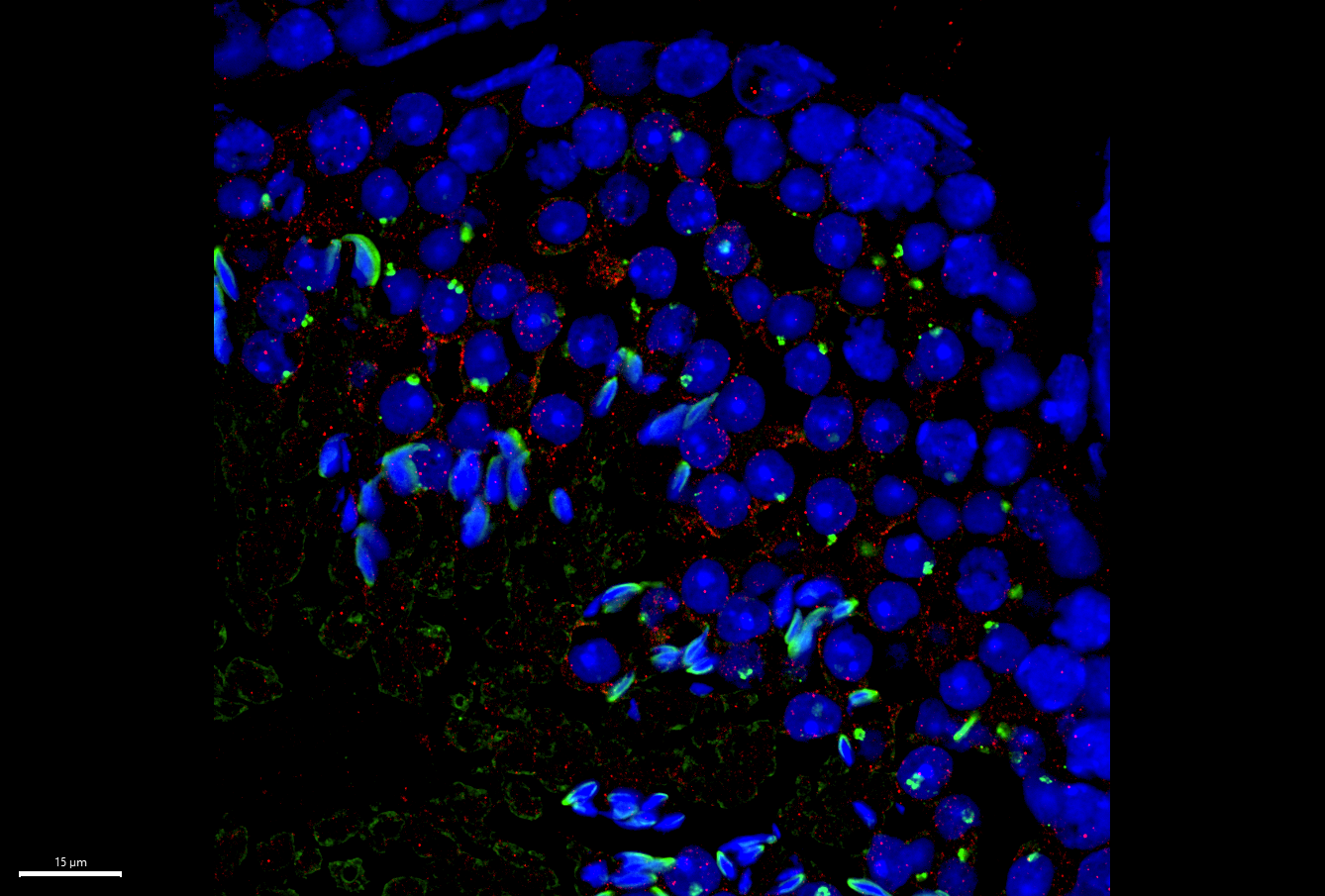

Supplement: Figure 4—source data 1. [file elife-83129-fig4-data1.zip › Figure4/Source data of Figure4D-4E/HA-100x-7.tif]

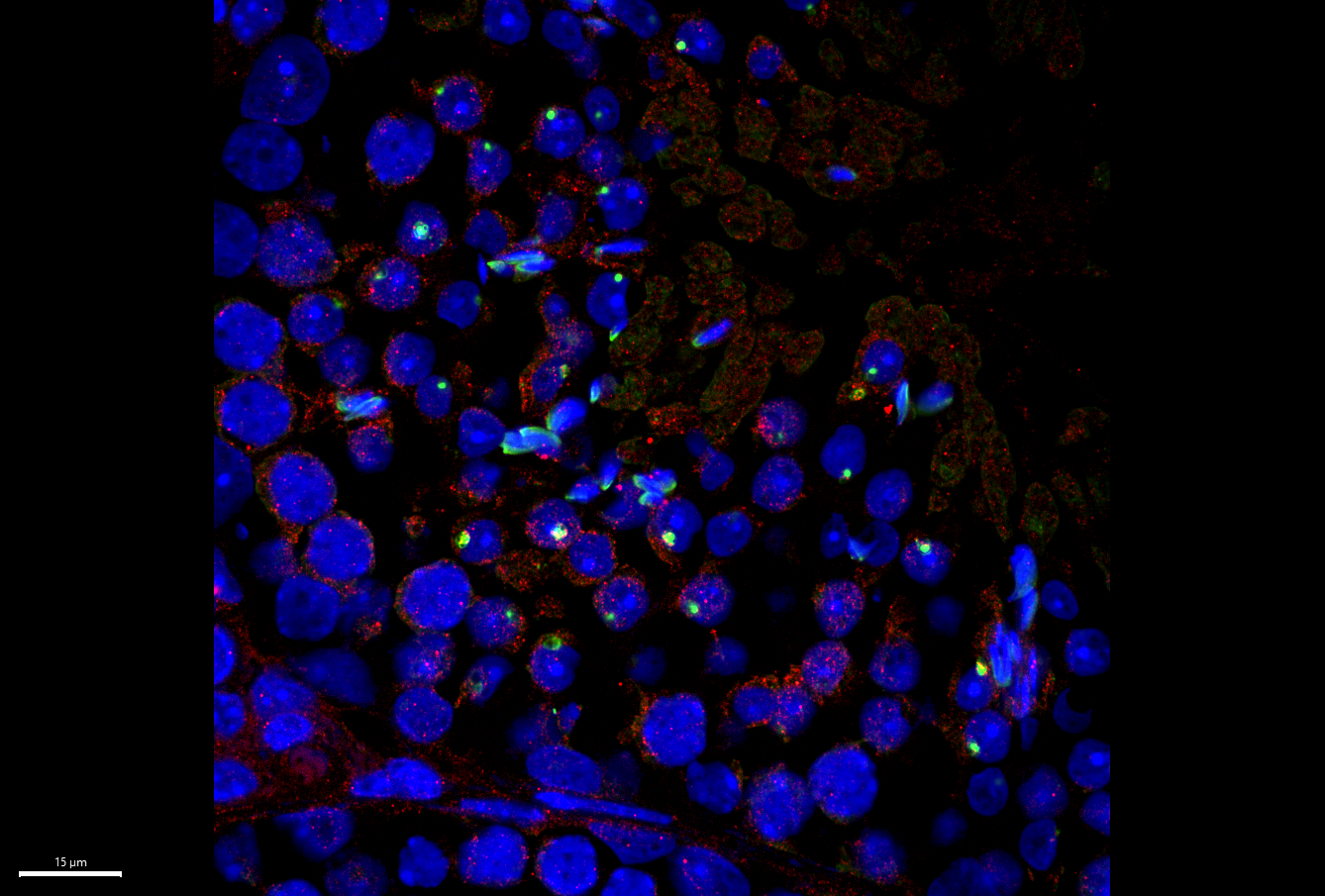

Supplement: Figure 4—source data 1. [file elife-83129-fig4-data1.zip › Figure4/Source data of Figure4D-4E/HA-100x-8.tif]
