# Supplementary figures and images for: The Slingshot phosphatase 2 is required for acrosome biogenesis during spermatogenesis in mice (part 3 of 4)
Source: eLife. 2023 Mar 21;12:e83129. doi: 10.7554/eLife.83129 (PMC10065795; doi:10.7554/eLife.83129)

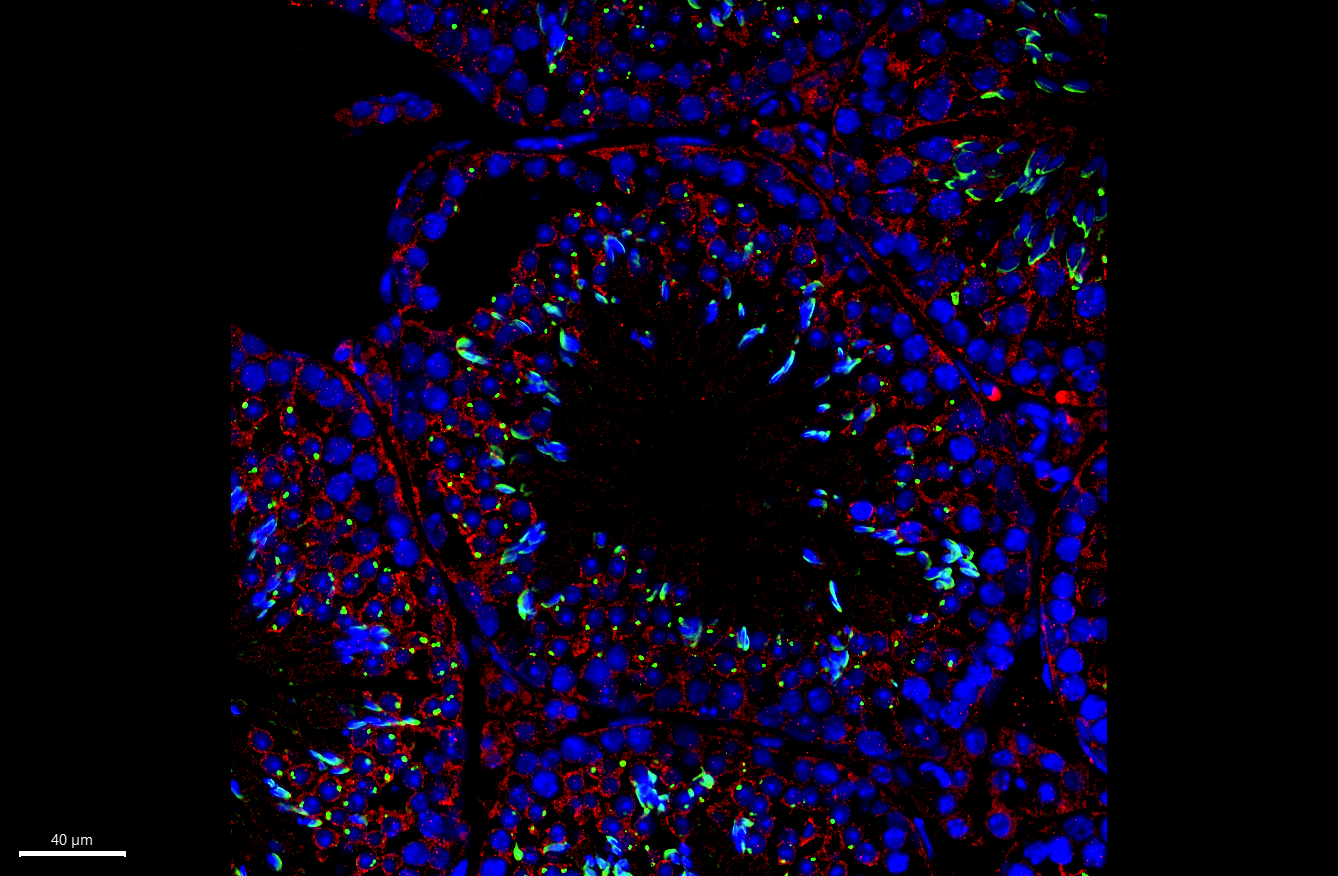

Supplement: Figure 4—source data 1. [file elife-83129-fig4-data1.zip › Figure4/Source data of Figure4D-4E/HA-40X.tif]

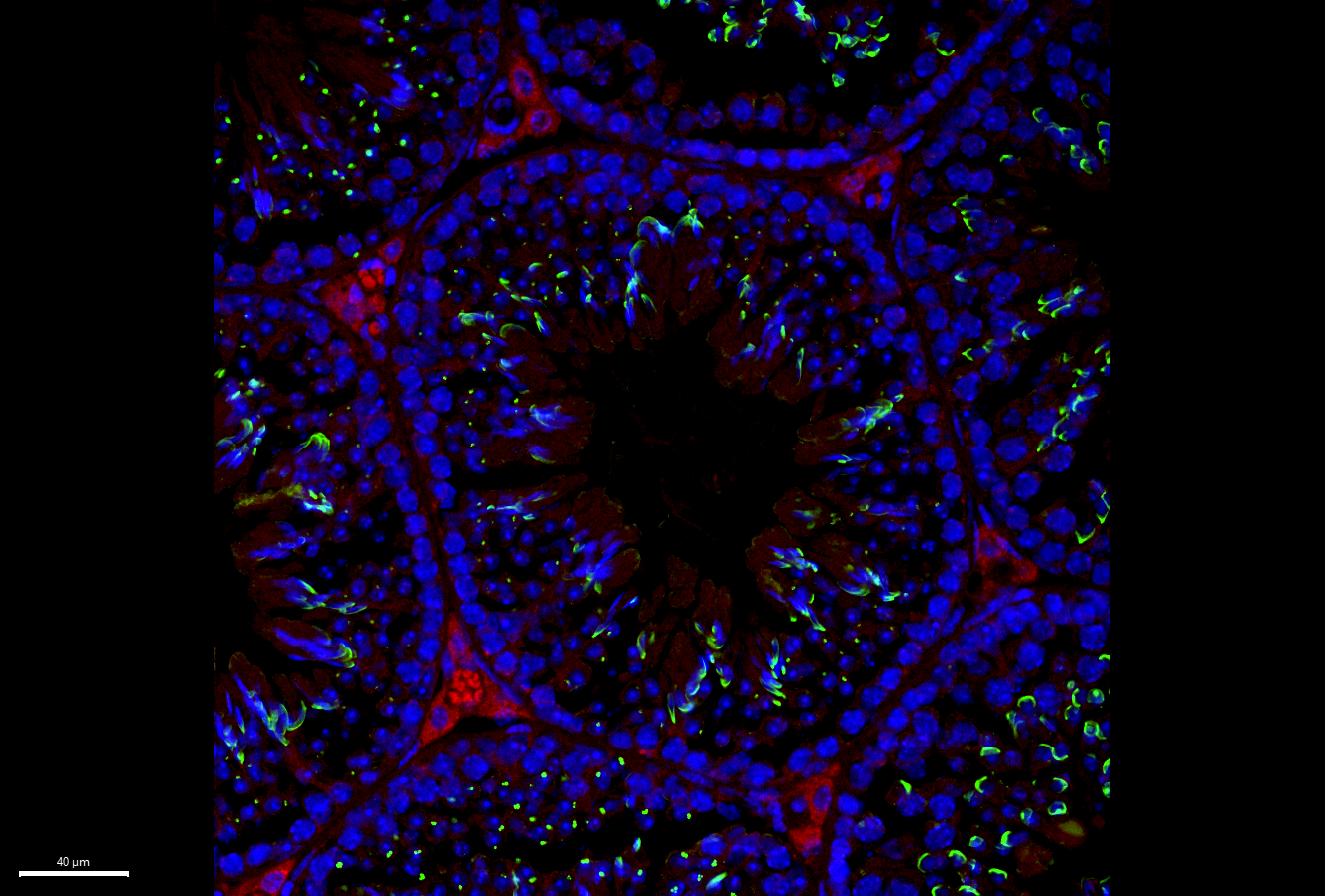

Supplement: Figure 4—source data 1. [file elife-83129-fig4-data1.zip › Figure4/Source data of Figure4D-4E/WT-40x.tif]

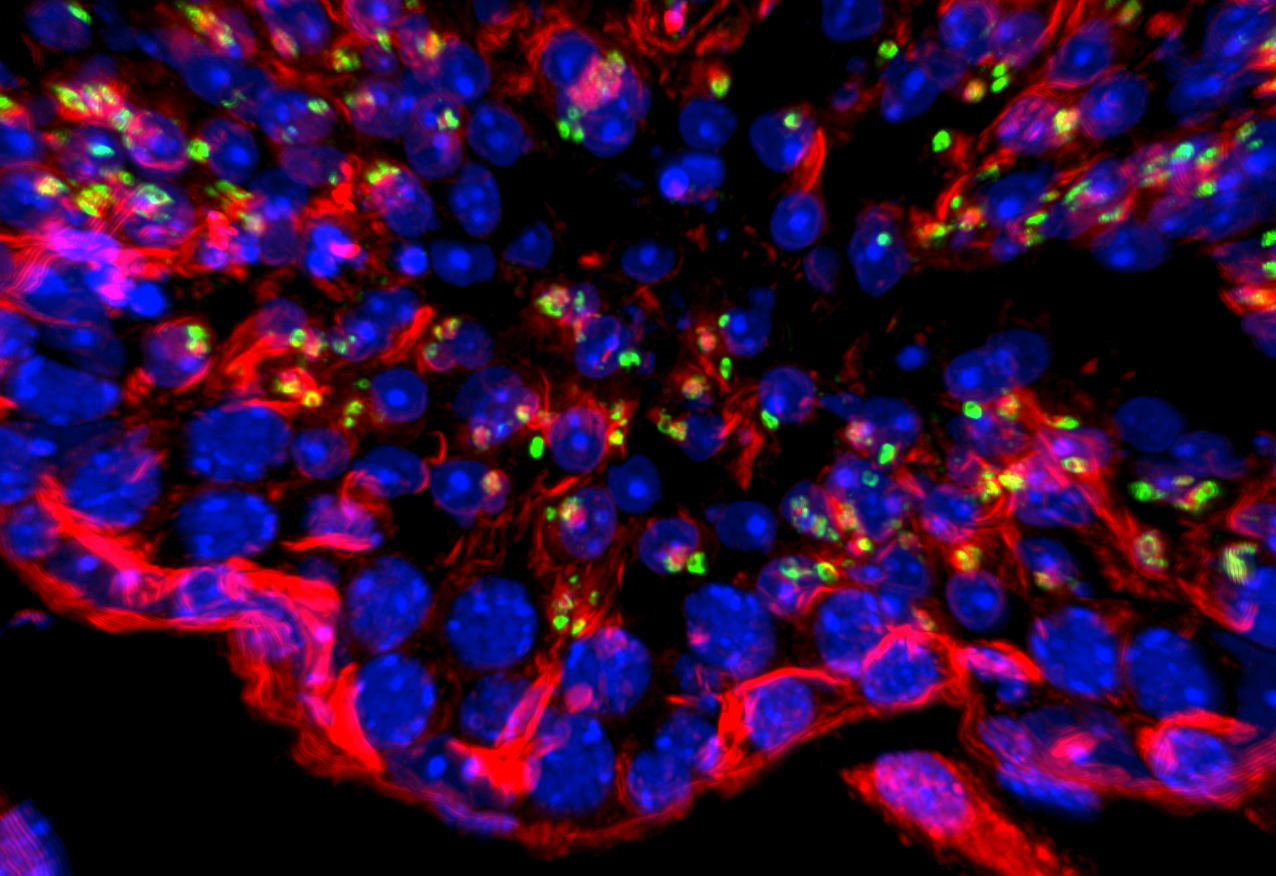

Supplement: Figure 5—source data 1. [file elife-83129-fig5-data1.zip › Figure5/Source data of Figure5A/F-actin+Lectin/HS-PD65-KO1.tif]

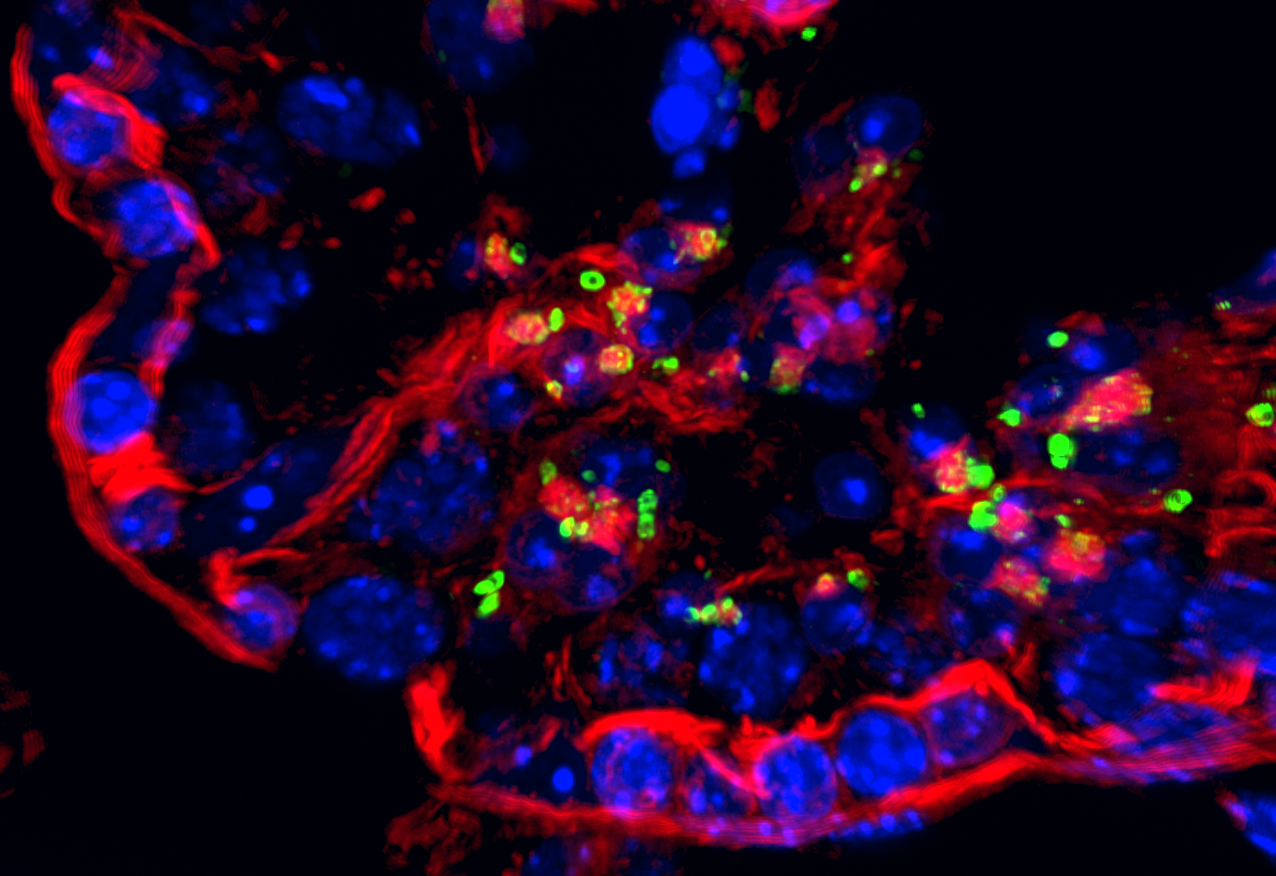

Supplement: Figure 5—source data 1. [file elife-83129-fig5-data1.zip › Figure5/Source data of Figure5A/F-actin+Lectin/HS-PD65-KO10.tif]

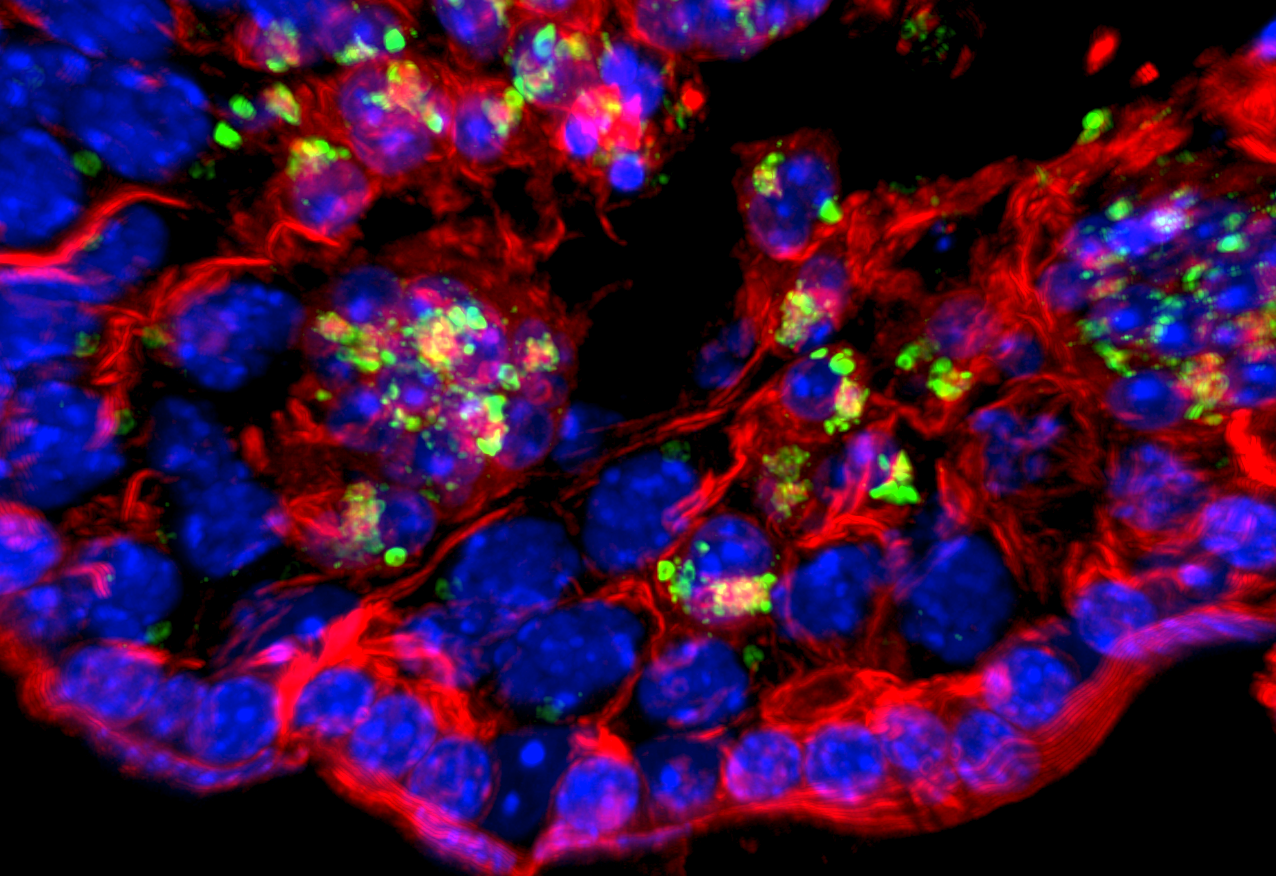

Supplement: Figure 5—source data 1. [file elife-83129-fig5-data1.zip › Figure5/Source data of Figure5A/F-actin+Lectin/HS-PD65-KO11.tif]

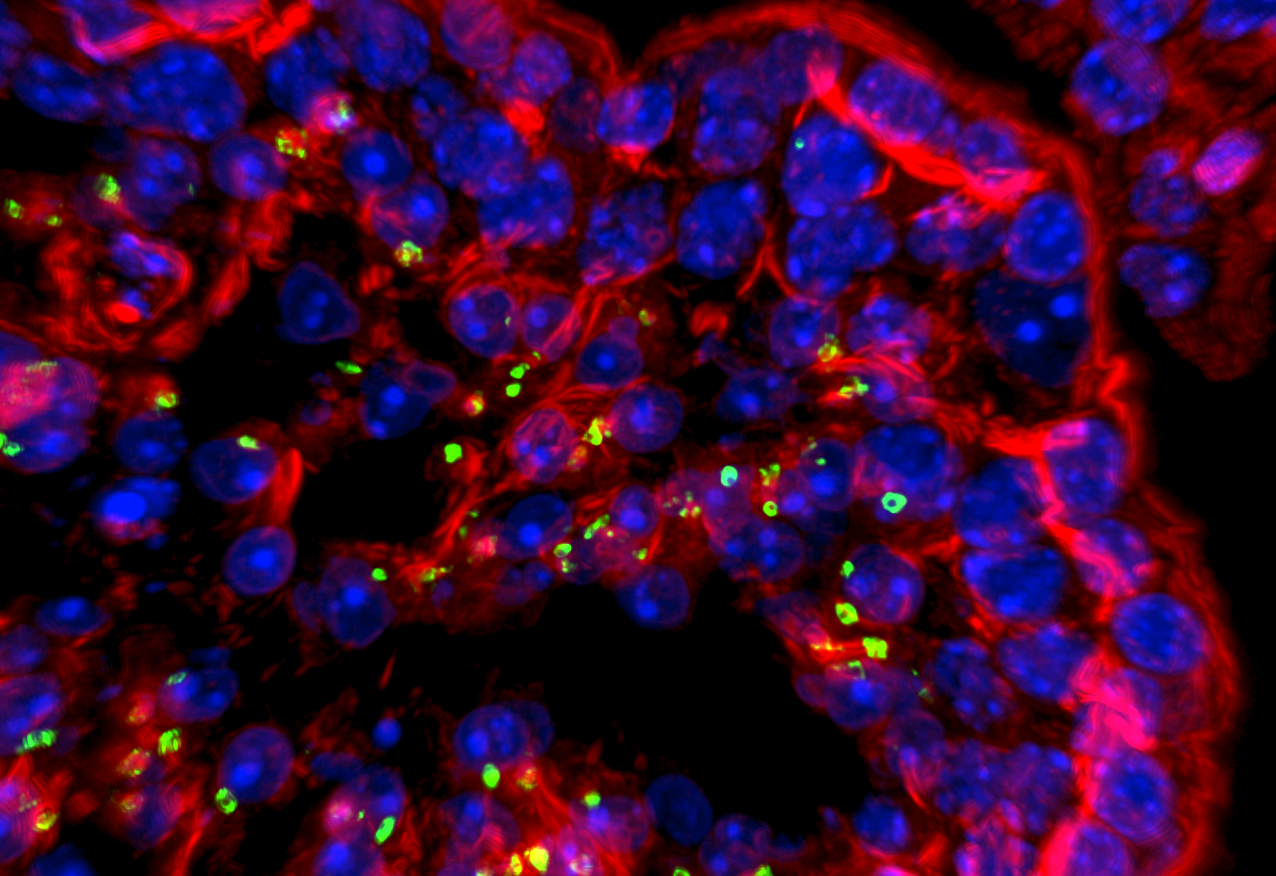

Supplement: Figure 5—source data 1. [file elife-83129-fig5-data1.zip › Figure5/Source data of Figure5A/F-actin+Lectin/HS-PD65-KO12.tif]

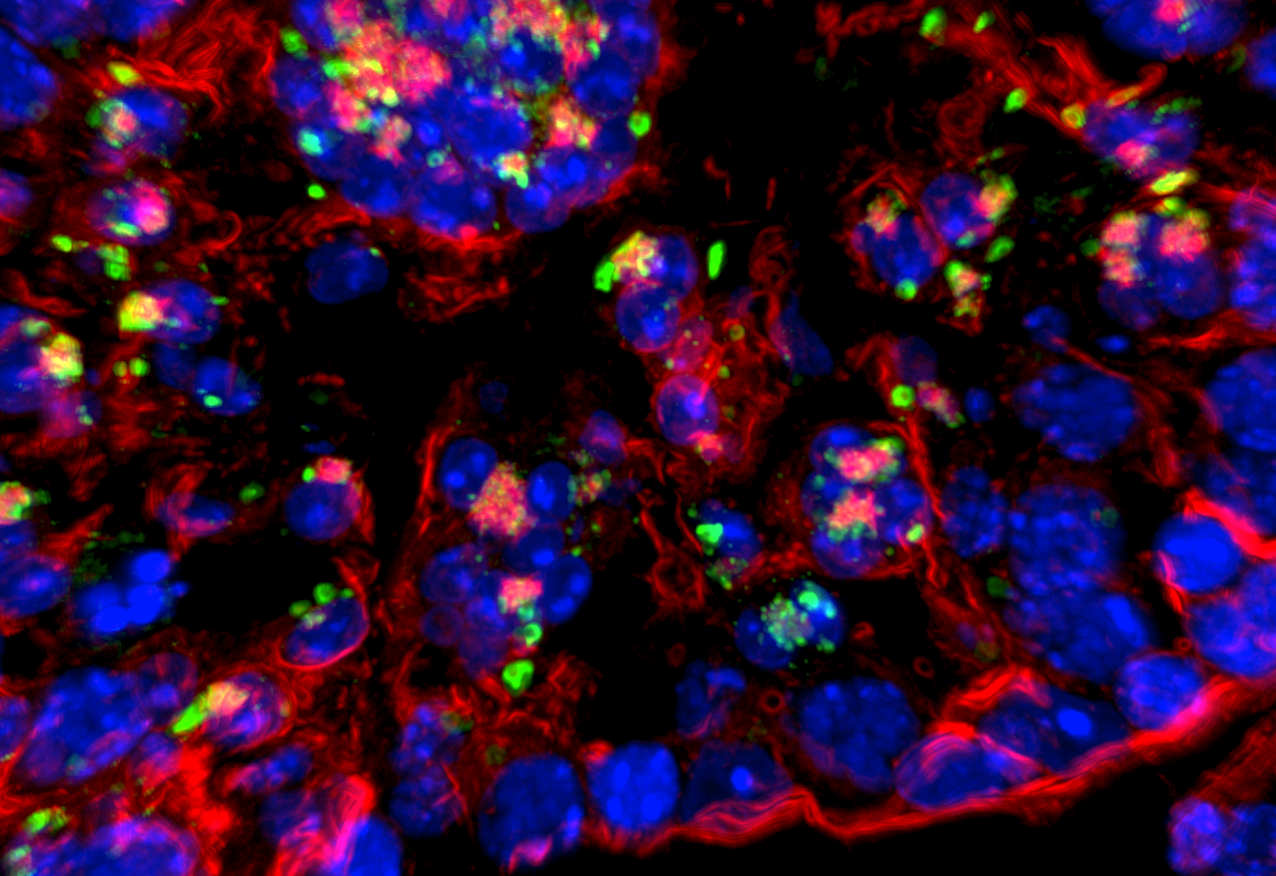

Supplement: Figure 5—source data 1. [file elife-83129-fig5-data1.zip › Figure5/Source data of Figure5A/F-actin+Lectin/HS-PD65-KO13.tif]

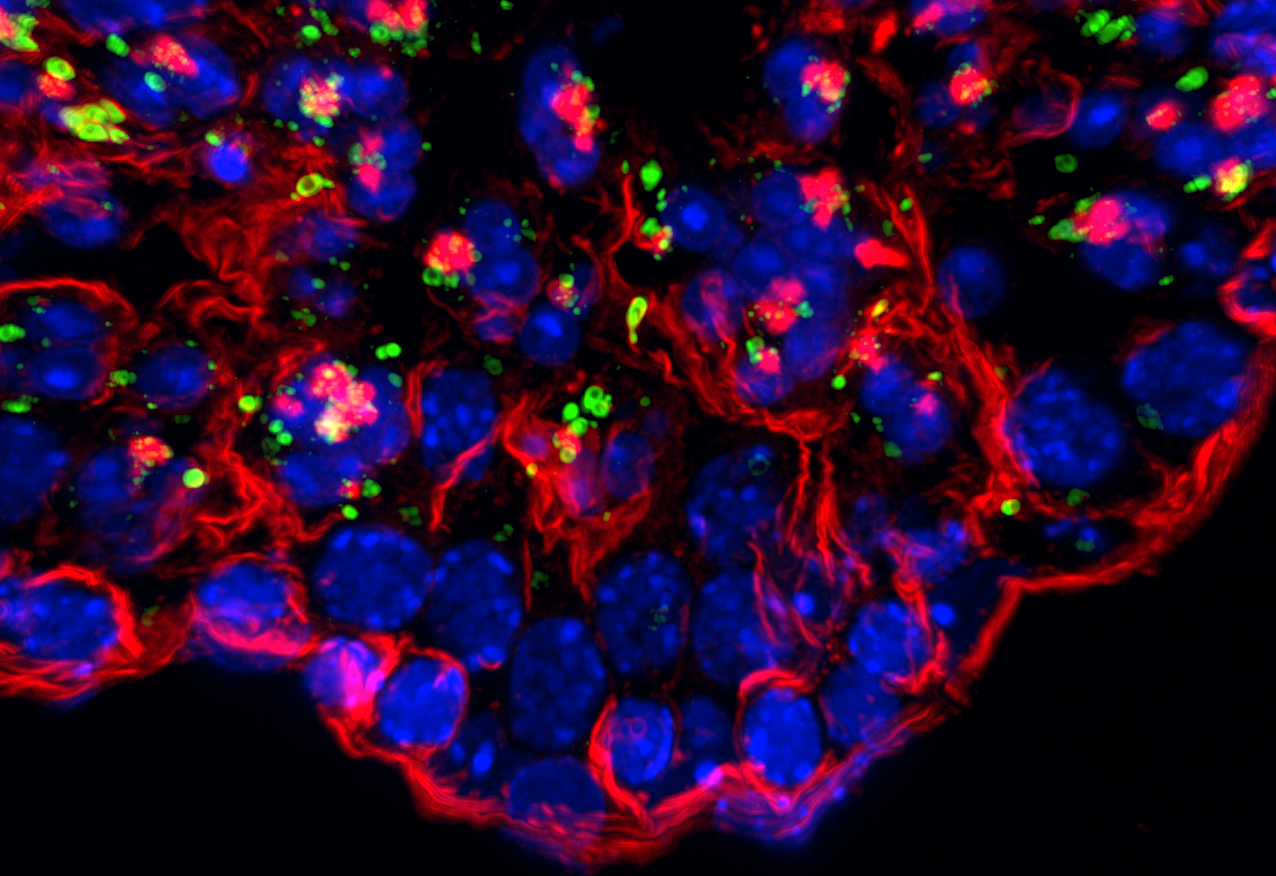

Supplement: Figure 5—source data 1. [file elife-83129-fig5-data1.zip › Figure5/Source data of Figure5A/F-actin+Lectin/HS-PD65-KO14.tif]

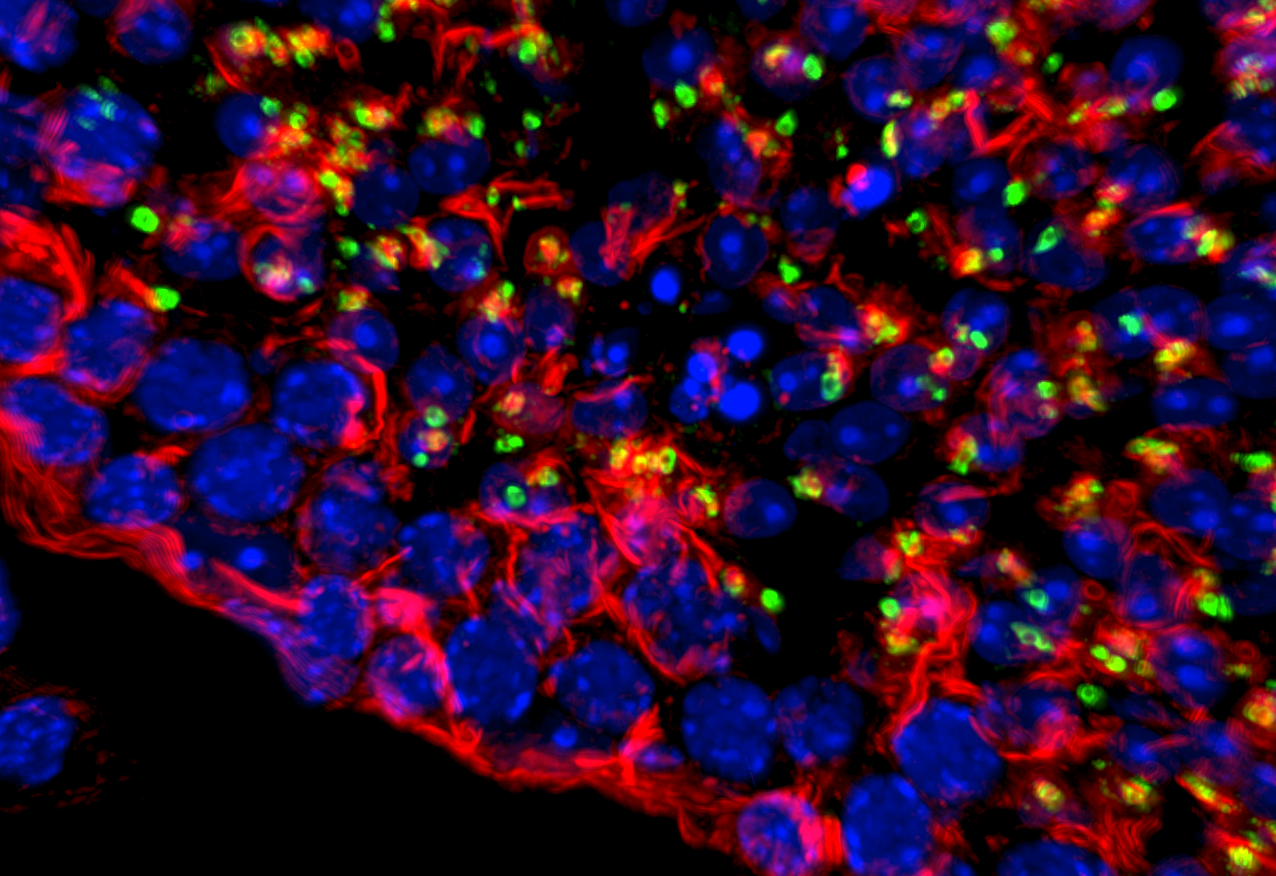

Supplement: Figure 5—source data 1. [file elife-83129-fig5-data1.zip › Figure5/Source data of Figure5A/F-actin+Lectin/HS-PD65-KO15.tif]

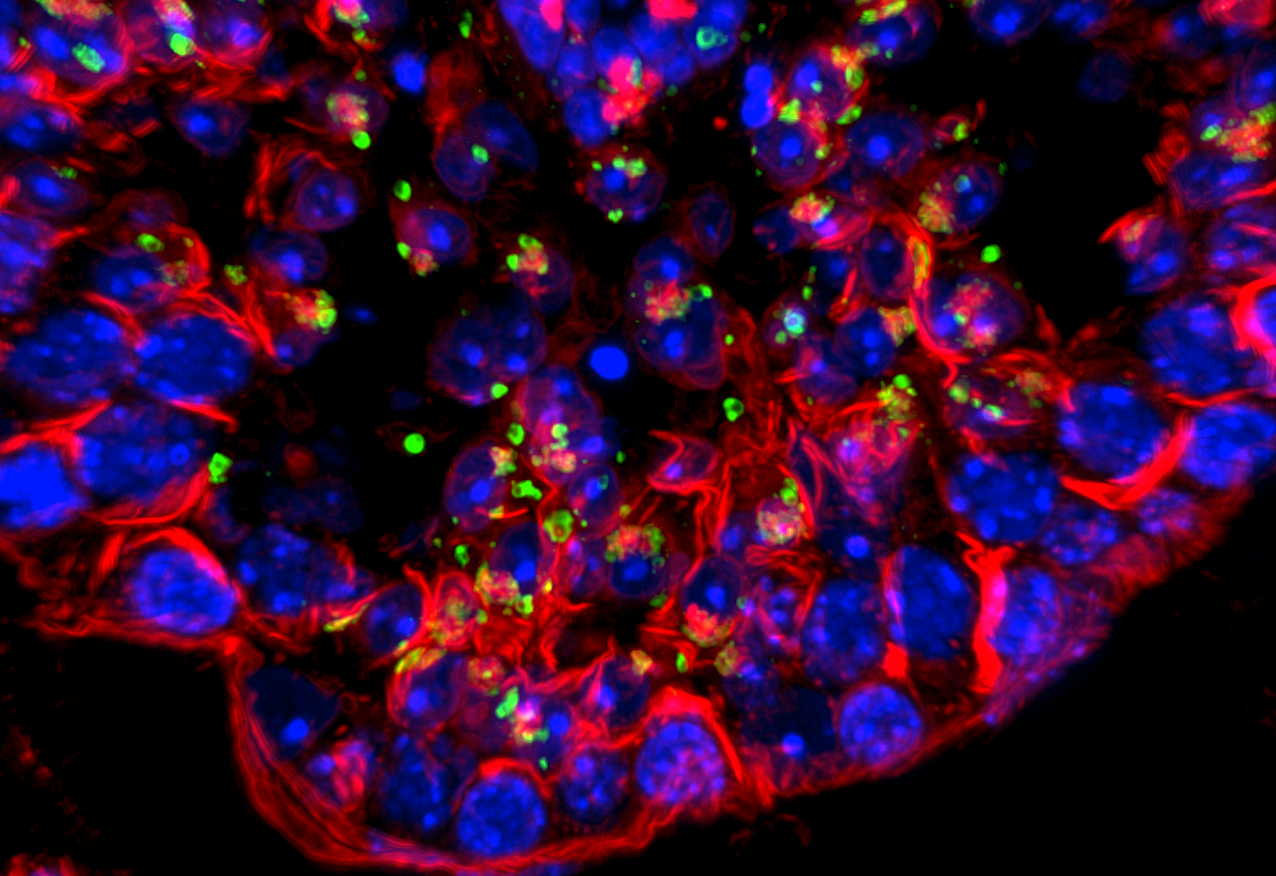

Supplement: Figure 5—source data 1. [file elife-83129-fig5-data1.zip › Figure5/Source data of Figure5A/F-actin+Lectin/HS-PD65-KO16.tif]

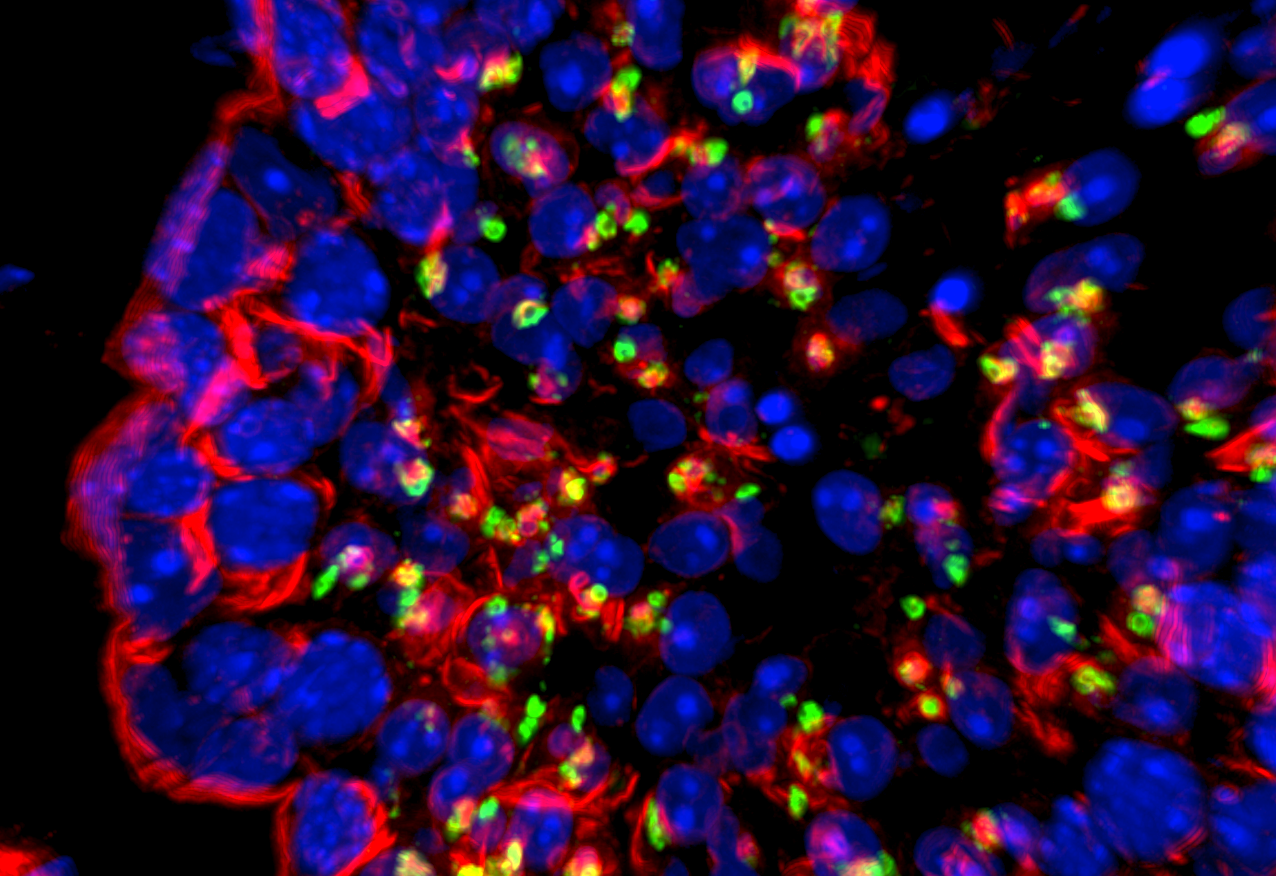

Supplement: Figure 5—source data 1. [file elife-83129-fig5-data1.zip › Figure5/Source data of Figure5A/F-actin+Lectin/HS-PD65-KO17.tif]

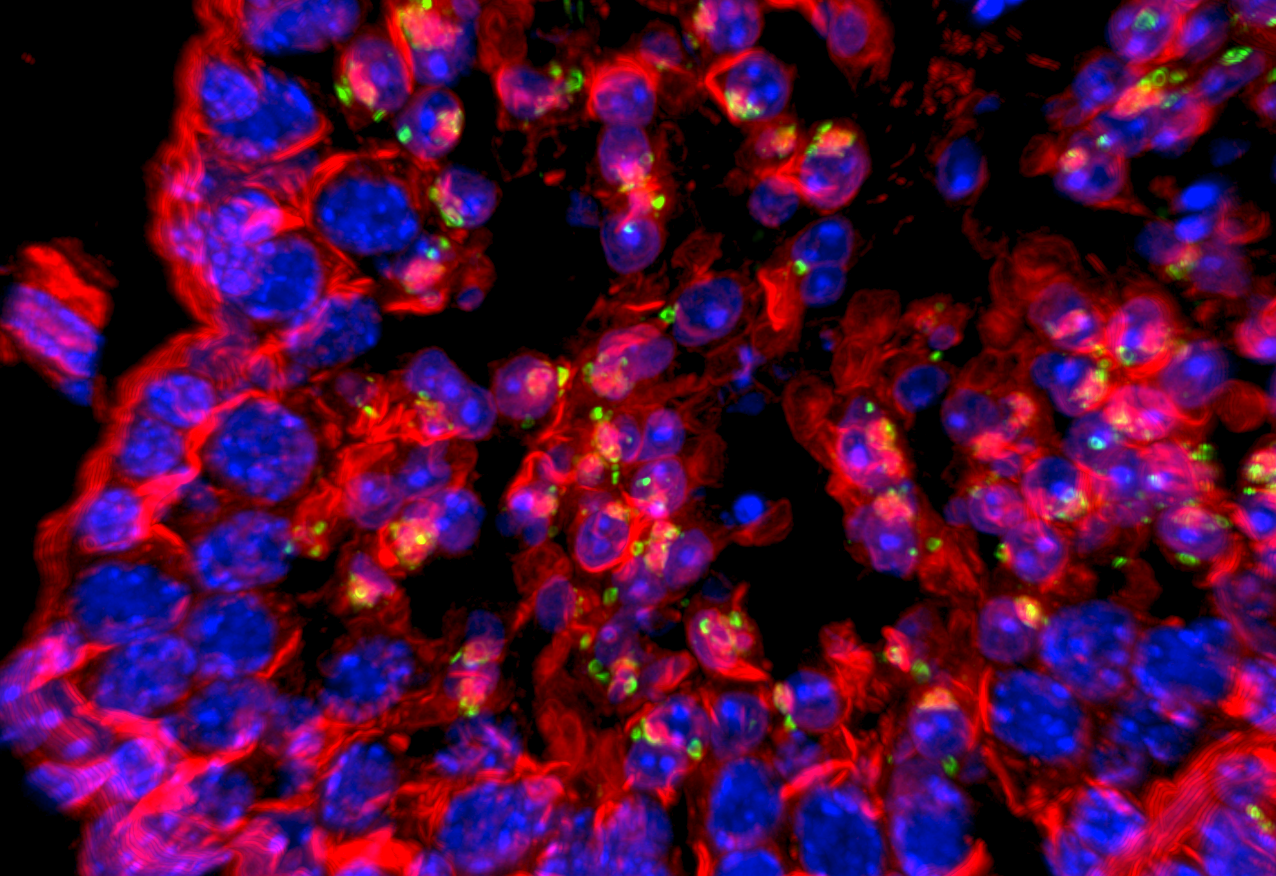

Supplement: Figure 5—source data 1. [file elife-83129-fig5-data1.zip › Figure5/Source data of Figure5A/F-actin+Lectin/HS-PD65-KO18.tif]

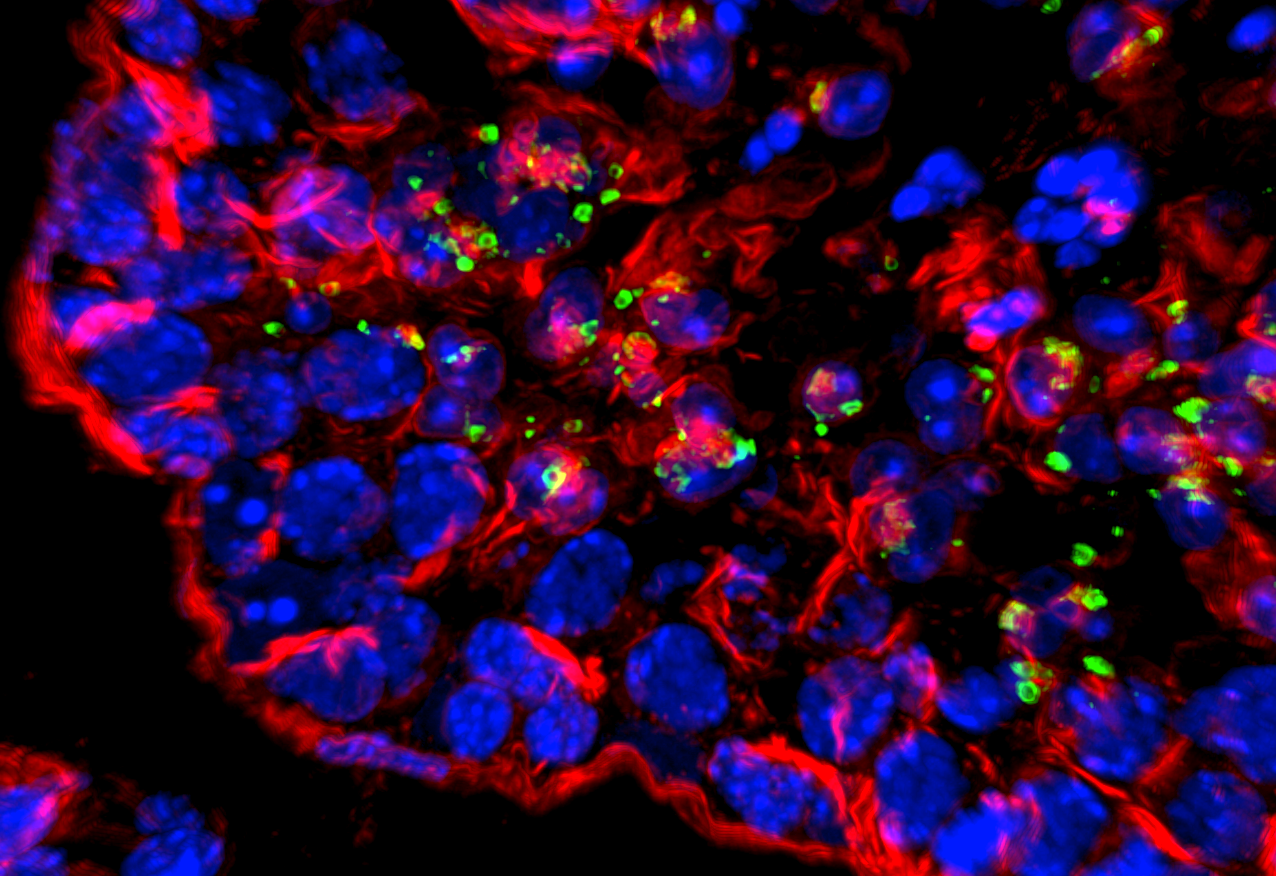

Supplement: Figure 5—source data 1. [file elife-83129-fig5-data1.zip › Figure5/Source data of Figure5A/F-actin+Lectin/HS-PD65-KO19.tif]

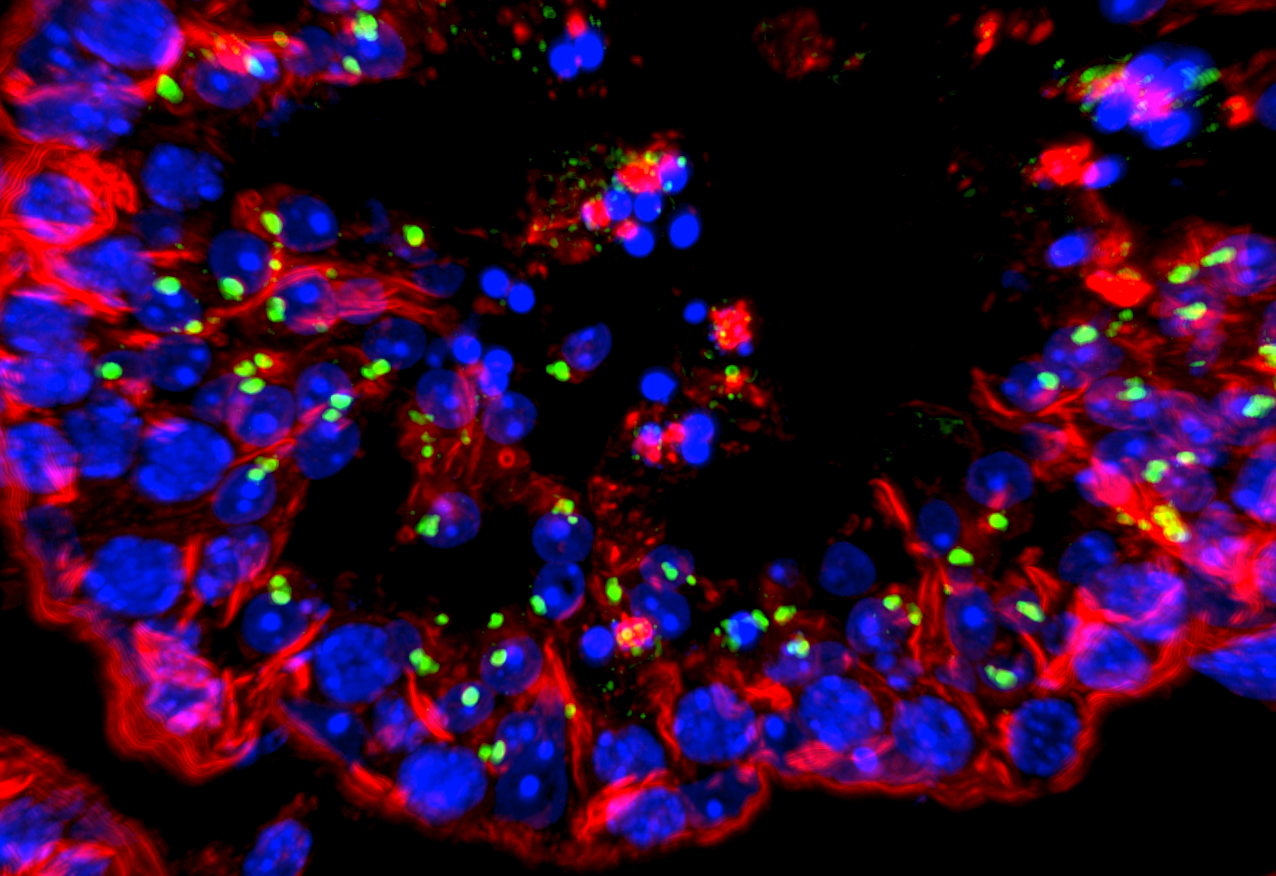

Supplement: Figure 5—source data 1. [file elife-83129-fig5-data1.zip › Figure5/Source data of Figure5A/F-actin+Lectin/HS-PD65-KO2.tif]

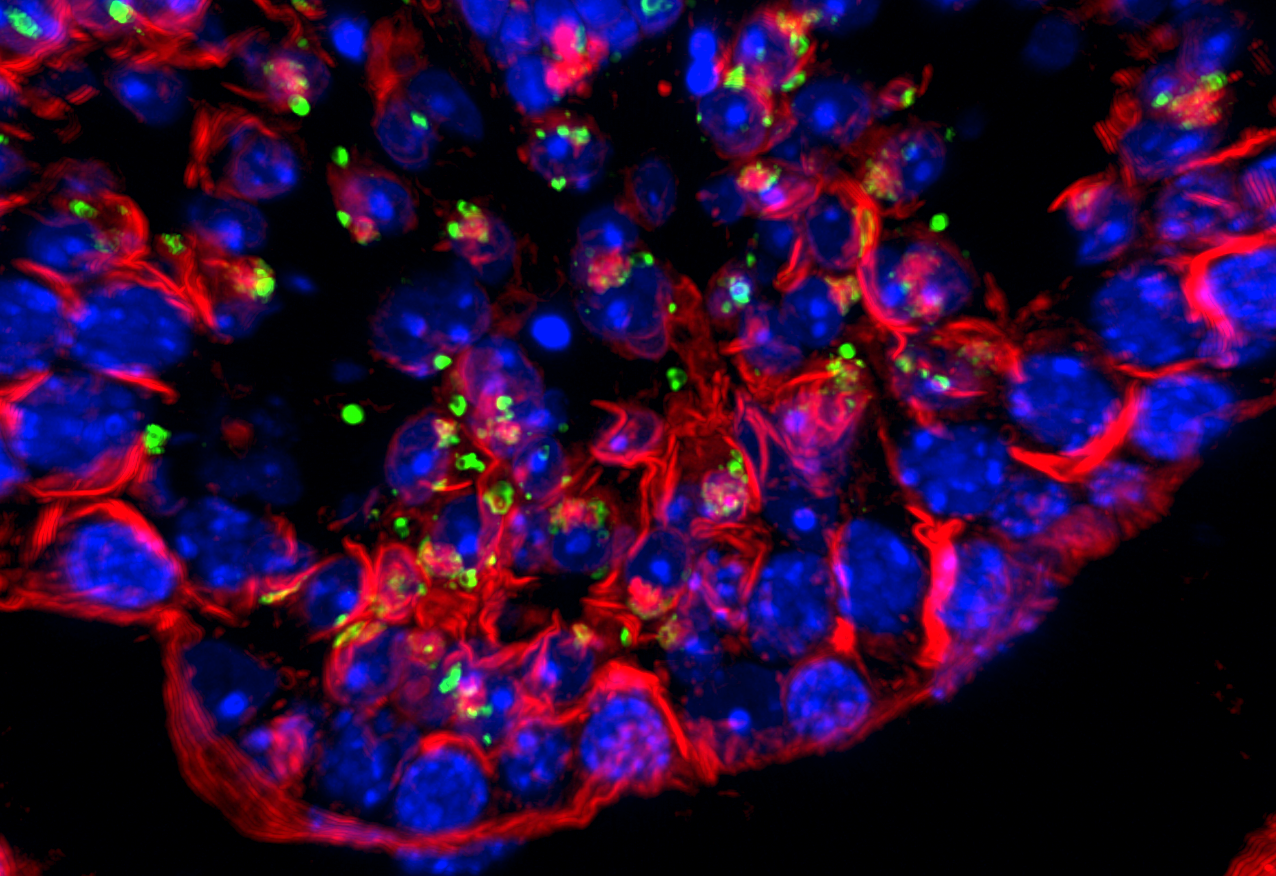

Supplement: Figure 5—source data 1. [file elife-83129-fig5-data1.zip › Figure5/Source data of Figure5A/F-actin+Lectin/HS-PD65-KO20.tif]

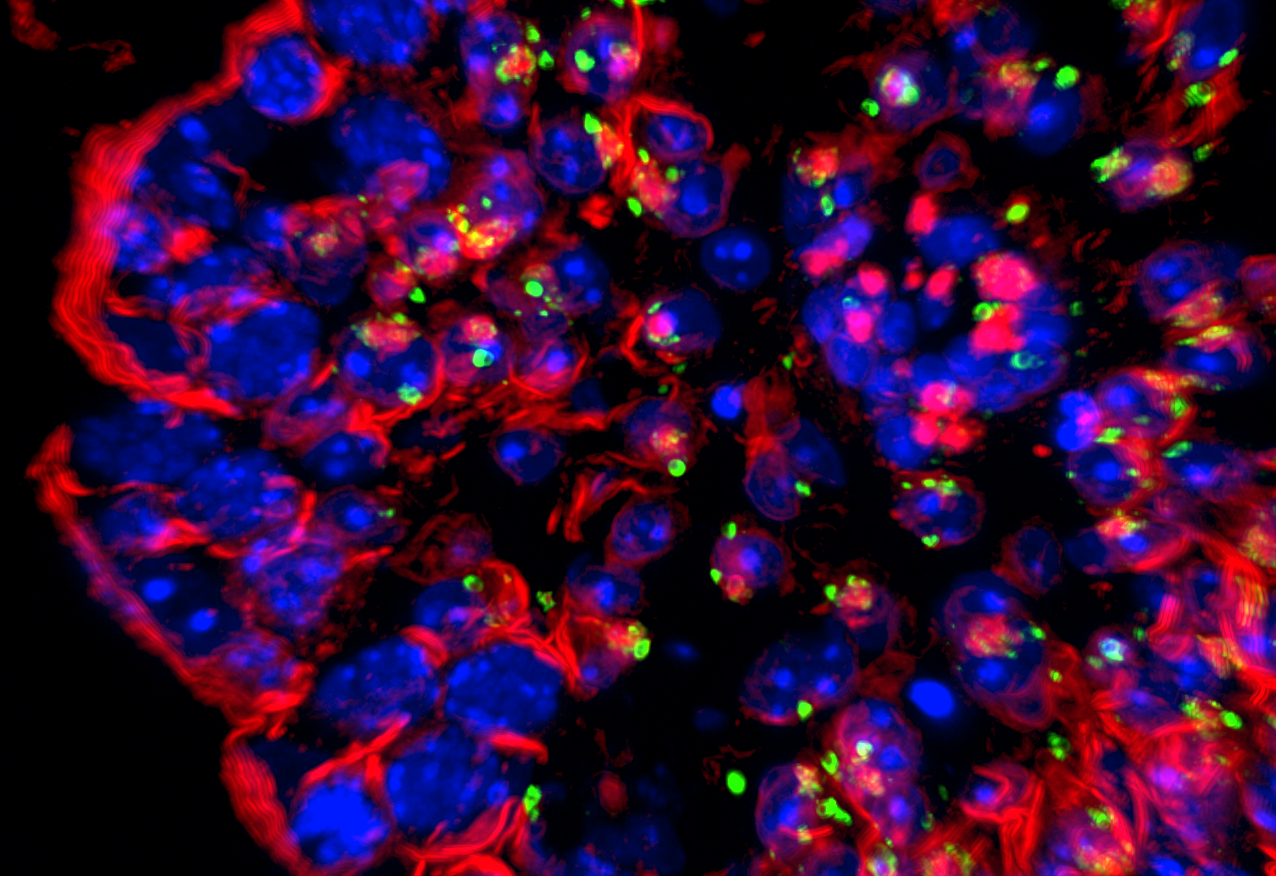

Supplement: Figure 5—source data 1. [file elife-83129-fig5-data1.zip › Figure5/Source data of Figure5A/F-actin+Lectin/HS-PD65-KO21.tif]

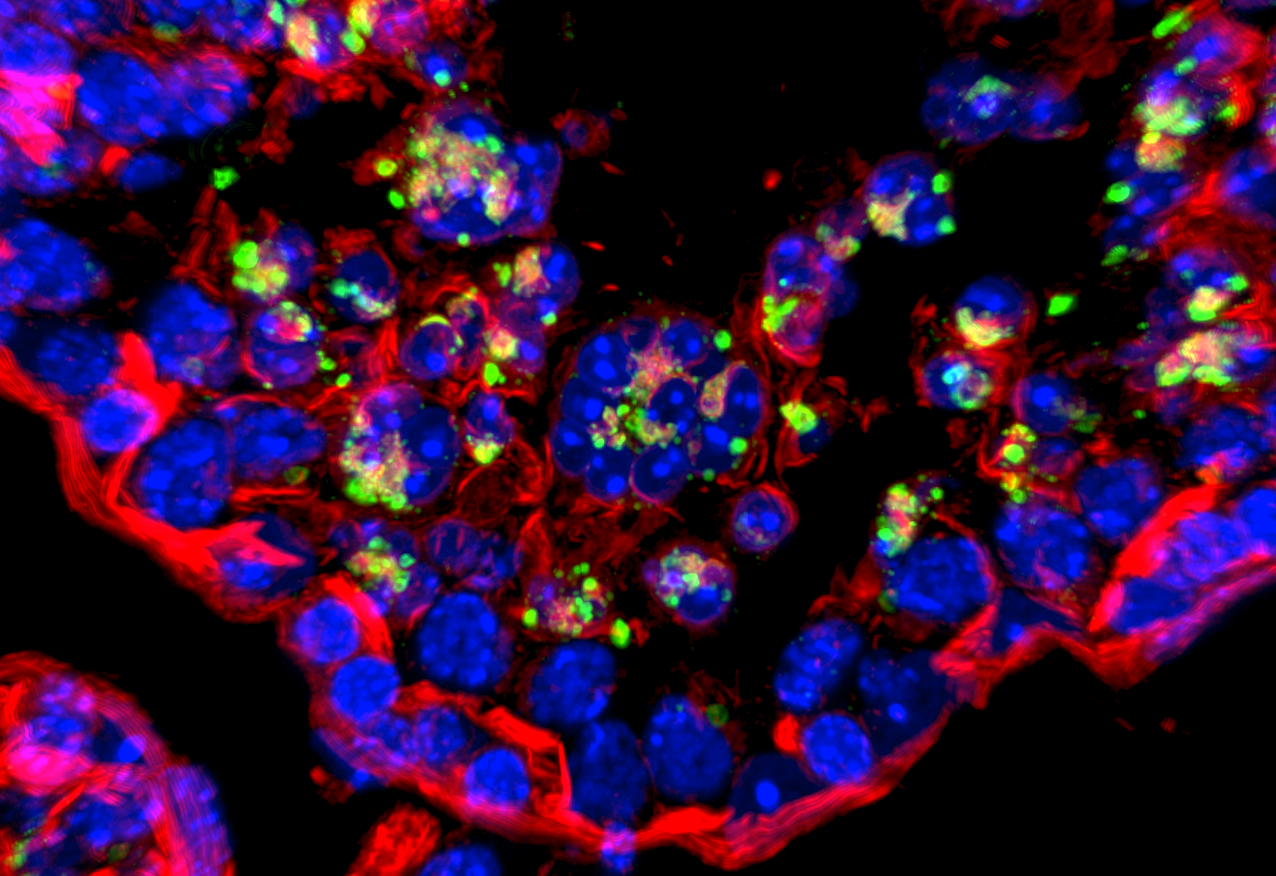

Supplement: Figure 5—source data 1. [file elife-83129-fig5-data1.zip › Figure5/Source data of Figure5A/F-actin+Lectin/HS-PD65-KO3.tif]

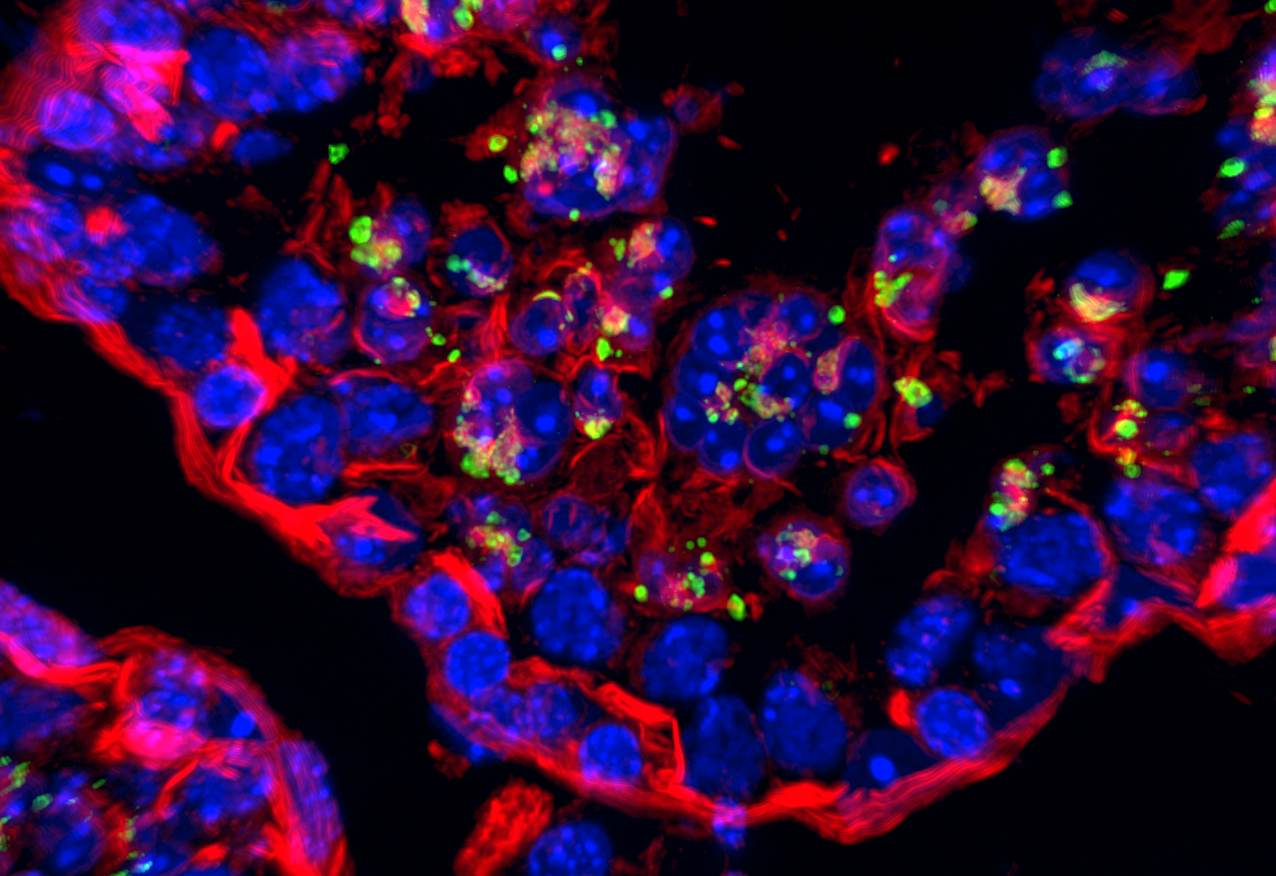

Supplement: Figure 5—source data 1. [file elife-83129-fig5-data1.zip › Figure5/Source data of Figure5A/F-actin+Lectin/HS-PD65-KO4.tif]

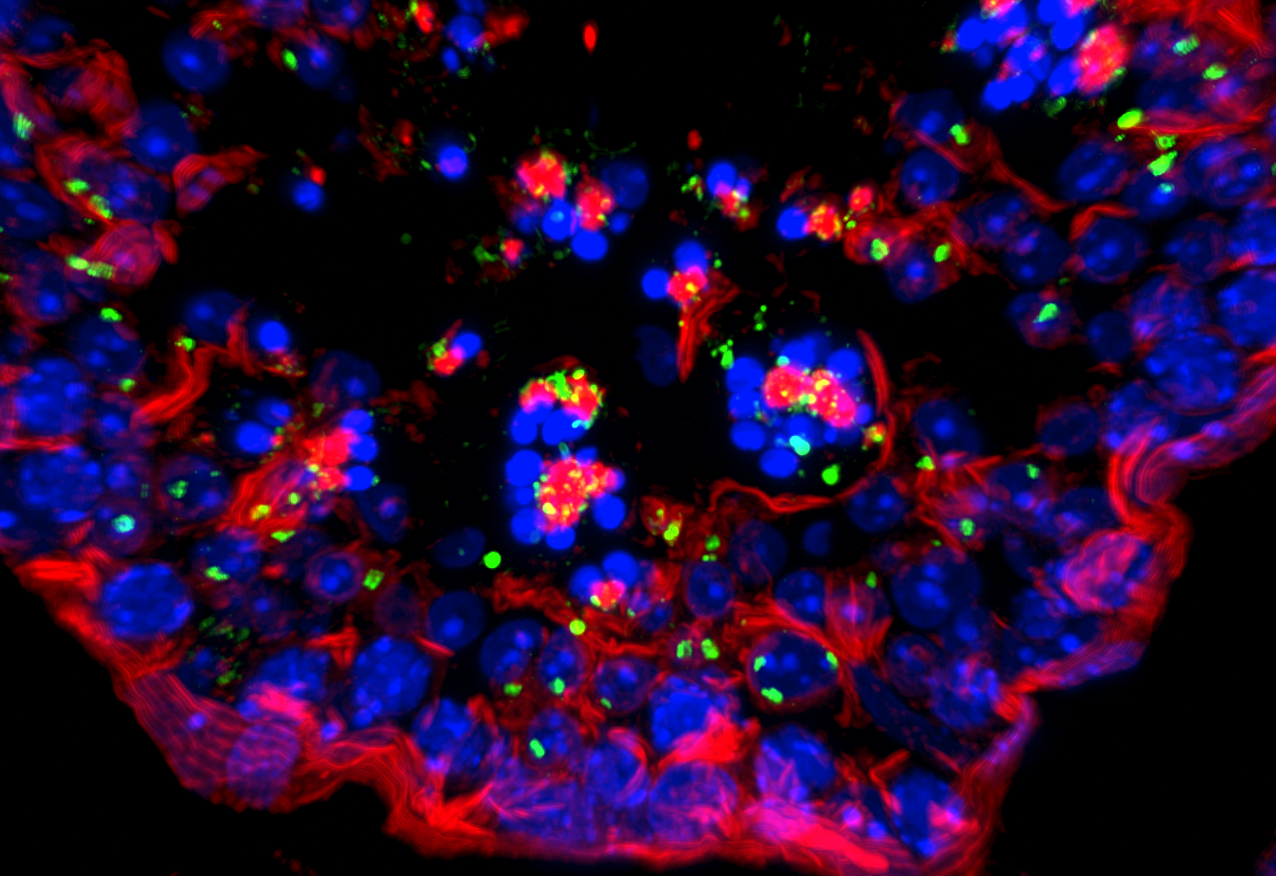

Supplement: Figure 5—source data 1. [file elife-83129-fig5-data1.zip › Figure5/Source data of Figure5A/F-actin+Lectin/HS-PD65-KO5.tif]

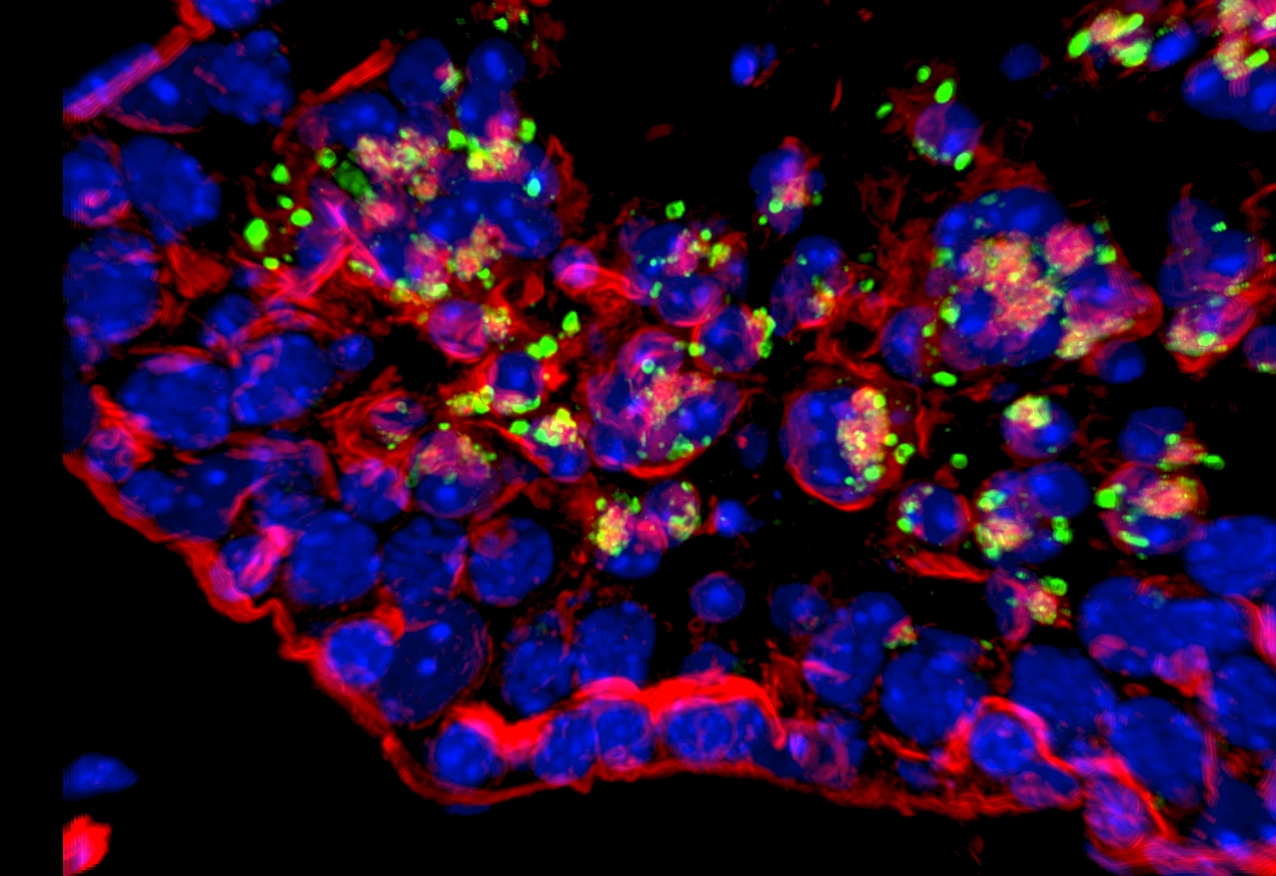

Supplement: Figure 5—source data 1. [file elife-83129-fig5-data1.zip › Figure5/Source data of Figure5A/F-actin+Lectin/HS-PD65-KO6.tif]

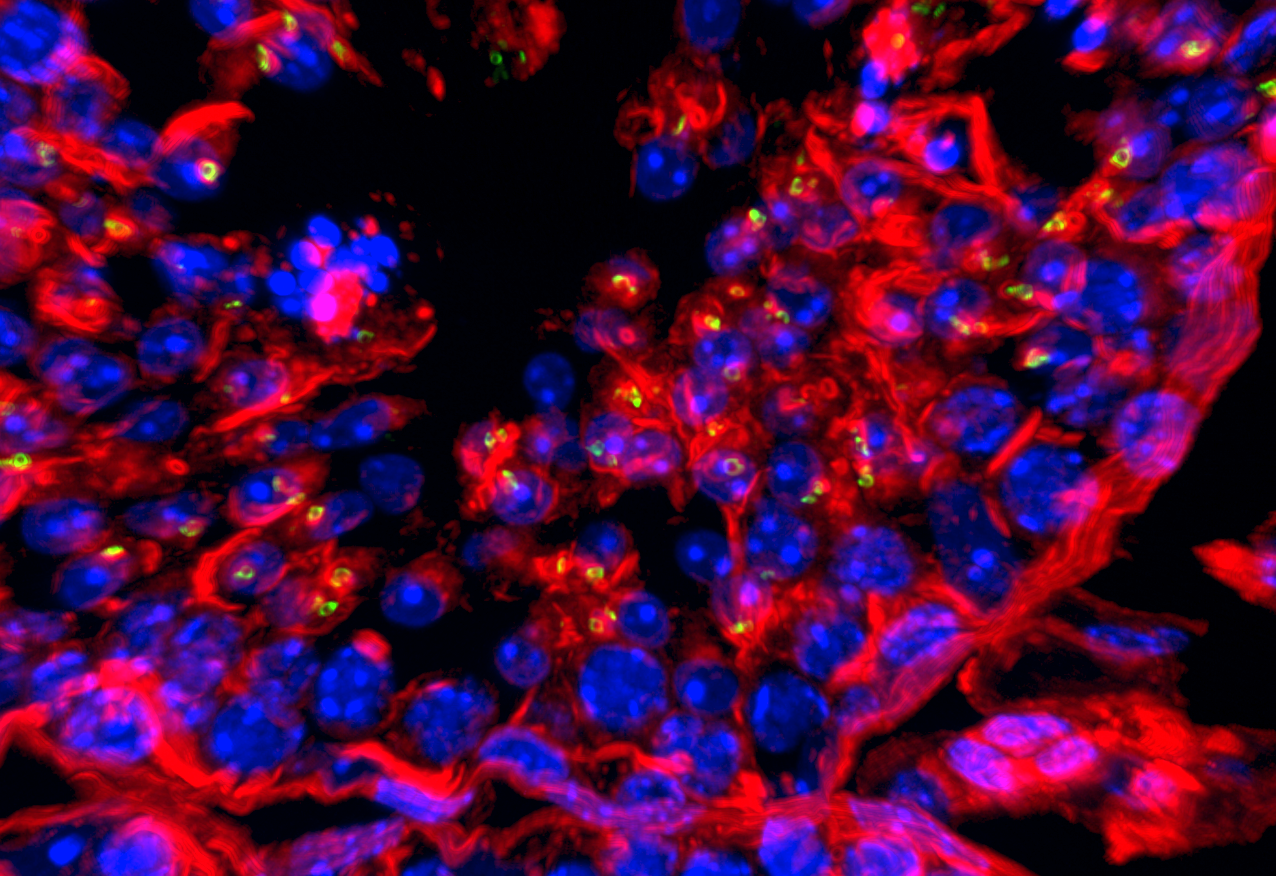

Supplement: Figure 5—source data 1. [file elife-83129-fig5-data1.zip › Figure5/Source data of Figure5A/F-actin+Lectin/HS-PD65-KO7.tif]

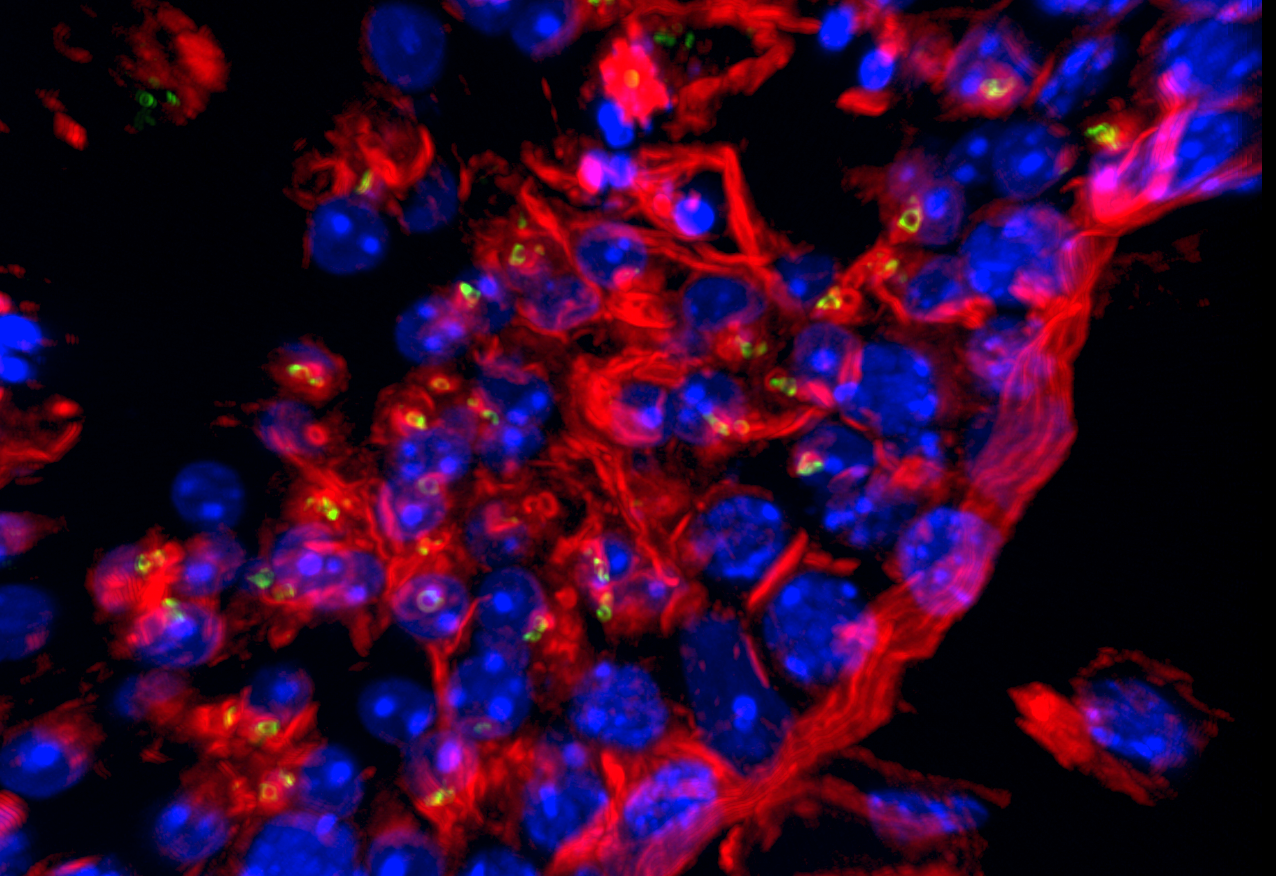

Supplement: Figure 5—source data 1. [file elife-83129-fig5-data1.zip › Figure5/Source data of Figure5A/F-actin+Lectin/HS-PD65-KO8.tif]

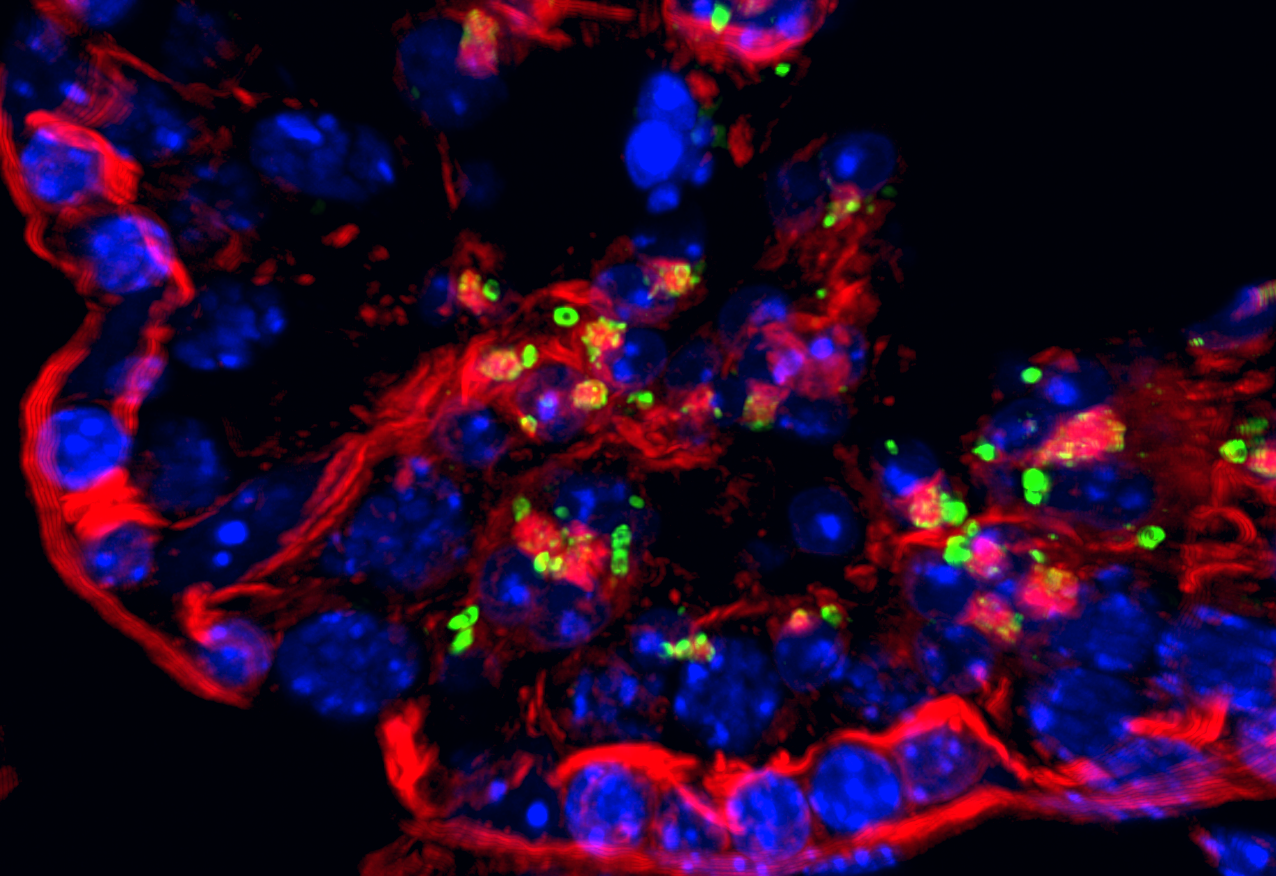

Supplement: Figure 5—source data 1. [file elife-83129-fig5-data1.zip › Figure5/Source data of Figure5A/F-actin+Lectin/HS-PD65-KO9.tif]

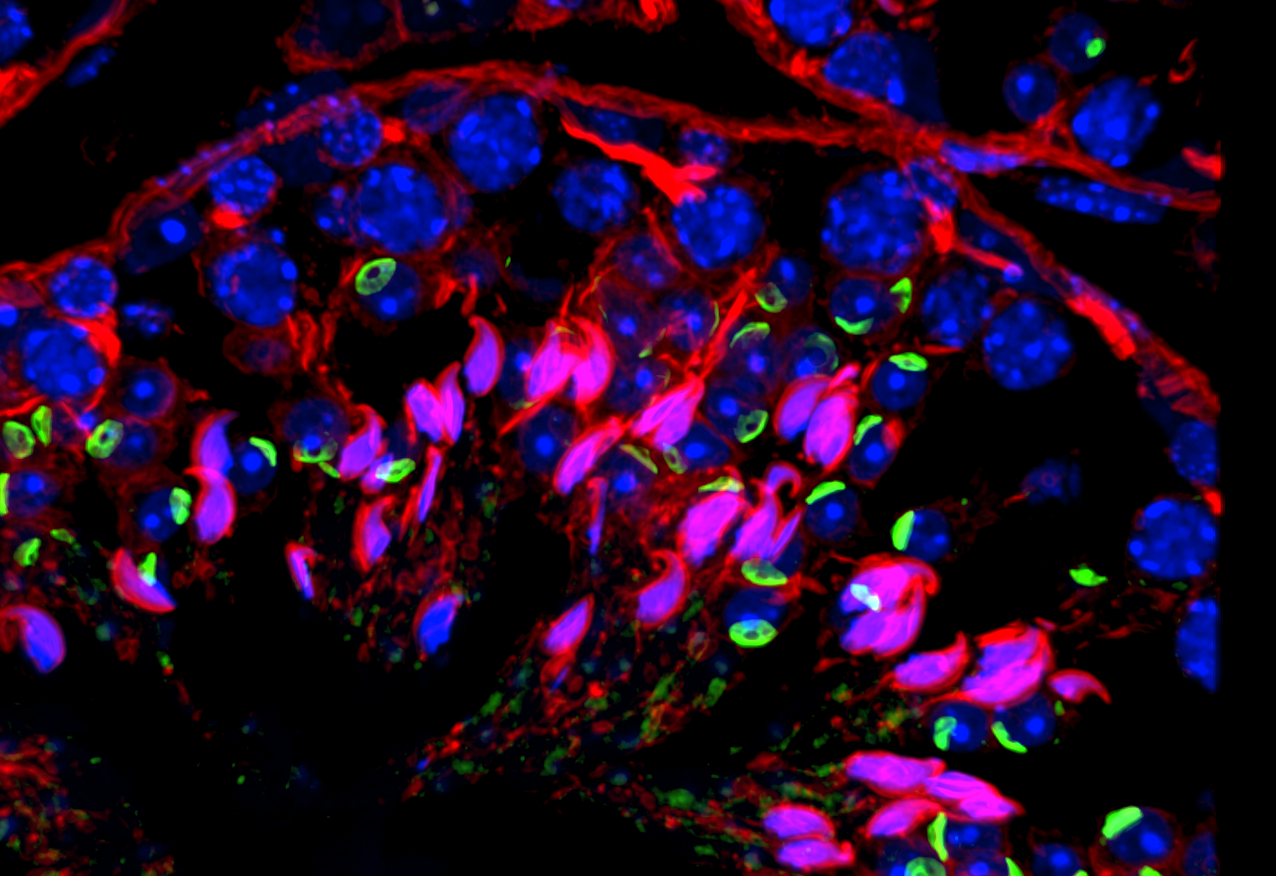

Supplement: Figure 5—source data 1. [file elife-83129-fig5-data1.zip › Figure5/Source data of Figure5A/F-actin+Lectin/HS-PD65-WT.tif]

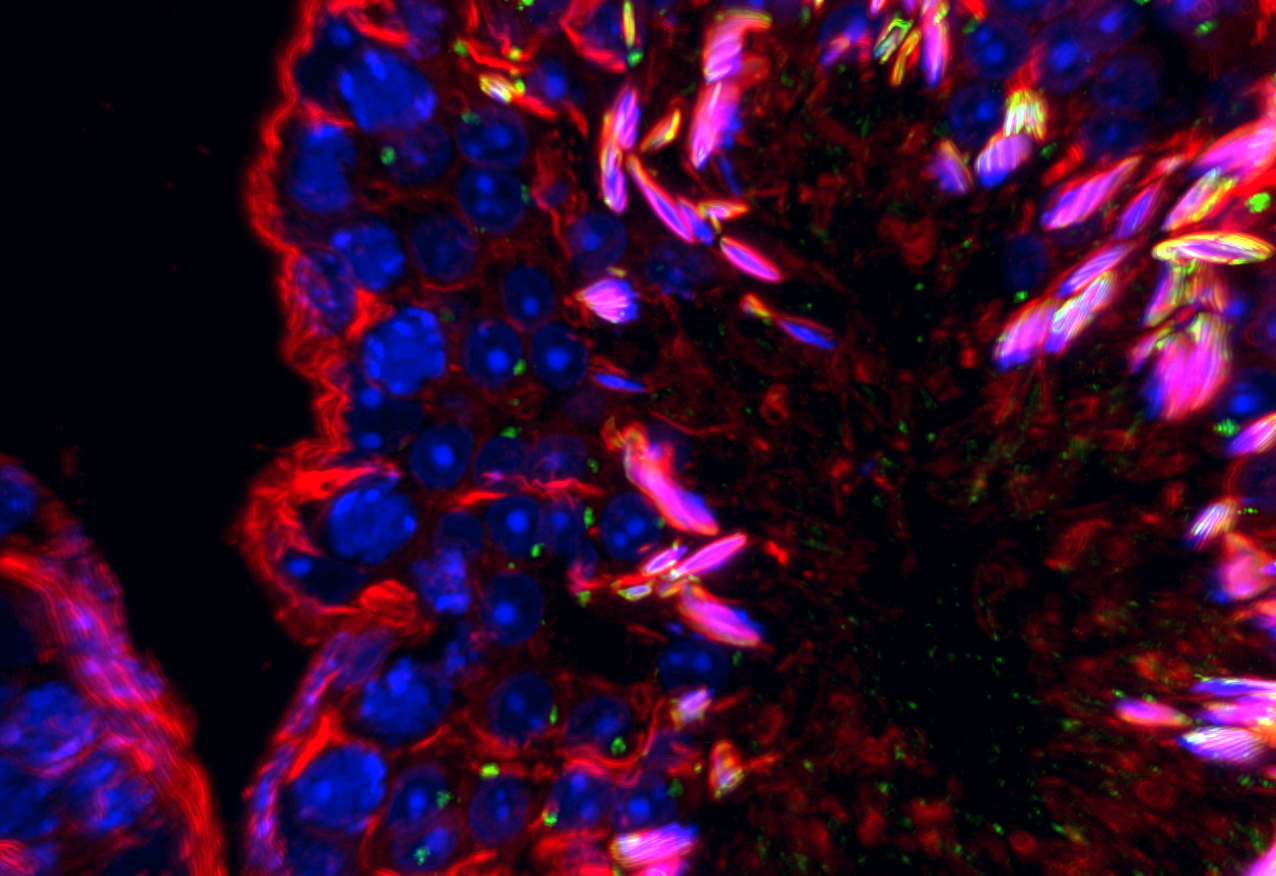

Supplement: Figure 5—source data 1. [file elife-83129-fig5-data1.zip › Figure5/Source data of Figure5A/F-actin+Lectin/HS-PD65-WT10.tif]

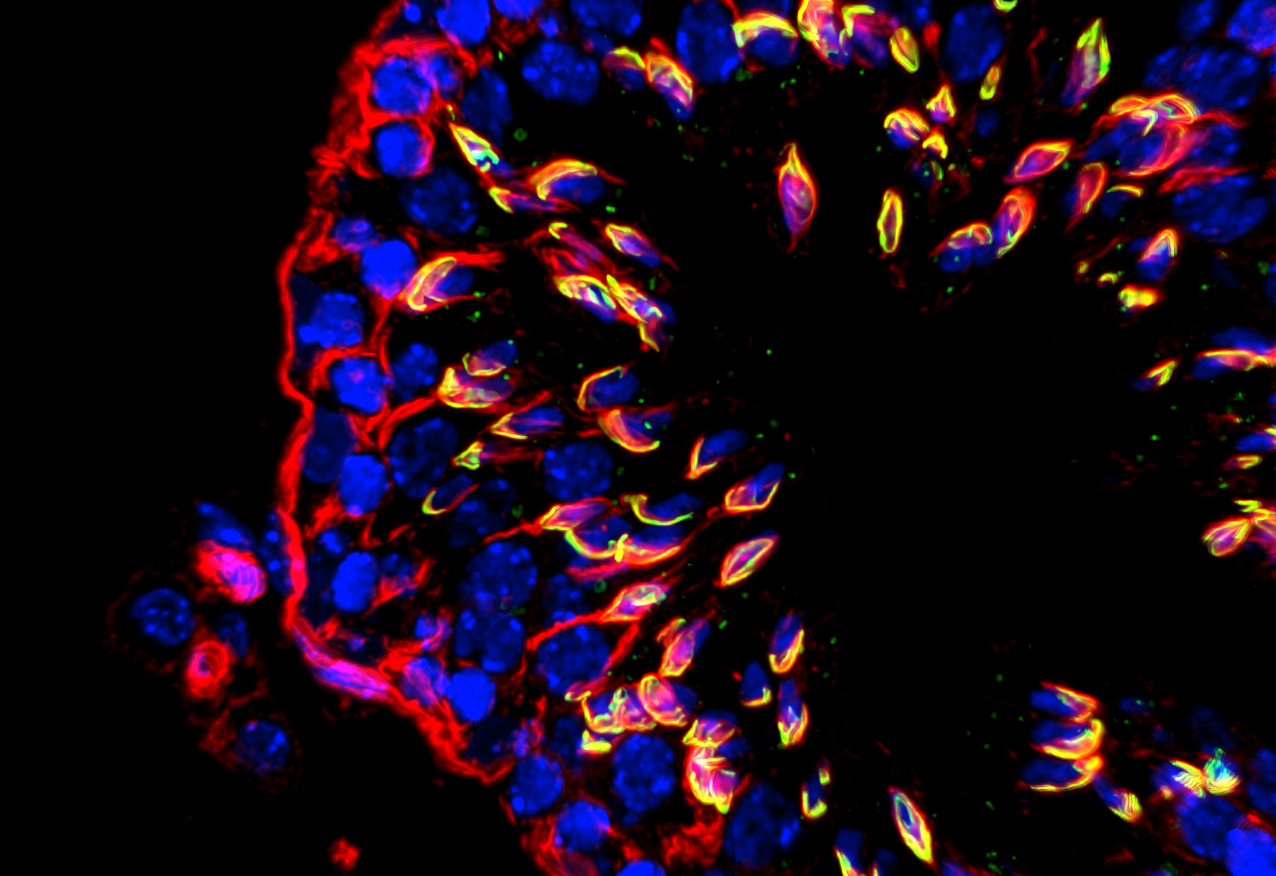

Supplement: Figure 5—source data 1. [file elife-83129-fig5-data1.zip › Figure5/Source data of Figure5A/F-actin+Lectin/HS-PD65-WT11.tif]

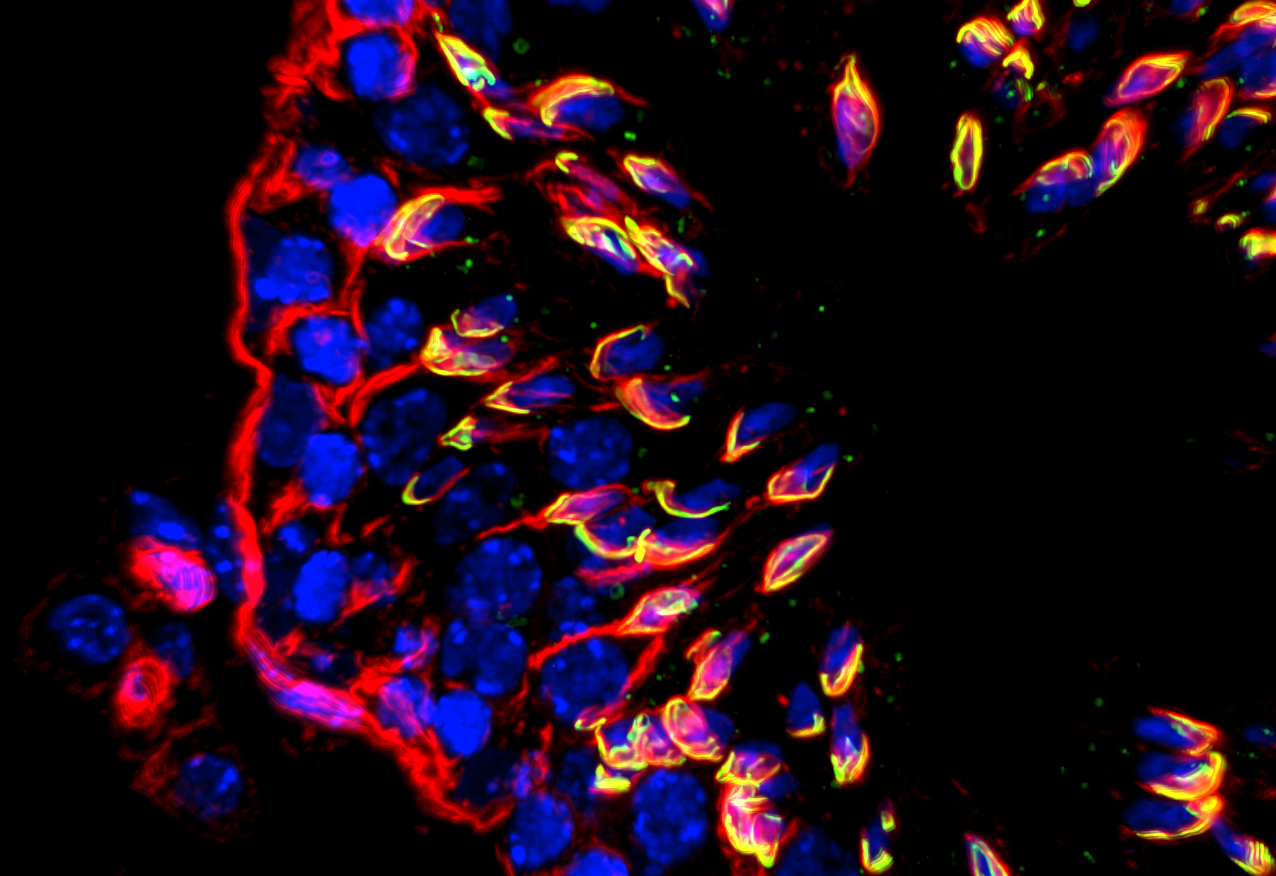

Supplement: Figure 5—source data 1. [file elife-83129-fig5-data1.zip › Figure5/Source data of Figure5A/F-actin+Lectin/HS-PD65-WT12.tif]

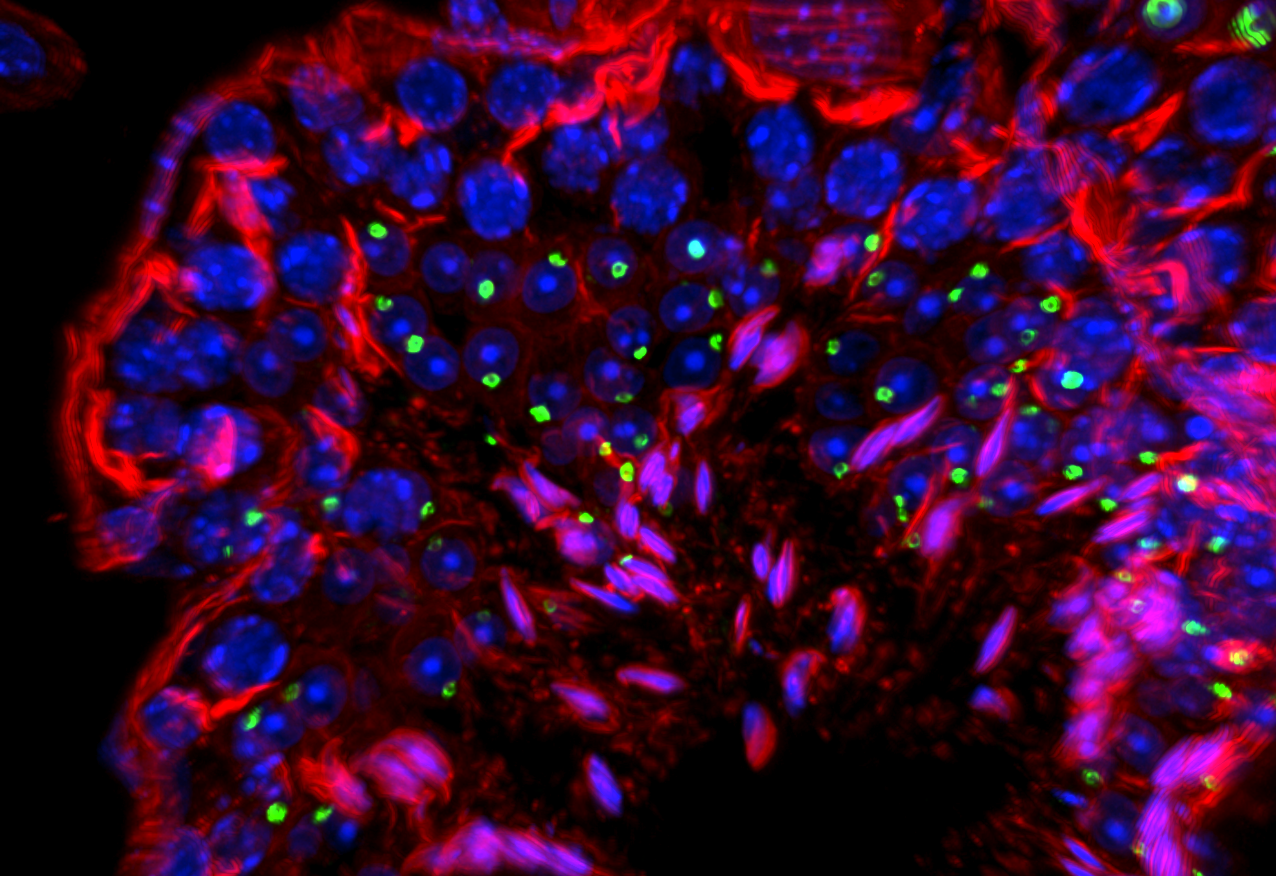

Supplement: Figure 5—source data 1. [file elife-83129-fig5-data1.zip › Figure5/Source data of Figure5A/F-actin+Lectin/HS-PD65-WT13.tif]

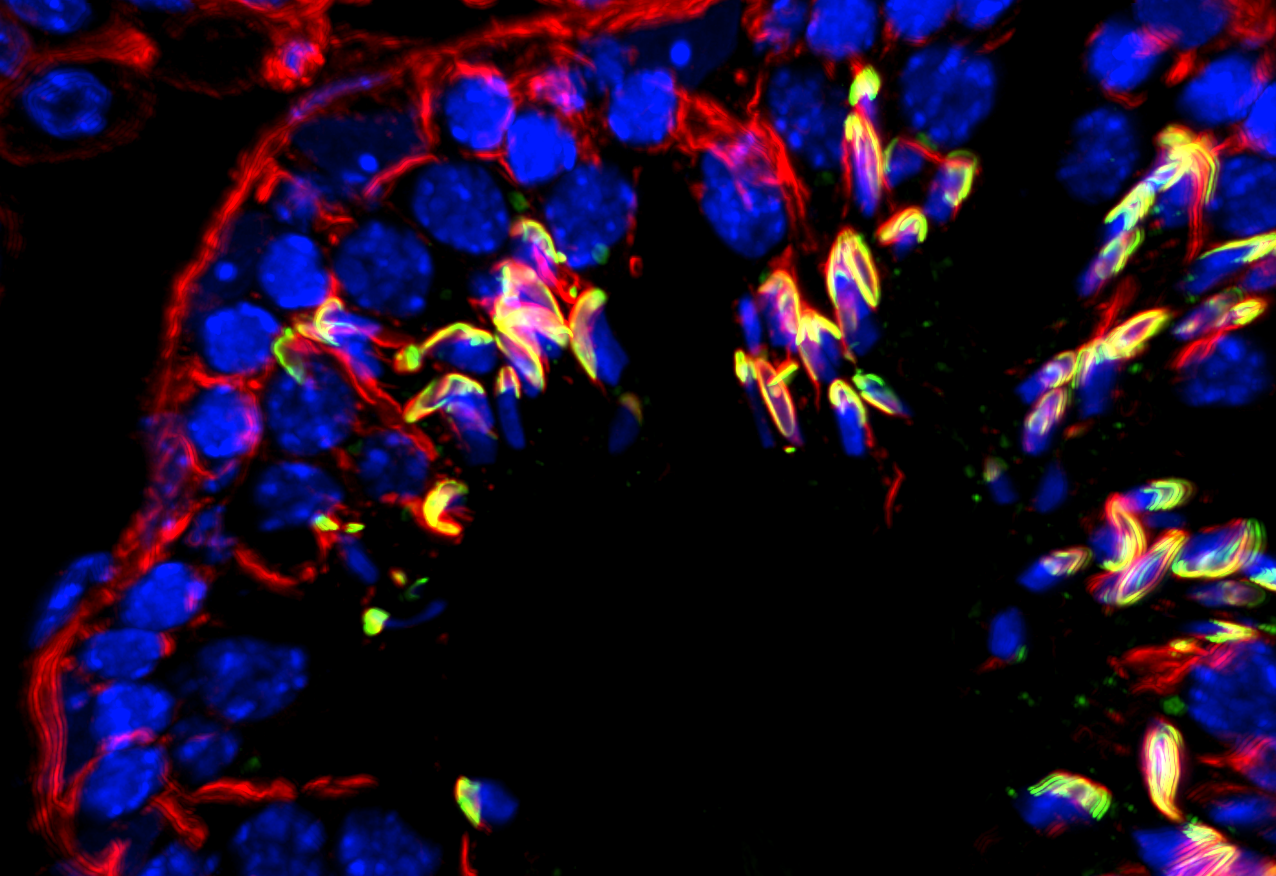

Supplement: Figure 5—source data 1. [file elife-83129-fig5-data1.zip › Figure5/Source data of Figure5A/F-actin+Lectin/HS-PD65-WT14.tif]

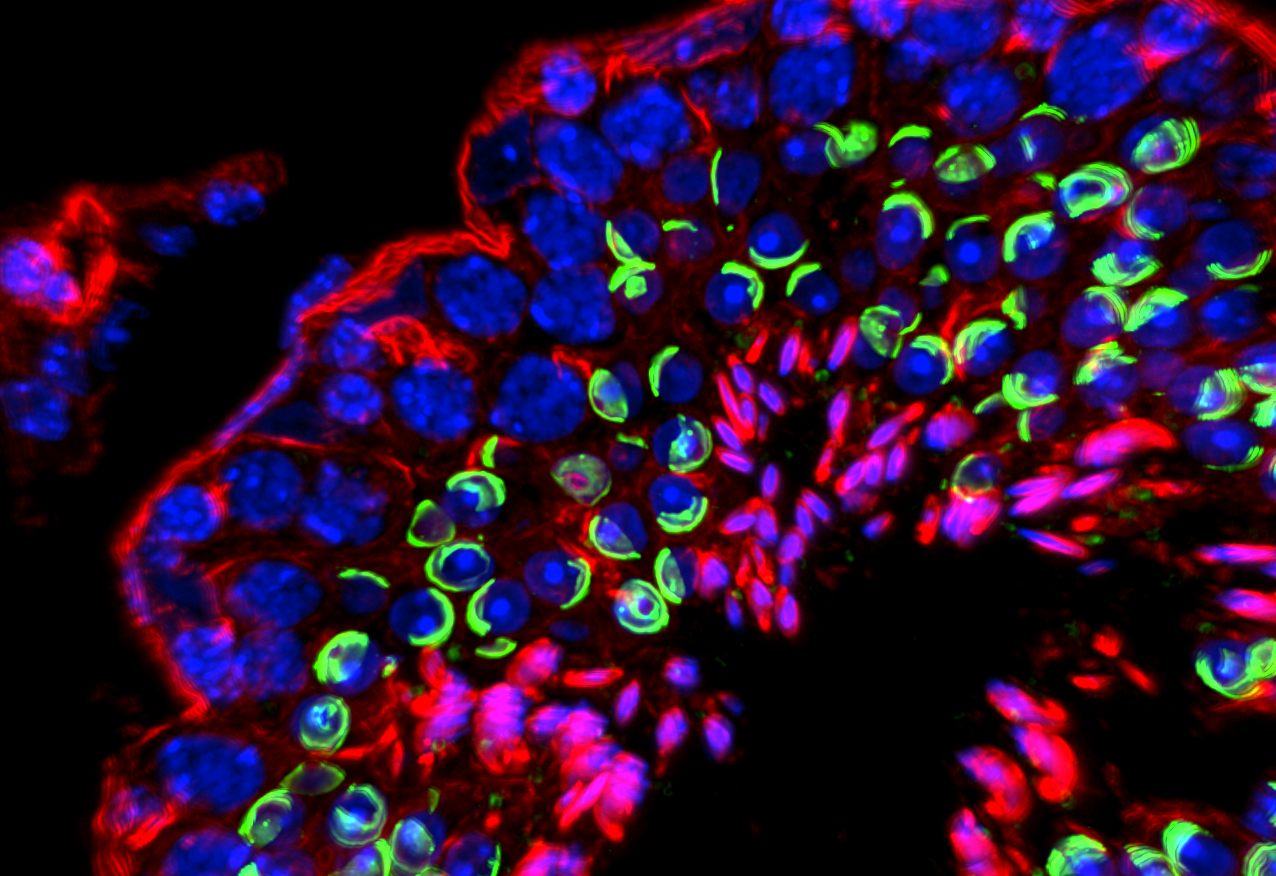

Supplement: Figure 5—source data 1. [file elife-83129-fig5-data1.zip › Figure5/Source data of Figure5A/F-actin+Lectin/HS-PD65-WT15.tif]

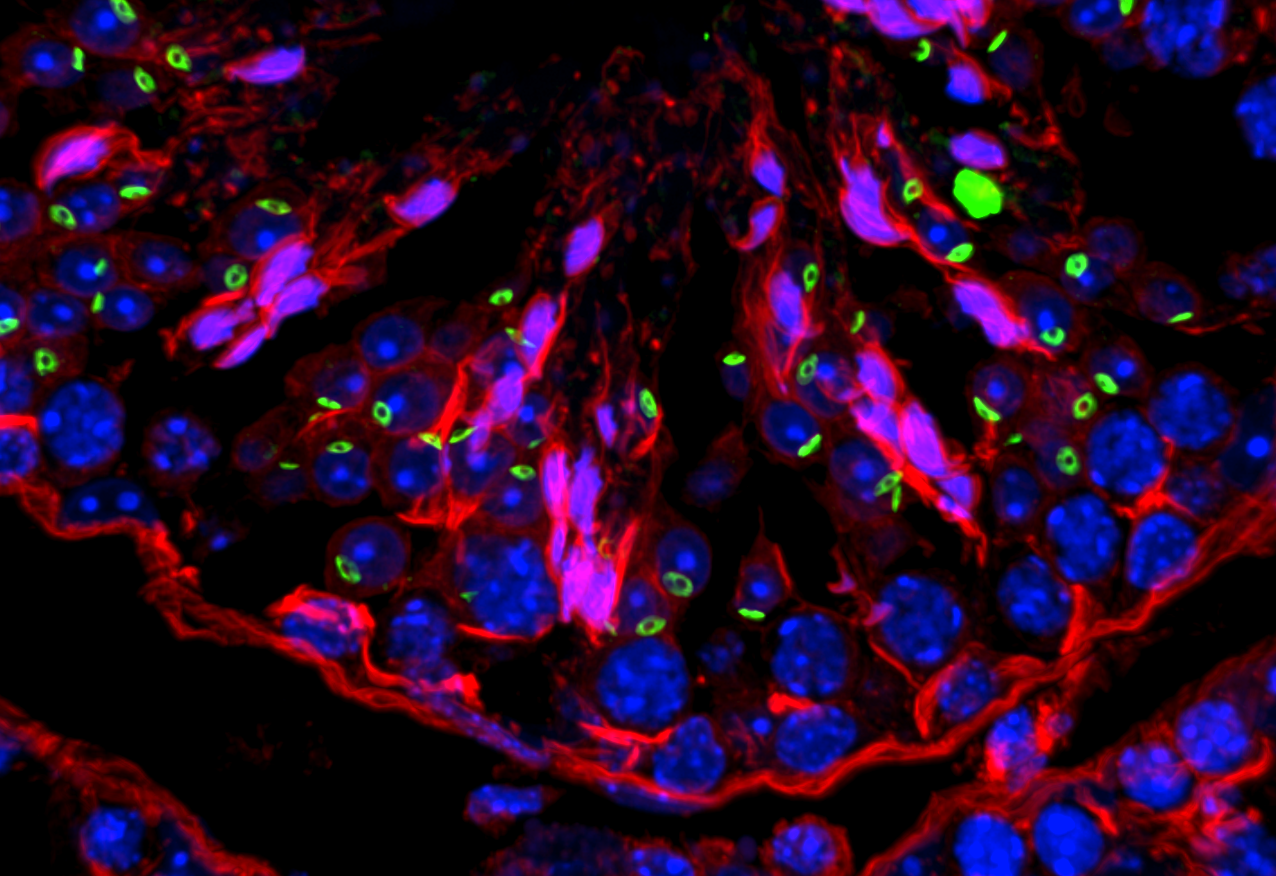

Supplement: Figure 5—source data 1. [file elife-83129-fig5-data1.zip › Figure5/Source data of Figure5A/F-actin+Lectin/HS-PD65-WT2.tif]

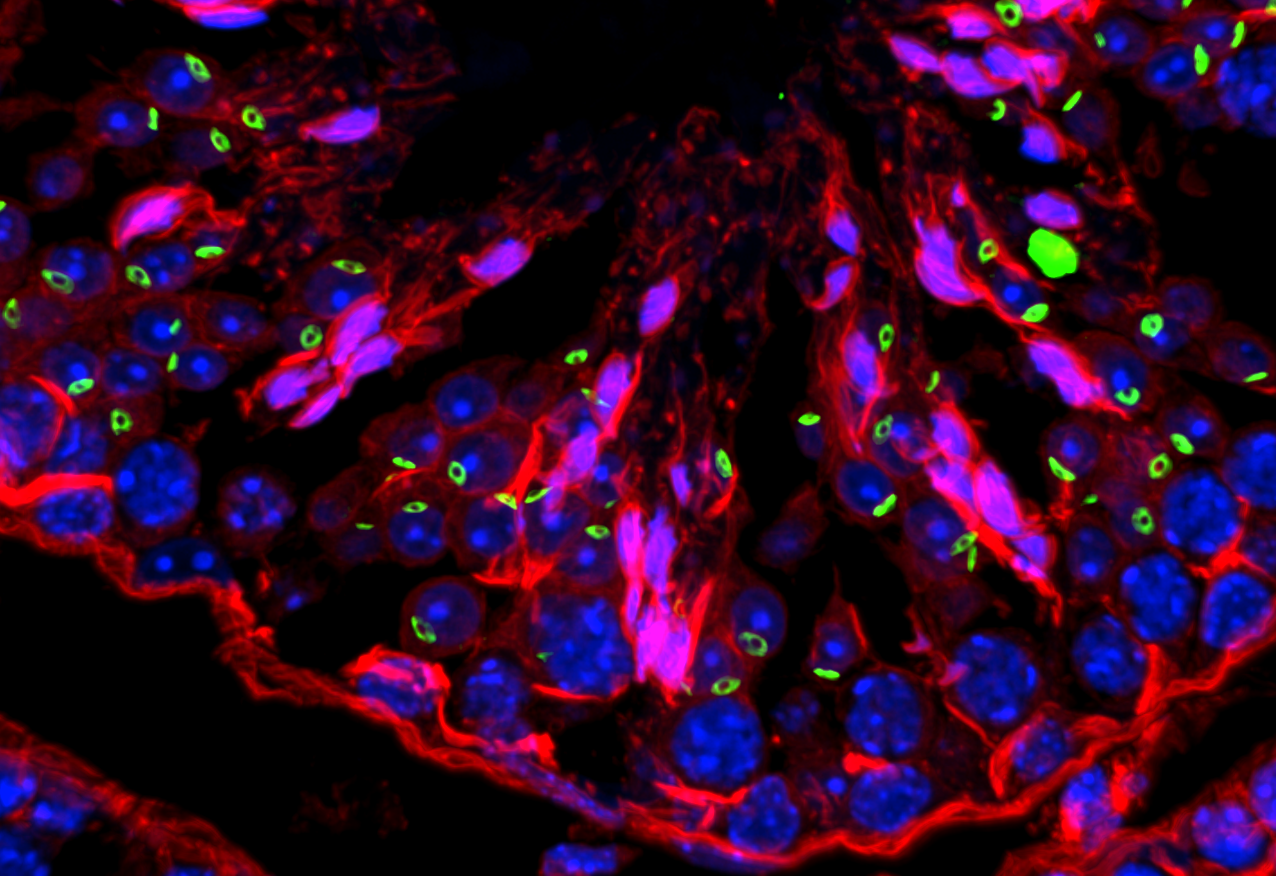

Supplement: Figure 5—source data 1. [file elife-83129-fig5-data1.zip › Figure5/Source data of Figure5A/F-actin+Lectin/HS-PD65-WT3.tif]

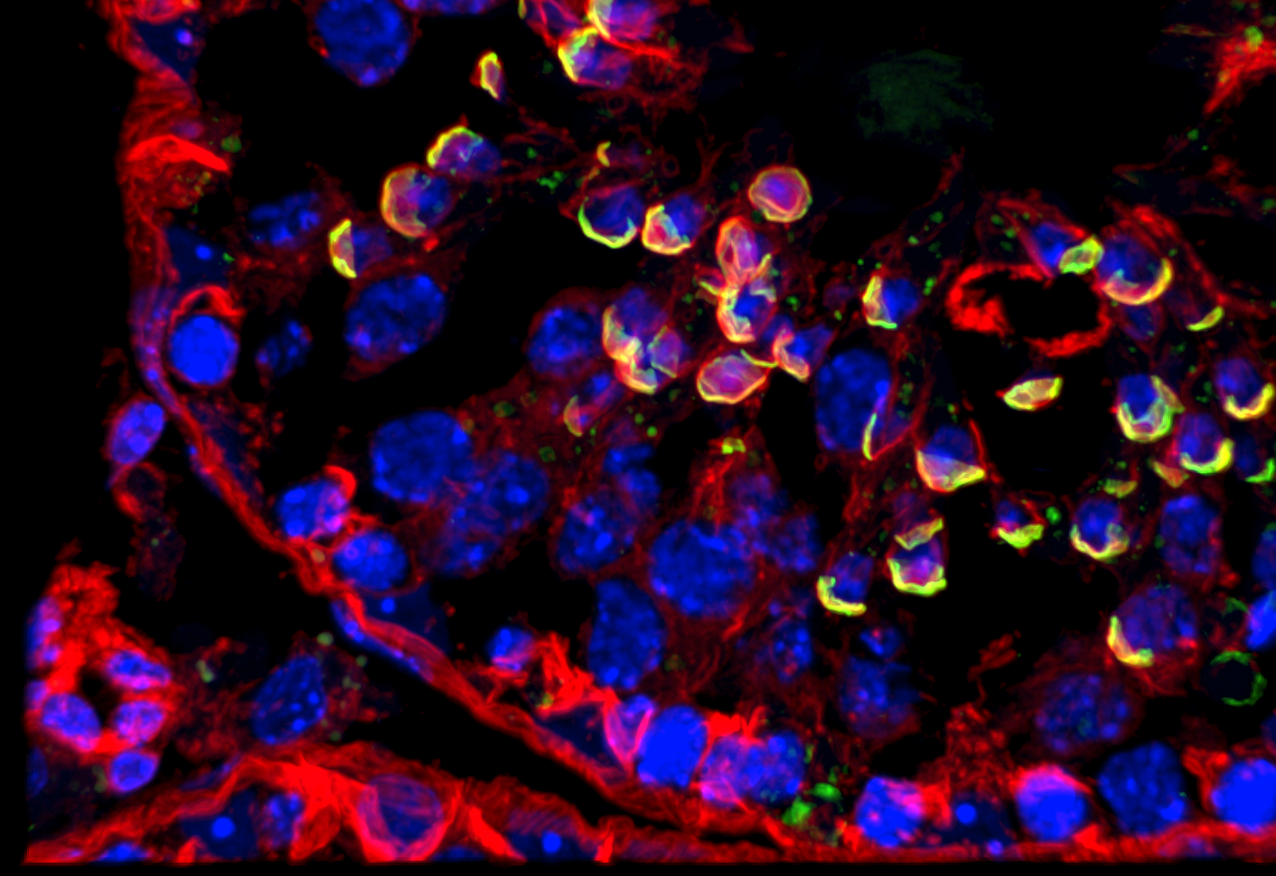

Supplement: Figure 5—source data 1. [file elife-83129-fig5-data1.zip › Figure5/Source data of Figure5A/F-actin+Lectin/HS-PD65-WT4.tif]

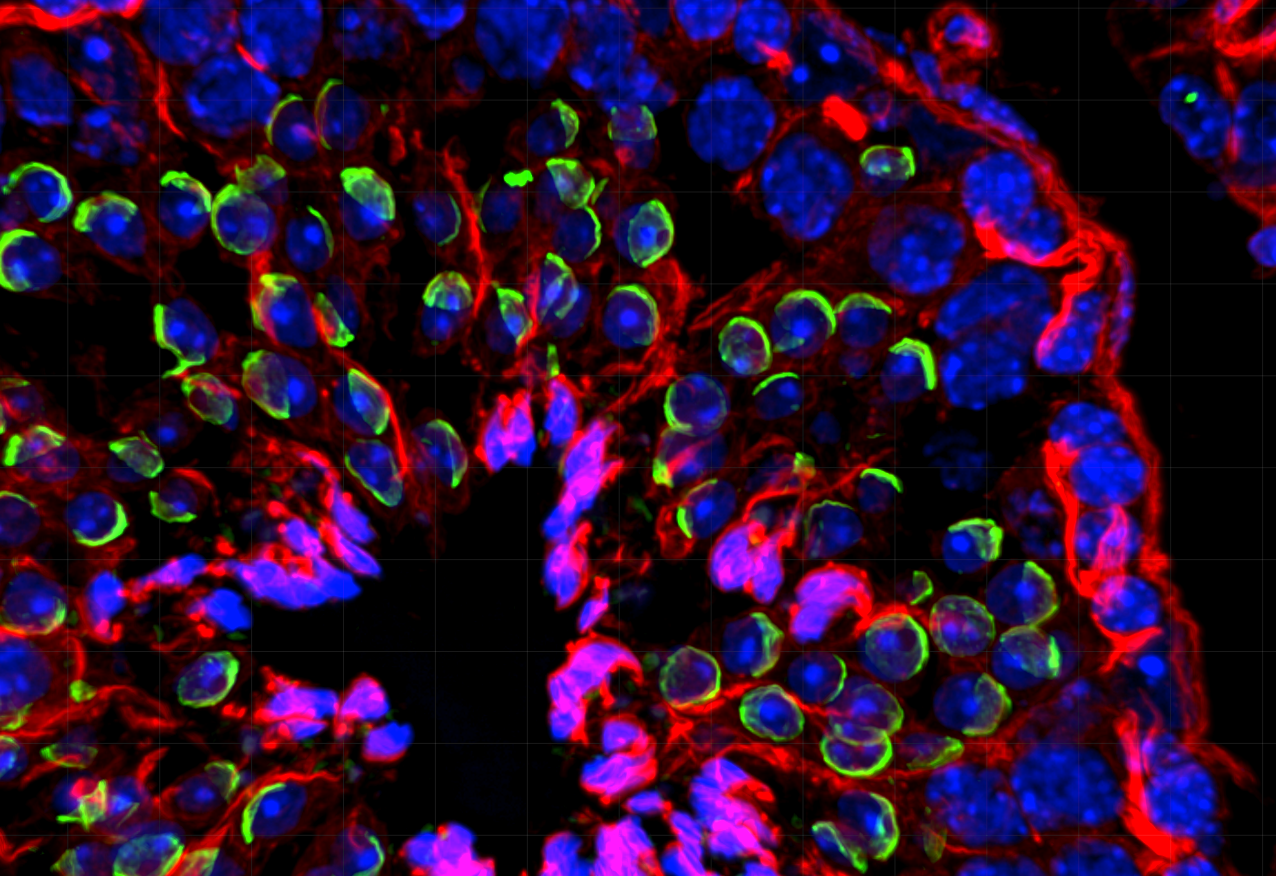

Supplement: Figure 5—source data 1. [file elife-83129-fig5-data1.zip › Figure5/Source data of Figure5A/F-actin+Lectin/HS-PD65-WT5.tif]

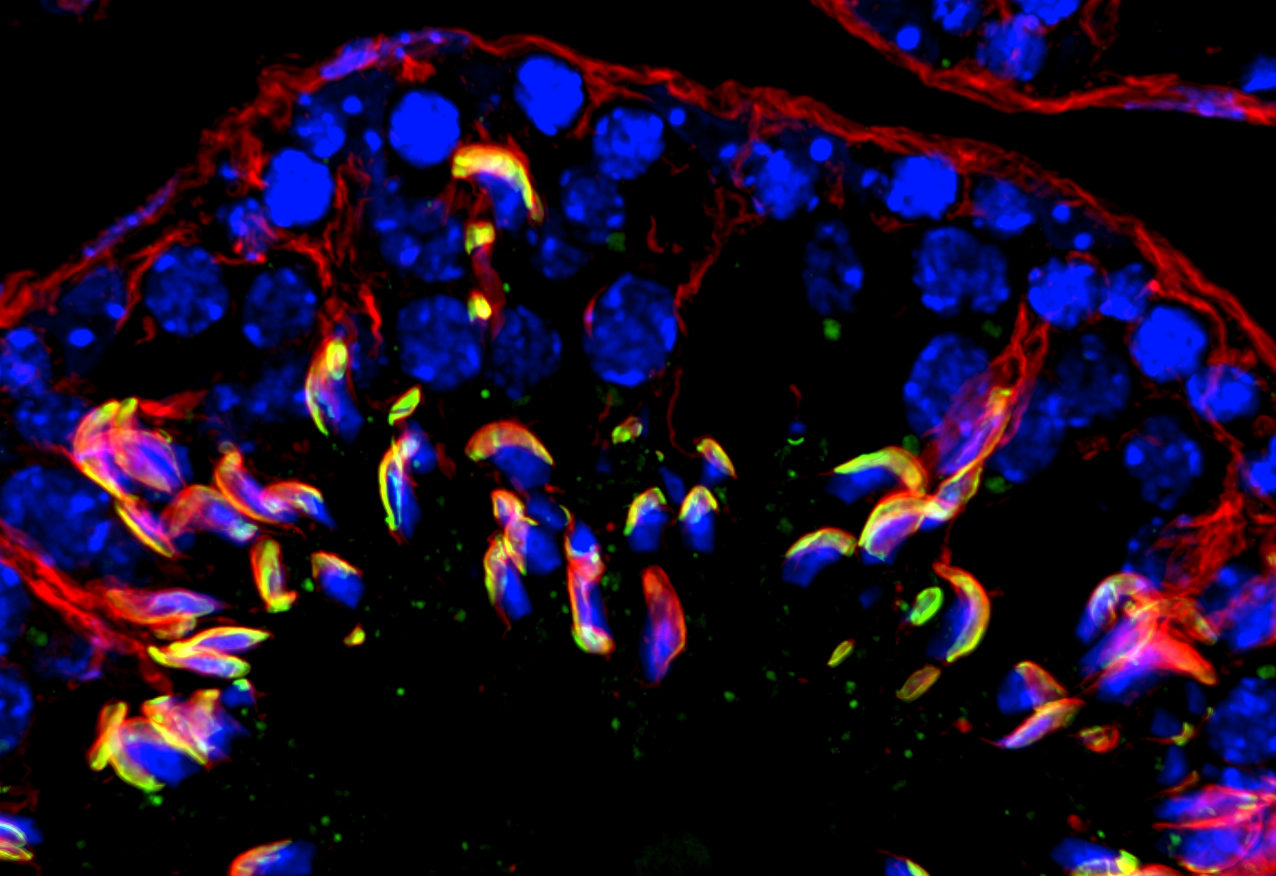

Supplement: Figure 5—source data 1. [file elife-83129-fig5-data1.zip › Figure5/Source data of Figure5A/F-actin+Lectin/HS-PD65-WT6.tif]

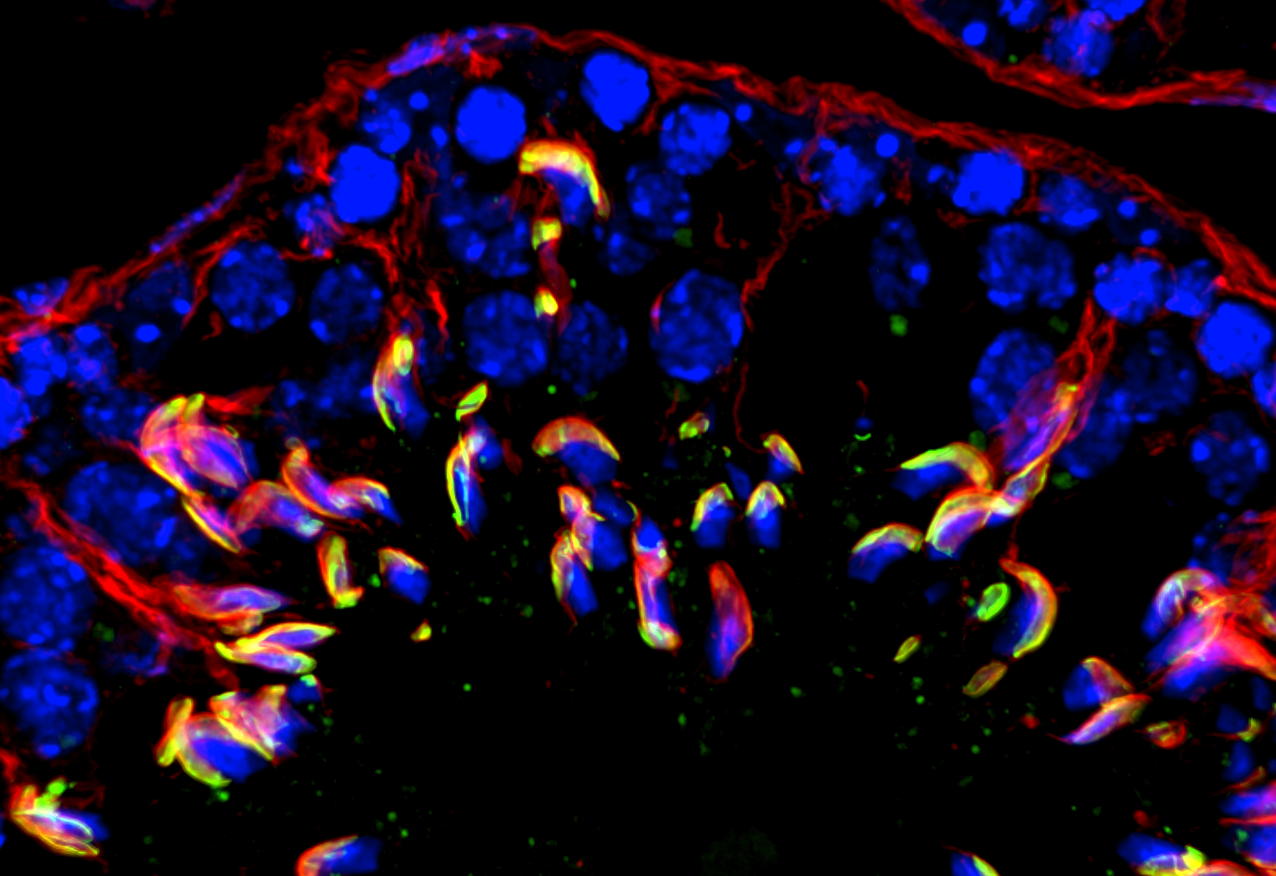

Supplement: Figure 5—source data 1. [file elife-83129-fig5-data1.zip › Figure5/Source data of Figure5A/F-actin+Lectin/HS-PD65-WT7.tif]

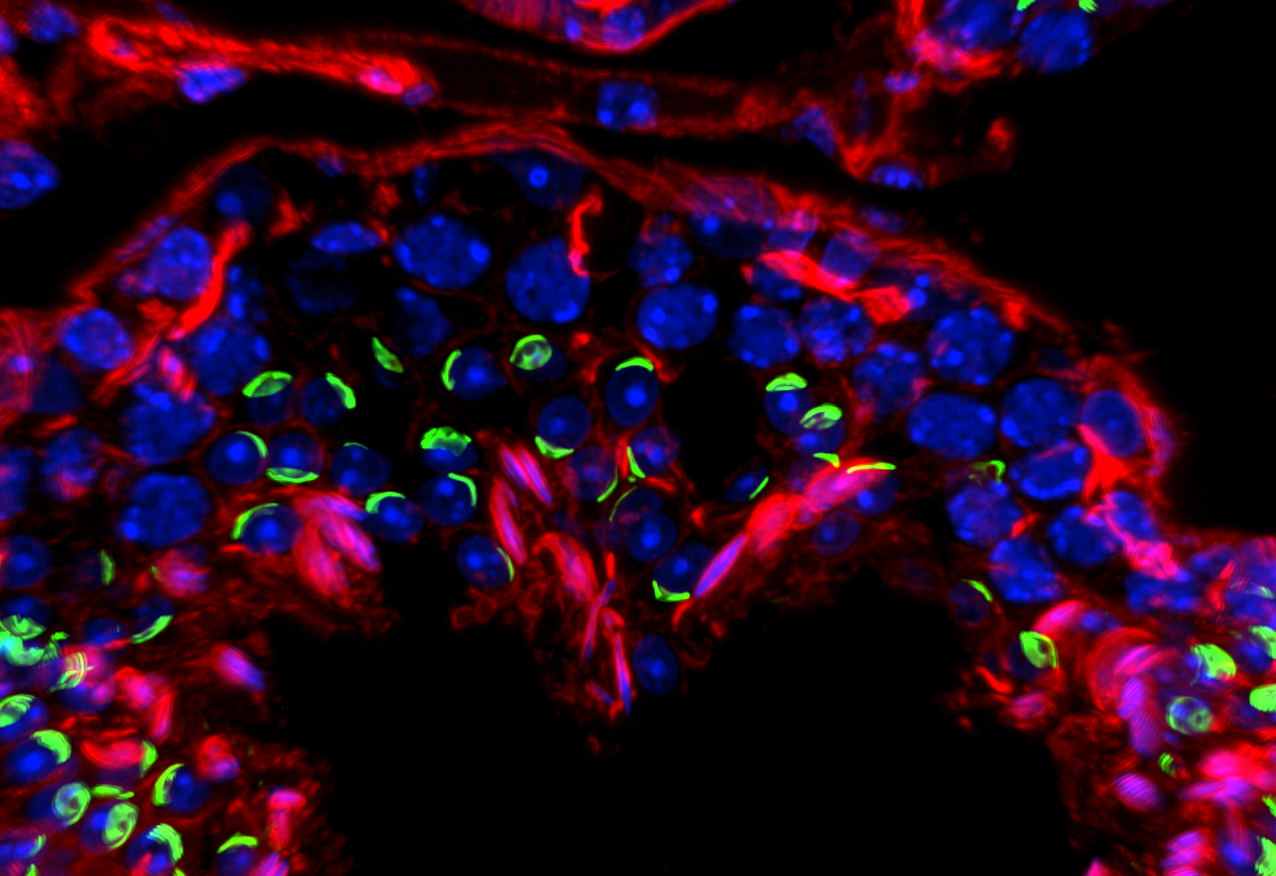

Supplement: Figure 5—source data 1. [file elife-83129-fig5-data1.zip › Figure5/Source data of Figure5A/F-actin+Lectin/HS-PD65-WT8.tif]

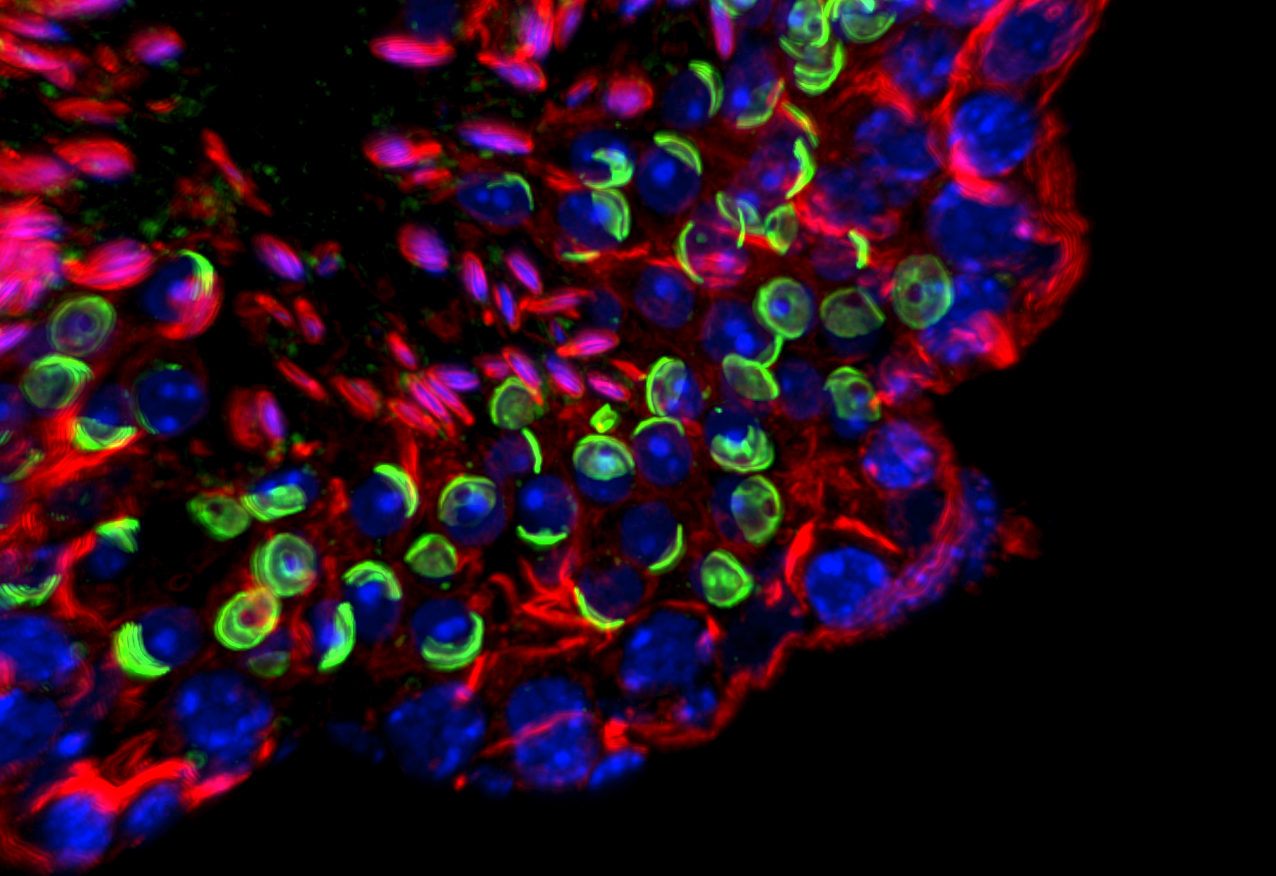

Supplement: Figure 5—source data 1. [file elife-83129-fig5-data1.zip › Figure5/Source data of Figure5A/F-actin+Lectin/HS-PD65-WT9.tif]

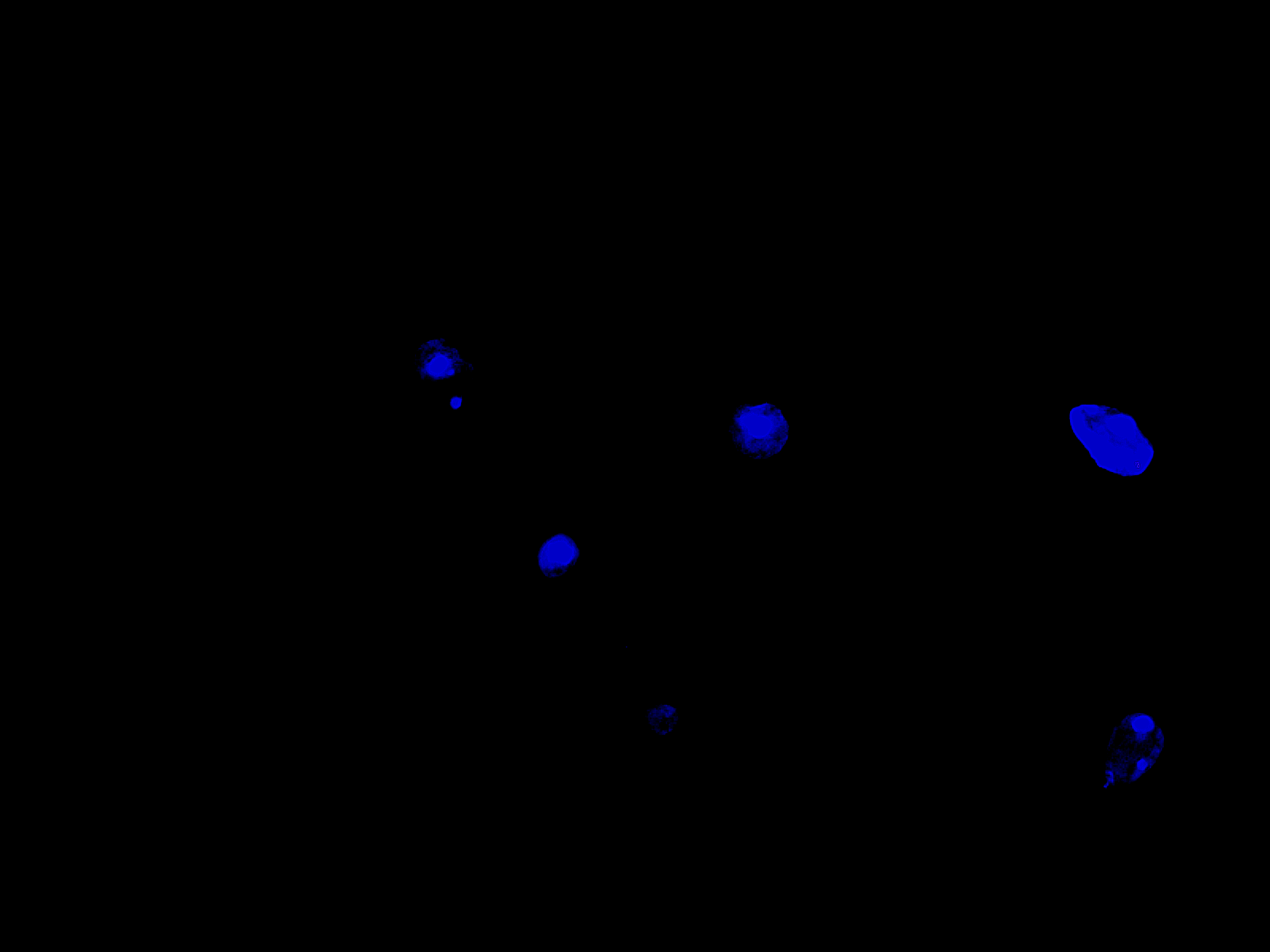

Supplement: Figure 5—source data 1. [file elife-83129-fig5-data1.zip › Figure5/Source data of Figure5B/KO/1/DAPI.tif]

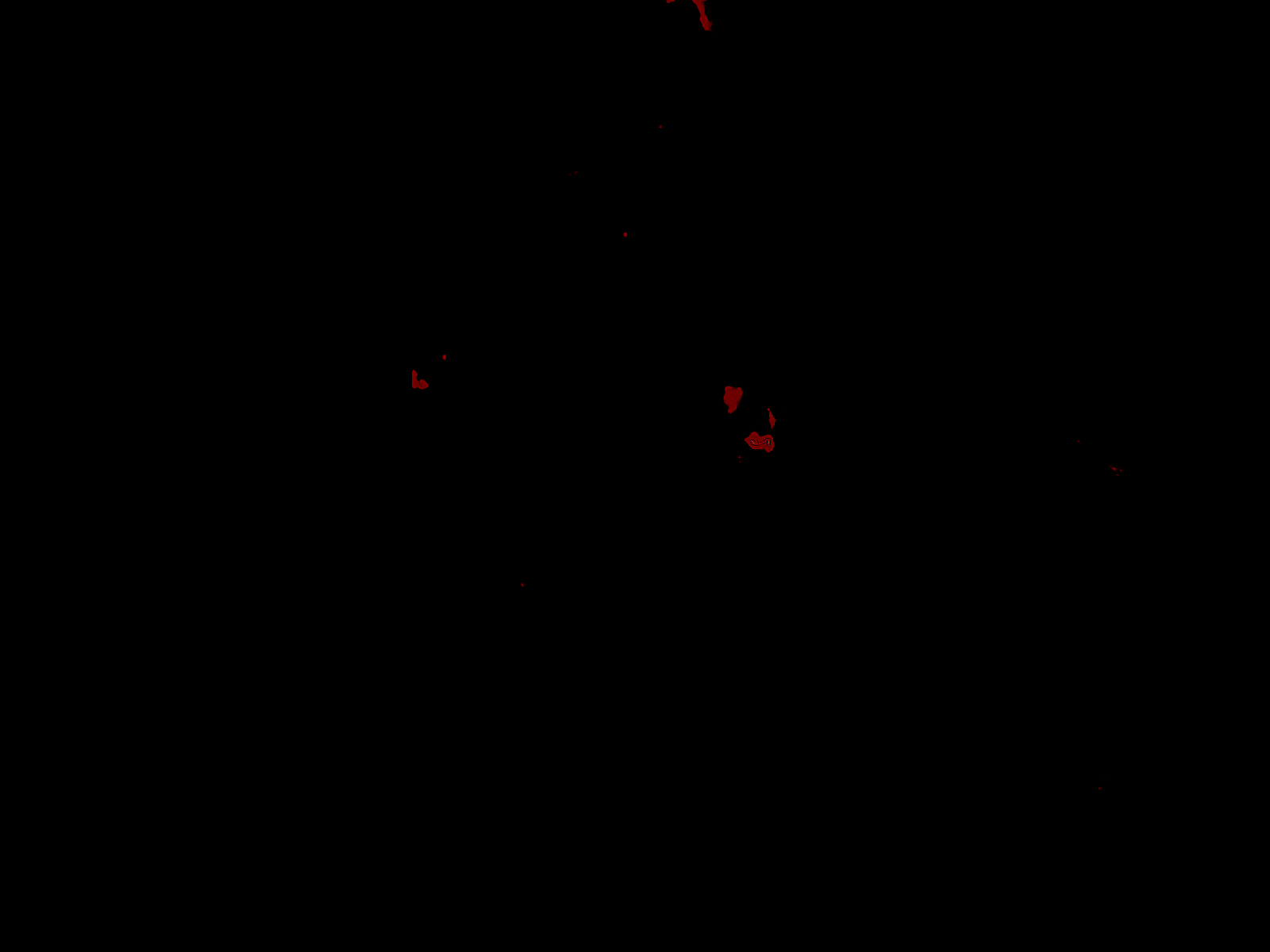

Supplement: Figure 5—source data 1. [file elife-83129-fig5-data1.zip › Figure5/Source data of Figure5B/KO/1/F-actin.tif]

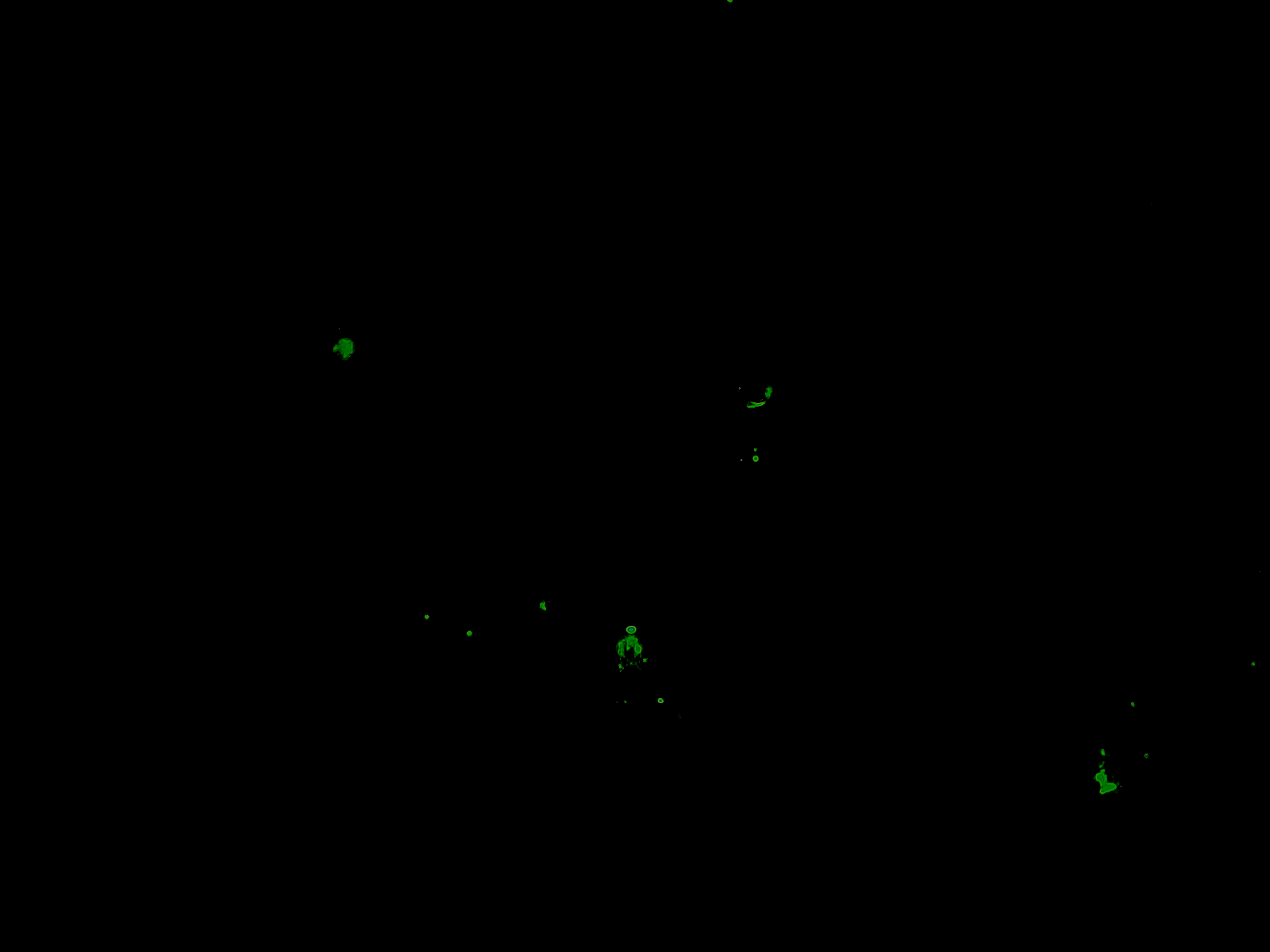

Supplement: Figure 5—source data 1. [file elife-83129-fig5-data1.zip › Figure5/Source data of Figure5B/KO/1/lectin.tif]

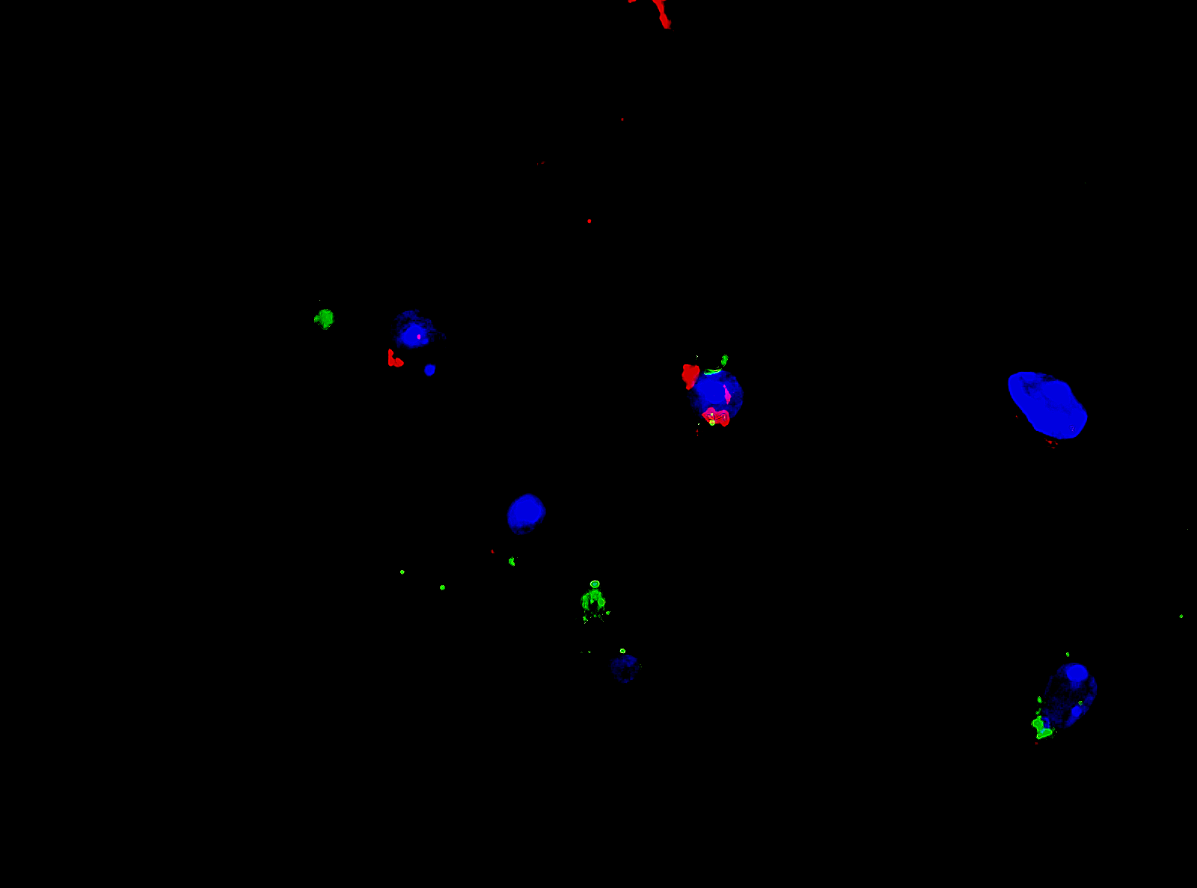

Supplement: Figure 5—source data 1. [file elife-83129-fig5-data1.zip › Figure5/Source data of Figure5B/KO/1/merge.tif]

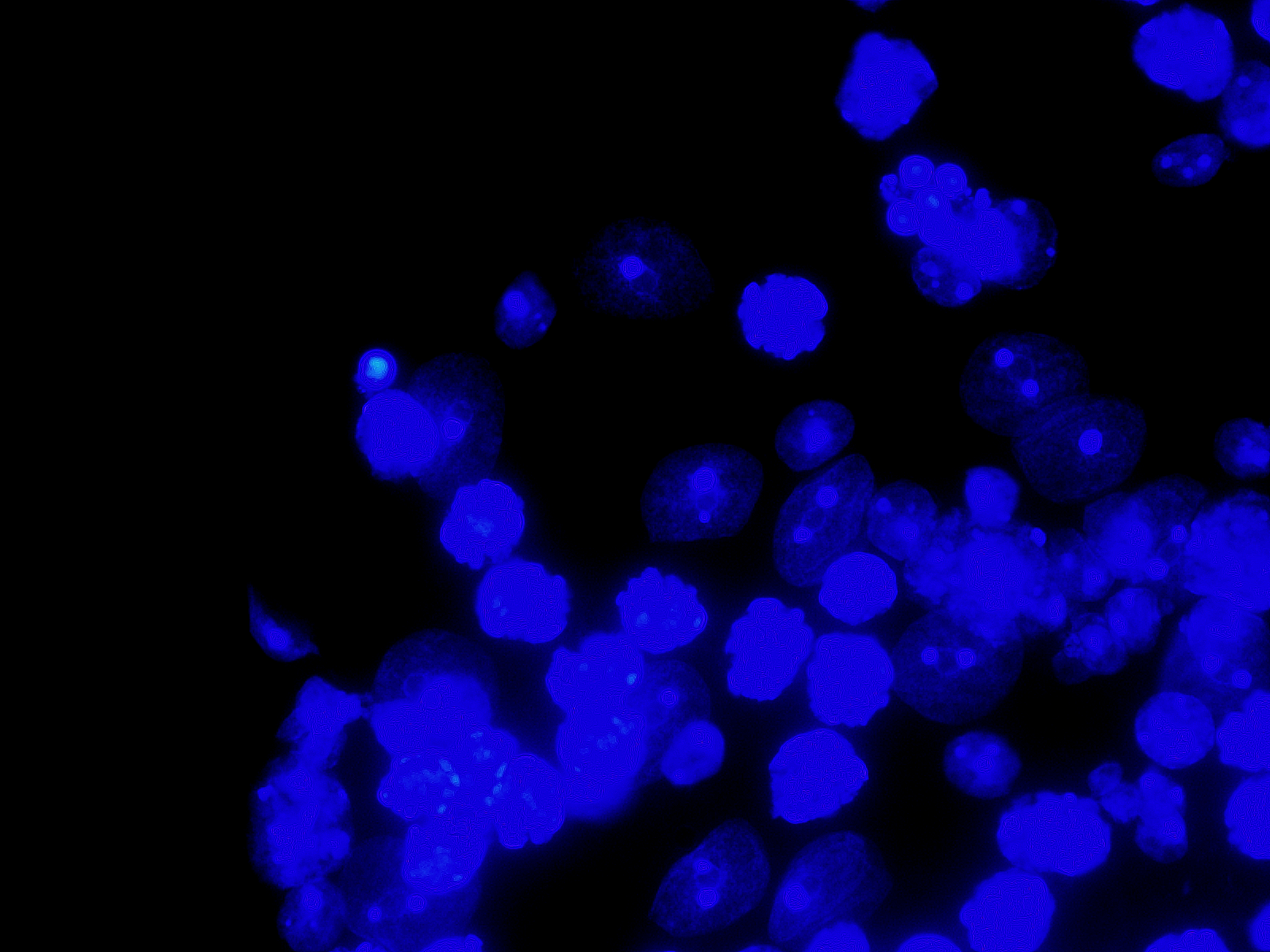

Supplement: Figure 5—source data 1. [file elife-83129-fig5-data1.zip › Figure5/Source data of Figure5B/KO/2/DAPI.tif]

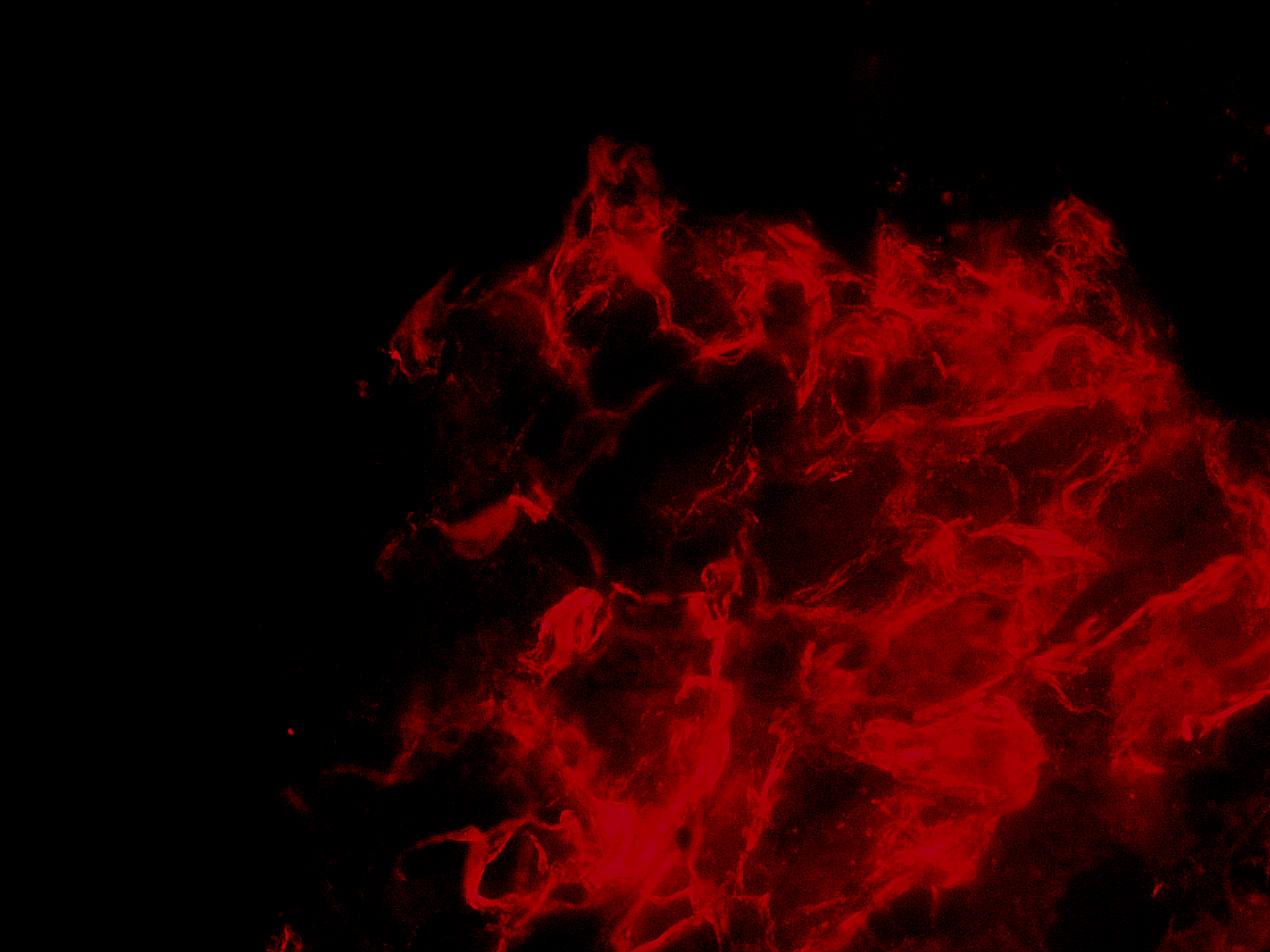

Supplement: Figure 5—source data 1. [file elife-83129-fig5-data1.zip › Figure5/Source data of Figure5B/KO/2/F-actin.tif]

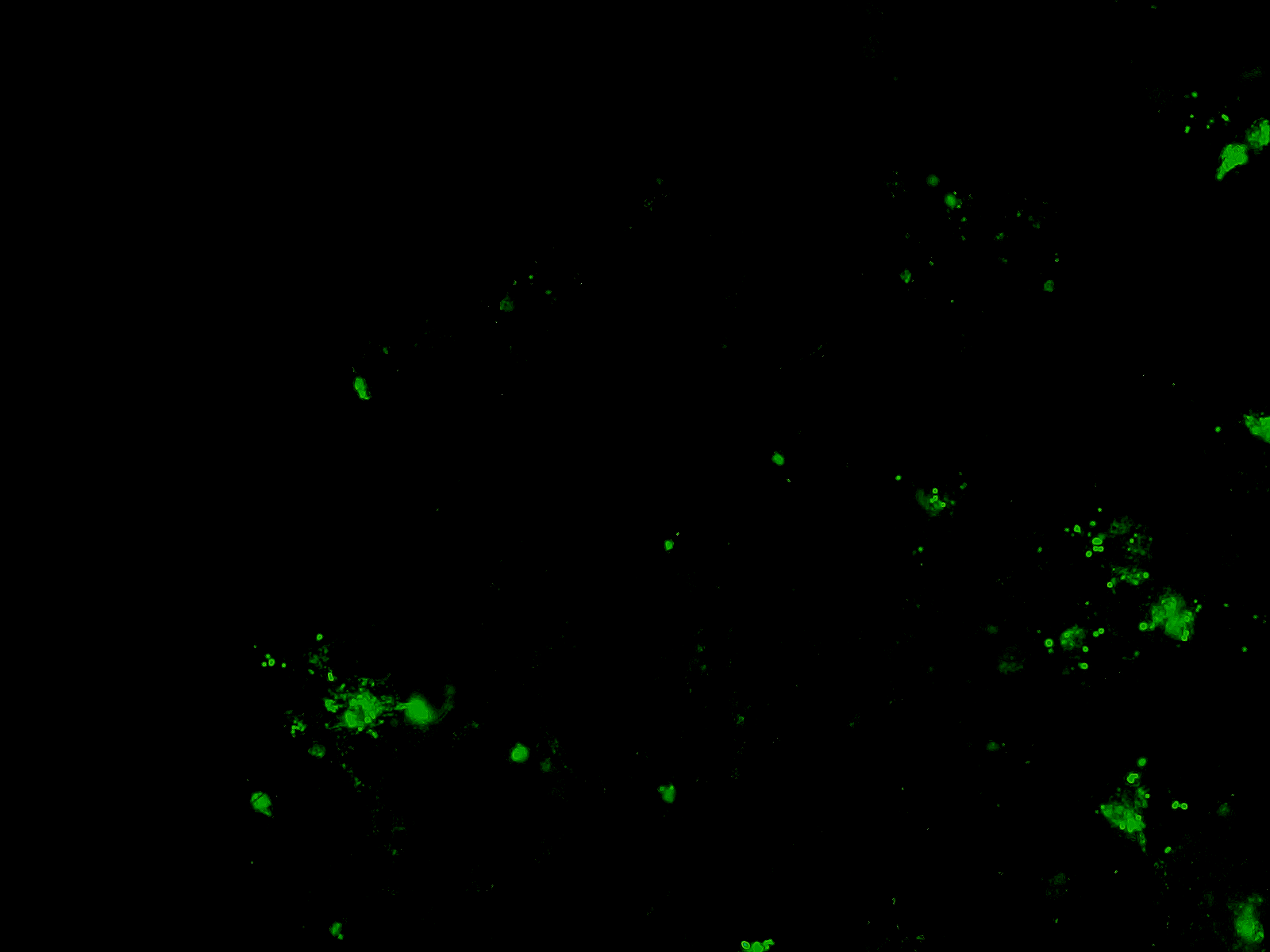

Supplement: Figure 5—source data 1. [file elife-83129-fig5-data1.zip › Figure5/Source data of Figure5B/KO/2/lectin.tif]

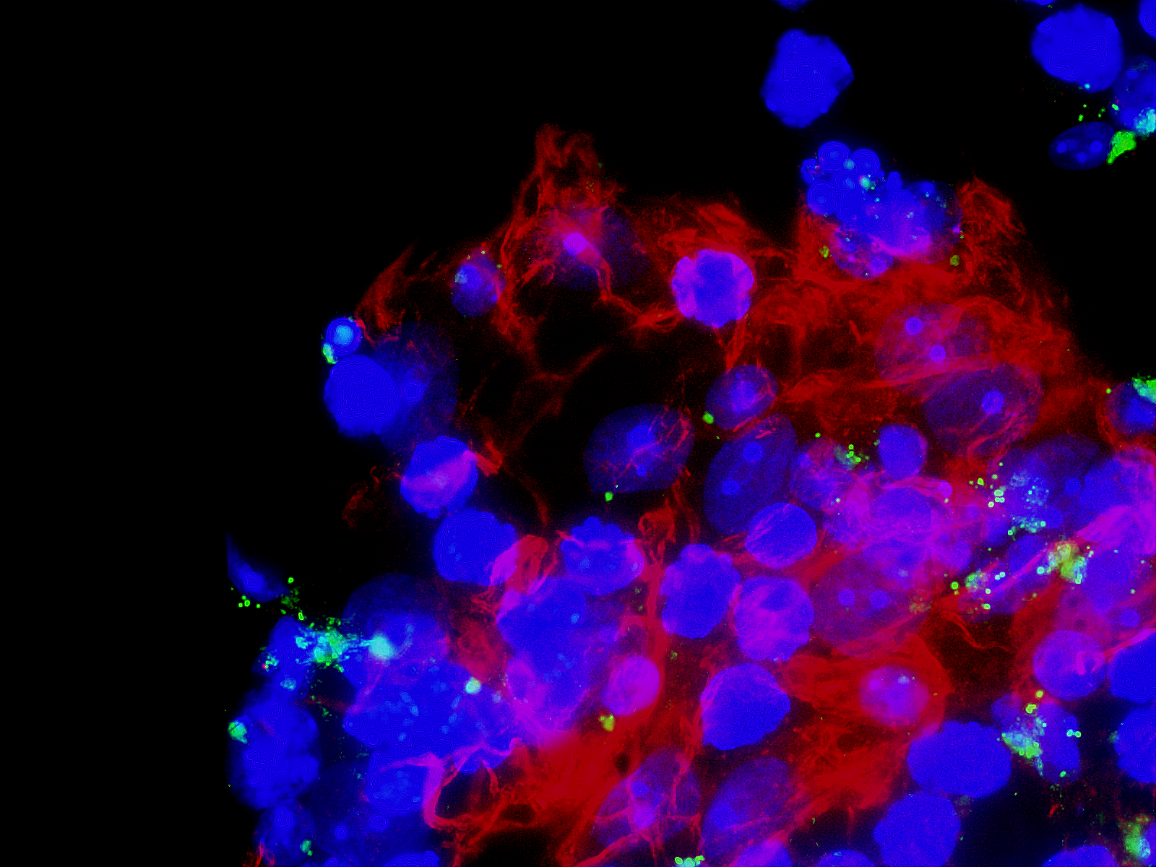

Supplement: Figure 5—source data 1. [file elife-83129-fig5-data1.zip › Figure5/Source data of Figure5B/KO/2/merge.tif]

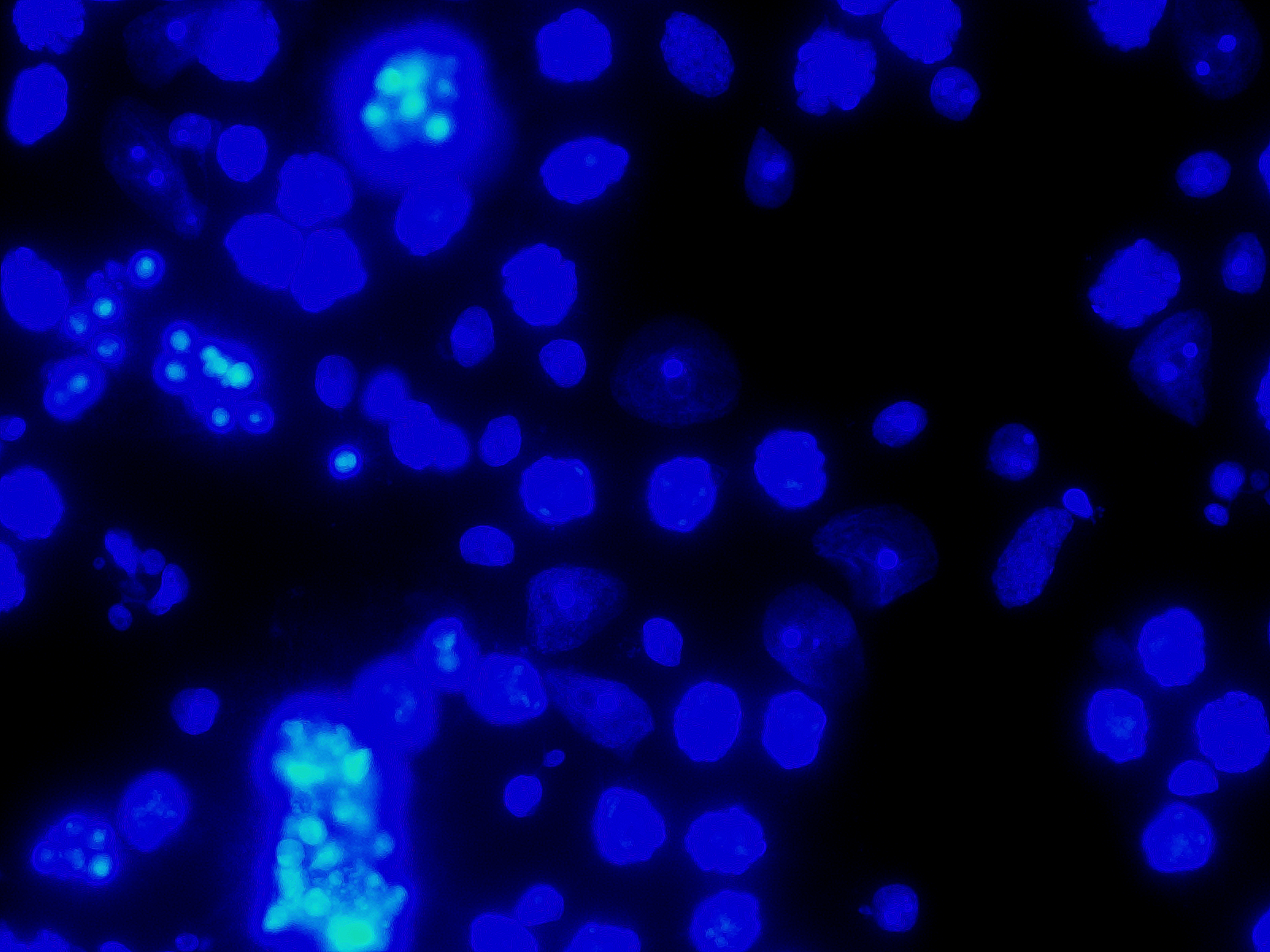

Supplement: Figure 5—source data 1. [file elife-83129-fig5-data1.zip › Figure5/Source data of Figure5B/KO/3/DAPI.tif]

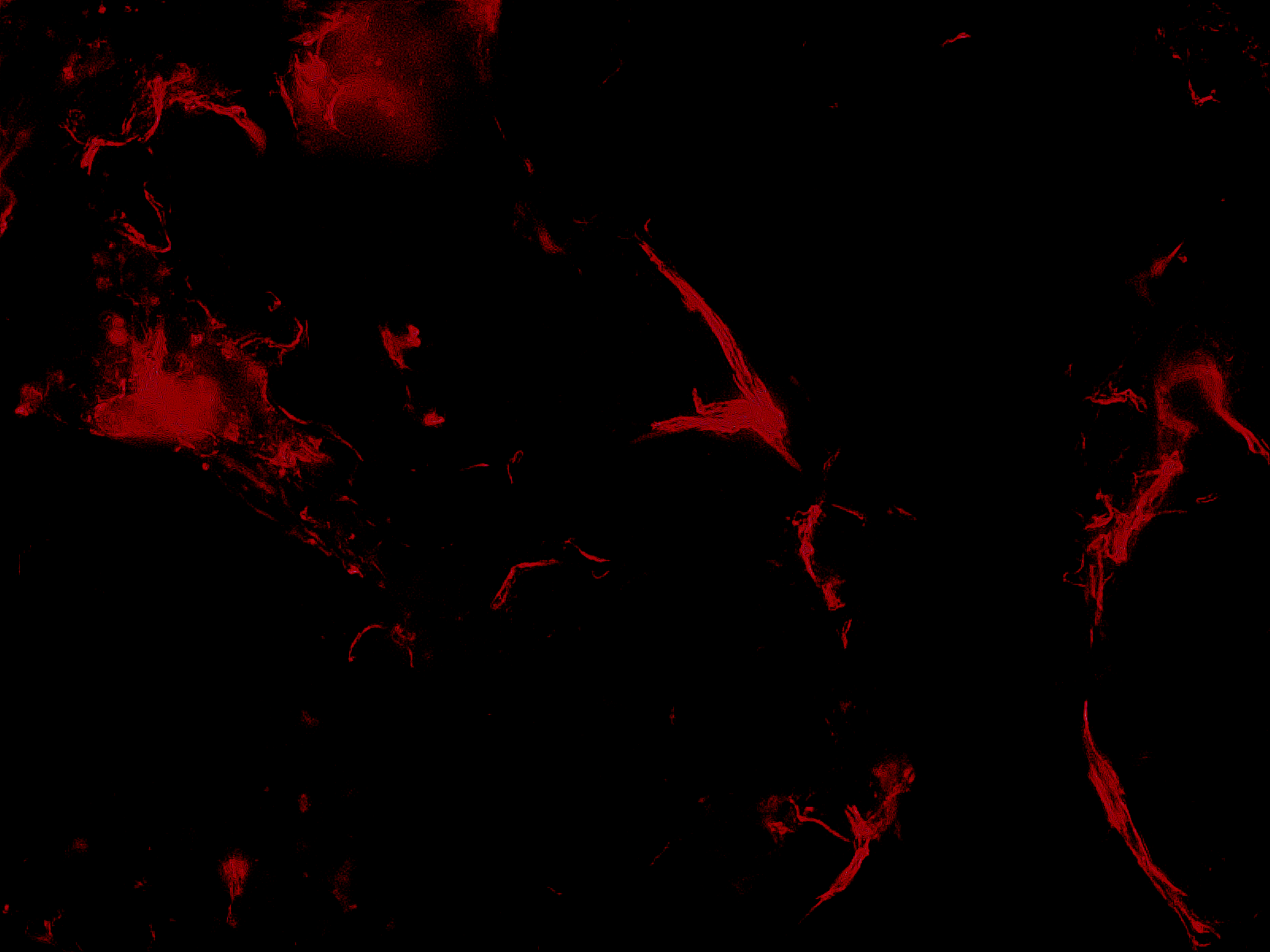

Supplement: Figure 5—source data 1. [file elife-83129-fig5-data1.zip › Figure5/Source data of Figure5B/KO/3/F-actin.tif]

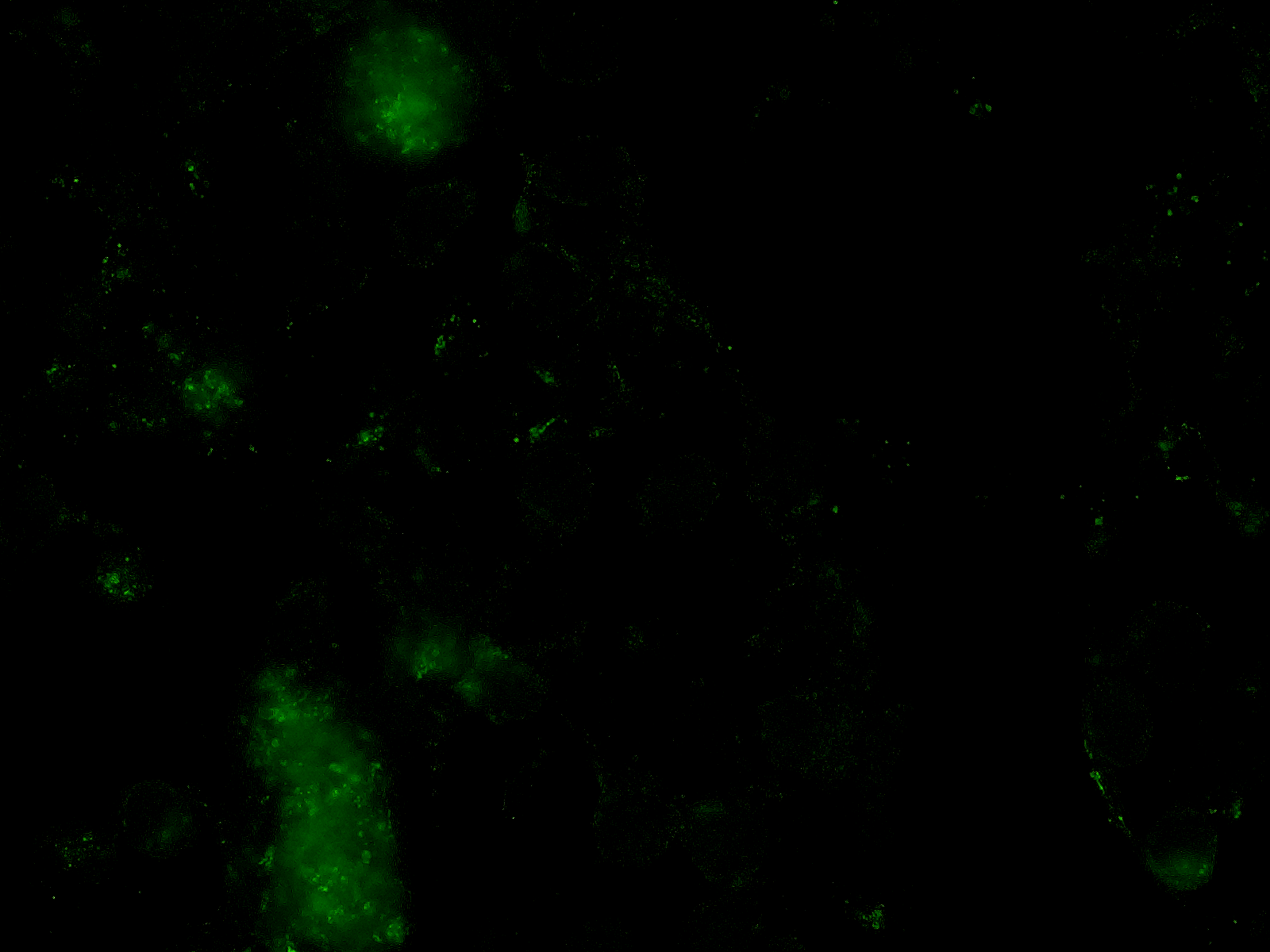

Supplement: Figure 5—source data 1. [file elife-83129-fig5-data1.zip › Figure5/Source data of Figure5B/KO/3/lectin.tif]

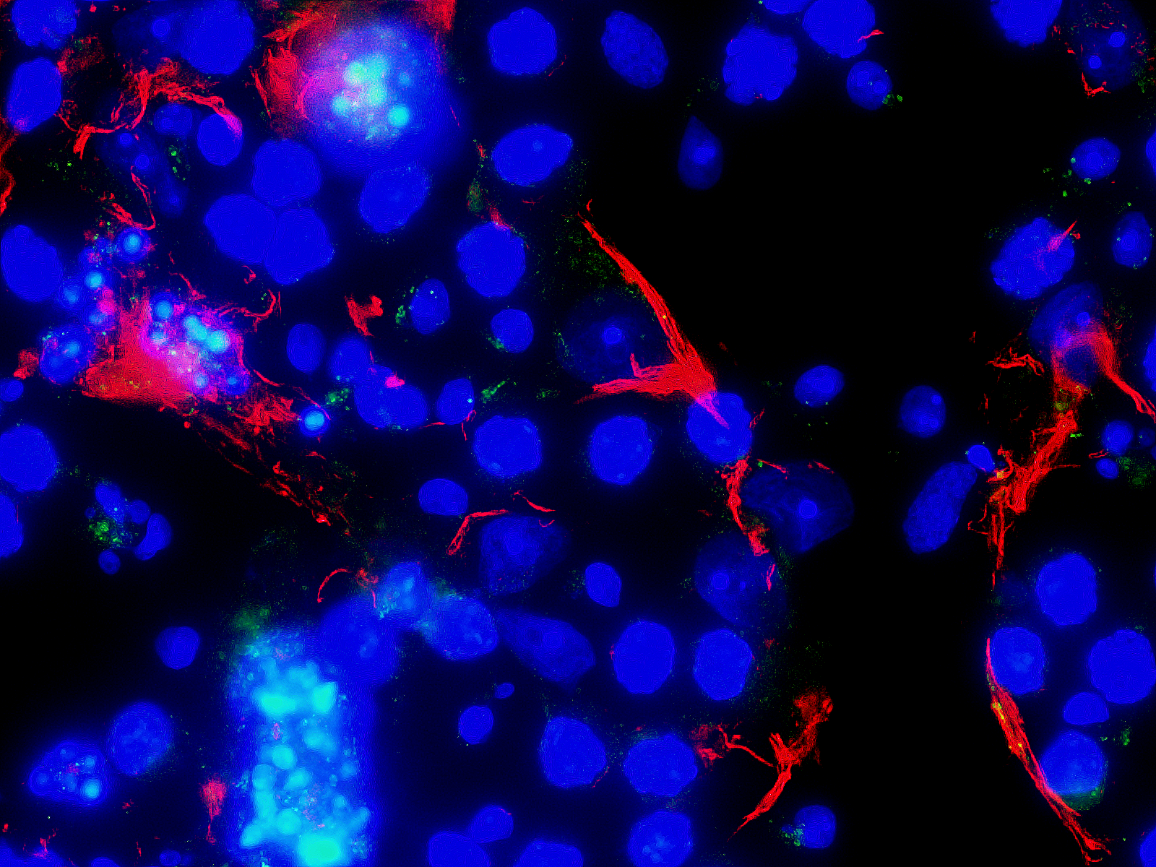

Supplement: Figure 5—source data 1. [file elife-83129-fig5-data1.zip › Figure5/Source data of Figure5B/KO/3/merge.tif]

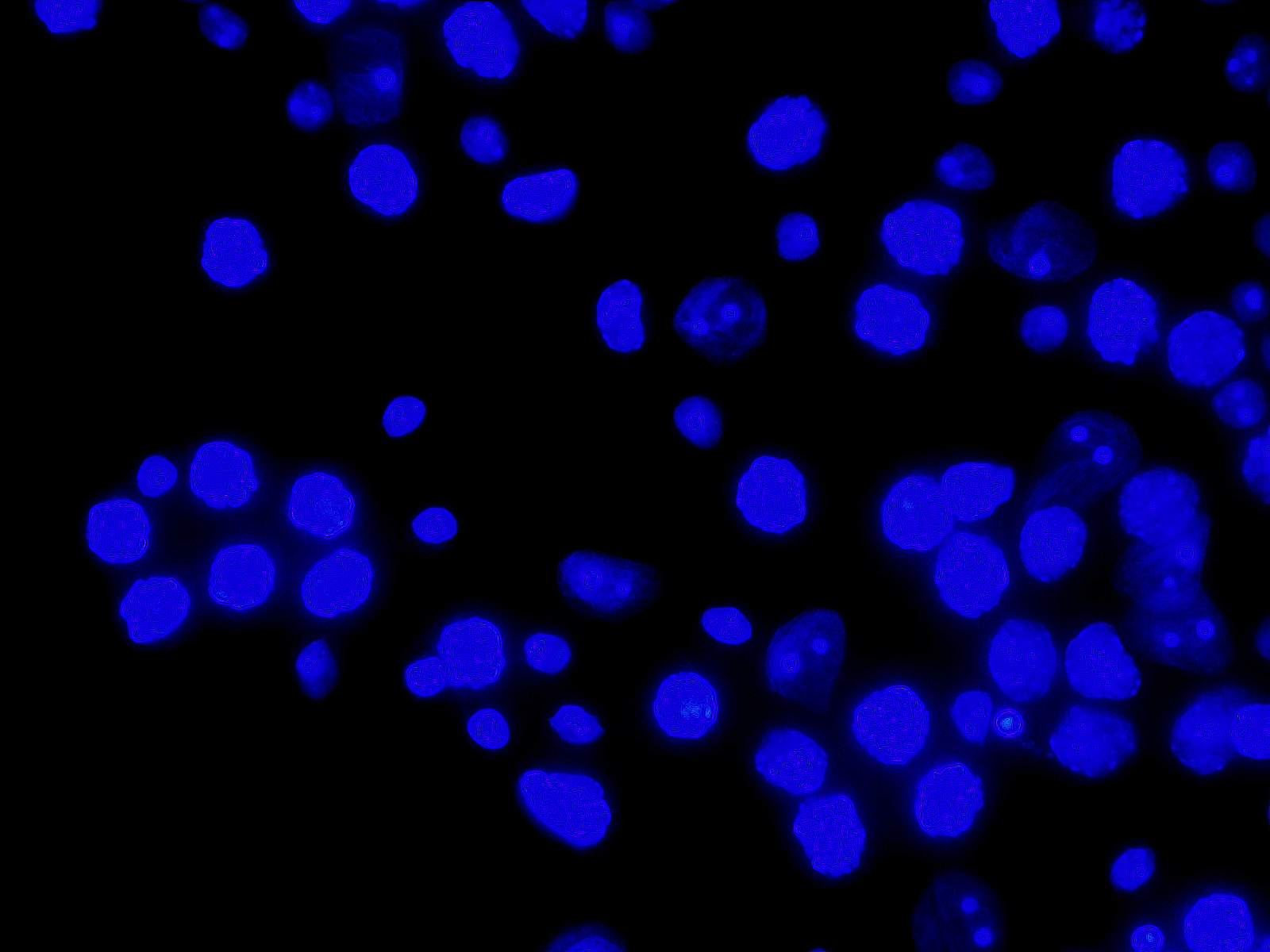

Supplement: Figure 5—source data 1. [file elife-83129-fig5-data1.zip › Figure5/Source data of Figure5B/KO/4/DAPI.tif]

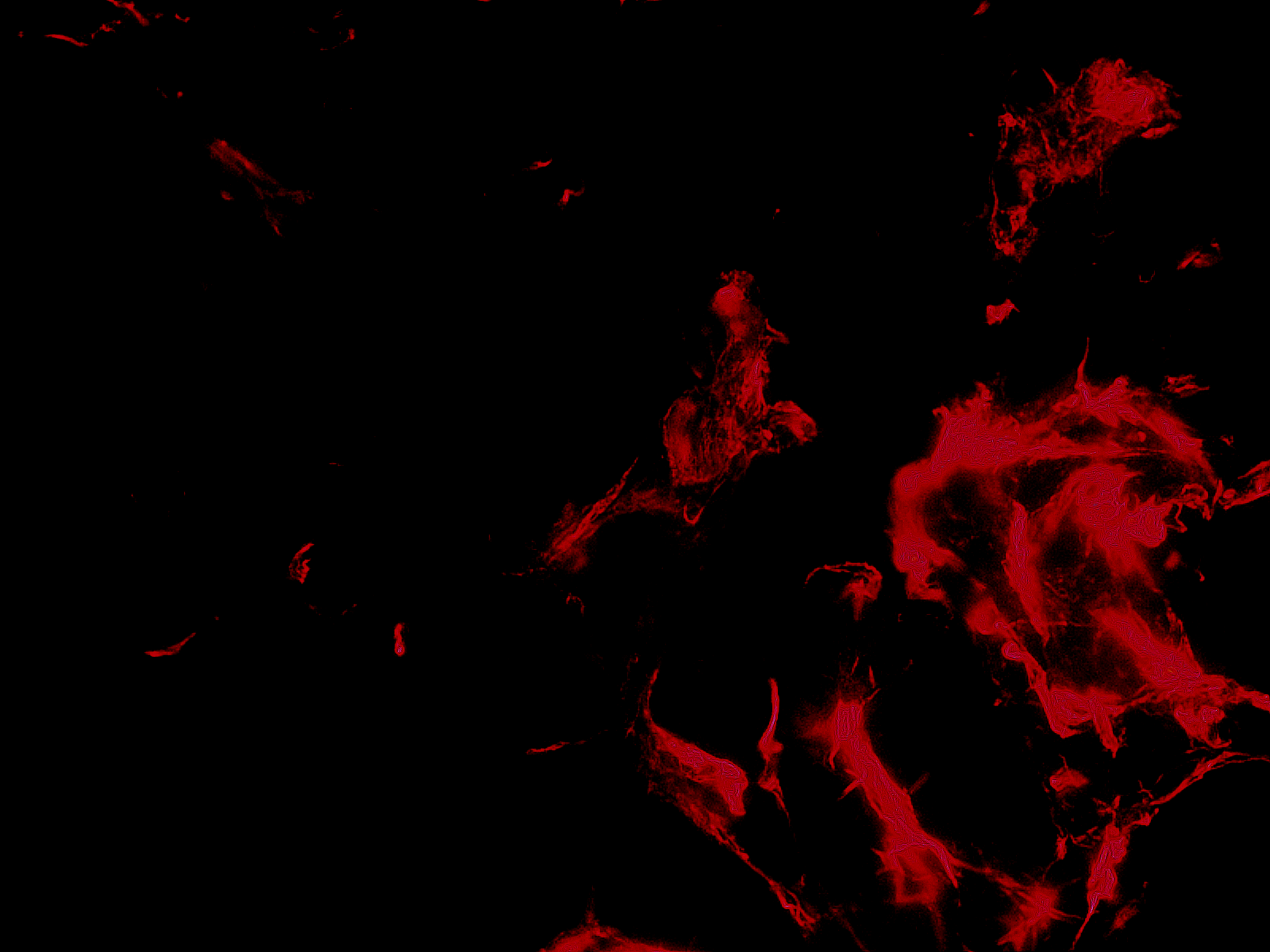

Supplement: Figure 5—source data 1. [file elife-83129-fig5-data1.zip › Figure5/Source data of Figure5B/KO/4/F-actin.tif]

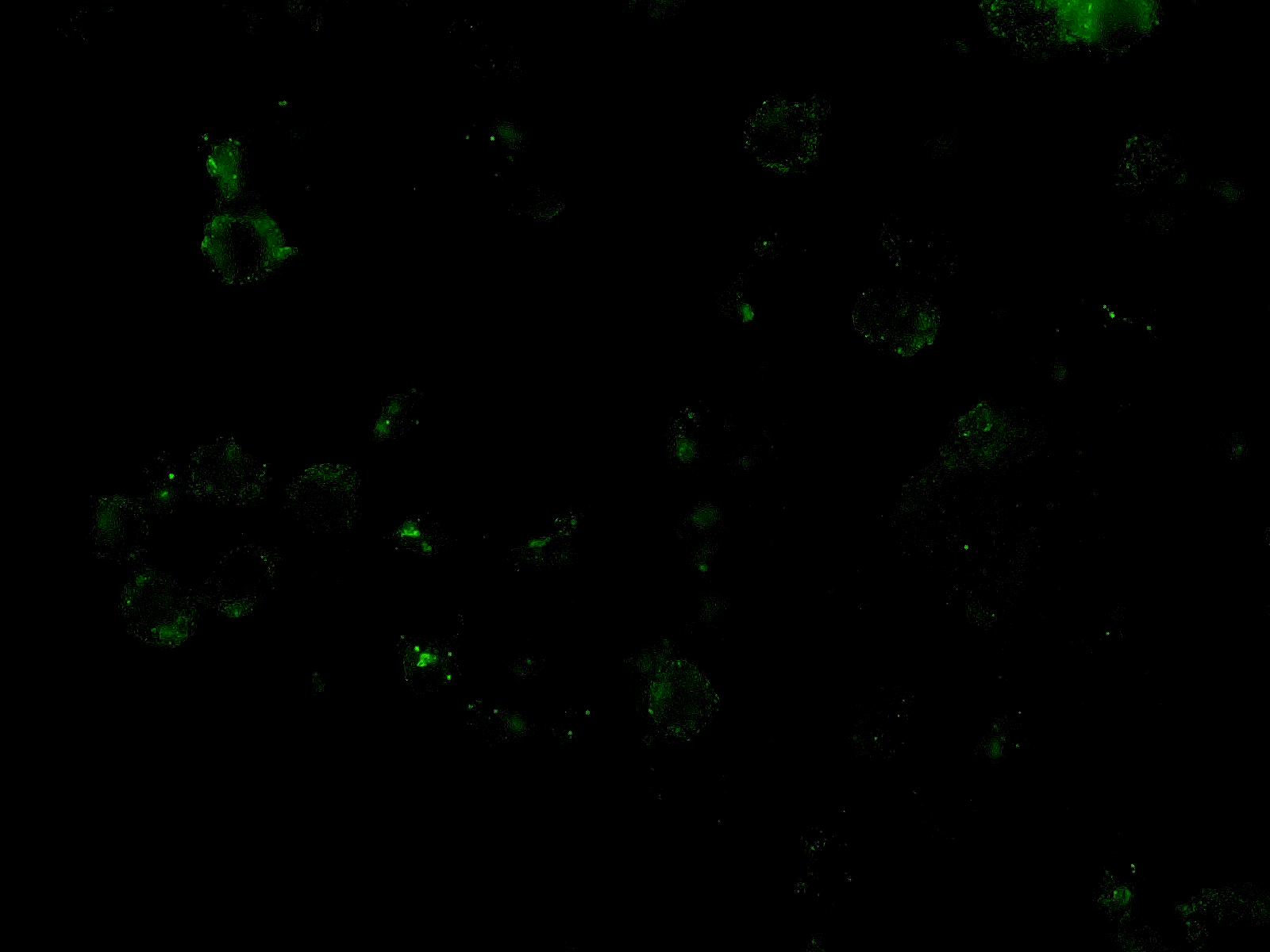

Supplement: Figure 5—source data 1. [file elife-83129-fig5-data1.zip › Figure5/Source data of Figure5B/KO/4/lectin.tif]

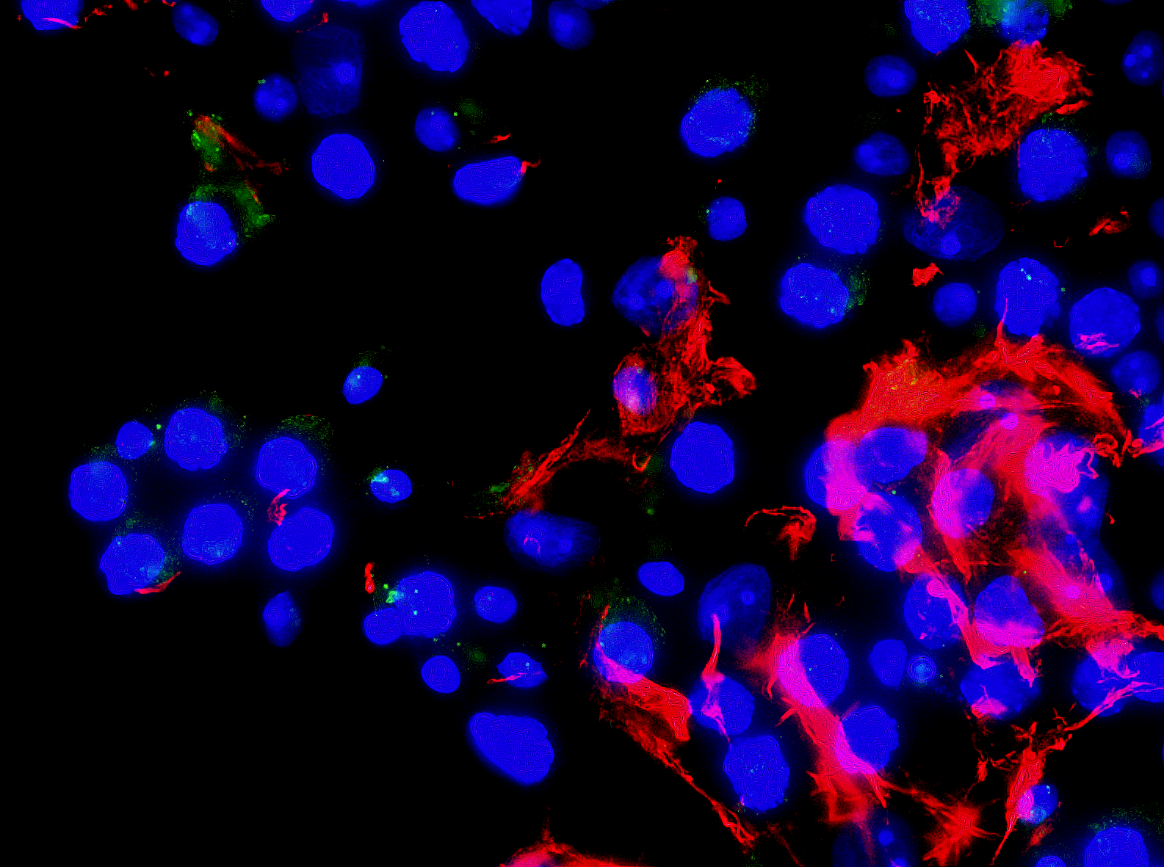

Supplement: Figure 5—source data 1. [file elife-83129-fig5-data1.zip › Figure5/Source data of Figure5B/KO/4/merge.tif]

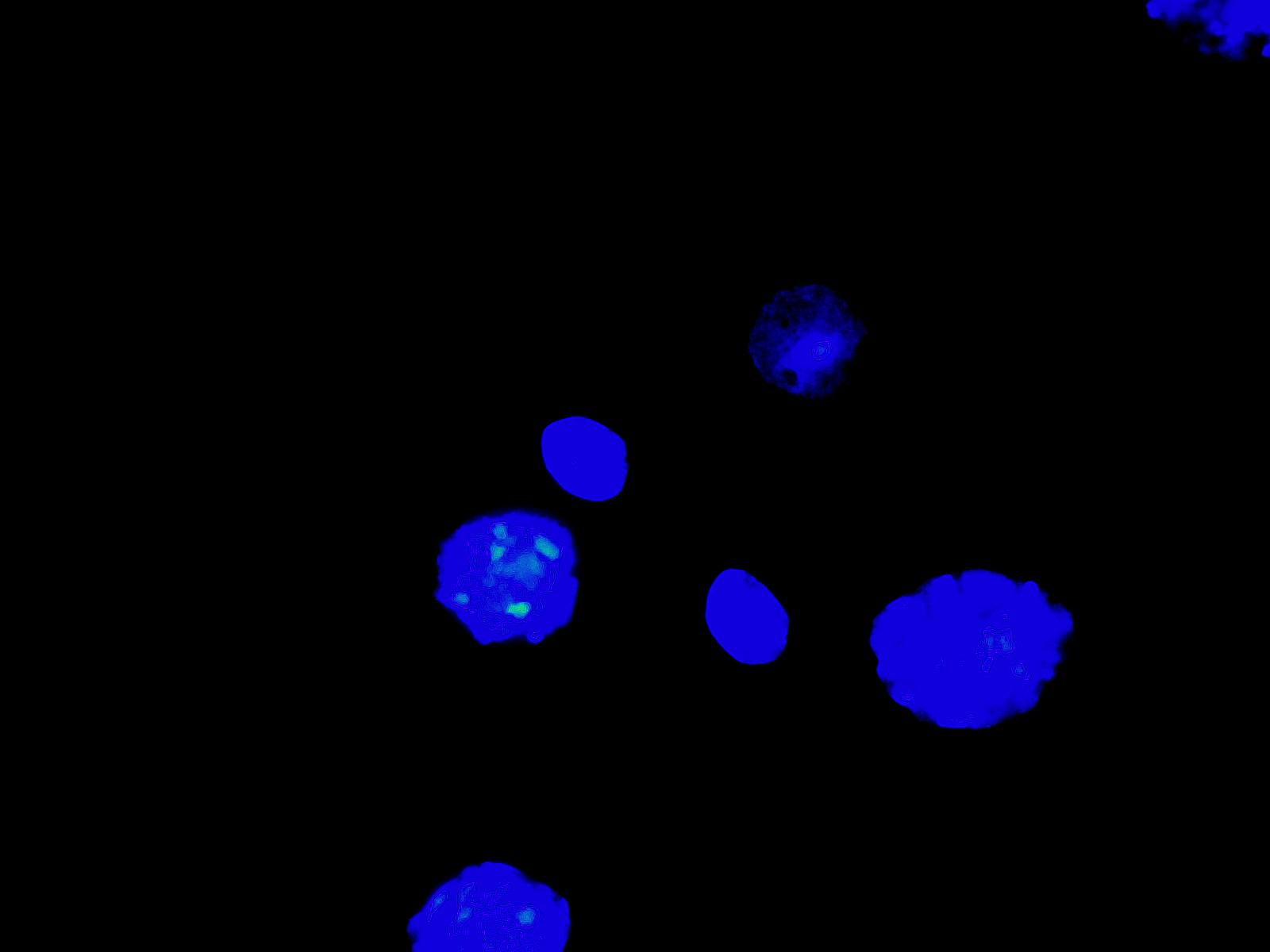

Supplement: Figure 5—source data 1. [file elife-83129-fig5-data1.zip › Figure5/Source data of Figure5B/WT/1/DAPI.tif]

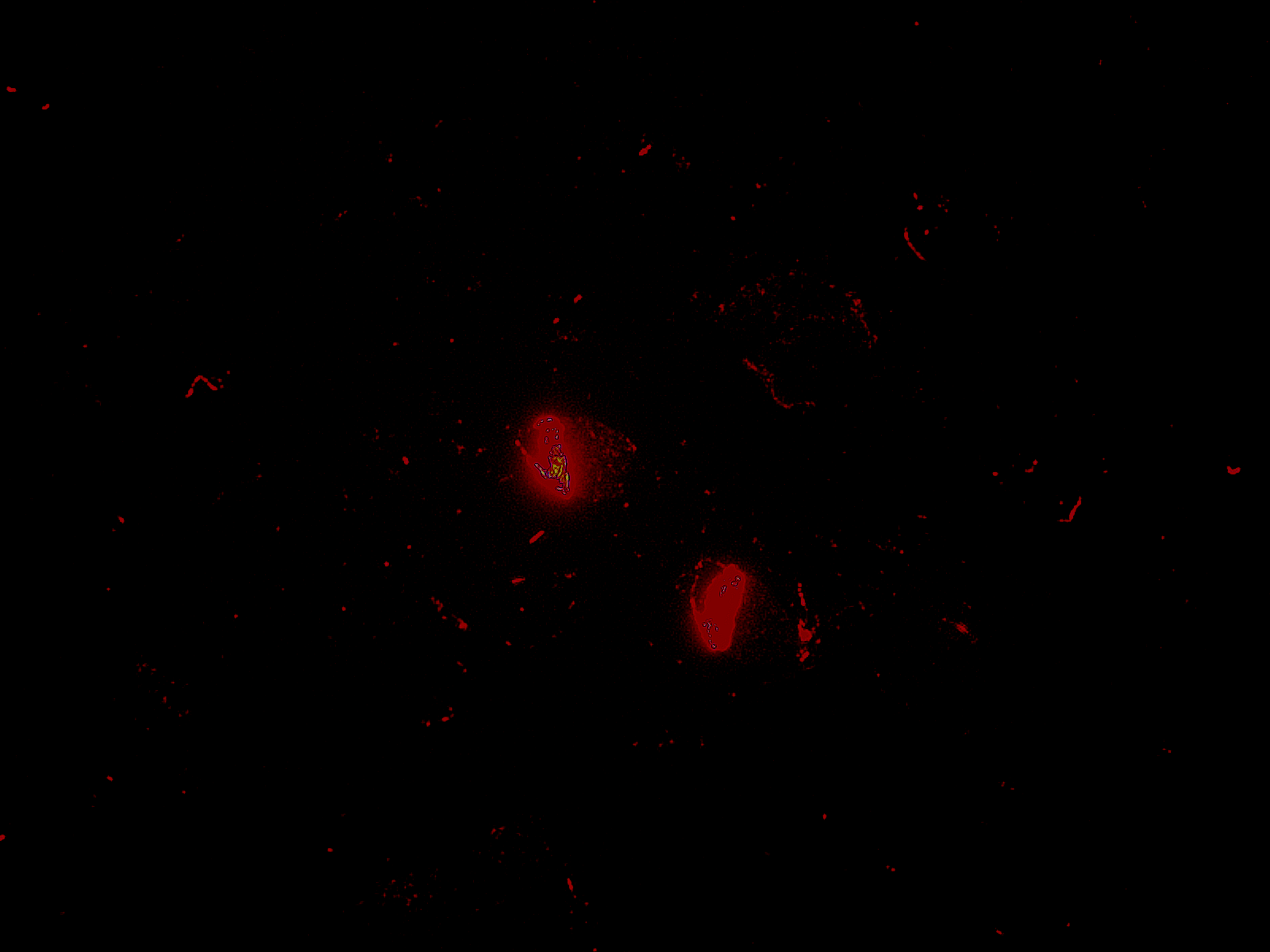

Supplement: Figure 5—source data 1. [file elife-83129-fig5-data1.zip › Figure5/Source data of Figure5B/WT/1/F-actin.tif]

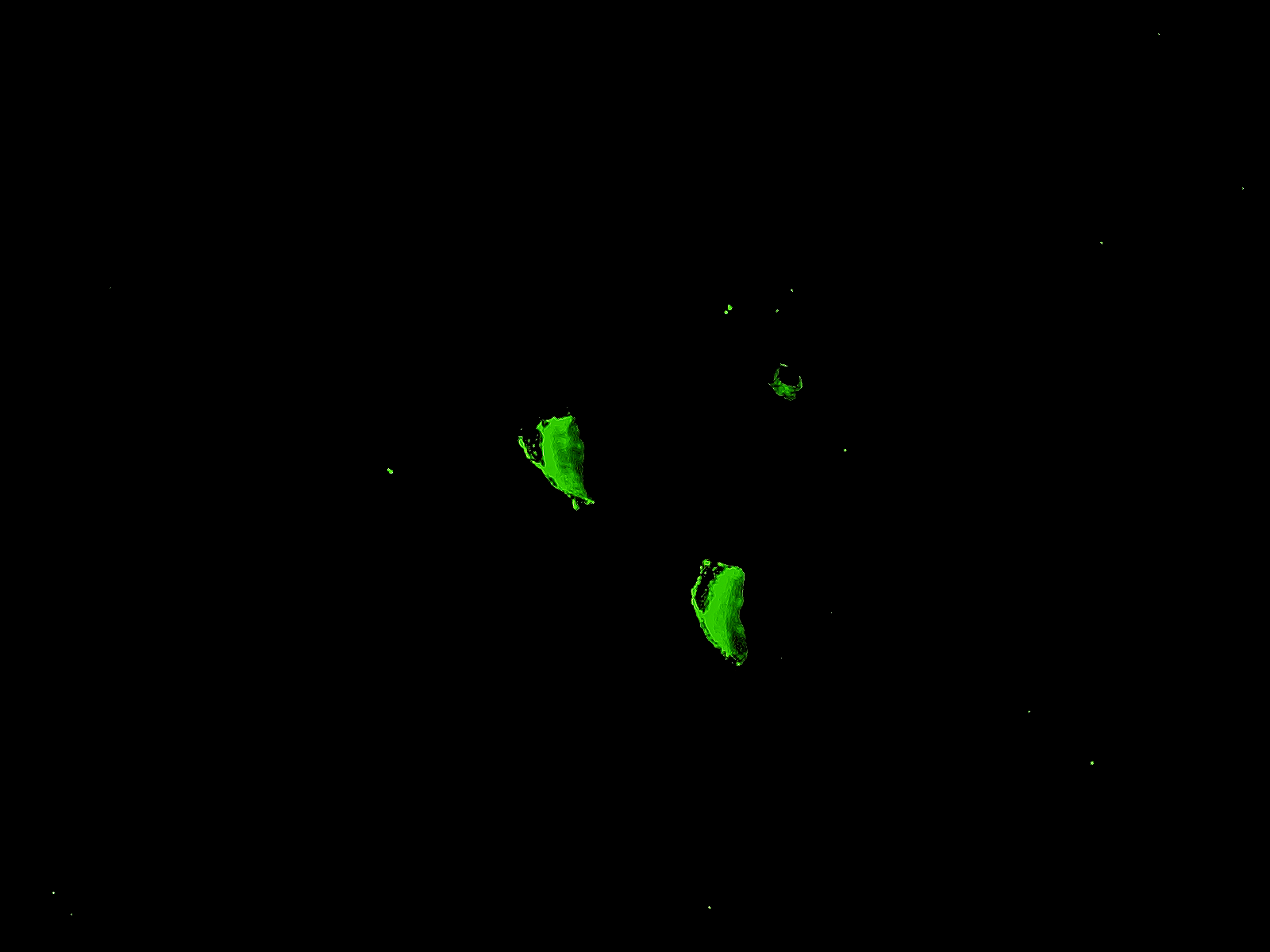

Supplement: Figure 5—source data 1. [file elife-83129-fig5-data1.zip › Figure5/Source data of Figure5B/WT/1/lectin.tif]

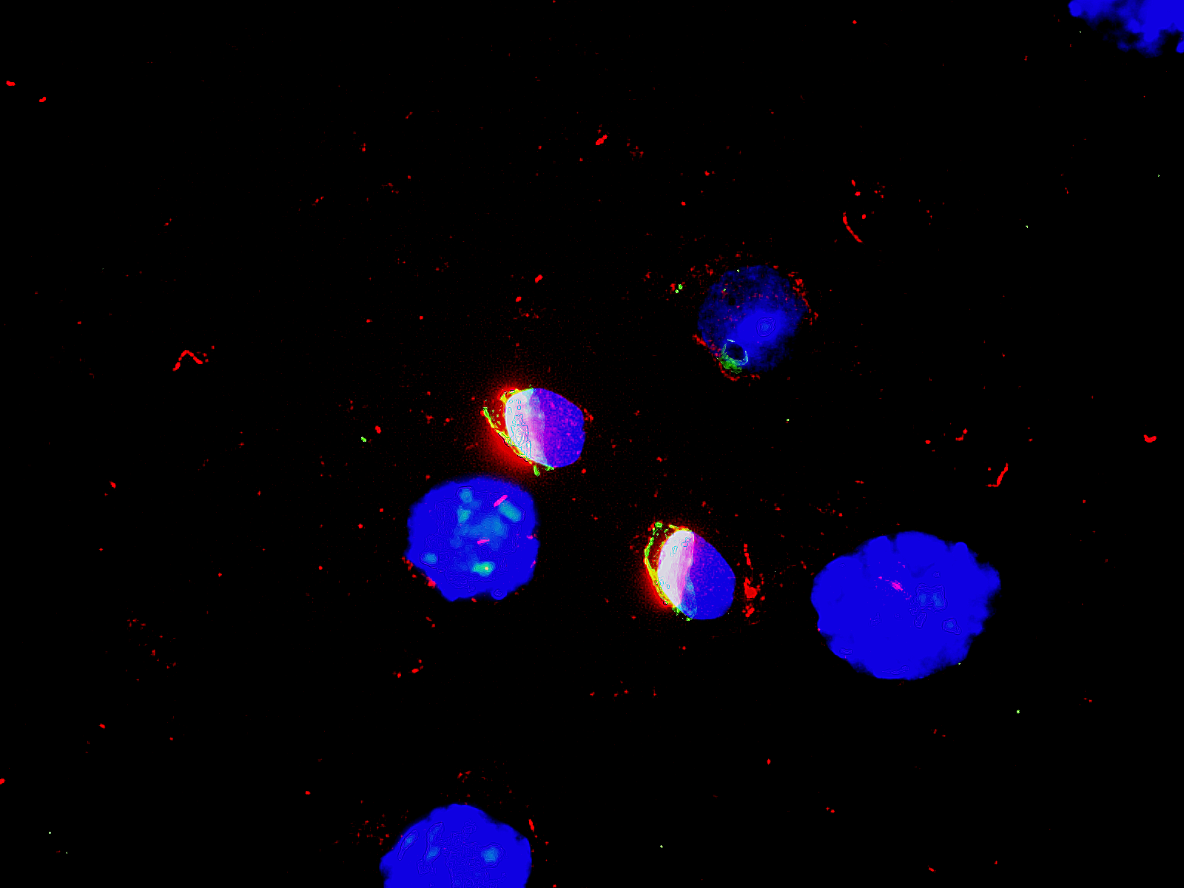

Supplement: Figure 5—source data 1. [file elife-83129-fig5-data1.zip › Figure5/Source data of Figure5B/WT/1/merge.tif]

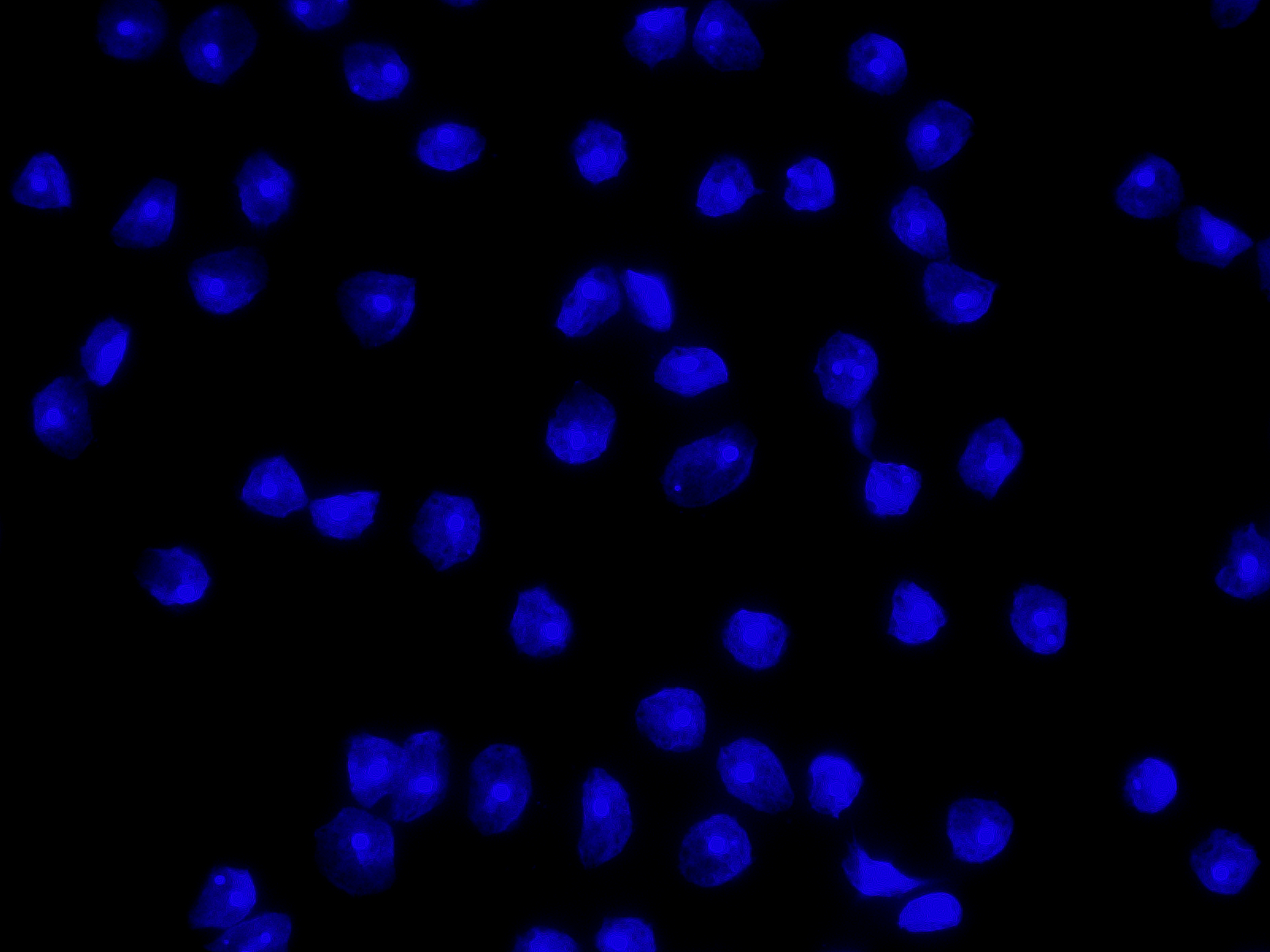

Supplement: Figure 5—source data 1. [file elife-83129-fig5-data1.zip › Figure5/Source data of Figure5B/WT/2/DAPI.tif]

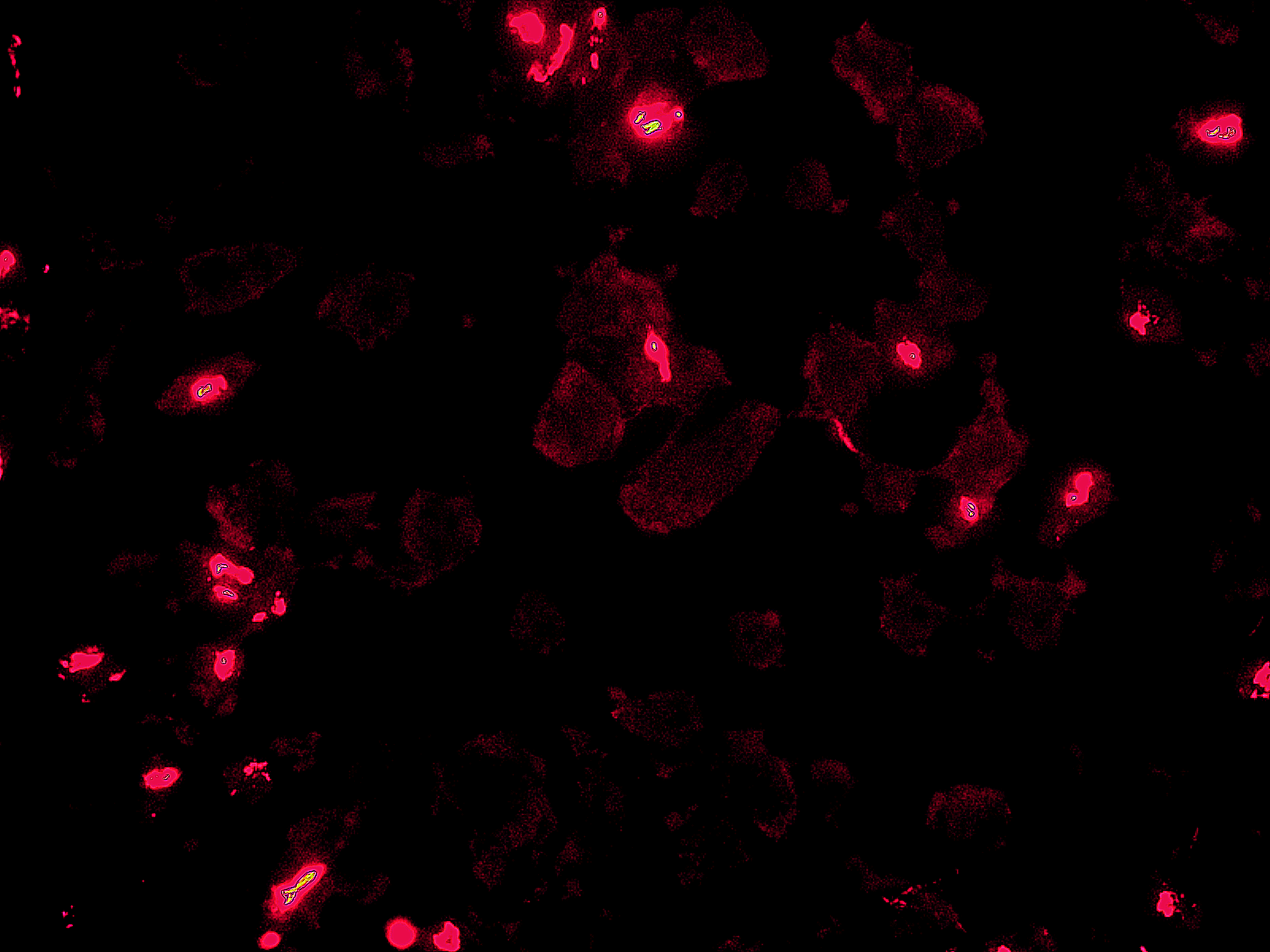

Supplement: Figure 5—source data 1. [file elife-83129-fig5-data1.zip › Figure5/Source data of Figure5B/WT/2/F-actin.tif]

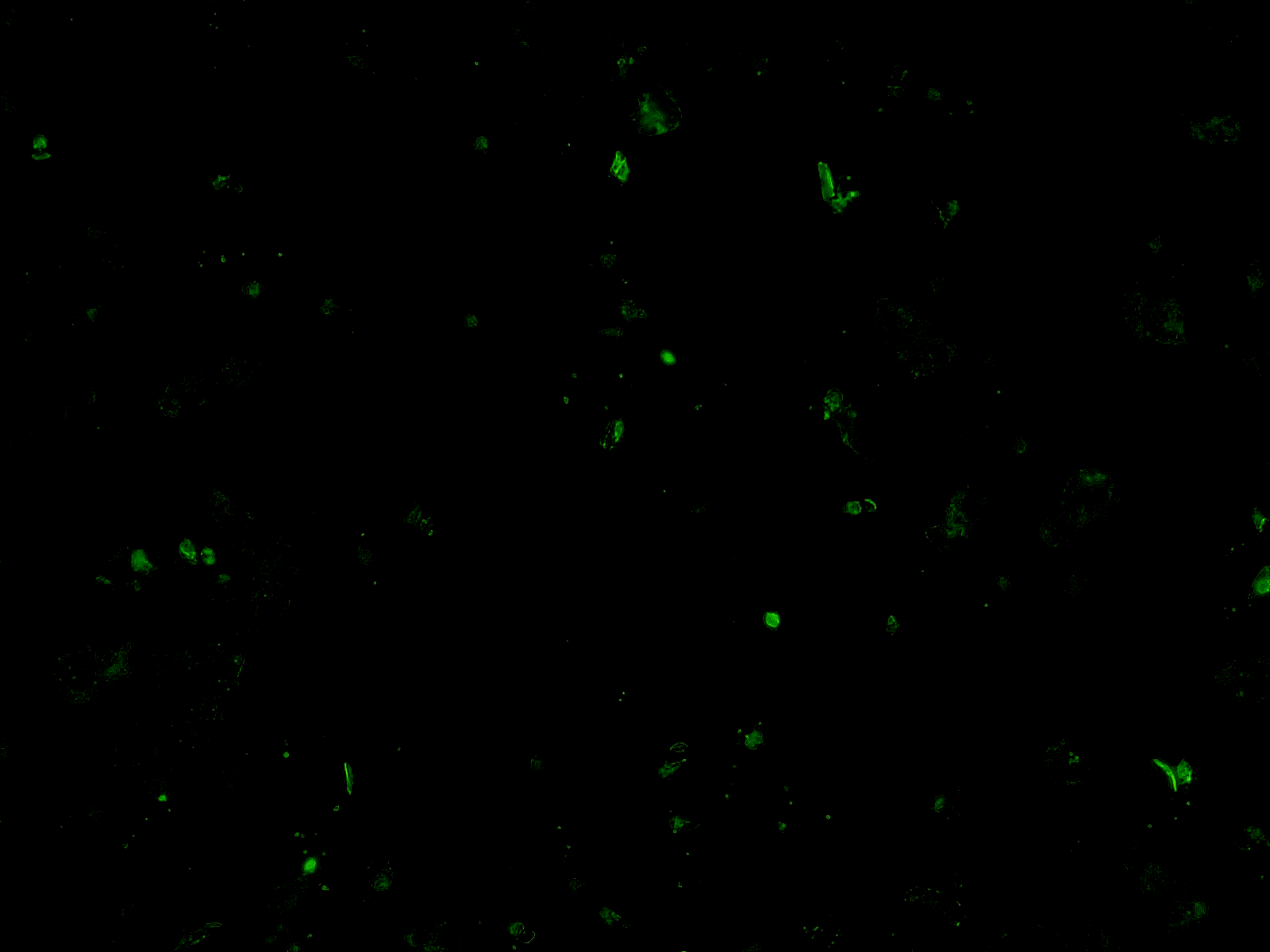

Supplement: Figure 5—source data 1. [file elife-83129-fig5-data1.zip › Figure5/Source data of Figure5B/WT/2/lectin.tif]

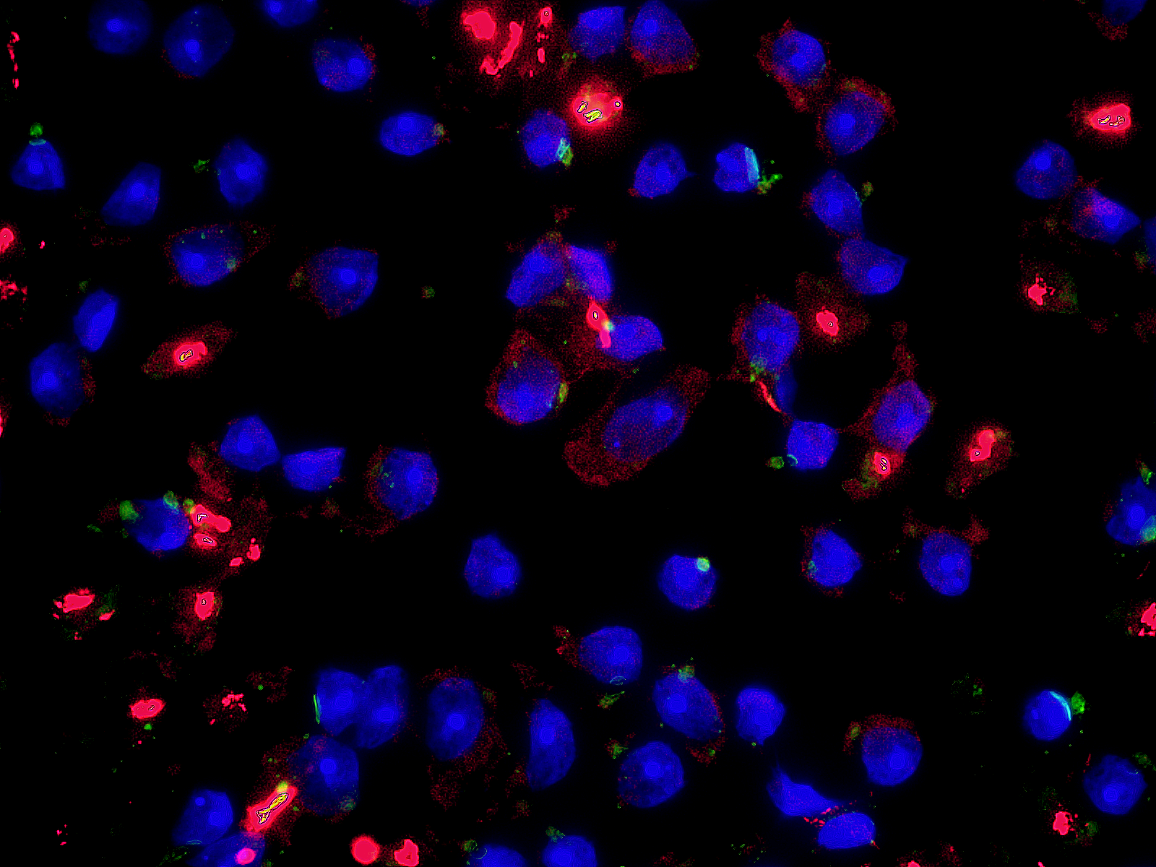

Supplement: Figure 5—source data 1. [file elife-83129-fig5-data1.zip › Figure5/Source data of Figure5B/WT/2/merge.tif]

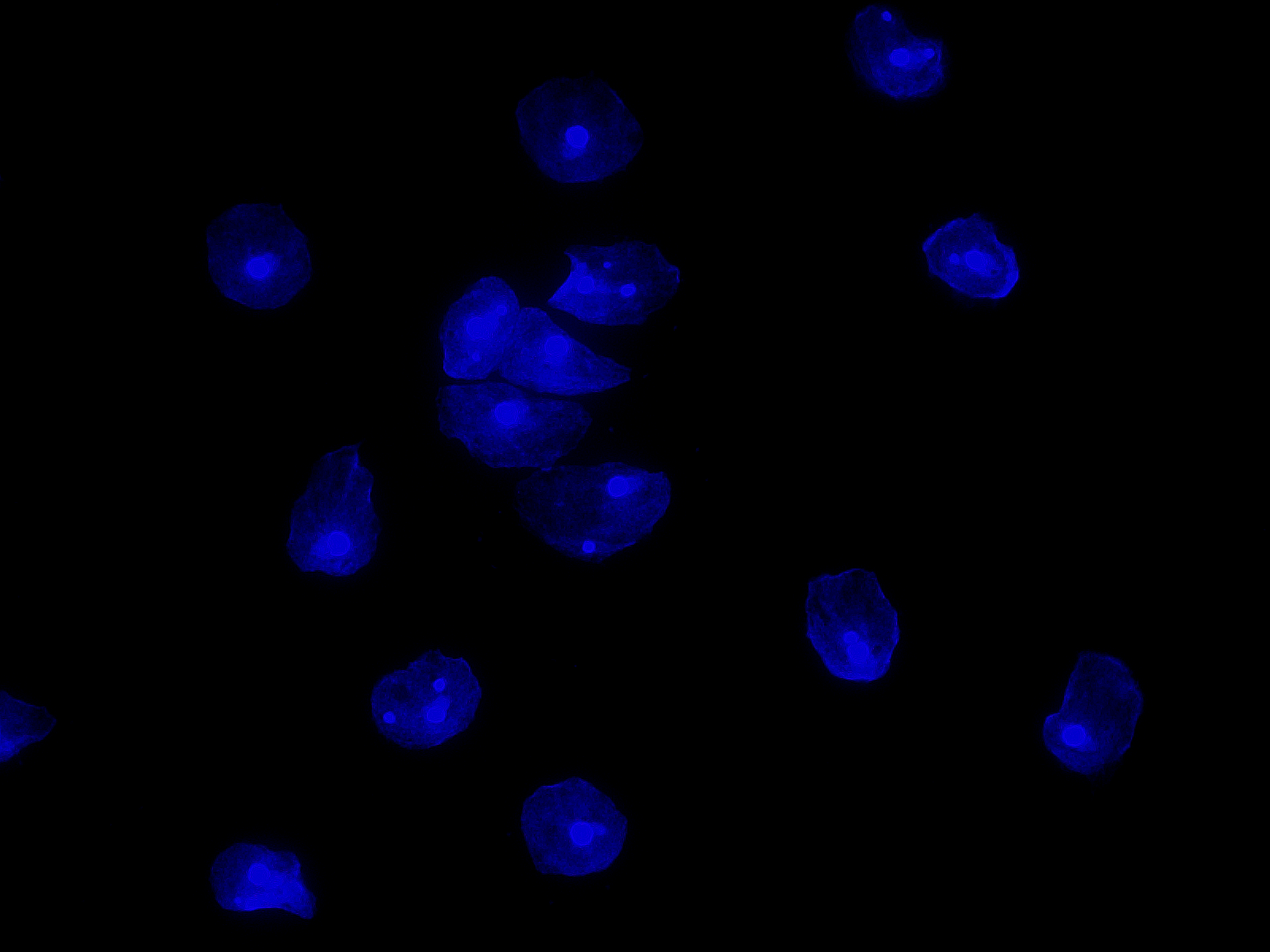

Supplement: Figure 5—source data 1. [file elife-83129-fig5-data1.zip › Figure5/Source data of Figure5B/WT/3/DAPI.tif]

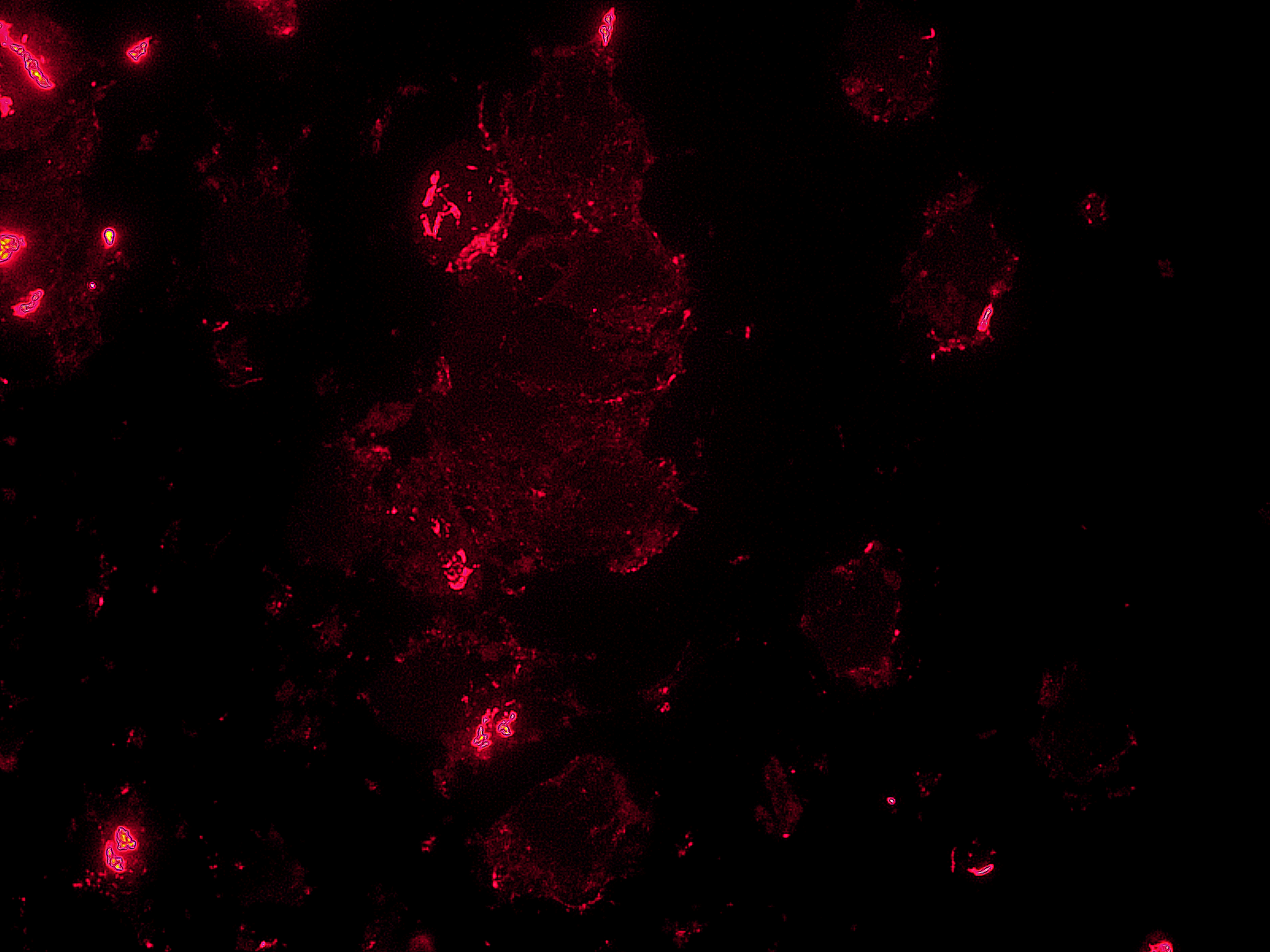

Supplement: Figure 5—source data 1. [file elife-83129-fig5-data1.zip › Figure5/Source data of Figure5B/WT/3/F-actin.tif]

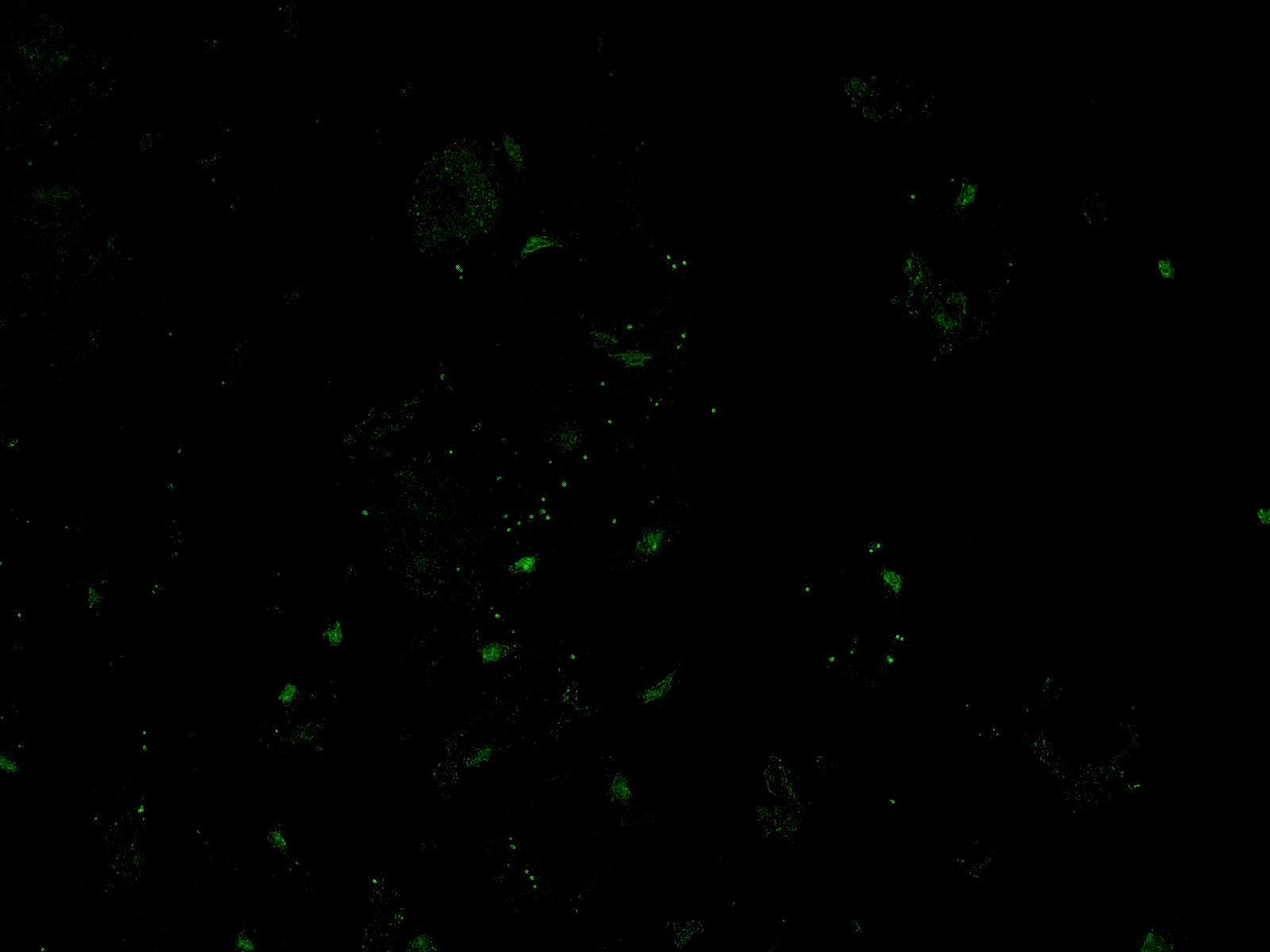

Supplement: Figure 5—source data 1. [file elife-83129-fig5-data1.zip › Figure5/Source data of Figure5B/WT/3/lectin.tif]

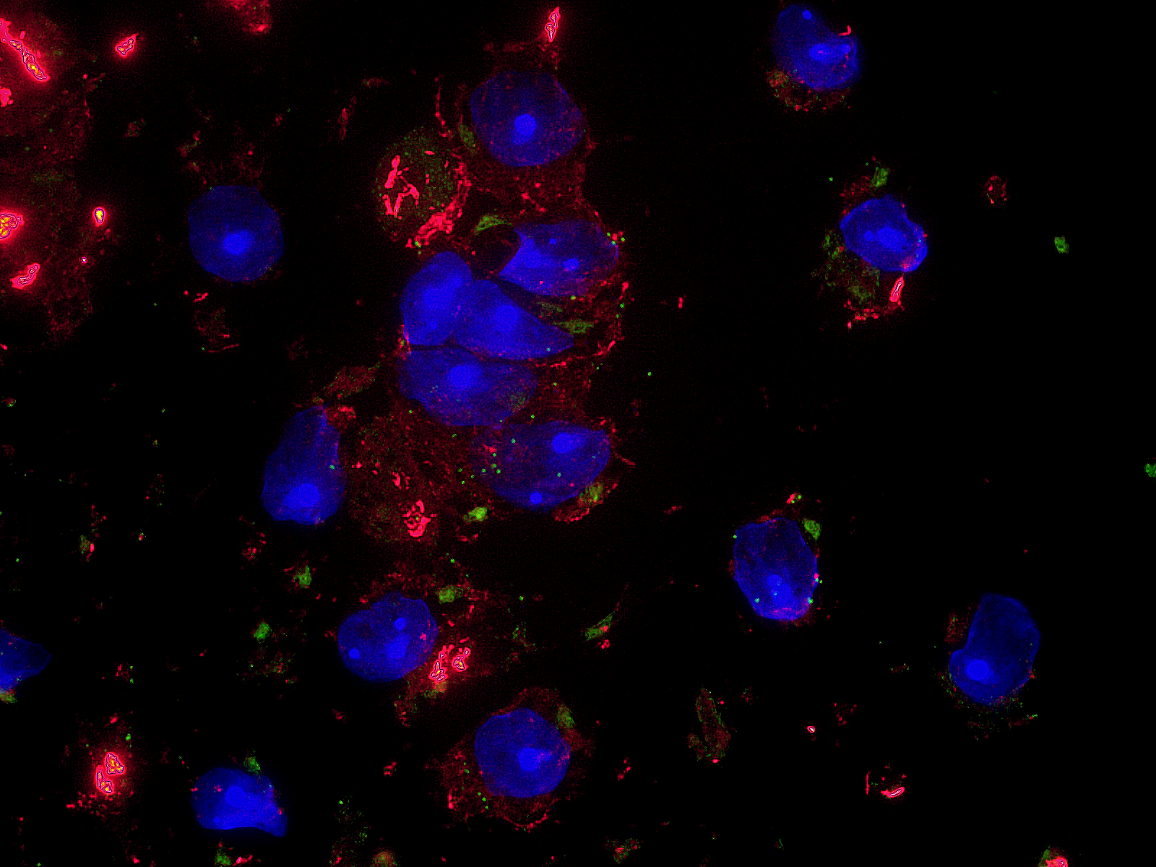

Supplement: Figure 5—source data 1. [file elife-83129-fig5-data1.zip › Figure5/Source data of Figure5B/WT/3/merge.tif]

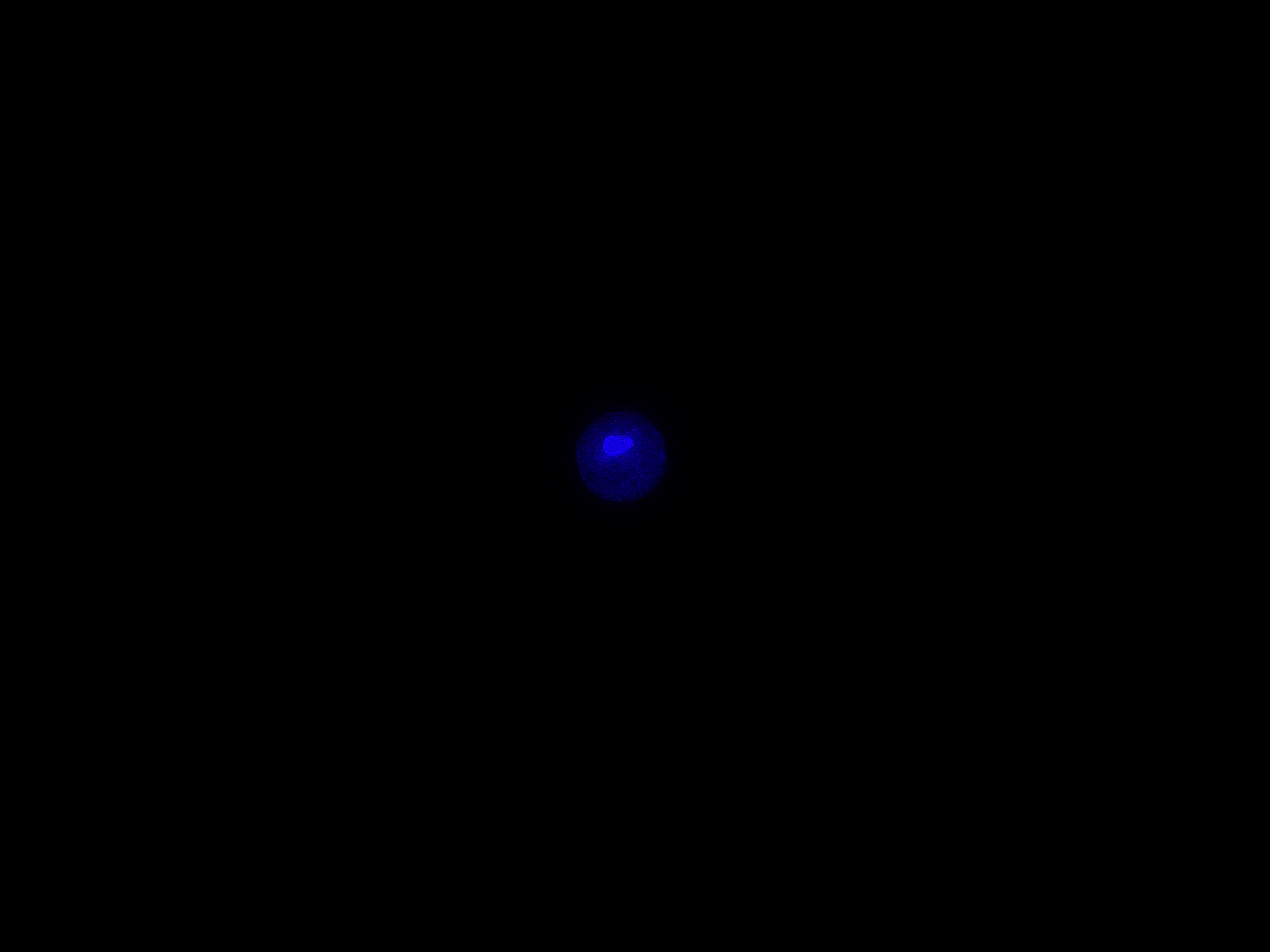

Supplement: Figure 5—source data 1. [file elife-83129-fig5-data1.zip › Figure5/Source data of Figure5B/WT/4/DAPI.tif]

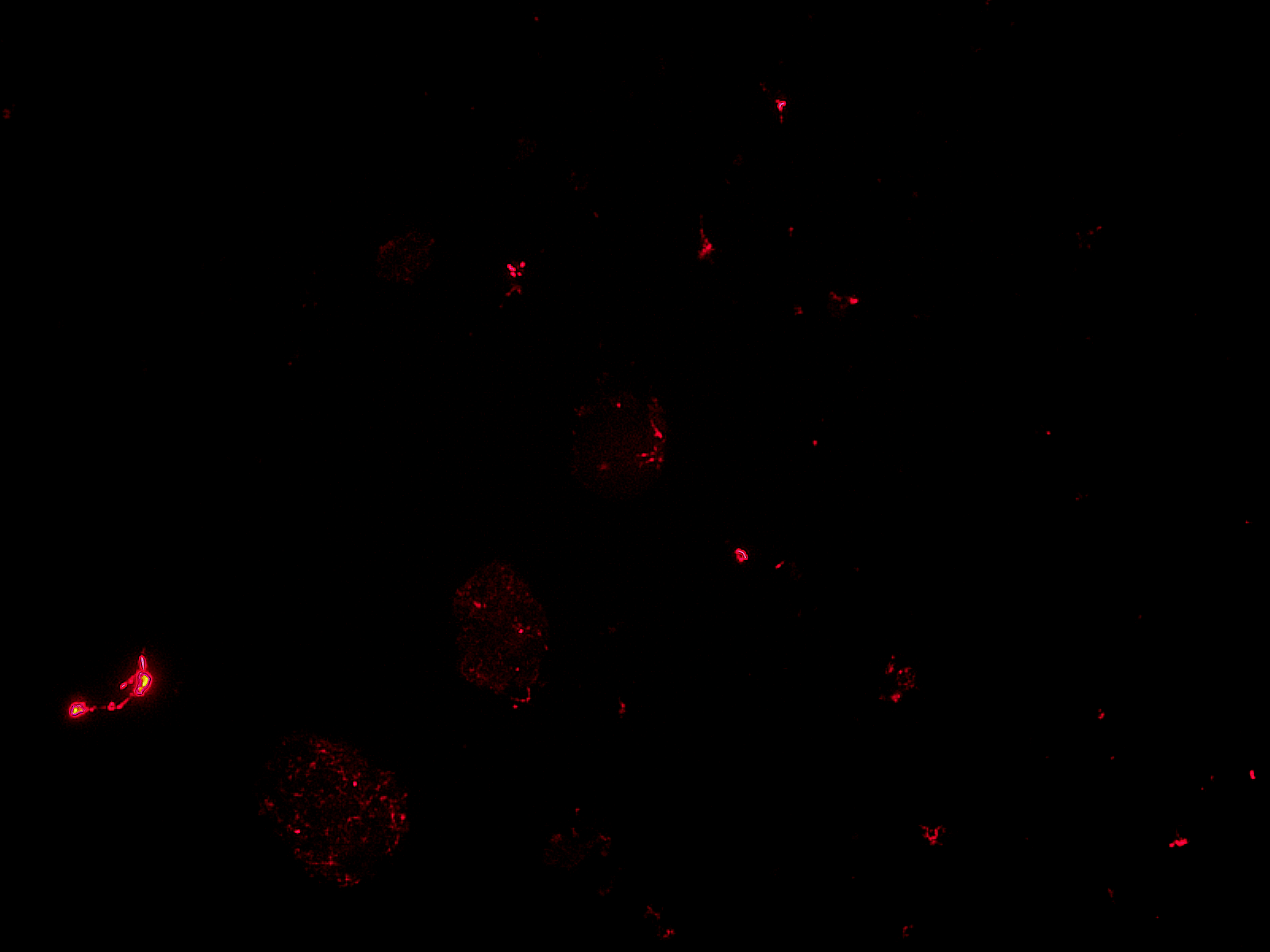

Supplement: Figure 5—source data 1. [file elife-83129-fig5-data1.zip › Figure5/Source data of Figure5B/WT/4/F-actin.tif]

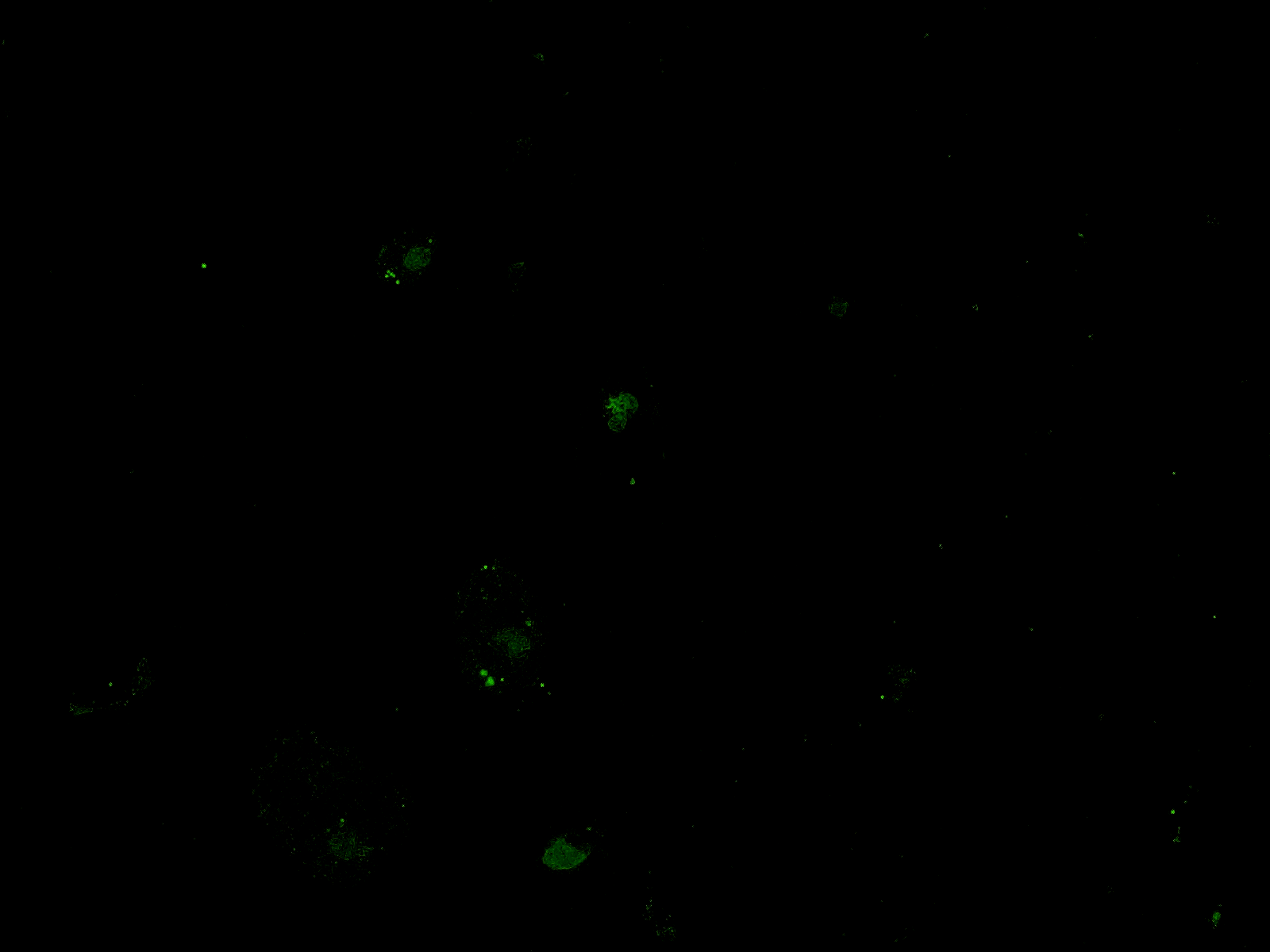

Supplement: Figure 5—source data 1. [file elife-83129-fig5-data1.zip › Figure5/Source data of Figure5B/WT/4/lectin.tif]

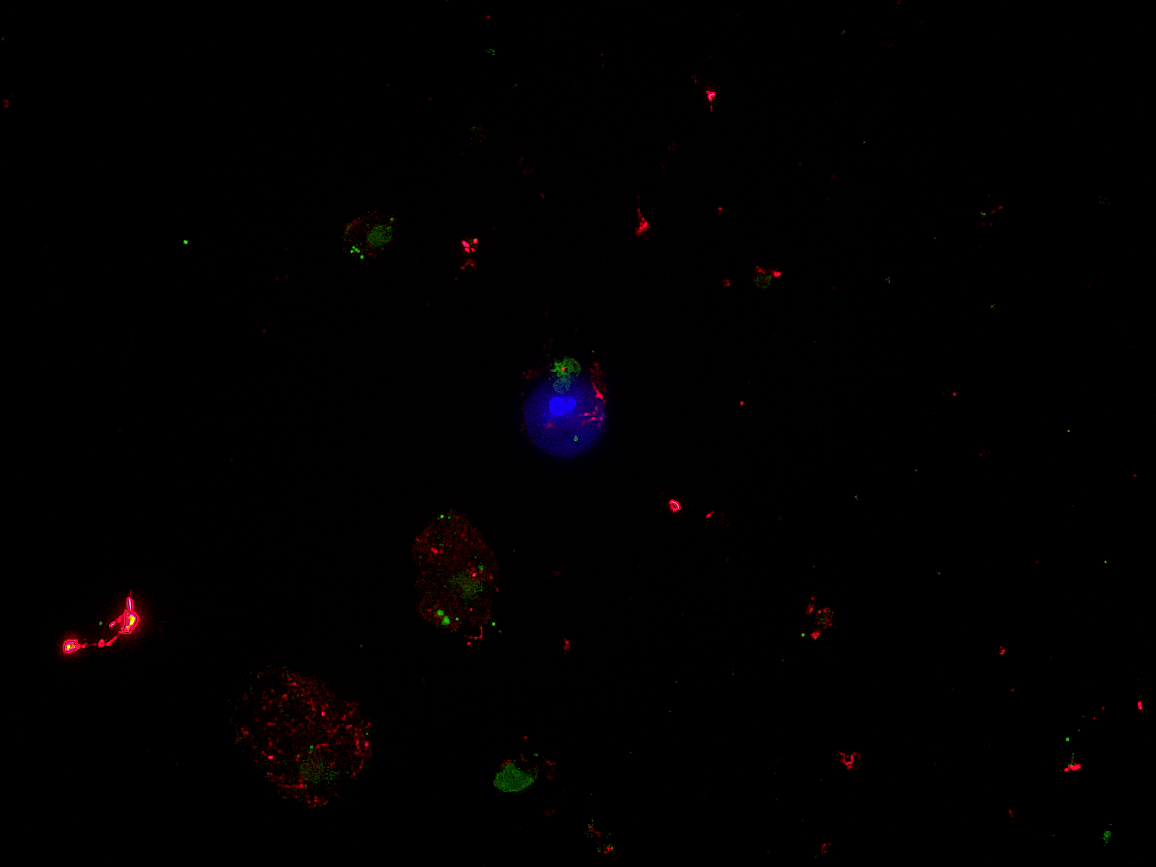

Supplement: Figure 5—source data 1. [file elife-83129-fig5-data1.zip › Figure5/Source data of Figure5B/WT/4/merge.tif]

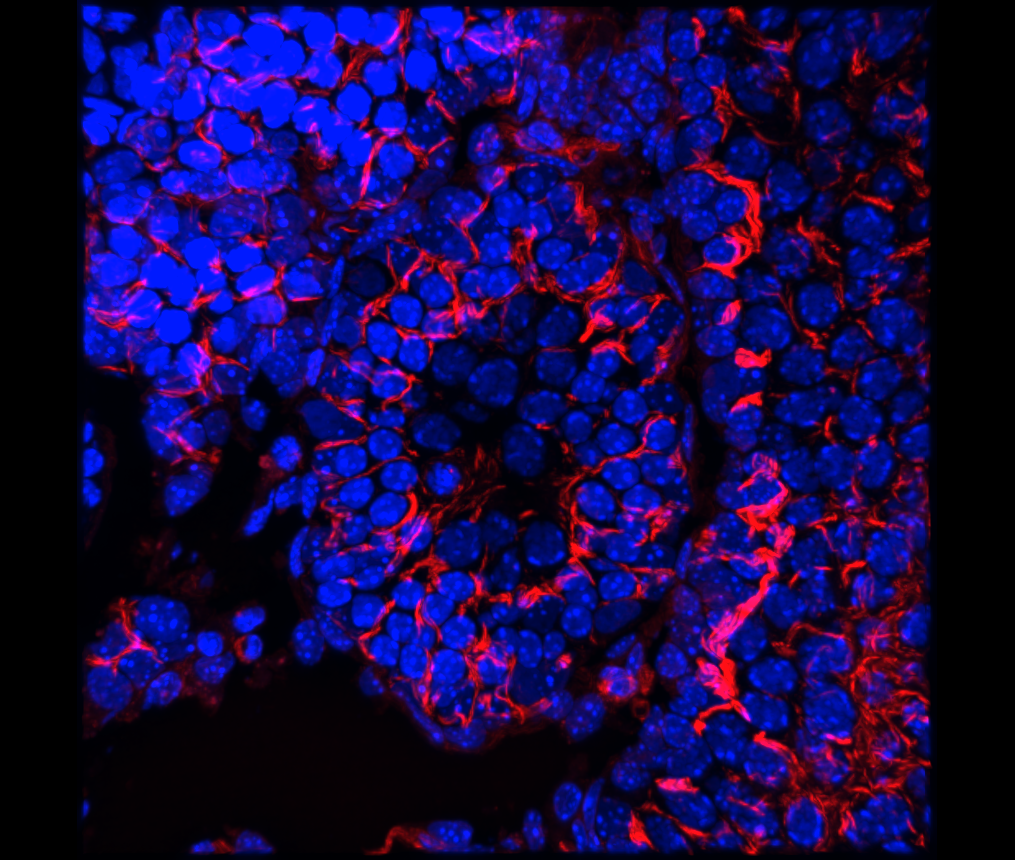

Supplement: Figure 5—figure supplement 1—source data 1. [file elife-83129-fig5-figsupp1-data1.zip › Figure supplement S5-source data 12/F-actin staining/hs-pd21-ko_1.tif]

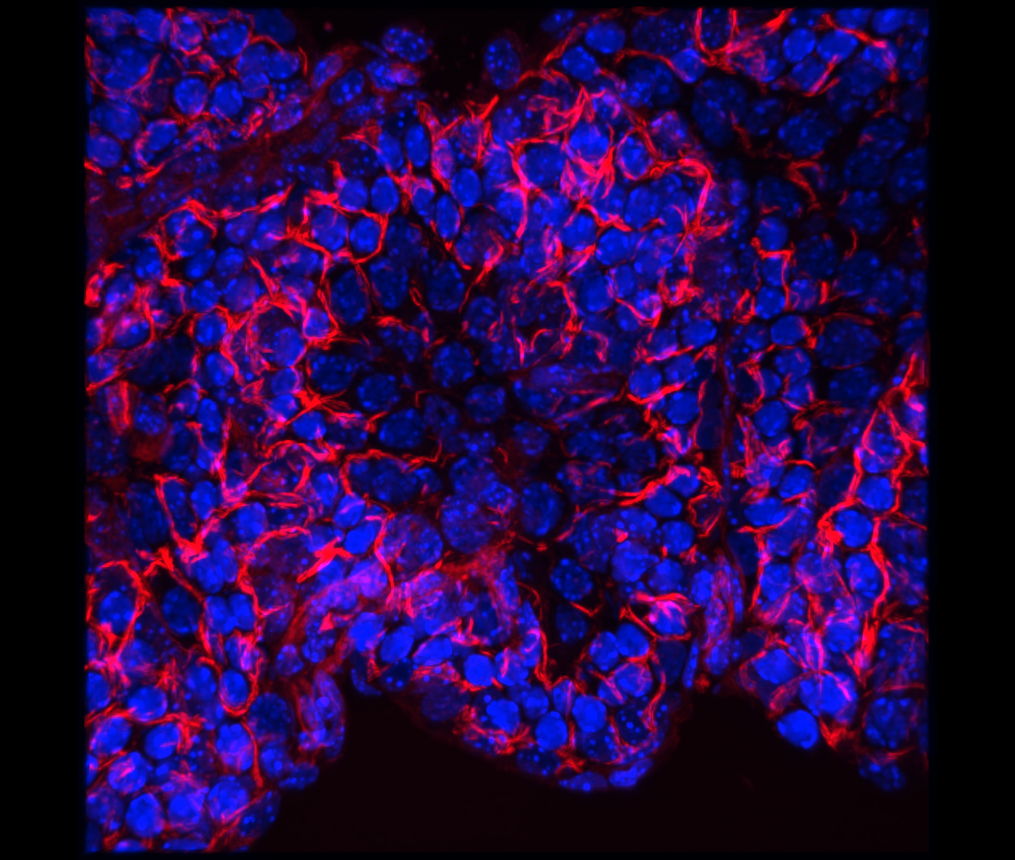

Supplement: Figure 5—figure supplement 1—source data 1. [file elife-83129-fig5-figsupp1-data1.zip › Figure supplement S5-source data 12/F-actin staining/hs-pd21-ko_2.tif]

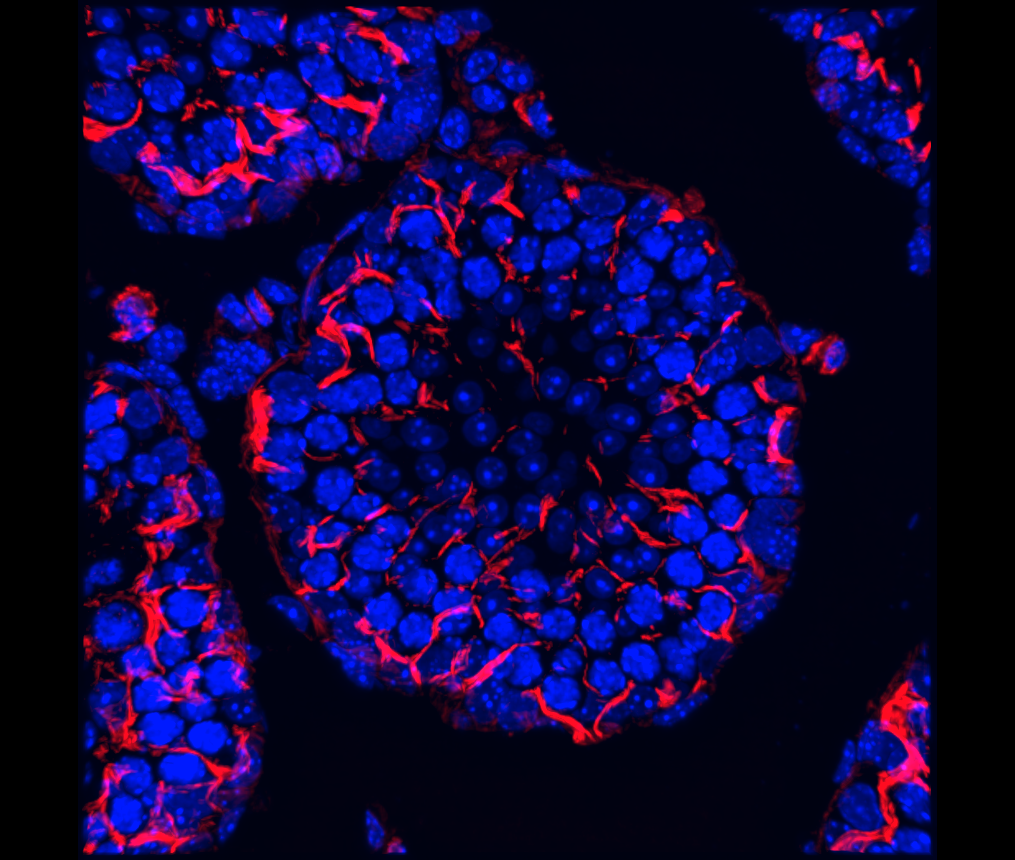

Supplement: Figure 5—figure supplement 1—source data 1. [file elife-83129-fig5-figsupp1-data1.zip › Figure supplement S5-source data 12/F-actin staining/hs-pd21-wt_1.tif]

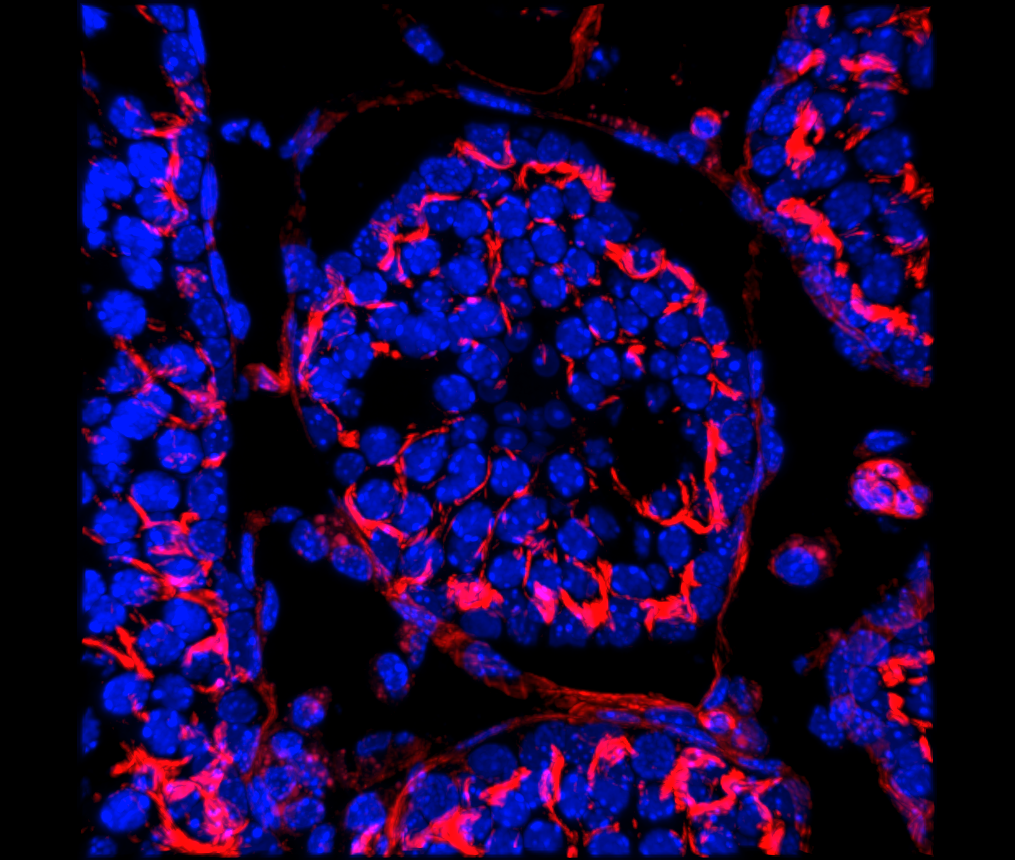

Supplement: Figure 5—figure supplement 1—source data 1. [file elife-83129-fig5-figsupp1-data1.zip › Figure supplement S5-source data 12/F-actin staining/hs-pd21-wt_2.tif]

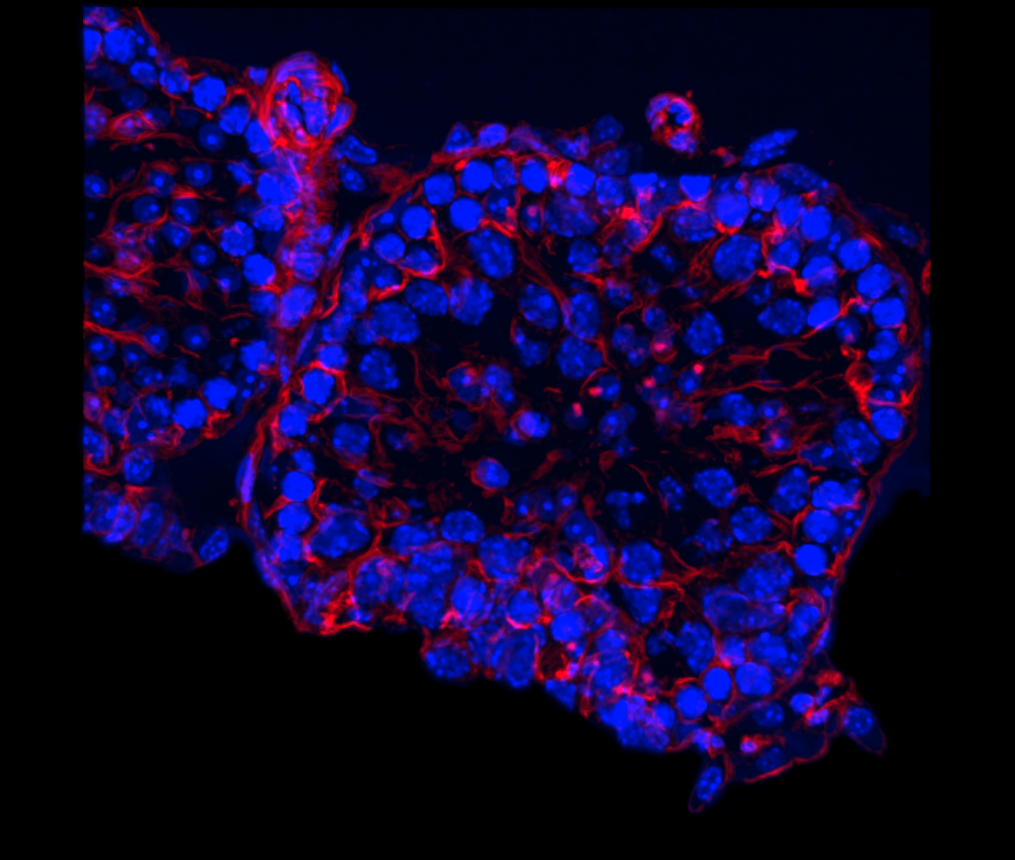

Supplement: Figure 5—figure supplement 1—source data 1. [file elife-83129-fig5-figsupp1-data1.zip › Figure supplement S5-source data 12/F-actin staining/hs-pd35-ko_1.tif]

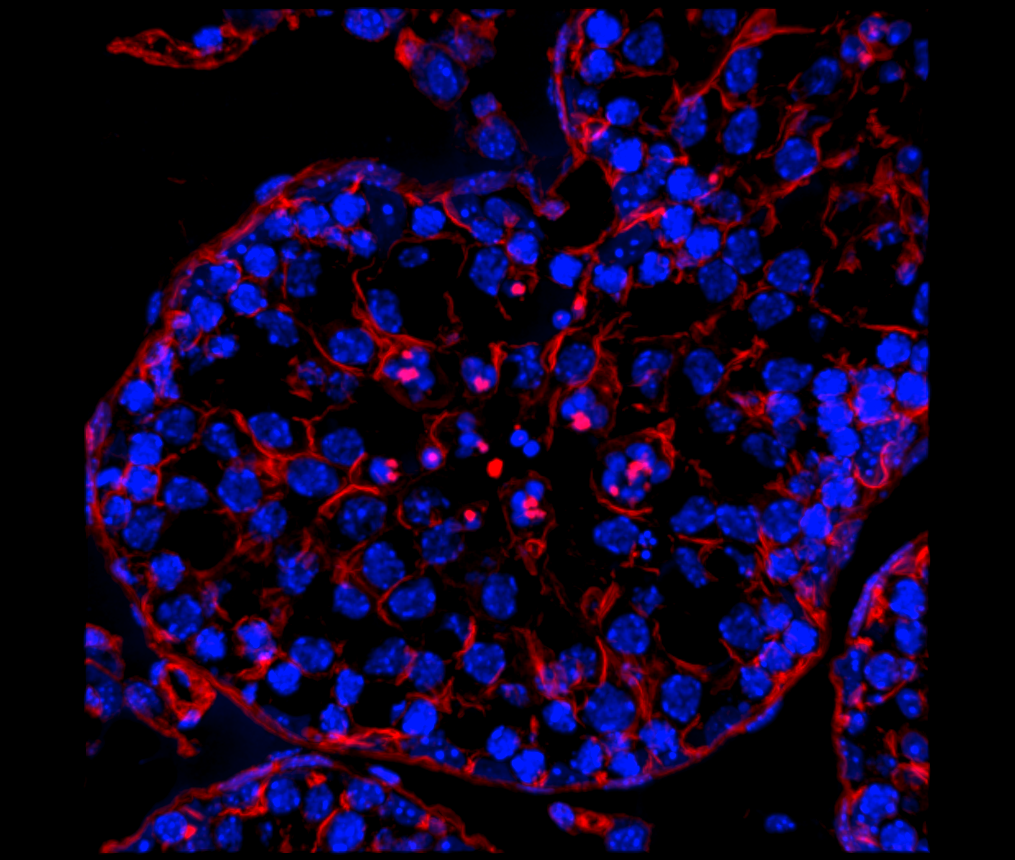

Supplement: Figure 5—figure supplement 1—source data 1. [file elife-83129-fig5-figsupp1-data1.zip › Figure supplement S5-source data 12/F-actin staining/hs-pd35-ko_2.tif]

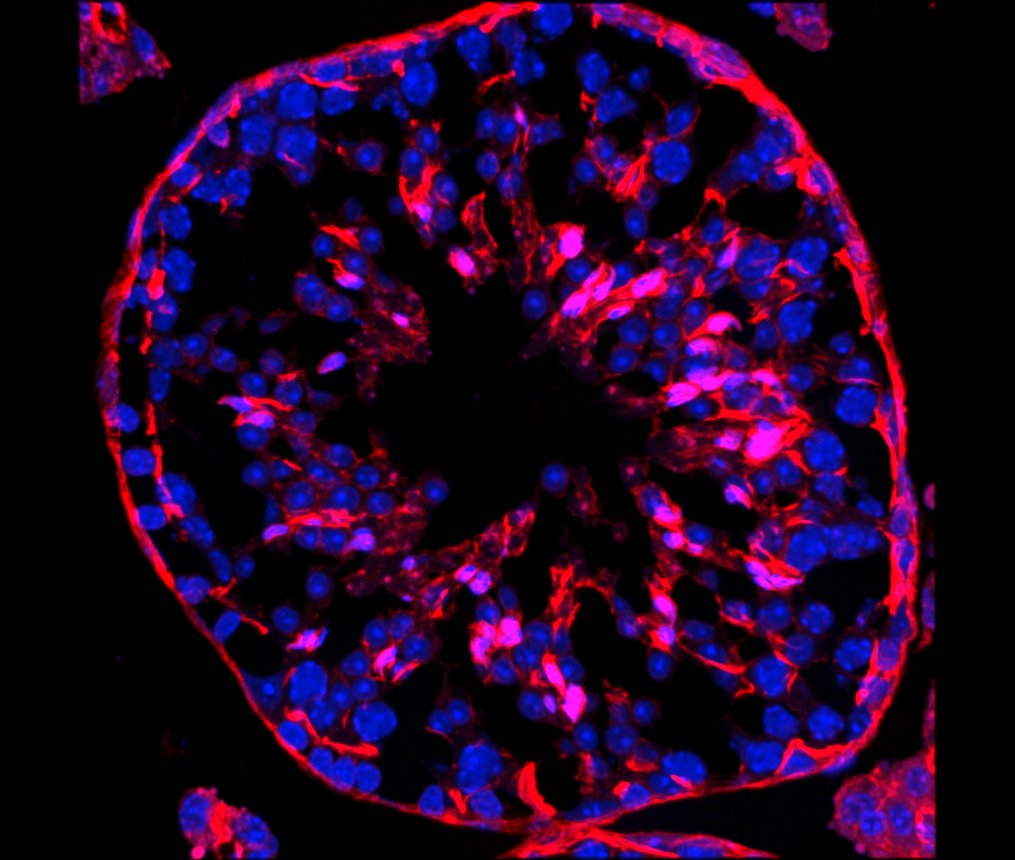

Supplement: Figure 5—figure supplement 1—source data 1. [file elife-83129-fig5-figsupp1-data1.zip › Figure supplement S5-source data 12/F-actin staining/hs-pd35-wt_1.tif]

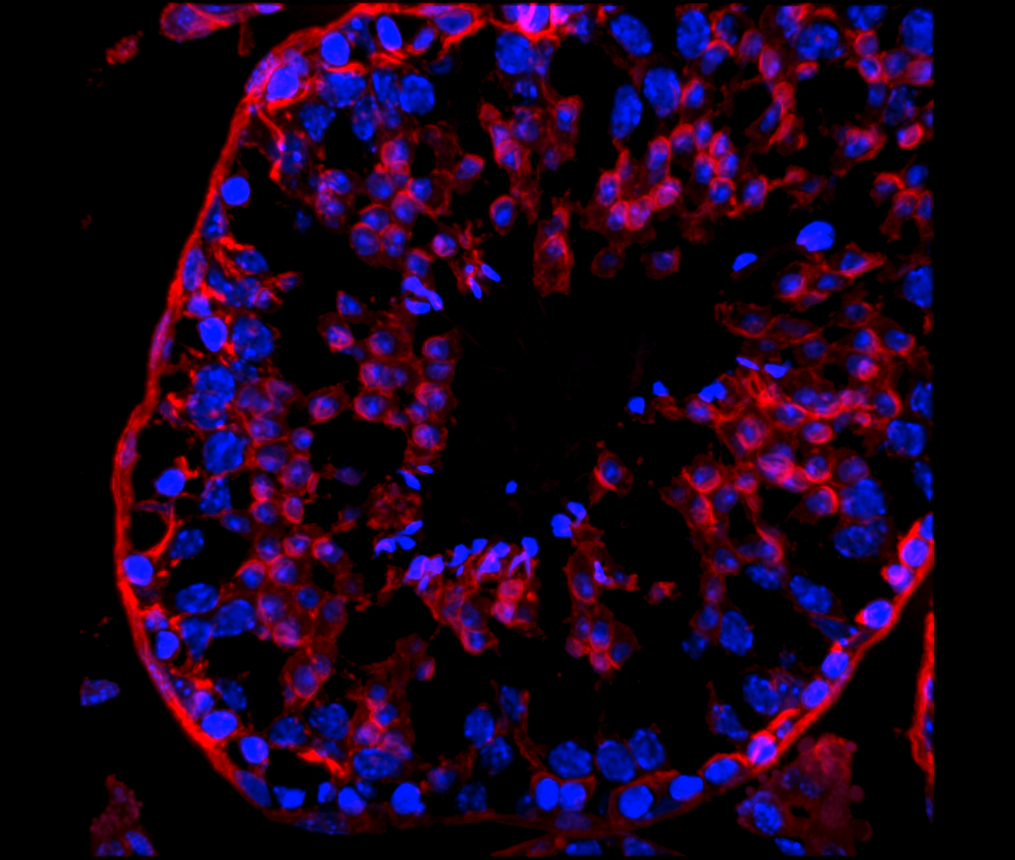

Supplement: Figure 5—figure supplement 1—source data 1. [file elife-83129-fig5-figsupp1-data1.zip › Figure supplement S5-source data 12/F-actin staining/hs-pd35-wt_2.tif]

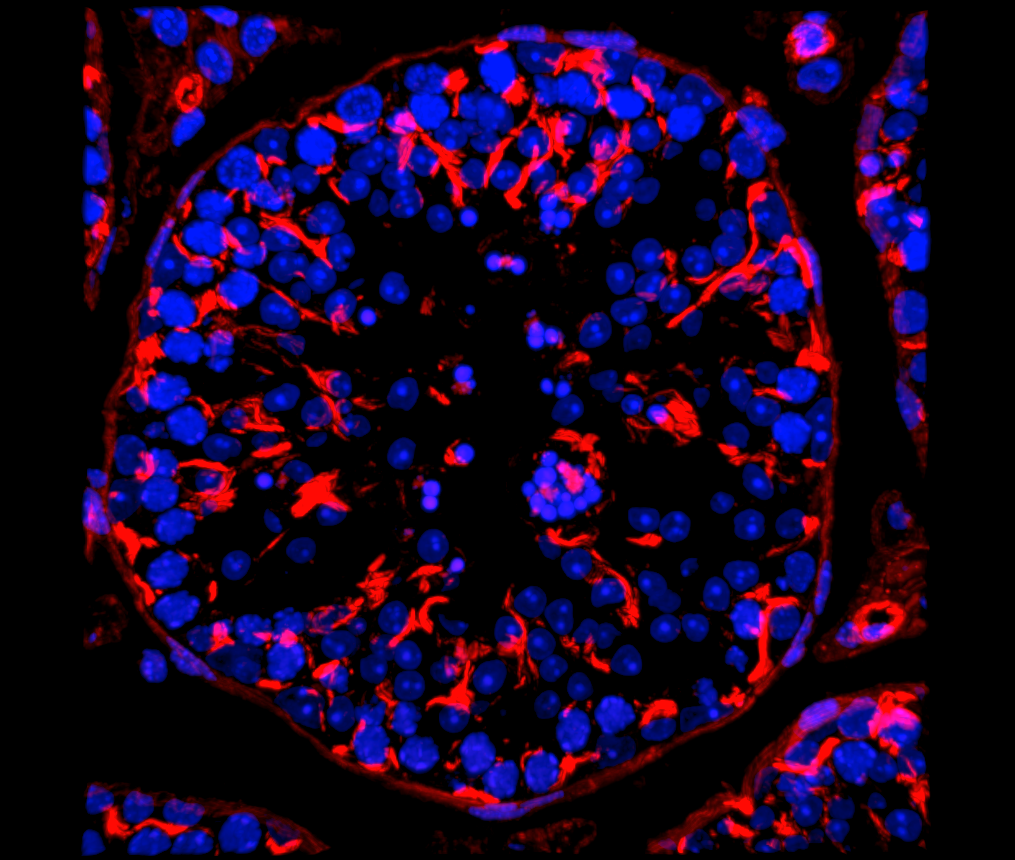

Supplement: Figure 5—figure supplement 1—source data 1. [file elife-83129-fig5-figsupp1-data1.zip › Figure supplement S5-source data 12/F-actin staining/hs-pd60-ko_1.tif]

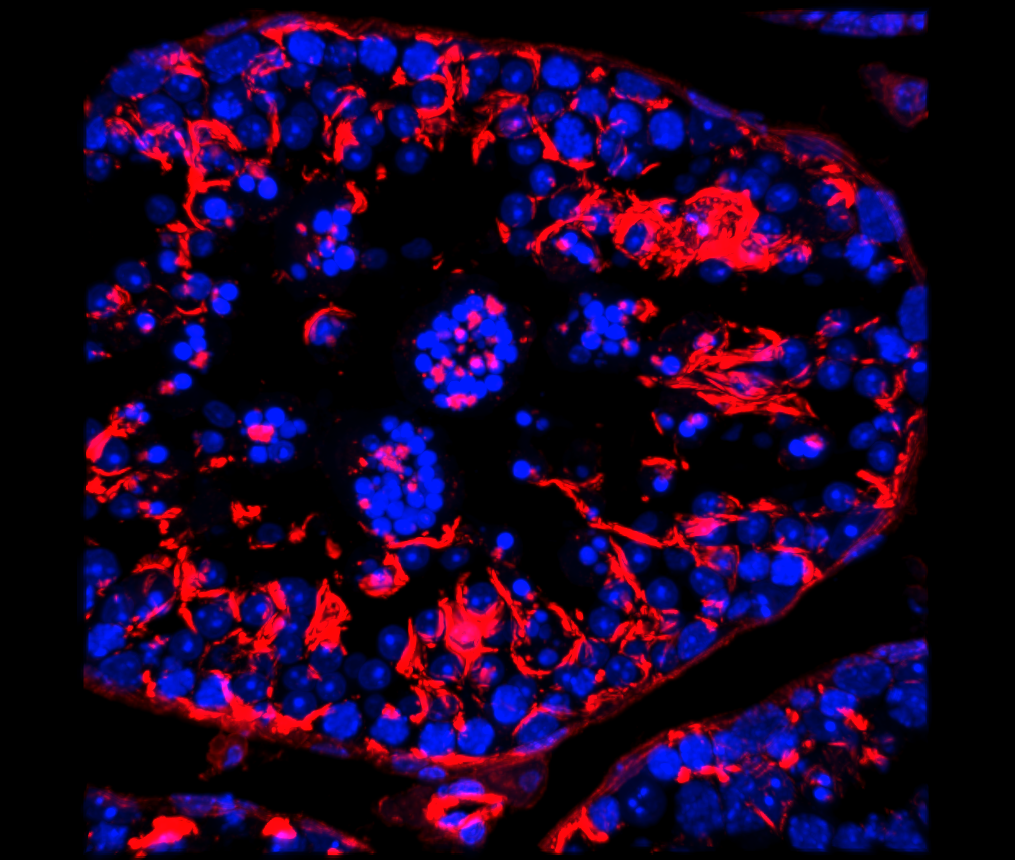

Supplement: Figure 5—figure supplement 1—source data 1. [file elife-83129-fig5-figsupp1-data1.zip › Figure supplement S5-source data 12/F-actin staining/hs-pd60-ko_2.tif]

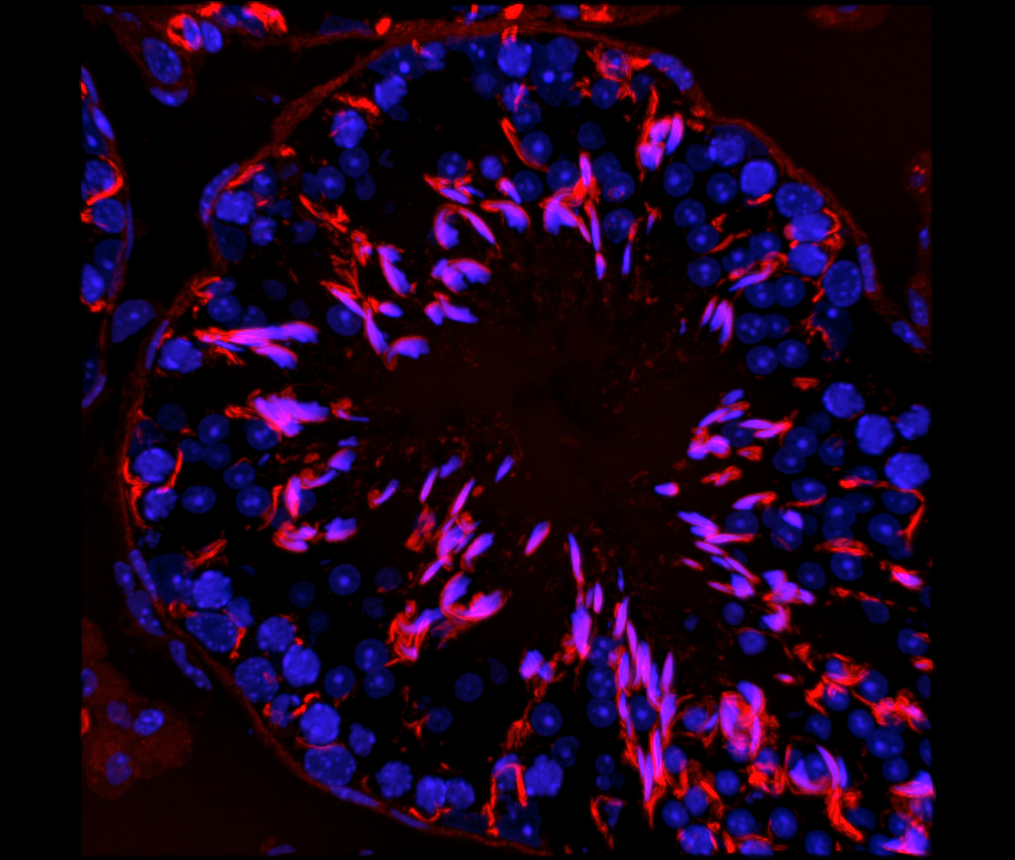

Supplement: Figure 5—figure supplement 1—source data 1. [file elife-83129-fig5-figsupp1-data1.zip › Figure supplement S5-source data 12/F-actin staining/hs-pd60-wt_1.tif]

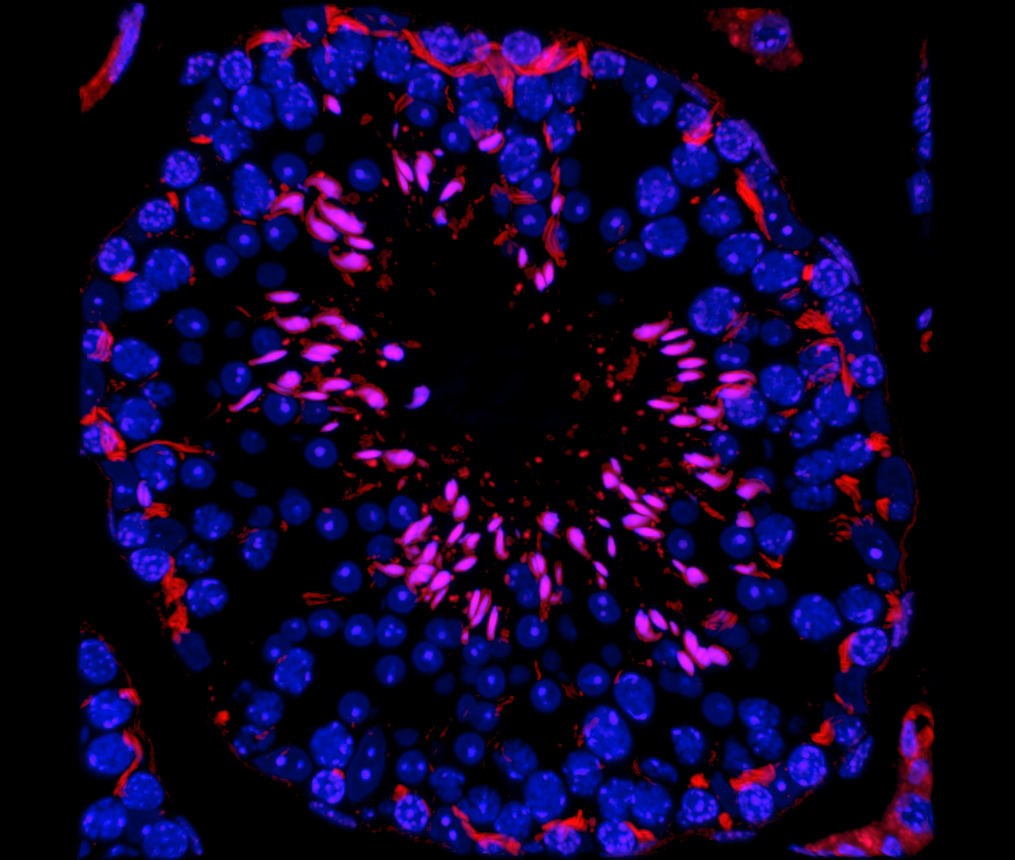

Supplement: Figure 5—figure supplement 1—source data 1. [file elife-83129-fig5-figsupp1-data1.zip › Figure supplement S5-source data 12/F-actin staining/hs-pd60-wt_2.tif]

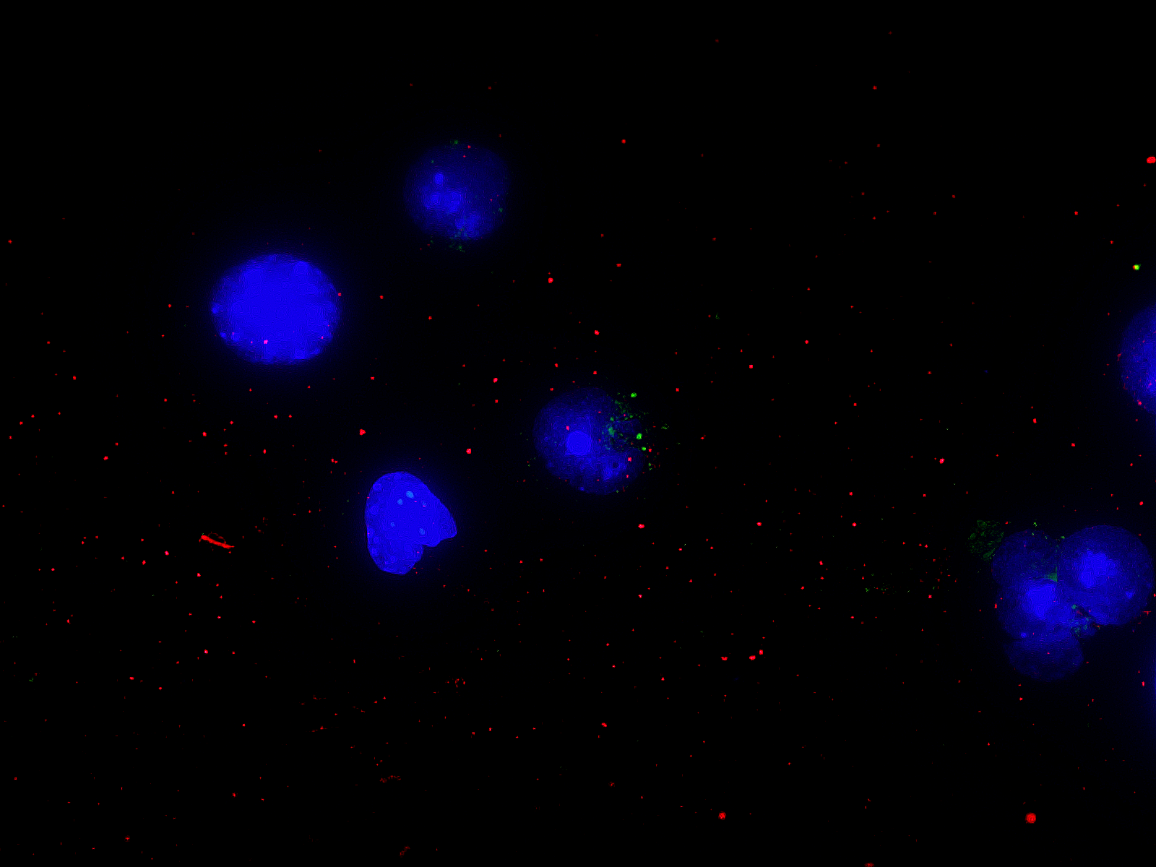

Supplement: Figure 6—source data 1. [file elife-83129-fig6-data1.zip › Figure6/Source data of Figure6A/GOPC/KO/GOPC-1.tif]

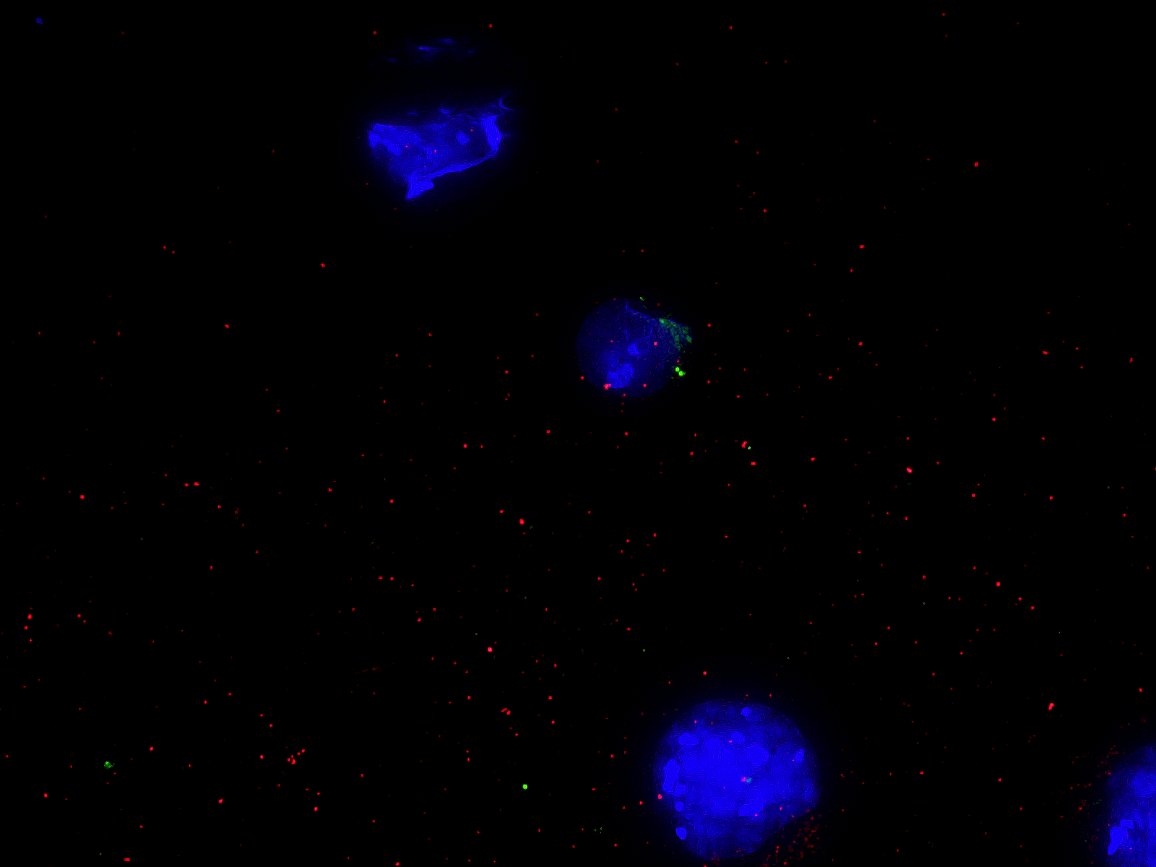

Supplement: Figure 6—source data 1. [file elife-83129-fig6-data1.zip › Figure6/Source data of Figure6A/GOPC/KO/GOPC-2.tif]

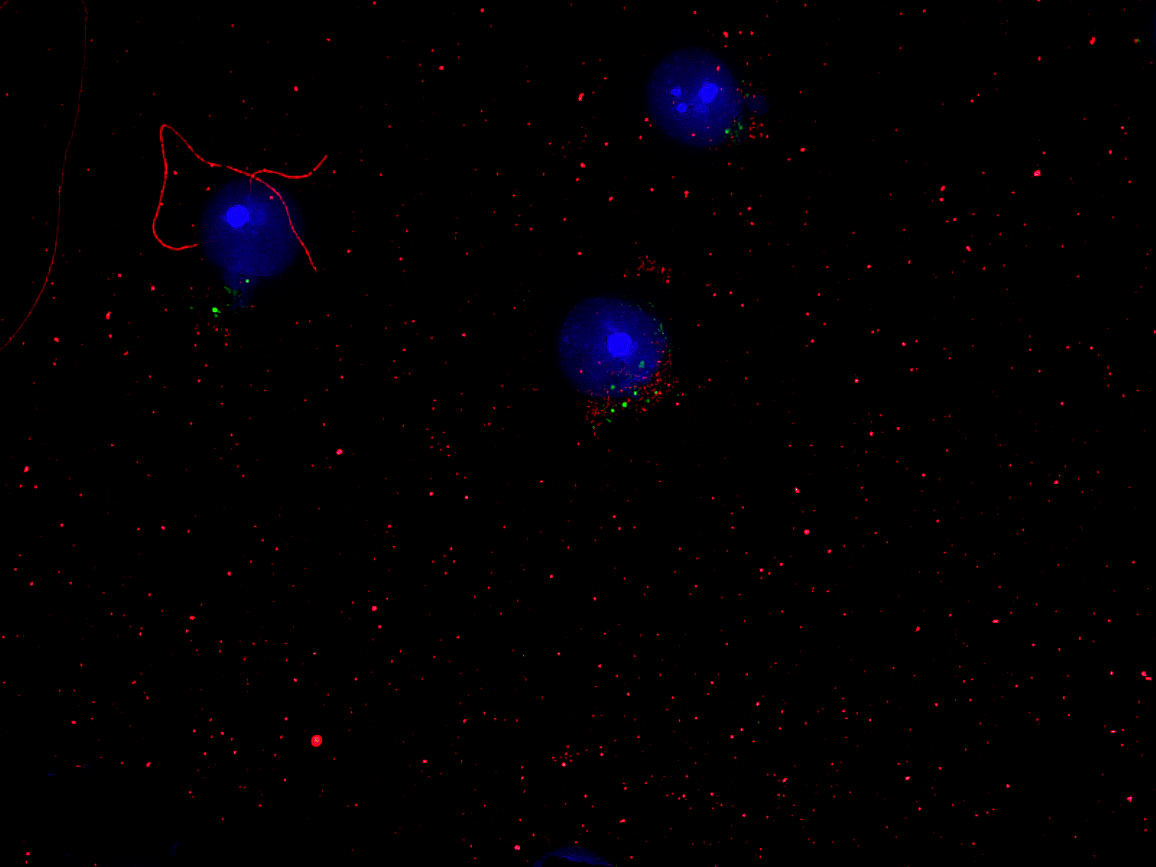

Supplement: Figure 6—source data 1. [file elife-83129-fig6-data1.zip › Figure6/Source data of Figure6A/GOPC/KO/GOPC-3.tif]

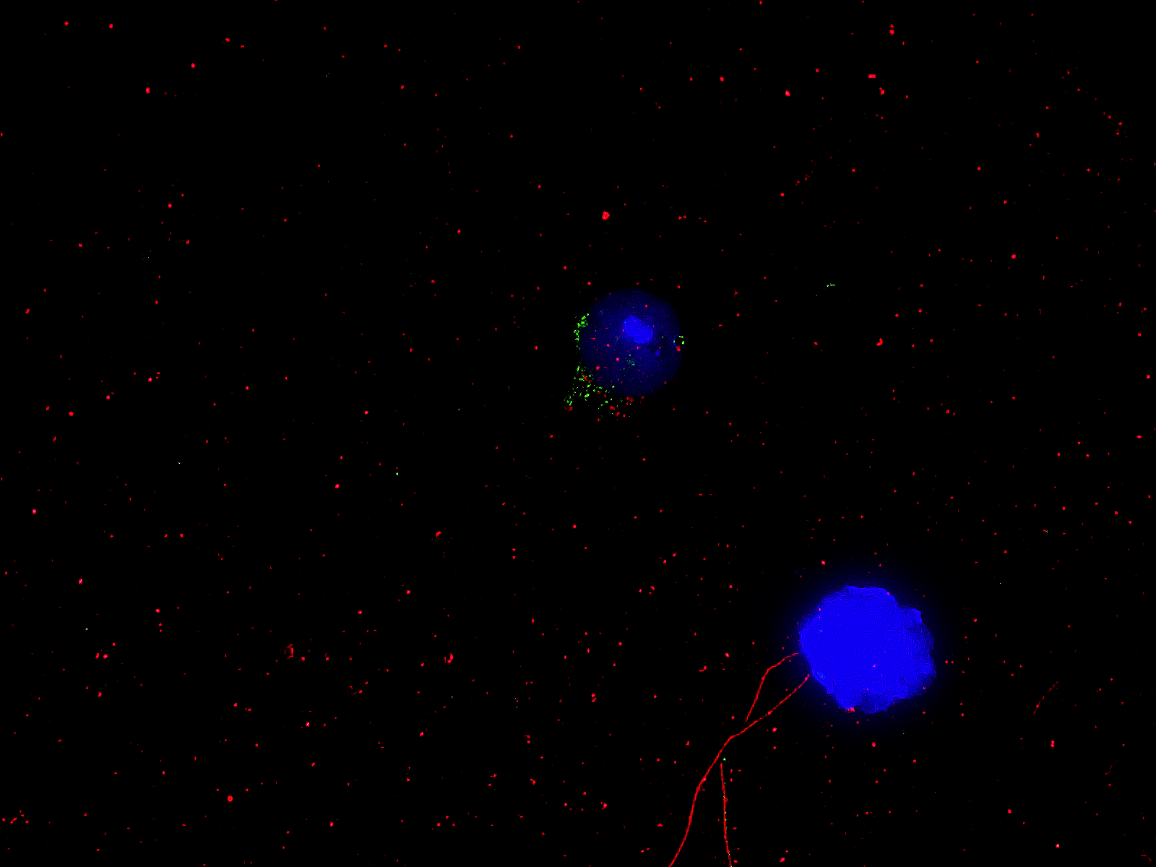

Supplement: Figure 6—source data 1. [file elife-83129-fig6-data1.zip › Figure6/Source data of Figure6A/GOPC/KO/GOPC-4.tif]

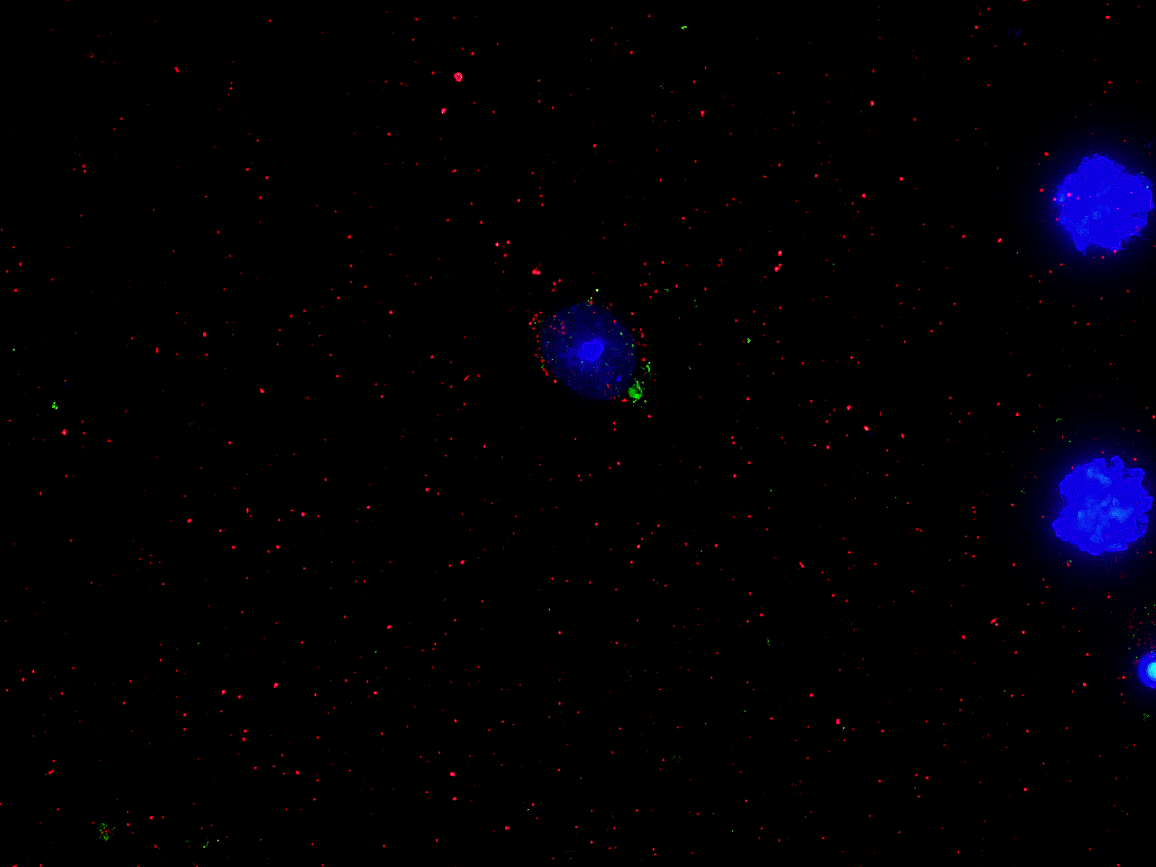

Supplement: Figure 6—source data 1. [file elife-83129-fig6-data1.zip › Figure6/Source data of Figure6A/GOPC/KO/GOPC-5.tif]

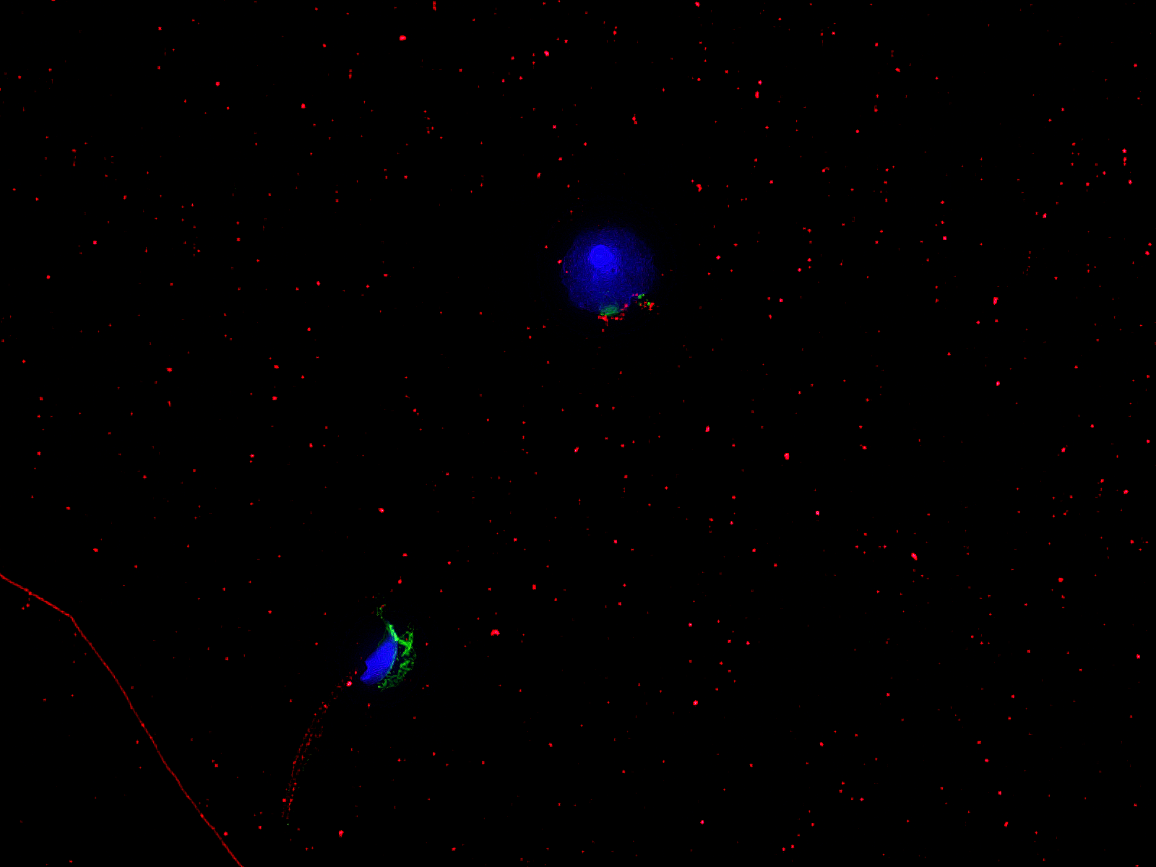

Supplement: Figure 6—source data 1. [file elife-83129-fig6-data1.zip › Figure6/Source data of Figure6A/GOPC/WT/GOPC-1.tif]

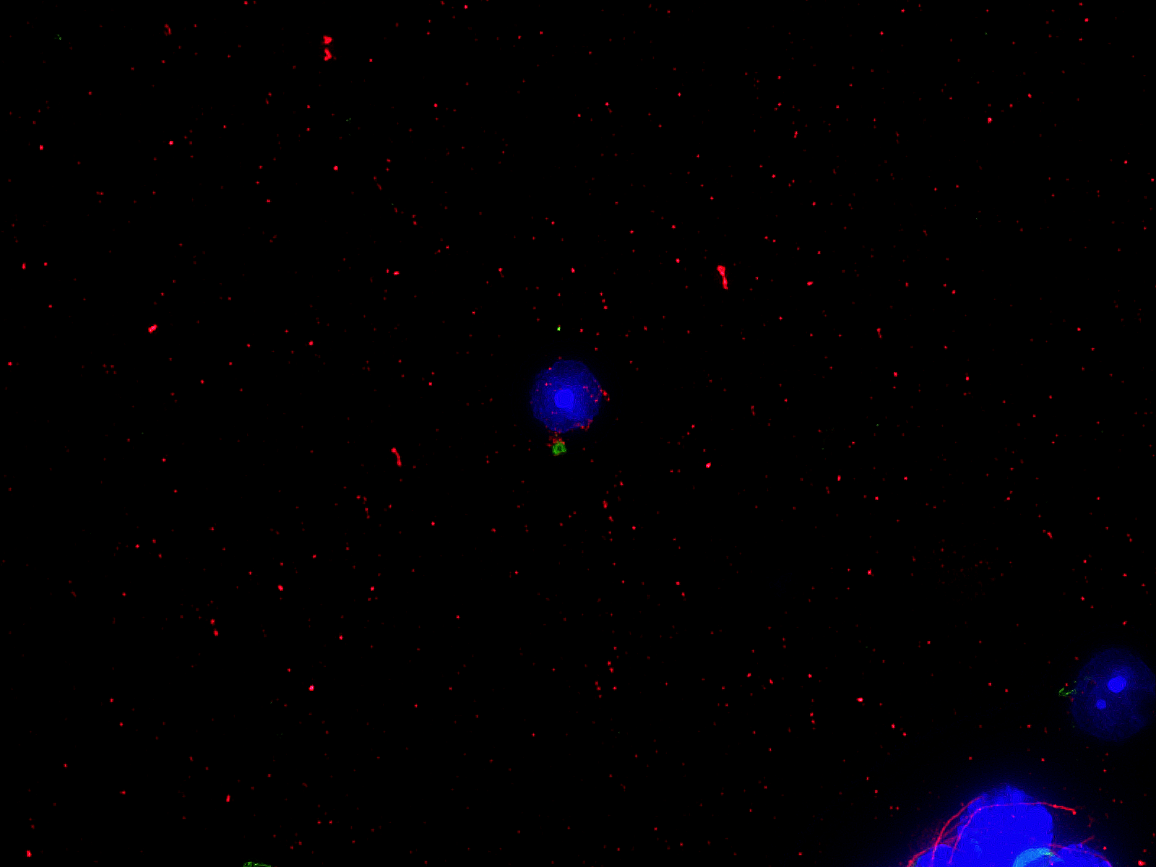

Supplement: Figure 6—source data 1. [file elife-83129-fig6-data1.zip › Figure6/Source data of Figure6A/GOPC/WT/GOPC-2.tif]

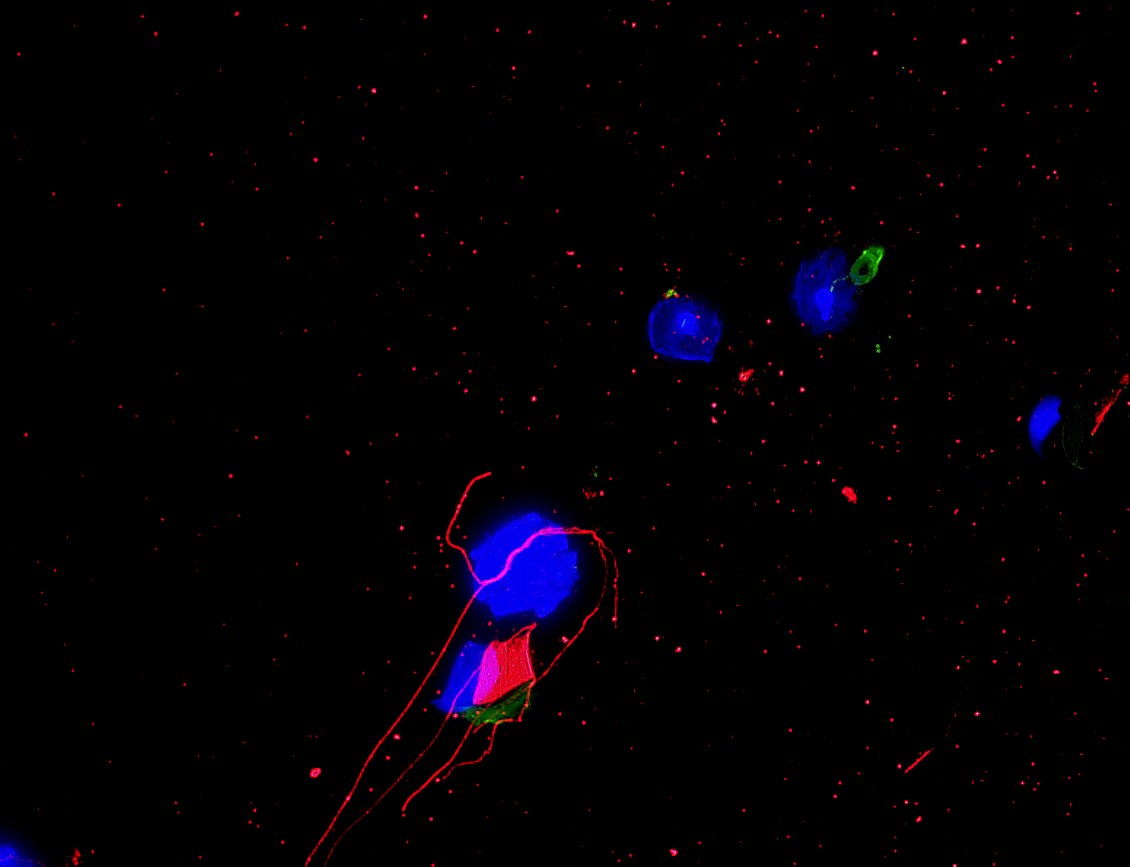

Supplement: Figure 6—source data 1. [file elife-83129-fig6-data1.zip › Figure6/Source data of Figure6A/GOPC/WT/GOPC-3.tif]

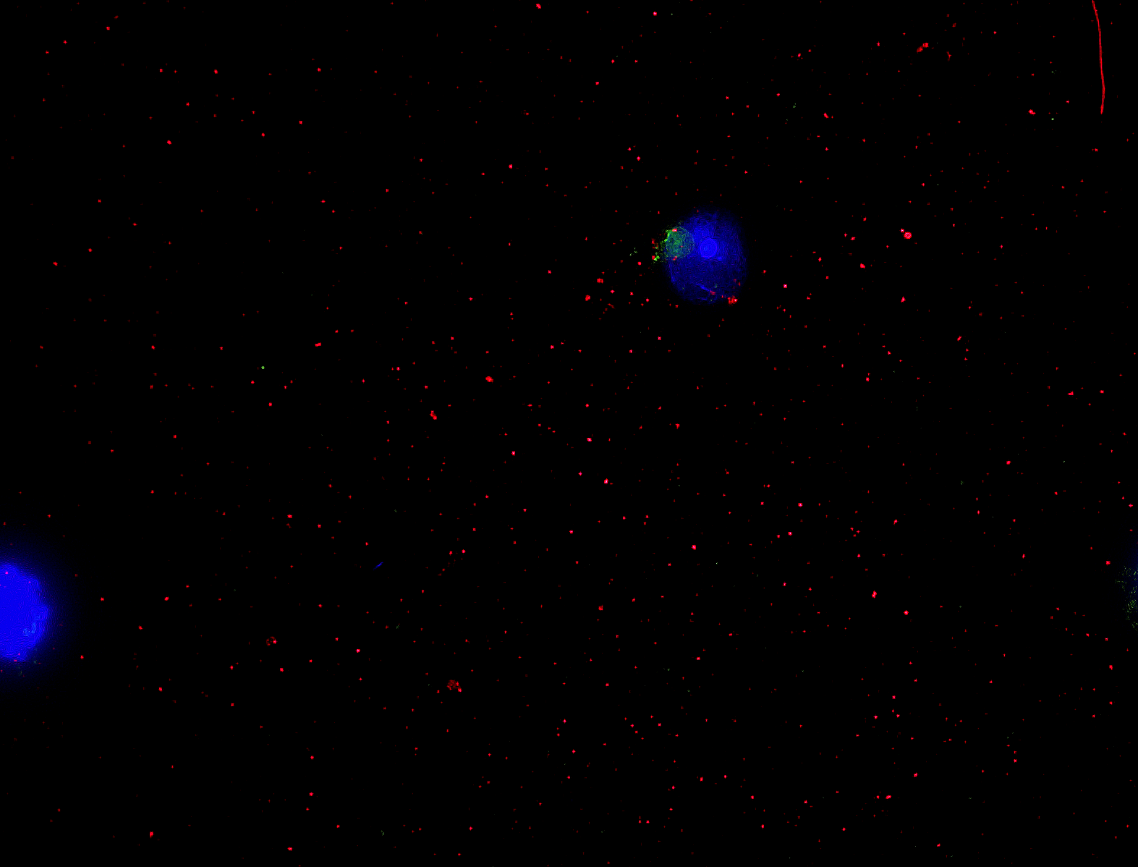

Supplement: Figure 6—source data 1. [file elife-83129-fig6-data1.zip › Figure6/Source data of Figure6A/GOPC/WT/GOPC-4.tif]

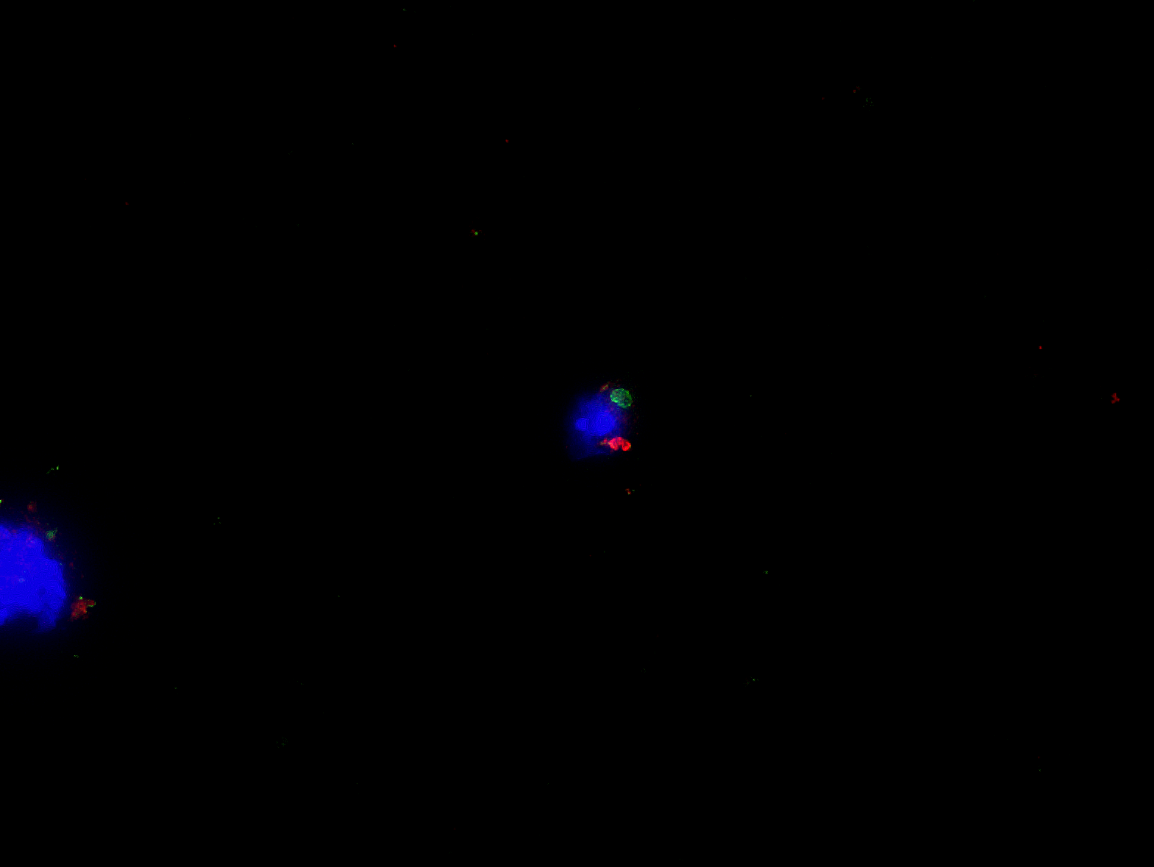

Supplement: Figure 6—source data 1. [file elife-83129-fig6-data1.zip › Figure6/Source data of Figure6C/LC3/KO/LC3AB-1.tif]

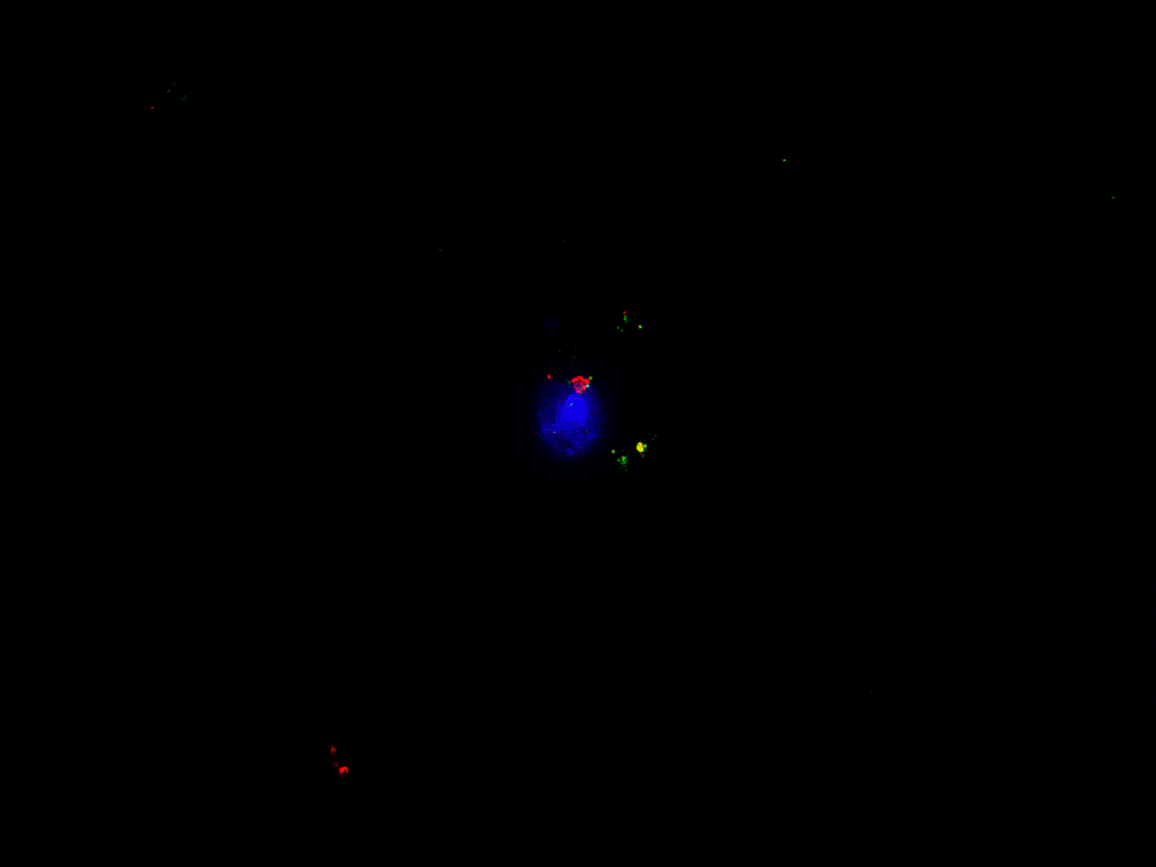

Supplement: Figure 6—source data 1. [file elife-83129-fig6-data1.zip › Figure6/Source data of Figure6C/LC3/KO/LC3AB-2.tif]

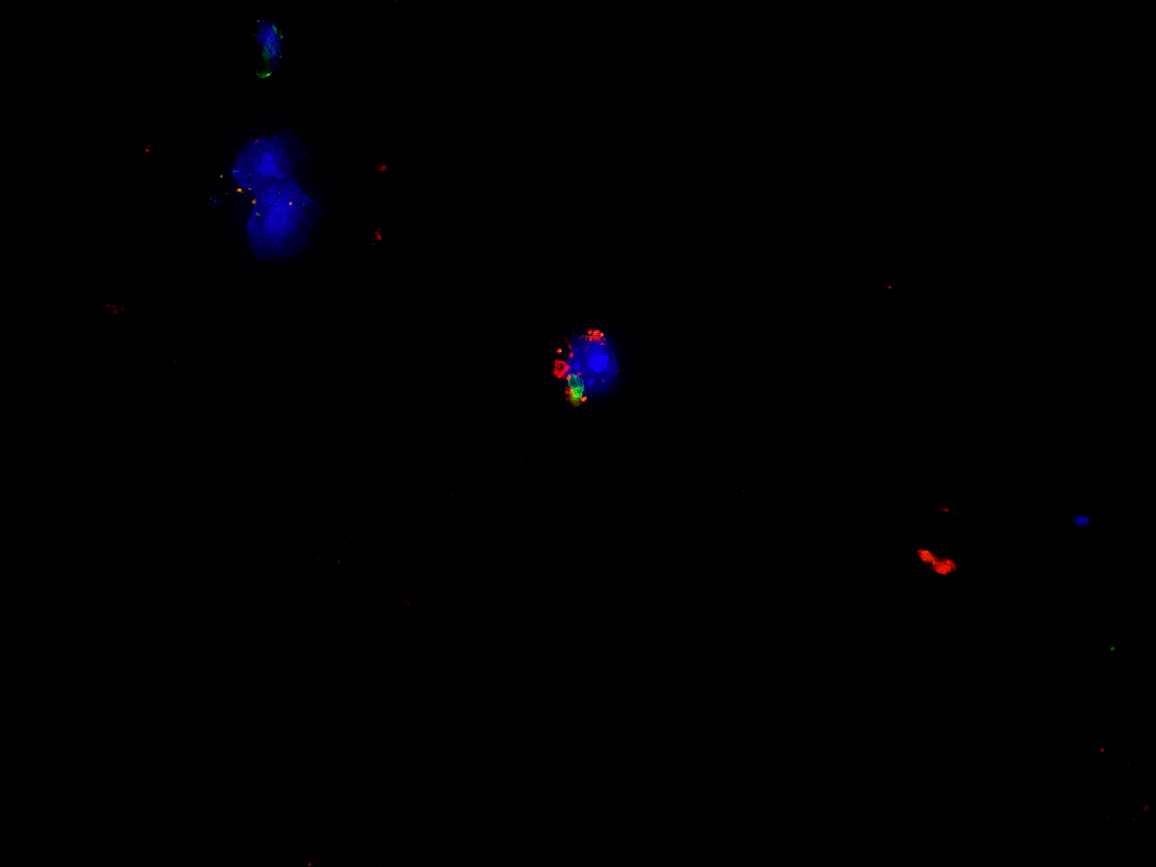

Supplement: Figure 6—source data 1. [file elife-83129-fig6-data1.zip › Figure6/Source data of Figure6C/LC3/KO/LC3AB-3.tif]

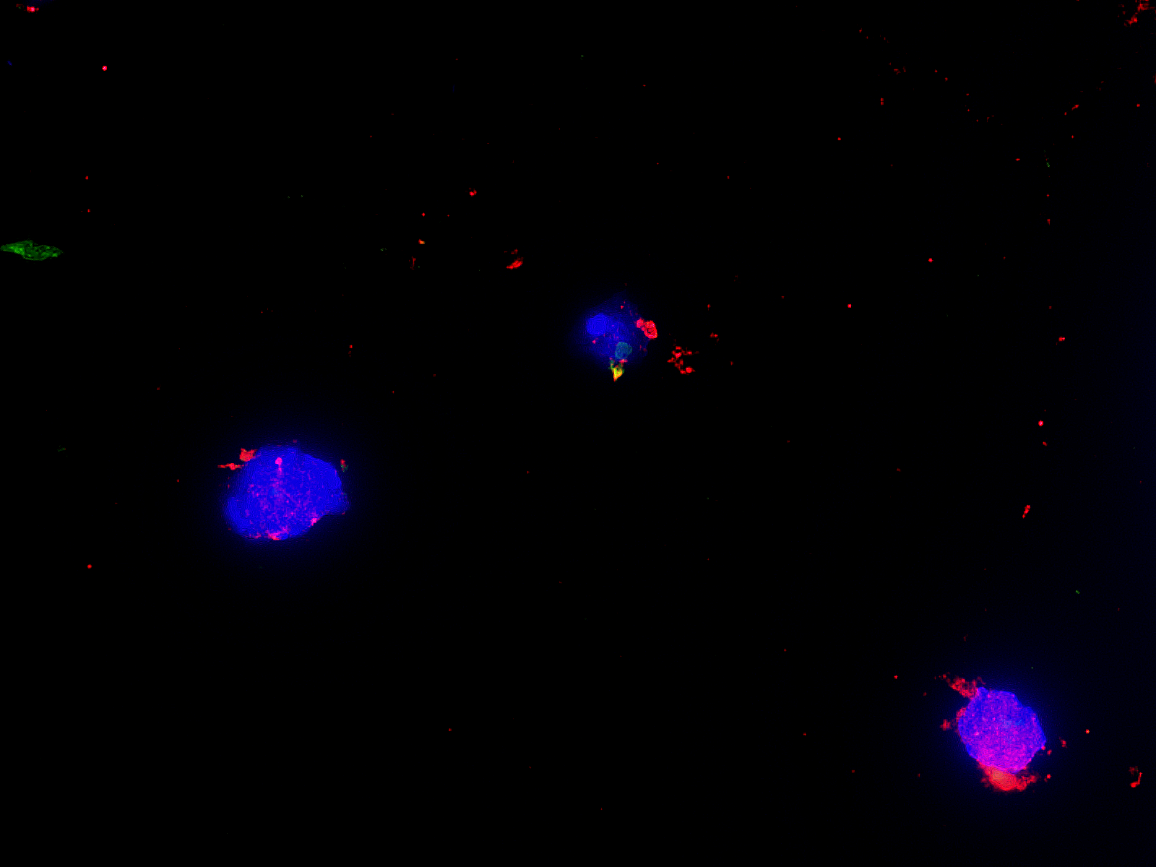

Supplement: Figure 6—source data 1. [file elife-83129-fig6-data1.zip › Figure6/Source data of Figure6C/LC3/KO/LC3AB-4.tif]

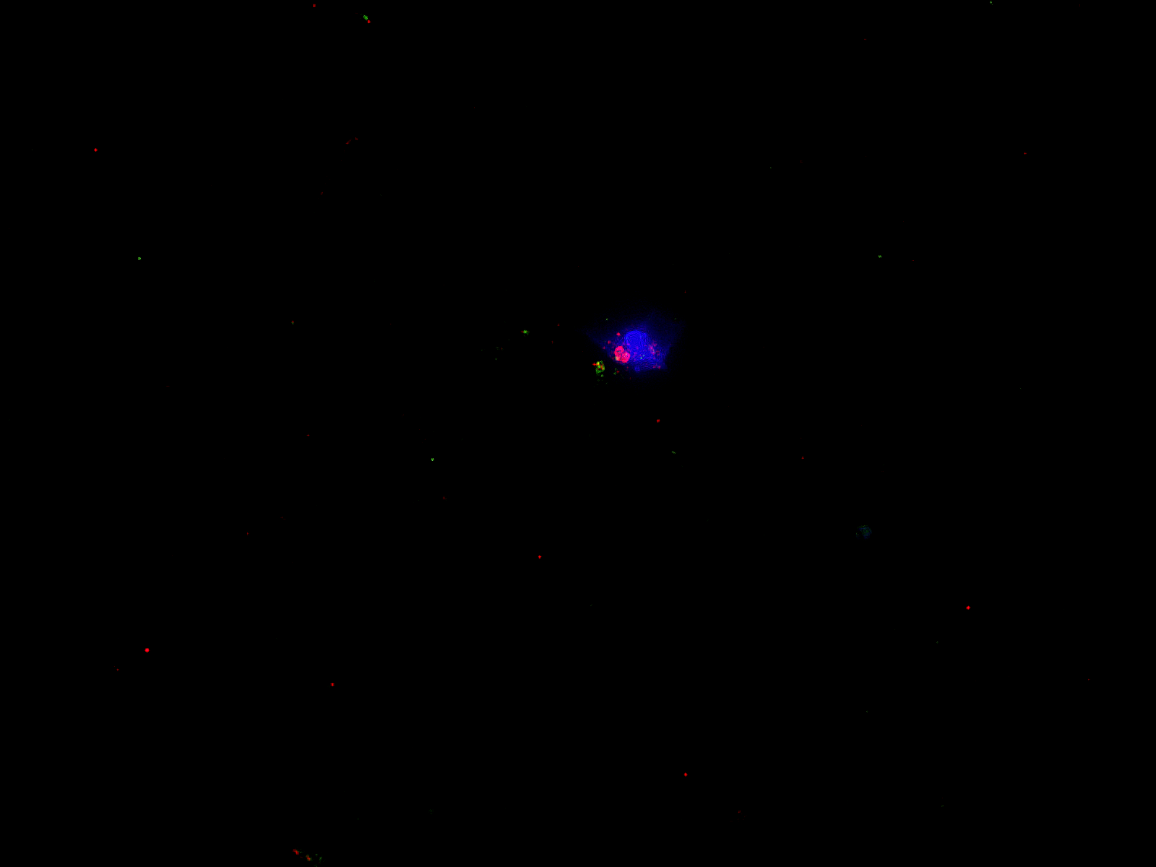

Supplement: Figure 6—source data 1. [file elife-83129-fig6-data1.zip › Figure6/Source data of Figure6C/LC3/KO/LC3AB-5.tif]

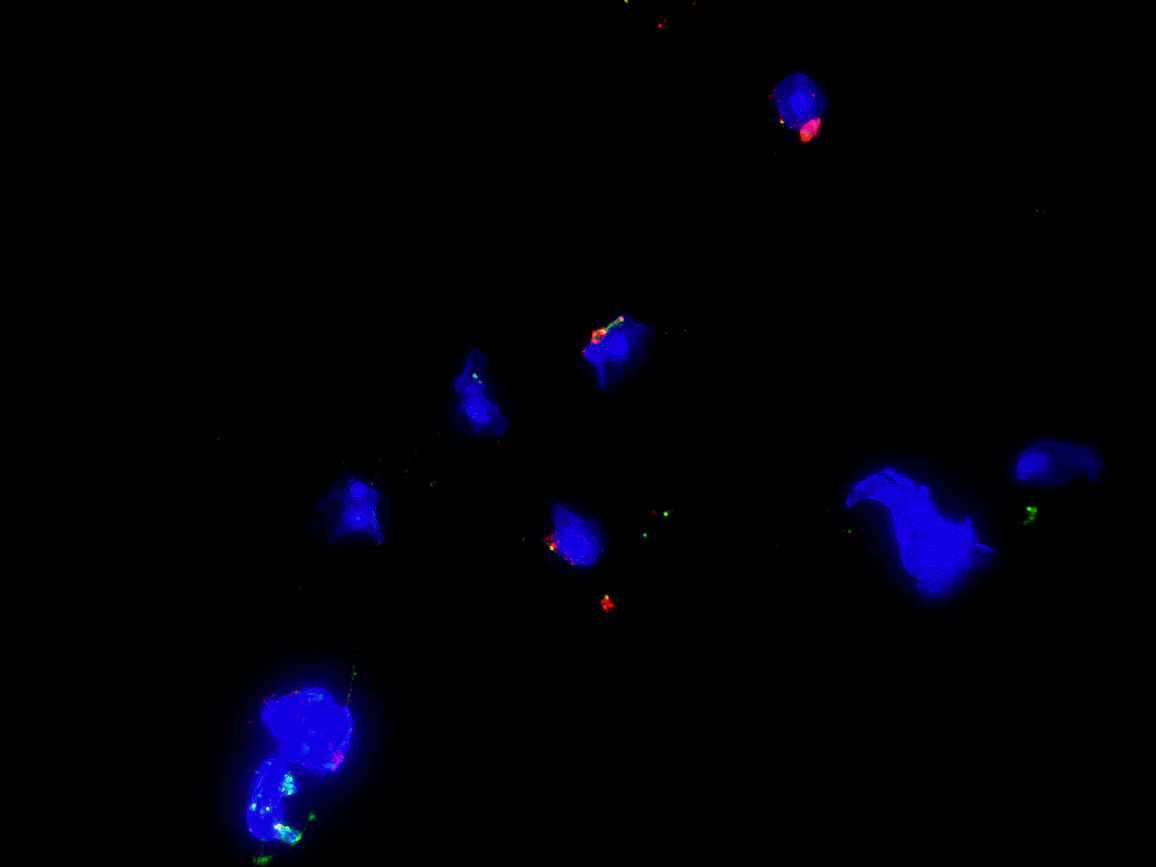

Supplement: Figure 6—source data 1. [file elife-83129-fig6-data1.zip › Figure6/Source data of Figure6C/LC3/KO/LC3AB-6.tif]

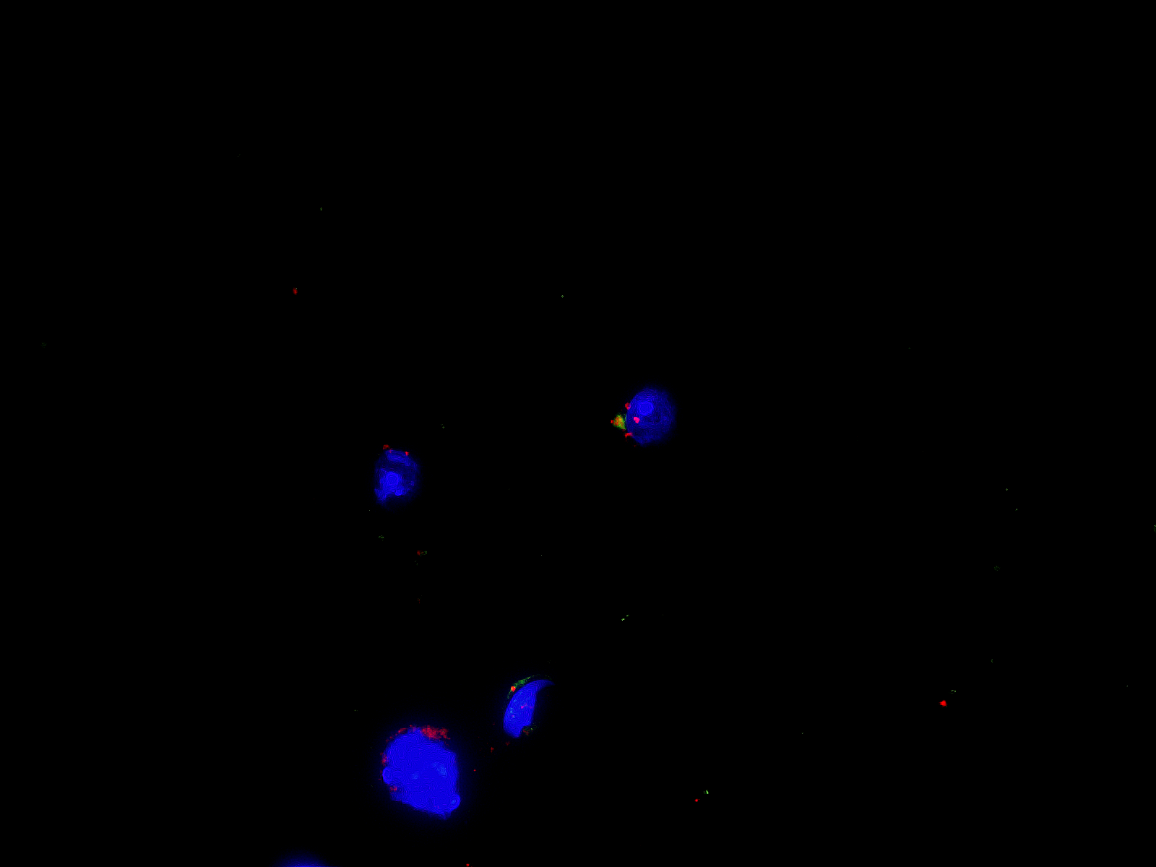

Supplement: Figure 6—source data 1. [file elife-83129-fig6-data1.zip › Figure6/Source data of Figure6C/LC3/WT/LC3AB-1.tif]

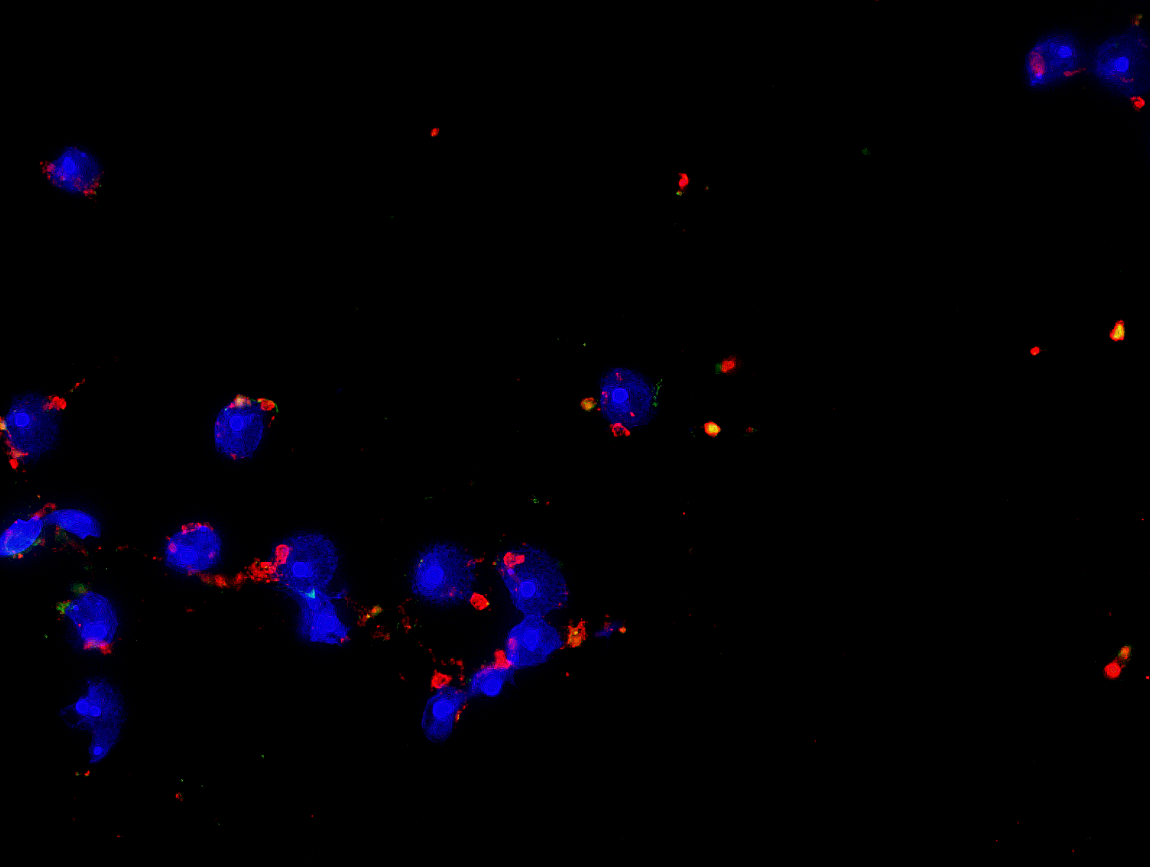

Supplement: Figure 6—source data 1. [file elife-83129-fig6-data1.zip › Figure6/Source data of Figure6C/LC3/WT/LC3AB-2.tif]

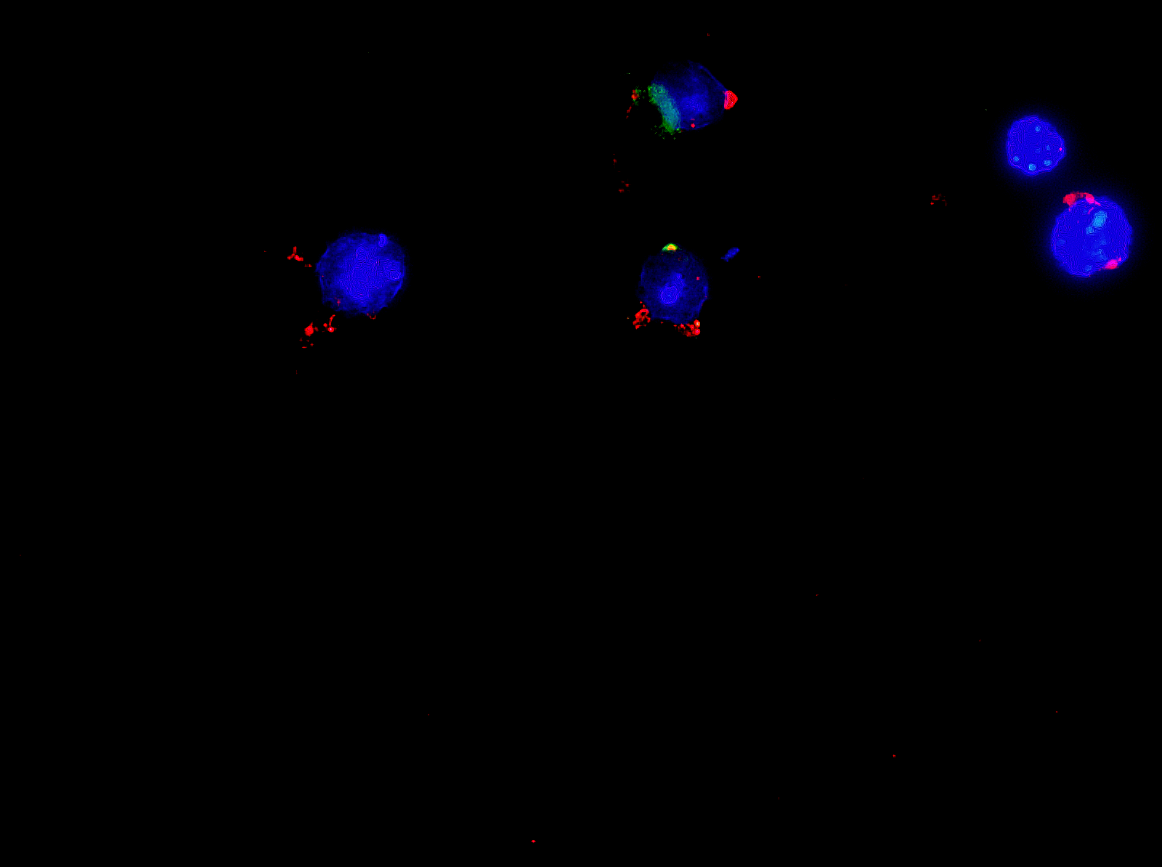

Supplement: Figure 6—source data 1. [file elife-83129-fig6-data1.zip › Figure6/Source data of Figure6C/LC3/WT/LC3AB-3.tif]
