# Supplementary figures and images for: The Slingshot phosphatase 2 is required for acrosome biogenesis during spermatogenesis in mice (part 4 of 4)
Source: eLife. 2023 Mar 21;12:e83129. doi: 10.7554/eLife.83129 (PMC10065795; doi:10.7554/eLife.83129)

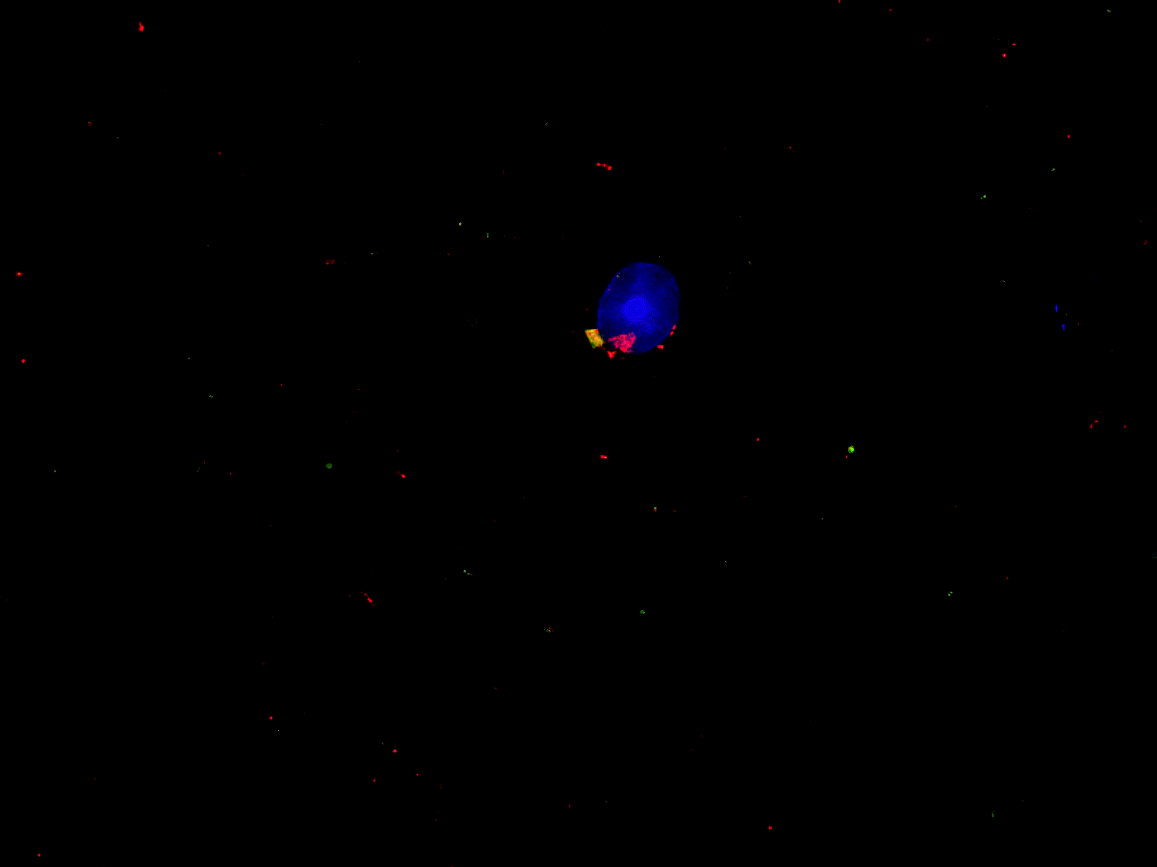

Supplement: Figure 6—source data 1. [file elife-83129-fig6-data1.zip › Figure6/Source data of Figure6C/LC3/WT/LC3AB-4.tif]

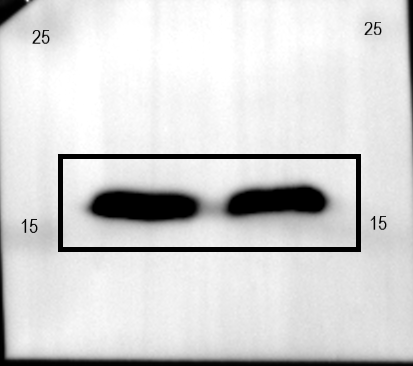

Supplement: Figure 7—source data 1. [file elife-83129-fig7-data1.zip › Figure7/Source data of Figure7A/Labelled blots of COFILIN.Tif]

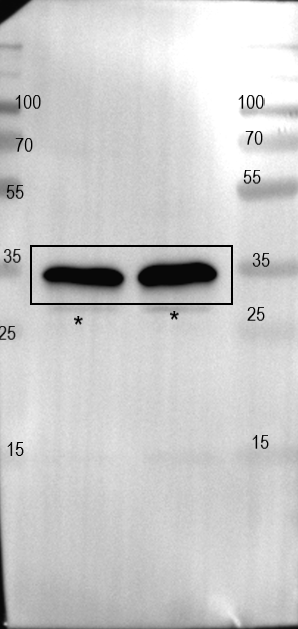

Supplement: Figure 7—source data 1. [file elife-83129-fig7-data1.zip › Figure7/Source data of Figure7A/Labelled blots of GAPDH-1.tif]

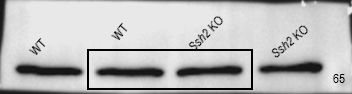

Supplement: Figure 7—source data 1. [file elife-83129-fig7-data1.zip › Figure7/Source data of Figure7A/Labelled blots of LIMK1.tif]

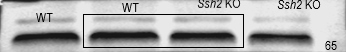

Supplement: Figure 7—source data 1. [file elife-83129-fig7-data1.zip › Figure7/Source data of Figure7A/Labelled blots of LIMK2.tif]

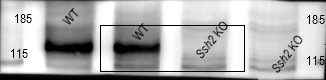

Supplement: Figure 7—source data 1. [file elife-83129-fig7-data1.zip › Figure7/Source data of Figure7A/Labelled blots of SSH2.tif]

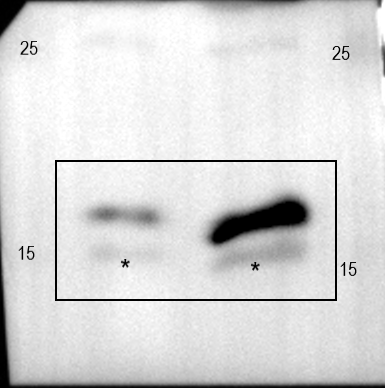

Supplement: Figure 7—source data 1. [file elife-83129-fig7-data1.zip › Figure7/Source data of Figure7A/Labelled blots of p-COFILIN-1.tiff]

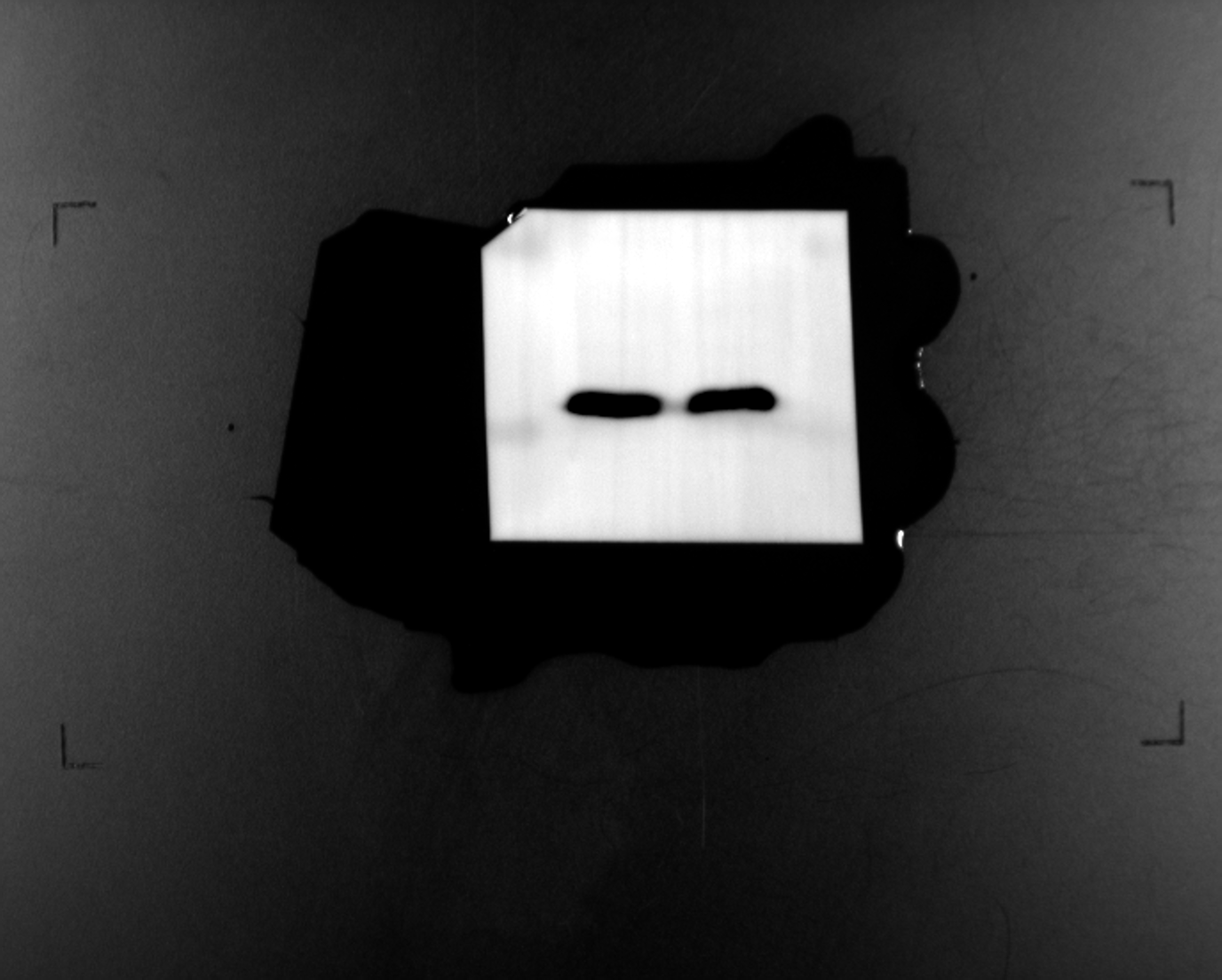

Supplement: Figure 7—source data 1. [file elife-83129-fig7-data1.zip › Figure7/Source data of Figure7A/Raw blots of COFILIN.Tif]

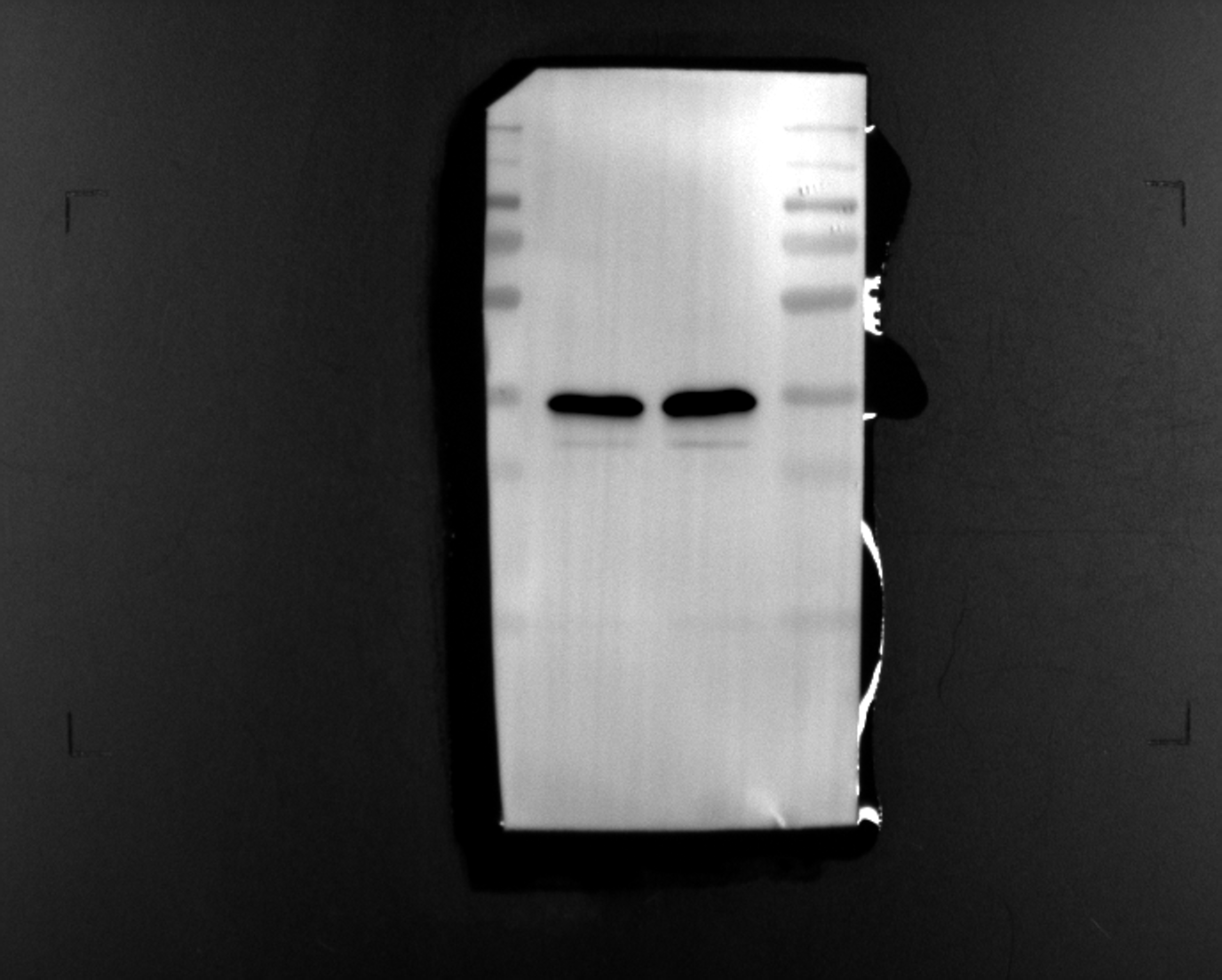

Supplement: Figure 7—source data 1. [file elife-83129-fig7-data1.zip › Figure7/Source data of Figure7A/Raw blots of GAPDH-1.Tif]

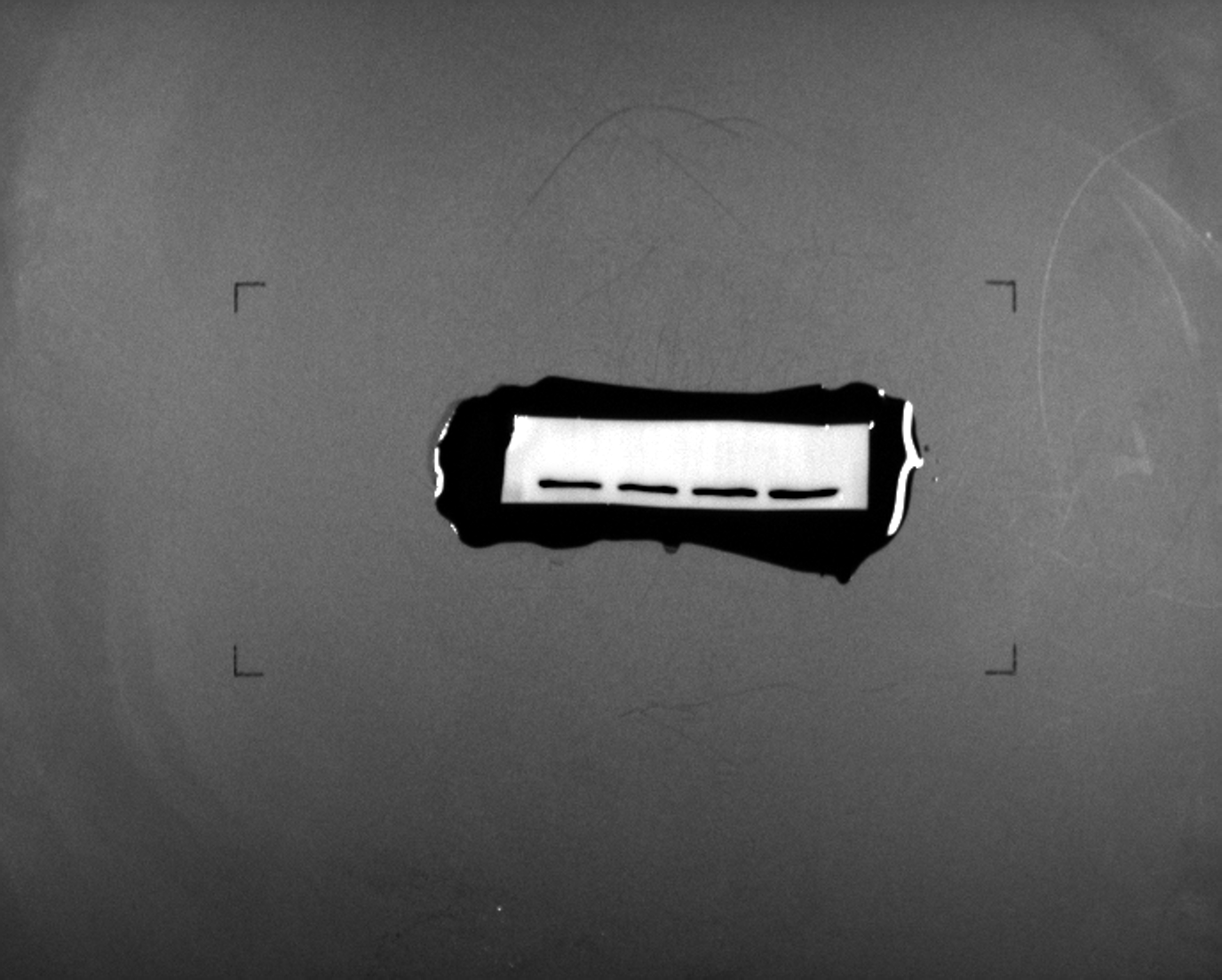

Supplement: Figure 7—source data 1. [file elife-83129-fig7-data1.zip › Figure7/Source data of Figure7A/Raw blots of LIMK1.Tif]

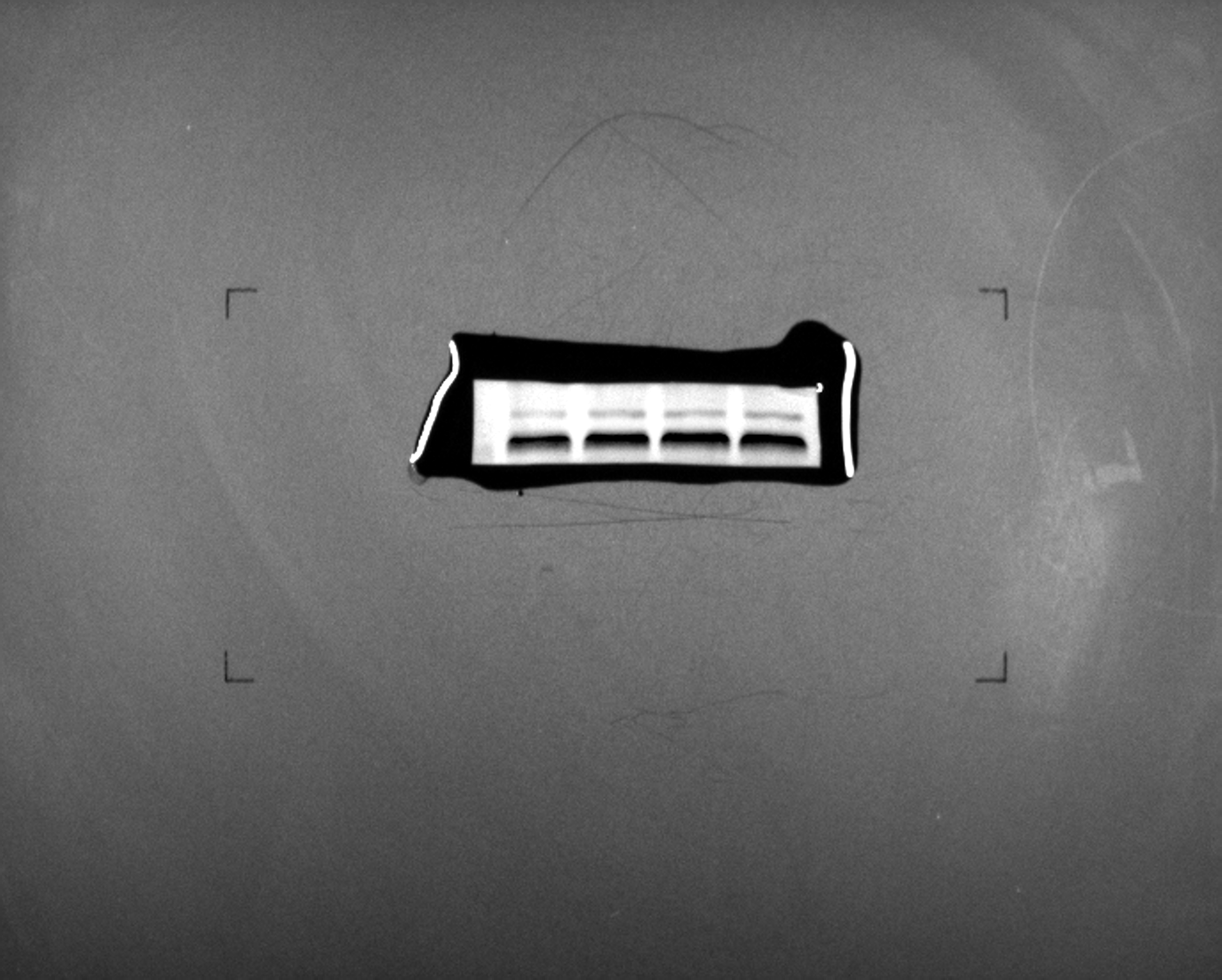

Supplement: Figure 7—source data 1. [file elife-83129-fig7-data1.zip › Figure7/Source data of Figure7A/Raw blots of LIMK2.Tif]

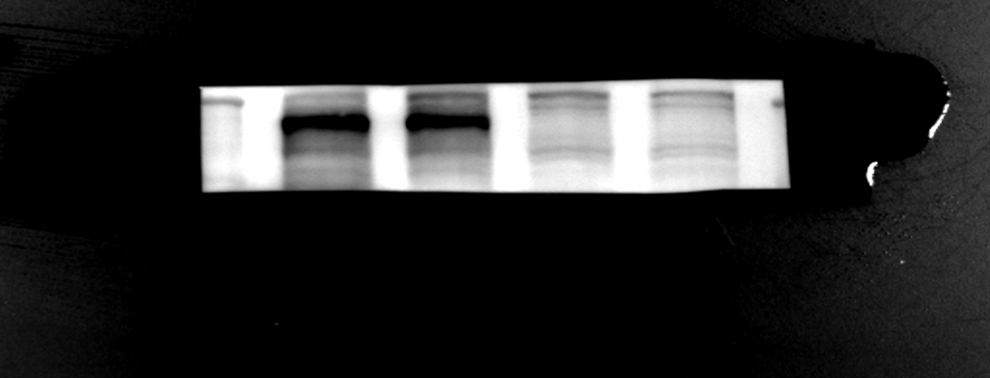

Supplement: Figure 7—source data 1. [file elife-83129-fig7-data1.zip › Figure7/Source data of Figure7A/Raw blots of SSH2-1.tif]

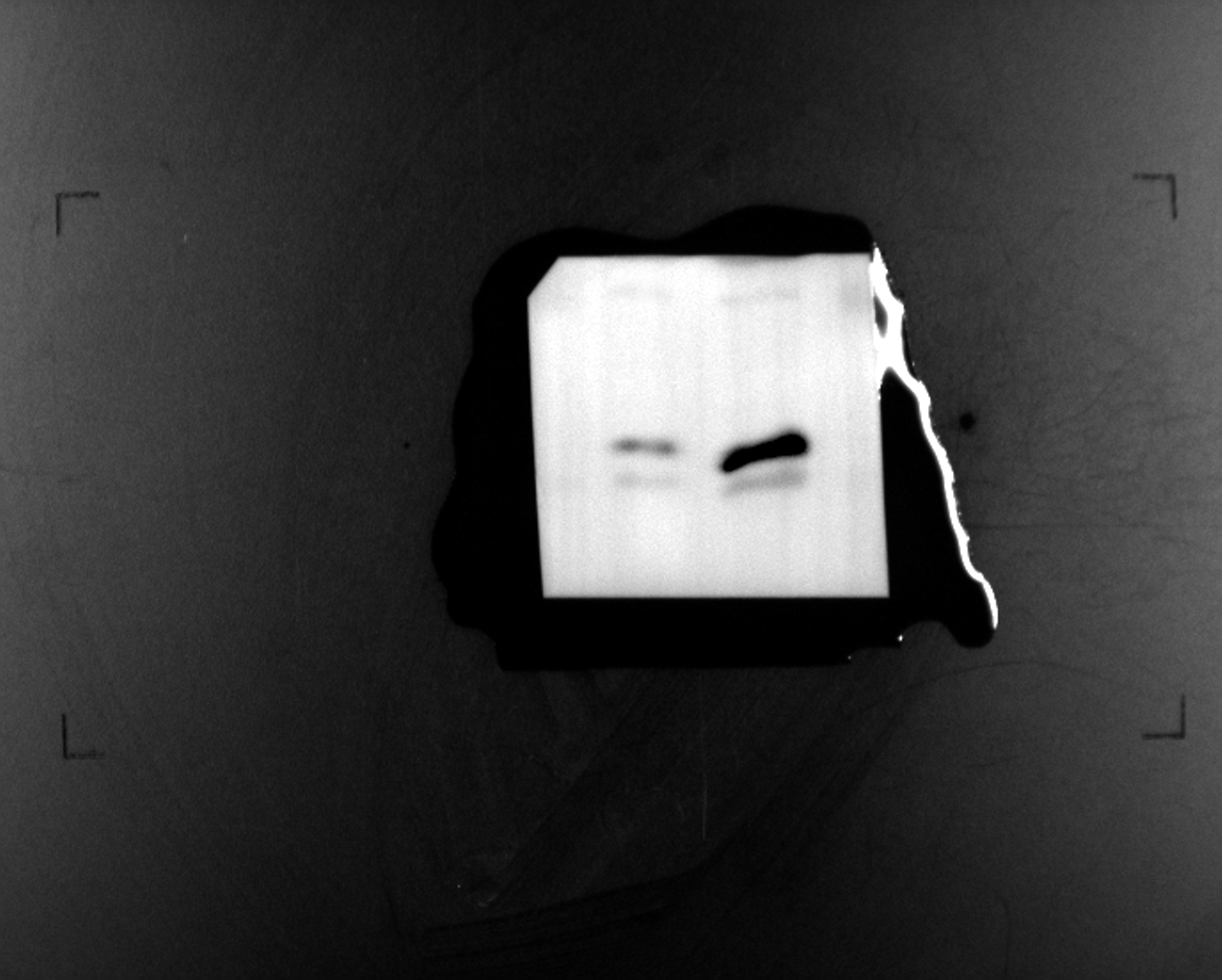

Supplement: Figure 7—source data 1. [file elife-83129-fig7-data1.zip › Figure7/Source data of Figure7A/Raw blots of p-COFILIN-1.Tif]

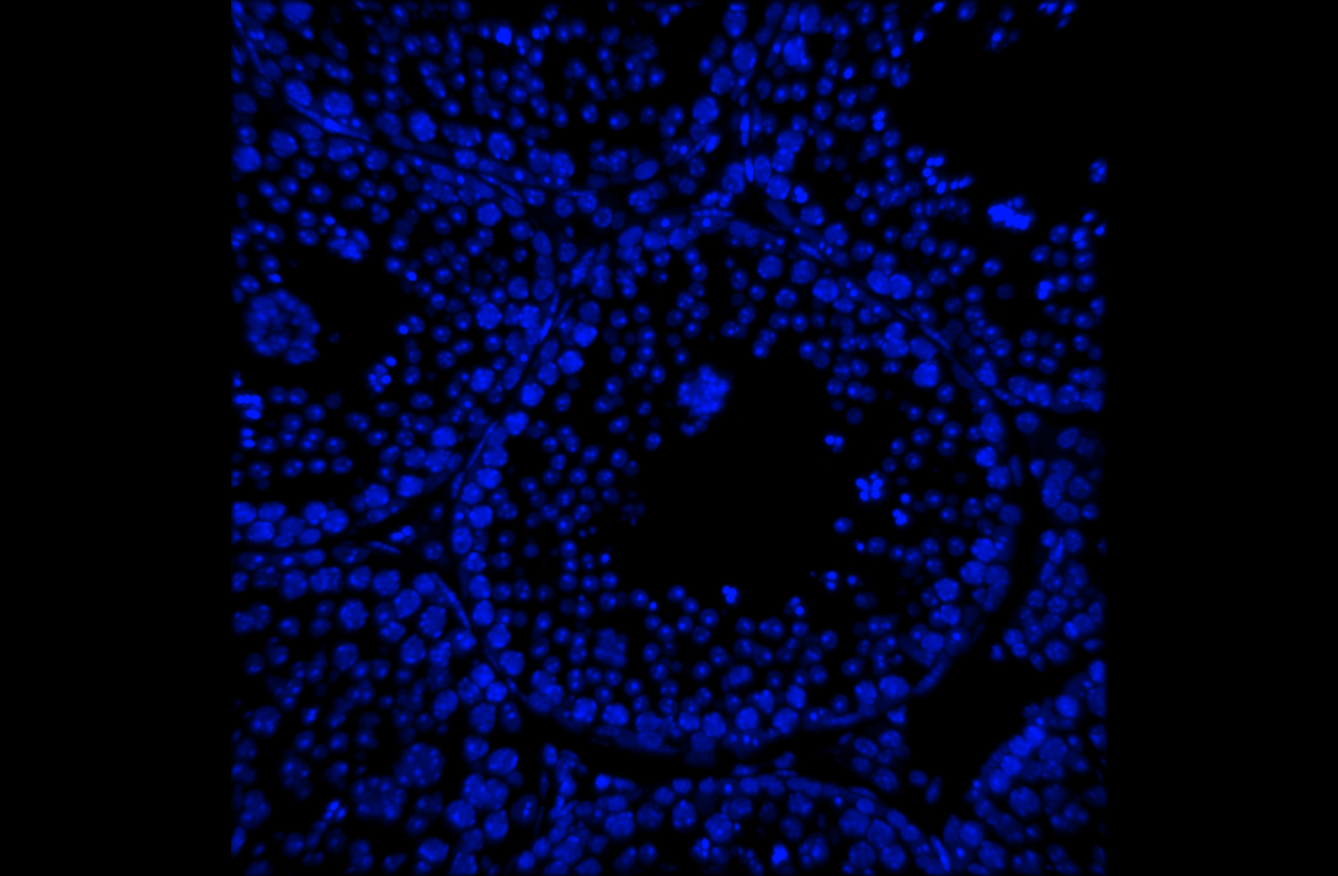

Supplement: Figure 7—source data 1. [file elife-83129-fig7-data1.zip › Figure7/Source data of Figure7B/hs-pd60-KO-DAPI-1.tif]

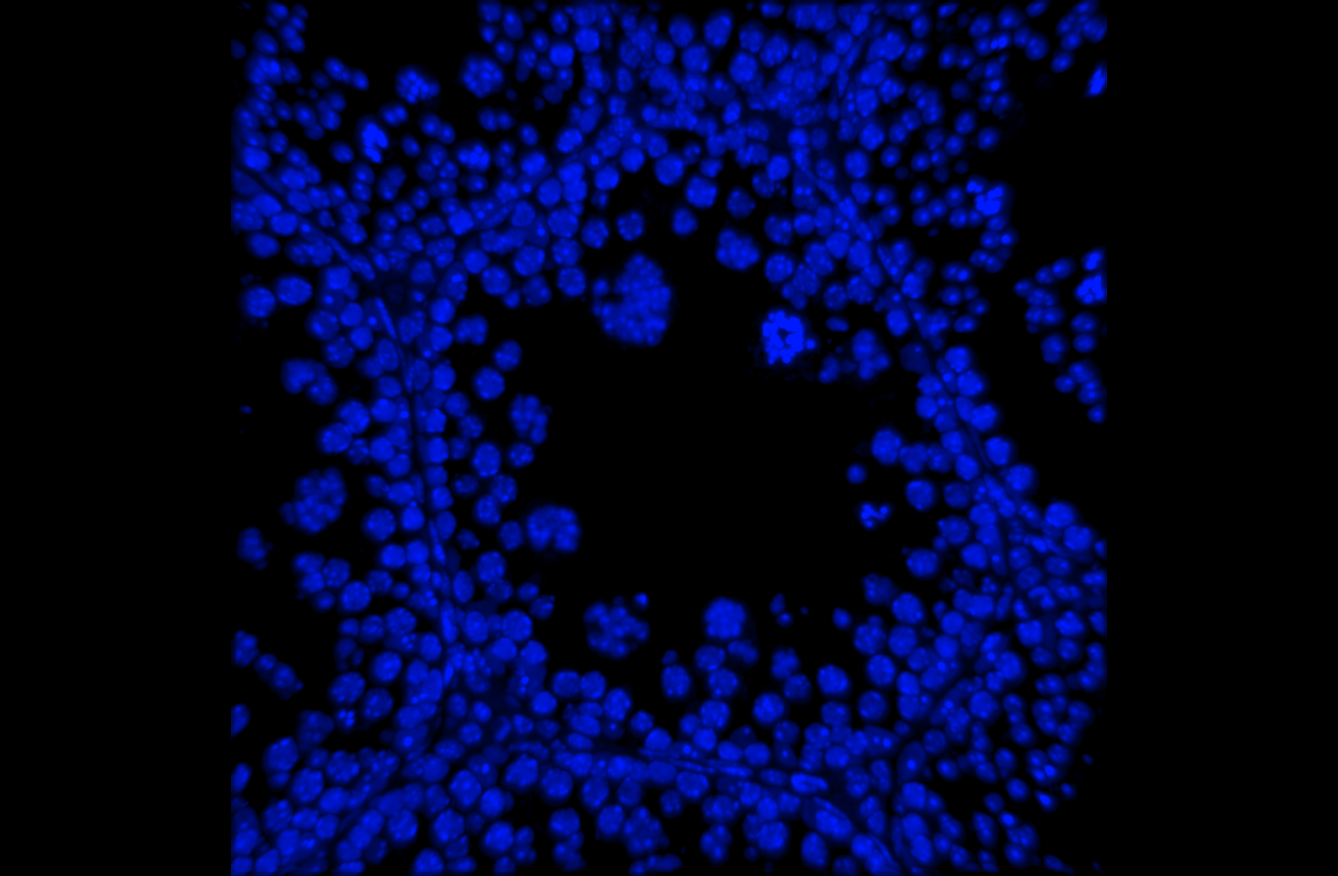

Supplement: Figure 7—source data 1. [file elife-83129-fig7-data1.zip › Figure7/Source data of Figure7B/hs-pd60-KO-DAPI-2.tif]

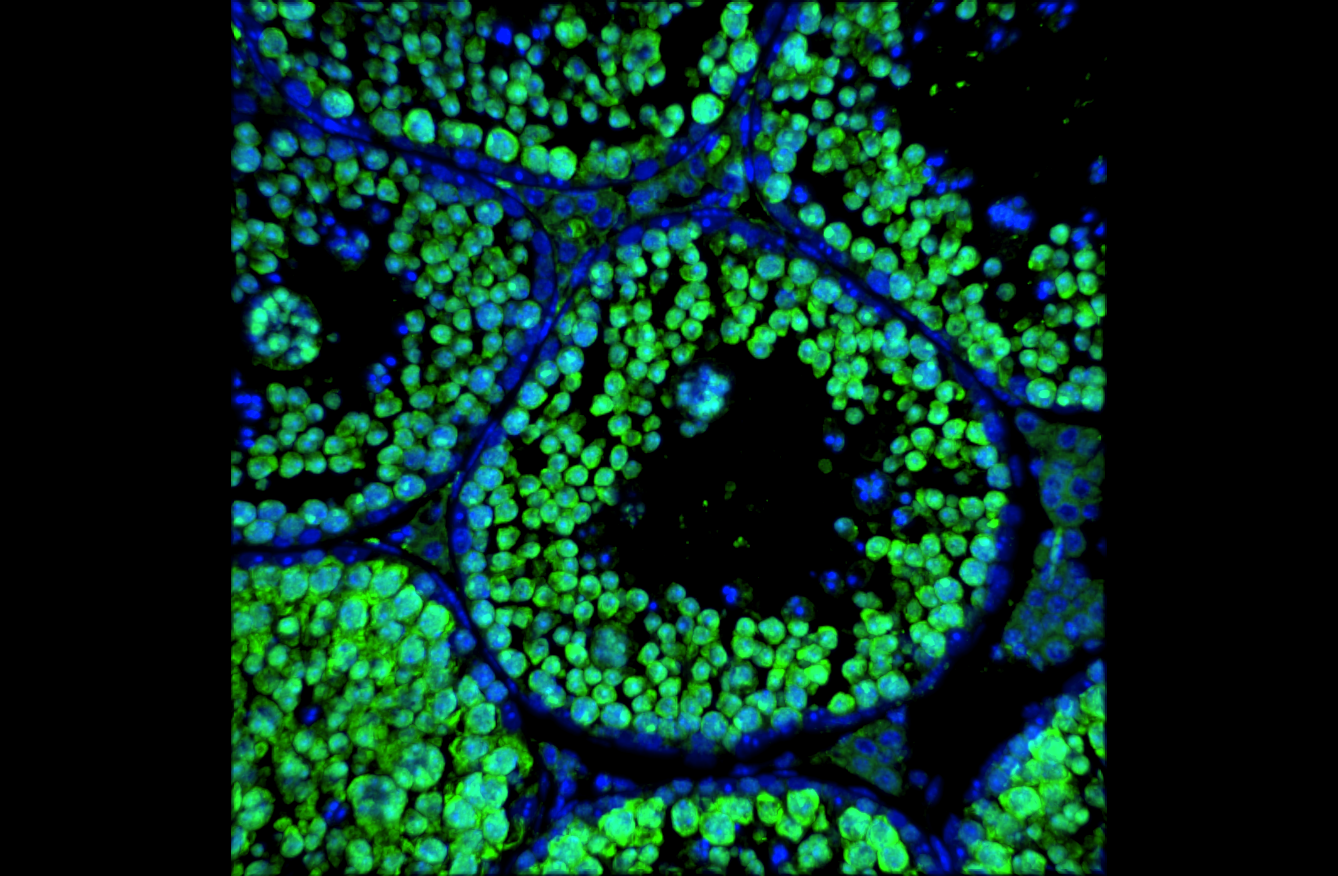

Supplement: Figure 7—source data 1. [file elife-83129-fig7-data1.zip › Figure7/Source data of Figure7B/hs-pd60-KO-MERGE-1.tif]

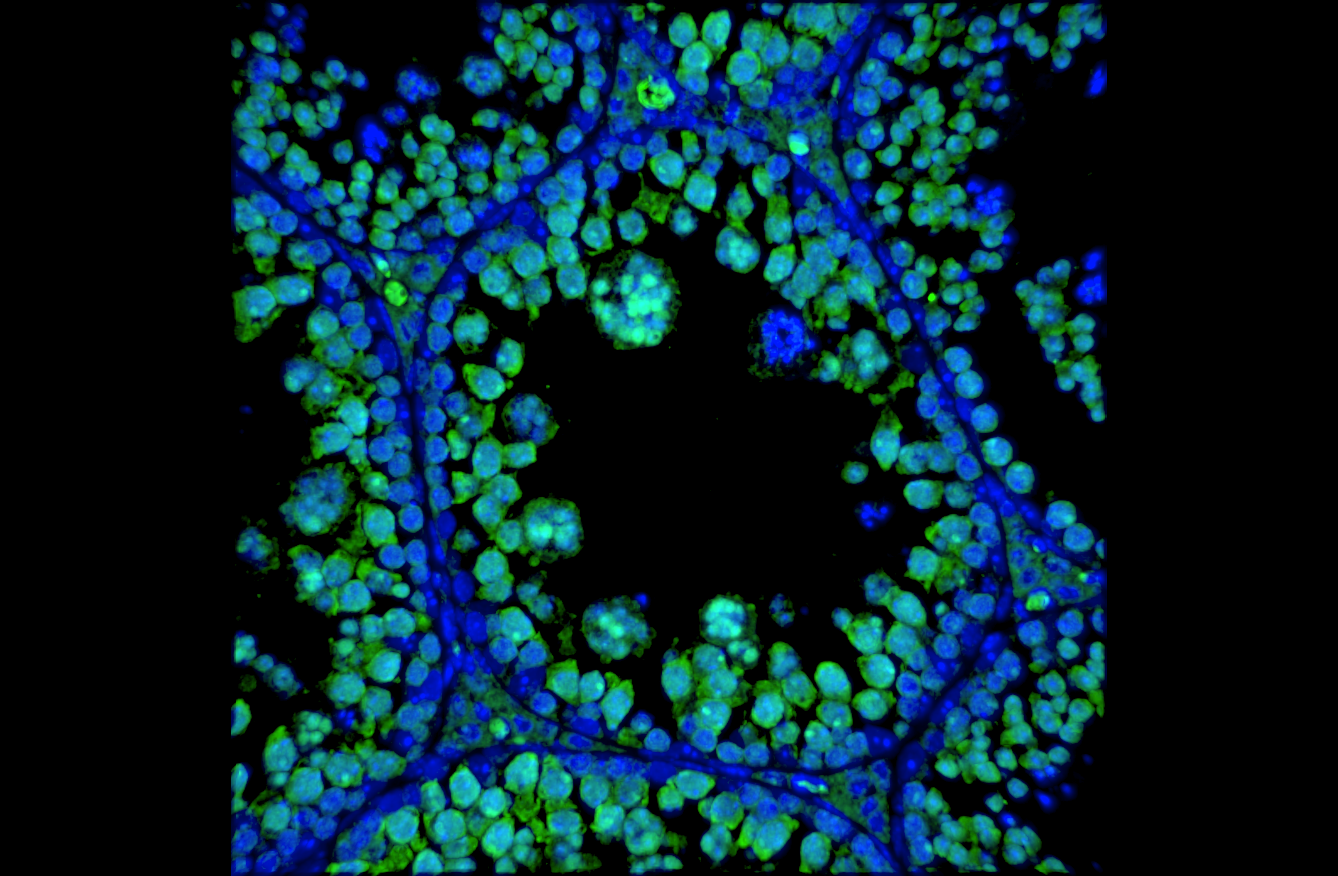

Supplement: Figure 7—source data 1. [file elife-83129-fig7-data1.zip › Figure7/Source data of Figure7B/hs-pd60-KO-MERGE-2.tif]

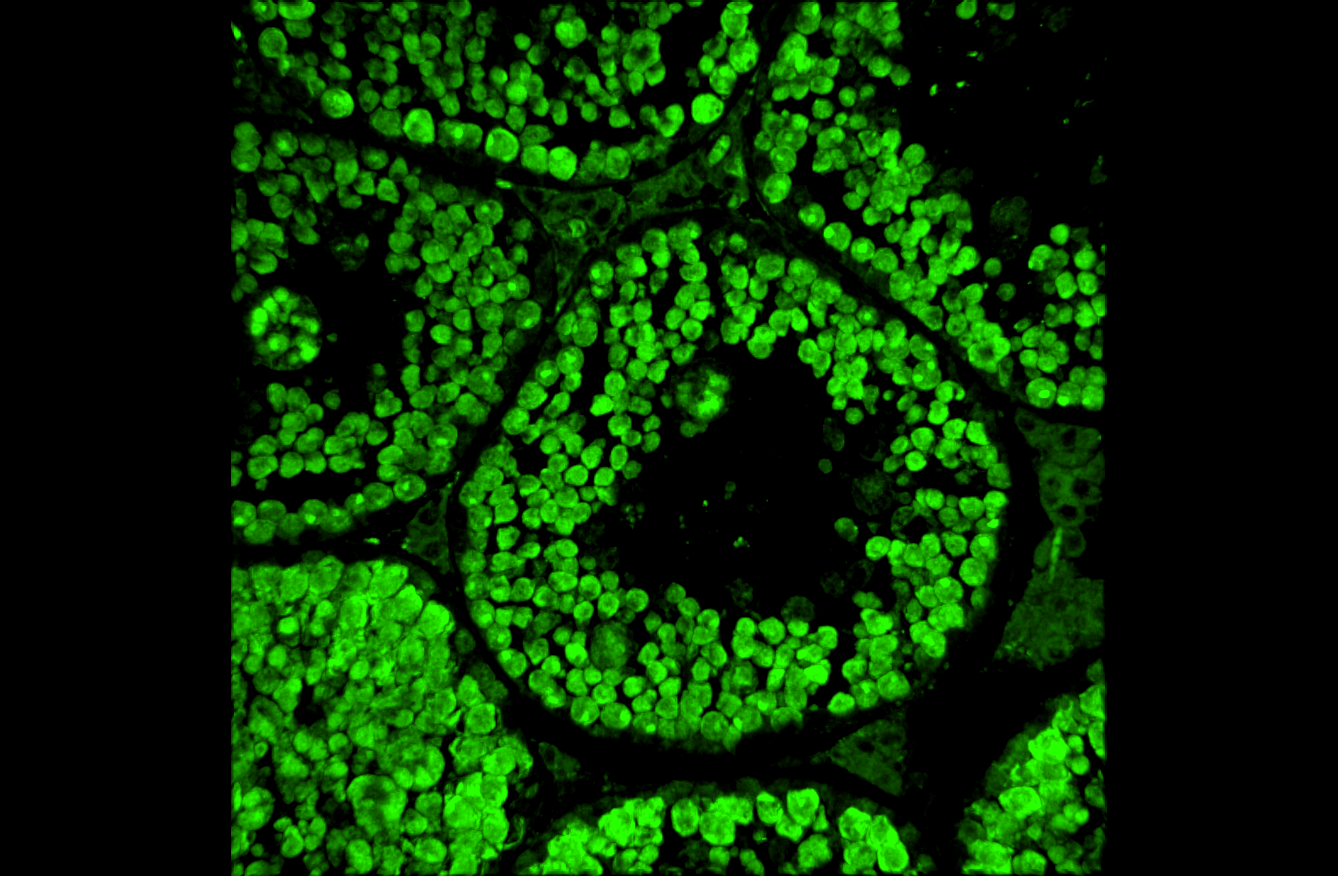

Supplement: Figure 7—source data 1. [file elife-83129-fig7-data1.zip › Figure7/Source data of Figure7B/hs-pd60-KO-PCOFILIN-1.tif]

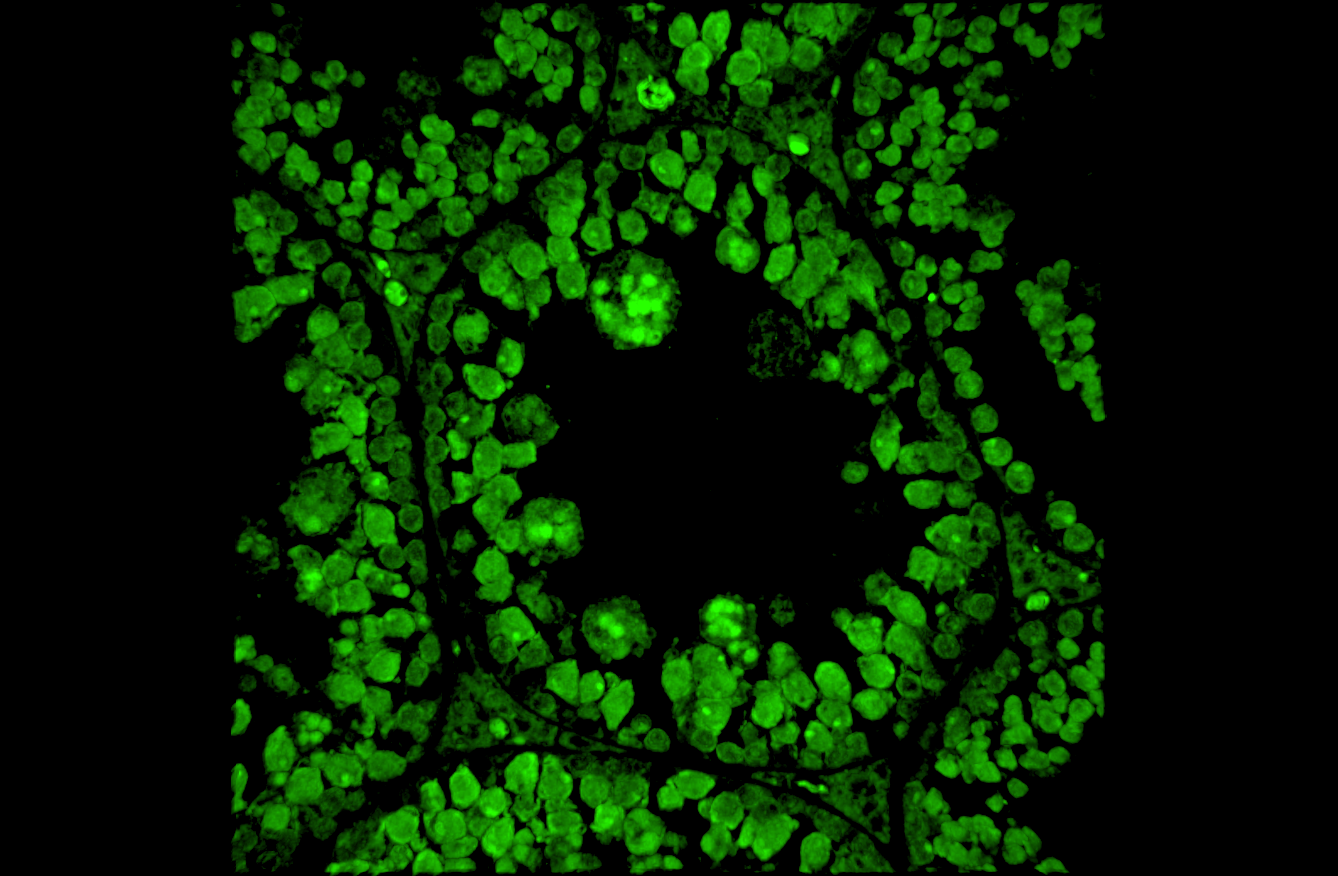

Supplement: Figure 7—source data 1. [file elife-83129-fig7-data1.zip › Figure7/Source data of Figure7B/hs-pd60-KO-PCOFILIN-2.tif]

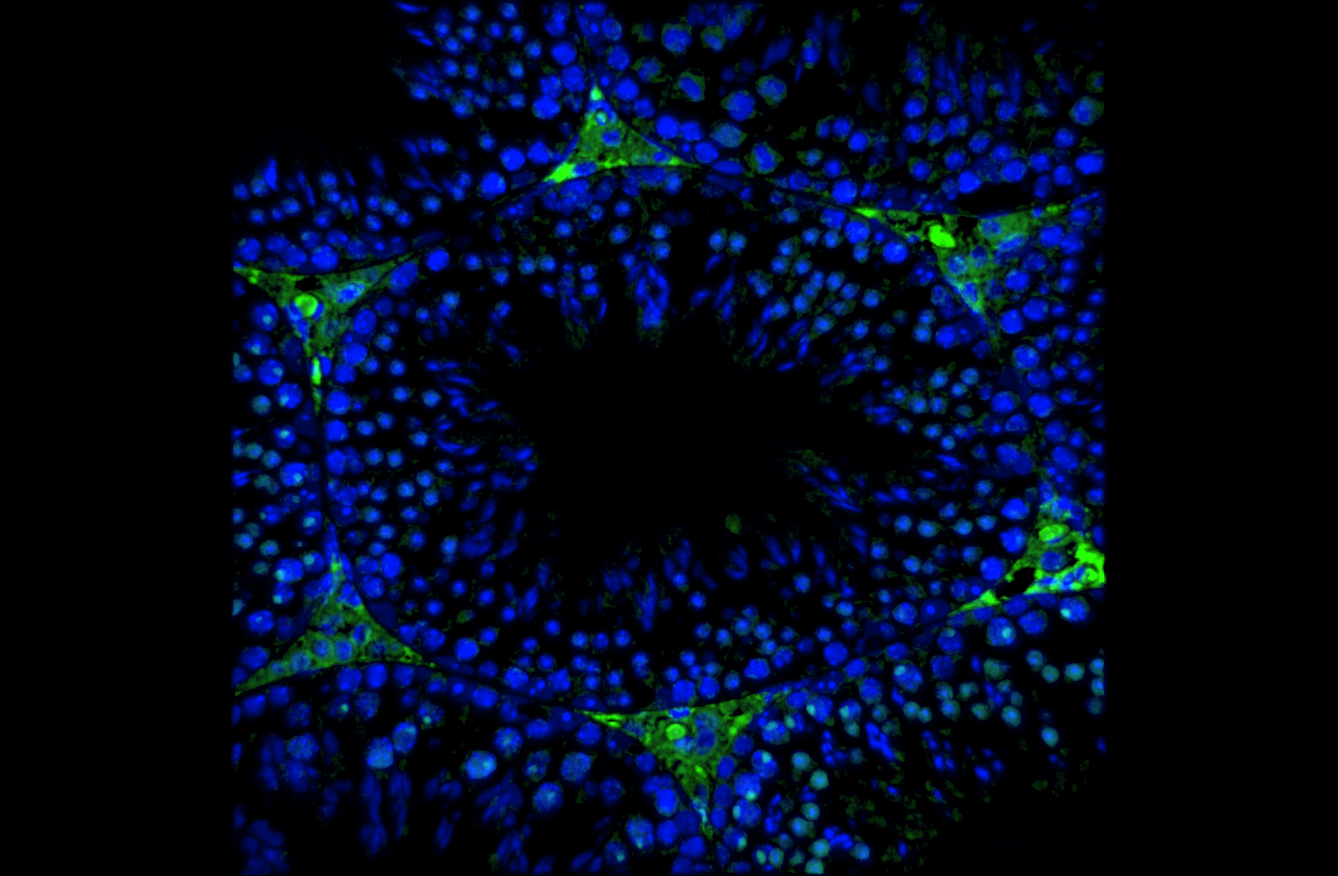

Supplement: Figure 7—source data 1. [file elife-83129-fig7-data1.zip › Figure7/Source data of Figure7B/hs-pd60-MERGE-1.tif]

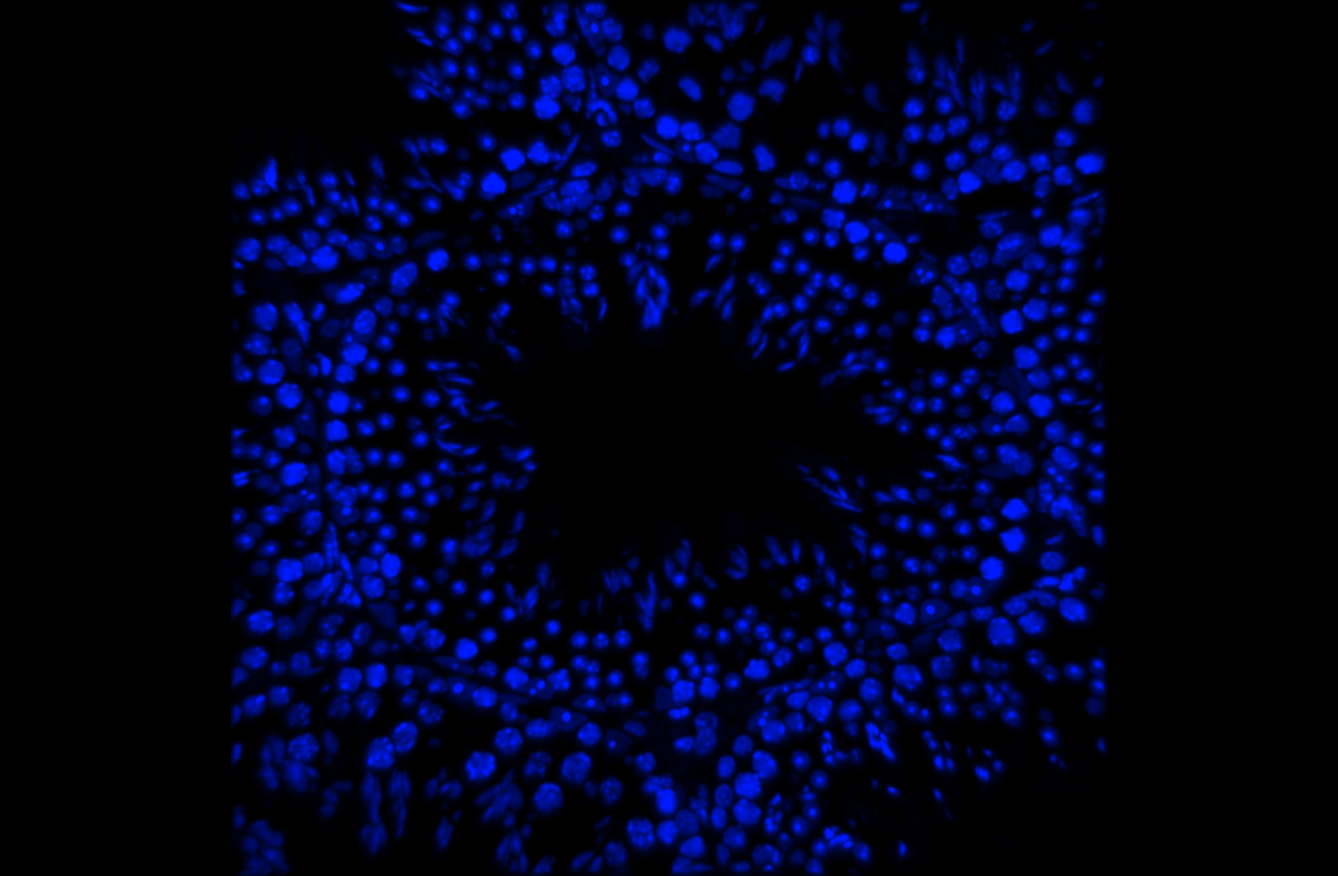

Supplement: Figure 7—source data 1. [file elife-83129-fig7-data1.zip › Figure7/Source data of Figure7B/hs-pd60-WT-DAPI-1.tif]

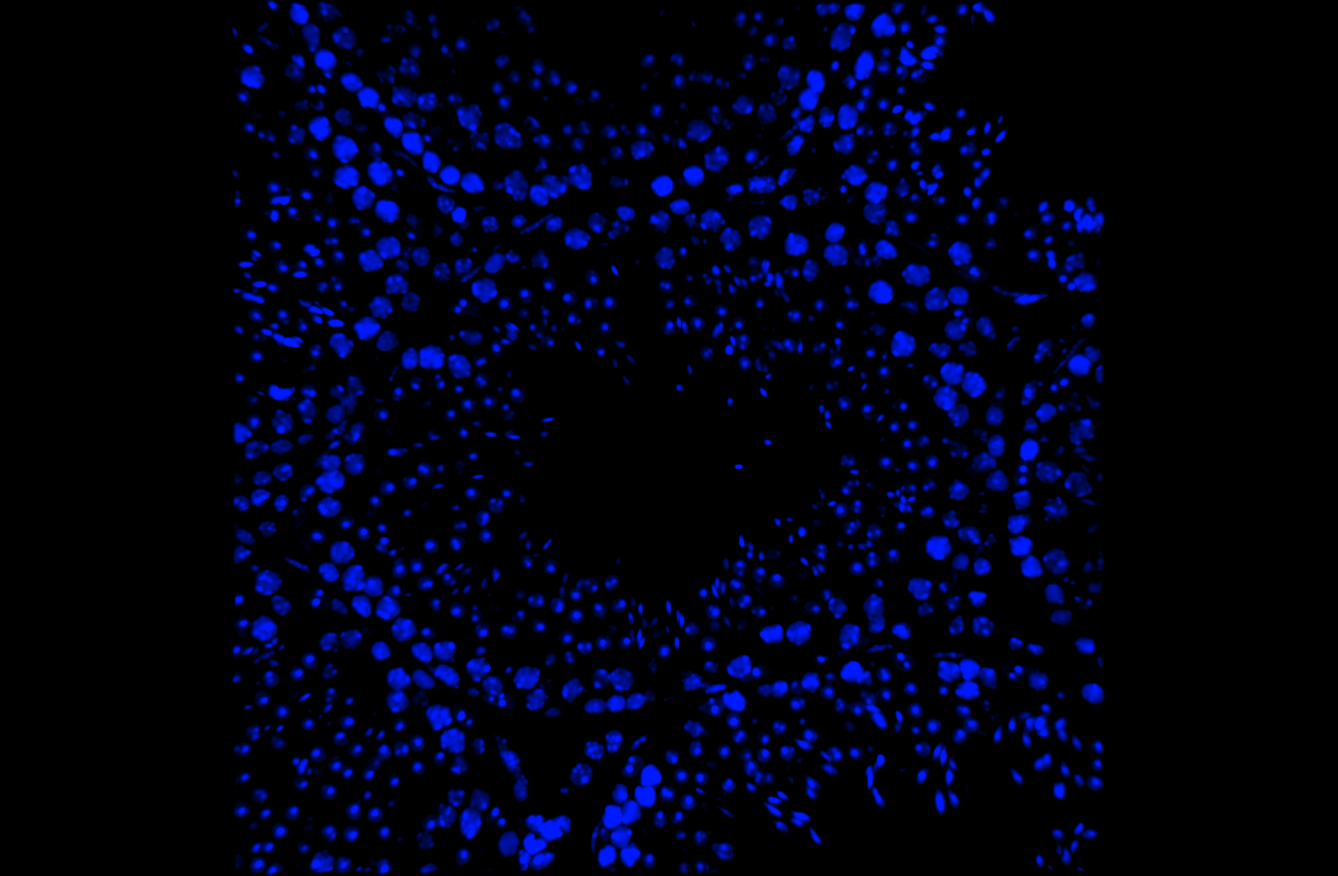

Supplement: Figure 7—source data 1. [file elife-83129-fig7-data1.zip › Figure7/Source data of Figure7B/hs-pd60-WT-DAPI-2.tif]

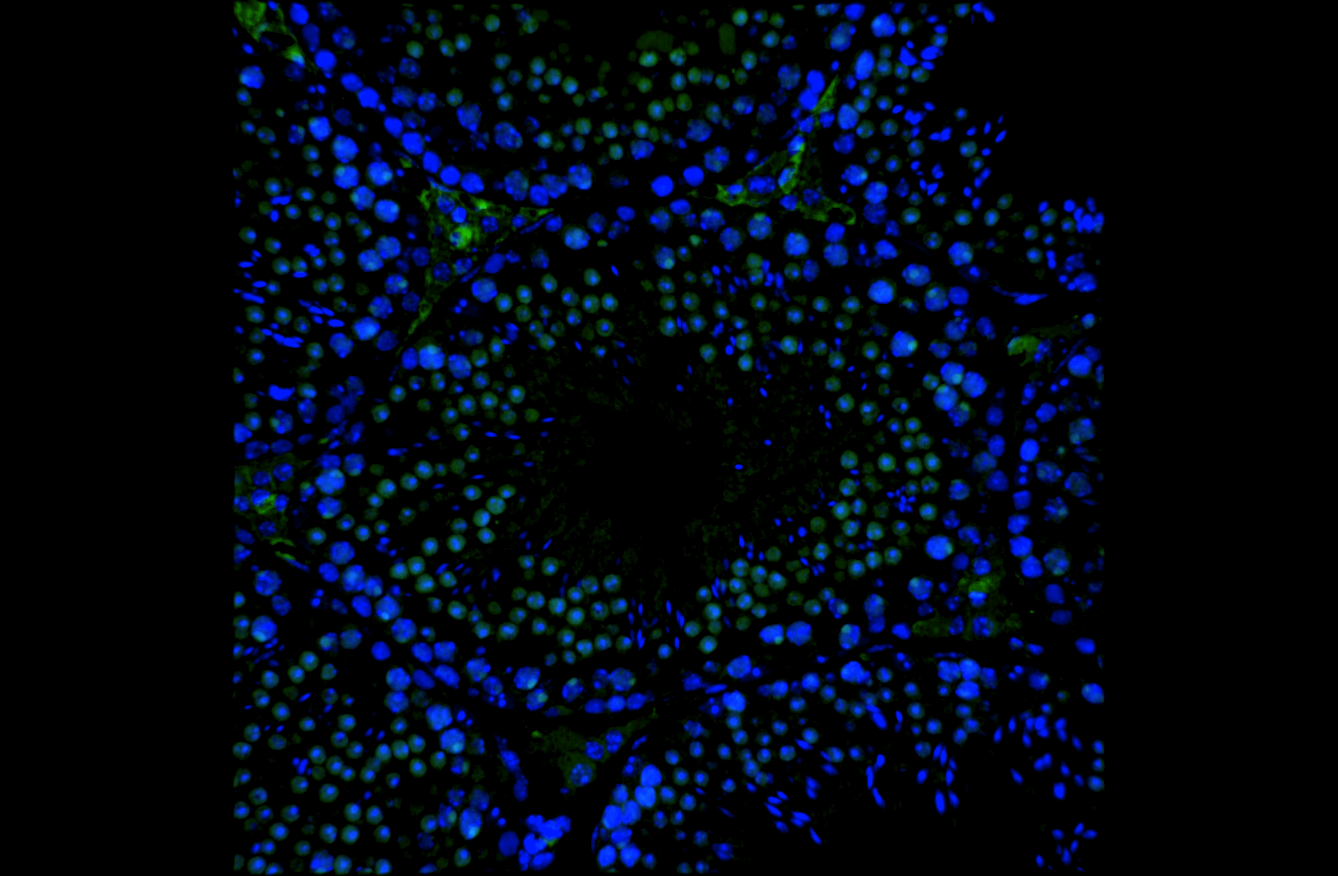

Supplement: Figure 7—source data 1. [file elife-83129-fig7-data1.zip › Figure7/Source data of Figure7B/hs-pd60-WT-MERGE-2.tif]

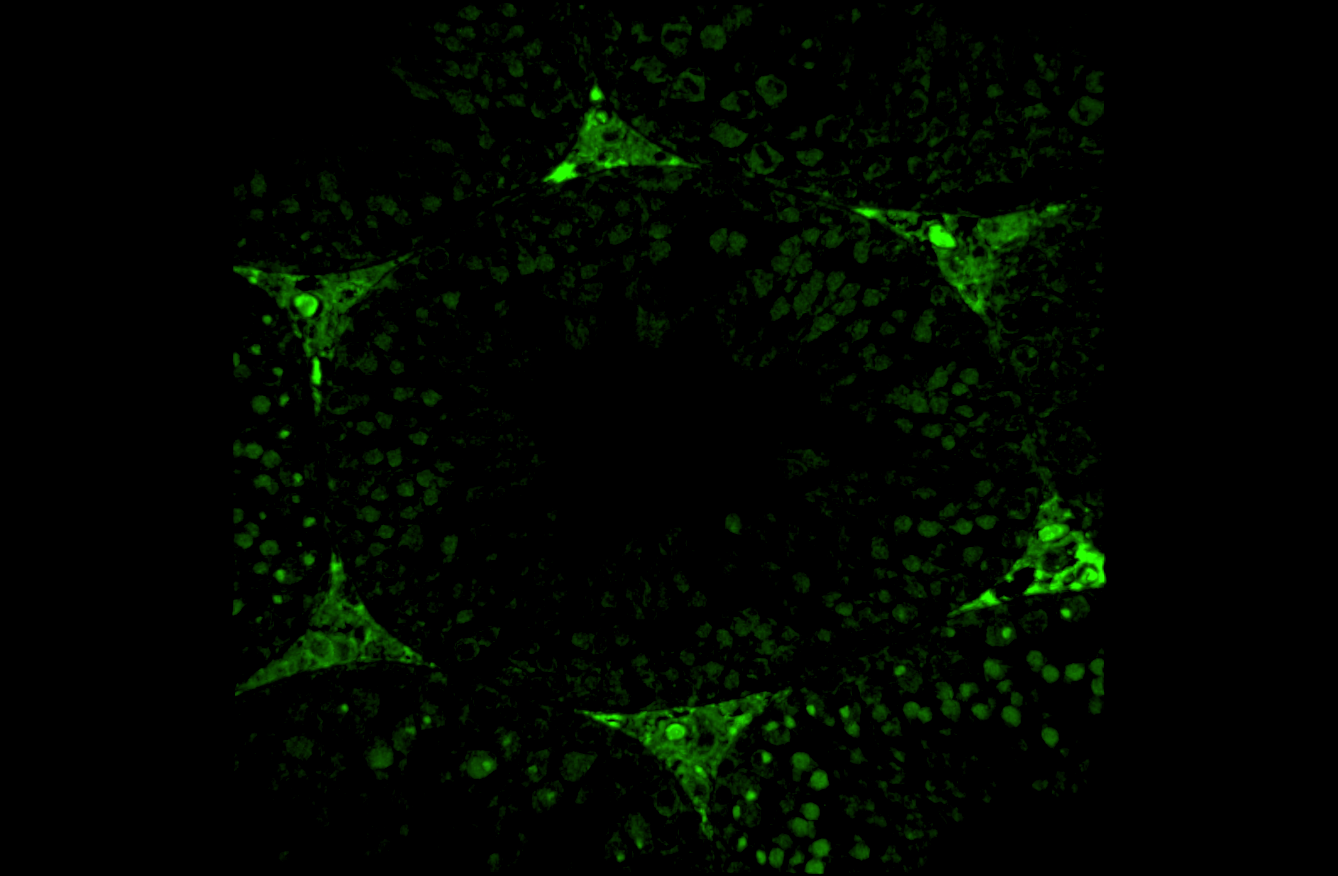

Supplement: Figure 7—source data 1. [file elife-83129-fig7-data1.zip › Figure7/Source data of Figure7B/hs-pd60-WT-PCOFILIN-1.tif]

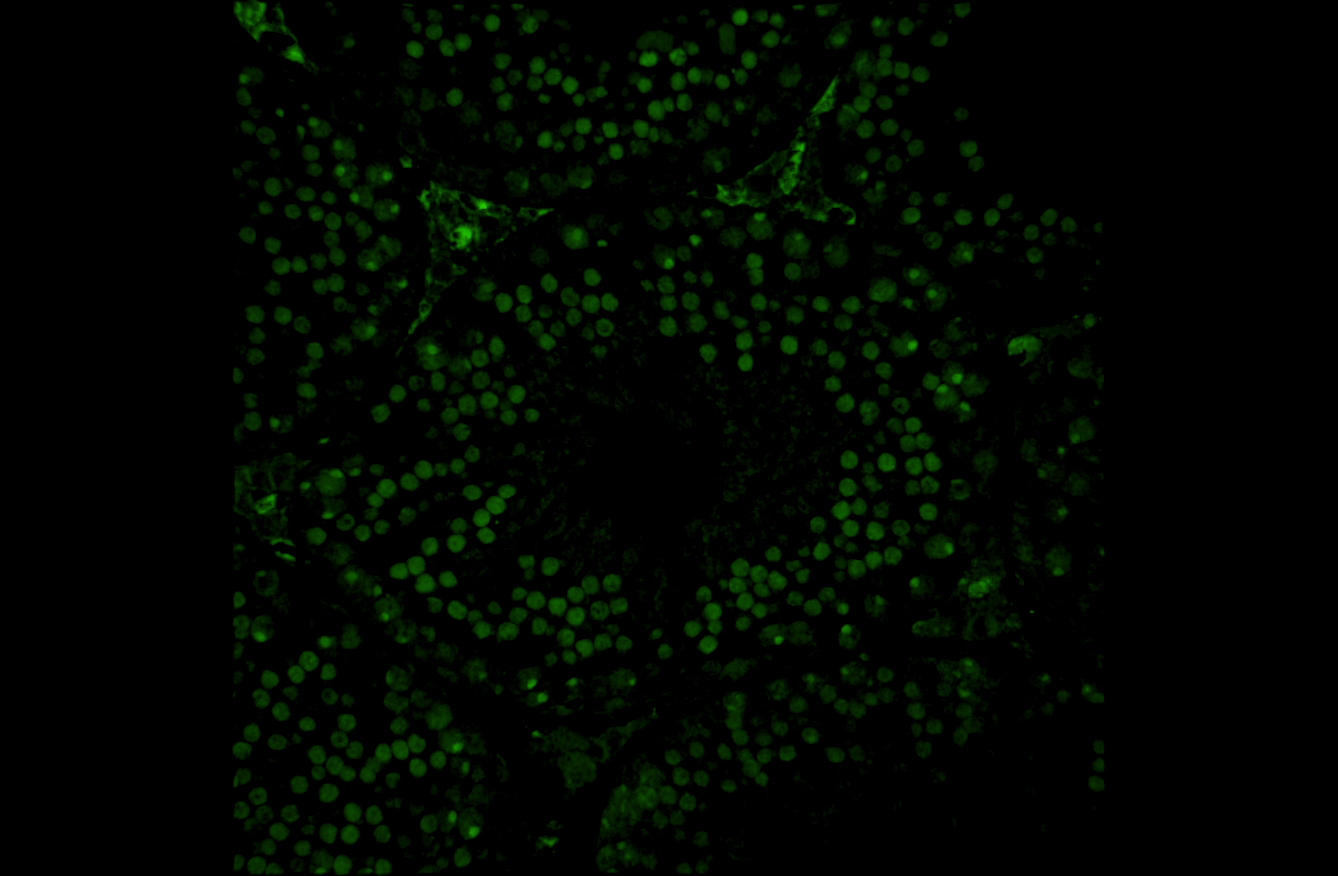

Supplement: Figure 7—source data 1. [file elife-83129-fig7-data1.zip › Figure7/Source data of Figure7B/hs-pd60-WT-PCOFILIN-2.tif]

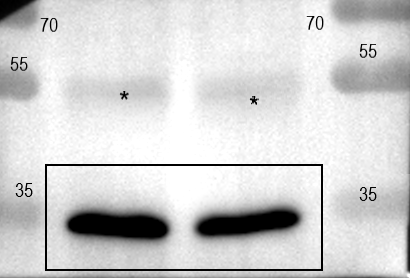

Supplement: Figure 7—source data 1. [file elife-83129-fig7-data1.zip › Figure7/Source data of Figure7C/Labelled blots of GAPDH-2.tiff]

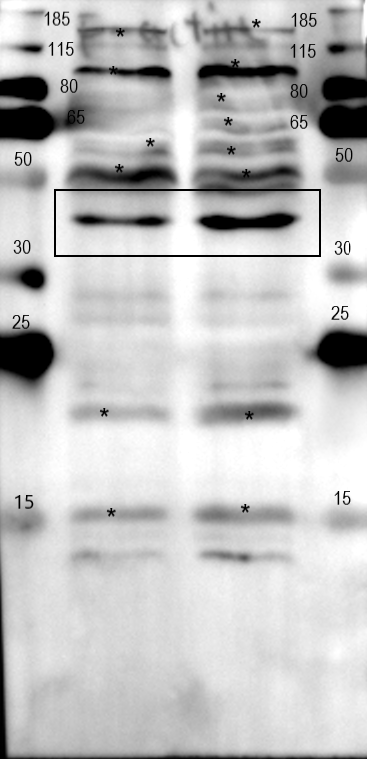

Supplement: Figure 7—source data 1. [file elife-83129-fig7-data1.zip › Figure7/Source data of Figure7C/Labelled blots of F-ACTIN.tiff]

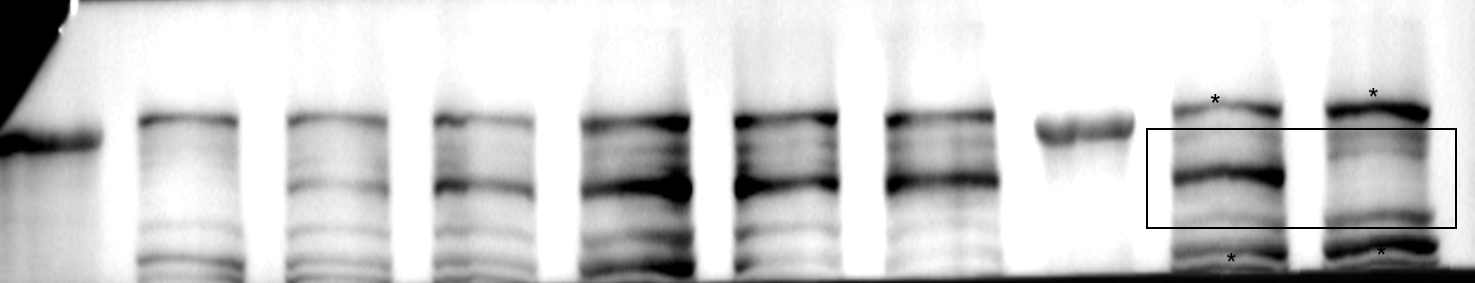

Supplement: Figure 7—source data 1. [file elife-83129-fig7-data1.zip › Figure7/Source data of Figure7C/Labelled blots of SSH2-2.tif]

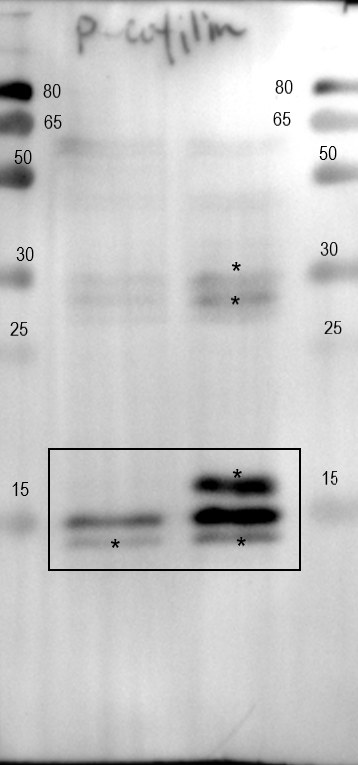

Supplement: Figure 7—source data 1. [file elife-83129-fig7-data1.zip › Figure7/Source data of Figure7C/Labelled blots of p-COFILIN-2.tif]

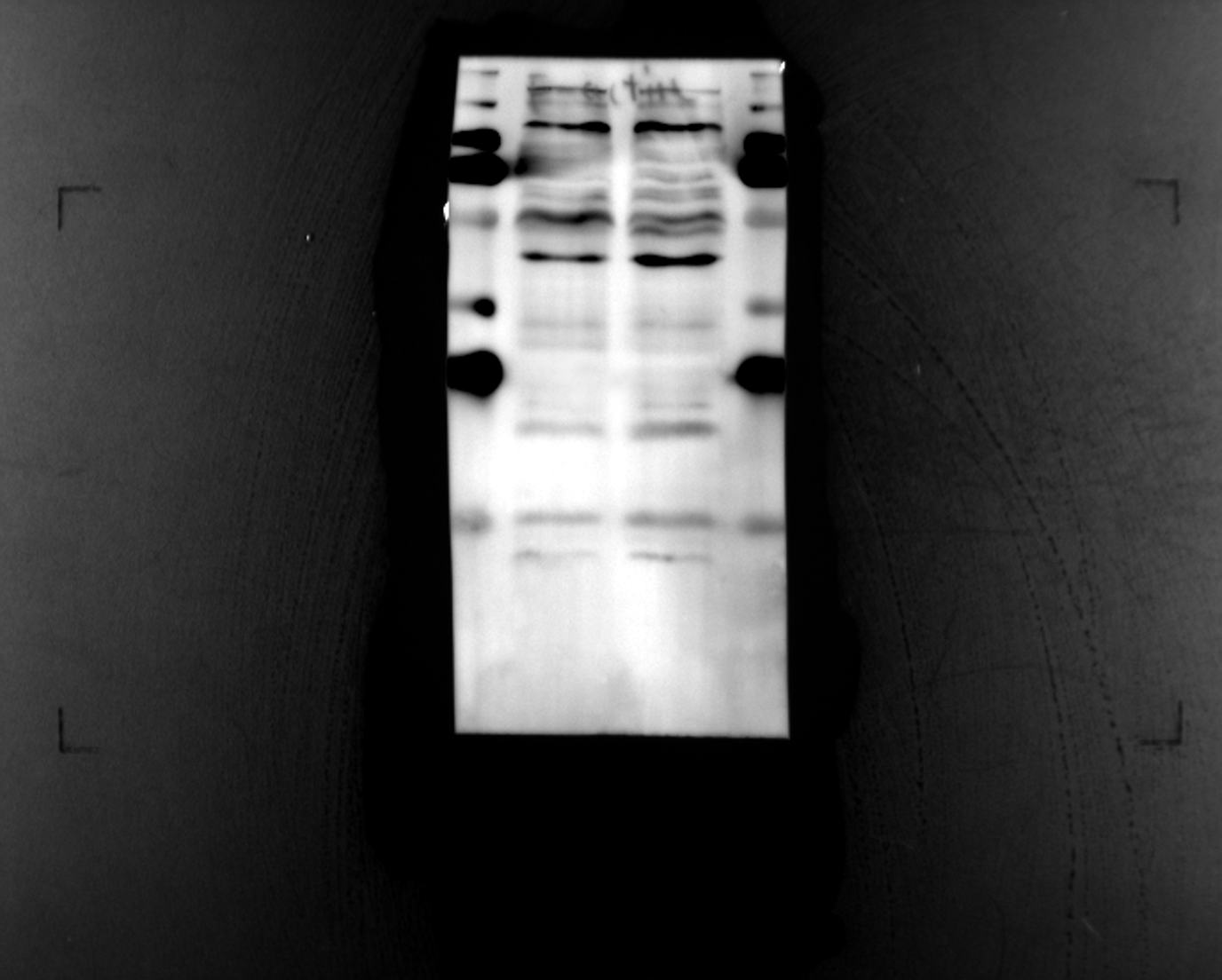

Supplement: Figure 7—source data 1. [file elife-83129-fig7-data1.zip › Figure7/Source data of Figure7C/Raw blots of F-ACTIN.Tif]

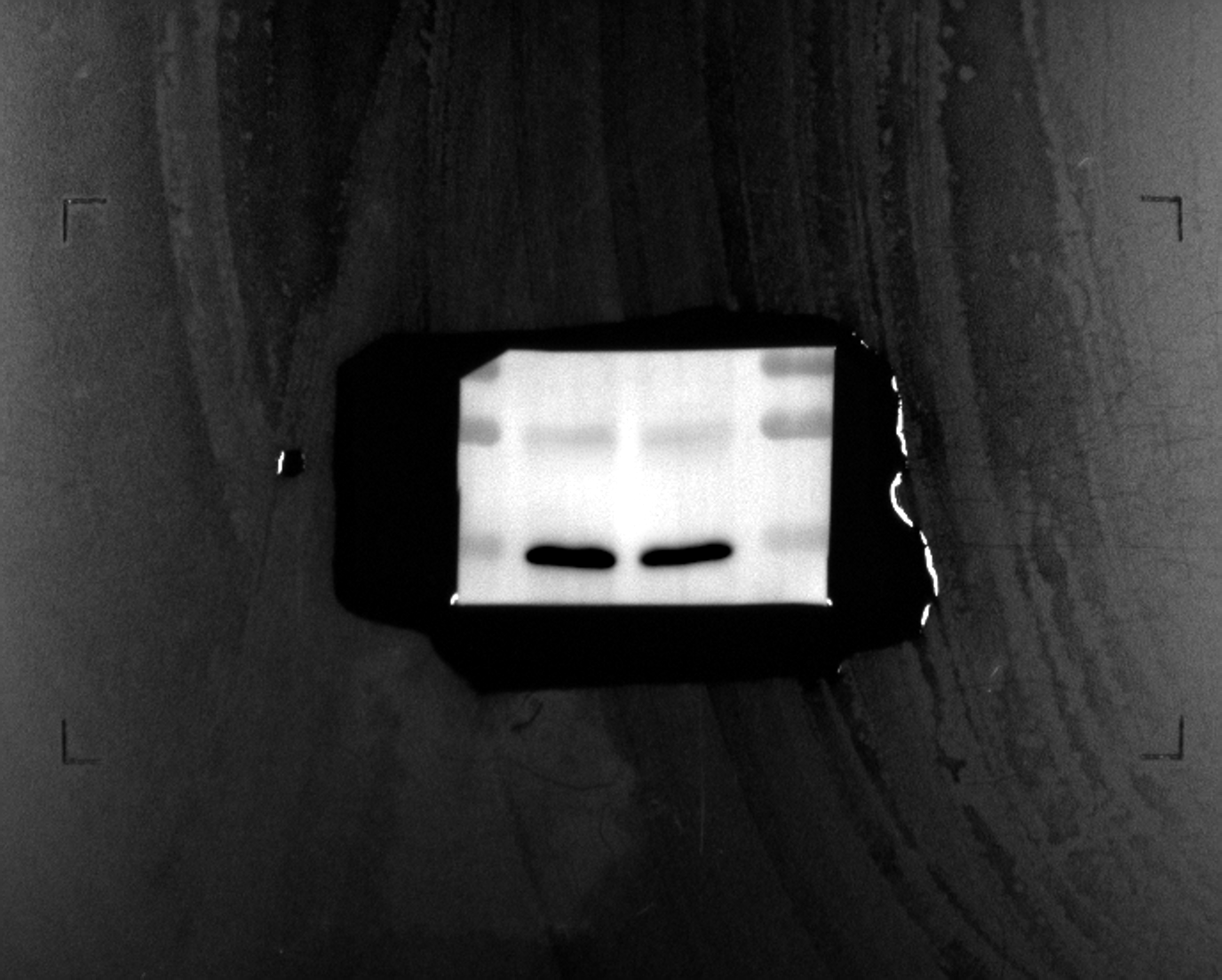

Supplement: Figure 7—source data 1. [file elife-83129-fig7-data1.zip › Figure7/Source data of Figure7C/Raw blots of GAPDH-2.Tif]

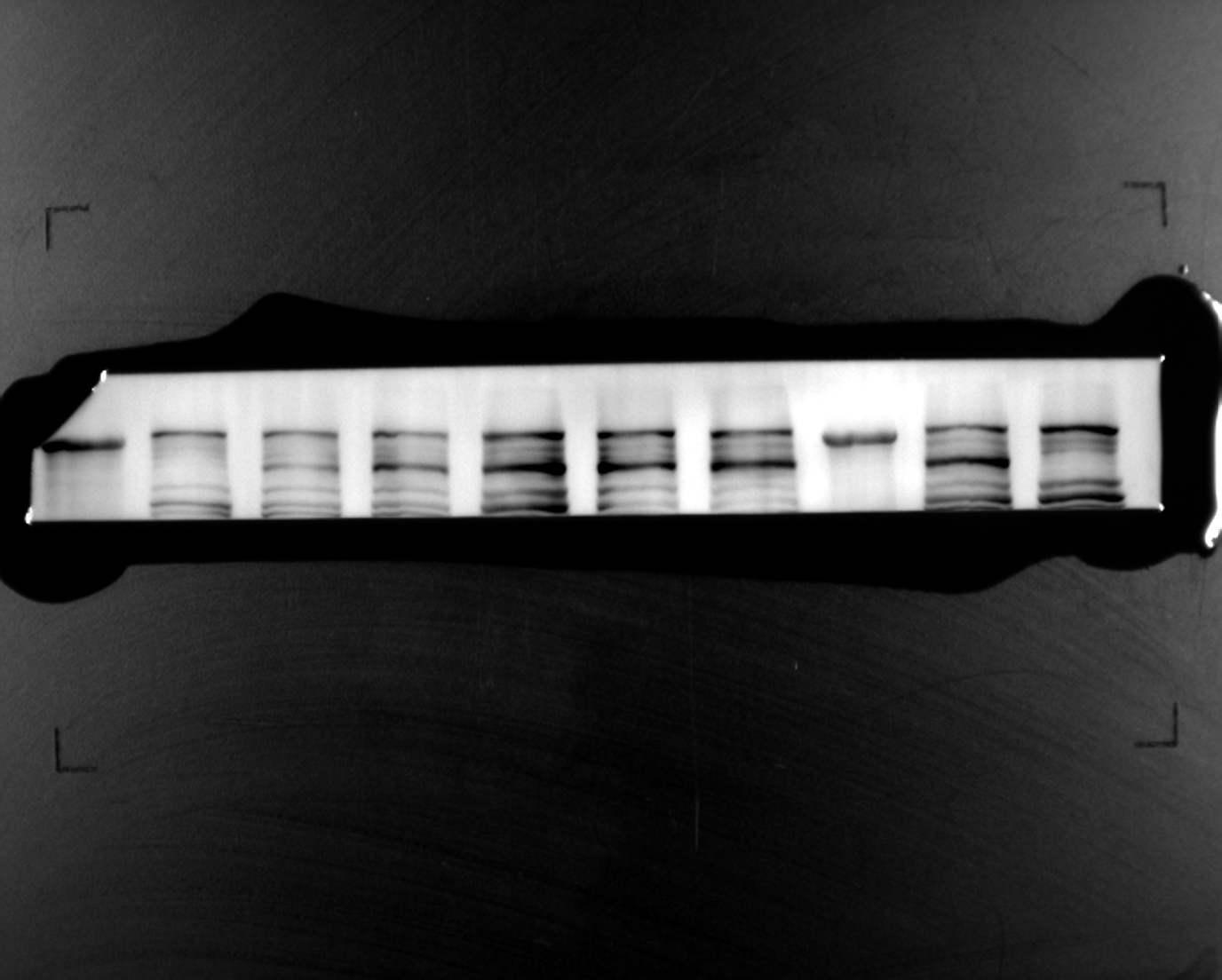

Supplement: Figure 7—source data 1. [file elife-83129-fig7-data1.zip › Figure7/Source data of Figure7C/Raw blots of SSH2-2.Tif]

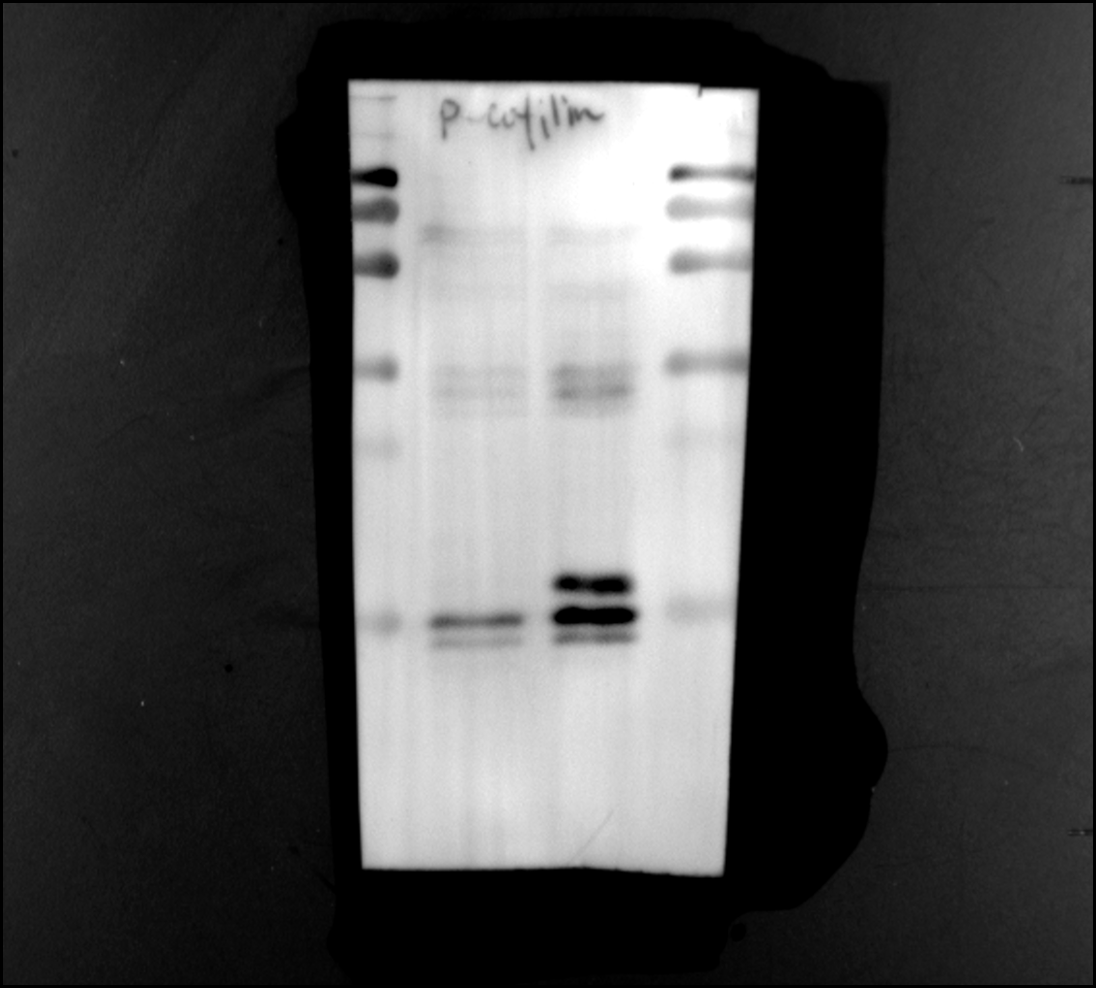

Supplement: Figure 7—source data 1. [file elife-83129-fig7-data1.zip › Figure7/Source data of Figure7C/Raw blots of p-COFILIN-2.tif]

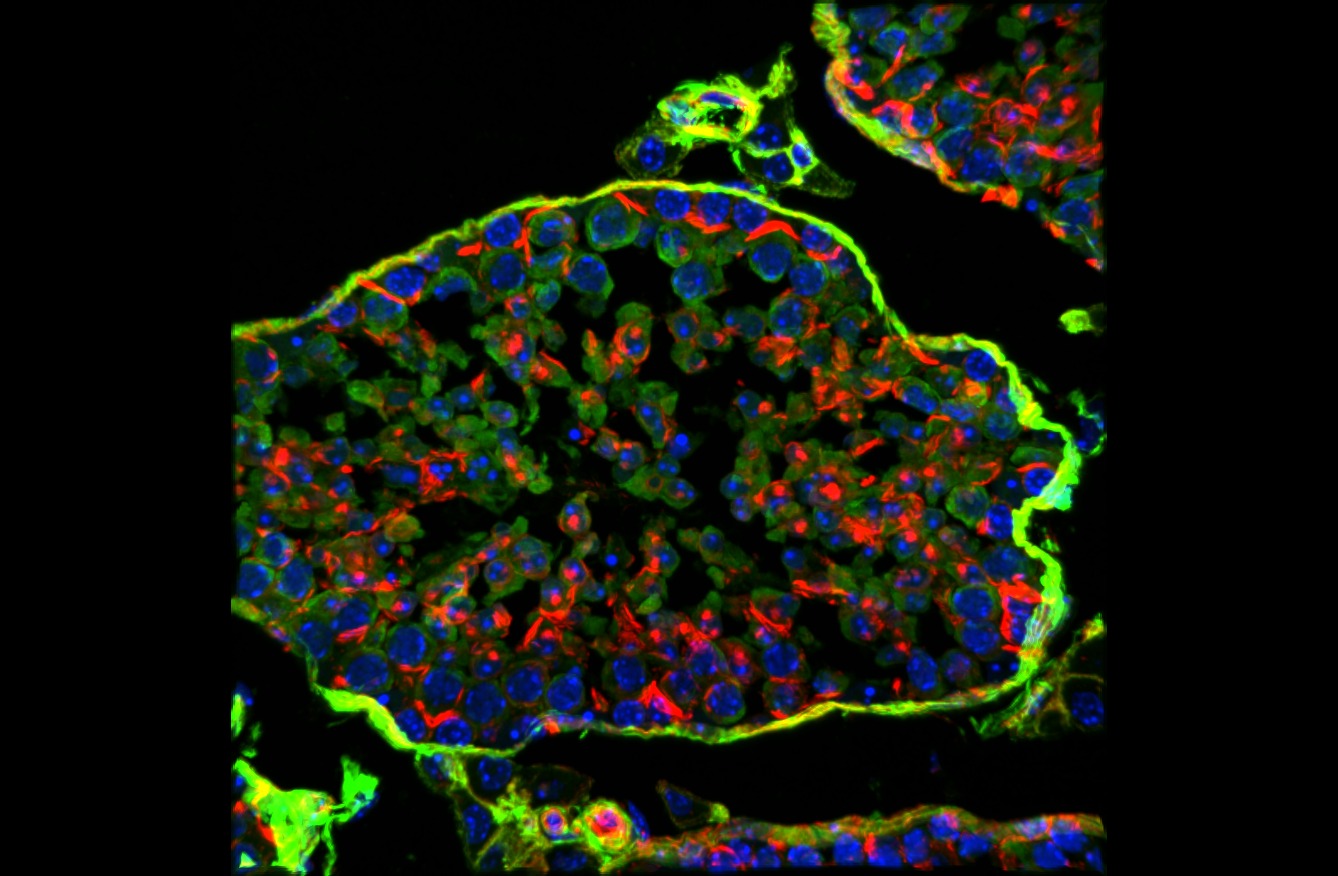

Supplement: Figure 7—source data 1. [file elife-83129-fig7-data1.zip › Figure7/Source data of Figure7D/FACTIN+PCOFILIN-pd65-KO-1.tif]

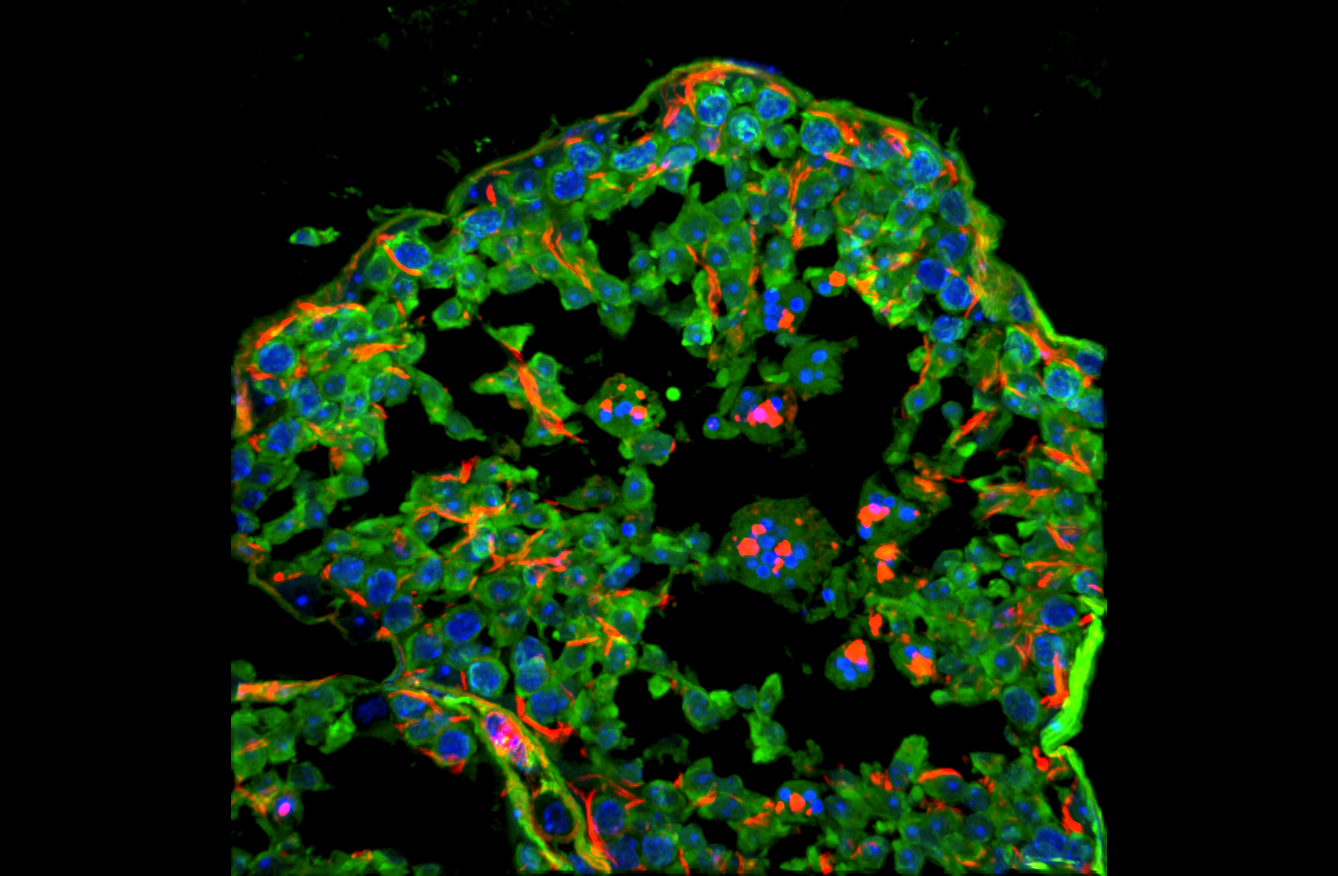

Supplement: Figure 7—source data 1. [file elife-83129-fig7-data1.zip › Figure7/Source data of Figure7D/FACTIN+PCOFILIN-pd65-KO-2.tif]

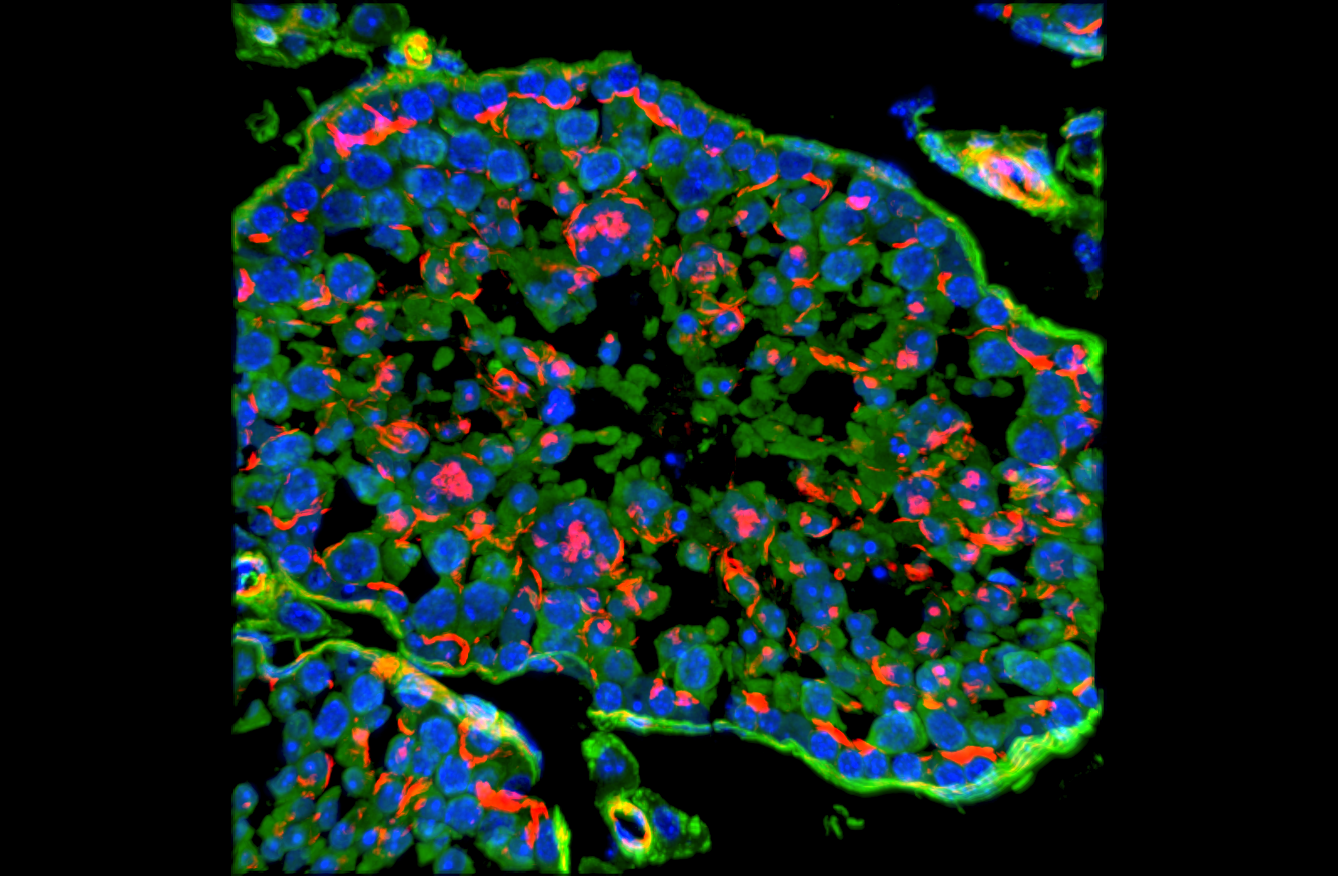

Supplement: Figure 7—source data 1. [file elife-83129-fig7-data1.zip › Figure7/Source data of Figure7D/FACTIN+PCOFILIN-pd65-KO-3.tif]

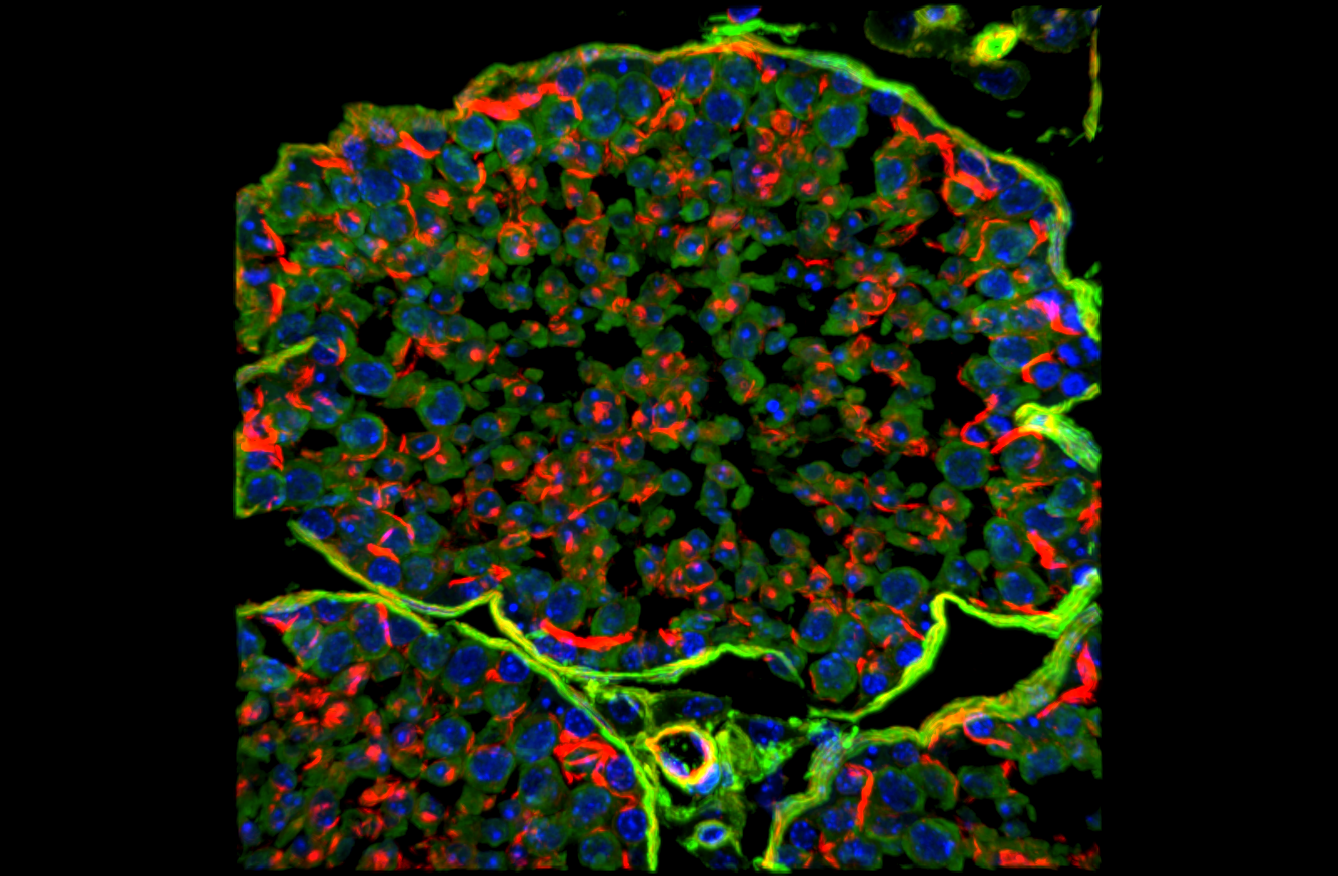

Supplement: Figure 7—source data 1. [file elife-83129-fig7-data1.zip › Figure7/Source data of Figure7D/FACTIN+PCOFILIN-pd65-KO-4.tif]

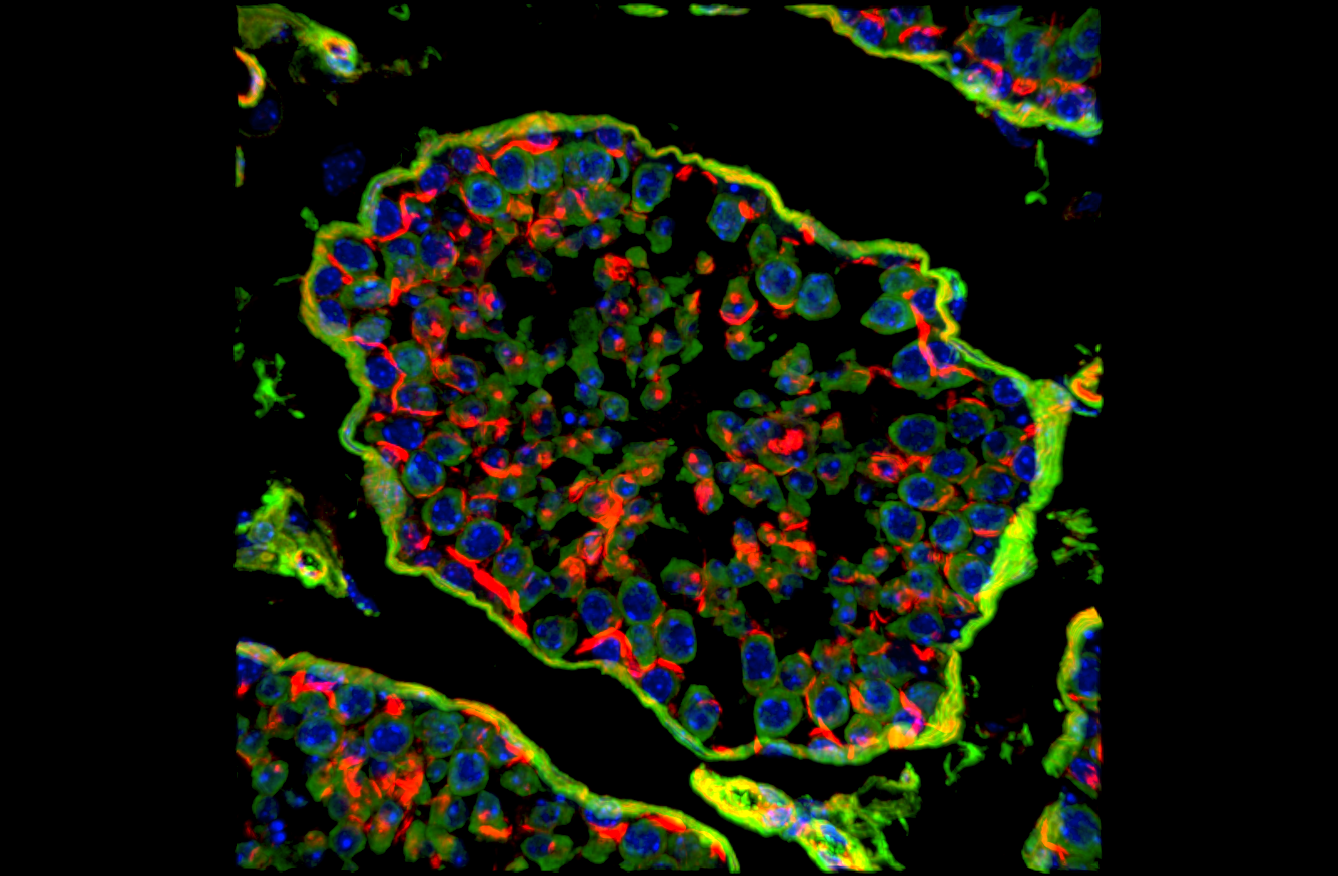

Supplement: Figure 7—source data 1. [file elife-83129-fig7-data1.zip › Figure7/Source data of Figure7D/FACTIN+PCOFILIN-pd65-KO-5.tif]

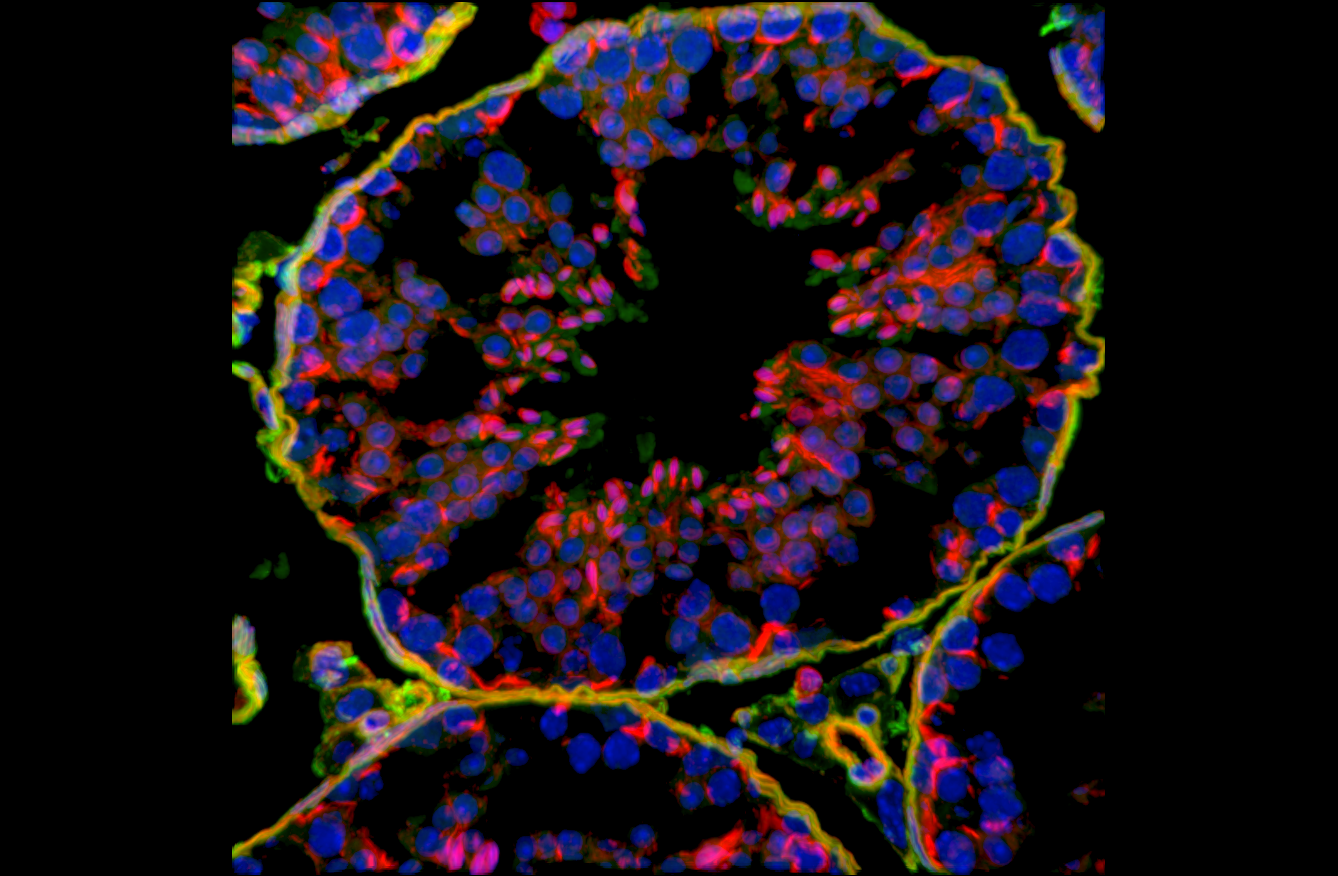

Supplement: Figure 7—source data 1. [file elife-83129-fig7-data1.zip › Figure7/Source data of Figure7D/FACTIN+PCOFILIN-pd65-WT-1.tif]

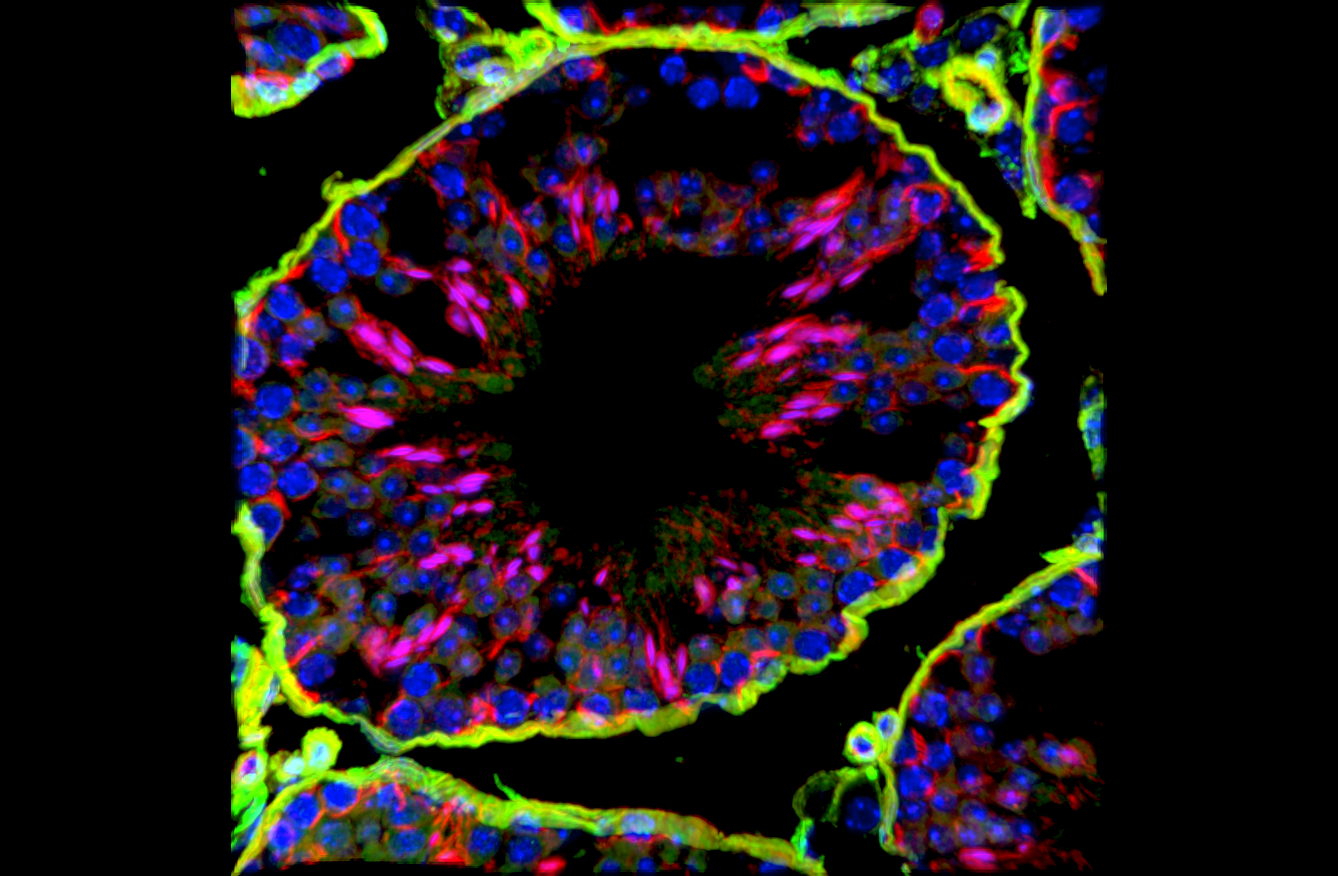

Supplement: Figure 7—source data 1. [file elife-83129-fig7-data1.zip › Figure7/Source data of Figure7D/FACTIN+PCOFILIN-pd65-WT-2.tif]

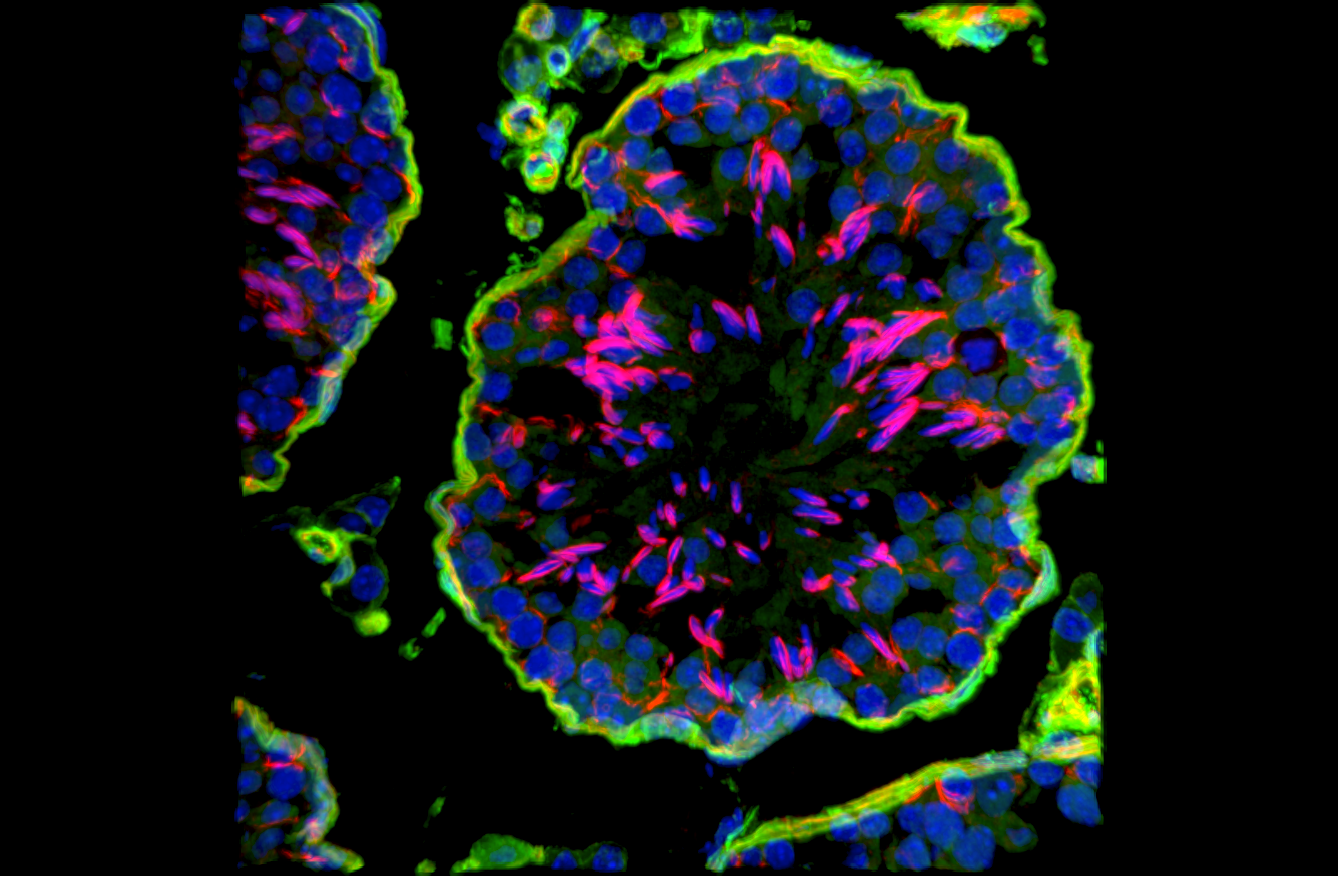

Supplement: Figure 7—source data 1. [file elife-83129-fig7-data1.zip › Figure7/Source data of Figure7D/FACTIN+PCOFILIN-pd65-WT-3.tif]

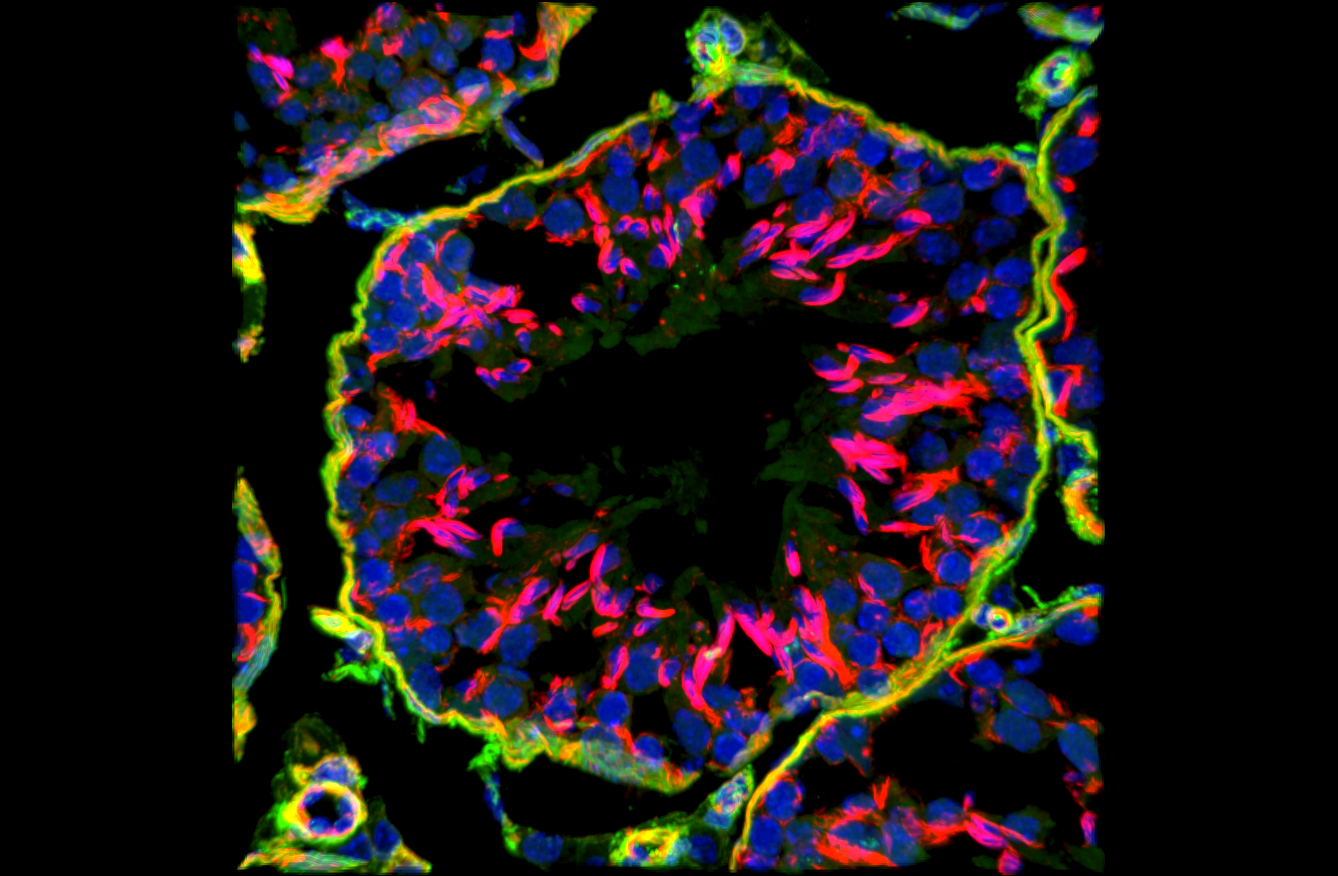

Supplement: Figure 7—source data 1. [file elife-83129-fig7-data1.zip › Figure7/Source data of Figure7D/FACTIN+PCOFILIN-pd65-WT-4.tif]

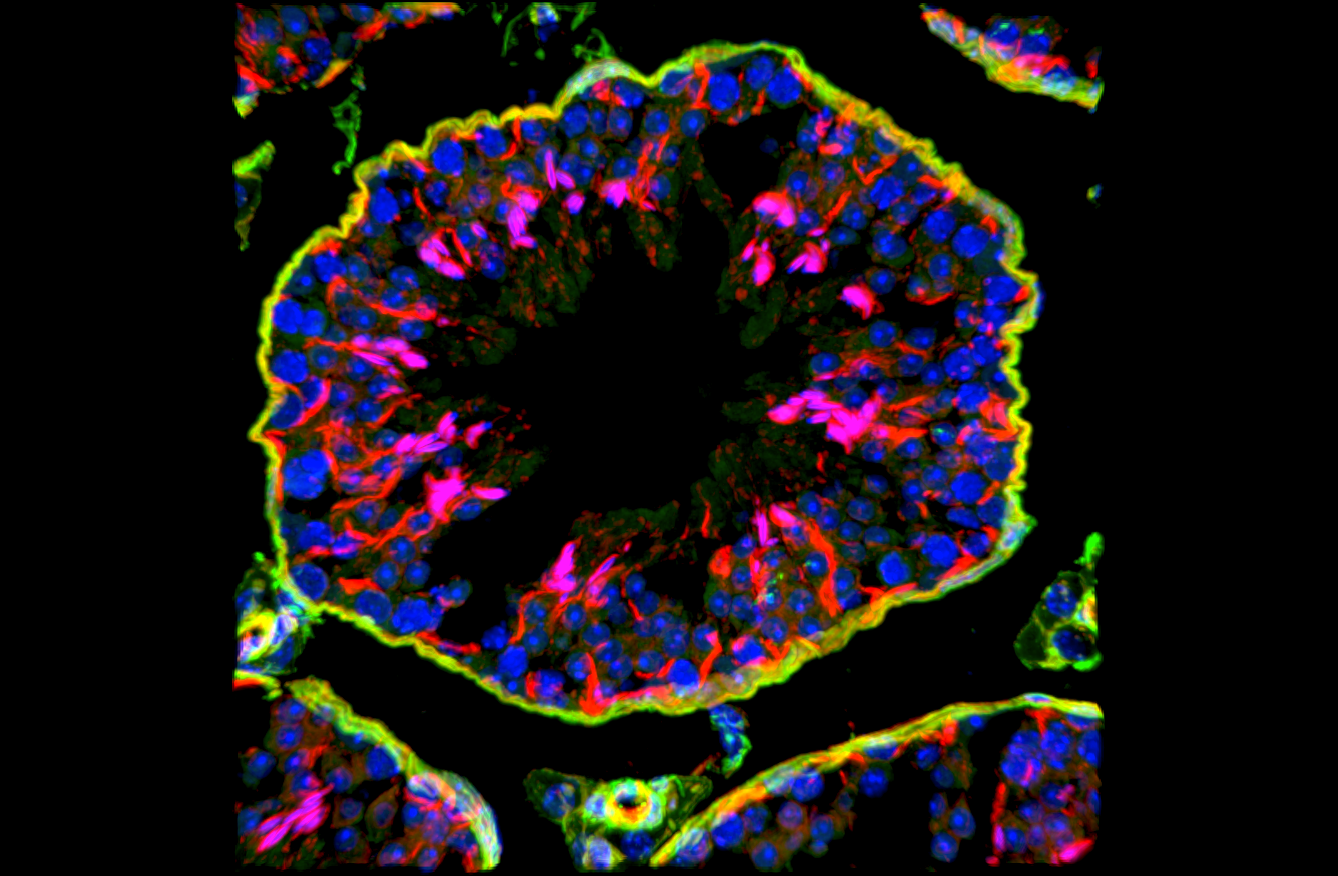

Supplement: Figure 7—source data 1. [file elife-83129-fig7-data1.zip › Figure7/Source data of Figure7D/FACTIN+PCOFILIN-pd65-WT-5.tif]

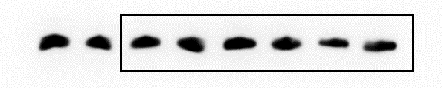

Supplement: Figure 7—figure supplement 1—source data 1. [file elife-83129-fig7-figsupp1-data1.zip › Figure supplement S6-source data 13/Labelled blots of COFILIN.tiff]

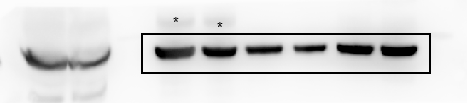

Supplement: Figure 7—figure supplement 1—source data 1. [file elife-83129-fig7-figsupp1-data1.zip › Figure supplement S6-source data 13/Labelled blots of GAPDH.tiff]

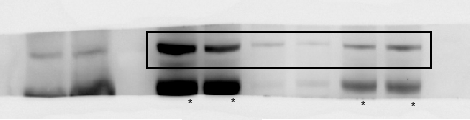

Supplement: Figure 7—figure supplement 1—source data 1. [file elife-83129-fig7-figsupp1-data1.zip › Figure supplement S6-source data 13/Labelled blots of LIMK1.tif]

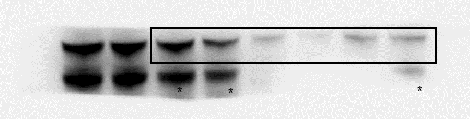

Supplement: Figure 7—figure supplement 1—source data 1. [file elife-83129-fig7-figsupp1-data1.zip › Figure supplement S6-source data 13/Labelled blots of LIMK2.tif]

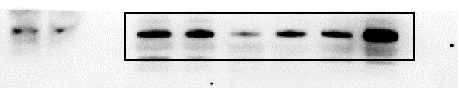

Supplement: Figure 7—figure supplement 1—source data 1. [file elife-83129-fig7-figsupp1-data1.zip › Figure supplement S6-source data 13/Labelled blots of p-COFILIN.tiff]

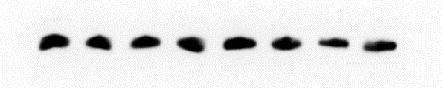

Supplement: Figure 7—figure supplement 1—source data 1. [file elife-83129-fig7-figsupp1-data1.zip › Figure supplement S6-source data 13/Raw blots of COFILIN.tif]

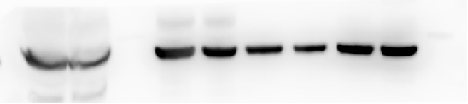

Supplement: Figure 7—figure supplement 1—source data 1. [file elife-83129-fig7-figsupp1-data1.zip › Figure supplement S6-source data 13/Raw blots of GAPDH.tif]

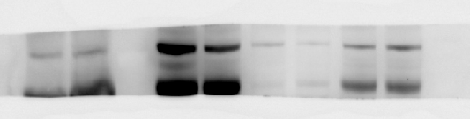

Supplement: Figure 7—figure supplement 1—source data 1. [file elife-83129-fig7-figsupp1-data1.zip › Figure supplement S6-source data 13/Raw blots of LIMK1.tif]

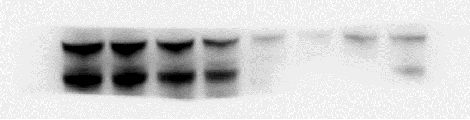

Supplement: Figure 7—figure supplement 1—source data 1. [file elife-83129-fig7-figsupp1-data1.zip › Figure supplement S6-source data 13/Raw blots of LIMK2.tif]

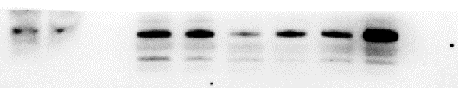

Supplement: Figure 7—figure supplement 1—source data 1. [file elife-83129-fig7-figsupp1-data1.zip › Figure supplement S6-source data 13/Raw blots of p-COFILIN.tif]
